# Serum albumin cysteine trioxidation is a potential oxidative stress biomarker of type 2 diabetes mellitus

**DOI:** 10.1038/s41598-020-62341-z

**Published:** 2020-04-15

**Authors:** Selvam Paramasivan, Sunil S. Adav, SoFong Cam Ngan, Rinkoo Dalan, Melvin Khee-Shing Leow, Hee Hwa Ho, Siu Kwan Sze

**Affiliations:** 1 School of Biological Sciences, Nanyang Technological University, 60 Nanyang Drive, Singapore, 637551 Singapore; 2 Department of Endocrinology, Tan Tock Seng Hospital, 11 Jalan Tan Tock Seng, Singapore, 308433 Singapore; 3 Cardiovascular and Metabolic Disorders Program, Duke-NUS Medical School, 8 College Road, Singapore, 169857 Singapore; 4 Department of Cardiology, Tan Tock Seng Hospital, 11 Jalan Tan Tock Seng, Singapore, 308433 Singapore

**Keywords:** Biomarkers, Mass spectrometry, Proteomic analysis

## Abstract

Metabolic disorders in T2DM generate multiple sources of free radicals and oxidative stress that accelerate nonenzymatic degenerative protein modifications (DPMs) such as protein oxidation, disrupt redox signaling and physiological function, and remain a major risk factor for clinical diabetic vascular complications. In order to identify potential oxidative biomarkers in the blood plasma of patients with T2DM, we used LC-MS/MS-based proteomics to profile plasma samples from patients with T2DM and healthy controls. The results showed that human serum albumin (HSA) is damaged by irreversible cysteine trioxidation, which can be a potential oxidative stress biomarker for the early diagnosis of T2DM. The quantitative detection of site-specific thiol trioxidation is technically challenging; thus, we developed a sensitive and selective LC-MS/MS workflow that has been used to discover and quantify three unique thiol-trioxidized HSA peptides, ALVLIAFAQYLQQC_(SO3H)_PFEDHVK (m/z 1241.13), YIC_(SO3H)_ENQDSISSK (m/z 717.80) and RPC_(SO3H)_FSALEVDETYVPK (m/z 951.45), in 16 individual samples of healthy controls (n = 8) and individuals with diabetes (n = 8). Targeted quantitative analysis using multiple reaction monitoring mass spectrometry revealed impairment of the peptides with *m/z* 1241.13, *m/z* 717.80 and *m/z* 951.45, with significance (P < 0.02, P < 0.002 and P < 0.03), in individuals with diabetes. The results demonstrated that a set of three HSA thiol-trioxidized peptides, which are irreversibly oxidatively damaged in HSA in the plasma of patients with T2DM, can be important indicators and potential biomarkers of oxidative stress in T2DM.

## Introduction

Protein damage by spontaneous nonenzymatic posttranslational modifications (PTMs) or degenerate protein modifications (DPMs), such as oxidation and glycation, has long been recognized as a mediator of various human degenerative diseases and natural aging. However, conventional biochemical methods could only quantify the total damaged proteins in biological or clinical samples. Recent advancements in LC-MS/MS-based proteomics technology have provided the necessary sensitive and specific methods to detect and quantify DPMs in precise amino acid residues in specific proteins and have therefore opened a new avenue to study protein damage in degenerative diseases and aging.

Type 2 diabetes mellitus (T2DM) is a chronic complex metabolic disorder characterized by hyperglycemia owing to either defects in insulin production or its action, which leads to impaired carbohydrate metabolism and extends its effects to almost every tissue and organ of the body through oxidative stress^1^. Oxidative stress is a hallmark of diseases associated with metabolic or vascular disorders. Importantly, T2DM is characterized by defects in both metabolic and vascular domains with multiple sources of free radicals, starting very early in the disease process that worsens over the course of the disease^2^. Studies have shown the occurrence of oxidative stress in the prediabetes stage^3,4^; thus, oxidative stress can predict the risk of diabetes at a very early stage. Notably, oxidative stress accelerates nonenzymatic DPMs, disrupts redox signaling and physiological function, and remains a major risk factor for clinical diabetic vascular complications such as cardiovascular disease, stroke, nephropathy, neuropathy and retinopathy^5^. However, measuring oxidative stress is challenging since reactive oxygen species (ROS) or other metabolic products are extremely unstable; hence, the gold standard to measure oxidative stress has not yet been established. As oxidative stress is a potential indicator to predict diabetes progression and is also beneficial for longitudinal follow-up to evaluate the response to treatment, there remains an unmet need to develop a reliable biomarker that can be used to estimate oxidative stress quantitatively.

Modified proteins are prospective biomarkers for both diagnoses and assessment of disease treatment; hence, the American Diabetes Association^6^ and the World Health Organization^7^ approved the use of glycated hemoglobin A1c (HbA1c) in diagnosing DM. Modified proteins such as plasma glycoproteins including human epidermal growth factor receptor 2 (HER2) in breast cancer, prostate-specific antigen (PSA) in prostate cancer, carcinoembryonic antigen (CEA) in colorectal cancer, cancer antigen 125 (CA-125) in ovarian cancer, alpha-fetoprotein in hepatocellular carcinoma have been approved by the Food and Drug Administration (FDA) as a potential diagnostic biomarker of various cancers^8^. Further, the role of DPMs, such as deamidation and citrullination, has been demonstrated in the pathogenesis of neurodegenerative diseases^9–14^. Restated, modified proteins play a key role as a potential biomarker and also in elucidating the mechanism of pathogenesis. In T2DM, the uptake of glucose by tissues is reduced, resulting in intracellular hypoglycemia and extracellular hyperglycemia. Although HbA1c is a good biomarker for measuring the average glucose level in patient plasma over the past few months, it may not be able to accurately predict clinical outcomes because diabetic vascular complication is a multifactorial disorder modifiable by glucose level, oxidative stress, lipid profile, environment and genetic factors. Hyperglycemia induces reactive oxygen species (ROS) accumulation that, in turn, induces endothelial dysfunction and cellular damage. Human serum albumin (HSA) is a major protein in the blood circulation, and the redox status of its thiol (SH) group plays the predominant role in redox regulation^15,16^, and it may accurately reveal oxidative stress and can act as a potential biomarker of oxidative stress and prognostic biomarker of diabetes^17^. Under physiological conditions, Cys34 of HSA exists in either the reduced or oxidized form. An equilibrium between these two forms totally depends on the redox state of Cys34, and their ratio indicates the disease state^18,19^. Oxidative stress induces the oxidation of cellular components, including protein oxidation, and the thiol (SH) group is vulnerable to such oxidative modifications. Cysteine (Cys) can be modified stepwise from monooxidation to dioxidation and finally to trioxidation. Cys monooxidation and dioxidation are reversible^20^, and such reversible oxidation by ROS is considered to be a key regulator of protein function^21^. Trioxidation of Cys residues is irreversible^20^ and generates sulfonic acid (Cys-SO_3_H) motifs that identify proteins subjected to extremely oxidative conditions. Moreover, conversion of the thiol group to strong sulfonic acid (Cys-SO_3_H) by oxidative stress can damage tissues. Thus, the accumulation of Cys-SO_3_H may be an oxidative biomarker predictive of disease progression. However, the detection and quantification of low-abundance peptides with trioxidized Cys residues in complex clinical samples are technically challenging.

Mass spectrometry-based proteomic techniques have the potential to profile quantitative abundances of proteins and to detect site-specific modifications quantitatively at a particular residue in a targeted protein; hence, they are emerging as a powerful technique in biomedical research. An MS-based approach for DM biomarkers discovery including increased levels of apolipoprotein C-III (Apoc-III), pancreatic polypeptide, apolipoprotein C-II, glucagon-like peptide 2, leukotactin-1, calcitonin, etc., has been proposed^22^. Further, Zhi *et al*.^23^ adopted proteomics coupled with an immune assay to identify DM-related biomarkers and found a significant increase in the expression level of adiponectin, insulin-like growth factor binding protein, C-reactive protein, and serum amyloid protein A in patients with DM. Decreased levels of transthyretin, hemoglobin α-chain and hemoglobin β-chain in plasma from subjects with diabetes have been documented^24^. An altered abundance of many of these proteins has also been reported in another disease; therefore, these proteins may not be specific and reliable biomarkers. HSA with site-specific Cys trioxidation identified in the plasma of individuals with T2DM may be a more specific and reliable biomarker of early oxidative damage in T2DM that may be a predictive biomarker for disease progression to diabetic vascular complications. In this study, using a discovery-based data-dependent LC-MS/MS proteomic approach, we detected and identified modified peptides in complex clinical plasma samples. After that, using a liquid chromatography-multiple reaction monitoring mass spectrometry (LC-MRM-MS)-based targeted approach to quantify irreversible oxidation at targeted cysteine, we quantified Cys trioxidation of HSA in patients with diabetes. The results indicated that site-specific trioxidized HSA could be a potential biomarker of diabetes diagnosis and prognosis.

## Results

### Subject characteristics and experimental strategy

**Figure 1:**
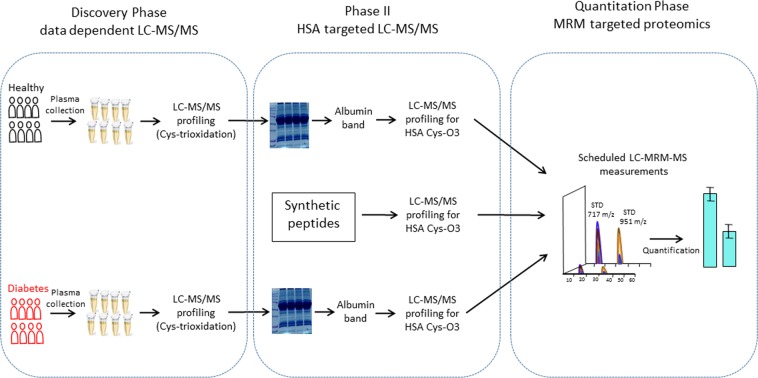
Schematic workflow showing three-phase experiments designed to identify, validate and quantify cysteine trioxidation. In the discovery phase, pooled plasma samples were subjected to proteomics profiling using data-dependent LC-MS/MS. Human serum albumin (HSA) was identified with significant Cys trioxidation. In phase II (HSA targeted phase), plasma samples were separated by SDS-PAGE, and albumin bands were excised for in-gel digestion and analyzed by LC-MS/MS to confirm the discovery phase results. In the quantitation phase, three HSA Cys-trioxidized peptides and their unmodified counter peptide pairs with strong signals were quantitatively measured using the MRM targeted proteomic method. The three peptide pairs were also synthesized and measured by both data-dependent MS/MS and MRM methods to confirm the positive identification of the trioxidation sites. The validated MRM method was used to quantitatively study HSA Cys trioxidation in plasma samples from patients with T2DM and healthy controls.

To systematically identify and verify Cys trioxidation of HSA, we analyzed 16 plasma samples collected from patients undergoing CCTA in the assessment of cardiac risk in the Department of Cardiology of Tan Tock Seng Hospital (TTSH). Samples were available from patients with T2DM (n = 8) and their sex-matched controls (n = 8) without DM. The mean age of the patients with DM was 63.75 ± 6.5 years, while the controls’ age was 55.5 ± 5.3 years. The level of glycated HbA1c was in the range of 7.7 to 10.9% among patients with DM, while it was less than 5% in controls. The clinical and biochemical characteristics of the subjects are tabulated in Table 1. The experimental design with a three-phase strategy including discovery, HSA targeted validation and quantitation phases allows for identification, verification and quantitation of Cys trioxidation of HSA is shown in Fig. 1. In the discovery phase, plasma samples from controls (n = 8) and patients with DM (n = 8) were pooled within the group, and the plasma proteome of both groups was profiled using discovery data-dependent LC-MS/MS analysis, which revealed significantly oxidized forms of HSA, particularly trioxidized cysteine, in patients with diabetes. In the second HSA targeted phase, to avoid the interference of other plasma proteins, we separated plasma proteins on SDS-PAGE, isolated the HSA protein band for in-gel digestion and profiled it using LC-MS/MS, which confirmed Cys trioxidation at 13 different sites of HSA (Supplementary Data S1a and S1b). The annotated MS/MS spectra of the detected unique HSA peptides with cysteine trioxidation in Mascot Peptides View are included in Supplementary Data S2, and the annotated MS/MS spectra of all three targeted peptides are shown in Supplementary Data S3. Isolation of the HSA band by SDS-PAGE increases sensitivity of detection and quantitation of HSA Cys-trioxidation in the clinical samples, but applied electric energy during electrophoresis may introduce oxidation in proteins. To assess whether the HSA Cys-trioxidation could be artificially generated during proteomic sample preparation we performed a control experiment using a pooled plasma sample that were subjected to three different proteomics sample preparation conditions. To determine if the thiol trioxidation can be induced by electrophoresis in SDS-PAGE separation, the plasma samples were run at 50 V or 100 V. The whole gel lane was excised and subjected to in-gel tryptic digestion. The sample was also independently prepared using in-solution digestion condition. Each proteomic sample preparation condition was carried out three times and each trypsin digested sample was injected eight times into LC-MS/MS. The acquired raw data were analyzed by PD2.2 for peptide identification and label-free quantitation using extracted ion chromatogram. Only peptides identified by PD2.2 with high confidence were used for further analysis to assess the oxidative protein damage in the samples prepared under different conditions (Supplementary Data S4). The results showed that the number and the total XIC area of identified peptides with Cys-trioxidation did not vary with different experimental conditions. To determine whether the level of HSA Cys-trioxidation are induced in other pathological conditions, we analyzed two sets of plasma samples from non-DM patients with cardiovascular disease and dementia. We did not observe similar increase in the HSA cysteine trioxidation. In the third phase, the HSA Cys-trioxidized peptides were quantitatively evaluated using the LC-MRM-MS targeted proteomics method. The HSA oxidized peptides were shortlisted for detailed quantitative MRM analysis only if both oxidized peptides and their matched unmodified counter peptides (carbamidomethylated by IAA during sample preparation) were quantitatively measurable with a strong signal using LC-MRM-MS. Then, the quantifiable trioxidized and carbamidomethylated peptide pairs were further confirmed and validated using synthetic peptides. Taken together, this study systematically identified and quantified Cys trioxidation of HSA as an oxidative stress biomarker of T2DM, and these modifications were further confirmed and validated by synthetic peptides.

**Table 1:** Clinical and biochemical parameters in healthy subjects and patients with disease.

No	Group	Sex	Age	HbA1c (%)
**Healthy**				
1	CONTROL-014	54	M	<5
2	CONTROL-063	53	M	<5
3	CONTROL-070	56	M	<5
4	CONTROL-088	53	M	<5
5	CONTROL-121	55	M	<5
6	CONTROL-126	68	F	<5
7	CONTROL-143	55	M	<5
8	CONTROL-151	50	M	<5
**Disease**				
1	PATIENT-006	69	M	7.7
2	PATIENT-007	67	M	9.9
3	PATIENT-016	65	M	9.1
4	PATIENT-059	59	M	7.8
5	PATIENT-077	59	M	7.8
6	PATIENT-106	67	M	7.6
7	PATIENT-127	52	F	10.9
8	PATIENT-149	72	M	7.9

HbA1c > 6.1% is considered diabetes according to the WHO criteria.

### Validation of HSA cysteine trioxidation

**Figure 2:**
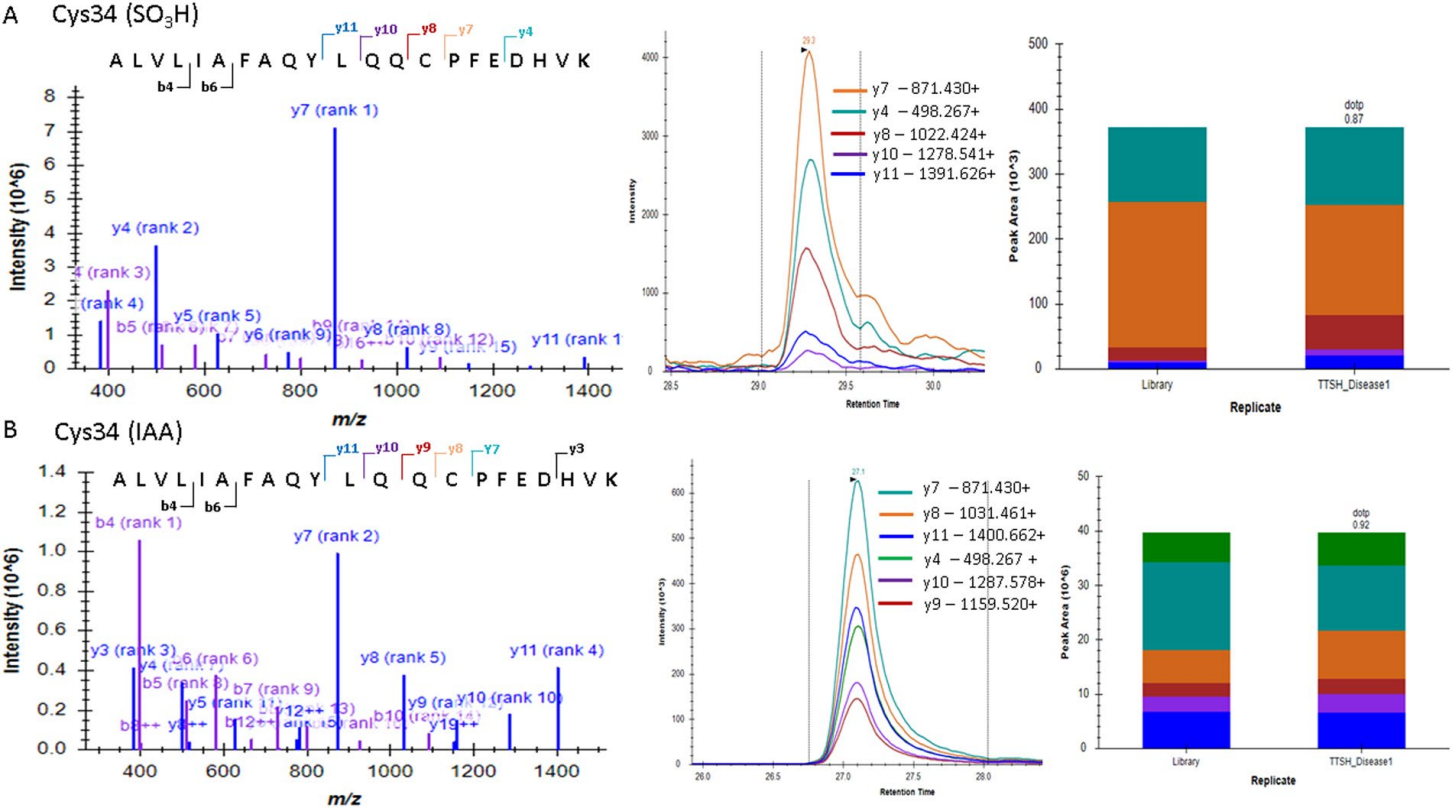
MS/MS spectrum of peptide ALVLIAFAQYLQQCPFEDHVK showing (**A**) trioxidation and (**B**) carbamidomethylation along with their fragmentation profiles. The Skyline dotp values of Cys34 trioxidation and unmodified counter peptides were 0.87 and 0.92, respectively, indicating high confidence identification and MRM quantitation of the peptides.

HSA was detected to have cysteine trioxidation at several residues in the discovery phase and was thus used as a model for investigating oxidative stress-induced redox-active cysteine modifications because this protein has been associated with insulin resistance and is susceptible to thiol-oxidation during oxidative stress^25^. HSA is the most abundant circulating protein in the plasma whose redox modifications modulate its physiological function. Due to the redox properties of its Cys34 thiol, plasma albumin displays an important antioxidant activity against oxidative damage and is considered a biomarker for oxidative stress. Among the different oxidation products of cysteine, sulfonic acid is considered the most highly oxidized and stable irreversible thiol, and its production and accumulation may be correlated with the extent of oxidative stress. Therefore, our focus was the identification and quantitation of Cys-SO_3_H in HSA. Based on the discovery and HSA targeted-phase data and peptide score, we identified 13 peptides that showed strong Cys trioxidation with good Mascot scores that were systematically evaluated for the extent of HSA Cys trioxidation in patients with T2DM and control subjects (Tables 2 and 3) using the MRM method. Three site-specific Cys trioxidations out of the 13 HSA oxidized peptides, including ALVLIAFAQYLQQC_(SO3H)_PFEDHVK (2+, m/z 1241.13), YIC_(SO3H)_ENQDSISSK (2+, m/z 717.80) and RPC_(SO3H)_FSALEVDETYVPK (2+, m/z 951.45), and their unmodified counter peptides were quantitatively measurable using LC-MRM-MS. The other 10 oxidized peptides identified in the discovery phase were not satisfactorily quantified by the MRM method due to relatively weak signals or missing matched unmodified counter peptides. Modifications such as trioxidation and carbamidomethylation (free cysteine modified by iodoacetamide IAA) of the same cysteine residue of the peptides need to be distinguished for quantitative analysis. Therefore, this study used synthetic peptides as a positive control to validate the annotated MS/MS spectra from clinical samples. Our results showed that the three pairs of targeted peptides have identical fragmentation patterns between tryptic peptides from plasma and synthetic peptides. The spectra of ALVLIAFAQYLQQC_(SO3H)_PFEDHVK (m/z 1241.13) and ALVLIAFAQYLQQC_(IAA)_PFEDHVK (m/z 1245.64) from patient plasma samples that differentiate Cys trioxidation and carbamidomethylation are shown in Fig. 2. The dotp value representing the relative abundance of each transition between MRM and the MS/MS spectra in the library adapted from the Mascot-searched DAT file was determined to be >0.87 in both oxidized and unmodified peptides, indicating high reliability of the peptide identification and MRM quantitation.

**Table 2:** Characteristics of the peptides selected for cysteine trioxidation and further MRM analysis.

SR. No	GS	Peptide sequence with trioxidation site(#)^a^	Charge state, z	Precursor ion m/z	Precursor ion mass error, ppm	Mascot score	pep_expect
1	ALB	K.ALVLIAFAQYLQQC#PFEDHVK.L	2	1241.128	−0.564	103.07	1.90E-08
2	ALB	K.SLHTLFGDKLC#TVATLR.E	2	962.0044	0.52	114.8	8.60E-10
3	ALB	K.QEPERNEC#FLQHKDDNPNLPR.L	3	876.4022	−1.18	104.96	2.10E-08
4	ALB	R.NEC#FLQHKDDNPNLPR.L	3	663.303	−1.057	116.97	6.80E-10
5	ALB	R.LVRPEVDVMC#TAFHDNEETFLK.K	3	881.0812	−1.704	76.52	1.70E-05
6	ALB	K.YIC#ENQDSISSK.L	2	717.8068	0.279	75.01	6.10E-06
7	ALB	K.LKEC#C#EKPLLEK.S	2	764.8658	−1.047	60.23	0.00028
8	ALB	K.SHC#IAEVENDEM#PADLPSLAADFVESK.D	3	1005.103	−5.079	67.46	6.10E-05
9	ALB	K.QNC#ELFEQLGEYKFQNALLVR.Y	2	1295.635	−0.657	120.69	4.10E-10
10	ALB	K.QNC#ELFEQLGEYK.F	2	824.8621	−0.971	78.46	1.10E-05
11	ALB	R.RPC#FSALEVDETYVPK.E	2	951.4517	−0.526	92.53	2.10E-07
12	ALB	K.EFNAETFTFHADIC#TLSEKER.Q	3	846.0486	0.592	70.59	3.90E-05
13	ALB	K.ADDKETC#FAEEGKK.L	3	540.2354	−1.051	104.82	5.80E-09

^a^The annotated MS/MS spectra in Mascot Peptide View of the individual peptides are shown in Supplemental Data S1 and S2.

**Table 3:** Peptides selected for cysteine carbamidomethylation and further MRM analysis to establish relative retention time.

SR. No	GS	Peptide sequence with trioxidation site(#)	Charge state, z	Precursor ion m/z	Precursor ion mass error, ppm	Mascot score	pep_expect
1	ALB	K.ALVLIAFAQYLQQC*PFEDHVK.L	2	1245.647	0.964	110.83	3.10E-09
2	ALB	K.SLHTLFGDKLC*TVATLR.E	2	966.5228	−1.657	133.43	9.00E-12
3	ALB	K.QEPERNEC*FLQHKDDNPNLPR.L	3	879.4144	−0.645	102.54	3.40E-08
4	ALB	R.NEC*FLQHKDDNPNLPR.L	3	666.3152	1.002	128.14	6.80E-11
5	ALB	R.LVRPEVDVMC*TAFHDNEETFLK.K	3	884.0934	−0.34	162.9	2.70E-14
6	ALB	K.YIC*ENQDSISSK.L	2	722.3252	0.277	96.98	4.50E-08
7	ALB	K.LKEC*C*EKPLLEK.S	2	769.3842	−2.017	97.05	8.40E-08
8	ALB	K.SHC*IAEVENDEM#PADLPSLAADFVESK.D	3	1008.115	−7.646	98.14	6.60E-08
9	ALB	K.QNC*ELFEQLGEYKFQNALLVR.Y	2	1300.153	−1.347	136.24	1.30E-11
10	ALB	K.QNC*ELFEQLGEYK.F	2	829.3805	−0.121	113.97	3.10E-09
11	ALB	R.RPC*FSALEVDETYVPK.E	2	955.97	−0.052	93.48	1.60E-07
12	ALB	K.EFNAETFTFHADIC*TLSEKER.Q	3	849.0609	−0.511	152.39	2.90E-13
13	ALB	K.ADDKETC*FAEEGKK.L	3	543.2477	1.045	130.24	2.20E-11

### Quantitative study of HSA Cys trioxidation in plasma samples using MRM

**Figure 3:**
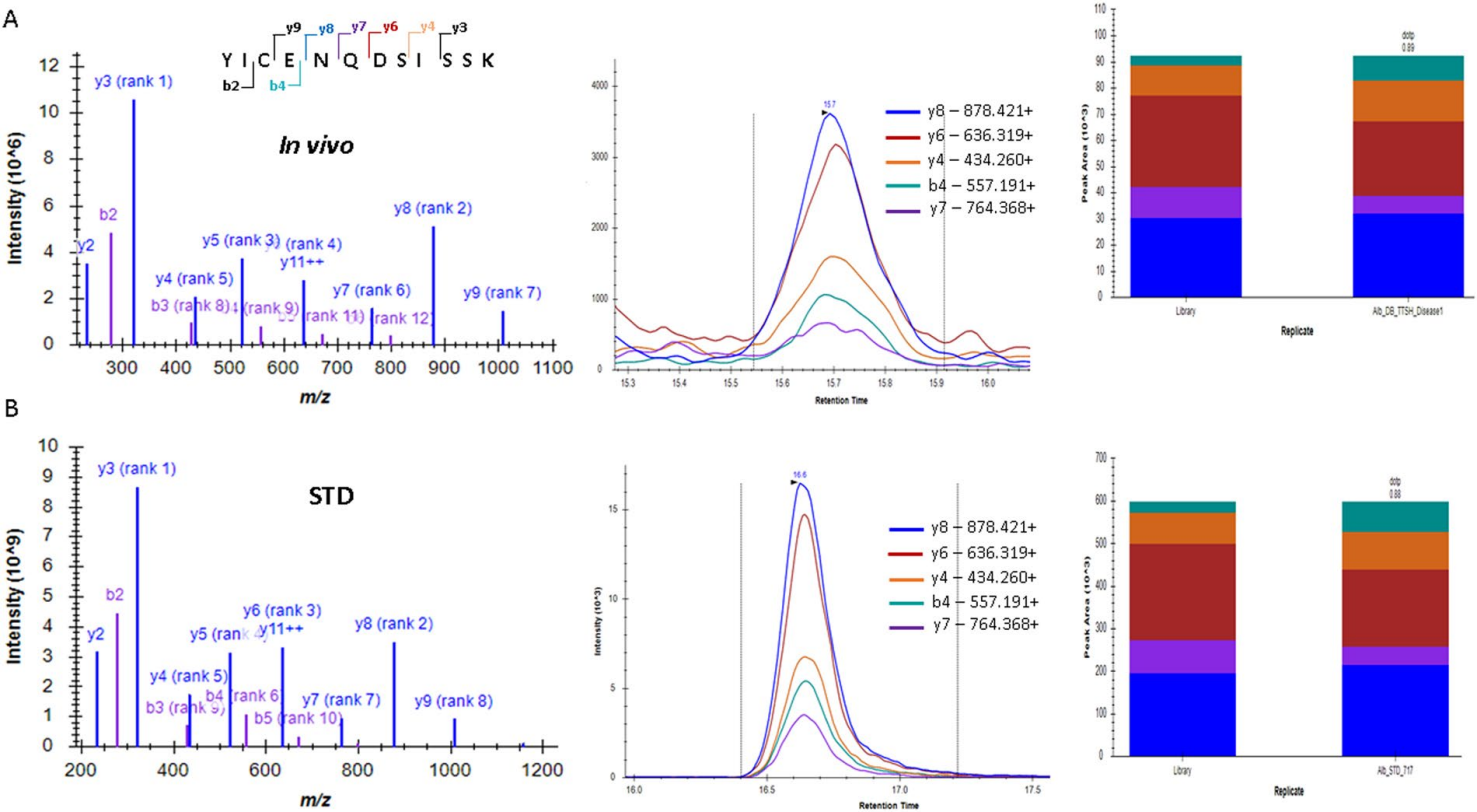
MRM MS/MS spectra, fragmentation profiles and Skyline dpot values of HSA Cys-trioxidized peptide, YIC_(SO3H)_ENQDSISSK. (**A**) Peptide identified in the plasma of patients with T2DM. (**B**) Results from synthetic peptide showing identical MS/MS fragmentation profile as the tryptic peptide from endogenous HSA.

**Figure 4:**
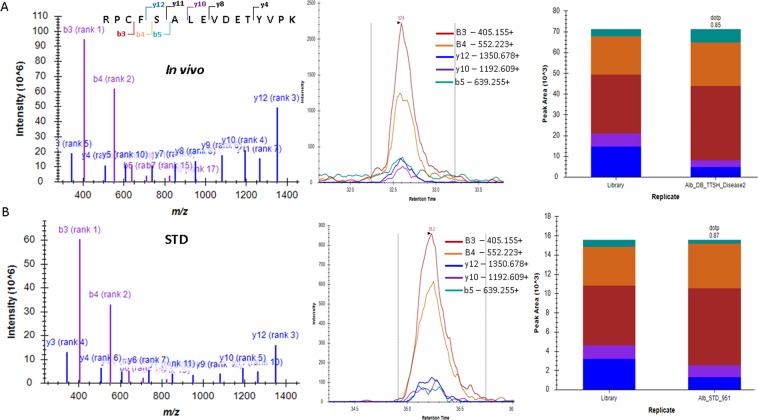
MRM MS/MS spectra, fragmentation profiles and Skyline dotp values of HSA Cys-trioxidized peptide, RPC_(SO3H)_FSALEVDETYVPK. (**A**) Peptide identified in the plasma of patients with T2DM. (**B**) Results from synthetic peptide showing identical MS/MS fragmentation profile as the tryptic peptide from endogenous HSA.

Intriguingly, peptides ALVLIAFAQYLQQCPFEDHVK with Cys34 from domain I, YICENQDSISSK with Cys265 from domain II and RPCFSALEVDETYVPK with Cys487 from domain III were trioxidized at the cysteine residues. The residue numbers stated here are from the secretory form. The detailed characteristics including the sequence with the site of modifications, peptide precursor ion m/z, charge state, collision energy, etc., are tabulated in Table S1 (Supplementary). Cys34 in the ALVLIAFAQYLQQCPFEDHVK peptide is the well-studied redox center of HSA and serves as a positive control, while Cys265 and Cys487 are newly identified as Cys trioxidation sites in this study. Cys trioxidation annotated MS/MS spectra of the peptides detected in plasma samples and the synthetic peptide YIC_(SO3H)_ENQDSISSK with their fragmentation profiles remain identical and are shown in Fig. 3. The MS/MS spectrum of the tryptic peptide YIC_(SO3H)_ENQDSISSK (m/z 717.80) from patient plasma identified crucial b3 ions of m/z 428.14 and a b4 ion of m/z 557.19, which sandwich the Cys trioxidation site. A mass shift of +47.98 Da to Cys-SO_3_H (sulfonic acid) was detected at Cys265 (Fig. 3A). The synthetic peptide YIC_(SO3H)_ENQDSISSK was detected with a fragmentation profile identical to a tryptic peptide (Fig. 3B), which suggests Cys trioxidation *in vivo*. The Skyline dotp value (>0.88) of the modified peptides in both tryptic plasma peptides and synthetic peptides shows a good match of the relative abundance of each fragmentation ion between the MRM experiment and library derived from the Mascot search results of data-dependent LC-MS/MS experiments. The matched unmodified (carbamidomethylation) YIC_(IAA)_ENQDSISSK peptide was also quantitatively measurable using the MRM method, as shown in Supplementary Fig. S3. The results indicated that the new Cys265 trioxidation in the YIC_(SO3H)_ENQDSISSK peptide was positively determined by both the discovery and validation phases, and the peptide can be accurately quantitatively measured by the MRM method. Similarly, Cys487 trioxidation was also positively identified and quantifiable using the MRM method. The MS/MS spectrum of tryptic peptide RPC_(SO3H)_FSALEVDETYVPK (m/z 951.45) from plasma samples (Fig. 4A) and the respective synthetic peptide (Fig. 4B) showed fragment b3, b4 and b5 ions of m/z 405.15, 552.22 and 639.25. A mass shift of +47.98 Da was detected at Cys487, indicating Cys trioxidation in domain III of HSA. Again, the Skyline dotp value (>0.9) of the modified RPC_(SO3H)_FSALEVDETYVPK peptide in both the tryptic plasma peptides and synthetic peptides showed a good match in the relative abundance of each fragmentation ion, as shown in Fig. 4. The matched unmodified (carbamidomethylation) RPC_(IAA)_FSALEVDETYVPK peptide was also quantitatively measurable using the MRM method, as shown in Supplementary Fig. S4. The results indicated that the new Cys487 trioxidation in the RPC_(SO3H)_FSALEVDETYVPK peptide was positively determined in both the discovery and validation phases, and the peptide can be accurately quantitatively measured by the MRM method.

**Figure 5:**
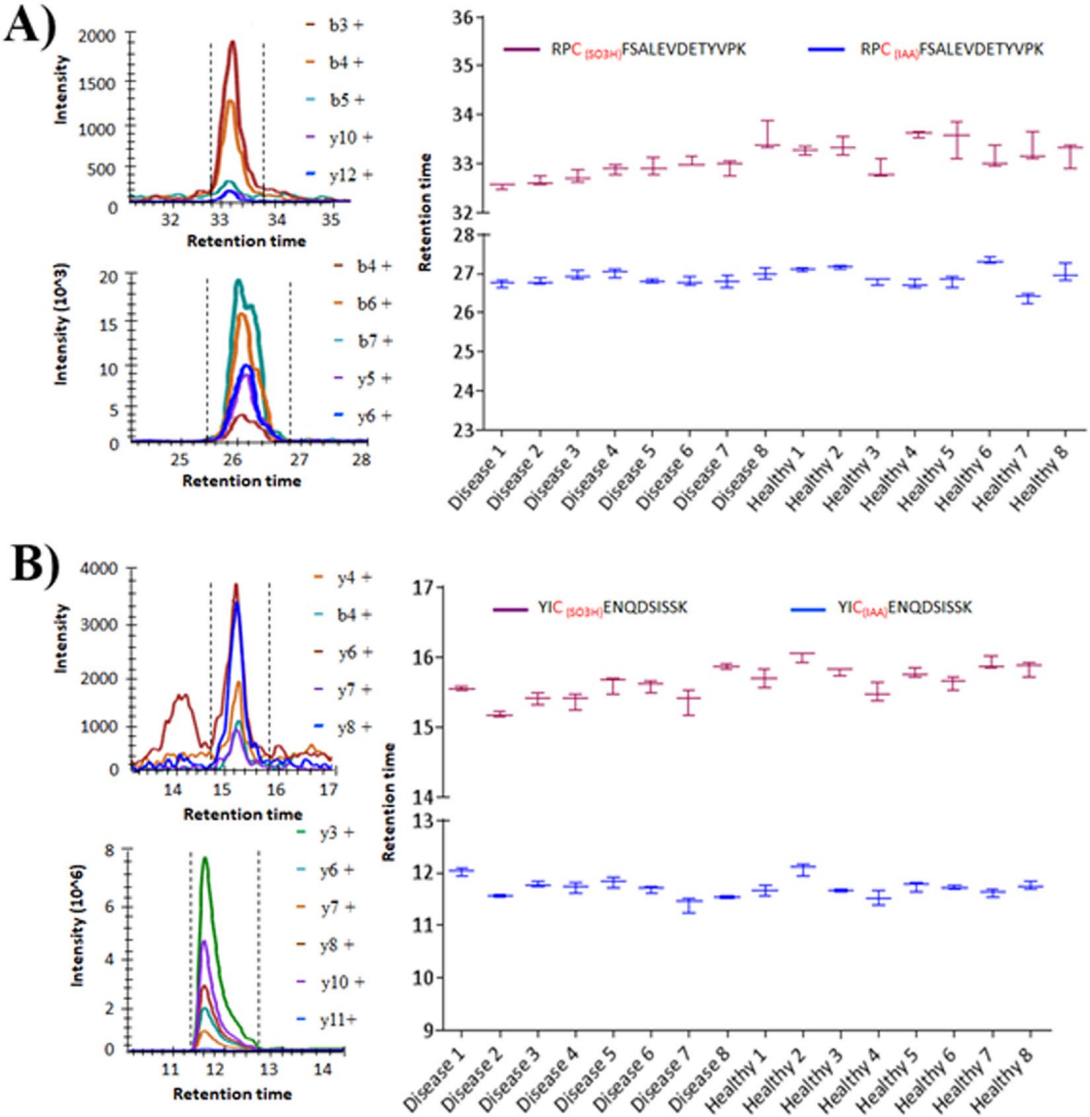
Retention time of LC separation of trioxidized and carbamidomethylated peptides. The unmodified (cysteine carbamidomethylation) and cysteine trioxidation peptides (in both **A**) RPCFSALEVDETYVPK and (**B**) YICENQDSISSK peptides) were well separated by reverse phase liquid chromatography using a C18 column. The retention times of the plasma tryptic peptides and the corresponding synthetic peptides were similar in the data-dependent LC-MS/MS. These retention times were then applied to the instrumental settings of the scheduled LC-MRM-MS. The variation of the retention time of the same peptide was less than 1 minute across 16 plasma samples.

**Figure 6:**
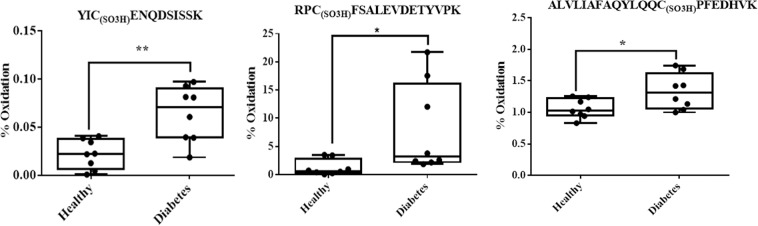
Quantitative cysteine trioxidation at different sites. Statistical significance is analyzed by one-way ANOVA.

After establishing proper separation, identification and validation of targeted peptides with Cys trioxidation and carbamidomethylation using synthetic peptides, we applied the instrumental settings established with synthetic peptides to study and quantify the extent of novel *in vivo* Cys trioxidation sites in the tryptic peptides RPCFSALEVDETYVPK (Fig. 5A) and YICENQDSISSK (Fig. 5B). The detailed retention times of trioxidized and carbamidomethylated peptides and their relative retention times were established and are shown in Supplementary Figs. S1 and S2. Quantitation of Cys trioxidation in healthy subjects and patients with diabetes was performed by the area under the curve (AUC) using Skyline software^26^, which revealed significantly higher Cys trioxidation in patients with diabetes when compared to that in controls (Fig. 6). The extent of cysteine oxidation varied with cysteine residue position and was highly significant in YIC_(SO3H)_ENQDSISSK (*P* < *0.002*), RPC_(SO3H)_FSALEVDETYVPK (*P* < *0.03*) and ALVLIAFAQYLQQC_(SO3H)_PFEDHVK (*P* < *0.02*), as analyzed by ANOVA. Thus, adopting three unique peptides, this study established a quantitative relationship between healthy controls (n = 8) and individuals with diabetes (n = 8) and found significantly higher Cys trioxidation in patients with diabetes. This implies that plasma Cys trioxidation through the irreversible form of Cys thiol trioxidation is an important modification that accurately reflects oxidative stress and can be a potential prognostic biomarker of DM. This study is the first to establish the formation of Cys-SO_3_H at Cys265 and Cys487 of HSA.

## Discussion

The present study demonstrated a systematic identification of Cys trioxidation of HSA and established a quantitative relationship between individuals with DM and healthy individuals, which revealed significant thiol trioxidation of HSA in individuals with diabetes. As the most abundant plasma protein with 35 cysteine residues, Cys34, which is sensitive to ROS, is well studied for its different oxidation states, including trioxidation. Oxidized HSA can reflect oxidative stress accurately, and hence, it could be a diagnostic biomarker for pre-DM and a prognostic biomarker for DM clinical outcomes. In addition to hyperglycemia-induced oxidative stress, reactive electrophiles, including aldehydes, epoxides, quinones, and short-lived oxygen and nitrogen species, enter the bloodstream and promote oxidation of proteins and lipids, oxidation of glucose, metabolism of exogenous compounds and inflammation associated with disease^27,28^. Owing to the short lifespan of these reactive electrophiles, estimating their concentration in body fluid is challenging. Since the redox-active cysteine of HSA is a highly reactive sulfhydryl and remains the main target of reactive oxygen species (ROS) and electrophiles, the oxidative products formed by their reactions with blood nucleophiles such as HSA can be measured. The oxidative product depends on the degree of oxidation state in redox-active Cys34 residues, which in turn depends on the magnitude of the oxidative stress. Cys34 can be oxidized to sulfenic (Cys-SOH), sulfinic (Cys-SO_2_H), and sulfonic acids (Cys-SO_3_H) and disulfide bonds (Cys-SS-Cys) and can alter and regulate the biological function of the protein^15,29^. Sulfonic acid, a stable product of cysteine-trioxidized HSA, is an ideal candidate protein to evaluate oxidative stress in DM due to the less invasive nature of obtaining plasma from patients with diabetes for analysis. Using an electrospray ionization time-of-flight mass spectrometer (ESI-TOF-MS), an increased cysteinylated Cys34-HSA in chronic liver and kidney diseases and DM is documented^17^. Unfortunately, except for Cys34, little is known about Cys trioxidation of HSA, including which cysteines are prone to trioxidation, the impact of site-specific Cys trioxidation on physiological processes during disease, the progression of the disease or the response to treatment.

The significantly higher sensitivity of MRM analysis permits evaluation and validation of low abundance site-specific cysteine trioxidation in a targeted protein. As far as the authors know, this is the first report that systematically established an LC-MRM-MS technique to identify novel modifications such as Cys trioxidation of HSA quantitatively in DM plasma. The concentration of endogenous free radicals depends on multiple factors, including environmental stress and genetic background. Free radicals are also generated during autoxidation of glucose, nonenzymatic glycation of proteins and increased lipid peroxidation in diabetes^30^, which results in an elevated level of oxidative stress leading toward an increased generation of glycoxidation products, including HbA1c, above the benchmark plasma value (<5%). Although HbA1c is a good biomarker for measuring the average glucose level in patient plasma over the past few months, it may not be able to accurately predict clinical outcomes because diabetic vascular complication is a multifactorial disorder modifiable by environmental stress and genetic background. Therefore, oxidative stress can potentially predict the clinical risk of DM at an earlier stage than elevated HbA1c, and it has the potential to be a key early stage prognostic biomarker of DM. Support also comes from the study by Styskal *et al*.^31^, who observed oxidative damage to DNA in pre-DM, even before the clinical development of DM.

The comprehensive systematic approach, including the identification of Cys trioxidation in the discovery phase, further confirmation in the targeted phase, as well as validation, allowed us to establish a quantitative relationship of such novel modifications in healthy subjects and patients with diabetes. When we estimated Cys trioxidation at Cys34 in individuals with diabetes, it was significantly higher than that in the controls. Cys34 is the only well-studied cysteine trioxidation of HSA, and it has been identified as a metal-binding site^32^. Although Cys34 trioxidation is relatively well-studied, intriguingly, this study for the first time identified significant trioxidation (as evaluated by ANOVA) at Cys265 and Cys487 residues in individuals with diabetes. The statistical analysis (ANOVA) revealed that trioxidation at Cys265 (p < 0.002) was more significant than that at Cys34 (p < 0.02). Thus, Cys265 of HSA may be more sensitive to oxidative stress and has the potential to evolve as a key biomarker. The oxidative damage at Cys265 and Cys487 of HSA may be induced by an upsurge of oxidative stress in DM. The damaged cysteine residues may result in increased exposure of HSA, enhanced accessibility to oxidative damage and increased risk of structural destabilization. HSA is known for its multiple functions, such as maintenance of colloid osmotic pressure, transport of endogenous and exogenous metabolites, including steroids, fatty acids, bilirubin, tryptophan, and hemin, and it also acts as an antioxidant by scavenging radicals^33^. The site-specific Cys trioxidation of HSA may hamper these processes. Moreover, the accumulation of strong sulfonic acid (Cys-SO_3_H) in proteins can potentially damage surrounding tissues. According to Jalan *et al*.^34^, the loss of ligand binding potential of albumin is associated with an increased mortality of patients with decompensated cirrhosis. Recently, Oettl *et al*.^35^ reported that in advanced liver disease, oxidative damage impairs the binding characteristics of HSA. The binding of endogenous substances (bilirubin and tryptophan) and drugs (warfarin and diazepam) to HSA purified from patients with chronic liver disease revealed altered binding ability due to oxidized Cys34^17^. It has been shown that the antioxidant property of albumin was altered following *in vitro* glycation^36^ and in patients with diabetes^37^. Hence, estimating the redox status of the cysteines of HSA may provide important information in both pre-DM and DM and shed light on the disease severity and degree of organ damage.

## Conclusion

Protein damage by nonenzymatic posttranslational modifications or degenerate protein modifications, such as oxidation and glycation, has long been recognized as a mediator of various degenerative diseases and natural aging. However, conventional biochemical methods could only quantify the total damaged proteins in biological or clinical samples. Recent advancements in LC-MS/MS-based proteomics technology have provided the necessary sensitive and specific methods to detect and quantify DPMs in precise amino acid residues in specific proteins and have opened a new avenue to study protein damage in degenerative diseases and aging. The redox state of HSA, as a biomarker of oxidative stress, has gained potential prognostic value in diabetes vascular complications. Owing to technical challenges in identifying and quantifying DPMs in complex clinical samples, Cys trioxidation of HSA has rarely been documented. This study developed an LC-MRM-MS-based mass spectrometry method to identify modified peptides quantitatively in complex clinical samples. In this study, thiol-trioxidized albumin peptides in *in vitro* synthetic peptides and *in vivo* samples were identified and quantified by adopting a three-phase systematic approach, including discovery, targeted and validation phases, which quantitatively established the Cys trioxidation profile in 16 individual samples and revealed impaired plasma albumin in individuals with diabetes. In this study, we found that the % of trioxidation in the three MRM quantifiable peptides was not correlated with the concentration of HbA1c in patients with T2DM. Although HbA1c is a good biomarker for measuring the average glucose level in patient plasma over 3–6 months, it may not be able to accurately predict clinical outcomes because diabetic vascular complication is a multifactorial disorder modifiable by the glucose level, oxidative stress, environmental and genetic factors. Therefore, oxidative stress biomarkers, glycated proteins and advanced glycation end products are likely complementary biomarker signature for predicting the clinical course of the disease. The biomarker signature has the potential to be a key early stage prognostic biomarker of DM. Thus, the detailed site-specific Cys trioxidation, its impact on the structure and possible quantitative relationships with the severity and outcomes of the disease need to be established in a longitudinal study with a larger cohort.

## Materials and Methods

### Subjects and samples

Patients who underwent cardiac computed tomographic angiography (CCTA) in the assessment of cardiac risk in the Department of Cardiology at Tan Tock Seng Hospital (TTSH) were recruited for this study. The study was approved by the institutional review board of TTSH and Nanyang Technological University (NTU). Experimental procedures conformed to the tenets of the Declaration of Helsinki. Prior to participation in the study, informed consent was taken from eligible subjects on the day of recruitment. A detailed history, clinical examination, and blood tests for HbA1c and CCTA were performed. Patient plasma was isolated from whole blood that was collected into EDTA tubes on the day of the CT scan. The study incorporated sixteen enrolled patients. Eight patients had DM as defined by the criteria of the World Health Organization, and their glycosylated hemoglobin (HbA1c) levels were retrieved and are tabulated in Table 1. Healthy individuals were defined as those with HbA1c < 6.0%. Three pairs of targeted peptides, ALVLIAFAQYLQQCPFEDHVK, YICENQDSISSK and RPCFSALEVDETYVPK, were synthesized by GL Biochem Ltd (Shanghai, China).

### Discovery proteomic phase using LC-MS/MS

Discovery proteomic experiments were performed using pooled plasma samples from a group of patients with T2DM and matched healthy controls. Plasma samples were purified by cold (−20 °C) acetone precipitation and redissolved in a sodium deoxycholate ammonium acetate buffer for reduction, alkylation and trypsin in solution digestion overnight^38^. The tryptic peptides were then fractionated using the ERLIC chromatography method as previously described^39^. Each fraction of the plasma tryptic peptides and three pairs of synthetic targeted peptides were separated and analyzed on a Dionex Ultimate 3000 RSLC NanoLC system coupled to a Q-Exactive tandem mass spectrometer (Thermo Fisher, MA) LC-MS/MS system as previously described^9^. Five microliters of peptide sample (~2 µg of tryptic peptides, or 1 pg of synthetic peptides) was injected into an Acclaim peptide trap using the autosampler of the NanoLC system. A 60-min LC-gradient was used to separate the tryptic peptides in a Dionex EASY-spray C18 column (PepMap C18, 3 µm, 100 A) using mobile phase A (0.1% FA in 3% ACN) and mobile phase B (0.1% FA in ACN) at a flow rate of 300 nl/min. Injected peptides were analyzed using an EASY nanospray source at an electrospray potential of 1.5 kV. To identify peptides and posttranslational modifications, the mass spectrometer was operated in positive mode using data-dependent acquisition of MS/MS. A full MS scan (350-1,600 m/z range) was acquired at a resolution of 70,000 at m/z 200 with a maximum ion accumulation time of 100 ms. The 10 most intense peptide ions were fragmented by higher energy collisional dissociation (HCD) with normalized collision energy of 28% and MS/MS spectra were recorded at a resolution of 17,500 at m/z 200. The automatic gain control (AGC) settings of the full MS scan and the MS/MS scan were 3E6 and 2E5, respectively. An isolation width of 2 was used for MS/MS. Fifteen seconds was set as dynamic exclusion. Single and unassigned charged ions were excluded from MS/MS.

Raw data were converted to Mascot generic file format using Proteome Discoverer (PD) v1.4 (Thermo Scientific, San Jose, USA) with deisotope and deconvolution in MS/MS spectra. A protein sequence database search was performed against the Uniprot human database (downloaded on February 6th, 2017; containing a total of 178,750 sequences and 61,972,042 residues) using the Mascot server version 2.6.02 (Matrix Science, London, UK). The search parameters were set as enzyme: trypsin; maximum miss cleavage: 2; precursor mass tolerance: 10 ppm; fragment mass tolerance: 30 ppm; # ^13^C: 2; variable modifications: carbamidomethyl (C) and trioxidation (CMWY). The identified peptides with Mascot pep-expect <0.05 were exported to Microsoft Excel or processed using an in-house script for further analysis. Peptides with Cys trioxidation were shortlisted for further analysis. Annotated MS/MS spectra were extracted from Mascot server as Mascot Peptide View and included as Supplemental Data S1 and S2.

### Targeted proteomics analysis using multiple reaction monitoring MS

Quantitative analysis of targeted thiol-trioxidized peptides was carried out in 16 individual samples (8 control vs 8 T2DM) as listed in Table 1. Individual plasma proteins (100 µg) were separated on a 12% SDS-PAGE gel, and protein bands were visualized by staining with Coomassie blue (Supplementary Fig. S5). The albumin band was excised, cut into small pieces of approximately 1 mm^2^ and destained completely. The gel pieces were reduced with 10 mM DDT for 60 min at 37 °C, alkylated using 55 mM iodoacetamide (IAA), dehydrated with 100% acetonitrile and then subjected to trypsin digestion with sequencing-grade modified trypsin (Promega, Madison, WI) at 37 °C overnight. The peptides were extracted from the gel, vacuum dried, and reconstituted in 0.1% formic acid for LC-MRM-MS analysis. Three pairs of targeted peptides were examined in 16 individual plasma samples together with the synthetic peptides using LC-MRM-MS analysis. Both modified (trioxidized) and unmodified (IAA alkylated) counter peptides and the transitions for targeted peptides were extracted from the MS/MS data derived from the discovery LC-MS/MS spectra described above. LC-MRM-MS analysis was performed on a TSQ Vantage triple quadrupole mass spectrometer coupled to a Dionex Ultimate 3000 RSLC NanoLC system (Thermo Scientific Inc., Bremen, Germany). Peptides were injected onto an peptide trap (Acclaim PepMap100, 75 μm x 2 cm; C18, 3 μm, 100 Å) and resolved on an C18 column (Acclaim PepMap RSLC column, 75 μm x 15 cm; C18, 2 μm, 100 Å) (Thermo Scientific, USA). A 60-min gradient was established using mobile phase A (0.1% FA in HPLC water) and mobile phase B (0.1% FA in acetonitrile) at a flow rate of 300 nL/min identical to the discovery phase. The TSQ Vantage was set to perform MRM data acquisition in positive ion mode. An electrospray potential of 1.5 kV, a capillary temperature of 250 °C and a collision gas pressure of argon for Q2 set at 1.2 mTorr were used. The selectivity for both Q1 and Q3 was set to 0.8 Da. Each sample was analyzed in triplicate (three injections). For quantitative interpretation of the MRM data, we adapted a platform–independent software package. Skyline v4.1 was applied to extract quantitative information from multiple LC-MRM-MS raw data^26^. For the verification of peptides, at least 3 transitions coeluting within the predicted time window were considered positive identification of the targeted peptides. The dotp value representing the relative abundance of each transition was set with a threshold >0.85. For the quantitative study of peptides, Skyline ranked the y and b fragmentation ions from the targeted peptide based on transition intensity. The most intense transitions from the coeluting peak were selected for calculating the peak area with the following criteria: precursor charge state 2, 3, 4, 5; ion charge state 1, 2; ion type b, y; and always select N-terminal to proline for special ions. Skyline extracted the area under the ion chromatogram curve through the duration of eluting by interpolating peak height between the raw data points. The extracted area of selected transitions was summed to generate the peptide area.

### Control experiments: plasma sample processed by in-gel and in-solution digestions

Because thiol trioxidation may be generated during proteomic sample preparation, particularly during electrophoresis as electric current may introduce oxidation in protein, a set of independent control experiments was performed with a pooled plasma sample using in-gel and in-solution digestion conditions. Plasma samples were separated by SDS-PAGE at 50 V or 100 V. The whole gel lane of each sample was then subjected to in-gel digestion as described in the targeted proteomic analysis section above. In-solution digestion of the plasma sample was the same as described in the section above describing the discovery proteomic phase using LC-MS/MS. Each experimental condition was performed in three biological replicates, and each tryptic peptide sample was injected eight times into Q-Exactive mass spectrometry for LC-MS/MS analysis. The raw data were searched against the Uniport human database using Proteome Discoverer version 2.2 (PD2.2). Label-free quantitation of each identified peptide was measured using extracted ion chromatogram (XIC) by PD2.2. The number of Cys-trioxidized peptides and their total XIC area were used to determine the extent of protein oxidative damage in each condition.

### Statistical analysis

All data are expressed as the mean ± SD. The statistical significance of differences between groups was examined through one-way ANOVA. Differences were considered significant at *P* < *0.05*. The statistical analysis was performed using GraphPad Prism version 7.00 for Windows, GraphPad Software, La Jolla California USA (www.graphpad.com).

## Supplementary information

Supplementary Information.

Supplementary Information 2.

Supplementary Information 3.

Supplementary Information 4.

Supplementary Information 5.

## Data Availability

Data are available via ProteomeXchange with identifier PXD012667.
