# Supplementary material for: Serum albumin cysteine trioxidation is a potential oxidative stress biomarker of type 2 diabetes mellitus
Source: Sci Rep. 2020 Apr 15;10:6475. doi: 10.1038/s41598-020-62341-z (PMC7160123; doi:10.1038/s41598-020-62341-z)
Supplement: Supplementary file 2 — Supplementary Information 2. [file 41598_2020_62341_MOESM2_ESM.pdf]

Serum albumin cysteine trioxidation is a potential oxidative stress biomarker of type 2 diabetes mellitus

Selvam Paramasivan, Sunil S Adav, SoFong Cam Ngan, Rinkoo Dalan, Melvin Khee-Shing Leow, Hee Hwa Ho, Siu Kwan Sze*

ALB_K.SHC#IAEVENDEMPADLPSLAADFVESK.D_136.16
ALB_K.QNC#ELFEQLGEYKFQNALLVR.Y_120.69
ALB_R.NEC#FLQHKDDNPNLPR.L_116.97
ALB_K.SLHTLFGDKLC#TVATLR.E_114.80
ALB_K.SHC#IAEVENDEMPADLPSLAADFVESK.D_105.88
ALB_K.QEPERNEC#FLQHKDDNPNLPR.L_104.96
ALB_K.ADDKETC#FAEEGKK.L_104.82
ALB_K.ALVLIAFAQYLQQC#PFEDHVK.L_103.07
ALB_K.QNC#ELFEQLGEYKFQNALLVR.Y_95.85
ALB_R.LVRPEVDVMC#TAFHDNEETFLKK.Y_93.52
ALB_R.RPC#FSALEVDETYVPK.E_92.53
ALB_R.FKDLGEENFKALVLIAFAQYLQQC#PFEDHVK.L_92.52
ALB_K.ALVLIAFAQYLQQC#PFEDHVK.L_86.66
ALB_K.QNC#ELFEQLGEYK.F_78.46
ALB_R.LVRPEVDVMC#TAFHDNEETFLK.K_76.52
ALB_K.SLHTLFGDKLC#TVATLR.E_75.36
ALB_K.YIC#ENQDSISSK.L_75.01
ALB_K.ADDKETC#FAEEGKK.L_72.68
ALB_K.EFNAETFTFHADIC#TLSEKER.Q_70.59
ALB_R.RPC#FSALEVDETYVPK.E_69.99
ALB_K.SHC#IAEVENDEM#PADLPSLAADFVESK.D_67.46
ALB_K.LKEC#C#EKPLLEK.S_60.23
ALB_R.FKDLGEENFKALVLIAFAQY#LQQC#PFEDHVK.L_59.17
ALB_K.SHC#IAEVENDEM#PADLPSLAADFVESK.D_58.72
ALB_R.LVRPEVDVM#C#TAFHDNEETFLKK.Y_56.30
ALB_K.C#C#TESLVNR.R_54.80
ALB_K.DLGEENFKALVLIAFAQYLQQC#PFEDHVK.L_53.28
ALB_K.SHC#IAEVENDEM#PADLPSLAADFVESK.D_51.02
ALB_K.ADDKETC#FAEEGK.K_50.71
ALB_R.VTKC#C#TESLVNR.R_49.92
ALB_R.LVRPEVDVM#C#TAFHDNEETFLKK.Y_47.51
ALB_K.SLHTLFGDKLC#TVATLR.E_46.50
ALB_R.LKC#ASLQKFGER.A_45.81
ALB_K.EFNAETFTFHADIC#TLSEK.E_44.89
ALB_K.VFDEFKPLVEEPQNLIKQNC#ELFEQLGEYK.F_43.92
ALB_K.SHC#IAEVENDEM#PADLPSLAADFVESK.D_43.64
ALB_K.YIC#ENQDSISSKLK.E_42.96
ALB_K.LKEC#C#EKPLLEKSHC#IAEVENDEM#PADLPSLAADFVESK.D_41.96
ALB_K.C#ASLQKFGER.A_36.32

MATRIX SCIENCE Mascot Search Results

Peptide View

MS/MS Fragmentation of **SHCIAEVENDEMPADLPSLAADFVESK**
Found in **sp|P02768|ALBU_HUMAN**, Serum albumin OS=Homo sapiens GN=ALB PE=1 SV=2

Match to Query 12981: 2964.295748 from(1483.155150,2+) intensity(4313752.5000) scans(16104) rtinseconds(3149) index(12883)
Title: 150801_TTSH_Patient_Plasma_76_Spectrum028044_scans__16104_RTINSECONDS=3149
Data file L:\\Ard_TTSH\\T1D\\T150801_TTSH_Patient_Plasma_76.mgf

Click mouse within plot area to zoom in by factor of two about that point
Or, Plot from 200 to 3000 Da Full range
Label all possible matches ○ Label matches used for scoring ⦿

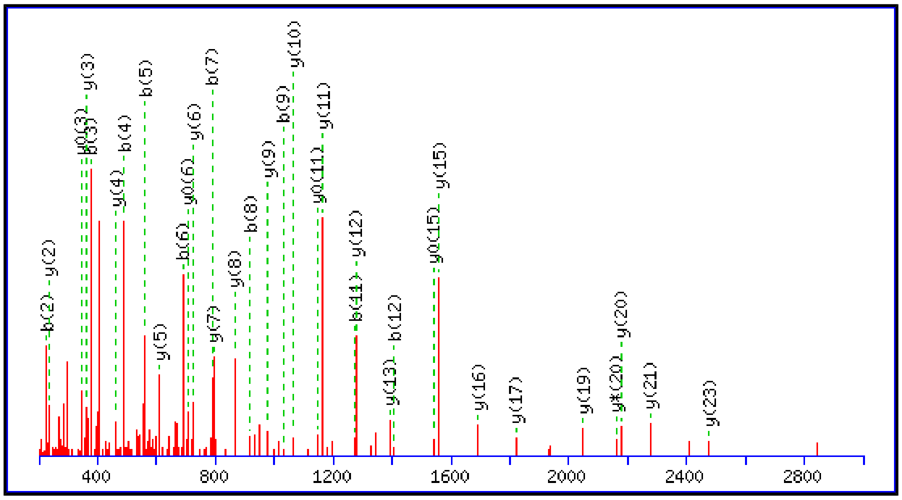

Monoisotopic mass of neutral peptide Mr(calc): 2964.3004
Variable modifications:
C3 : 4Trioxidation (CMWY)
Ions Score: 136 **Expect:** 1.2e-011
Matches : 34/294 fragment ions using 58 most intense peaks (help)

#	b	b++	b*	b*++	b0	b0++	Seq.	y	y++	y*	y*++	y0	y0++	#
1	88.0393	44.5233			70.0287	35.5180	S							27
2	225.0982	113.0527			207.0877	104.0475	H	2878.2757	1439.6415	2861.2492	1431.1282	2860.2652	1430.6362	26
3	376.0921	188.5497			358.0816	179.5444	C	2741.2168	1371.1120	2724.1903	1362.5988	2723.2063	1362.1068	25
4	489.1762	245.0917			471.1656	236.0865	I	2590.2229	1295.6151	2573.1963	1287.1018	2572.2123	1286.6098	24
5	560.2133	280.6103			542.2028	271.6050	A	2477.1388	1239.0731	2460.1123	1230.5598	2459.1283	1230.0678	23
6	689.2559	345.1316			671.2454	336.1263	E	2406.1017	1203.5545	2389.0752	1195.0412	2388.0911	1194.5492	22
7	788.3243	394.6658			770.3138	385.6605	V	2277.0591	1139.0332	2260.0326	1130.5199	2259.0486	1130.0279	21
8	917.3669	459.1871			899.3564	450.1818	E	2177.9907	1089.4990	2160.9642	1080.9857	2159.9801	1080.4937	20
9	1031.4099	516.2086	1014.3833	507.6953	1013.3993	507.2033	N	2048.9481	1024.9777	2031.9216	1016.4644	2030.9375	1015.9724	19
10	1146.4368	573.7220	1129.4102	565.2088	1128.4262	564.7168	D	1934.9052	967.9562	1917.8786	959.4430	1916.8946	958.9509	18
11	1275.4794	638.2433	1258.4528	629.7301	1257.4688	629.2380	E	1819.8782	910.4428	1802.8517	901.9295	1801.8677	901.4375	17
12	1406.5199	703.7636	1389.4933	695.2503	1388.5093	694.7583	M	1690.8357	845.9215	1673.8091	837.4082	1672.8251	836.9162	16
13	1503.5726	752.2900	1486.5461	743.7767	1485.5621	743.2847	P	1559.7952	780.4012	1542.7686	771.8879	1541.7846	771.3959	15
14	1574.6097	787.8085	1557.5832	779.2952	1556.5992	778.8032	A	1462.7424	731.8748	1445.7159	723.3616	1444.7318	722.8696	14
15	1689.6367	845.3220	1672.6101	836.8087	1671.6261	836.3167	D	1391.7053	696.3563	1374.6787	687.8430	1373.6947	687.3510	13
16	1802.7208	901.8640	1785.6942	893.3507	1784.7102	892.8587	L	1276.6783	638.8428	1259.6518	630.3295	1258.6678	629.8375	12
17	1899.7735	950.3904	1882.7470	941.8771	1881.7630	941.3851	P	1163.5943	582.3008	1146.5677	573.7875	1145.5837	573.2955	11
18	1986.8055	993.9064	1969.7790	985.3931	1968.7950	984.9011	S	1066.5415	533.7744	1049.5150	525.2611	1048.5310	524.7691	10
19	2099.8896	1050.4484	2082.8631	1041.9352	2081.8790	1041.4432	L	979.5095	490.2584	962.4829	481.7451	961.4989	481.2531	9

**20**	2170.9267	1085.9670	2153.9002	1077.4537	2152.9162	1076.9617	**A**	***866.4254***	433.7164	849.3989	425.2031	848.4149	424.7111	**8**
**21**	2241.9638	1121.4856	2224.9373	1112.9723	2223.9533	1112.4803	**A**	***795.3883***	398.1978	778.3618	389.6845	777.3777	389.1925	**7**
**22**	2356.9908	1178.9990	2339.9642	1170.4858	2338.9802	1169.9937	**D**	***724.3512***	362.6792	707.3246	354.1660	706.3406	353.6740	**6**
**23**	2504.0592	1252.5332	2487.0326	1244.0200	2486.0486	1243.5280	**F**	***609.3243***	305.1658	592.2977	296.6525	591.3137	296.1605	**5**
**24**	2603.1276	1302.0674	2586.1011	1293.5542	2585.1170	1293.0622	**V**	***462.2558***	231.6316	445.2293	223.1183	444.2453	222.6263	**4**
**25**	2732.1702	1366.5887	2715.1437	1358.0755	2714.1596	1357.5835	**E**	***363.1874***	182.0974	346.1609	173.5841	345.1769	173.0921	**3**
**26**	2819.2022	1410.1048	2802.1757	1401.5915	2801.1917	1401.0995	**S**	***234.1448***	117.5761	217.1183	109.0628	216.1343	108.5708	**2**
**27**							**K**	147.1128	74.0600	130.0863	65.5468			**1**

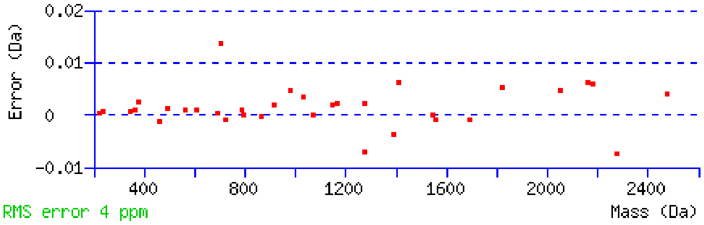

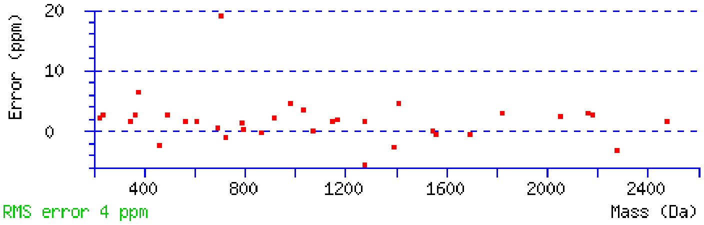

NCBI **BLAST** search of SHCIAEVENDEMPADLPSLAADFVESK
(Parameters: blastp, nr protein database, expect=20000, no filter, PAM30)
Other BLAST web gateways

All matches to this query

Score	Mr(calc):	Delta	Sequence
136.2	2964.3004	-0.0047	SHCIAEVENDEMPADLPSLAADFVESK
46.9	2964.3004	-0.0047	SHCIAEVENDEMPADLPSLAADFVESK

{MATRIX SCIENCE} Mascot Search Results

Peptide View

MS/MS Fragmentation of **QNCELFEQLGEYKFQNALLVR**
Found in **sp|P02768|ALBU_HUMAN**, Serum albumin OS=Homo sapiens GN=ALB PE=1 SV=2

Match to Query 12865: 2589.251568 from(1295.633060,2+) intensity(2075408.6250) scans(15909) rtinseconds(2838) index(13600)
Title: 150818_TTSH_Patient_Plasma_94_Spectrum031069_scans__15909_RTINSECONDS=2838
Data file L:\\Ard_TTSH\\T1D\\T150818_TTSH_Patient_Plasma_94.mgf

Click mouse within plot area to zoom in by factor of two about that point
Or, Plot from 0 to 2600 Da Full range
Label all possible matches ○ Label matches used for scoring ⊙

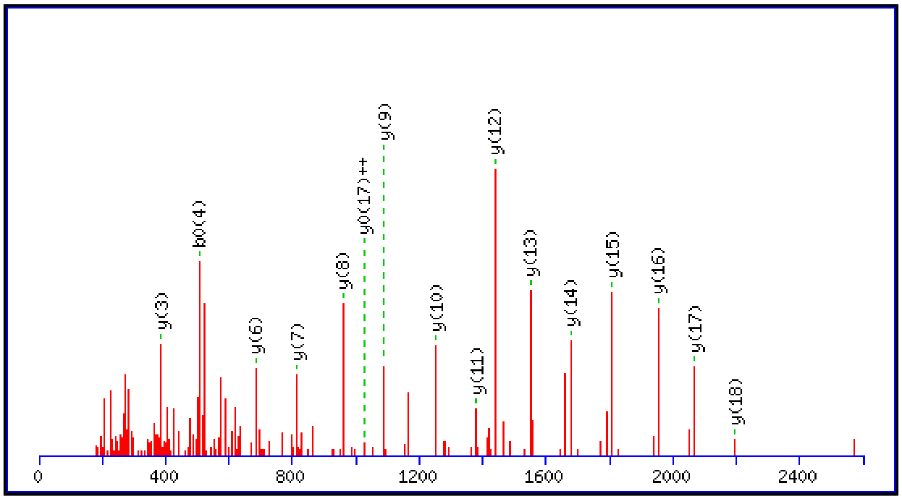

Monoisotopic mass of neutral peptide Mr(calc): 2589.2533
Variable modifications:
C3 : 4Trioxidation (CMWY)
Ions Score: 121 **Expect:** 4.1e-010
Matches : 16/214 fragment ions using 21 most intense peaks (help)

#	b	b^{++}	b*	b^{*++}	b^0	b^{0++}	Seq.	y	y^{++}	y*	y^{*++}	y^0	y^{0++}	#
1	129.0659	65.0366	112.0393	56.5233			Q							21
2	243.1088	122.0580	226.0822	113.5448			N	2462.2020	1231.6047	2445.1755	1223.0914	2444.1915	1222.5994	20
3	394.1027	197.5550	377.0762	189.0417			C	2348.1591	1174.5832	2331.1326	1166.0699	2330.1485	1165.5779	19
4	523.1453	262.0763	506.1188	253.5630	505.1347	253.0710	E	***2197.1652***	1099.0862	2180.1386	1090.5730	2179.1546	1090.0809	18
5	636.2294	318.6183	619.2028	310.1050	618.2188	309.6130	L	***2068.1226***	1034.5649	2051.0960	1026.0517	2050.1120	1025.5596	17
6	783.2978	392.1525	766.2712	383.6393	765.2872	383.1472	F	***1955.0385***	978.0229	1938.0120	969.5096	1937.0280	969.0176	16
7	912.3404	456.6738	895.3138	448.1606	894.3298	447.6685	E	***1807.9701***	904.4887	1790.9436	895.9754	1789.9595	895.4834	15
8	1040.3990	520.7031	1023.3724	512.1898	1022.3884	511.6978	Q	***1678.9275***	839.9674	1661.9010	831.4541	1660.9170	830.9621	14
9	1153.4830	577.2451	1136.4565	568.7319	1135.4725	568.2399	L	***1550.8689***	775.9381	1533.8424	767.4248	1532.8584	766.9328	13
10	1210.5045	605.7559	1193.4779	597.2426	1192.4939	596.7506	G	***1437.7849***	719.3961	1420.7583	710.8828	1419.7743	710.3908	12
11	1339.5471	670.2772	1322.5205	661.7639	1321.5365	661.2719	E	***1380.7634***	690.8853	1363.7369	682.3721	1362.7528	681.8801	11
12	1502.6104	751.8088	1485.5839	743.2956	1484.5998	742.8036	Y	***1251.7208***	626.3640	1234.6943	617.8508			10
13	1630.7054	815.8563	1613.6788	807.3430	1612.6948	806.8510	K	***1088.6575***	544.8324	1071.6309	536.3191			9
14	1777.7738	889.3905	1760.7472	880.8773	1759.7632	880.3852	F	***960.5625***	480.7849	943.5360	472.2716			8
15	1905.8324	953.4198	1888.8058	944.9065	1887.8218	944.4145	Q	***813.4941***	407.2507	796.4676	398.7374			7
16	2019.8753	1010.4413	2002.8487	1001.9280	2001.8647	1001.4360	N	***685.4355***	343.2214	668.4090	334.7081			6
17	2090.9124	1045.9598	2073.8858	1037.4466	2072.9018	1036.9546	A	571.3926	286.1999	554.3661	277.6867			5
18	2203.9965	1102.5019	2186.9699	1093.9886	2185.9859	1093.4966	L	500.3555	250.6814	483.3289	242.1681			4
19	2317.0805	1159.0439	2300.0540	1150.5306	2299.0700	1150.0386	L	***387.2714***	194.1394	370.2449	185.6261			3

**20**	2416.1489	1208.5781	2399.1224	1200.0648	2398.1384	1199.5728	**V**	274.1874	137.5973	257.1608	129.0840			**2**
**21**							**R**	175.1190	88.0631	158.0924	79.5498			**1**

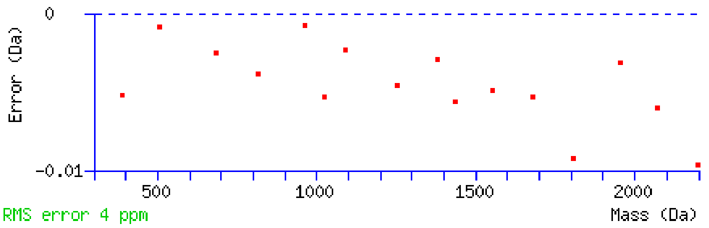

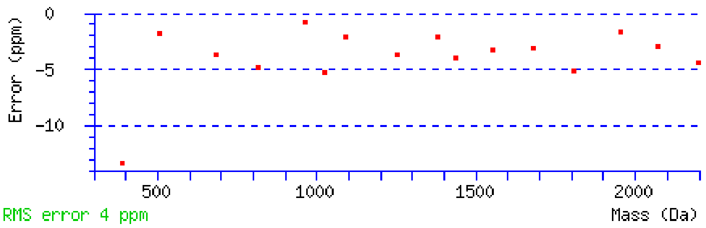

NCBI **BLAST** search of QNCELFEQLGEYKFQNALLVR
(Parameters: blastp, nr protein database, expect=20000, no filter, PAM30)
Other BLAST web gateways

All matches to this query

Score	Mr(calc):	Delta	Sequence
120.7	2589.2533	-0.0017	QNCELFEQLGEYKFQNALLVR
4.4	2589.2533	-0.0017	QNCELFEQLGEYKFQNALLVR

Mascot: http://www.matrixscience.com/

Mascot Search Results

Peptide View

MS/MS Fragmentation of **NECFLQHKDDNPNLPR**
Found in **sp|P02768|ALBU_HUMAN**, Serum albumin OS=Homo sapiens GN=ALB PE=1 SV=2

Match to Query 9782: 1986.883272 from(663.301700,3+) intensity(6449636.5000) scans(7329) rtinseconds(1302) index(6396)
Title: 150801_TTSH_Patient_Plasma_34_Spectrum024428_scans__7329_RTINSECONDS=1302
Data file L:\\Ard_TTSH\\T1D\\T150801_TTSH_Patient_Plasma_34.mgf

Click mouse within plot area to zoom in by factor of two about that point
Or, Plot from 100 to 1900 Da Full range
Label all possible matches ○ Label matches used for scoring ⊙

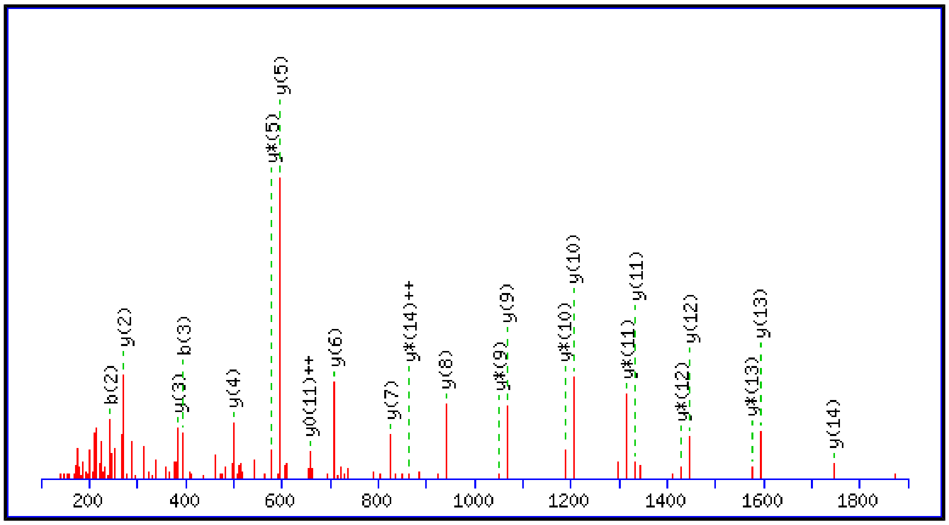

Monoisotopic mass of neutral peptide Mr(calc): 1986.8854
Variable modifications:
C3 : 4Trioxidation (CMWY)
Ions Score: 117 **Expect:** 6.8e-010
Matches : 23/166 fragment ions using 30 most intense peaks (help)

#	b	b^{++}	b*	b^{*++}	b^0	b^{0++}	Seq.	y	y^{++}	y*	y^{*++}	y^0	y^{0++}	#
1	115.0502	58.0287	98.0237	49.5155			N							16
2	**244.0928**	122.5500	227.0662	114.0368	226.0822	113.5448	E	1873.8497	937.4285	1856.8232	928.9152	1855.8392	928.4232	15
3	**395.0867**	198.0470	378.0602	189.5337	377.0762	189.0417	C	***1744.8071***	872.9072	1727.7806	864.3939	1726.7966	863.9019	14
4	542.1551	271.5812	525.1286	263.0679	524.1446	262.5759	F	***1593.8132***	797.4102	1576.7867	788.8970	1575.8026	788.4050	13
5	655.2392	328.1232	638.2127	319.6100	637.2286	319.1180	L	***1446.7448***	723.8760	1429.7183	715.3628	1428.7342	714.8708	12
6	783.2978	392.1525	766.2712	383.6393	765.2872	383.1472	Q	***1333.6607***	667.3340	1316.6342	658.8207	1315.6502	658.3287	11
7	920.3567	460.6820	903.3301	452.1687	902.3461	451.6767	H	***1205.6022***	603.3047	1188.5756	594.7914	1187.5916	594.2994	10
8	1048.4517	524.7295	1031.4251	516.2162	1030.4411	515.7242	K	***1068.5432***	534.7753	1051.5167	526.2620	1050.5327	525.7700	9
9	1163.4786	582.2429	1146.4521	573.7297	1145.4680	573.2377	D	***940.4483***	470.7278	923.4217	462.2145	922.4377	461.7225	8
10	1278.5055	639.7564	1261.4790	631.2431	1260.4950	630.7511	D	***825.4213***	413.2143	808.3948	404.7010	807.4108	404.2090	7
11	1392.5485	696.7779	1375.5219	688.2646	1374.5379	687.7726	N	***710.3944***	355.7008	693.3678	347.1876			6
12	1489.6012	745.3043	1472.5747	736.7910	1471.5907	736.2990	P	***596.3515***	298.6794	579.3249	290.1661			5
13	1603.6442	802.3257	1586.6176	793.8124	1585.6336	793.3204	N	***499.2987***	250.1530	482.2722	241.6397			4
14	1716.7282	858.8678	1699.7017	850.3545	1698.7177	849.8625	L	***385.2558***	193.1315	368.2292	184.6183			3
15	1813.7810	907.3941	1796.7544	898.8809	1795.7704	898.3888	P	***272.1717***	136.5895	255.1452	128.0762			2
16							R	175.1190	88.0631	158.0924	79.5498			1

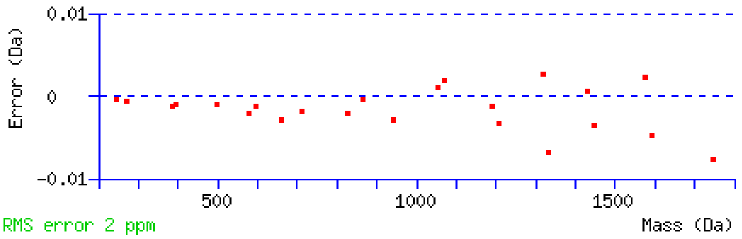

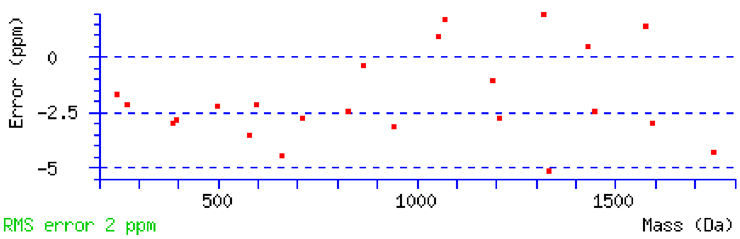

NCBI **BLAST** search of NECFLQHKDDNPNLPR
(Parameters: blastp, nr protein database, expect=20000, no filter, PAM30)
Other BLAST web gateways

All matches to this query

Score	Mr(calc):	Delta	Sequence
117.0	1986.8854	-0.0021	NECFLQHKDDNPNLPR
0.5	1986.8937	-0.0104	MCFCLQVWVVRMDPR
0.5	1986.8937	-0.0104	MCFCLQVWVVRMDPR
0.5	1986.8937	-0.0104	MCFCLQVWVVRMDPR
0.5	1986.8937	-0.0104	MCFCLQVWVVRMDPR

Mascot: http://www.matrixscience.com/

MATRIX SCIENCE Mascot Search Results

Peptide View

MS/MS Fragmentation of **SLHTLFGDKLCTVATLR**
Found in **sp|P02768|ALBU_HUMAN**, Serum albumin OS=Homo sapiens GN=ALB PE=1 SV=2

Match to Query 9433: 1921.994108 from(962.004330,2+) intensity(6899729.0000) scans(14193) rtinseconds(2472) index(12311)
Title: 150818_TTSH_Patient_Plasma_43_Spectrum030760_scans__14193_RTINSECONDS=2472
Data file L:\\Ard_TTSH\\T1D\\T150818_TTSH_Patient_Plasma_43.mgf

Click mouse within plot area to zoom in by factor of two about that point
Or, Plot from 100 to 2000 Da Full range
Label all possible matches ○ Label matches used for scoring ⊙

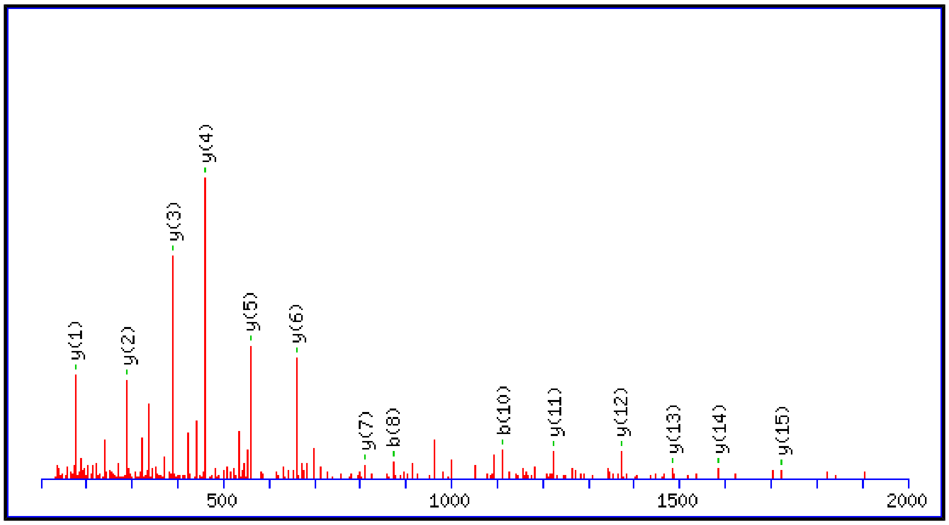

Monoisotopic mass of neutral peptide Mr(calc): 1921.9931
Variable modifications:
C11 : 4Trioxidation (CMWY)
Ions Score: 115 **Expect:** 8.6e-010
Matches : 14/172 fragment ions using 18 most intense peaks (help)

#	b	b^{++}	b*	b^{*++}	b^{0}	b^{0++}	Seq.	y	y^{++}	y*	y^{*++}	y^{0}	y^{0++}	#
1	88.0393	44.5233			70.0287	35.5180	S							17
2	201.1234	101.0653			183.1128	92.0600	L	1835.9684	918.4878	1818.9418	909.9746	1817.9578	909.4826	16
3	338.1823	169.5948			320.1717	160.5895	H	1722.8843	861.9458	1705.8578	853.4325	1704.8738	852.9405	15
4	439.2300	220.1186			421.2194	211.1133	T	1585.8254	793.4163	1568.7989	784.9031	1567.8149	784.4111	14
5	552.3140	276.6606			534.3035	267.6554	L	1484.7777	742.8925	1467.7512	734.3792	1466.7672	733.8872	13
6	699.3824	350.1949			681.3719	341.1896	F	1371.6937	686.3505	1354.6671	677.8372	1353.6831	677.3452	12
7	756.4039	378.7056			738.3933	369.7003	G	1224.6253	612.8163	1207.5987	604.3030	1206.6147	603.8110	11
8	871.4308	436.2191			853.4203	427.2138	D	1167.6038	584.3055	1150.5773	575.7923	1149.5932	575.3003	10
9	999.5258	500.2665	982.4993	491.7533	981.5152	491.2613	K	1052.5769	526.7921	1035.5503	518.2788	1034.5663	517.7868	9
10	1112.6099	556.8086	1095.5833	548.2953	1094.5993	547.8033	L	924.4819	462.7446	907.4553	454.2313	906.4713	453.7393	8
11	1263.6038	632.3055	1246.5773	623.7923	1245.5932	623.3003	C	811.3978	406.2026	794.3713	397.6893	793.3873	397.1973	7
12	1364.6515	682.8294	1347.6249	674.3161	1346.6409	673.8241	T	660.4039	330.7056	643.3774	322.1923	642.3933	321.7003	6
13	1463.7199	732.3636	1446.6933	723.8503	1445.7093	723.3583	V	559.3562	280.1817	542.3297	271.6685	541.3457	271.1765	5
14	1534.7570	767.8821	1517.7305	759.3689	1516.7464	758.8769	A	460.2878	230.6475	443.2613	222.1343	442.2772	221.6423	4
15	1635.8047	818.4060	1618.7781	809.8927	1617.7941	809.4007	T	389.2507	195.1290	372.2241	186.6157	371.2401	186.1237	3
16	1748.8887	874.9480	1731.8622	866.4347	1730.8782	865.9427	L	288.2030	144.6051	271.1765	136.0919			2
17							R	175.1190	88.0631	158.0924	79.5498			1

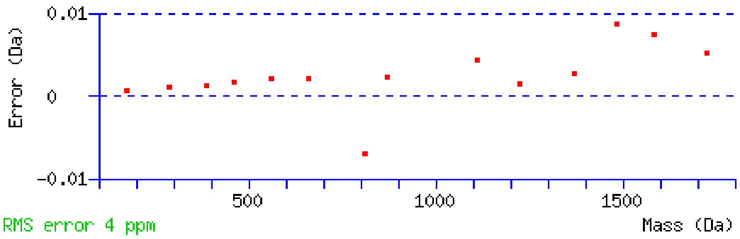

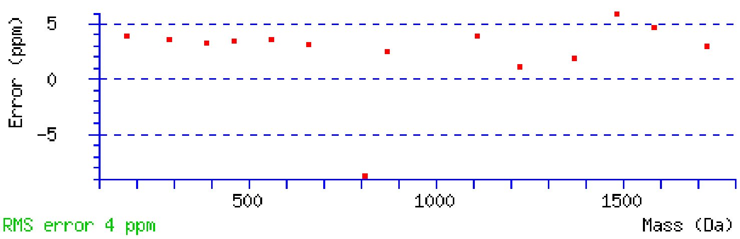

NCBI **BLAST** search of SLHTLFGDKLCTVATLR
(Parameters: blastp, nr protein database, expect=20000, no filter, PAM30)
Other BLAST web gateways

All matches to this query

Score	Mr(calc):	Delta	Sequence
114.8	1921.9931	0.0010	SLHTLFGDKLCTVATLR
1.4	1921.9891	0.0050	NMTGLVDLTLSRNAITR
0.9	1922.0109	-0.0168	GVYTVEAKNASGSAKAEIK
0.4	1919.9927	2.0014	CDTVYRKYLYVLATR

Mascot: http://www.matrixscience.com/

Mascot Search Results

Peptide View

MS/MS Fragmentation of **SHCIAEVENDEMPADLPSLAADFVESK**
Found in **sp|P02768|ALBU_HUMAN**, Serum albumin OS=Homo sapiens GN=ALB PE=1 SV=2

Match to Query 12982: 2964.300072 from(989.107300,3+) intensity(2071083.2500) scans(16098) rtinseconds(3148) index(12879)
Title: 150801_TTSH_Patient_Plasma_76_Spectrum028040_scans__16098_RTINSECONDS=3148
Data file L:\\Ard_TTSH\\T1D\\T150801_TTSH_Patient_Plasma_76.mgf

Click mouse within plot area to zoom in by factor of two about that point
Or, Plot from 200 to 3000 Da Full range
Label all possible matches ○ Label matches used for scoring ⊙

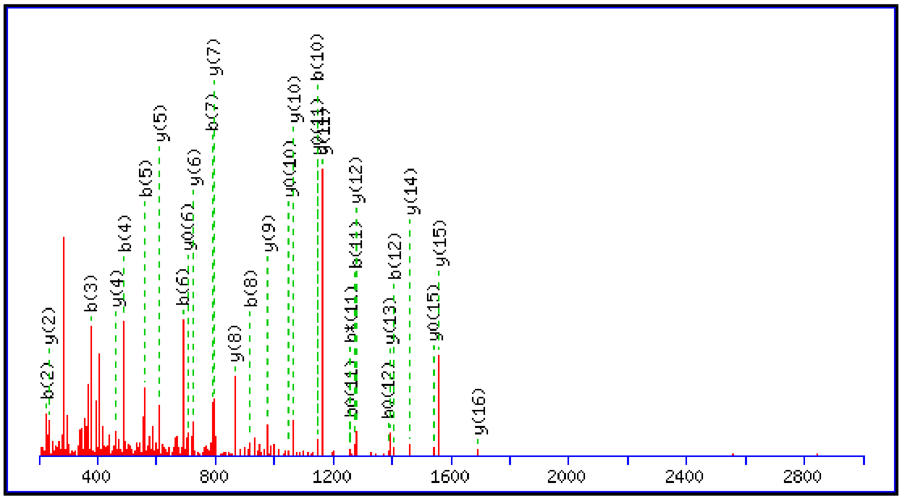

Monoisotopic mass of neutral peptide Mr(calc): 2964.3004
Variable modifications:
C3 : 4Trioxidation (CMWY)
Ions Score: 106 **Expect:** 1.3e-008
Matches : 31/294 fragment ions using 55 most intense peaks (help)

#	b	b^{++}	b*	b*$^{++}$	b^{0}	b^{0++}	Seq.	y	y^{++}	y*	y*$^{++}$	y^{0}	y^{0++}	#
1	88.0393	44.5233			70.0287	35.5180	S							27
2	***225.0982***	113.0527			207.0877	104.0475	H	2878.2757	1439.6415	2861.2492	1431.1282	2860.2652	1430.6362	26
3	***376.0921***	188.5497			358.0816	179.5444	C	2741.2168	1371.1120	2724.1903	1362.5988	2723.2063	1362.1068	25
4	***489.1762***	245.0917			471.1656	236.0865	I	2590.2229	1295.6151	2573.1963	1287.1018	2572.2123	1286.6098	24
5	***560.2133***	280.6103			542.2028	271.6050	A	2477.1388	1239.0731	2460.1123	1230.5598	2459.1283	1230.0678	23
6	***689.2559***	345.1316			671.2454	336.1263	E	2406.1017	1203.5545	2389.0752	1195.0412	2388.0911	1194.5492	22
7	***788.3243***	394.6658			770.3138	385.6605	V	2277.0591	1139.0332	2260.0326	1130.5199	2259.0486	1130.0279	21
8	***917.3669***	459.1871			899.3564	450.1818	E	2177.9907	1089.4990	2160.9642	1080.9857	2159.9801	1080.4937	20
9	1031.4099	516.2086	1014.3833	507.6953	1013.3993	507.2033	N	2048.9481	1024.9777	2031.9216	1016.4644	2030.9375	1015.9724	19
10	***1146.4368***	573.7220	1129.4102	565.2088	1128.4262	564.7168	D	1934.9052	967.9562	1917.8786	959.4430	1916.8946	958.9509	18
11	***1275.4794***	638.2433	1258.4528	629.7301	1257.4688	629.2380	E	1819.8782	910.4428	1802.8517	901.9295	1801.8677	901.4375	17
12	***1406.5199***	703.7636	1389.4933	695.2503	1388.5093	694.7583	M	***1690.8357***	845.9215	1673.8091	837.4082	1672.8251	836.9162	16
13	1503.5726	752.2900	1486.5461	743.7767	1485.5621	743.2847	P	***1559.7952***	780.4012	1542.7686	771.8879	1541.7846	771.3959	15
14	1574.6097	787.8085	1557.5832	779.2952	1556.5992	778.8032	A	***1462.7424***	731.8748	1445.7159	723.3616	1444.7318	722.8696	14
15	1689.6367	845.3220	1672.6101	836.8087	1671.6261	836.3167	D	***1391.7053***	696.3563	1374.6787	687.8430	1373.6947	687.3510	13
16	1802.7208	901.8640	1785.6942	893.3507	1784.7102	892.8587	L	***1276.6783***	638.8428	1259.6518	630.3295	1258.6678	629.8375	12
17	1899.7735	950.3904	1882.7470	941.8771	1881.7630	941.3851	P	***1163.5943***	582.3008	1146.5677	573.7875	1145.5837	573.2955	11
18	1986.8055	993.9064	1969.7790	985.3931	1968.7950	984.9011	S	***1066.5415***	533.7744	1049.5150	525.2611	1048.5310	524.7691	10
19	2099.8896	1050.4484	2082.8631	1041.9352	2081.8790	1041.4432	L	***979.5095***	490.2584	962.4829	481.7451	961.4989	481.2531	9

**20**	2170.9267	1085.9670	2153.9002	1077.4537	2152.9162	1076.9617	**A**	***866.4254***	433.7164	849.3989	425.2031	848.4149	424.7111	**8**
**21**	2241.9638	1121.4856	2224.9373	1112.9723	2223.9533	1112.4803	**A**	***795.3883***	398.1978	778.3618	389.6845	777.3777	389.1925	**7**
**22**	2356.9908	1178.9990	2339.9642	1170.4858	2338.9802	1169.9937	**D**	***724.3512***	362.6792	707.3246	354.1660	706.3406	353.6740	**6**
**23**	2504.0592	1252.5332	2487.0326	1244.0200	2486.0486	1243.5280	**F**	***609.3243***	305.1658	592.2977	296.6525	591.3137	296.1605	**5**
**24**	2603.1276	1302.0674	2586.1011	1293.5542	2585.1170	1293.0622	**V**	***462.2558***	231.6316	445.2293	223.1183	444.2453	222.6263	**4**
**25**	2732.1702	1366.5887	2715.1437	1358.0755	2714.1596	1357.5835	**E**	363.1874	182.0974	346.1609	173.5841	345.1769	173.0921	**3**
**26**	2819.2022	1410.1048	2802.1757	1401.5915	2801.1917	1401.0995	**S**	***234.1448***	117.5761	217.1183	109.0628	216.1343	108.5708	**2**
**27**							**K**	147.1128	74.0600	130.0863	65.5468			**1**

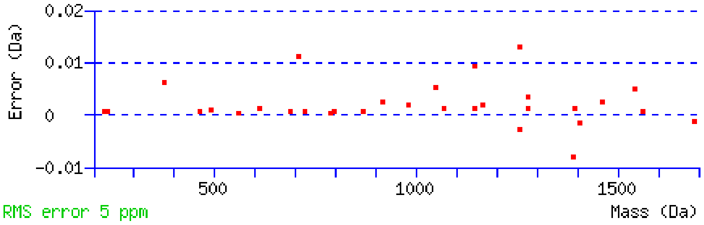

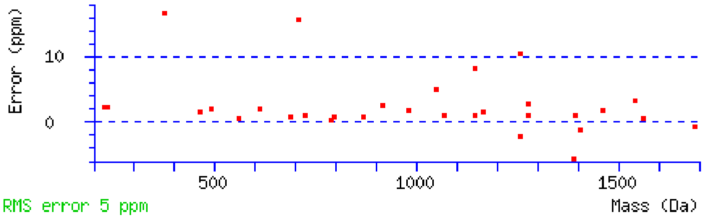

NCBI **BLAST** search of SHCIAEVENDEMPADLPSLAADFVESK
(Parameters: blastp, nr protein database, expect=20000, no filter, PAM30)
Other BLAST web gateways

All matches to this query

Score	Mr(calc):	Delta	Sequence
105.9	2964.3004	-0.0004	SHCIAEVENDEMPADLPSLAADFVESK
51.0	2964.3004	-0.0004	SHCIAEVENDEMPADLPSLAADFVESK

Mascot: http://www.matrixscience.com/

Mascot Search Results

Peptide View

MS/MS Fragmentation of **QEPERNECFLQHKDDNPNLPR**
Found in **sp|P02768|ALBU_HUMAN**, Serum albumin OS=Homo sapiens GN=ALB PE=1 SV=2

Match to Query 13218: 2626.179882 from(876.400570,3+) intensity(1040825.1875) scans(8275) rtinseconds(1538) index(6984)
Title: 150808_TTSH_Patient_Plasma_74_Spectrum023404_scans__8275_RTINSECONDS=1538
Data file L:\\Ard_TTSH\\T1D\\T150808_TTSH_Patient_Plasma_74.mgf

Click mouse within plot area to zoom in by factor of two about that point
Or, Plot from 0 to 2800 Da Full range
Label all possible matches ○ Label matches used for scoring ⦿

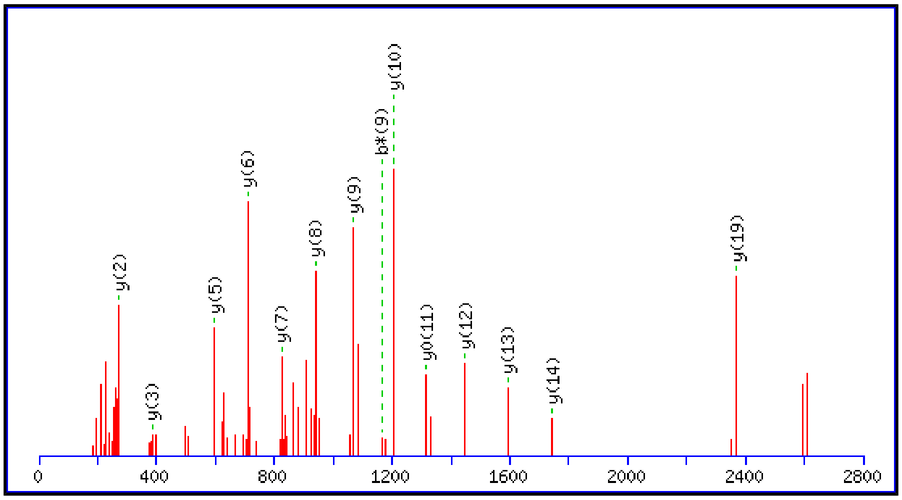

Monoisotopic mass of neutral peptide Mr(calc): 2626.1830
Variable modifications:
C8 : 4Trioxidation (CMWY)
Ions Score: 105 **Expect:** 2.1e-008
Matches : 14/226 fragment ions using 17 most intense peaks (help)

#	b	b^{++}	b*	b^{*++}	b^0	b^{0++}	Seq.	y	y^{++}	y*	y^{*++}	y^0	y^{0++}	#
1	129.0659	65.0366	112.0393	56.5233			Q							21
2	258.1084	129.5579	241.0819	121.0446	240.0979	120.5526	E	2499.1317	1250.0695	2482.1052	1241.5562	2481.1212	1241.0642	20
3	355.1612	178.0842	338.1347	169.5710	337.1506	169.0790	P	***2370.0891***	1185.5482	2353.0626	1177.0349	2352.0786	1176.5429	19
4	484.2038	242.6055	467.1773	234.0923	466.1932	233.6003	E	2273.0364	1137.0218	2256.0098	1128.5085	2255.0258	1128.0165	18
5	640.3049	320.6561	623.2784	312.1428	622.2944	311.6508	R	2143.9938	1072.5005	2126.9672	1063.9873	2125.9832	1063.4952	17
6	754.3478	377.6776	737.3213	369.1643	736.3373	368.6723	N	1987.8927	994.4500	1970.8661	985.9367	1969.8821	985.4447	16
7	883.3904	442.1989	866.3639	433.6856	865.3799	433.1936	E	1873.8497	937.4285	1856.8232	928.9152	1855.8392	928.4232	15
8	1034.3844	517.6958	1017.3578	509.1825	1016.3738	508.6905	C	***1744.8071***	872.9072	1727.7806	864.3939	1726.7966	863.9019	14
9	1181.4528	591.2300	1164.4262	582.7168	1163.4422	582.2247	F	***1593.8132***	797.4102	1576.7867	788.8970	1575.8027	788.4050	13
10	1294.5368	647.7721	1277.5103	639.2588	1276.5263	638.7668	L	***1446.7448***	723.8760	1429.7183	715.3628	1428.7342	714.8708	12
11	1422.5954	711.8013	1405.5689	703.2881	1404.5849	702.7961	Q	1333.6607	667.3340	1316.6342	658.8207	1315.6502	658.3287	11
12	1559.6543	780.3308	1542.6278	771.8175	1541.6438	771.3255	H	***1205.6022***	603.3047	1188.5756	594.7914	1187.5916	594.2994	10
13	1687.7493	844.3783	1670.7227	835.8650	1669.7387	835.3730	K	***1068.5432***	534.7753	1051.5167	526.2620	1050.5327	525.7700	9
14	1802.7762	901.8918	1785.7497	893.3785	1784.7657	892.8865	D	***940.4483***	470.7278	923.4217	462.2145	922.4377	461.7225	8
15	1917.8032	959.4052	1900.7766	950.8920	1899.7926	950.3999	D	***825.4213***	413.2143	808.3948	404.7010	807.4108	404.2090	7
16	2031.8461	1016.4267	2014.8196	1007.9134	2013.8355	1007.4214	N	***710.3944***	355.7008	693.3678	347.1876			6
17	2128.8989	1064.9531	2111.8723	1056.4398	2110.8883	1055.9478	P	***596.3515***	298.6794	579.3249	290.1661			5
18	2242.9418	1121.9745	2225.9153	1113.4613	2224.9312	1112.9693	N	499.2987	250.1530	482.2722	241.6397			4
19	2356.0259	1178.5166	2338.9993	1170.0033	2338.0153	1169.5113	L	***385.2558***	193.1315	368.2292	184.6183			3

**20**	2453.0786	1227.0430	2436.0521	1218.5297	2435.0681	1218.0377	**P**	*272.1717*	136.5895	255.1452	128.0762			**2**
**21**							**R**	175.1190	88.0631	158.0924	79.5498			**1**

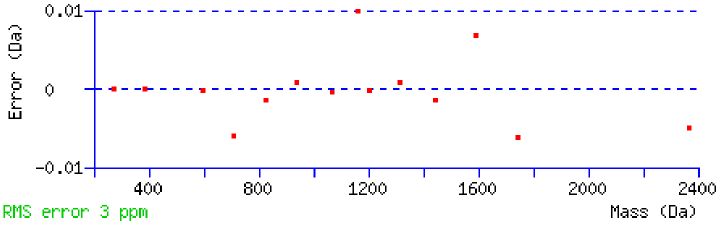

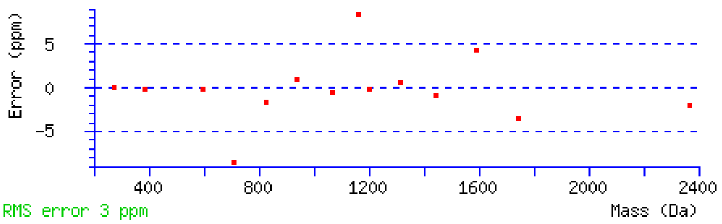

NCBI **BLAST** search of QEPERNECFLQHKDDNPNLPR
(Parameters: blastp, nr protein database, expect=20000, no filter, PAM30)
Other BLAST web gateways

All matches to this query

Score	Mr(calc):	Delta	Sequence
105.0	2626.1830	-0.0031	QEPERNECFLQHKDDNPNLPR

Mascot: http://www.matrixscience.com/

Mascot Search Results

Peptide View

MS/MS Fragmentation of **ADDKETCFAEEGKK**
Found in **sp|P02768|ALBU_HUMAN**, Serum albumin OS=Homo sapiens GN=ALB PE=1 SV=2

Match to Query 7413: 1617.681102 from(540.234310,3+) intensity(61276500.0000) scans(1374) rtinseconds(281) index(1072)
Title: 150801_TTSH_Patient_Plasma_40_Spectrum018007_scans__1374_RTINSECONDS=281
Data file L:\\Ard_TTSH\\T1D\\T150801_TTSH_Patient_Plasma_40.mgf

Click mouse within plot area to zoom in by factor of two about that point
Or, Plot from 100 to 1600 Da Full range
Label all possible matches ○ Label matches used for scoring ⦿

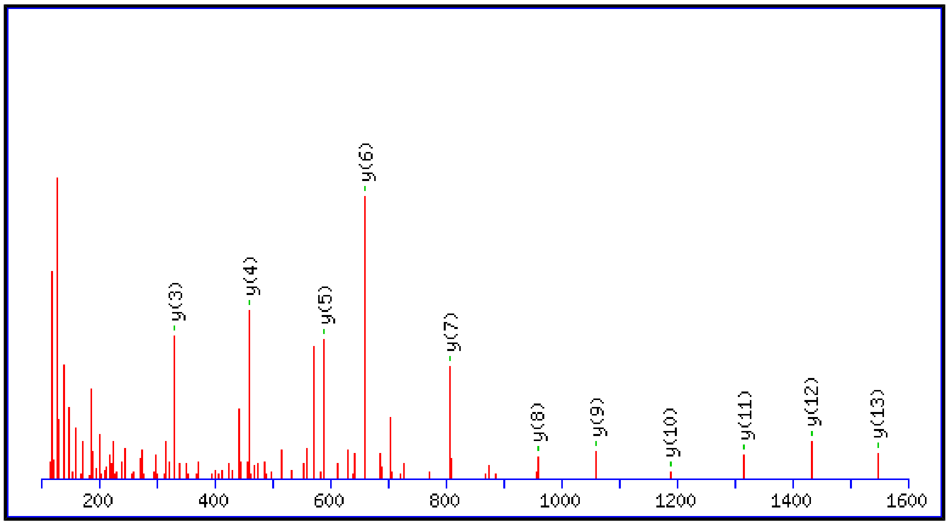


```
Monoisotopic mass of neutral peptide Mr(calc): 1617.6828
Variable modifications:
C7     : 4Trioxidation (CMWY)
Ions Score: 105  Expect: 5.8e-009
Matches : 11/142 fragment ions using 14 most intense peaks   (help)
```

#	b	b^{++}	b*	b^{*++}	b^{0}	b^{0++}	Seq.	y	y^{++}	y*	y^{*++}	y^{0}	y^{0++}	#
1	72.0444	36.5258					A							14
2	187.0713	94.0393			169.0608	85.0340	D	***1547.6530***	774.3301	1530.6264	765.8169	1529.6424	765.3249	13
3	302.0983	151.5528			284.0877	142.5475	D	***1432.6261***	716.8167	1415.5995	708.3034	1414.6155	707.8114	12
4	430.1932	215.6003	413.1667	207.0870	412.1827	206.5950	K	***1317.5991***	659.3032	1300.5726	650.7899	1299.5885	650.2979	11
5	559.2358	280.1216	542.2093	271.6083	541.2253	271.1163	E	***1189.5041***	595.2557	1172.4776	586.7424	1171.4936	586.2504	10
6	660.2835	330.6454	643.2570	322.1321	642.2729	321.6401	T	***1060.4616***	530.7344	1043.4350	522.2211	1042.4510	521.7291	9
7	811.2774	406.1424	794.2509	397.6291	793.2669	397.1371	C	***959.4139***	480.2106	942.3873	471.6973	941.4033	471.2053	8
8	958.3459	479.6766	941.3193	471.1633	940.3353	470.6713	F	***808.4199***	404.7136	791.3934	396.2003	790.4094	395.7083	7
9	1029.3830	515.1951	1012.3564	506.6818	1011.3724	506.1898	A	***661.3515***	331.1794	644.3250	322.6661	643.3410	322.1741	6
10	1158.4256	579.7164	1141.3990	571.2031	1140.4150	570.7111	E	***590.3144***	295.6608	573.2879	287.1476	572.3039	286.6556	5
11	1287.4682	644.2377	1270.4416	635.7244	1269.4576	635.2324	E	***461.2718***	231.1396	444.2453	222.6263	443.2613	222.1343	4
12	1344.4896	672.7484	1327.4631	664.2352	1326.4791	663.7432	G	***332.2292***	166.6183	315.2027	158.1050			3
13	1472.5846	736.7959	1455.5580	728.2827	1454.5740	727.7906	K	275.2078	138.1075	258.1812	129.5942			2
14							K	147.1128	74.0600	130.0863	65.5468			1

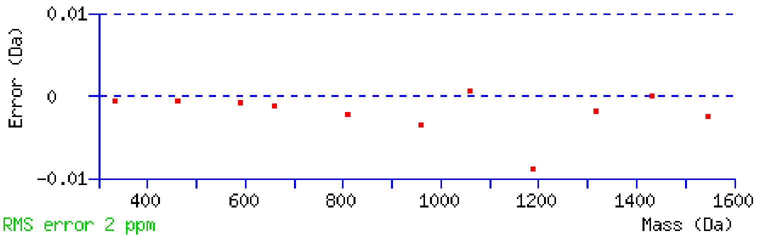

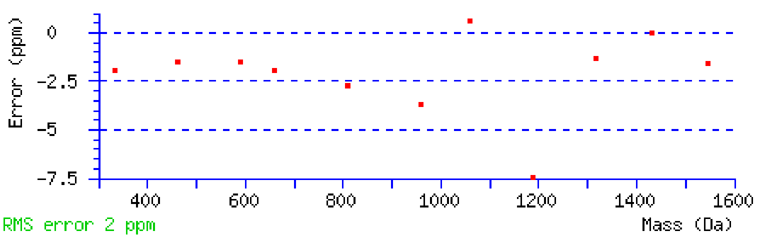

NCBI **BLAST** search of ADDKETCFAEEGKK
(Parameters: blastp, nr protein database, expect=20000, no filter, PAM30)
Other BLAST web gateways

All matches to this query

Score	Mr(calc):	Delta	Sequence
104.8	1617.6828	-0.0017	ADDKETCFAEEGKK

Mascot: http://www.matrixscience.com/

Mascot Search Results

Peptide View

MS/MS Fragmentation of **ALVLIAFAQYLQQCPFEDHVK**
Found in **sp|P02768|ALBU_HUMAN**, Serum albumin OS=Homo sapiens GN=ALB PE=1 SV=2

Match to Query 12476: 2480.239608 from(1241.127080,2+) intensity(10307705.0000) scans(18076) rtinseconds(3116) index(15668)
Title: 150818_TTSH_Patient_Plasma_37_Spectrum034070_scans__18076_RTINSECONDS=3116
Data file L:\\Ard_TTSH\\T1D\\T150818_TTSH_Patient_Plasma_37.mgf

Click mouse within plot area to zoom in by factor of two about that point
Or, [Plot from] [0] to [2200] Da [Full range]
Label all possible matches ○ Label matches used for scoring ⦿

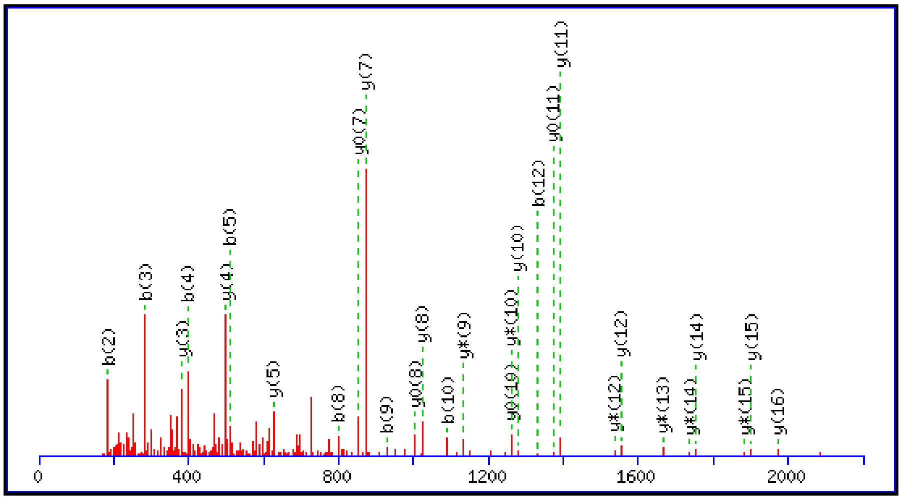

Monoisotopic mass of neutral peptide Mr(calc): 2480.2410
Variable modifications:
C14 : 4Trioxidation (CMWY)
Ions Score: 103 **Expect:** 1.9e-008
Matches : 29/186 fragment ions using 35 most intense peaks (help)

#	b	b^{++}	b*	b^{*++}	b^0	b^{0++}	Seq.	y	y^{++}	y*	y^{*++}	y^0	y^{0++}	#
1	72.0444	36.5258					A							21
2	185.1285	93.0679					L	2410.2111	1205.6092	2393.1846	1197.0959	2392.2006	1196.6039	20
3	284.1969	142.6021					V	2297.1271	1149.0672	2280.1005	1140.5539	2279.1165	1140.0619	19
4	397.2809	199.1441					L	2198.0587	1099.5330	2181.0321	1091.0197	2180.0481	1090.5277	18
5	510.3650	255.6861					I	2084.9746	1042.9909	2067.9481	1034.4777	2066.9640	1033.9857	17
6	581.4021	291.2047					A	1971.8905	986.4489	1954.8640	977.9356	1953.8800	977.4436	16
7	728.4705	364.7389					F	1900.8534	950.9304	1883.8269	942.4171	1882.8429	941.9251	15
8	799.5076	400.2575					A	1753.7850	877.3961	1736.7585	868.8829	1735.7744	868.3909	14
9	927.5662	464.2867	910.5397	455.7735			Q	1682.7479	841.8776	1665.7214	833.3643	1664.7373	832.8723	13
10	1090.6295	545.8184	1073.6030	537.3051			Y	1554.6893	777.8483	1537.6628	769.3350	1536.6788	768.8430	12
11	1203.7136	602.3604	1186.6871	593.8472			L	1391.6260	696.3166	1374.5994	687.8034	1373.6154	687.3114	11
12	1331.7722	666.3897	1314.7456	657.8765			Q	1278.5419	639.7746	1261.5154	631.2613	1260.5314	630.7693	10
13	1459.8308	730.4190	1442.8042	721.9057			Q	1150.4834	575.7453	1133.4568	567.2320	1132.4728	566.7400	9
14	1610.8247	805.9160	1593.7981	797.4027			C	1022.4248	511.7160	1005.3982	503.2027	1004.4142	502.7107	8
15	1707.8775	854.4424	1690.8509	845.9291			P	871.4308	436.2191	854.4043	427.7058	853.4203	427.2138	7
16	1854.9459	927.9766	1837.9193	919.4633			F	774.3781	387.6927	757.3515	379.1794	756.3675	378.6874	6
17	1983.9885	992.4979	1966.9619	983.9846	1965.9779	983.4926	E	627.3097	314.1585	610.2831	305.6452	609.2991	305.1532	5
18	2099.0154	1050.0113	2081.9889	1041.4981	2081.0048	1041.0061	D	498.2671	249.6372	481.2405	241.1239	480.2565	240.6319	4
19	2236.0743	1118.5408	2219.0478	1110.0275	2218.0638	1109.5355	H	383.2401	192.1237	366.2136	183.6104			3

**20**	2335.1427	1168.0750	2318.1162	1159.5617	2317.1322	1159.0697	**V**	246.1812	123.5942	229.1547	115.0810			**2**
**21**							**K**	147.1128	74.0600	130.0863	65.5468			**1**

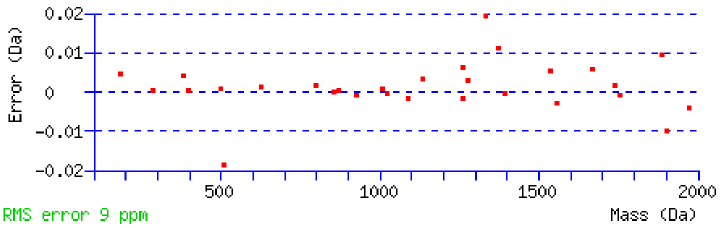

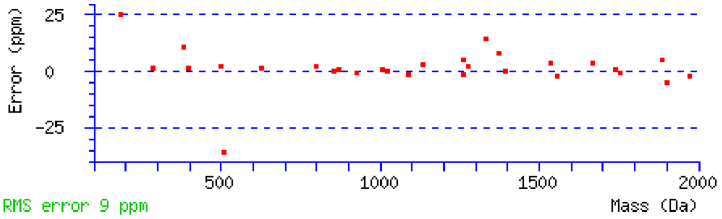

NCBI **BLAST** search of ALVLIAFAQYLQQCPFEDHVK
(Parameters: blastp, nr protein database, expect=20000, no filter, PAM30)
Other BLAST web gateways

All matches to this query

Score	Mr(calc):	Delta	Sequence
103.1	2480.2410	-0.0014	ALVLIAFAQYLQQCPFEDHVK
46.6	2480.2410	-0.0014	ALVLIAFAQYLQQCPFEDHVK

Mascot: http://www.matrixscience.com/

Mascot Search Results

Peptide View

MS/MS Fragmentation of **QNCELFEQLGEYKFQNALLVR**
Found in **sp|P02768|ALBU_HUMAN**, Serum albumin OS=Homo sapiens GN=ALB PE=1 SV=2

Match to Query 12412: 2589.238902 from(864.086910,3+) intensity(2067157.5000) scans(15772) rtinseconds(2821) index(13429)
Title: 150825_TTSH_Patient_Plasma_13_Spectrum030867_scans__15772_RTINSECONDS=2821
Data file L:\\Ard_TTSH\\T1D\\T150825_TTSH_Patient_Plasma_13.mgf

Click mouse within plot area to zoom in by factor of two about that point
Or, Plot from 100 to 2100 Da Full range
Label all possible matches ○ Label matches used for scoring ⊙

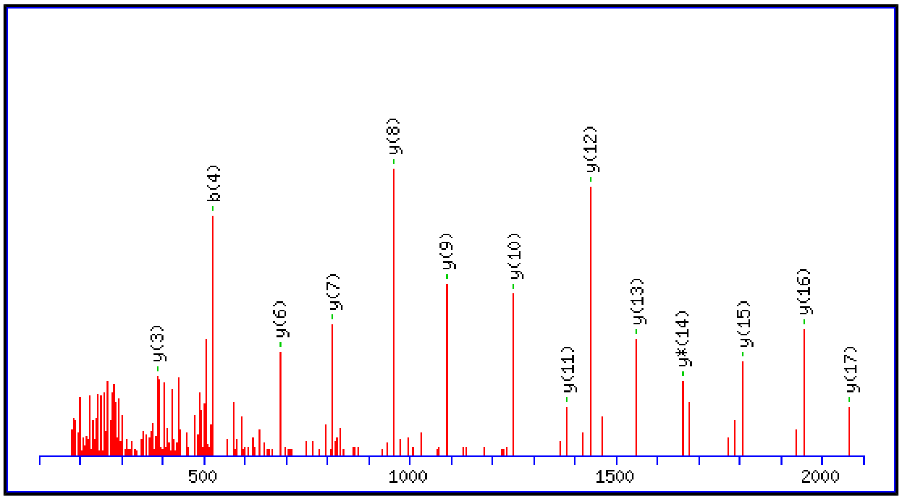

Monoisotopic mass of neutral peptide Mr(calc): 2589.2533
Variable modifications:
C3 : 4Trioxidation (CMWY)
Ions Score: 96 **Expect:** 1.3e-007
Matches : 14/214 fragment ions using 19 most intense peaks (help)

#	b	b^{++}	b*	b^{*++}	b^{0}	b^{0++}	Seq.	y	y^{++}	y*	y^{*++}	y^{0}	y^{0++}	#
1	129.0659	65.0366	112.0393	56.5233			Q							21
2	243.1088	122.0580	226.0822	113.5448			N	2462.2020	1231.6047	2445.1755	1223.0914	2444.1915	1222.5994	20
3	394.1027	197.5550	377.0762	189.0417			C	2348.1591	1174.5832	2331.1326	1166.0699	2330.1485	1165.5779	19
4	**523.1453**	262.0763	506.1188	253.5630	505.1347	253.0710	E	2197.1652	1099.0862	2180.1386	1090.5730	2179.1546	1090.0809	18
5	636.2294	318.6183	619.2028	310.1050	618.2188	309.6130	L	***2068.1226***	1034.5649	2051.0960	1026.0517	2050.1120	1025.5596	17
6	783.2978	392.1525	766.2712	383.6393	765.2872	383.1472	F	***1955.0385***	978.0229	1938.0120	969.5096	1937.0280	969.0176	16
7	912.3404	456.6738	895.3138	448.1606	894.3298	447.6685	E	***1807.9701***	904.4887	1790.9436	895.9754	1789.9595	895.4834	15
8	1040.3990	520.7031	1023.3724	512.1898	1022.3884	511.6978	Q	1678.9275	839.9674	1661.9010	831.4541	1660.9170	830.9621	14
9	1153.4830	577.2451	1136.4565	568.7319	1135.4725	568.2399	L	***1550.8689***	775.9381	1533.8424	767.4248	1532.8584	766.9328	13
10	1210.5045	605.7559	1193.4779	597.2426	1192.4939	596.7506	G	***1437.7849***	719.3961	1420.7583	710.8828	1419.7743	710.3908	12
11	1339.5471	670.2772	1322.5205	661.7639	1321.5365	661.2719	E	***1380.7634***	690.8853	1363.7369	682.3721	1362.7528	681.8801	11
12	1502.6104	751.8088	1485.5839	743.2956	1484.5998	742.8036	Y	***1251.7208***	626.3640	1234.6943	617.8508			10
13	1630.7054	815.8563	1613.6788	807.3430	1612.6948	806.8510	K	***1088.6575***	544.8324	1071.6309	536.3191			9
14	1777.7738	889.3905	1760.7472	880.8773	1759.7632	880.3852	F	***960.5625***	480.7849	943.5360	472.2716			8
15	1905.8324	953.4198	1888.8058	944.9065	1887.8218	944.4145	Q	***813.4941***	407.2507	796.4676	398.7374			7
16	2019.8753	1010.4413	2002.8487	1001.9280	2001.8647	1001.4360	N	***685.4355***	343.2214	668.4090	334.7081			6
17	2090.9124	1045.9598	2073.8858	1037.4466	2072.9018	1036.9546	A	571.3926	286.1999	554.3661	277.6867			5
18	2203.9965	1102.5019	2186.9699	1093.9886	2185.9859	1093.4966	L	500.3555	250.6814	483.3289	242.1681			4
19	2317.0805	1159.0439	2300.0540	1150.5306	2299.0700	1150.0386	L	***387.2714***	194.1394	370.2449	185.6261			3

**20**	2416.1489	1208.5781	2399.1224	1200.0648	2398.1384	1199.5728	**V**	274.1874	137.5973	257.1608	129.0840			**2**
**21**							**R**	175.1190	88.0631	158.0924	79.5498			**1**

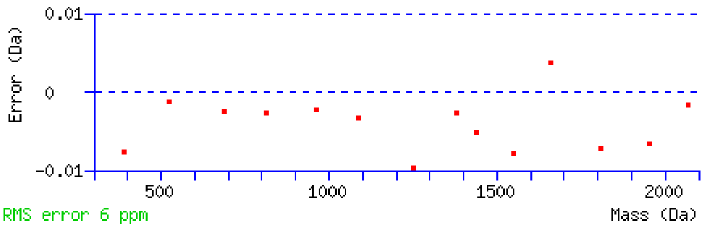

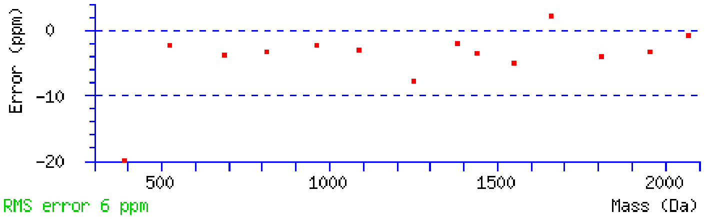

NCBI **BLAST** search of QNCELFEQLGEYKFQNALLVR
(Parameters: blastp, nr protein database, expect=20000, no filter, PAM30)
Other BLAST web gateways

All matches to this query

Score	Mr(calc):	Delta	Sequence
95.8	2589.2533	-0.0144	QNCELFEQLGEYKFQNALLVR
6.2	2589.2533	-0.0144	QNCELFEQLGEYKFQNALLVR

Mascot: http://www.matrixscience.com/

Mascot Search Results

Peptide View

MS/MS Fragmentation of **LVRPEVDVMCTAFHDNEETFLKK**
Found in **sp|P02768|ALBU_HUMAN**, Serum albumin OS=Homo sapiens GN=ALB PE=1 SV=2

Match to Query 12999: 2768.316856 from(693.086490,4+) intensity(1351313.7500) scans(13026) rtinseconds(2315) index(11133)
Title: 150801_TTSH_Patient_Plasma_45_Spectrum028559_scans__13026_RTINSECONDS=2315
Data file L:\\Ard_TTSH\\T1D\\T150801_TTSH_Patient_Plasma_45.mgf

Click mouse within plot area to zoom in by factor of two about that point
Or, Plot from 100 to 1900 Da Full range
Label all possible matches ○ Label matches used for scoring ⊙

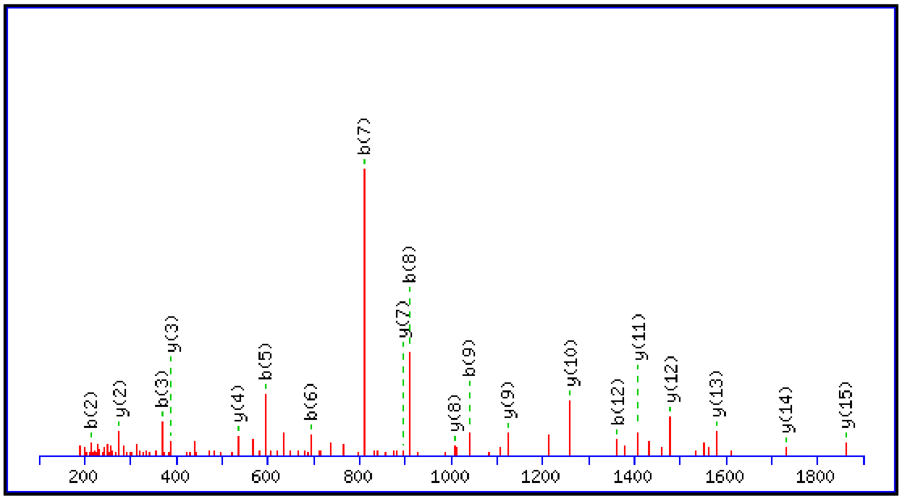

Monoisotopic mass of neutral peptide Mr(calc): 2768.3149
Variable modifications:
C10 : 4Trioxidation (CMWY)
Ions Score: 94 **Expect:** 2.4e-007
Matches : 20/244 fragment ions using 31 most intense peaks (help)

#	b	b++	b*	b*++	b0	b0++	Seq.	y	y++	y*	y*++	y0	y0++	#
1	114.0913	57.5493					L							23
2	***213.1598***	107.0835					V	2656.2382	1328.6227	2639.2116	1320.1094	2638.2276	1319.6174	22
3	***369.2609***	185.1341	352.2343	176.6208			R	2557.1698	1279.0885	2540.1432	1270.5752	2539.1592	1270.0832	21
4	466.3136	233.6605	449.2871	225.1472			P	2401.0686	1201.0380	2384.0421	1192.5247	2383.0581	1192.0327	20
5	***595.3562***	298.1817	578.3297	289.6685	577.3457	289.1765	E	2304.0159	1152.5116	2286.9893	1143.9983	2286.0053	1143.5063	19
6	***694.4246***	347.7160	677.3981	339.2027	676.4141	338.7107	V	2174.9733	1087.9903	2157.9467	1079.4770	2156.9627	1078.9850	18
7	***809.4516***	405.2294	792.4250	396.7162	791.4410	396.2241	D	2075.9049	1038.4561	2058.8783	1029.9428	2057.8943	1029.4508	17
8	***908.5200***	454.7636	891.4934	446.2504	890.5094	445.7584	V	1960.8779	980.9426	1943.8514	972.4293	1942.8674	971.9373	16
9	***1039.5605***	520.2839	1022.5339	511.7706	1021.5499	511.2786	M	***1861.8095***	931.4084	1844.7830	922.8951	1843.7989	922.4031	15
10	1190.5544	595.7808	1173.5279	587.2676	1172.5438	586.7756	C	***1730.7690***	865.8882	1713.7425	857.3749	1712.7585	856.8829	14
11	1291.6021	646.3047	1274.5755	637.7914	1273.5915	637.2994	T	***1579.7751***	790.3912	1562.7486	781.8779	1561.7645	781.3859	13
12	***1362.6392***	681.8232	1345.6127	673.3100	1344.6286	672.8180	A	***1478.7274***	739.8673	1461.7009	731.3541	1460.7169	730.8621	12
13	1509.7076	755.3574	1492.6811	746.8442	1491.6970	746.3522	F	***1407.6903***	704.3488	1390.6638	695.8355	1389.6797	695.3435	11
14	1646.7665	823.8869	1629.7400	815.3736	1628.7560	814.8816	H	***1260.6219***	630.8146	1243.5953	622.3013	1242.6113	621.8093	10
15	1761.7935	881.4004	1744.7669	872.8871	1743.7829	872.3951	D	***1123.5630***	562.2851	1106.5364	553.7719	1105.5524	553.2798	9
16	1875.8364	938.4218	1858.8098	929.9086	1857.8258	929.4166	N	***1008.5360***	504.7717	991.5095	496.2584	990.5255	495.7664	8
17	2004.8790	1002.9431	1987.8524	994.4299	1986.8684	993.9379	E	***894.4931***	447.7502	877.4666	439.2369	876.4825	438.7449	7
18	2133.9216	1067.4644	2116.8950	1058.9512	2115.9110	1058.4591	E	765.4505	383.2289	748.4240	374.7156	747.4400	374.2236	6
19	2234.9693	1117.9883	2217.9427	1109.4750	2216.9587	1108.9830	T	636.4079	318.7076	619.3814	310.1943	618.3974	309.7023	5

**20**	2382.0377	1191.5225	2365.0111	1183.0092	2364.0271	1182.5172	**F**	***535.3602***	268.1838	518.3337	259.6705			**4**
**21**	2495.1217	1248.0645	2478.0952	1239.5512	2477.1112	1239.0592	**L**	***388.2918***	194.6496	371.2653	186.1363			**3**
**22**	2623.2167	1312.1120	2606.1902	1303.5987	2605.2061	1303.1067	**K**	***275.2078***	138.1075	258.1812	129.5942			**2**
**23**							**K**	147.1128	74.0600	130.0863	65.5468			**1**

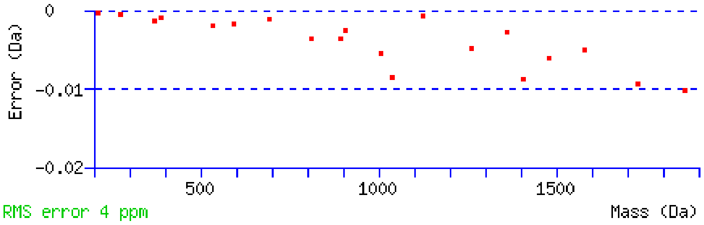

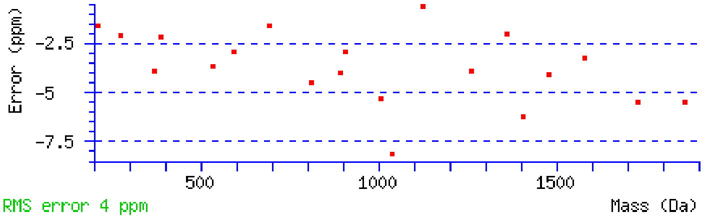

NCBI **BLAST** search of LVRPEVDVMCTAFHDNEETFLKK
(Parameters: blastp, nr protein database, expect=20000, no filter, PAM30)
Other BLAST web gateways

All matches to this query

Score	Mr(calc):	Delta	Sequence
93.5	2768.3149	0.0019	LVRPEVDVMCTAFHDNEETFLKK
77.2	2768.3149	0.0019	LVRPEVDVMCTAFHDNEETFLKK

Mascot Search Results

Peptide View

MS/MS Fragmentation of **RPCFSALEVDETYVPK**
Found in **sp|P02768|ALBU_HUMAN**, Serum albumin OS=Homo sapiens GN=ALB PE=1 SV=2

Match to Query 11558: 1900.886688 from(951.450620,2+) intensity(56712968.0000) scans(14692) rtinseconds(2639) index(12737)
Title: 150818_TTSH_Patient_Plasma_76_Spectrum029881_scans__14692_RTINSECONDS=2639
Data file L:\\Ard_TTSH\\T1D\\T150818_TTSH_Patient_Plasma_76.mgf

Click mouse within plot area to zoom in by factor of two about that point
Or, Plot from 100 to 1700 Da Full range
Label all possible matches ○ Label matches used for scoring ⊙

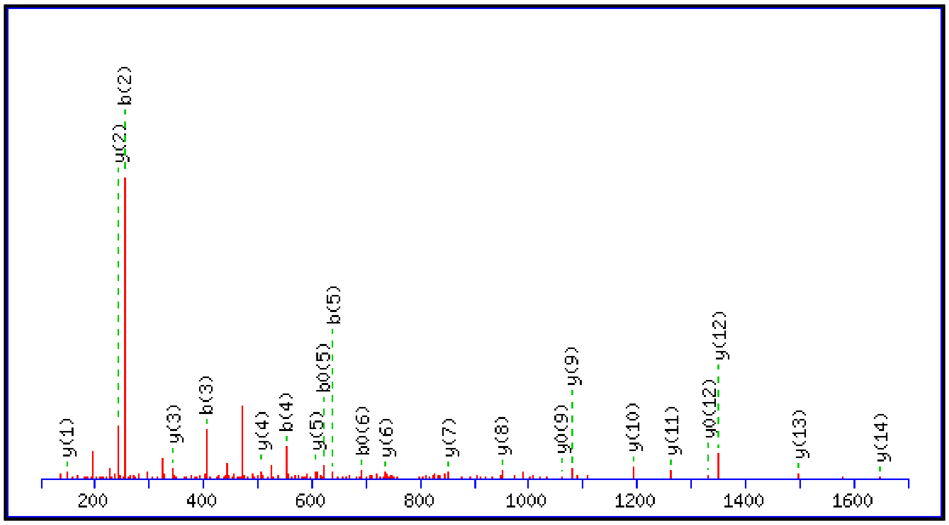

Monoisotopic mass of neutral peptide Mr(calc): 1900.8877
Variable modifications:
C3 : 4Trioxidation (CMWY)
Ions Score: 93 **Expect:** 2.1e-007
Matches : 22/164 fragment ions using 47 most intense peaks (help)

#	b	b^{++}	b*	b^{*++}	b^{0}	b^{0++}	Seq.	y	y^{++}	y*	y^{*++}	y^{0}	y^{0++}	#
1	157.1084	79.0578	140.0818	70.5446			R							16
2	**254.1612**	127.5842	237.1346	119.0709			P	1745.7938	873.4006	1728.7673	864.8873	1727.7833	864.3953	15
3	**405.1551**	203.0812	388.1285	194.5679			C	***1648.7411***	824.8742	1631.7145	816.3609	1630.7305	815.8689	14
4	**552.2235**	276.6154	535.1969	268.1021			F	***1497.7472***	749.3772	1480.7206	740.8639	1479.7366	740.3719	13
5	**639.2555**	320.1314	622.2290	311.6181	621.2450	311.1261	S	***1350.6787***	675.8430	1333.6522	667.3297	1332.6682	666.8377	12
6	710.2926	355.6500	693.2661	347.1367	692.2821	346.6447	A	***1263.6467***	632.3270	1246.6202	623.8137	1245.6361	623.3217	11
7	823.3767	412.1920	806.3502	403.6787	805.3661	403.1867	L	***1192.6096***	596.8084	1175.5830	588.2952	1174.5990	587.8032	10
8	952.4193	476.7133	935.3927	468.2000	934.4087	467.7080	E	***1079.5255***	540.2664	1062.4990	531.7531	1061.5150	531.2611	9
9	1051.4877	526.2475	1034.4612	517.7342	1033.4771	517.2422	V	***950.4829***	475.7451	933.4564	467.2318	932.4724	466.7398	8
10	1166.5147	583.7610	1149.4881	575.2477	1148.5041	574.7557	D	***851.4145***	426.2109	834.3880	417.6976	833.4040	417.2056	7
11	1295.5572	648.2823	1278.5307	639.7690	1277.5467	639.2770	E	***736.3876***	368.6974	719.3610	360.1842	718.3770	359.6921	6
12	1396.6049	698.8061	1379.5784	690.2928	1378.5944	689.8008	T	***607.3450***	304.1761	590.3184	295.6629	589.3344	295.1709	5
13	1559.6683	780.3378	1542.6417	771.8245	1541.6577	771.3325	Y	***506.2973***	253.6523	489.2708	245.1390			4
14	1658.7367	829.8720	1641.7101	821.3587	1640.7261	820.8667	V	***343.2340***	172.1206	326.2074	163.6074			3
15	1755.7894	878.3984	1738.7629	869.8851	1737.7789	869.3931	P	***244.1656***	122.5864	227.1390	114.0731			2
16							K	***147.1128***	74.0600	130.0863	65.5468			1

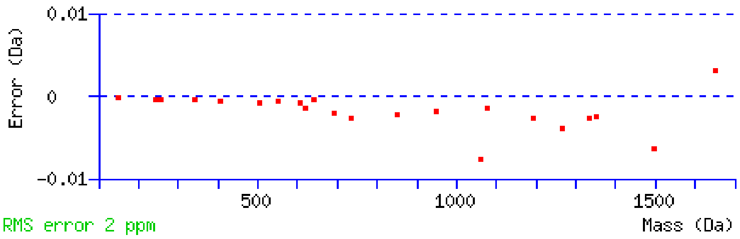

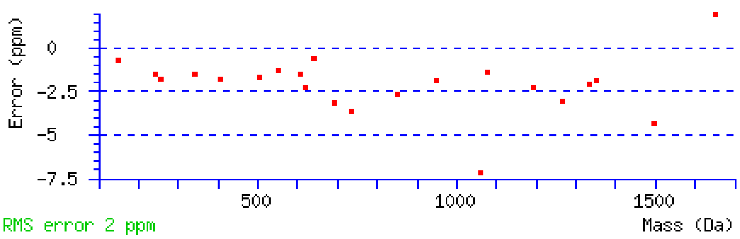

NCBI **BLAST** search of RPCFSALEVDETYVPK
(Parameters: blastp, nr protein database, expect=20000, no filter, PAM30)
Other BLAST web gateways

All matches to this query

Score	Mr(calc):	Delta	Sequence
92.5	1900.8877	-0.0010	RPCFSALEVDETYVPK
1.9	1900.8877	-0.0010	RPCFSALEVDETYVPK

**Mascot:** http://www.matrixscience.com/

MATRIX SCIENCE Mascot Search Results

Peptide View

MS/MS Fragmentation of **FKDLGEENFKALVLIAFAQYLQQCPFEDHVK**
Found in **sp|P02768|ALBU_HUMAN**, Serum albumin OS=Homo sapiens GN=ALB PE=1 SV=2

Match to Query 15254: 3687.828336 from(922.964360,4+) intensity(13059633.0000) scans(17520) rtinseconds(3085) index(14948)
Title: 150801_TTSH_Patient_Plasma_45_Spectrum032374_scans__17520_RTINSECONDS=3085
Data file L:\\Ard_TTSH\\T1D\\T150801_TTSH_Patient_Plasma_45.mgf

Click mouse within plot area to zoom in by factor of two about that point
Or, Plot from 200 to 3200 Da Full range
Label all possible matches ○ Label matches used for scoring ⊙

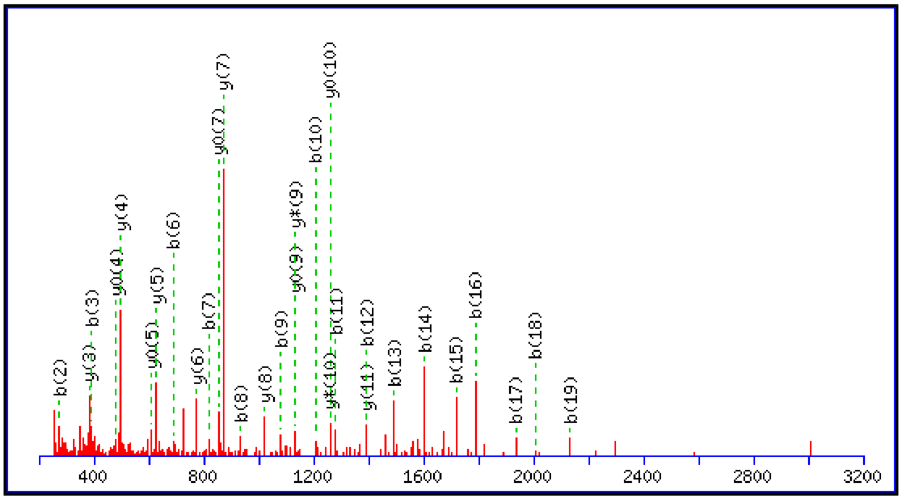

Monoisotopic mass of neutral peptide Mr(calc): 3687.8283
Variable modifications:
C24 : 4Trioxidation (CMWY)
Ions Score: 93 **Expect:** 2.4e-007
Matches : 30/348 fragment ions using 57 most intense peaks (help)

#	b	b^{++}	b*	b^{*++}	b^0	b^{0++}	Seq.	y	y^{++}	y*	y^{*++}	y^0	y^{0++}	#
1	148.0757	74.5415					F							31
2	***276.1707***	138.5890	259.1441	130.0757			K	3541.7672	1771.3872	3524.7406	1762.8740	3523.7566	1762.3819	30
3	***391.1976***	196.1024	374.1710	187.5892	373.1870	187.0972	D	3413.6722	1707.3397	3396.6457	1698.8265	3395.6617	1698.3345	29
4	504.2817	252.6445	487.2551	244.1312	486.2711	243.6392	L	3298.6453	1649.8263	3281.6187	1641.3130	3280.6347	1640.8210	28
5	561.3031	281.1552	544.2766	272.6419	543.2926	272.1499	G	3185.5612	1593.2842	3168.5347	1584.7710	3167.5507	1584.2790	27
6	***690.3457***	345.6765	673.3192	337.1632	672.3352	336.6712	E	3128.5398	1564.7735	3111.5132	1556.2602	3110.5292	1555.7682	26
7	***819.3883***	410.1978	802.3618	401.6845	801.3777	401.1925	E	2999.4972	1500.2522	2982.4706	1491.7389	2981.4866	1491.2469	25
8	***933.4312***	467.2193	916.4047	458.7060	915.4207	458.2140	N	2870.4546	1435.7309	2853.4280	1427.2176	2852.4440	1426.7256	24
9	***1080.4997***	540.7535	1063.4731	532.2402	1062.4891	531.7482	F	2756.4116	1378.7095	2739.3851	1370.1962	2738.4011	1369.7042	23
10	***1208.5946***	604.8009	1191.5681	596.2877	1190.5840	595.7957	K	2609.3432	1305.1753	2592.3167	1296.6620	2591.3327	1296.1700	22
11	***1279.6317***	640.3195	1262.6052	631.8062	1261.6212	631.3142	A	2481.2483	1241.1278	2464.2217	1232.6145	2463.2377	1232.1225	21
12	***1392.7158***	696.8615	1375.6892	688.3483	1374.7052	687.8563	L	2410.2111	1205.6092	2393.1846	1197.0959	2392.2006	1196.6039	20
13	***1491.7842***	746.3957	1474.7577	737.8825	1473.7736	737.3905	V	2297.1271	1149.0672	2280.1005	1140.5539	2279.1165	1140.0619	19
14	***1604.8683***	802.9378	1587.8417	794.4245	1586.8577	793.9325	L	2198.0587	1099.5330	2181.0321	1091.0197	2180.0481	1090.5277	18
15	***1717.9523***	859.4798	1700.9258	850.9665	1699.9418	850.4745	I	2084.9746	1042.9909	2067.9481	1034.4777	2066.9640	1033.9857	17
16	***1788.9894***	894.9984	1771.9629	886.4851	1770.9789	885.9931	A	1971.8905	986.4489	1954.8640	977.9356	1953.8800	977.4436	16
17	***1936.0579***	968.5326	1919.0313	960.0193	1918.0473	959.5273	F	1900.8534	950.9304	1883.8269	942.4171	1882.8429	941.9251	15
18	***2007.0950***	1004.0511	1990.0684	995.5379	1989.0844	995.0458	A	1753.7850	877.3961	1736.7585	868.8829	1735.7744	868.3909	14
19	***2135.1536***	1068.0804	2118.1270	1059.5671	2117.1430	1059.0751	Q	1682.7479	841.8776	1665.7214	833.3643	1664.7373	832.8723	13

20	2298.2169	1149.6121	2281.1903	1141.0988	2280.2063	1140.6068	Y	1554.6893	777.8483	1537.6628	769.3350	1536.6788	768.8430	12
21	2411.3009	1206.1541	2394.2744	1197.6408	2393.2904	1197.1488	L	1391.6260	696.3166	1374.5994	687.8034	1373.6154	687.3114	11
22	2539.3595	1270.1834	2522.3330	1261.6701	2521.3490	1261.1781	Q	1278.5419	639.7746	1261.5154	631.2613	1260.5314	630.7693	10
23	2667.4181	1334.2127	2650.3916	1325.6994	2649.4075	1325.2074	Q	1150.4834	575.7453	1133.4568	567.2320	1132.4728	566.7400	9
24	2818.4120	1409.7097	2801.3855	1401.1964	2800.4015	1400.7044	C	1022.4248	511.7160	1005.3982	503.2027	1004.4142	502.7107	8
25	2915.4648	1458.2360	2898.4382	1449.7228	2897.4542	1449.2308	P	871.4308	436.2191	854.4043	427.7058	853.4203	427.2138	7
26	3062.5332	1531.7702	3045.5067	1523.2570	3044.5226	1522.7650	F	774.3781	387.6927	757.3515	379.1794	756.3675	378.6874	6
27	3191.5758	1596.2915	3174.5493	1587.7783	3173.5652	1587.2863	E	627.3097	314.1585	610.2831	305.6452	609.2991	305.1532	5
28	3306.6027	1653.8050	3289.5762	1645.2917	3288.5922	1644.7997	D	498.2671	249.6372	481.2405	241.1239	480.2565	240.6319	4
29	3443.6617	1722.3345	3426.6351	1713.8212	3425.6511	1713.3292	H	383.2401	192.1237	366.2136	183.6104			3
30	3542.7301	1771.8687	3525.7035	1763.3554	3524.7195	1762.8634	V	246.1812	123.5942	229.1547	115.0810			2
31							K	147.1128	74.0600	130.0863	65.5468			1

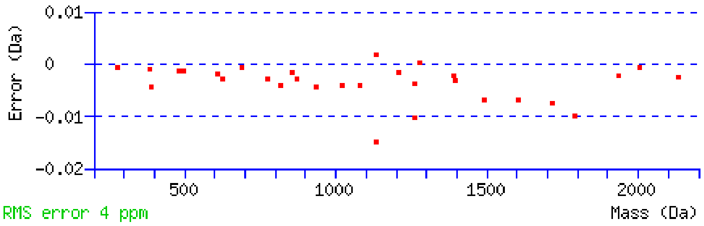

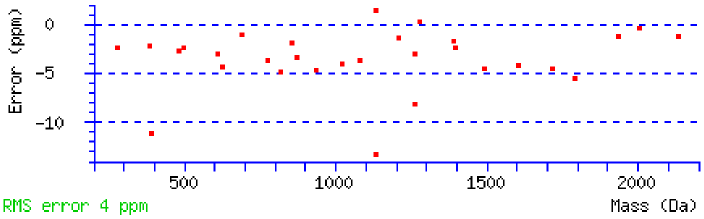

NCBI **BLAST** search of FKDLGEENFKALVLIAFAQYLQQCPFEDHVK
(Parameters: blastp, nr protein database, expect=20000, no filter, PAM30)
Other BLAST web gateways

All matches to this query

Score	Mr(calc):	Delta	Sequence
92.5	3687.8283	0.0000	FKDLGEENFKALVLIAFAQYLQQCPFEDHVK
74.4	3687.8283	0.0000	FKDLGEENFKALVLIAFAQYLQQCPFEDHVK

Mascot Search Results

Peptide View

MS/MS Fragmentation of **ALVLIAFAQYLQQCPFEDHVK**
Found in **sp|P02768|ALBU_HUMAN**, Serum albumin OS=Homo sapiens GN=ALB PE=1 SV=2

Match to Query 12027: 2480.242452 from(827.754760,3+) intensity(2998150.5000) scans(18020) rtinseconds(3115) index(15413)
Title: 150818_TTSH_Patient_Plasma_35_Spectrum033685_scans__18020_RTINSECONDS=3115
Data file L:\\Ard_TTSH\\T1D\\T150818_TTSH_Patient_Plasma_35.mgf

Click mouse within plot area to zoom in by factor of two about that point
Or, Plot from 100 to 2000 Da Full range
Label all possible matches ○ Label matches used for scoring ⊙

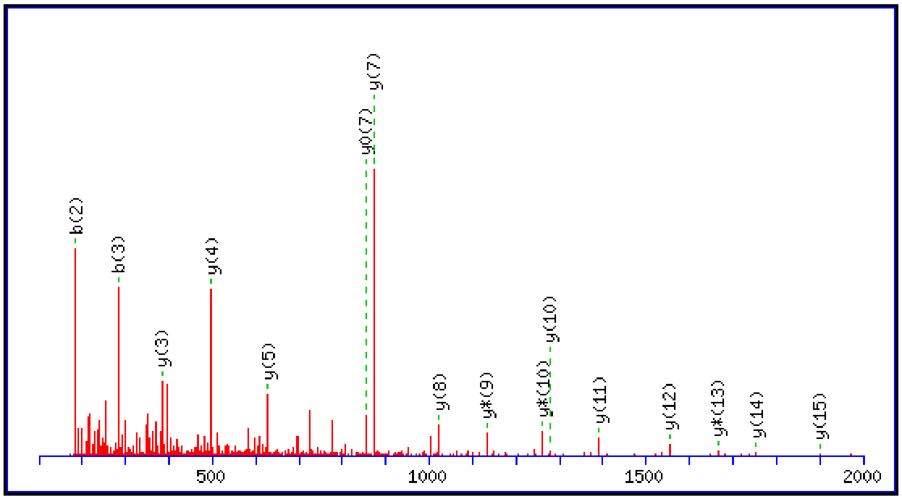

Monoisotopic mass of neutral peptide Mr(calc): 2480.2410
Variable modifications:
C14 : 4Trioxidation (CMWY)
Ions Score: 87 **Expect:** 8.3e-007
Matches : 16/186 fragment ions using 18 most intense peaks (help)

#	b	b++	b*	b*++	b0	b0++	Seq.	y	y++	y*	y*++	y0	y0++	#
1	72.0444	36.5258					A							21
2	**185.1285**	93.0679					L	2410.2111	1205.6092	2393.1846	1197.0959	2392.2006	1196.6039	20
3	**284.1969**	142.6021					V	2297.1271	1149.0672	2280.1005	1140.5539	2279.1165	1140.0619	19
4	397.2809	199.1441					L	2198.0587	1099.5330	2181.0321	1091.0197	2180.0481	1090.5277	18
5	510.3650	255.6861					I	2084.9746	1042.9909	2067.9481	1034.4777	2066.9640	1033.9857	17
6	581.4021	291.2047					A	1971.8905	986.4489	1954.8640	977.9356	1953.8800	977.4436	16
7	728.4705	364.7389					F	***1900.8534***	950.9304	1883.8269	942.4171	1882.8429	941.9251	15
8	799.5076	400.2575					A	***1753.7850***	877.3961	1736.7585	868.8829	1735.7744	868.3909	14
9	927.5662	464.2867	910.5397	455.7735			Q	1682.7479	841.8776	1665.7214	833.3643	1664.7373	832.8723	13
10	1090.6295	545.8184	1073.6030	537.3051			Y	***1554.6893***	777.8483	1537.6628	769.3350	1536.6788	768.8430	12
11	1203.7136	602.3604	1186.6871	593.8472			L	***1391.6260***	696.3166	1374.5994	687.8034	1373.6154	687.3114	11
12	1331.7722	666.3897	1314.7456	657.8765			Q	***1278.5419***	639.7746	1261.5154	631.2613	1260.5314	630.7693	10
13	1459.8308	730.4190	1442.8042	721.9057			Q	1150.4834	575.7453	1133.4568	567.2320	1132.4728	566.7400	9
14	1610.8247	805.9160	1593.7981	797.4027			C	***1022.4248***	511.7160	1005.3982	503.2027	1004.4142	502.7107	8
15	1707.8775	854.4424	1690.8509	845.9291			P	***871.4308***	436.2191	854.4043	427.7058	853.4203	427.2138	7
16	1854.9459	927.9766	1837.9193	919.4633			F	774.3781	387.6927	757.3515	379.1794	756.3675	378.6874	6
17	1983.9885	992.4979	1966.9619	983.9846	1965.9779	983.4926	E	***627.3097***	314.1585	610.2831	305.6452	609.2991	305.1532	5
18	2099.0154	1050.0113	2081.9889	1041.4981	2081.0048	1041.0061	D	***498.2671***	249.6372	481.2405	241.1239	480.2565	240.6319	4
19	2236.0743	1118.5408	2219.0478	1110.0275	2218.0638	1109.5355	H	***383.2401***	192.1237	366.2136	183.6104			3

**20**	2335.1427	1168.0750	2318.1162	1159.5617	2317.1322	1159.0697	**V**	246.1812	123.5942	229.1547	115.0810			**2**
**21**							**K**	147.1128	74.0600	130.0863	65.5468			**1**

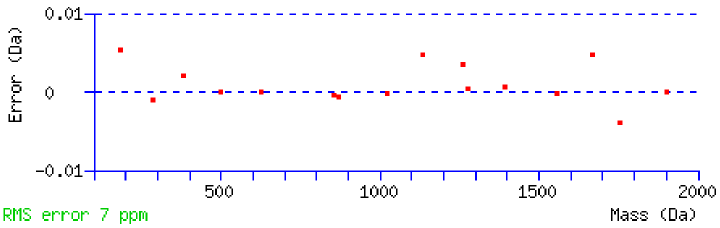

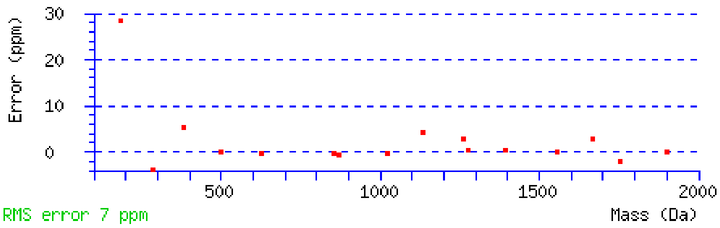

NCBI **BLAST** search of ALVLIAFAQYLQQCPFEDHVK
(Parameters: blastp, nr protein database, expect=20000, no filter, PAM30)
Other BLAST web gateways

All matches to this query

Score	Mr(calc):	Delta	Sequence
86.7	2480.2410	0.0015	ALVLIAFAQYLQQCPFEDHVK
39.0	2480.2410	0.0015	ALVLIAFAQYLQQCPFEDHVK

Mascot: http://www.matrixscience.com/

Mascot Search Results

Peptide View

MS/MS Fragmentation of **QNCELFEQLGEYK**
Found in **sp|P02768|ALBU_HUMAN**, Serum albumin OS=Homo sapiens GN=ALB PE=1 SV=2

Match to Query 5807: 1647.707008 from(824.860780,2+) intensity(1007369.4375) scans(14337) rtinseconds(2607) index(12103)
Title: 150801_TTSH_Patient_Plasma_37_Spectrum029017_scans__14337_RTINSECONDS=2607
Data file L:\\Ard_TTSH\\T1D\\T150801_TTSH_Patient_Plasma_37.mgf

Click mouse within plot area to zoom in by factor of two about that point
Or, [Plot from] 100 to 1300 Da [Full range]
Label all possible matches ○ Label matches used for scoring ⊙

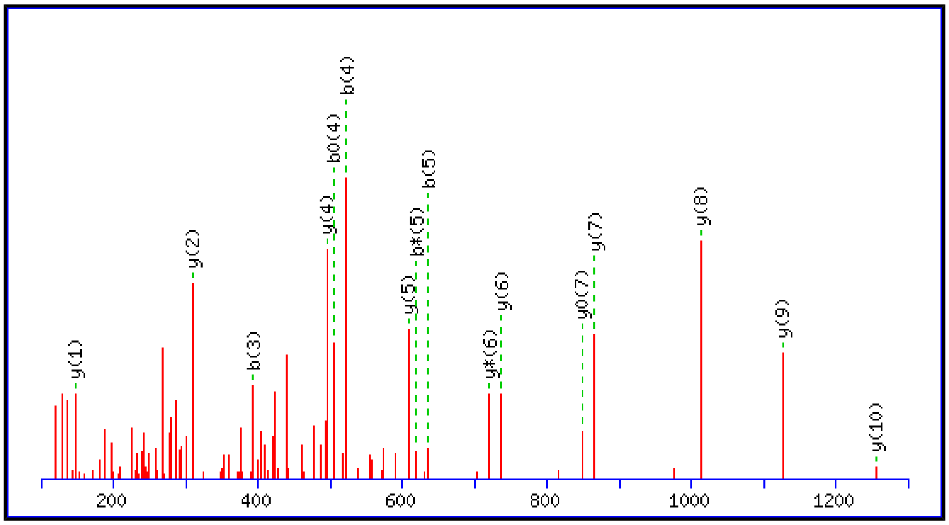

Monoisotopic mass of neutral peptide Mr(calc): 1647.7086
Variable modifications:
C3 : 4Trioxidation (CMWY)
Ions Score: 78 **Expect:** 1.1e-005
Matches : 16/134 fragment ions using 20 most intense peaks (help)

#	b	b^{++}	b^{*}	b^{*++}	b^{0}	b^{0++}	Seq.	y	y^{++}	y^{*}	y^{*++}	y^{0}	y^{0++}	#
1	129.0659	65.0366	112.0393	56.5233			Q							13
2	243.1088	122.0580	226.0822	113.5448			N	1520.6574	760.8323	1503.6308	752.3190	1502.6468	751.8270	12
3	**394.1027**	197.5550	377.0762	189.0417			C	1406.6144	703.8109	1389.5879	695.2976	1388.6039	694.8056	11
4	**523.1453**	262.0763	506.1188	253.5630	505.1347	253.0710	E	***1255.6205***	628.3139	1238.5939	619.8006	1237.6099	619.3086	10
5	**636.2294**	318.6183	619.2028	310.1050	618.2188	309.6130	L	***1126.5779***	563.7926	1109.5514	555.2793	1108.5673	554.7873	9
6	783.2978	392.1525	766.2712	383.6393	765.2872	383.1472	F	***1013.4938***	507.2506	996.4673	498.7373	995.4833	498.2453	8
7	912.3404	456.6738	895.3138	448.1606	894.3298	447.6685	E	***866.4254***	433.7164	849.3989	425.2031	848.4149	424.7111	7
8	1040.3990	520.7031	1023.3724	512.1898	1022.3884	511.6978	Q	***737.3828***	369.1951	720.3563	360.6818	719.3723	360.1898	6
9	1153.4830	577.2451	1136.4565	568.7319	1135.4725	568.2399	L	***609.3243***	305.1658	592.2977	296.6525	591.3137	296.1605	5
10	1210.5045	605.7559	1193.4779	597.2426	1192.4939	596.7506	G	***496.2402***	248.6237	479.2136	240.1105	478.2296	239.6185	4
11	1339.5471	670.2772	1322.5205	661.7639	1321.5365	661.2719	E	439.2187	220.1130	422.1922	211.5997	421.2082	211.1077	3
12	1502.6104	751.8088	1485.5839	743.2956	1484.5998	742.8036	Y	***310.1761***	155.5917	293.1496	147.0784			2
13							K	***147.1128***	74.0600	130.0863	65.5468			1

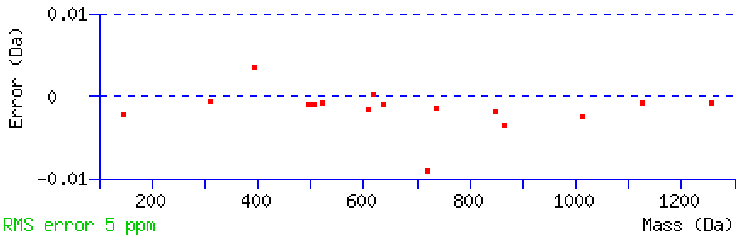

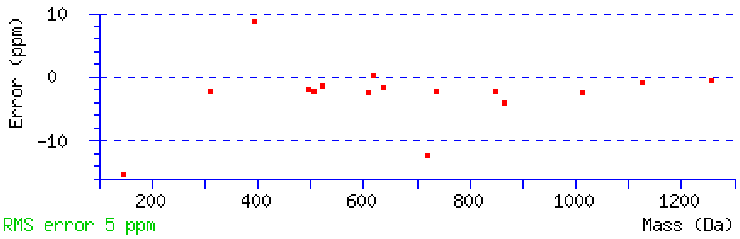

NCBI **BLAST** search of QNCELFEQLGEYK
(Parameters: blastp, nr protein database, expect=20000, no filter, PAM30)
Other BLAST web gateways

All matches to this query

Score	Mr(calc):	Delta	Sequence
78.5	1647.7086	-0.0016	QNCELFEQLGEYK

**Mascot:** http://www.matrixscience.com/

Mascot Search Results

Peptide View

MS/MS Fragmentation of **LVRPEVDVMCTAFHDNEETFLK**
Found in **sp|P02768|ALBU_HUMAN**, Serum albumin OS=Homo sapiens GN=ALB PE=1 SV=2

Match to Query 13594: 2640.215472 from(881.079100,3+) intensity(2163006.7500) scans(15187) rtinseconds(2653) index(13467)
Title: 150825_TTSH_Patient_Plasma_07_Spectrum031326_scans__15187_RTINSECONDS=2653
Data file L:\\Ard_TTSH\\T1D\\T150825_TTSH_Patient_Plasma_07.mgf

Click mouse within plot area to zoom in by factor of two about that point
Or, [Plot from] [0] to [2600] Da [Full range]
Label all possible matches ○ Label matches used for scoring ⦿

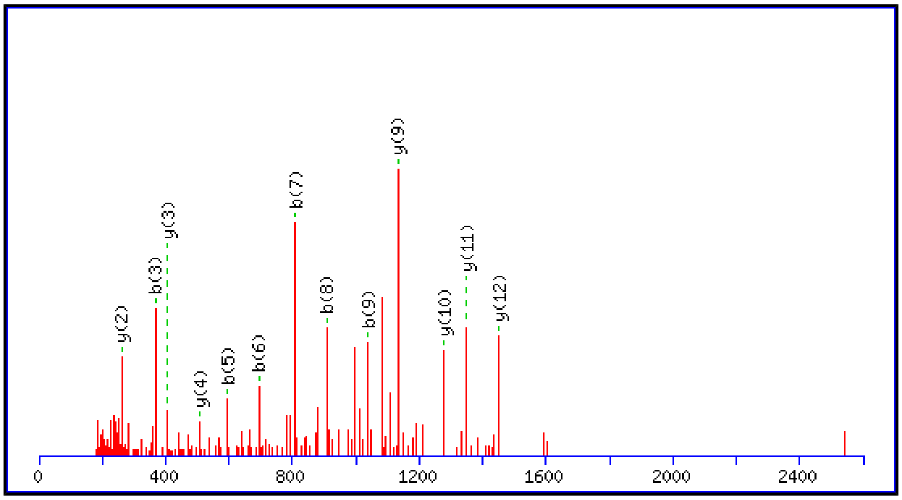

Monoisotopic mass of neutral peptide Mr(calc): 2640.2200
Variable modifications:
C10 : 4Trioxidation (CMWY)
Ions Score: 77 **Expect:** 1.7e-005
Matches : 13/234 fragment ions using 15 most intense peaks (help)

#	b	b^{++}	b*	b^{*++}	b^0	b^{0++}	Seq.	y	y^{++}	y*	y^{*++}	y^0	y^{0++}	#
1	114.0913	57.5493					L							22
2	213.1598	107.0835					V	2528.1432	1264.5752	2511.1167	1256.0620	2510.1326	1255.5700	21
3	***369.2609***	185.1341	352.2343	176.6208			R	2429.0748	1215.0410	2412.0482	1206.5278	2411.0642	1206.0357	20
4	466.3136	233.6605	449.2871	225.1472			P	2272.9737	1136.9905	2255.9471	1128.4772	2254.9631	1127.9852	19
5	***595.3562***	298.1817	578.3297	289.6685	577.3457	289.1765	E	2175.9209	1088.4641	2158.8944	1079.9508	2157.9103	1079.4588	18
6	***694.4246***	347.7160	677.3981	339.2027	676.4141	338.7107	V	2046.8783	1023.9428	2029.8518	1015.4295	2028.8678	1014.9375	17
7	***809.4516***	405.2294	792.4250	396.7162	791.4410	396.2241	D	1947.8099	974.4086	1930.7834	965.8953	1929.7993	965.4033	16
8	***908.5200***	454.7636	891.4934	446.2504	890.5094	445.7584	V	1832.7830	916.8951	1815.7564	908.3818	1814.7724	907.8898	15
9	***1039.5605***	520.2839	1022.5339	511.7706	1021.5499	511.2786	M	1733.7145	867.3609	1716.6880	858.8476	1715.7040	858.3556	14
10	1190.5544	595.7808	1173.5279	587.2676	1172.5438	586.7756	C	1602.6741	801.8407	1585.6475	793.3274	1584.6635	792.8354	13
11	1291.6021	646.3047	1274.5755	637.7914	1273.5915	637.2994	T	***1451.6801***	726.3437	1434.6536	717.8304	1433.6696	717.3384	12
12	1362.6392	681.8232	1345.6127	673.3100	1344.6286	672.8180	A	***1350.6325***	675.8199	1333.6059	667.3066	1332.6219	666.8146	11
13	1509.7076	755.3574	1492.6811	746.8442	1491.6970	746.3522	F	***1279.5953***	640.3013	1262.5688	631.7880	1261.5848	631.2960	10
14	1646.7665	823.8869	1629.7400	815.3736	1628.7560	814.8816	H	***1132.5269***	566.7671	1115.5004	558.2538	1114.5164	557.7618	9
15	1761.7935	881.4004	1744.7669	872.8871	1743.7829	872.3951	D	995.4680	498.2376	978.4415	489.7244	977.4575	489.2324	8
16	1875.8364	938.4218	1858.8098	929.9086	1857.8258	929.4166	N	880.4411	440.7242	863.4145	432.2109	862.4305	431.7189	7
17	2004.8790	1002.9431	1987.8524	994.4299	1986.8684	993.9379	E	766.3981	383.7027	749.3716	375.1894	748.3876	374.6974	6
18	2133.9216	1067.4644	2116.8950	1058.9512	2115.9110	1058.4591	E	637.3556	319.1814	620.3290	310.6681	619.3450	310.1761	5
19	2234.9693	1117.9883	2217.9427	1109.4750	2216.9587	1108.9830	T	***508.3130***	254.6601	491.2864	246.1468	490.3024	245.6548	4

**20**	2382.0377	1191.5225	2365.0111	1183.0092	2364.0271	1182.5172	**F**	***407.2653***	204.1363	390.2387	195.6230			**3**
**21**	2495.1217	1248.0645	2478.0952	1239.5512	2477.1112	1239.0592	**L**	***260.1969***	130.6021	243.1703	122.0888			**2**
**22**							**K**	147.1128	74.0600	130.0863	65.5468			**1**

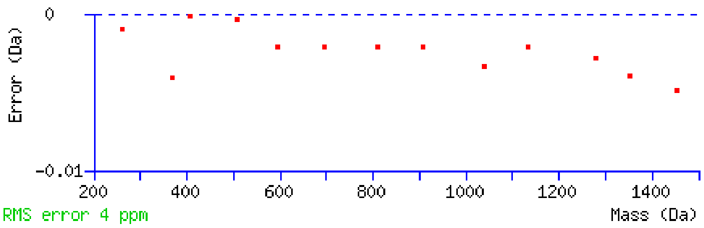

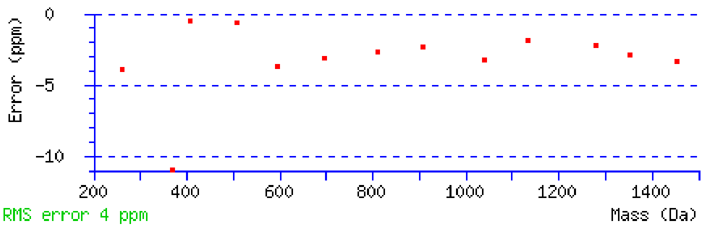

NCBI **BLAST** search of LVRPEVDVMCTAFHDNEETFLK
(Parameters: blastp, nr protein database, expect=20000, no filter, PAM30)
Other BLAST web gateways

All matches to this query

Score	Mr(calc):	Delta	Sequence
76.5	2640.2200	-0.0045	LVRPEVDVMCTAFHDNEETFLK
63.8	2640.2200	-0.0045	LVRPEVDVMCTAFHDNEETFLK

Mascot: http://www.matrixscience.com/

Mascot Search Results

Peptide View

MS/MS Fragmentation of **SLHTLFGDKLCTVATLR**
Found in **sp|P02768|ALBU_HUMAN**, Serum albumin OS=Homo sapiens GN=ALB PE=1 SV=2

Match to Query 9362: 1921.990512 from(641.670780,3+) intensity(899308.2500) scans(13379) rtinseconds(2398) index(11020)
Title: 150801_TTSH_Patient_Plasma_54_Spectrum027352_scans__13379_RTINSECONDS=2398
Data file L:\\Ard_TTSH\\T1D\\T150801_TTSH_Patient_Plasma_54.mgf

Click mouse within plot area to zoom in by factor of two about that point
Or, Plot from 100 to 1900 Da Full range
Label all possible matches ○ Label matches used for scoring ⊙

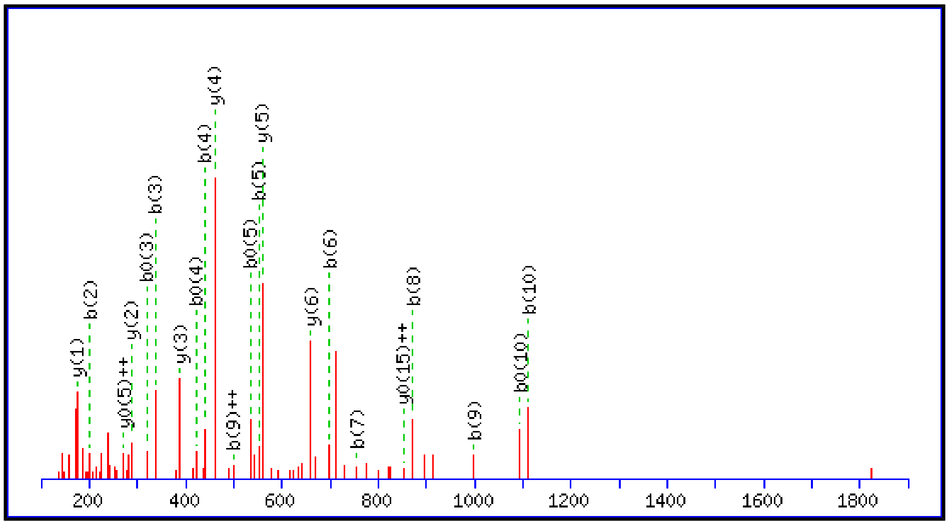

Monoisotopic mass of neutral peptide Mr(calc): 1921.9931
Variable modifications:
C11 : 4Trioxidation (CMWY)
Ions Score: 75 **Expect:** 7.8e-006
Matches : 23/172 fragment ions using 36 most intense peaks (help)

#	b	b^{++}	b*	b^{*++}	b^0	b^{0++}	Seq.	y	y^{++}	y*	y^{*++}	y^0	y^{0++}	#
1	88.0393	44.5233			70.0287	35.5180	S							17
2	201.1234	101.0653			183.1128	92.0600	L	1835.9684	918.4878	1818.9418	909.9746	1817.9578	909.4826	16
3	338.1823	169.5948			320.1717	160.5895	H	1722.8843	861.9458	1705.8578	853.4325	1704.8738	852.9405	15
4	439.2300	220.1186			421.2194	211.1133	T	1585.8254	793.4163	1568.7989	784.9031	1567.8149	784.4111	14
5	552.3140	276.6606			534.3035	267.6554	L	1484.7777	742.8925	1467.7512	734.3792	1466.7672	733.8872	13
6	699.3824	350.1949			681.3719	341.1896	F	1371.6937	686.3505	1354.6671	677.8372	1353.6831	677.3452	12
7	756.4039	378.7056			738.3933	369.7003	G	1224.6253	612.8163	1207.5987	604.3030	1206.6147	603.8110	11
8	871.4308	436.2191			853.4203	427.2138	D	1167.6038	584.3055	1150.5773	575.7923	1149.5932	575.3003	10
9	999.5258	500.2665	982.4993	491.7533	981.5152	491.2613	K	1052.5769	526.7921	1035.5503	518.2788	1034.5663	517.7868	9
10	1112.6099	556.8086	1095.5833	548.2953	1094.5993	547.8033	L	924.4819	462.7446	907.4553	454.2313	906.4713	453.7393	8
11	1263.6038	632.3055	1246.5773	623.7923	1245.5932	623.3003	C	811.3978	406.2026	794.3713	397.6893	793.3873	397.1973	7
12	1364.6515	682.8294	1347.6249	674.3161	1346.6409	673.8241	T	660.4039	330.7056	643.3774	322.1923	642.3933	321.7003	6
13	1463.7199	732.3636	1446.6933	723.8503	1445.7093	723.3583	V	559.3562	280.1817	542.3297	271.6685	541.3457	271.1765	5
14	1534.7570	767.8821	1517.7305	759.3689	1516.7464	758.8769	A	460.2878	230.6475	443.2613	222.1343	442.2772	221.6423	4
15	1635.8047	818.4060	1618.7781	809.8927	1617.7941	809.4007	T	389.2507	195.1290	372.2241	186.6157	371.2401	186.1237	3
16	1748.8887	874.9480	1731.8622	866.4347	1730.8782	865.9427	L	288.2030	144.6051	271.1765	136.0919			2
17							R	175.1190	88.0631	158.0924	79.5498			1

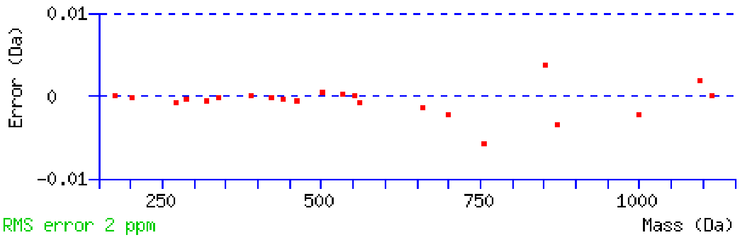

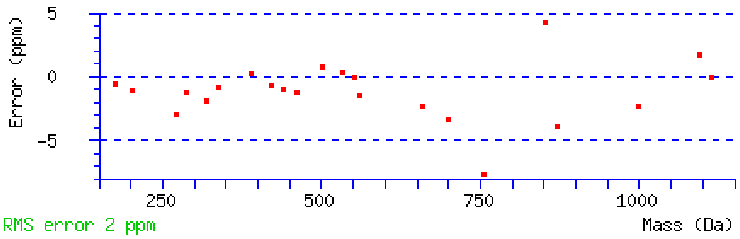

NCBI **BLAST** search of SLHTLFGDKLCTVATLR
(Parameters: blastp, nr protein database, expect=20000, no filter, PAM30)
Other BLAST web gateways

All matches to this query

Score	Mr(calc):	Delta	Sequence
75.4	1921.9931	-0.0026	SLHTLFGDKLCTVATLR
3.6	1919.9927	1.9978	CDTVYRKYLYVLATR
2.8	1921.9891	0.0014	NMTGLVDLTLSRNAITR
1.7	1921.9785	0.0120	EVVLDSWPDFKAVITR

Mascot Search Results

Peptide View

MS/MS Fragmentation of **YICENQDSISSK**
Found in **sp|P02768|ALBU_HUMAN**, Serum albumin OS=Homo sapiens GN=ALB PE=1 SV=2

Match to Query 5504: 1433.598368 from(717.806460,2+) intensity(5163122.5000) scans(7564) rtinseconds(1480) index(5860)
Title: 150808_TTSH_Patient_Plasma_54_Spectrum021590_scans__7564_RTINSECONDS=1480
Data file L:\\Ard_TTSH\\T1D\\T150808_TTSH_Patient_Plasma_54.mgf

Click mouse within plot area to zoom in by factor of two about that point
Or, Plot from 100 to 1200 Da Full range
Label all possible matches ○ Label matches used for scoring ⊙

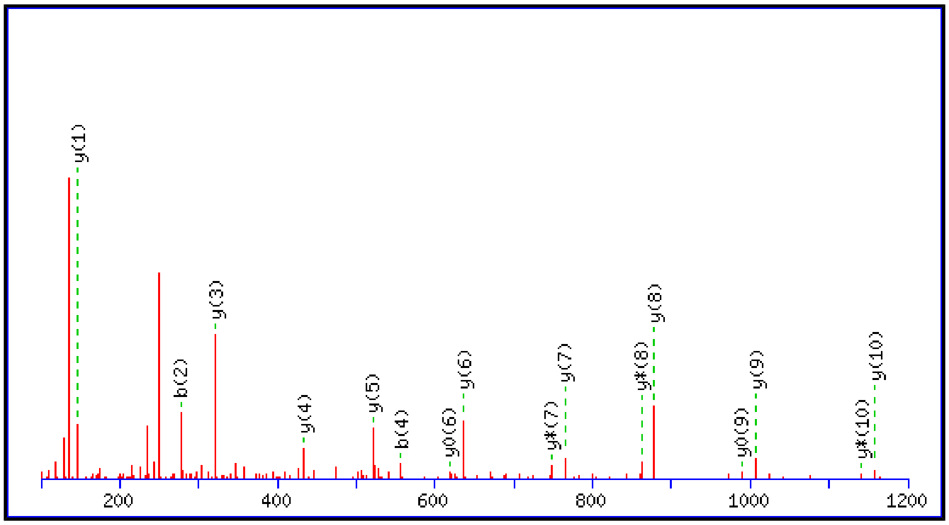


```
Monoisotopic mass of neutral peptide Mr(calc): 1433.5980
Variable modifications:
C3     : 4Trioxidation (CMWY)
Ions Score: 75  Expect: 6.1e-006
Matches : 16/116 fragment ions using 22 most intense peaks   (help)
```

#	b	b++	b*	b*++	b0	b0++	Seq.	y	y++	y*	y*++	y0	y0++	#
1	164.0706	82.5389					Y							12
2	**277.1547**	139.0810					I	1271.5420	636.2746	1254.5154	627.7614	1253.5314	627.2693	11
3	428.1486	214.5779					C	***1158.4579***	579.7326	1141.4314	571.2193	1140.4474	570.7273	10
4	**557.1912**	279.0992			539.1806	270.0940	E	***1007.4640***	504.2356	990.4374	495.7224	989.4534	495.2304	9
5	671.2341	336.1207	654.2076	327.6074	653.2236	327.1154	N	***878.4214***	439.7143	861.3949	431.2011	860.4108	430.7091	8
6	799.2927	400.1500	782.2661	391.6367	781.2821	391.1447	Q	***764.3785***	382.6929	747.3519	374.1796	746.3679	373.6876	7
7	914.3196	457.6635	897.2931	449.1502	896.3091	448.6582	D	***636.3199***	318.6636	619.2933	310.1503	618.3093	309.6583	6
8	1001.3517	501.1795	984.3251	492.6662	983.3411	492.1742	S	***521.2930***	261.1501	504.2664	252.6368	503.2824	252.1448	5
9	1114.4357	557.7215	1097.4092	549.2082	1096.4252	548.7162	I	***434.2609***	217.6341	417.2344	209.1208	416.2504	208.6288	4
10	1201.4678	601.2375	1184.4412	592.7242	1183.4572	592.2322	S	***321.1769***	161.0921	304.1503	152.5788	303.1663	152.0868	3
11	1288.4998	644.7535	1271.4732	636.2403	1270.4892	635.7482	S	234.1448	117.5761	217.1183	109.0628	216.1343	108.5708	2
12							K	***147.1128***	74.0600	130.0863	65.5468			1

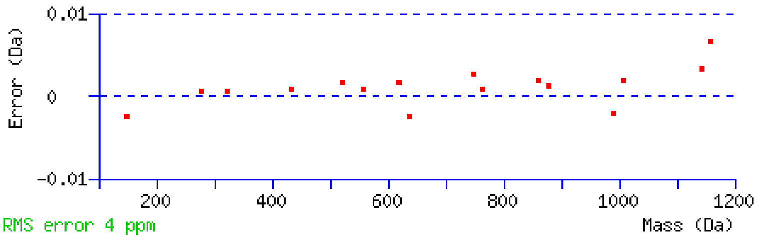

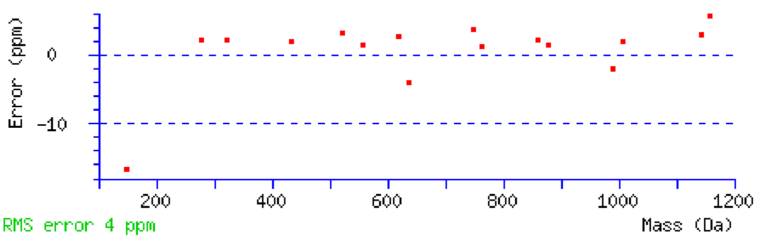

NCBI **BLAST** search of YICENQDSISSK
(Parameters: blastp, nr protein database, expect=20000, no filter, PAM30)
Other BLAST web gateways

All matches to this query

Score	Mr(calc):	Delta	Sequence
75.0	1433.5980	0.0004	YICENQDSISSK
54.9	1433.5980	0.0004	YICENQDSISSK
0.4	1431.6041	1.9942	XEESENEFYIK

Mascot: http://www.matrixscience.com/

Mascot Search Results

Peptide View

MS/MS Fragmentation of **ADDKETCFAEEGKK**
Found in **sp|P02768|ALBU_HUMAN**, Serum albumin OS=Homo sapiens GN=ALB PE=1 SV=2

Match to Query 7414: 1617.681608 from(809.848080,2+) intensity(7392763.0000) scans(1345) rtinseconds(276) index(1046)
Title: 150801_TTSH_Patient_Plasma_40_Spectrum017981_scans__1345_RTINSECONDS=276
Data file L:\\Ard_TTSH\\T1D\\T150801_TTSH_Patient_Plasma_40.mgf

Click mouse within plot area to zoom in by factor of two about that point
Or, Plot from 100 to 1700 Da Full range
Label all possible matches ○ Label matches used for scoring ⦿

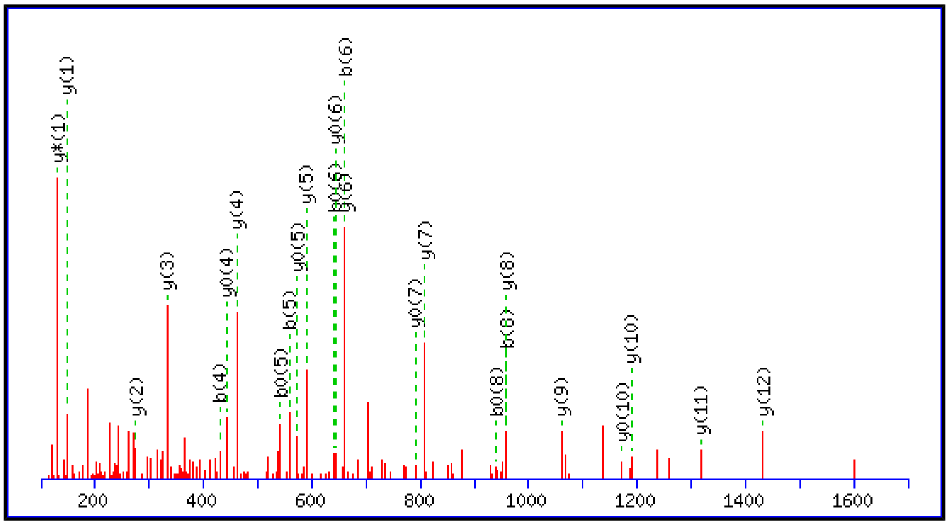

Monoisotopic mass of neutral peptide Mr(calc): 1617.6828
Variable modifications:
C7 : 4Trioxidation (CMWY)
Ions Score: 73 **Expect:** 9.5e-006
Matches : 25/142 fragment ions using 57 most intense peaks (help)

#	b	b^{++}	b*	b^{*++}	b^0	b^{0++}	Seq.	y	y^{++}	y*	y^{*++}	y^0	y^{0++}	#
1	72.0444	36.5258					A							14
2	187.0713	94.0393			169.0608	85.0340	D	1547.6530	774.3301	1530.6264	765.8169	1529.6424	765.3249	13
3	302.0983	151.5528			284.0877	142.5475	D	***1432.6261***	716.8167	1415.5995	708.3034	1414.6155	707.8114	12
4	**430.1932**	215.6003	413.1667	207.0870	412.1827	206.5950	K	***1317.5991***	659.3032	1300.5726	650.7899	1299.5885	650.2979	11
5	**559.2358**	280.1216	542.2093	271.6083	541.2253	271.1163	E	***1189.5041***	595.2557	1172.4776	586.7424	1171.4936	586.2504	10
6	**660.2835**	330.6454	643.2570	322.1321	642.2729	321.6401	T	***1060.4616***	530.7344	1043.4350	522.2211	1042.4510	521.7291	9
7	811.2774	406.1424	794.2509	397.6291	793.2669	397.1371	C	***959.4139***	480.2106	942.3873	471.6973	941.4033	471.2053	8
8	**958.3459**	479.6766	941.3193	471.1633	940.3353	470.6713	F	***808.4199***	404.7136	791.3934	396.2003	790.4094	395.7083	7
9	1029.3830	515.1951	1012.3564	506.6818	1011.3724	506.1898	A	***661.3515***	331.1794	644.3250	322.6661	643.3410	322.1741	6
10	1158.4256	579.7164	1141.3990	571.2031	1140.4150	570.7111	E	***590.3144***	295.6608	573.2879	287.1476	572.3039	286.6556	5
11	1287.4682	644.2377	1270.4416	635.7244	1269.4576	635.2324	E	***461.2718***	231.1396	444.2453	222.6263	443.2613	222.1343	4
12	1344.4896	672.7484	1327.4631	664.2352	1326.4791	663.7432	G	***332.2292***	166.6183	315.2027	158.1050			3
13	1472.5846	736.7959	1455.5580	728.2827	1454.5740	727.7906	K	***275.2078***	138.1075	258.1812	129.5942			2
14							K	***147.1128***	74.0600	130.0863	65.5468			1

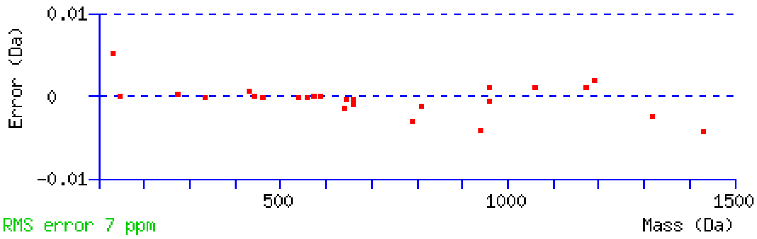

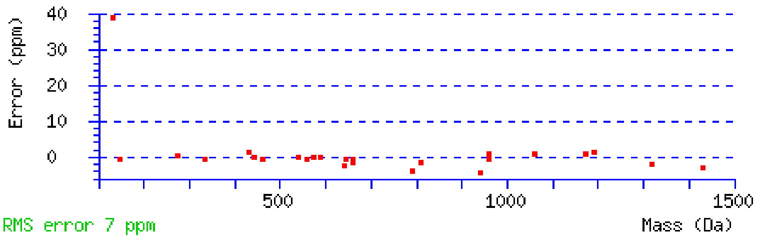

NCBI **BLAST** search of ADDKETCFAEEGKK
(Parameters: blastp, nr protein database, expect=20000, no filter, PAM30)
Other BLAST web gateways

All matches to this query

Score	Mr(calc):	Delta	Sequence
72.7	1617.6828	-0.0012	ADDKETCFAEEGKK

Mascot: http://www.matrixscience.com/

Mascot Search Results

Peptide View

MS/MS Fragmentation of **EFNAETFTFHADICTLSEKER**
Found in **sp|P02768|ALBU_HUMAN**, Serum albumin OS=Homo sapiens GN=ALB PE=1 SV=2

Match to Query 11891: 2535.123912 from(846.048580,3+) intensity(571849.5000) scans(13565) rtinseconds(2407) index(11620)
Title: 150801_TTSH_Patient_Plasma_45_Spectrum029046_scans__13565_RTINSECONDS=2407
Data file L:\\Ard_TTSH\\T1D\\T150801_TTSH_Patient_Plasma_45.mgf

Click mouse within plot area to zoom in by factor of two about that point
Or, Plot from 100 to 2000 Da Full range
Label all possible matches ○ Label matches used for scoring ⊙

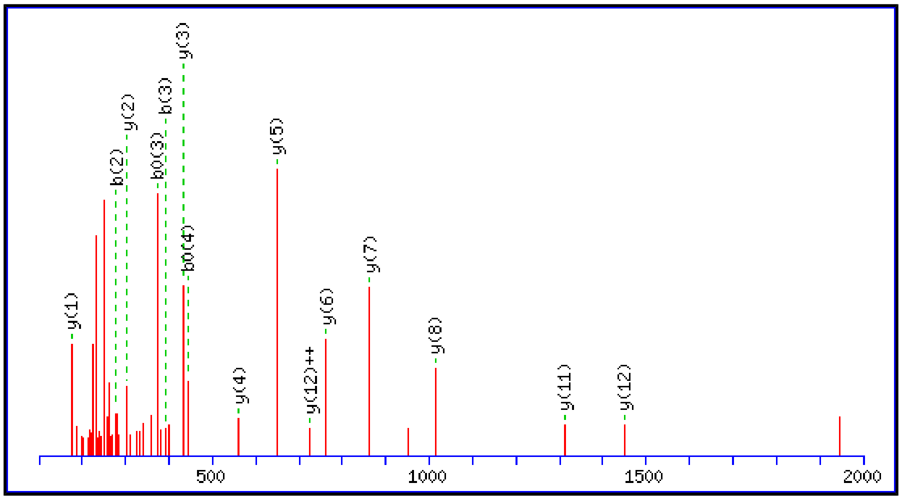

Monoisotopic mass of neutral peptide Mr(calc): 2535.1224
Variable modifications:
C14 : 4Trioxidation (CMWY)
Ions Score: 71 **Expect:** 3.9e-005
Matches : 15/234 fragment ions using 22 most intense peaks (help)

#	b	b^{++}	b*	b^{*++}	b^0	b^{0++}	Seq.	y	y^{++}	y*	y^{*++}	y^0	y^{0++}	#
1	130.0499	65.5286			112.0393	56.5233	E							21
2	**277.1183**	139.0628			259.1077	130.0575	F	2407.0871	1204.0472	2390.0605	1195.5339	2389.0765	1195.0419	20
3	**391.1612**	196.0842	374.1347	187.5710	373.1506	187.0790	N	2260.0187	1130.5130	2242.9921	1121.9997	2242.0081	1121.5077	19
4	462.1983	231.6028	445.1718	223.0895	444.1878	222.5975	A	2145.9757	1073.4915	2128.9492	1064.9782	2127.9652	1064.4862	18
5	591.2409	296.1241	574.2144	287.6108	573.2304	287.1188	E	2074.9386	1037.9729	2057.9121	1029.4597	2056.9280	1028.9677	17
6	692.2886	346.6479	675.2620	338.1347	674.2780	337.6427	T	1945.8960	973.4516	1928.8695	964.9384	1927.8855	964.4464	16
7	839.3570	420.1821	822.3305	411.6689	821.3464	411.1769	F	1844.8483	922.9278	1827.8218	914.4145	1826.8378	913.9225	15
8	940.4047	470.7060	923.3781	462.1927	922.3941	461.7007	T	1697.7799	849.3936	1680.7534	840.8803	1679.7694	840.3883	14
9	1087.4731	544.2402	1070.4466	535.7269	1069.4625	535.2349	F	1596.7322	798.8698	1579.7057	790.3565	1578.7217	789.8645	13
10	1224.5320	612.7696	1207.5055	604.2564	1206.5215	603.7644	H	***1449.6638***	725.3356	1432.6373	716.8223	1431.6533	716.3303	12
11	1295.5691	648.2882	1278.5426	639.7749	1277.5586	639.2829	A	***1312.6049***	656.8061	1295.5784	648.2928	1294.5944	647.8008	11
12	1410.5961	705.8017	1393.5695	697.2884	1392.5855	696.7964	D	1241.5678	621.2875	1224.5413	612.7743	1223.5572	612.2823	10
13	1523.6801	762.3437	1506.6536	753.8304	1505.6696	753.3384	I	1126.5409	563.7741	1109.5143	555.2608	1108.5303	554.7688	9
14	1674.6741	837.8407	1657.6475	829.3274	1656.6635	828.8354	C	***1013.4568***	507.2320	996.4303	498.7188	995.4462	498.2268	8
15	1775.7217	888.3645	1758.6952	879.8512	1757.7112	879.3592	T	***862.4629***	431.7351	845.4363	423.2218	844.4523	422.7298	7
16	1888.8058	944.9065	1871.7793	936.3933	1870.7952	935.9013	L	***761.4152***	381.2112	744.3886	372.6980	743.4046	372.2060	6
17	1975.8378	988.4226	1958.8113	979.9093	1957.8273	979.4173	S	***648.3311***	324.6692	631.3046	316.1559	630.3206	315.6639	5
18	2104.8804	1052.9439	2087.8539	1044.4306	2086.8699	1043.9386	E	***561.2991***	281.1532	544.2726	272.6399	543.2885	272.1479	4
19	2232.9754	1116.9913	2215.9488	1108.4781	2214.9648	1107.9861	K	***432.2565***	216.6319	415.2300	208.1186	414.2459	207.6266	3

**20**	2362.0180	1181.5126	2344.9914	1172.9994	2344.0074	1172.5073	**E**	***304.1615***	152.5844	287.1350	144.0711	286.1510	143.5791	**2**
**21**							**R**	***175.1190***	88.0631	158.0924	79.5498			**1**

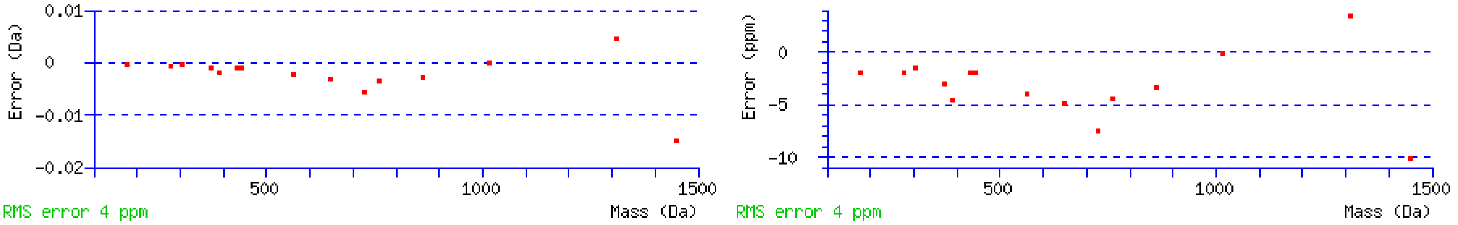

NCBI **BLAST** search of EFNAETFTFHADICTLSEKER
(Parameters: blastp, nr protein database, expect=20000, no filter, PAM30)
Other BLAST web gateways

All matches to this query

Score	Mr(calc):	Delta	Sequence
70.6	2535.1224	0.0016	EFNAETFTFHADICTLSEKER

Mascot: http://www.matrixscience.com/

Mascot Search Results

Peptide View

MS/MS Fragmentation of **RPCFSALEVDETYVPK**
Found in **sp|P02768|ALBU_HUMAN**, Serum albumin OS=Homo sapiens GN=ALB PE=1 SV=2

Match to Query 10838: 1900.888152 from(634.636660,3+) intensity(1511580.2500) scans(14740) rtinseconds(2729) index(12399)
Title: 150808_TTSH_Patient_Plasma_20_Spectrum028726_scans__14740_RTINSECONDS=2729
Data file L:\\Ard_TTSH\\T1D\\T150808_TTSH_Patient_Plasma_20.mgf

Click mouse within plot area to zoom in by factor of two about that point
Or, Plot from 100 to 1100 Da Full range
Label all possible matches ○ Label matches used for scoring ⊙

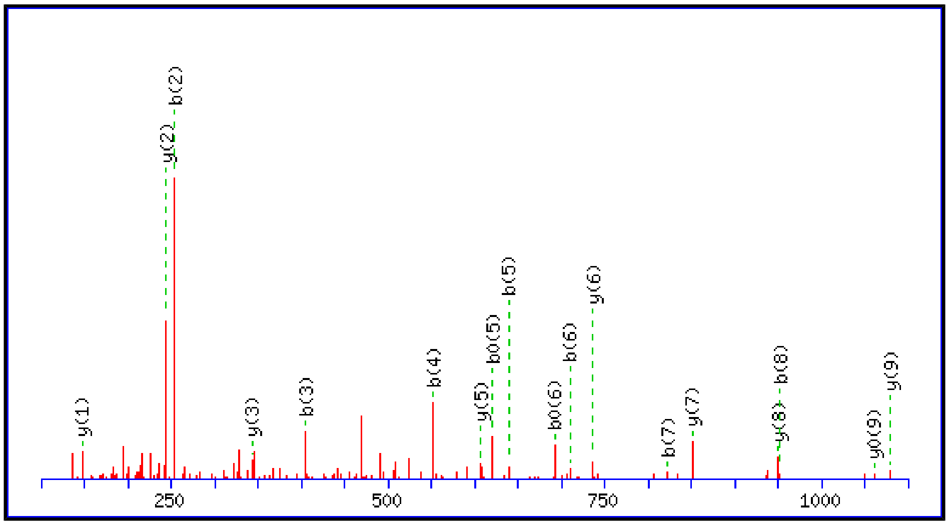

Monoisotopic mass of neutral peptide Mr(calc): 1900.8877
Variable modifications:
C3 : 4Trioxidation (CMWY)
Ions Score: 70 **Expect:** 3.8e-005
Matches : 18/164 fragment ions using 28 most intense peaks (help)

#	b	b^{++}	b*	b^{*++}	b^{0}	b^{0++}	Seq.	y	y^{++}	y*	y^{*++}	y^{0}	y^{0++}	#
1	157.1084	79.0578	140.0818	70.5446			R							16
2	***254.1612***	127.5842	237.1346	119.0709			P	1745.7938	873.4006	1728.7673	864.8873	1727.7833	864.3953	15
3	***405.1551***	203.0812	388.1285	194.5679			C	1648.7411	824.8742	1631.7145	816.3609	1630.7305	815.8689	14
4	***552.2235***	276.6154	535.1969	268.1021			F	1497.7472	749.3772	1480.7206	740.8639	1479.7366	740.3719	13
5	***639.2555***	320.1314	622.2290	311.6181	621.2450	311.1261	S	1350.6787	675.8430	1333.6522	667.3297	1332.6682	666.8377	12
6	***710.2926***	355.6500	693.2661	347.1367	692.2821	346.6447	A	1263.6467	632.3270	1246.6202	623.8137	1245.6361	623.3217	11
7	***823.3767***	412.1920	806.3502	403.6787	805.3661	403.1867	L	1192.6096	596.8084	1175.5830	588.2952	1174.5990	587.8032	10
8	***952.4193***	476.7133	935.3927	468.2000	934.4087	467.7080	E	***1079.5255***	540.2664	1062.4990	531.7531	1061.5150	531.2611	9
9	1051.4877	526.2475	1034.4612	517.7342	1033.4771	517.2422	V	***950.4829***	475.7451	933.4564	467.2318	932.4724	466.7398	8
10	1166.5147	583.7610	1149.4881	575.2477	1148.5041	574.7557	D	***851.4145***	426.2109	834.3880	417.6976	833.4040	417.2056	7
11	1295.5572	648.2823	1278.5307	639.7690	1277.5467	639.2770	E	***736.3876***	368.6974	719.3610	360.1842	718.3770	359.6921	6
12	1396.6049	698.8061	1379.5784	690.2928	1378.5944	689.8008	T	***607.3450***	304.1761	590.3184	295.6629	589.3344	295.1709	5
13	1559.6683	780.3378	1542.6417	771.8245	1541.6577	771.3325	Y	506.2973	253.6523	489.2708	245.1390			4
14	1658.7367	829.8720	1641.7101	821.3587	1640.7261	820.8667	V	***343.2340***	172.1206	326.2074	163.6074			3
15	1755.7894	878.3984	1738.7629	869.8851	1737.7789	869.3931	P	***244.1656***	122.5864	227.1390	114.0731			2
16							K	***147.1128***	74.0600	130.0863	65.5468			1

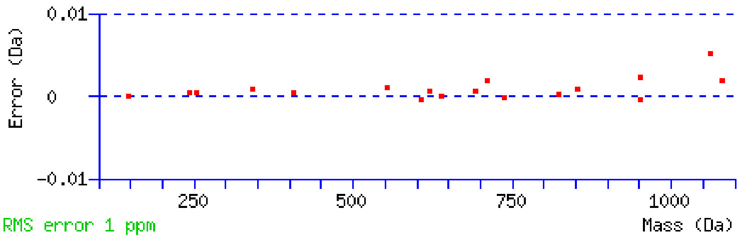

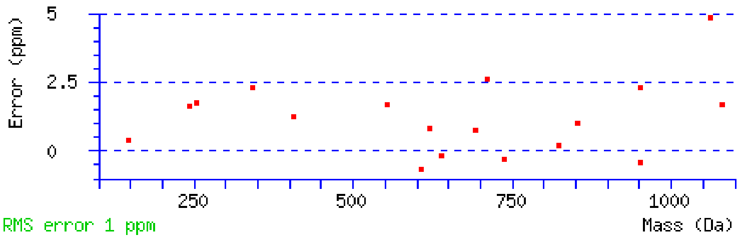

NCBI **BLAST** search of RPCFSALEVDETYVPK
(Parameters: blastp, nr protein database, expect=20000, no filter, PAM30)
Other BLAST web gateways

All matches to this query

Score	Mr(calc):	Delta	Sequence
70.0	1900.8877	0.0005	RPCFSALEVDETYVPK
0.3	1900.8877	0.0005	RPCFSALEVDETYVPK

Mascot Search Results

Peptide View

MS/MS Fragmentation of **SHCIAEVENDEMPADLPSLAADFVESK**
Found in **sp|P02768|ALBU_HUMAN**, Serum albumin OS=Homo sapiens GN=ALB PE=1 SV=2

Match to Query 13865: 3012.269862 from(1005.097230,3+) intensity(981595.7500) scans(16995) rtinseconds(3049) index(14532)
Title: 150808_TTSH_Patient_Plasma_63_Spectrum031512_scans__16995_RTINSECONDS=3049
Data file L:\\Ard_TTSH\\T1D\\T150808_TTSH_Patient_Plasma_63.mgf

Click mouse within plot area to zoom in by factor of two about that point
Or, Plot from 200 to 3000 Da Full range
Label all possible matches ○ Label matches used for scoring ⊙

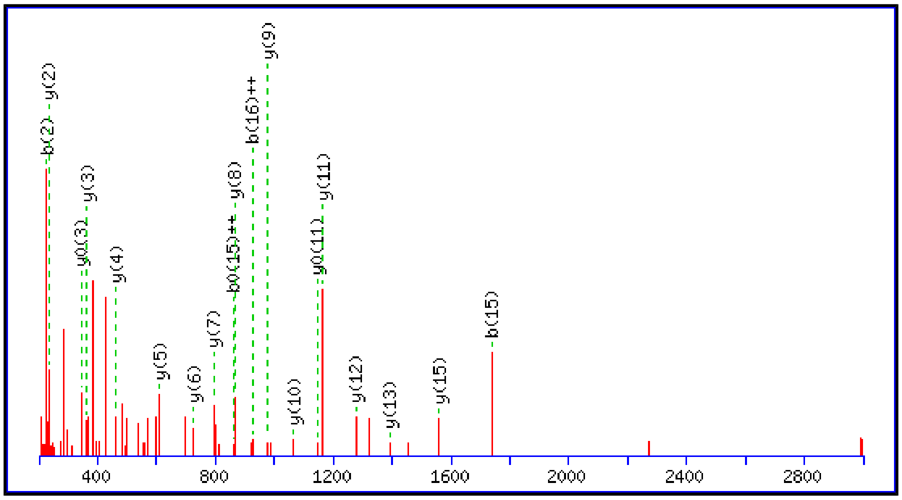

Monoisotopic mass of neutral peptide Mr(calc): 3012.2852
Variable modifications:
C3 : 4Trioxidation (CMWY)
M12 : 4Trioxidation (CMWY)
Ions Score: 67 **Expect:** 6.1e-005
Matches : 19/294 fragment ions using 42 most intense peaks (help)

#	b	b^{++}	b*	b^{*++}	b^0	b^{0++}	Seq.	y	y^{++}	y*	y^{*++}	y^0	y^{0++}	#
1	88.0393	44.5233			70.0287	35.5180	S							27
2	225.0982	113.0527			207.0877	104.0475	H	2926.2605	1463.6339	2909.2339	1455.1206	2908.2499	1454.6286	26
3	376.0921	188.5497			358.0816	179.5444	C	2789.2016	1395.1044	2772.1750	1386.5911	2771.1910	1386.0991	25
4	489.1762	245.0917			471.1656	236.0865	I	2638.2076	1319.6075	2621.1811	1311.0942	2620.1971	1310.6022	24
5	560.2133	280.6103			542.2028	271.6050	A	2525.1236	1263.0654	2508.0970	1254.5521	2507.1130	1254.0601	23
6	689.2559	345.1316			671.2454	336.1263	E	2454.0865	1227.5469	2437.0599	1219.0336	2436.0759	1218.5416	22
7	788.3243	394.6658			770.3138	385.6605	V	2325.0439	1163.0256	2308.0173	1154.5123	2307.0333	1154.0203	21
8	917.3669	459.1871			899.3564	450.1818	E	2225.9754	1113.4914	2208.9489	1104.9781	2207.9649	1104.4861	20
9	1031.4099	516.2086	1014.3833	507.6953	1013.3993	507.2033	N	2096.9329	1048.9701	2079.9063	1040.4568	2078.9223	1039.9648	19
10	1146.4368	573.7220	1129.4102	565.2088	1128.4262	564.7168	D	1982.8899	991.9486	1965.8634	983.4353	1964.8794	982.9433	18
11	1275.4794	638.2433	1258.4528	629.7301	1257.4688	629.2380	E	1867.8630	934.4351	1850.8364	925.9219	1849.8524	925.4298	17
12	1454.5046	727.7559	1437.4781	719.2427	1436.4941	718.7507	M	1738.8204	869.9138	1721.7938	861.4006	1720.8098	860.9086	16
13	1551.5574	776.2823	1534.5308	767.7691	1533.5468	767.2770	P	*1559.7952*	780.4012	1542.7686	771.8879	1541.7846	771.3959	15
14	1622.5945	811.8009	1605.5679	803.2876	1604.5839	802.7956	A	1462.7424	731.8748	1445.7159	723.3616	1444.7318	722.8696	14
15	1737.6214	869.3144	1720.5949	860.8011	1719.6109	860.3091	D	*1391.7053*	696.3563	1374.6787	687.8430	1373.6947	687.3510	13
16	1850.7055	925.8564	1833.6790	917.3431	1832.6949	916.8511	L	*1276.6783*	638.8428	1259.6518	630.3295	1258.6678	629.8375	12
17	1947.7583	974.3828	1930.7317	965.8695	1929.7477	965.3775	P	*1163.5943*	582.3008	1146.5677	573.7875	1145.5837	573.2955	11
18	2034.7903	1017.8988	2017.7637	1009.3855	2016.7797	1008.8935	S	*1066.5415*	533.7744	1049.5150	525.2611	1048.5310	524.7691	10
19	2147.8744	1074.4408	2130.8478	1065.9275	2129.8638	1065.4355	L	*979.5095*	490.2584	962.4829	481.7451	961.4989	481.2531	9

**20**	2218.9115	1109.9594	2201.8849	1101.4461	2200.9009	1100.9541	**A**	***866.4254***	433.7164	849.3989	425.2031	848.4149	424.7111	**8**
**21**	2289.9486	1145.4779	2272.9220	1136.9647	2271.9380	1136.4726	**A**	***795.3883***	398.1978	778.3618	389.6845	777.3777	389.1925	**7**
**22**	2404.9755	1202.9914	2387.9490	1194.4781	2386.9650	1193.9861	**D**	***724.3512***	362.6792	707.3246	354.1660	706.3406	353.6740	**6**
**23**	2552.0439	1276.5256	2535.0174	1268.0123	2534.0334	1267.5203	**F**	***609.3243***	305.1658	592.2977	296.6525	591.3137	296.1605	**5**
**24**	2651.1124	1326.0598	2634.0858	1317.5465	2633.1018	1317.0545	**V**	***462.2558***	231.6316	445.2293	223.1183	444.2453	222.6263	**4**
**25**	2780.1549	1390.5811	2763.1284	1382.0678	2762.1444	1381.5758	**E**	***363.1874***	182.0974	346.1609	173.5841	345.1769	173.0921	**3**
**26**	2867.1870	1434.0971	2850.1604	1425.5839	2849.1764	1425.0918	**S**	***234.1448***	117.5761	217.1183	109.0628	216.1343	108.5708	**2**
**27**							**K**	147.1128	74.0600	130.0863	65.5468			**1**

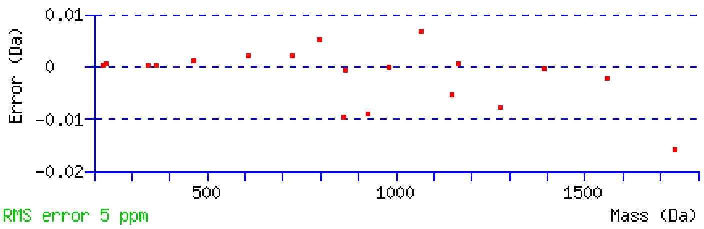

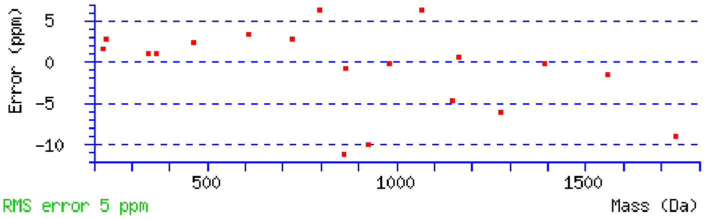

NCBI **BLAST** search of SHCIAEVENDEMPADLPSLAADFVESK
(Parameters: blastp, nr protein database, expect=20000, no filter, PAM30)
Other BLAST web gateways

All matches to this query

Score	Mr(calc):	Delta	Sequence
67.5	3012.2852	-0.0153	SHCIAEVENDEMPADLPSLAADFVESK

Mascot: http://www.matrixscience.com/

Mascot Search Results

Peptide View

MS/MS Fragmentation of **LKECCEKPLLEK**
Found in **sp|P02768|ALBU_HUMAN**, Serum albumin OS=Homo sapiens GN=ALB PE=1 SV=2

Match to Query 5998: 1527.714448 from(764.864500,2+) intensity(815917.1875) scans(4976) rtinseconds(906) index(4055)
Title: 150801_TTSH_Patient_Plasma_26_Spectrum021108_scans__4976_RTINSECONDS=906
Data file L:\\Ard_TTSH\\T1D\\T150801_TTSH_Patient_Plasma_26.mgf

Click mouse within plot area to zoom in by factor of two about that point
Or, Plot from 100 to 1050 Da Full range
Label all possible matches ○ Label matches used for scoring ⦿

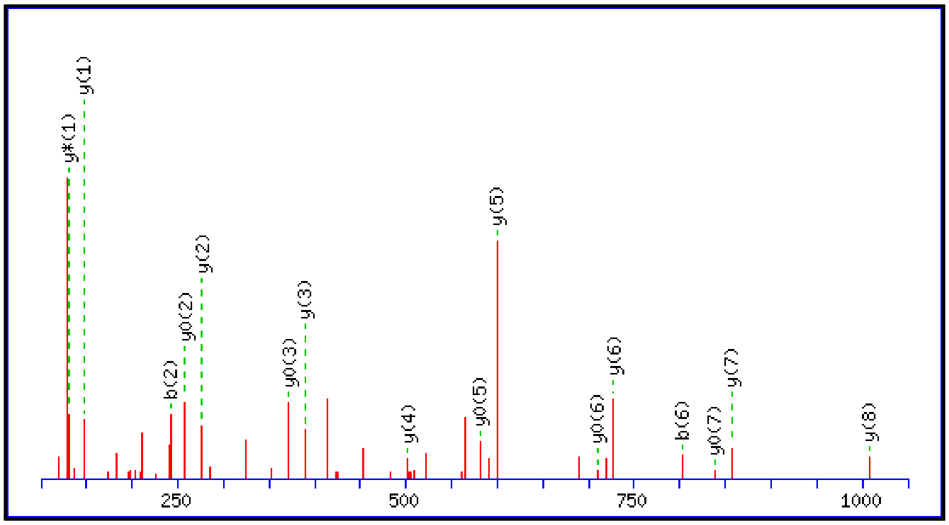

Monoisotopic mass of neutral peptide Mr(calc): 1527.7160
Variable modifications:
C4 : 4Trioxidation (CMWY)
C5 : 4Trioxidation (CMWY)
Ions Score: 60 **Expect:** 0.00028
Matches : 17/124 fragment ions using 23 most intense peaks (help)

#	b	b^{++}	b*	b^{*++}	b^{0}	b^{0++}	Seq.	y	y^{++}	y*	y^{*++}	y^{0}	y^{0++}	#
1	114.0913	57.5493					L							12
2	**242.1863**	121.5968	225.1598	113.0835			K	1415.6393	708.3233	1398.6127	699.8100	1397.6287	699.3180	11
3	**371.2289**	186.1181	354.2023	177.6048	353.2183	177.1128	E	1287.5443	644.2758	1270.5177	635.7625	1269.5337	635.2705	10
4	522.2228	261.6151	505.1963	253.1018	504.2123	252.6098	C	1158.5017	579.7545	1141.4752	571.2412	1140.4911	570.7492	9
5	673.2168	337.1120	656.1902	328.5987	655.2062	328.1067	C	***1007.5078***	504.2575	990.4812	495.7443	989.4972	495.2522	8
6	**802.2593**	401.6333	785.2328	393.1200	784.2488	392.6280	E	***856.5138***	428.7606	839.4873	420.2473	838.5033	419.7553	7
7	930.3543	465.6808	913.3278	457.1675	912.3437	456.6755	K	***727.4713***	364.2393	710.4447	355.7260	709.4607	355.2340	6
8	1027.4071	514.2072	1010.3805	505.6939	1009.3965	505.2019	P	***599.3763***	300.1918	582.3497	291.6785	581.3657	291.1865	5
9	1140.4911	570.7492	1123.4646	562.2359	1122.4806	561.7439	L	***502.3235***	251.6654	485.2970	243.1521	484.3130	242.6601	4
10	1253.5752	627.2912	1236.5487	618.7780	1235.5646	618.2860	L	***389.2395***	195.1234	372.2129	186.6101	371.2289	186.1181	3
11	1382.6178	691.8125	1365.5912	683.2993	1364.6072	682.8073	E	***276.1554***	138.5813	259.1288	130.0681	258.1448	129.5761	2
12							K	***147.1128***	74.0600	130.0863	65.5468			1

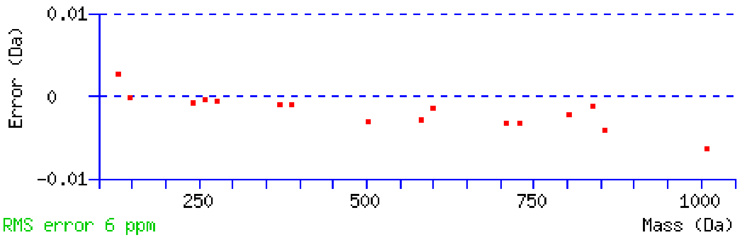

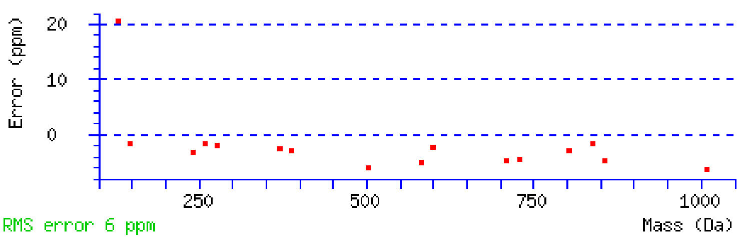

NCBI **BLAST** search of LKECCEKPLLEK
(Parameters: blastp, nr protein database, expect=20000, no filter, PAM30)
Other BLAST web gateways

All matches to this query

Score	Mr(calc):	Delta	Sequence
60.2	1527.7160	-0.0015	LKECCEKPLLEK
7.5	1525.7049	2.0096	DQDDHIDWLLEK
5.7	1526.7181	0.9964	KNLSRMQTQMDK
4.3	1526.7109	1.0036	CKVPGCFLQDLEK
1.5	1527.7239	-0.0094	KIENCNYAVELGK
1.5	1527.7239	-0.0094	KIENCNYAVELGK
1.5	1527.7239	-0.0094	KLENCNYAVELGK
1.5	1527.7239	-0.0094	KLENCNYAVELGK
1.1	1525.6959	2.0186	KELHFCCLHWP
0.9	1526.7181	0.9964	KNLSRMQTQMDK

Mascot Search Results

Peptide View

MS/MS Fragmentation of **FKDLGEENFKALVLIAFAQYLQQCPFEDHVK**
Found in **sp|P02768|ALBU_HUMAN**, Serum albumin OS=Homo sapiens GN=ALB PE=1 SV=2

Match to Query 15398: 3735.797970 from(748.166870,5+) intensity(3512849.7500) scans(17266) rtinseconds(3042) index(14748)
Title: 150801_TTSH_Patient_Plasma_45_Spectrum032174_scans__17266_RTINSECONDS=3042
Data file L:\\Ard_TTSH\\T1D\\T150801_TTSH_Patient_Plasma_45.mgf

Click mouse within plot area to zoom in by factor of two about that point
Or, Plot from 200 to 3800 Da Full range
Label all possible matches ○ Label matches used for scoring ⦿

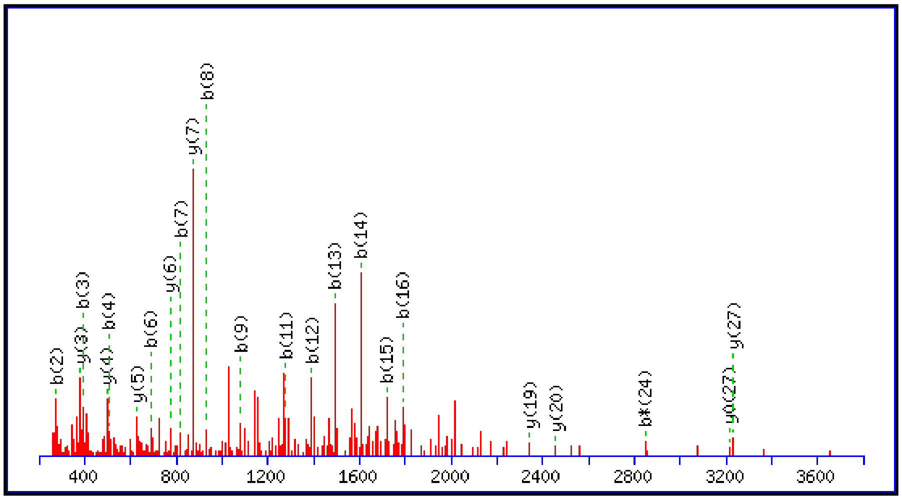

Monoisotopic mass of neutral peptide Mr(calc): 3735.8130
Variable modifications:
Y20 : 4Trioxidation (CMWY)
C24 : 4Trioxidation (CMWY)
Ions Score: 59 **Expect:** 0.00072
Matches : 23/348 fragment ions using 52 most intense peaks (help)

#	b	b++	b*	b*++	b0	b0++	Seq.	y	y++	y*	y*++	y0	y0++	#
1	148.0757	74.5415					F							31
2	***276.1707***	138.5890	259.1441	130.0757			K	3589.7519	1795.3796	3572.7254	1786.8663	3571.7414	1786.3743	30
3	***391.1976***	196.1024	374.1710	187.5892	373.1870	187.0972	D	3461.6570	1731.3321	3444.6304	1722.8188	3443.6464	1722.3268	29
4	***504.2817***	252.6445	487.2551	244.1312	486.2711	243.6392	L	3346.6300	1673.8186	3329.6035	1665.3054	3328.6195	1664.8134	28
5	561.3031	281.1552	544.2766	272.6419	543.2926	272.1499	G	***3233.5460***	1617.2766	3216.5194	1608.7633	3215.5354	1608.2713	27
6	***690.3457***	345.6765	673.3192	337.1632	672.3352	336.6712	E	3176.5245	1588.7659	3159.4979	1580.2526	3158.5139	1579.7606	26
7	***819.3883***	410.1978	802.3618	401.6845	801.3777	401.1925	E	3047.4819	1524.2446	3030.4554	1515.7313	3029.4713	1515.2393	25
8	***933.4312***	467.2193	916.4047	458.7060	915.4207	458.2140	N	2918.4393	1459.7233	2901.4128	1451.2100	2900.4287	1450.7180	24
9	***1080.4997***	540.7535	1063.4731	532.2402	1062.4891	531.7482	F	2804.3964	1402.7018	2787.3698	1394.1886	2786.3858	1393.6965	23
10	1208.5946	604.8009	1191.5681	596.2877	1190.5840	595.7957	K	2657.3280	1329.1676	2640.3014	1320.6543	2639.3174	1320.1623	22
11	***1279.6317***	640.3195	1262.6052	631.8062	1261.6212	631.3142	A	2529.2330	1265.1201	2512.2065	1256.6069	2511.2224	1256.1149	21
12	***1392.7158***	696.8615	1375.6892	688.3483	1374.7052	687.8563	L	***2458.1959***	1229.6016	2441.1693	1221.0883	2440.1853	1220.5963	20
13	***1491.7842***	746.3957	1474.7577	737.8825	1473.7736	737.3905	V	***2345.1118***	1173.0596	2328.0853	1164.5463	2327.1013	1164.0543	19
14	***1604.8683***	802.9378	1587.8417	794.4245	1586.8577	793.9325	L	2246.0434	1123.5253	2229.0169	1115.0121	2228.0328	1114.5201	18
15	***1717.9523***	859.4798	1700.9258	850.9665	1699.9418	850.4745	I	2132.9593	1066.9833	2115.9328	1058.4700	2114.9488	1057.9780	17
16	***1788.9894***	894.9984	1771.9629	886.4851	1770.9789	885.9931	A	2019.8753	1010.4413	2002.8487	1001.9280	2001.8647	1001.4360	16
17	1936.0579	968.5326	1919.0313	960.0193	1918.0473	959.5273	F	1948.8382	974.9227	1931.8116	966.4094	1930.8276	965.9174	15
18	2007.0950	1004.0511	1990.0684	995.5379	1989.0844	995.0458	A	1801.7698	901.3885	1784.7432	892.8752	1783.7592	892.3832	14
19	2135.1536	1068.0804	2118.1270	1059.5671	2117.1430	1059.0751	Q	1730.7326	865.8700	1713.7061	857.3567	1712.7221	856.8647	13

**20**	2346.2016	1173.6045	2329.1751	1165.0912	2328.1911	1164.5992	**Y**	1602.6741	801.8407	1585.6475	793.3274	1584.6635	792.8354	**12**
**21**	2459.2857	1230.1465	2442.2591	1221.6332	2441.2751	1221.1412	**L**	1391.6260	696.3166	1374.5994	687.8034	1373.6154	687.3114	**11**
**22**	2587.3443	1294.1758	2570.3177	1285.6625	2569.3337	1285.1705	**Q**	1278.5419	639.7746	1261.5154	631.2613	1260.5314	630.7693	**10**
**23**	2715.4028	1358.2051	2698.3763	1349.6918	2697.3923	1349.1998	**Q**	1150.4834	575.7453	1133.4568	567.2320	1132.4728	566.7400	**9**
**24**	2866.3968	1433.7020	2849.3702	1425.1888	2848.3862	1424.6967	**C**	1022.4248	511.7160	1005.3982	503.2027	1004.4142	502.7107	**8**
**25**	2963.4495	1482.2284	2946.4230	1473.7151	2945.4390	1473.2231	**P**	***871.4308***	436.2191	854.4043	427.7058	853.4203	427.2138	**7**
**26**	3110.5180	1555.7626	3093.4914	1547.2493	3092.5074	1546.7573	**F**	***774.3781***	387.6927	757.3515	379.1794	756.3675	378.6874	**6**
**27**	3239.5605	1620.2839	3222.5340	1611.7706	3221.5500	1611.2786	**E**	***627.3097***	314.1585	610.2831	305.6452	609.2991	305.1532	**5**
**28**	3354.5875	1677.7974	3337.5609	1669.2841	3336.5769	1668.7921	**D**	***498.2671***	249.6372	481.2405	241.1239	480.2565	240.6319	**4**
**29**	3491.6464	1746.3268	3474.6199	1737.8136	3473.6358	1737.3216	**H**	***383.2401***	192.1237	366.2136	183.6104			**3**
**30**	3590.7148	1795.8610	3573.6883	1787.3478	3572.7043	1786.8558	**V**	246.1812	123.5942	229.1547	115.0810			**2**
**31**							**K**	147.1128	74.0600	130.0863	65.5468			**1**

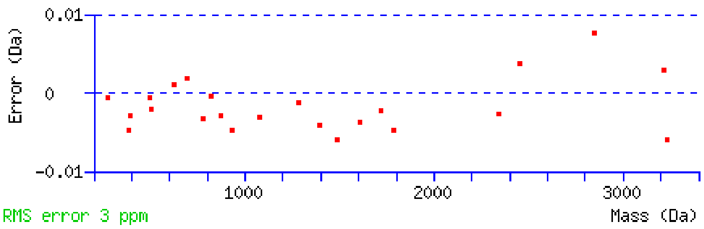

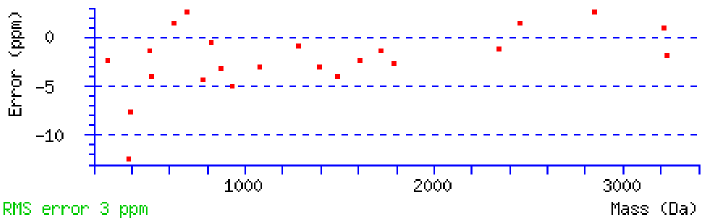

NCBI **BLAST** search of FKDLGEENFKALVLIAFAQYLQQCPFEDHVK
(Parameters: blastp, nr protein database, expect=20000, no filter, PAM30)
Other BLAST web gateways

All matches to this query

Score	Mr(calc):	Delta	Sequence
59.2	3735.8130	-0.0151	FKDLGEENFKALVLIAFAQYLQQCPFEDHVK

Mascot: http://www.matrixscience.com/

Mascot Search Results

Peptide View

MS/MS Fragmentation of **SHCIAEVENDEMPADLPSLAADFVESK**
Found in **sp|P02768|ALBU_HUMAN**, Serum albumin OS=Homo sapiens GN=ALB PE=1 SV=2

Match to Query 7064: 3012.265096 from(754.073550,4+) intensity(318314.5938) scans(11716) rtinseconds(3103) index(6563)
Title: 150808_TTSH_Patient_Plasma_04_Spectrum015407_scans__11716_RTINSECONDS=3103
Data file L:\\Ard_TTSH\\T1D\\T150808_TTSH_Patient_Plasma_04.mgf

Click mouse within plot area to zoom in by factor of two about that point
Or, Plot from 200 to 1800 Da Full range
Label all possible matches ○ Label matches used for scoring ⊙

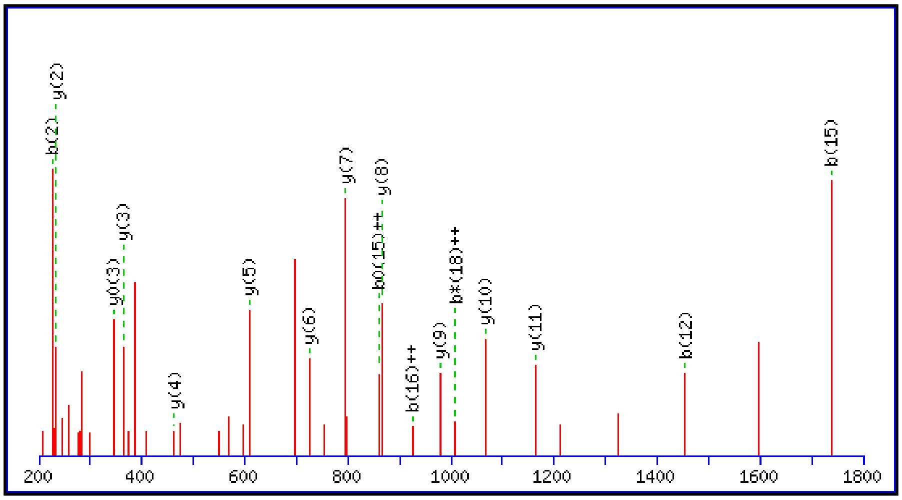

Monoisotopic mass of neutral peptide Mr(calc): 3012.2852
Variable modifications:
C3 : 4Trioxidation (CMWY)
M12 : 4Trioxidation (CMWY)
Ions Score: 59 **Expect:** 0.00043
Matches : 17/294 fragment ions using 28 most intense peaks (help)

#	b	b++	b*	b*++	b0	b0++	Seq.	y	y++	y*	y*++	y0	y0++	#
1	88.0393	44.5233			70.0287	35.5180	S							27
2	**225.0982**	113.0527			207.0877	104.0475	H	2926.2605	1463.6339	2909.2339	1455.1206	2908.2499	1454.6286	26
3	376.0921	188.5497			358.0816	179.5444	C	2789.2016	1395.1044	2772.1750	1386.5911	2771.1910	1386.0991	25
4	489.1762	245.0917			471.1656	236.0865	I	2638.2076	1319.6075	2621.1811	1311.0942	2620.1971	1310.6022	24
5	560.2133	280.6103			542.2028	271.6050	A	2525.1236	1263.0654	2508.0970	1254.5521	2507.1130	1254.0601	23
6	689.2559	345.1316			671.2454	336.1263	E	2454.0865	1227.5469	2437.0599	1219.0336	2436.0759	1218.5416	22
7	788.3243	394.6658			770.3138	385.6605	V	2325.0439	1163.0256	2308.0173	1154.5123	2307.0333	1154.0203	21
8	917.3669	459.1871			899.3564	450.1818	E	2225.9754	1113.4914	2208.9489	1104.9781	2207.9649	1104.4861	20
9	1031.4099	516.2086	1014.3833	507.6953	1013.3993	507.2033	N	2096.9329	1048.9701	2079.9063	1040.4568	2078.9223	1039.9648	19
10	1146.4368	573.7220	1129.4102	565.2088	1128.4262	564.7168	D	1982.8899	991.9486	1965.8634	983.4353	1964.8794	982.9433	18
11	1275.4794	638.2433	1258.4528	629.7301	1257.4688	629.2380	E	1867.8630	934.4351	1850.8364	925.9219	1849.8524	925.4298	17
12	**1454.5046**	727.7559	1437.4781	719.2427	1436.4941	718.7507	M	1738.8204	869.9138	1721.7938	861.4006	1720.8098	860.9086	16
13	1551.5574	776.2823	1534.5308	767.7691	1533.5468	767.2770	P	1559.7952	780.4012	1542.7686	771.8879	1541.7846	771.3959	15
14	1622.5945	811.8009	1605.5679	803.2876	1604.5839	802.7956	A	1462.7424	731.8748	1445.7159	723.3616	1444.7318	722.8696	14
15	**1737.6214**	869.3144	1720.5949	860.8011	1719.6109	860.3091	D	1391.7053	696.3563	1374.6787	687.8430	1373.6947	687.3510	13
16	1850.7055	**925.8564**	1833.6790	917.3431	1832.6949	916.8511	L	1276.6783	638.8428	1259.6518	630.3295	1258.6678	629.8375	12
17	1947.7583	974.3828	1930.7317	965.8695	1929.7477	965.3775	P	***1163.5943***	582.3008	1146.5677	573.7875	1145.5837	573.2955	11
18	2034.7903	1017.8988	2017.7637	1009.3855	2016.7797	1008.8935	S	***1066.5415***	533.7744	1049.5150	525.2611	1048.5310	524.7691	10
19	2147.8744	1074.4408	2130.8478	1065.9275	2129.8638	1065.4355	L	***979.5095***	490.2584	962.4829	481.7451	961.4989	481.2531	9

**20**	2218.9115	1109.9594	2201.8849	1101.4461	2200.9009	1100.9541	**A**	***866.4254***	433.7164	849.3989	425.2031	848.4149	424.7111	**8**
**21**	2289.9486	1145.4779	2272.9220	1136.9647	2271.9380	1136.4726	**A**	***795.3883***	398.1978	778.3618	389.6845	777.3777	389.1925	**7**
**22**	2404.9755	1202.9914	2387.9490	1194.4781	2386.9650	1193.9861	**D**	***724.3512***	362.6792	707.3246	354.1660	706.3406	353.6740	**6**
**23**	2552.0439	1276.5256	2535.0174	1268.0123	2534.0334	1267.5203	**F**	***609.3243***	305.1658	592.2977	296.6525	591.3137	296.1605	**5**
**24**	2651.1124	1326.0598	2634.0858	1317.5465	2633.1018	1317.0545	**V**	***462.2558***	231.6316	445.2293	223.1183	444.2453	222.6263	**4**
**25**	2780.1549	1390.5811	2763.1284	1382.0678	2762.1444	1381.5758	**E**	***363.1874***	182.0974	346.1609	173.5841	345.1769	173.0921	**3**
**26**	2867.1870	1434.0971	2850.1604	1425.5839	2849.1764	1425.0918	**S**	***234.1448***	117.5761	217.1183	109.0628	216.1343	108.5708	**2**
**27**							**K**	147.1128	74.0600	130.0863	65.5468			**1**

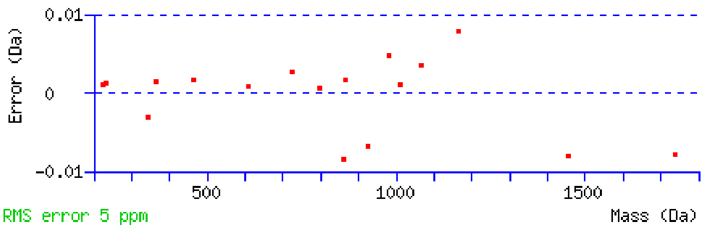

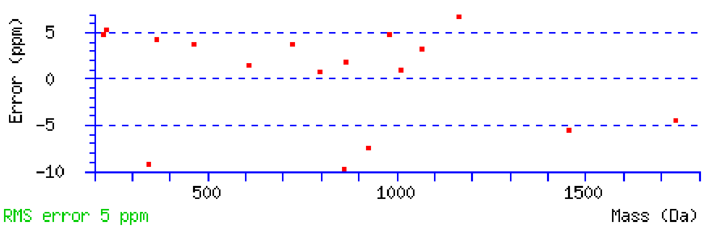

NCBI **BLAST** search of SHCIAEVENDEMPADLPSLAADFVESK
(Parameters: blastp, nr protein database, expect=20000, no filter, PAM30)
Other BLAST web gateways

All matches to this query

Score	Mr(calc):	Delta	Sequence
58.7	3012.2852	-0.0201	SHCIAEVENDEMPADLPSLAADFVESK

Mascot: http://www.matrixscience.com/

Mascot Search Results

Peptide View

MS/MS Fragmentation of **LVRPEVDVMCTAFHDNEETFLKK**
Found in **sp|P02768|ALBU_HUMAN**, Serum albumin OS=Homo sapiens GN=ALB PE=1 SV=2

Match to Query 14420: 2816.299670 from(564.267210,5+) intensity(748852.2500) scans(15740) rtinseconds(2809) index(13638)
Title: 150818_TTSH_Patient_Plasma_58_Spectrum031088_scans__15740_RTINSECONDS=2809
Data file L:\\Ard_TTSH\\T1D\\T150818_TTSH_Patient_Plasma_58.mgf

Click mouse within plot area to zoom in by factor of two about that point
Or, Plot from 0 to 2200 Da Full range
Label all possible matches ○ Label matches used for scoring ⊙

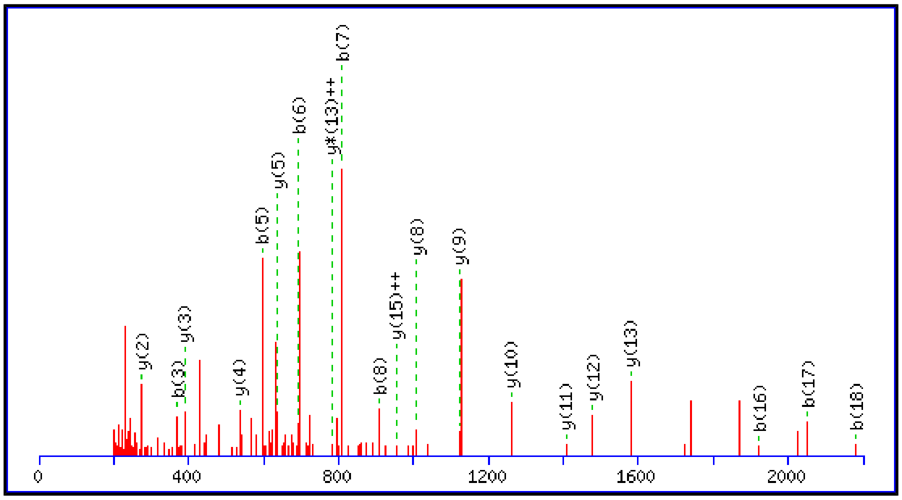

Monoisotopic mass of neutral peptide Mr(calc): 2816.2997
Variable modifications:
M9 : 4Trioxidation (CMWY)
C10 : 4Trioxidation (CMWY)
Ions Score: 56 **Expect:** 0.0012
Matches : 20/244 fragment ions using 48 most intense peaks (help)

#	b	b^{++}	b*	b^{*++}	b^{0}	b^{0++}	Seq.	y	y^{++}	y*	y^{*++}	y^{0}	y^{0++}	#
1	114.0913	57.5493					L							23
2	213.1598	107.0835					V	2704.2229	1352.6151	2687.1964	1344.1018	2686.2123	1343.6098	22
3	***369.2609***	185.1341	352.2343	176.6208			R	2605.1545	1303.0809	2588.1279	1294.5676	2587.1439	1294.0756	21
4	466.3136	233.6605	449.2871	225.1472			P	2449.0534	1225.0303	2432.0268	1216.5171	2431.0428	1216.0250	20
5	***595.3562***	298.1817	578.3297	289.6685	577.3457	289.1765	E	2352.0006	1176.5039	2334.9741	1167.9907	2333.9901	1167.4987	19
6	***694.4246***	347.7160	677.3981	339.2027	676.4141	338.7107	V	2222.9580	1111.9827	2205.9315	1103.4694	2204.9475	1102.9774	18
7	***809.4516***	405.2294	792.4250	396.7162	791.4410	396.2241	D	2123.8896	1062.4484	2106.8631	1053.9352	2105.8790	1053.4432	17
8	***908.5200***	454.7636	891.4934	446.2504	890.5094	445.7584	V	2008.8627	1004.9350	1991.8361	996.4217	1990.8521	995.9297	16
9	1087.5452	544.2762	1070.5187	535.7630	1069.5347	535.2710	M	1909.7943	955.4008	1892.7677	946.8875	1891.7837	946.3955	15
10	1238.5392	619.7732	1221.5126	611.2599	1220.5286	610.7679	C	1730.7690	865.8882	1713.7425	857.3749	1712.7585	856.8829	14
11	1339.5868	670.2971	1322.5603	661.7838	1321.5763	661.2918	T	***1579.7751***	790.3912	1562.7486	781.8779	1561.7645	781.3859	13
12	1410.6239	705.8156	1393.5974	697.3023	1392.6134	696.8103	A	***1478.7274***	739.8673	1461.7009	731.3541	1460.7169	730.8621	12
13	1557.6924	779.3498	1540.6658	770.8365	1539.6818	770.3445	F	***1407.6903***	704.3488	1390.6638	695.8355	1389.6797	695.3435	11
14	1694.7513	847.8793	1677.7247	839.3660	1676.7407	838.8740	H	***1260.6219***	630.8146	1243.5953	622.3013	1242.6113	621.8093	10
15	1809.7782	905.3927	1792.7517	896.8795	1791.7676	896.3875	D	***1123.5630***	562.2851	1106.5364	553.7719	1105.5524	553.2798	9
16	***1923.8211***	962.4142	1906.7946	953.9009	1905.8106	953.4089	N	***1008.5360***	504.7717	991.5095	496.2584	990.5255	495.7664	8
17	***2052.8637***	1026.9355	2035.8372	1018.4222	2034.8532	1017.9302	E	894.4931	447.7502	877.4666	439.2369	876.4825	438.7449	7
18	***2181.9063***	1091.4568	2164.8798	1082.9435	2163.8958	1082.4515	E	765.4505	383.2289	748.4240	374.7156	747.4400	374.2236	6
19	2282.9540	1141.9806	2265.9275	1133.4674	2264.9434	1132.9754	T	***636.4079***	318.7076	619.3814	310.1943	618.3974	309.7023	5

**20**	2430.0224	1215.5148	2412.9959	1207.0016	2412.0119	1206.5096	**F**	***535.3602***	268.1838	518.3337	259.6705			**4**
**21**	2543.1065	1272.0569	2526.0799	1263.5436	2525.0959	1263.0516	**L**	***388.2918***	194.6496	371.2653	186.1363			**3**
**22**	2671.2014	1336.1044	2654.1749	1327.5911	2653.1909	1327.0991	**K**	***275.2078***	138.1075	258.1812	129.5942			**2**
**23**							**K**	147.1128	74.0600	130.0863	65.5468			**1**

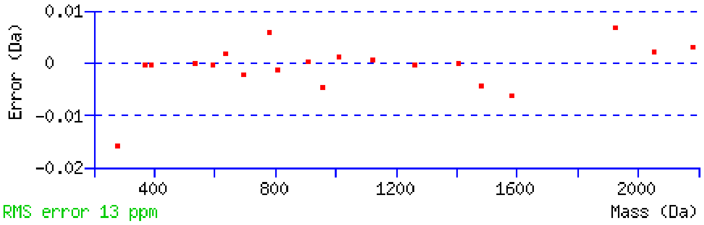

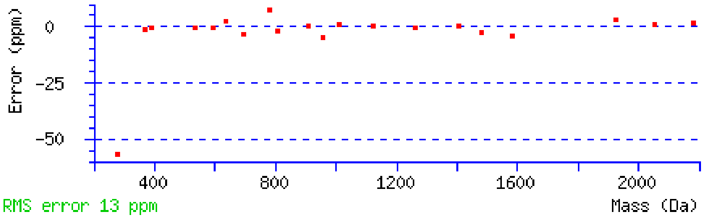

NCBI **BLAST** search of LVRPEVDVMCTAFHDNEETFLKK
(Parameters: blastp, nr protein database, expect=20000, no filter, PAM30)
Other BLAST web gateways

All matches to this query

Score	Mr(calc):	Delta	Sequence
56.3	2816.2997	-0.0000	LVRPEVDVMCTAFHDNEETFLKK

**Mascot:** http://www.matrixscience.com/

Mascot Search Results

Peptide View

MS/MS Fragmentation of **CCTESLVNR**
Found in **sp|P02768|ALBU_HUMAN**, Serum albumin OS=Homo sapiens GN=ALB PE=1 SV=2

Match to Query 1269: 1119.421368 from(560.717960,2+) intensity(219617.5781) scans(8158) rtinseconds(1460) index(7090)
Title: 150801_TTSH_Patient_Plasma_28_Spectrum024491_scans__8158_RTINSECONDS=1460
Data file L:\\Ard_TTSH\\T1D\\T150801_TTSH_Patient_Plasma_28.mgf

Click mouse within plot area to zoom in by factor of two about that point
Or, Plot from 50 to 850 Da Full range
Label all possible matches ○ Label matches used for scoring ⦿

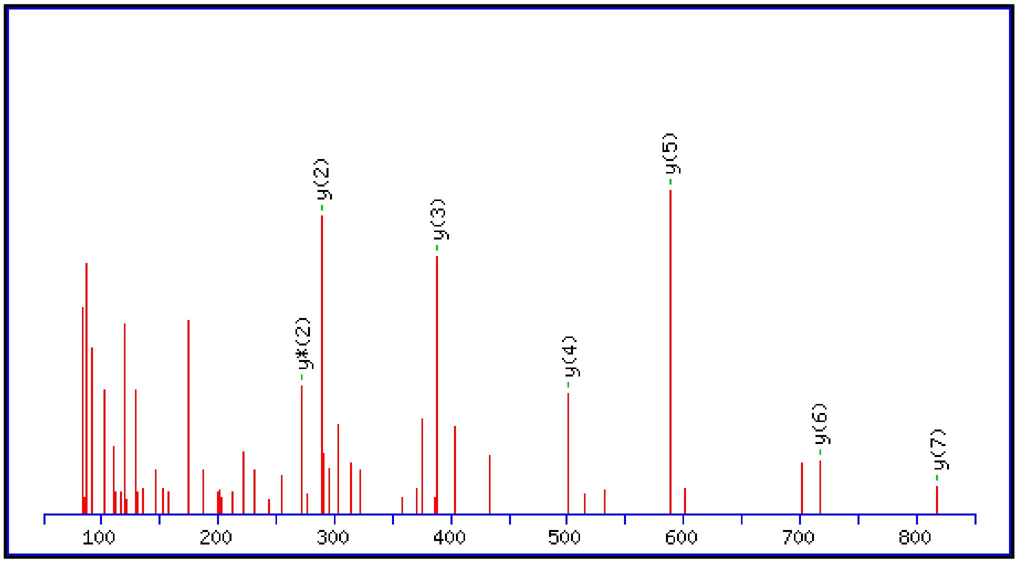

Monoisotopic mass of neutral peptide Mr(calc): 1119.4172
Variable modifications:
C1 : 4Trioxidation (CMWY)
C2 : 4Trioxidation (CMWY)
Ions Score: 55 **Expect:** 0.00018
Matches : 7/70 fragment ions using 8 most intense peaks (help)

#	b	b^{++}	b*	b^{*++}	b^0	b^{0++}	Seq.	y	y^{++}	y*	y^{*++}	y^0	y^{0++}	#
1	152.0012	76.5042					C							9
2	302.9951	152.0012					C	969.4306	485.2189	952.4040	476.7057	951.4200	476.2136	8
3	404.0428	202.5250			386.0322	193.5198	T	***818.4367***	409.7220	801.4101	401.2087	800.4261	400.7167	7
4	533.0854	267.0463			515.0748	258.0411	E	***717.3890***	359.1981	700.3624	350.6849	699.3784	350.1928	6
5	620.1174	310.5624			602.1069	301.5571	S	***588.3464***	294.6768	571.3198	286.1636	570.3358	285.6715	5
6	733.2015	367.1044			715.1909	358.0991	L	***501.3144***	251.1608	484.2878	242.6475			4
7	832.2699	416.6386			814.2593	407.6333	V	***388.2303***	194.6188	371.2037	186.1055			3
8	946.3128	473.6601	929.2863	465.1468	928.3023	464.6548	N	***289.1619***	145.0846	272.1353	136.5713			2
9							R	175.1190	88.0631	158.0924	79.5498			1

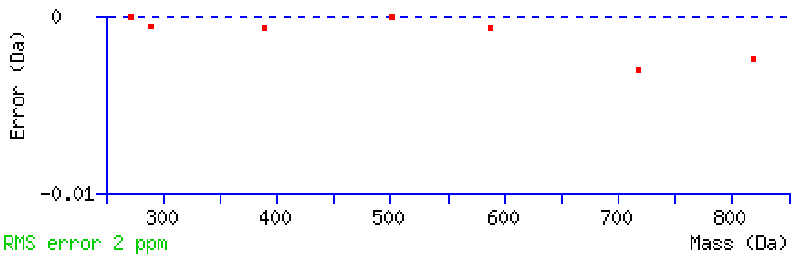

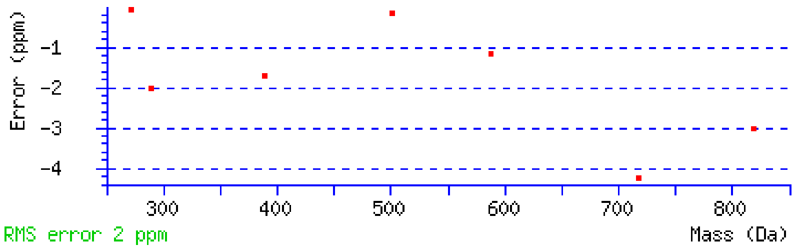

NCBI **BLAST** search of CCTESLVNR
(Parameters: blastp, nr protein database, expect=20000, no filter, PAM30)
Other BLAST web gateways

All matches to this query

Score	Mr(calc):	Delta	Sequence
54.8	1119.4172	0.0041	CCTESLVNR

**Mascot:** http://www.matrixscience.com/

{MATRIX SCIENCE} Mascot Search Results

Peptide View

MS/MS Fragmentation of **DLGEENFKALVLIAFAQYLQQCPFEDHVK**
Found in **sp|P02768|ALBU_HUMAN**, Serum albumin OS=Homo sapiens GN=ALB PE=1 SV=2

Match to Query 14604: 3412.664952 from(1138.562260,3+) intensity(338621.3125) scans(17368) rtinseconds(3120) index(14356)
Title: 150801_TTSH_Patient_Plasma_60_Spectrum031142_scans__17368_RTINSECONDS=3120
Data file L:\\Ard_TTSH\\T1D\\T150801_TTSH_Patient_Plasma_60.mgf

Click mouse within plot area to zoom in by factor of two about that point
Or, Plot from 200 to 1600 Da Full range
Label all possible matches ○ Label matches used for scoring ⊙

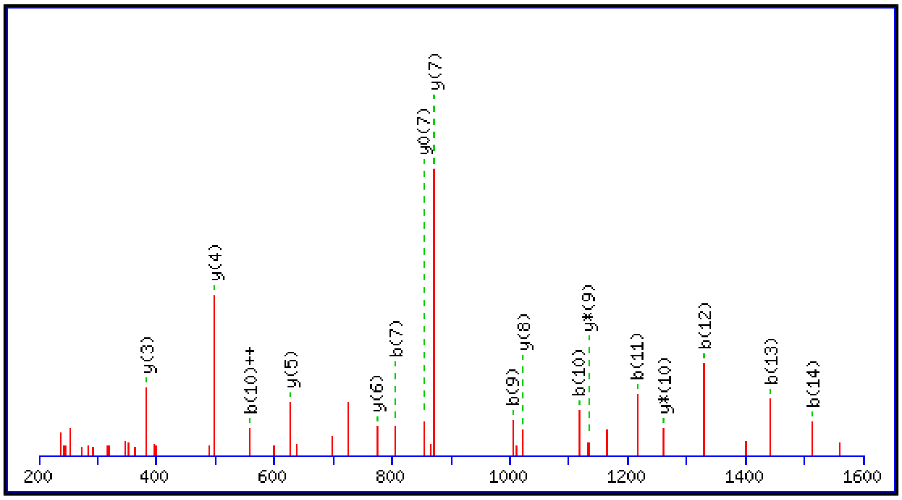

Monoisotopic mass of neutral peptide Mr(calc): 3412.6649
Variable modifications:
C22 : 4Trioxidation (CMWY)
Ions Score: 53 **Expect:** 0.0023
Matches : 17/320 fragment ions using 26 most intense peaks (help)

#	b	b^{++}	b*	b^{*++}	b^0	b^{0++}	Seq.	y	y^{++}	y*	y^{*++}	y^0	y^{0++}	#
1	116.0342	58.5207			98.0237	49.5155	D							29
2	229.1183	115.0628			211.1077	106.0575	L	3298.6453	1649.8263	3281.6187	1641.3130	3280.6347	1640.8210	28
3	286.1397	143.5735			268.1292	134.5682	G	3185.5612	1593.2842	3168.5347	1584.7710	3167.5507	1584.2790	27
4	415.1823	208.0948			397.1718	199.0895	E	3128.5398	1564.7735	3111.5132	1556.2602	3110.5292	1555.7682	26
5	544.2249	272.6161			526.2144	263.6108	E	2999.4972	1500.2522	2982.4706	1491.7389	2981.4866	1491.2469	25
6	658.2679	329.6376	641.2413	321.1243	640.2573	320.6323	N	2870.4546	1435.7309	2853.4280	1427.2176	2852.4440	1426.7256	24
7	***805.3363***	403.1718	788.3097	394.6585	787.3257	394.1665	F	2756.4116	1378.7095	2739.3851	1370.1962	2738.4011	1369.7042	23
8	933.4312	467.2193	916.4047	458.7060	915.4207	458.2140	K	2609.3432	1305.1753	2592.3167	1296.6620	2591.3327	1296.1700	22
9	***1004.4684***	502.7378	987.4418	494.2245	986.4578	493.7325	A	2481.2483	1241.1278	2464.2217	1232.6145	2463.2377	1232.1225	21
10	***1117.5524***	**559.2798**	1100.5259	550.7666	1099.5418	550.2746	L	2410.2111	1205.6092	2393.1846	1197.0959	2392.2006	1196.6039	20
11	***1216.6208***	608.8141	1199.5943	600.3008	1198.6103	599.8088	V	2297.1271	1149.0672	2280.1005	1140.5539	2279.1165	1140.0619	19
12	***1329.7049***	665.3561	1312.6783	656.8428	1311.6943	656.3508	L	2198.0587	1099.5330	2181.0321	1091.0197	2180.0481	1090.5277	18
13	***1442.7890***	721.8981	1425.7624	713.3848	1424.7784	712.8928	I	2084.9746	1042.9909	2067.9481	1034.4777	2066.9640	1033.9857	17
14	***1513.8261***	757.4167	1496.7995	748.9034	1495.8155	748.4114	A	1971.8905	986.4489	1954.8640	977.9356	1953.8800	977.4436	16
15	1660.8945	830.9509	1643.8679	822.4376	1642.8839	821.9456	F	1900.8534	950.9304	1883.8269	942.4171	1882.8429	941.9251	15
16	1731.9316	866.4694	1714.9050	857.9562	1713.9210	857.4642	A	1753.7850	877.3961	1736.7585	868.8829	1735.7744	868.3909	14
17	1859.9902	930.4987	1842.9636	921.9855	1841.9796	921.4934	Q	1682.7479	841.8776	1665.7214	833.3643	1664.7373	832.8723	13
18	2023.0535	1012.0304	2006.0270	1003.5171	2005.0429	1003.0251	Y	1554.6893	777.8483	1537.6628	769.3350	1536.6788	768.8430	12
19	2136.1376	1068.5724	2119.1110	1060.0591	2118.1270	1059.5671	L	1391.6260	696.3166	1374.5994	687.8034	1373.6154	687.3114	11

20	2264.1961	1132.6017	2247.1696	1124.0884	2246.1856	1123.5964	Q	1278.5419	639.7746	1261.5154	631.2613	1260.5314	630.7693	10
21	2392.2547	1196.6310	2375.2282	1188.1177	2374.2442	1187.6257	Q	1150.4834	575.7453	1133.4568	567.2320	1132.4728	566.7400	9
22	2543.2487	1272.1280	2526.2221	1263.6147	2525.2381	1263.1227	C	***1022.4248***	511.7160	1005.3982	503.2027	1004.4142	502.7107	8
23	2640.3014	1320.6543	2623.2749	1312.1411	2622.2909	1311.6491	P	***871.4308***	436.2191	854.4043	427.7058	853.4203	427.2138	7
24	2787.3698	1394.1886	2770.3433	1385.6753	2769.3593	1385.1833	F	***774.3781***	387.6927	757.3515	379.1794	756.3675	378.6874	6
25	2916.4124	1458.7099	2899.3859	1450.1966	2898.4019	1449.7046	E	***627.3097***	314.1585	610.2831	305.6452	609.2991	305.1532	5
26	3031.4394	1516.2233	3014.4128	1507.7100	3013.4288	1507.2180	D	***498.2671***	249.6372	481.2405	241.1239	480.2565	240.6319	4
27	3168.4983	1584.7528	3151.4717	1576.2395	3150.4877	1575.7475	H	***383.2401***	192.1237	366.2136	183.6104			3
28	3267.5667	1634.2870	3250.5401	1625.7737	3249.5561	1625.2817	V	246.1812	123.5942	229.1547	115.0810			2
29							K	147.1128	74.0600	130.0863	65.5468			1

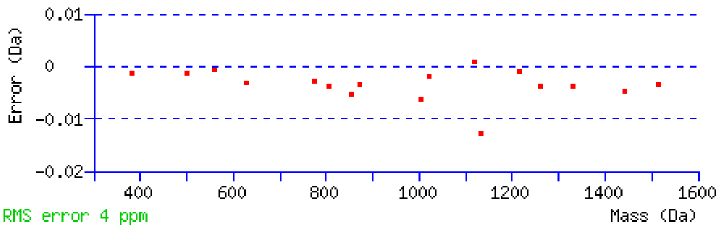

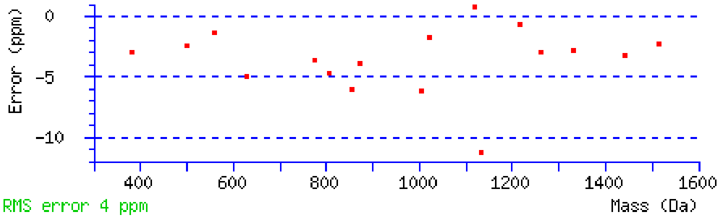

NCBI **BLAST** search of DLGEENFKALVLIAFAQYLQQCPFEDHVK
(Parameters: blastp, nr protein database, expect=20000, no filter, PAM30)
Other BLAST web gateways

All matches to this query

Score	Mr(calc):	Delta	Sequence
53.3	3412.6649	0.0000	DLGEENFKALVLIAFAQYLQQCPFEDHVK
43.1	3412.6649	0.0000	DLGEENFKALVLIAFAQYLQQCPFEDHVK

Mascot Search Results

Peptide View

MS/MS Fragmentation of **SHCIAEVENDEMPADLPSLAADFVESK**
Found in **sp|P02768|ALBU_HUMAN**, Serum albumin OS=Homo sapiens GN=ALB PE=1 SV=2

Match to Query 13335: 3012.284028 from(1507.149290,2+) intensity(0.0000) scans(14939) rtinseconds(2739) index(12582)
Title: 150825_TTSH_Patient_Plasma_56_Spectrum029170_scans__14939_RTINSECONDS=2739
Data file L:\\Ard_TTSH\\T1D\\T150825_TTSH_Patient_Plasma_56.mgf

Click mouse within plot area to zoom in by factor of two about that point
Or, Plot from 200 to 3000 Da Full range
Label all possible matches ○ Label matches used for scoring ⊙

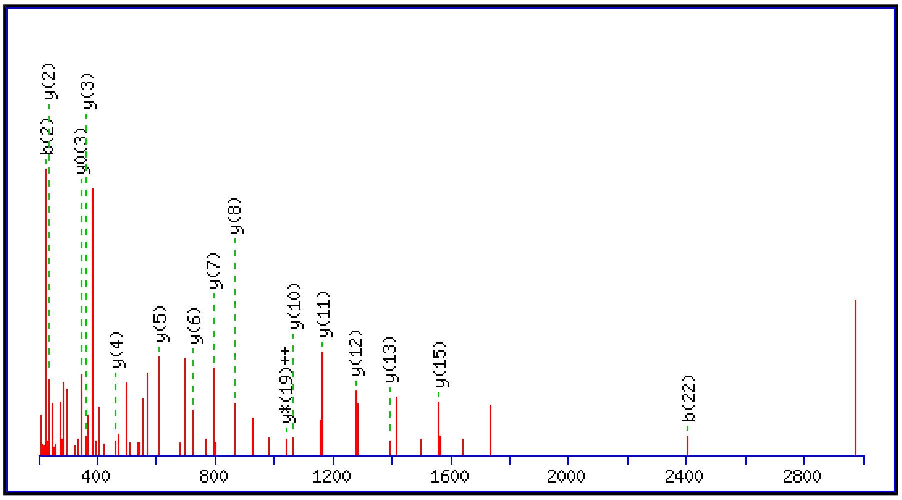

Monoisotopic mass of neutral peptide Mr(calc): 3012.2852
Variable modifications:
C3 : 4Trioxidation (CMWY)
M12 : 4Trioxidation (CMWY)
Ions Score: 51 **Expect:** 0.0031
Matches : 16/294 fragment ions using 41 most intense peaks (help)

#	b	b^{++}	b*	b^{*++}	b^{0}	b^{0++}	Seq.	y	y^{++}	y*	y^{*++}	y^{0}	y^{0++}	#
1	88.0393	44.5233			70.0287	35.5180	S							27
2	**225.0982**	113.0527			207.0877	104.0475	H	2926.2605	1463.6339	2909.2339	1455.1206	2908.2499	1454.6286	26
3	376.0921	188.5497			358.0816	179.5444	C	2789.2016	1395.1044	2772.1750	1386.5911	2771.1910	1386.0991	25
4	489.1762	245.0917			471.1656	236.0865	I	2638.2076	1319.6075	2621.1811	1311.0942	2620.1971	1310.6022	24
5	560.2133	280.6103			542.2028	271.6050	A	2525.1236	1263.0654	2508.0970	1254.5521	2507.1130	1254.0601	23
6	689.2559	345.1316			671.2454	336.1263	E	2454.0865	1227.5469	2437.0599	1219.0336	2436.0759	1218.5416	22
7	788.3243	394.6658			770.3138	385.6605	V	2325.0439	1163.0256	2308.0173	1154.5123	2307.0333	1154.0203	21
8	917.3669	459.1871			899.3564	450.1818	E	2225.9754	1113.4914	2208.9489	1104.9781	2207.9649	1104.4861	20
9	1031.4099	516.2086	1014.3833	507.6953	1013.3993	507.2033	N	2096.9329	1048.9701	2079.9063	1040.4568	2078.9223	1039.9648	19
10	1146.4368	573.7220	1129.4102	565.2088	1128.4262	564.7168	D	1982.8899	991.9486	1965.8634	983.4353	1964.8794	982.9433	18
11	1275.4794	638.2433	1258.4528	629.7301	1257.4688	629.2380	E	1867.8630	934.4351	1850.8364	925.9219	1849.8524	925.4298	17
12	1454.5046	727.7559	1437.4781	719.2427	1436.4941	718.7507	M	1738.8204	869.9138	1721.7938	861.4006	1720.8098	860.9086	16
13	1551.5574	776.2823	1534.5308	767.7691	1533.5468	767.2770	P	***1559.7952***	780.4012	1542.7686	771.8879	1541.7846	771.3959	15
14	1622.5945	811.8009	1605.5679	803.2876	1604.5839	802.7956	A	1462.7424	731.8748	1445.7159	723.3616	1444.7318	722.8696	14
15	1737.6214	869.3144	1720.5949	860.8011	1719.6109	860.3091	D	***1391.7053***	696.3563	1374.6787	687.8430	1373.6947	687.3510	13
16	1850.7055	925.8564	1833.6790	917.3431	1832.6949	916.8511	L	***1276.6783***	638.8428	1259.6518	630.3295	1258.6678	629.8375	12
17	1947.7583	974.3828	1930.7317	965.8695	1929.7477	965.3775	P	***1163.5943***	582.3008	1146.5677	573.7875	1145.5837	573.2955	11
18	2034.7903	1017.8988	2017.7637	1009.3855	2016.7797	1008.8935	S	***1066.5415***	533.7744	1049.5150	525.2611	1048.5310	524.7691	10
19	2147.8744	1074.4408	2130.8478	1065.9275	2129.8638	1065.4355	L	979.5095	490.2584	962.4829	481.7451	961.4989	481.2531	9

**20**	2218.9115	1109.9594	2201.8849	1101.4461	2200.9009	1100.9541	**A**	***866.4254***	433.7164	849.3989	425.2031	848.4149	424.7111	**8**
**21**	2289.9486	1145.4779	2272.9220	1136.9647	2271.9380	1136.4726	**A**	***795.3883***	398.1978	778.3618	389.6845	777.3777	389.1925	**7**
**22**	**2404.9755**	1202.9914	2387.9490	1194.4781	2386.9650	1193.9861	**D**	***724.3512***	362.6792	707.3246	354.1660	706.3406	353.6740	**6**
**23**	2552.0439	1276.5256	2535.0174	1268.0123	2534.0334	1267.5203	**F**	***609.3243***	305.1658	592.2977	296.6525	591.3137	296.1605	**5**
**24**	2651.1124	1326.0598	2634.0858	1317.5465	2633.1018	1317.0545	**V**	***462.2558***	231.6316	445.2293	223.1183	444.2453	222.6263	**4**
**25**	2780.1549	1390.5811	2763.1284	1382.0678	2762.1444	1381.5758	**E**	***363.1874***	182.0974	346.1609	173.5841	345.1769	173.0921	**3**
**26**	2867.1870	1434.0971	2850.1604	1425.5839	2849.1764	1425.0918	**S**	***234.1448***	117.5761	217.1183	109.0628	216.1343	108.5708	**2**
**27**							**K**	147.1128	74.0600	130.0863	65.5468			**1**

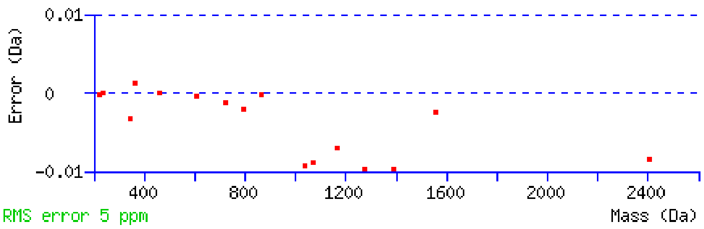

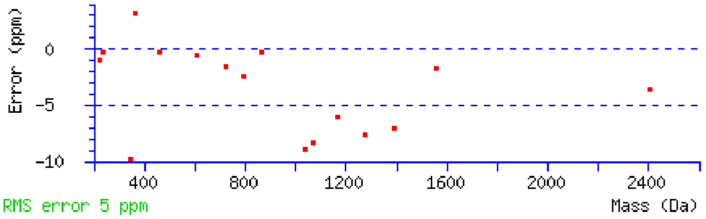

NCBI **BLAST** search of SHCIAEVENDEMPADLPSLAADFVESK
(Parameters: blastp, nr protein database, expect=20000, no filter, PAM30)
Other BLAST web gateways

All matches to this query

Score	Mr(calc):	Delta	Sequence
51.0	3012.2852	-0.0012	SHCIAEVENDEMPADLPSLAADFVESK
0.1	3010.2631	2.0210	GTISQYFTTLHCPVCDDLTQHGICSK

Mascot: http://www.matrixscience.com/

Mascot Search Results

Peptide View

MS/MS Fragmentation of **ADDKETCFAEEGK**
Found in **sp|P02768|ALBU_HUMAN**, Serum albumin OS=Homo sapiens GN=ALB PE=1 SV=2

Match to Query 7544: 1489.593728 from(745.804140,2+) intensity(161432.0469) scans(4285) rtinseconds(867) index(3471)
Title: 150818_TTSH_Patient_Plasma_83_Spectrum020095_scans__4285_RTINSECONDS=867
Data file L:\\Ard_TTSH\\T1D\\T150818_TTSH_Patient_Plasma_83.mgf

Click mouse within plot area to zoom in by factor of two about that point
Or, Plot from 100 to 700 Da Full range
Label all possible matches ○ Label matches used for scoring ⊙

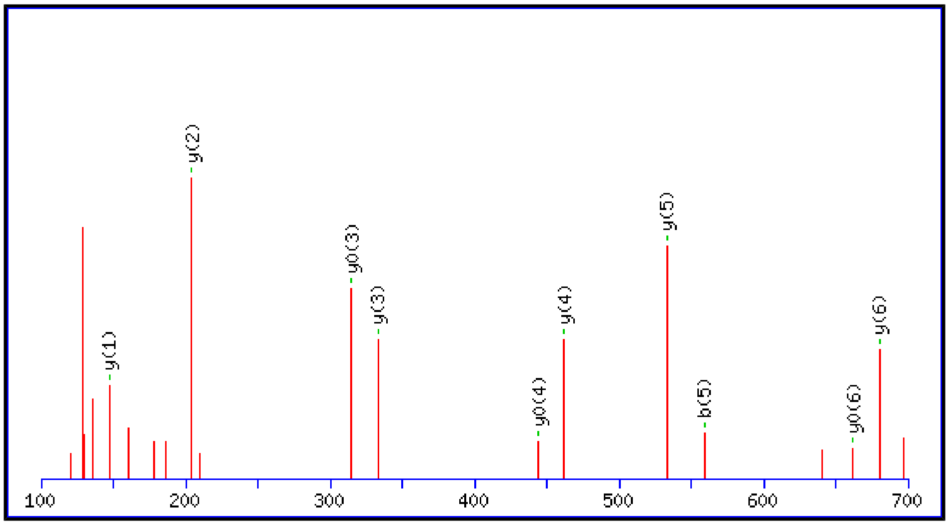

Monoisotopic mass of neutral peptide Mr(calc): 1489.5878
Variable modifications:
C7 : 4Trioxidation (CMWY)
Ions Score: 51 **Expect:** 0.00081
Matches : 10/132 fragment ions using 12 most intense peaks (help)

#	b	b++	b*	b*++	b0	b0++	Seq.	y	y++	y*	y*++	y0	y0++	#
1	72.0444	36.5258					A							13
2	187.0713	94.0393			169.0608	85.0340	D	1419.5580	710.2827	1402.5315	701.7694	1401.5475	701.2774	12
3	302.0983	151.5528			284.0877	142.5475	D	1304.5311	652.7692	1287.5045	644.2559	1286.5205	643.7639	11
4	430.1932	215.6003	413.1667	207.0870	412.1827	206.5950	K	1189.5041	595.2557	1172.4776	586.7424	1171.4936	586.2504	10
5	**559.2358**	280.1216	542.2093	271.6083	541.2253	271.1163	E	1061.4092	531.2082	1044.3826	522.6950	1043.3986	522.2029	9
6	660.2835	330.6454	643.2570	322.1321	642.2729	321.6401	T	932.3666	466.6869	915.3400	458.1737	914.3560	457.6817	8
7	811.2774	406.1424	794.2509	397.6291	793.2669	397.1371	C	831.3189	416.1631	814.2924	407.6498	813.3083	407.1578	7
8	958.3459	479.6766	941.3193	471.1633	940.3353	470.6713	F	***680.3250***	340.6661	663.2984	332.1529	662.3144	331.6608	6
9	1029.3830	515.1951	1012.3564	506.6818	1011.3724	506.1898	A	***533.2566***	267.1319	516.2300	258.6186	515.2460	258.1266	5
10	1158.4256	579.7164	1141.3990	571.2031	1140.4150	570.7111	E	***462.2195***	231.6134	445.1929	223.1001	444.2089	222.6081	4
11	1287.4682	644.2377	1270.4416	635.7244	1269.4576	635.2324	E	***333.1769***	167.0921	316.1503	158.5788	315.1663	158.0868	3
12	1344.4896	672.7484	1327.4631	664.2352	1326.4791	663.7432	G	***204.1343***	102.5708	187.1077	94.0575			2
13							K	***147.1128***	74.0600	130.0863	65.5468			1

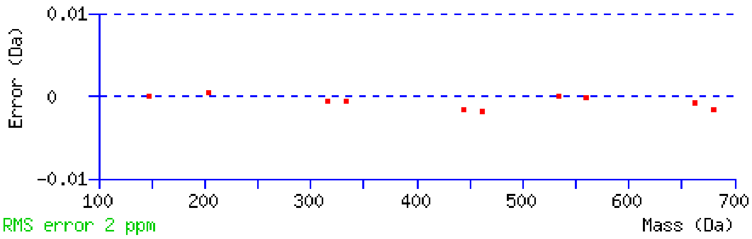

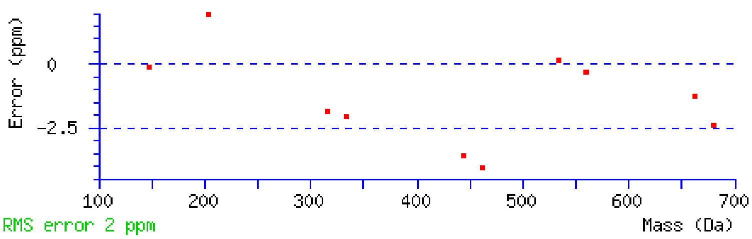

NCBI **BLAST** search of ADDKETCFAEEGK
(Parameters: blastp, nr protein database, expect=20000, no filter, PAM30)
Other BLAST web gateways

All matches to this query

Score	Mr(calc):	Delta	Sequence
50.7	1489.5878	0.0059	ADDKETCFAEEGK
2.1	1489.6069	-0.0132	ESEHTGPGWESAR

Mascot: http://www.matrixscience.com/

Mascot Search Results

Peptide View

MS/MS Fragmentation of **VTKCCTESLVNR**
Found in **sp|P02768|ALBU_HUMAN**, Serum albumin OS=Homo sapiens GN=ALB PE=1 SV=2

Match to Query 4050: 1447.628268 from(724.821410,2+) intensity(581654.6250) scans(5660) rtinseconds(1069) index(4659)
Title: 150801_TTSH_Patient_Plasma_37_Spectrum021573_scans__5660_RTINSECONDS=1069
Data file L:\\Ard_TTSH\\T1D\\T150801_TTSH_Patient_Plasma_37.mgf

Click mouse within plot area to zoom in by factor of two about that point
Or, Plot from 100 to 1500 Da Full range
Label all possible matches ○ Label matches used for scoring ⦿

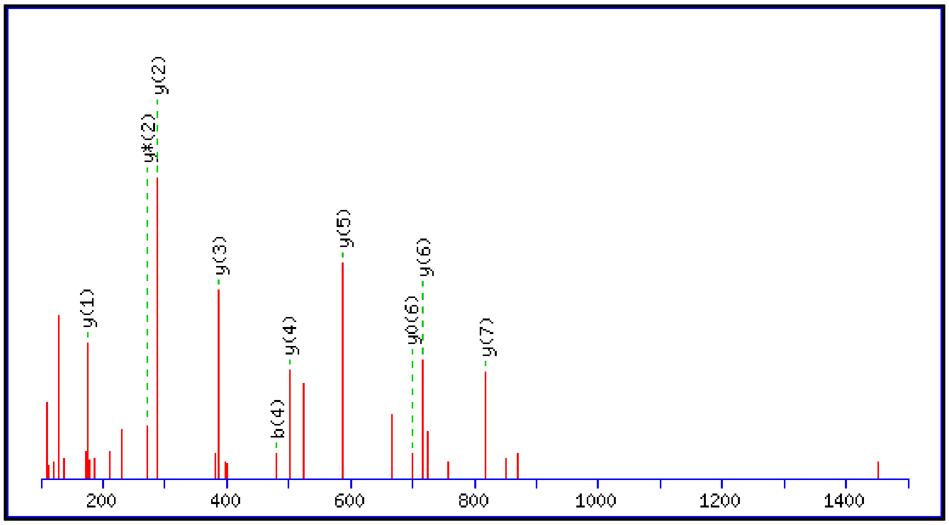

Monoisotopic mass of neutral peptide Mr(calc): 1447.6283
Variable modifications:
C4 : 4Trioxidation (CMWY)
C5 : 4Trioxidation (CMWY)
Ions Score: 50 **Expect:** 0.0021
Matches : 10/118 fragment ions using 16 most intense peaks (help)

#	b	b^{++}	b^{*}	b^{*++}	b^{0}	b^{0++}	Seq.	y	y^{++}	y^{*}	y^{*++}	y^{0}	y^{0++}	#
**1**	100.0757	50.5415					**V**							**12**
**2**	201.1234	101.0653			183.1128	92.0600	**T**	1349.5672	675.2872	1332.5406	666.7739	1331.5566	666.2819	**11**
**3**	329.2183	165.1128	312.1918	156.5995	311.2078	156.1075	**K**	1248.5195	624.7634	1231.4929	616.2501	1230.5089	615.7581	**10**
**4**	**480.2123**	240.6098	463.1857	232.0965	462.2017	231.6045	**C**	1120.4245	560.7159	1103.3980	552.2026	1102.4139	551.7106	**9**
**5**	631.2062	316.1067	614.1796	307.5935	613.1956	307.1015	**C**	969.4306	485.2189	952.4040	476.7057	951.4200	476.2136	**8**
**6**	732.2539	366.6306	715.2273	358.1173	714.2433	357.6253	**T**	***818.4367***	409.7220	801.4101	401.2087	800.4261	400.7167	**7**
**7**	861.2965	431.1519	844.2699	422.6386	843.2859	422.1466	**E**	***717.3890***	359.1981	700.3624	350.6849	699.3784	350.1928	**6**
**8**	948.3285	474.6679	931.3019	466.1546	930.3179	465.6626	**S**	***588.3464***	294.6768	571.3198	286.1636	570.3358	285.6715	**5**
**9**	1061.4126	531.2099	1044.3860	522.6966	1043.4020	522.2046	**L**	***501.3144***	251.1608	484.2878	242.6475			**4**
**10**	1160.4810	580.7441	1143.4544	572.2308	1142.4704	571.7388	**V**	***388.2303***	194.6188	371.2037	186.1055			**3**
**11**	1274.5239	637.7656	1257.4973	629.2523	1256.5133	628.7603	**N**	***289.1619***	145.0846	272.1353	136.5713			**2**
**12**							**R**	***175.1190***	88.0631	158.0924	79.5498			**1**

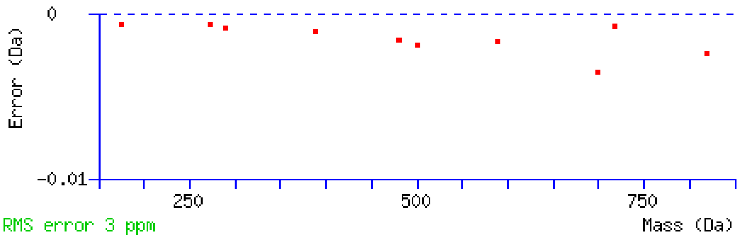

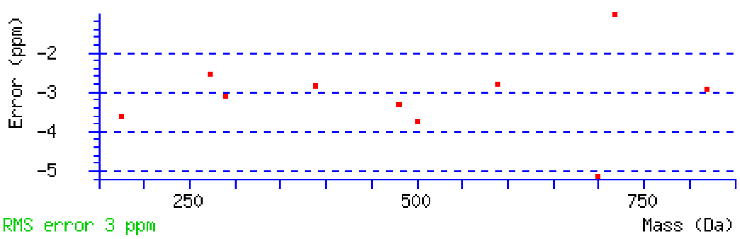

NCBI **BLAST** search of VTKCCTESLVNR
(Parameters: blastp, nr protein database, expect=20000, no filter, PAM30)
Other BLAST web gateways

All matches to this query

Score	Mr(calc):	Delta	Sequence
49.9	1447.6283	-0.0000	VTKCCTESLVNR
3.7	1447.6415	-0.0133	GWGCGQAWGASVGGR

**Mascot:** http://www.matrixscience.com/

MATRIX SCIENCE Mascot Search Results

Peptide View

MS/MS Fragmentation of **LVRPEVDVMCTAFHDNEETFLKK**
Found in **sp|P02768|ALBU_HUMAN**, Serum albumin OS=Homo sapiens GN=ALB PE=1 SV=2

Match to Query 13436: 2816.304136 from(705.083310,4+) intensity(1886551.1250) scans(9916) rtinseconds(1788) index(8429)
Title: 150825_TTSH_Patient_Plasma_21_Spectrum026143_scans__9916_RTINSECONDS=1788
Data file L:\\Ard_TTSH\\T1D\\T150825_TTSH_Patient_Plasma_21.mgf

Click mouse within plot area to zoom in by factor of two about that point
Or, Plot from 0 to 3000 Da Full range
Label all possible matches ○ Label matches used for scoring ⦿

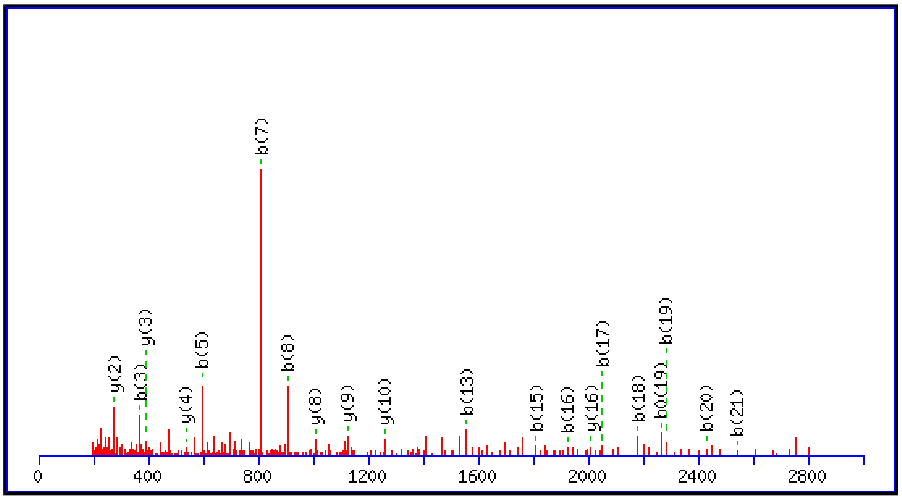

Monoisotopic mass of neutral peptide Mr(calc): 2816.2997
Variable modifications:
M9 : 4Trioxidation (CMWY)
C10 : 4Trioxidation (CMWY)
Ions Score: 48 **Expect:** 0.0095
Matches : 20/244 fragment ions using 53 most intense peaks (help)

#	b	b++	b*	b*++	b0	b0++	Seq.	y	y++	y*	y*++	y0	y0++	#
1	114.0913	57.5493					L							23
2	213.1598	107.0835					V	2704.2229	1352.6151	2687.1964	1344.1018	2686.2123	1343.6098	22
3	***369.2609***	185.1341	352.2343	176.6208			R	2605.1545	1303.0809	2588.1279	1294.5676	2587.1439	1294.0756	21
4	466.3136	233.6605	449.2871	225.1472			P	2449.0534	1225.0303	2432.0268	1216.5171	2431.0428	1216.0250	20
5	***595.3562***	298.1817	578.3297	289.6685	577.3457	289.1765	E	2352.0006	1176.5039	2334.9741	1167.9907	2333.9901	1167.4987	19
6	694.4246	347.7160	677.3981	339.2027	676.4141	338.7107	V	2222.9580	1111.9827	2205.9315	1103.4694	2204.9475	1102.9774	18
7	***809.4516***	405.2294	792.4250	396.7162	791.4410	396.2241	D	2123.8896	1062.4484	2106.8631	1053.9352	2105.8790	1053.4432	17
8	***908.5200***	454.7636	891.4934	446.2504	890.5094	445.7584	V	**2008.8627**	1004.9350	1991.8361	996.4217	1990.8521	995.9297	16
9	1087.5452	544.2762	1070.5187	535.7630	1069.5347	535.2710	M	1909.7943	955.4008	1892.7677	946.8875	1891.7837	946.3955	15
10	1238.5392	619.7732	1221.5126	611.2599	1220.5286	610.7679	C	1730.7690	865.8882	1713.7425	857.3749	1712.7585	856.8829	14
11	1339.5868	670.2971	1322.5603	661.7838	1321.5763	661.2918	T	1579.7751	790.3912	1562.7486	781.8779	1561.7645	781.3859	13
12	1410.6239	705.8156	1393.5974	697.3023	1392.6134	696.8103	A	1478.7274	739.8673	1461.7009	731.3541	1460.7169	730.8621	12
13	***1557.6924***	779.3498	1540.6658	770.8365	1539.6818	770.3445	F	1407.6903	704.3488	1390.6638	695.8355	1389.6797	695.3435	11
14	1694.7513	847.8793	1677.7247	839.3660	1676.7407	838.8740	H	**1260.6219**	630.8146	1243.5953	622.3013	1242.6113	621.8093	10
15	***1809.7782***	905.3927	1792.7517	896.8795	1791.7676	896.3875	D	**1123.5630**	562.2851	1106.5364	553.7719	1105.5524	553.2798	9
16	***1923.8211***	962.4142	1906.7946	953.9009	1905.8106	953.4089	N	**1008.5360**	504.7717	991.5095	496.2584	990.5255	495.7664	8
17	***2052.8637***	1026.9355	2035.8372	1018.4222	2034.8532	1017.9302	E	894.4931	447.7502	877.4666	439.2369	876.4825	438.7449	7
18	***2181.9063***	1091.4568	2164.8798	1082.9435	2163.8958	1082.4515	E	765.4505	383.2289	748.4240	374.7156	747.4400	374.2236	6
19	***2282.9540***	1141.9806	2265.9275	1133.4674	2264.9434	1132.9754	T	636.4079	318.7076	619.3814	310.1943	618.3974	309.7023	5

**20**	***2430.0224***	1215.5148	2412.9959	1207.0016	2412.0119	1206.5096	**F**	**535.3602**	268.1838	518.3337	259.6705			**4**
**21**	***2543.1065***	1272.0569	2526.0799	1263.5436	2525.0959	1263.0516	**L**	**388.2918**	194.6496	371.2653	186.1363			**3**
**22**	2671.2014	1336.1044	2654.1749	1327.5911	2653.1909	1327.0991	**K**	**275.2078**	138.1075	258.1812	129.5942			**2**
**23**							**K**	147.1128	74.0600	130.0863	65.5468			**1**

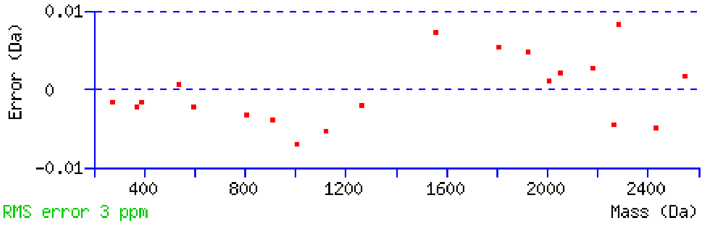

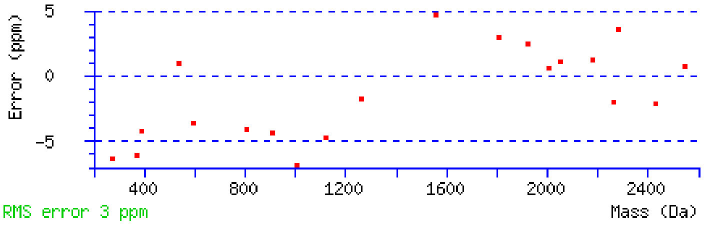

NCBI **BLAST** search of LVRPEVDVMCTAFHDNEETFLKK
(Parameters: blastp, nr protein database, expect=20000, no filter, PAM30)
Other BLAST web gateways

All matches to this query

Score	Mr(calc):	Delta	Sequence
47.5	2816.2997	0.0045	LVRPEVDVMCTAFHDNEETFLKK

**Mascot:** http://www.matrixscience.com/

{MATRIX SCIENCE} Mascot Search Results

Peptide View

MS/MS Fragmentation of **SLHTLFGDKLCTVATLR**
Found in **sp|P02768|ALBU_HUMAN**, Serum albumin OS=Homo sapiens GN=ALB PE=1 SV=2

Match to Query 9188: 1921.995936 from(481.506260,4+) intensity(1283057.1250) scans(9113) rtinseconds(1817) index(7143)
Title: N50825_TTSH_Patient_Plasma_42_Spectrum007269_scans__9113_RTINSECONDS=1817
Data file L:\\Ard_TTSH\\TN1D\\TN50825_TTSH_Patient_Plasma_42.mgf

Click mouse within plot area to zoom in by factor of two about that point
Or, [Plot from] 100 to 1300 Da [Full range]
Label all possible matches ○ Label matches used for scoring ⊙

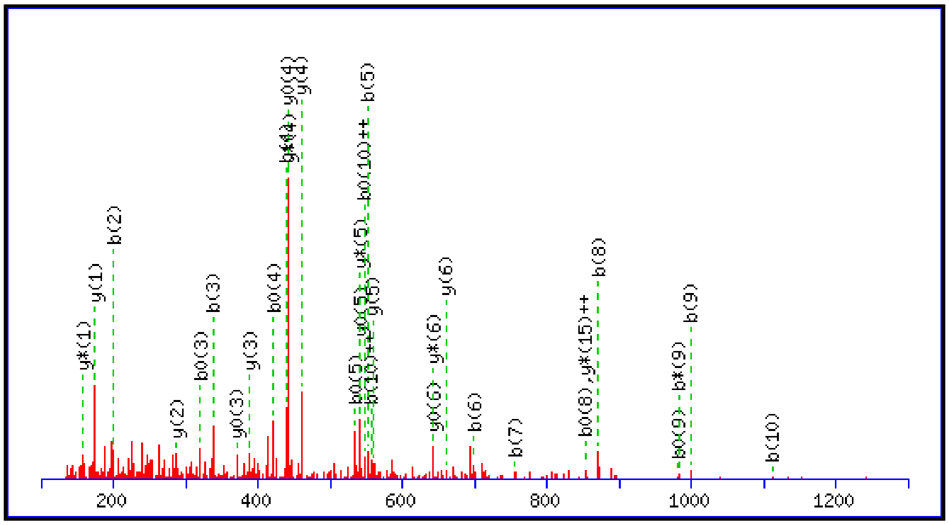

Monoisotopic mass of neutral peptide Mr(calc): 1921.9931
Variable modifications:
C11 : 4Trioxidation (CMWY)
Ions Score: 47 **Expect:** 0.0056
Matches : 32/172 fragment ions using 81 most intense peaks (help)

#	b	b^{++}	b*	b^{*++}	b^0	b^{0++}	Seq.	y	y^{++}	y*	y^{*++}	y^0	y^{0++}	#
1	88.0393	44.5233			70.0287	35.5180	S							17
2	**201.1234**	101.0653			183.1128	92.0600	L	1835.9684	918.4878	1818.9418	909.9746	1817.9578	909.4826	16
3	**338.1823**	169.5948			320.1717	160.5895	H	1722.8843	861.9458	1705.8578	853.4325	1704.8738	852.9405	15
4	**439.2300**	220.1186			421.2194	211.1133	T	1585.8254	793.4163	1568.7989	784.9031	1567.8149	784.4111	14
5	**552.3140**	276.6606			534.3035	267.6554	L	1484.7777	742.8925	1467.7512	734.3792	1466.7672	733.8872	13
6	**699.3824**	350.1949			681.3719	341.1896	F	1371.6937	686.3505	1354.6671	677.8372	1353.6831	677.3452	12
7	**756.4039**	378.7056			738.3933	369.7003	G	1224.6253	612.8163	1207.5987	604.3030	1206.6147	603.8110	11
8	**871.4308**	436.2191			853.4203	427.2138	D	1167.6038	584.3055	1150.5773	575.7923	1149.5932	575.3003	10
9	**999.5258**	500.2665	982.4993	491.7533	981.5152	491.2613	K	1052.5769	526.7921	1035.5503	518.2788	1034.5663	517.7868	9
10	**1112.6099**	**556.8086**	1095.5833	548.2953	1094.5993	547.8033	L	924.4819	462.7446	907.4553	454.2313	906.4713	453.7393	8
11	1263.6038	632.3055	1246.5773	623.7923	1245.5932	623.3003	C	811.3978	406.2026	794.3713	397.6893	793.3873	397.1973	7
12	1364.6515	682.8294	1347.6249	674.3161	1346.6409	673.8241	T	**660.4039**	330.7056	643.3774	322.1923	642.3933	321.7003	6
13	1463.7199	732.3636	1446.6933	723.8503	1445.7093	723.3583	V	**559.3562**	280.1817	542.3297	271.6685	541.3457	271.1765	5
14	1534.7570	767.8821	1517.7305	759.3689	1516.7464	758.8769	A	**460.2878**	230.6475	443.2613	222.1343	442.2772	221.6423	4
15	1635.8047	818.4060	1618.7781	809.8927	1617.7941	809.4007	T	**389.2507**	195.1290	372.2241	186.6157	371.2401	186.1237	3
16	1748.8887	874.9480	1731.8622	866.4347	1730.8782	865.9427	L	**288.2030**	144.6051	271.1765	136.0919			2
17							R	**175.1190**	88.0631	158.0924	79.5498			1

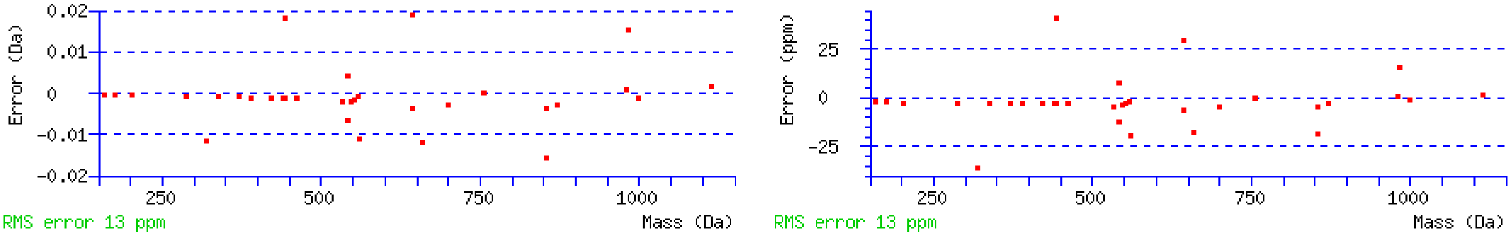

NCBI **BLAST** search of SLHTLFGDKLCTVATLR
(Parameters: blastp, nr protein database, expect=20000, no filter, PAM30)
Other BLAST web gateways

All matches to this query

Score	Mr(calc):	Delta	Sequence
46.5	1921.9931	0.0028	SLHTLFGDKLCTVATLR

MATRIX SCIENCE Mascot Search Results

Peptide View

MS/MS Fragmentation of **LKCASLQKFGER**
Found in **sp|P02768|ALBU_HUMAN**, Serum albumin OS=Homo sapiens GN=ALB PE=1 SV=2

Match to Query 4478: 1426.721622 from(476.581150,3+) intensity(325013.6563) scans(5097) rtinseconds(983) index(3674)
Title: 150808_TTSH_Patient_Plasma_57_Spectrum020309_scans__5097_RTINSECONDS=983
Data file L:\\Ard_TTSH\\T1D\\T150808_TTSH_Patient_Plasma_57.mgf

Click mouse within plot area to zoom in by factor of two about that point
Or, Plot from 100 to 1400 Da Full range
Label all possible matches ○ Label matches used for scoring ⊙

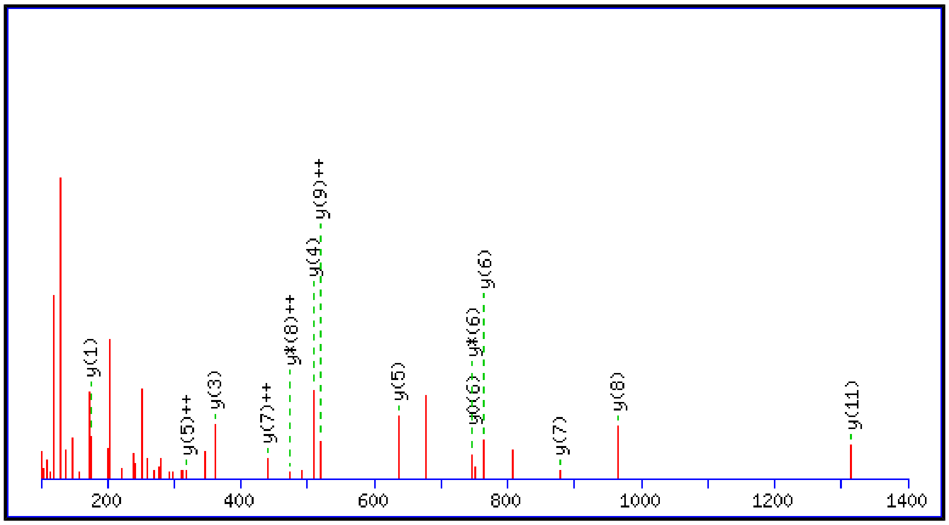

Monoisotopic mass of neutral peptide Mr(calc): 1426.7238
Variable modifications:
C3 : 4Trioxidation (CMWY)
Ions Score: 46 **Expect:** 0.011
Matches : 14/120 fragment ions using 28 most intense peaks (help)

#	b	b^{++}	b*	b*$^{++}$	b^{0}	b^{0++}	Seq.	y	y^{++}	y*	y*$^{++}$	y^{0}	y^{0++}	#
1	114.0913	57.5493					L							12
2	242.1863	121.5968	225.1598	113.0835			K	***1314.6471***	657.8272	1297.6205	649.3139	1296.6365	648.8219	11
3	393.1802	197.0938	376.1537	188.5805			C	1186.5521	593.7797	1169.5255	585.2664	1168.5415	584.7744	10
4	464.2173	232.6123	447.1908	224.0990			A	1035.5582	518.2827	1018.5316	509.7694	1017.5476	509.2774	9
5	551.2494	276.1283	534.2228	267.6151	533.2388	267.1230	S	***964.5211***	482.7642	947.4945	474.2509	946.5105	473.7589	8
6	664.3334	332.6704	647.3069	324.1571	646.3229	323.6651	L	***877.4890***	439.2482	860.4625	430.7349	859.4785	430.2429	7
7	792.3920	396.6996	775.3655	388.1864	774.3815	387.6944	Q	***764.4050***	382.7061	747.3784	374.1928	746.3944	373.7008	6
8	920.4870	460.7471	903.4604	452.2339	902.4764	451.7418	K	***636.3464***	318.6768	619.3198	310.1636	618.3358	309.6715	5
9	1067.5554	534.2813	1050.5288	525.7681	1049.5448	525.2761	F	***508.2514***	254.6293	491.2249	246.1161	490.2409	245.6241	4
10	1124.5769	562.7921	1107.5503	554.2788	1106.5663	553.7868	G	***361.1830***	181.0951	344.1565	172.5819	343.1724	172.0899	3
11	1253.6194	627.3134	1236.5929	618.8001	1235.6089	618.3081	E	304.1615	152.5844	287.1350	144.0711	286.1510	143.5791	2
12							R	***175.1190***	88.0631	158.0924	79.5498			1

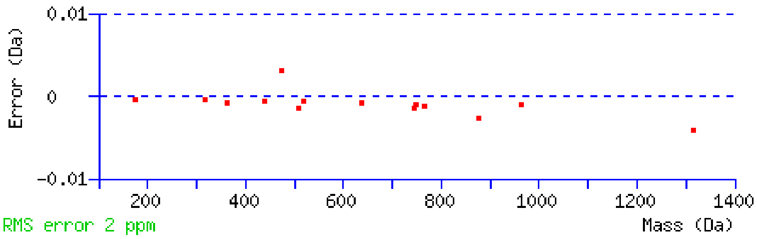

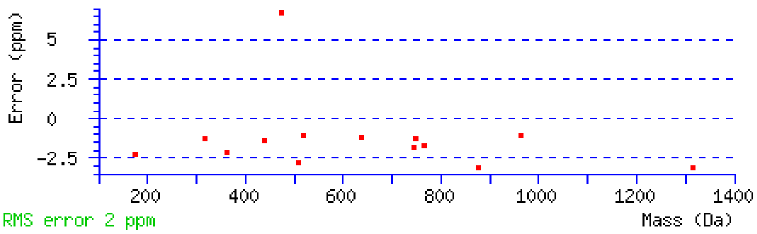

NCBI **BLAST** search of LKCASLQKFGER
(Parameters: blastp, nr protein database, expect=20000, no filter, PAM30)
Other BLAST web gateways

All matches to this query

Score	Mr(calc):	Delta	Sequence
45.8	1426.7238	-0.0022	LKCASLQKFGER

Mascot: http://www.matrixscience.com/

Mascot Search Results

Peptide View

MS/MS Fragmentation of **EFNAETFTFHADICTLSEK**
Found in **sp|P02768|ALBU_HUMAN**, Serum albumin OS=Homo sapiens GN=ALB PE=1 SV=2

Match to Query 11313: 2249.974208 from(1125.994380,2+) intensity(172060.1406) scans(15330) rtinseconds(3099) index(12278)
Title: 150801_TTSH_Patient_Plasma_88_Spectrum026956_scans__15330_RTINSECONDS=3099
Data file L:\\Ard_TTSH\\T1D\\T150801_TTSH_Patient_Plasma_88.mgf

Click mouse within plot area to zoom in by factor of two about that point
Or, Plot from 150 to 750 Da Full range
Label all possible matches ○ Label matches used for scoring ⊙

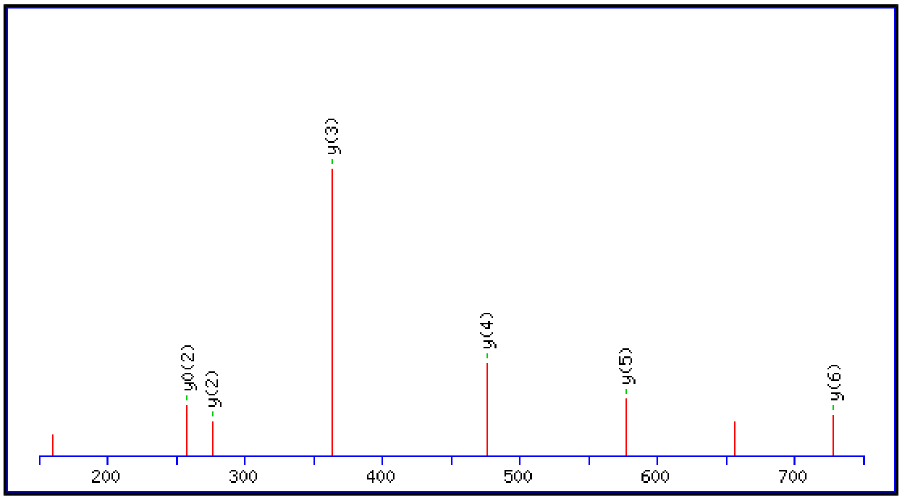

Monoisotopic mass of neutral peptide Mr(calc): 2249.9787
Variable modifications:
C14 : 4Trioxidation (CMWY)
Ions Score: 45 **Expect:** 0.029
Matches : 6/210 fragment ions using 6 most intense peaks (help)

#	b	b^{++}	b*	b^{*++}	b^{0}	b^{0++}	Seq.	y	y^{++}	y*	y^{*++}	y^{0}	y^{0++}	#
1	130.0499	65.5286			112.0393	56.5233	E							19
2	277.1183	139.0628			259.1077	130.0575	F	2121.9434	1061.4753	2104.9168	1052.9620	2103.9328	1052.4700	18
3	391.1612	196.0842	374.1347	187.5710	373.1506	187.0790	N	1974.8750	987.9411	1957.8484	979.4278	1956.8644	978.9358	17
4	462.1983	231.6028	445.1718	223.0895	444.1878	222.5975	A	1860.8320	930.9196	1843.8055	922.4064	1842.8215	921.9144	16
5	591.2409	296.1241	574.2144	287.6108	573.2304	287.1188	E	1789.7949	895.4011	1772.7684	886.8878	1771.7843	886.3958	15
6	692.2886	346.6479	675.2620	338.1347	674.2780	337.6427	T	1660.7523	830.8798	1643.7258	822.3665	1642.7418	821.8745	14
7	839.3570	420.1821	822.3305	411.6689	821.3464	411.1769	F	1559.7046	780.3560	1542.6781	771.8427	1541.6941	771.3507	13
8	940.4047	470.7060	923.3781	462.1927	922.3941	461.7007	T	1412.6362	706.8217	1395.6097	698.3085	1394.6257	697.8165	12
9	1087.4731	544.2402	1070.4466	535.7269	1069.4625	535.2349	F	1311.5885	656.2979	1294.5620	647.7846	1293.5780	647.2926	11
10	1224.5320	612.7696	1207.5055	604.2564	1206.5215	603.7644	H	1164.5201	582.7637	1147.4936	574.2504	1146.5096	573.7584	10
11	1295.5691	648.2882	1278.5426	639.7749	1277.5586	639.2829	A	1027.4612	514.2342	1010.4347	505.7210	1009.4507	505.2290	9
12	1410.5961	705.8017	1393.5695	697.2884	1392.5855	696.7964	D	956.4241	478.7157	939.3976	470.2024	938.4135	469.7104	8
13	1523.6801	762.3437	1506.6536	753.8304	1505.6696	753.3384	I	841.3972	421.2022	824.3706	412.6889	823.3866	412.1969	7
14	1674.6741	837.8407	1657.6475	829.3274	1656.6635	828.8354	C	***728.3131***	364.6602	711.2865	356.1469	710.3025	355.6549	6
15	1775.7217	888.3645	1758.6952	879.8512	1757.7112	879.3592	T	***577.3192***	289.1632	560.2926	280.6499	559.3086	280.1579	5
16	1888.8058	944.9065	1871.7793	936.3933	1870.7952	935.9013	L	***476.2715***	238.6394	459.2449	230.1261	458.2609	229.6341	4
17	1975.8378	988.4226	1958.8113	979.9093	1957.8273	979.4173	S	***363.1874***	182.0974	346.1609	173.5841	345.1769	173.0921	3
18	2104.8804	1052.9439	2087.8539	1044.4306	2086.8699	1043.9386	E	***276.1554***	138.5813	259.1288	130.0681	258.1448	129.5761	2
19							K	147.1128	74.0600	130.0863	65.5468			1

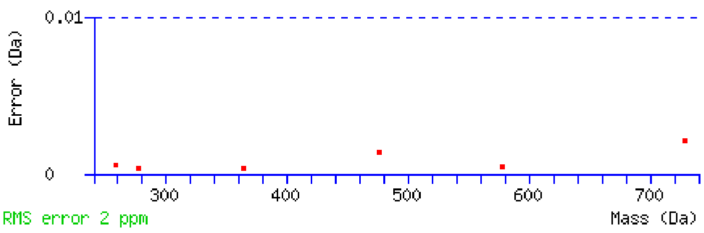

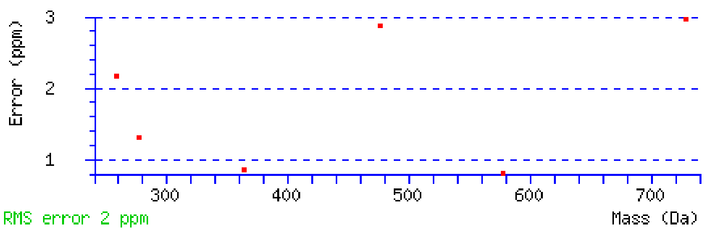

NCBI **BLAST** search of EFNAETFTFHADICTLSEK
(Parameters: blastp, nr protein database, expect=20000, no filter, PAM30)
Other BLAST web gateways

All matches to this query

Score	Mr(calc):	Delta	Sequence
44.9	2249.9787	-0.0045	EFNAETFTFHADICTLSEK
20.7	2248.9641	1.0101	SSTSGATSFGSNEEISECLSEK
6.2	2249.9755	-0.0013	CHADPCPNIVDCFISKPSEK
6.2	2249.9755	-0.0013	CHADPCPNIVDCFISKPSEK
6.2	2249.9755	-0.0013	CHADPCPNIVDCFISKPSEK
6.2	2247.9631	2.0112	DRGMVQVQCPQALRCGSTR
6.2	2248.9517	1.0225	XENMYYSQSSMFPHRSEK
6.2	2248.9754	0.9989	MSDTSESGAGLTRFQAEASEK
6.2	2248.9525	1.0218	NPTRSGSCSMAPWTHCGSRT
6.2	2247.9677	2.0065	XENMYYSQSSMFPHRSEK

Mascot: http://www.matrixscience.com/

MATRIX SCIENCE Mascot Search Results

Peptide View

MS/MS Fragmentation of **VFDEFKPLVEEPQNLIKQNCELFEQLGEYK**
Found in **sp|P02768|ALBU_HUMAN**, Serum albumin OS=Homo sapiens GN=ALB PE=1 SV=2

Match to Query 12995: 3673.792002 from(1225.604610,3+) intensity(69667.1328) scans(15639) rtinseconds(3143) index(12104)
Title: 150808_TTSH_Patient_Plasma_39_Spectrum026247_scans__15639_RTINSECONDS=3143
Data file L:\\Ard_TTSH\\T1D\\T150808_TTSH_Patient_Plasma_39.mgf

Click mouse within plot area to zoom in by factor of two about that point
Or, Plot from 200 to 3600 Da Full range
Label all possible matches ○ Label matches used for scoring ⊙

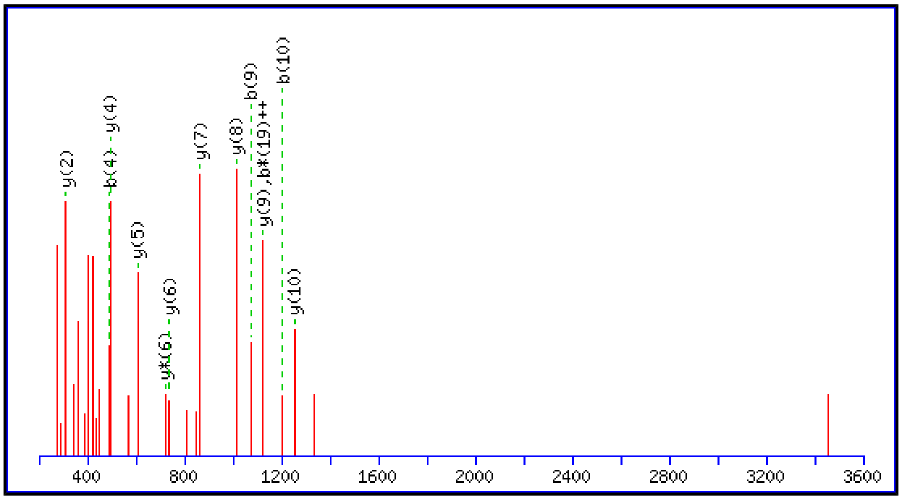

Monoisotopic mass of neutral peptide Mr(calc): 3673.7861
Variable modifications:
C20 : 4Trioxidation (CMWY)
Ions Score: 44 **Expect:** 0.022
Matches : 13/330 fragment ions using 19 most intense peaks (help)

#	b	b^{++}	b*	b^{*++}	b^0	b^{0++}	Seq.	y	y^{++}	y*	y^{*++}	y^0	y^{0++}	#
1	100.0757	50.5415					V							30
2	247.1441	124.0757					F	3575.7250	1788.3662	3558.6985	1779.8529	3557.7145	1779.3609	29
3	362.1710	181.5892			344.1605	172.5839	D	3428.6566	1714.8320	3411.6301	1706.3187	3410.6461	1705.8267	28
4	491.2136	246.1105			473.2031	237.1052	E	3313.6297	1657.3185	3296.6031	1648.8052	3295.6191	1648.3132	27
5	638.2821	319.6447			620.2715	310.6394	F	3184.5871	1592.7972	3167.5605	1584.2839	3166.5765	1583.7919	26
6	766.3770	383.6921	749.3505	375.1789	748.3665	374.6869	K	3037.5187	1519.2630	3020.4921	1510.7497	3019.5081	1510.2577	25
7	863.4298	432.2185	846.4032	423.7053	845.4192	423.2132	P	2909.4237	1455.2155	2892.3972	1446.7022	2891.4132	1446.2102	24
8	976.5138	488.7606	959.4873	480.2473	958.5033	479.7553	L	2812.3710	1406.6891	2795.3444	1398.1758	2794.3604	1397.6838	23
9	1075.5823	538.2948	1058.5557	529.7815	1057.5717	529.2895	V	2699.2869	1350.1471	2682.2603	1341.6338	2681.2763	1341.1418	22
10	1204.6249	602.8161	1187.5983	594.3028	1186.6143	593.8108	E	2600.2185	1300.6129	2583.1919	1292.0996	2582.2079	1291.6076	21
11	1333.6674	667.3374	1316.6409	658.8241	1315.6569	658.3321	E	2471.1759	1236.0916	2454.1493	1227.5783	2453.1653	1227.0863	20
12	1430.7202	715.8637	1413.6937	707.3505	1412.7096	706.8585	P	2342.1333	1171.5703	2325.1067	1163.0570	2324.1227	1162.5650	19
13	1558.7788	779.8930	1541.7522	771.3798	1540.7682	770.8877	Q	2245.0805	1123.0439	2228.0540	1114.5306	2227.0700	1114.0386	18
14	1672.8217	836.9145	1655.7952	828.4012	1654.8111	827.9092	N	2117.0219	1059.0146	2099.9954	1050.5013	2099.0114	1050.0093	17
15	1785.9058	893.4565	1768.8792	884.9433	1767.8952	884.4512	L	2002.9790	1001.9931	1985.9525	993.4799	1984.9685	992.9879	16
16	1898.9898	949.9986	1881.9633	941.4853	1880.9793	940.9933	I	1889.8950	945.4511	1872.8684	936.9378	1871.8844	936.4458	15
17	2027.0848	1014.0460	2010.0583	1005.5328	2009.0742	1005.0408	K	1776.8109	888.9091	1759.7843	880.3958	1758.8003	879.9038	14
18	2155.1434	1078.0753	2138.1168	1069.5621	2137.1328	1069.0700	Q	1648.7159	824.8616	1631.6894	816.3483	1630.7054	815.8563	13
19	2269.1863	1135.0968	2252.1598	1126.5835	2251.1757	1126.0915	N	1520.6574	760.8323	1503.6308	752.3190	1502.6468	751.8270	12

**20**	2420.1802	1210.5938	2403.1537	1202.0805	2402.1697	1201.5885	**C**	1406.6144	703.8109	1389.5879	695.2976	1388.6039	694.8056	**11**
**21**	2549.2228	1275.1151	2532.1963	1266.6018	2531.2123	1266.1098	**E**	***1255.6205***	628.3139	1238.5939	619.8006	1237.6099	619.3086	**10**
**22**	2662.3069	1331.6571	2645.2803	1323.1438	2644.2963	1322.6518	**L**	***1126.5779***	563.7926	1109.5514	555.2793	1108.5673	554.7873	**9**
**23**	2809.3753	1405.1913	2792.3488	1396.6780	2791.3647	1396.1860	**F**	***1013.4938***	507.2506	996.4673	498.7373	995.4833	498.2453	**8**
**24**	2938.4179	1469.7126	2921.3914	1461.1993	2920.4073	1460.7073	**E**	***866.4254***	433.7164	849.3989	425.2031	848.4149	424.7111	**7**
**25**	3066.4765	1533.7419	3049.4499	1525.2286	3048.4659	1524.7366	**Q**	***737.3828***	369.1951	720.3563	360.6818	719.3723	360.1898	**6**
**26**	3179.5605	1590.2839	3162.5340	1581.7706	3161.5500	1581.2786	**L**	***609.3243***	305.1658	592.2977	296.6525	591.3137	296.1605	**5**
**27**	3236.5820	1618.7946	3219.5555	1610.2814	3218.5714	1609.7894	**G**	***496.2402***	248.6237	479.2136	240.1105	478.2296	239.6185	**4**
**28**	3365.6246	1683.3159	3348.5981	1674.8027	3347.6140	1674.3107	**E**	439.2187	220.1130	422.1922	211.5997	421.2082	211.1077	**3**
**29**	3528.6879	1764.8476	3511.6614	1756.3343	3510.6774	1755.8423	**Y**	***310.1761***	155.5917	293.1496	147.0784			**2**
**30**							**K**	147.1128	74.0600	130.0863	65.5468			**1**

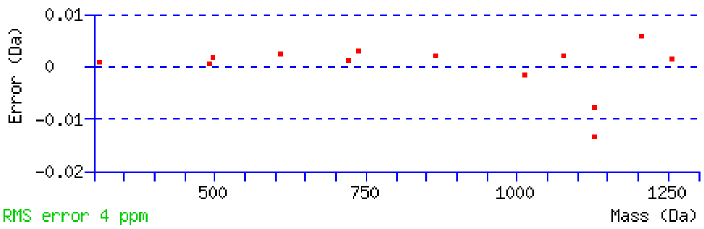

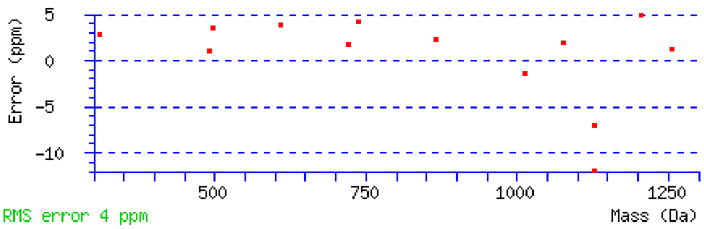

NCBI **BLAST** search of VFDEFKPLVEEPQNLIKQNCELFEQLGEYK
(Parameters: blastp, nr protein database, expect=20000, no filter, PAM30)
Other BLAST web gateways

All matches to this query

Score	Mr(calc):	Delta	Sequence
43.9	3673.7861	0.0059	VFDEFKPLVEEPQNLIKQNCELFEQLGEYK
0.4	3673.7861	0.0059	VFDEFKPLVEEPQNLIKQNCELFEQLGEYK

Mascot: http://www.matrixscience.com/

Mascot Search Results

Peptide View

MS/MS Fragmentation of **SHCIAEVENDEMPADLPSLAADFVESK**
Found in **sp|P02768|ALBU_HUMAN**, Serum albumin OS=Homo sapiens GN=ALB PE=1 SV=2

Match to Query 13203: 3012.266470 from(603.460570,5+) intensity(258869.9219) scans(15562) rtinseconds(3055) index(12459)
Title: 150801_TTSH_Patient_Plasma_76_Spectrum027620_scans__15562_RTINSECONDS=3055
Data file L:\\Ard_TTSH\\T1D\\T150801_TTSH_Patient_Plasma_76.mgf

Click mouse within plot area to zoom in by factor of two about that point
Or, Plot from 200 to 2400 Da Full range
Label all possible matches ○ Label matches used for scoring ⊙

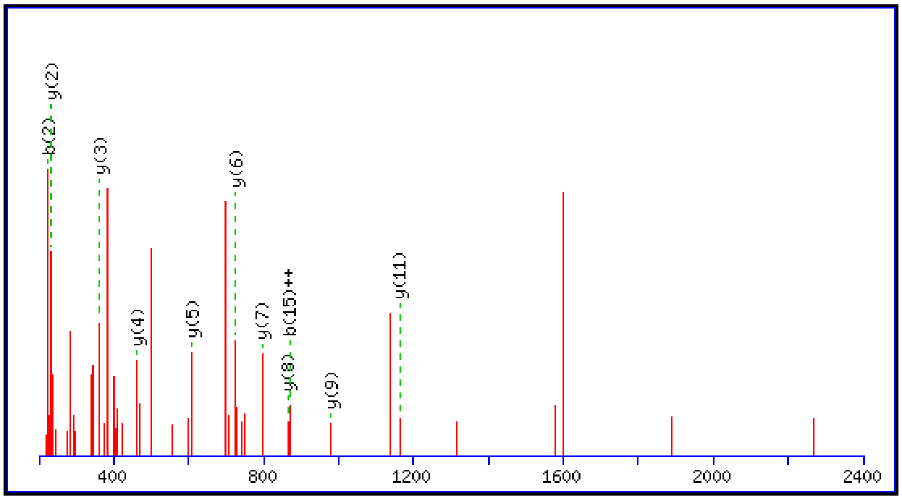

Monoisotopic mass of neutral peptide Mr(calc): 3012.2852
Variable modifications:
C3 : 4Trioxidation (CMWY)
M12 : 4Trioxidation (CMWY)
Ions Score: 44 **Expect:** 0.014
Matches : 11/294 fragment ions using 22 most intense peaks (help)

#	b	b^{++}	b*	b*$^{++}$	b^{0}	b^{0++}	Seq.	y	y^{++}	y*	y*$^{++}$	y^{0}	y^{0++}	#
1	88.0393	44.5233			70.0287	35.5180	S							27
2	**225.0982**	113.0527			207.0877	104.0475	H	2926.2605	1463.6339	2909.2339	1455.1206	2908.2499	1454.6286	26
3	376.0921	188.5497			358.0816	179.5444	C	2789.2016	1395.1044	2772.1750	1386.5911	2771.1910	1386.0991	25
4	489.1762	245.0917			471.1656	236.0865	I	2638.2076	1319.6075	2621.1811	1311.0942	2620.1971	1310.6022	24
5	560.2133	280.6103			542.2028	271.6050	A	2525.1236	1263.0654	2508.0970	1254.5521	2507.1130	1254.0601	23
6	689.2559	345.1316			671.2454	336.1263	E	2454.0865	1227.5469	2437.0599	1219.0336	2436.0759	1218.5416	22
7	788.3243	394.6658			770.3138	385.6605	V	2325.0439	1163.0256	2308.0173	1154.5123	2307.0333	1154.0203	21
8	917.3669	459.1871			899.3564	450.1818	E	2225.9754	1113.4914	2208.9489	1104.9781	2207.9649	1104.4861	20
9	1031.4099	516.2086	1014.3833	507.6953	1013.3993	507.2033	N	2096.9329	1048.9701	2079.9063	1040.4568	2078.9223	1039.9648	19
10	1146.4368	573.7220	1129.4102	565.2088	1128.4262	564.7168	D	1982.8899	991.9486	1965.8634	983.4353	1964.8794	982.9433	18
11	1275.4794	638.2433	1258.4528	629.7301	1257.4688	629.2380	E	1867.8630	934.4351	1850.8364	925.9219	1849.8524	925.4298	17
12	1454.5046	727.7559	1437.4781	719.2427	1436.4941	718.7507	M	1738.8204	869.9138	1721.7938	861.4006	1720.8098	860.9086	16
13	1551.5574	776.2823	1534.5308	767.7691	1533.5468	767.2770	P	1559.7952	780.4012	1542.7686	771.8879	1541.7846	771.3959	15
14	1622.5945	811.8009	1605.5679	803.2876	1604.5839	802.7956	A	1462.7424	731.8748	1445.7159	723.3616	1444.7318	722.8696	14
15	1737.6214	**869.3144**	1720.5949	860.8011	1719.6109	860.3091	D	1391.7053	696.3563	1374.6787	687.8430	1373.6947	687.3510	13
16	1850.7055	925.8564	1833.6790	917.3431	1832.6949	916.8511	L	1276.6783	638.8428	1259.6518	630.3295	1258.6678	629.8375	12
17	1947.7583	974.3828	1930.7317	965.8695	1929.7477	965.3775	P	***1163.5943***	582.3008	1146.5677	573.7875	1145.5837	573.2955	11
18	2034.7903	1017.8988	2017.7637	1009.3855	2016.7797	1008.8935	S	1066.5415	533.7744	1049.5150	525.2611	1048.5310	524.7691	10
19	2147.8744	1074.4408	2130.8478	1065.9275	2129.8638	1065.4355	L	***979.5095***	490.2584	962.4829	481.7451	961.4989	481.2531	9

**20**	2218.9115	1109.9594	2201.8849	1101.4461	2200.9009	1100.9541	**A**	***866.4254***	433.7164	849.3989	425.2031	848.4149	424.7111	**8**
**21**	2289.9486	1145.4779	2272.9220	1136.9647	2271.9380	1136.4726	**A**	***795.3883***	398.1978	778.3618	389.6845	777.3777	389.1925	**7**
**22**	2404.9755	1202.9914	2387.9490	1194.4781	2386.9650	1193.9861	**D**	***724.3512***	362.6792	707.3246	354.1660	706.3406	353.6740	**6**
**23**	2552.0439	1276.5256	2535.0174	1268.0123	2534.0334	1267.5203	**F**	***609.3243***	305.1658	592.2977	296.6525	591.3137	296.1605	**5**
**24**	2651.1124	1326.0598	2634.0858	1317.5465	2633.1018	1317.0545	**V**	***462.2558***	231.6316	445.2293	223.1183	444.2453	222.6263	**4**
**25**	2780.1549	1390.5811	2763.1284	1382.0678	2762.1444	1381.5758	**E**	***363.1874***	182.0974	346.1609	173.5841	345.1769	173.0921	**3**
**26**	2867.1870	1434.0971	2850.1604	1425.5839	2849.1764	1425.0918	**S**	***234.1448***	117.5761	217.1183	109.0628	216.1343	108.5708	**2**
**27**							**K**	147.1128	74.0600	130.0863	65.5468			**1**

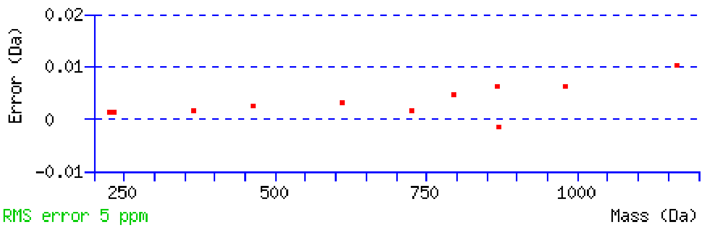

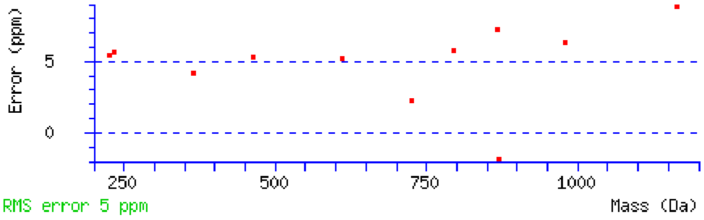

NCBI **BLAST** search of SHCIAEVENDEMPADLPSLAADFVESK
(Parameters: blastp, nr protein database, expect=20000, no filter, PAM30)
Other BLAST web gateways

All matches to this query

Score	Mr(calc):	Delta	Sequence
43.6	3012.2852	-0.0187	SHCIAEVENDEMPADLPSLAADFVESK
0.1	3010.2623	2.0041	XLEDLDGFEGYSLSDWLCLAFVESK
0.1	3010.2623	2.0041	XLEDLDGFEGYSLSDWLCLAFVESK
0.1	3010.2623	2.0041	XLEDLDGFEGYSLSDWLCLAFVESK
0.1	3010.2623	2.0041	XLEDLDGFEGYSLSDWLCLAFVESK

Mascot: http://www.matrixscience.com/

Mascot Search Results

Peptide View

MS/MS Fragmentation of **YICENQDSISSKLK**
Found in **sp|P02768|ALBU_HUMAN**, Serum albumin OS=Homo sapiens GN=ALB PE=1 SV=2

Match to Query 6967: 1674.770848 from(838.392700,2+) intensity(287787.3750) scans(7774) rtinseconds(1451) index(6162)
Title: 150808_TTSH_Patient_Plasma_59_Spectrum022968_scans__7774_RTINSECONDS=1451
Data file L:\\Ard_TTSH\\T1D\\T150808_TTSH_Patient_Plasma_59.mgf

Click mouse within plot area to zoom in by factor of two about that point
Or, Plot from 100 to 1400 Da Full range
Label all possible matches ○ Label matches used for scoring ⦿

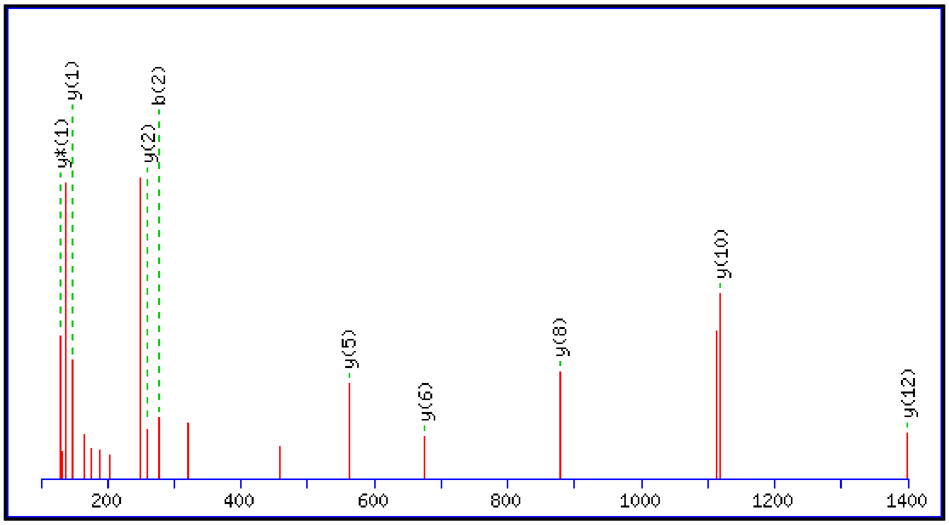

Monoisotopic mass of neutral peptide Mr(calc): 1674.7770
Variable modifications:
C3 : 4Trioxidation (CMWY)
Ions Score: 43 **Expect:** 0.018
Matches : 9/136 fragment ions using 16 most intense peaks (help)

#	b	b^{++}	b*	b^{*++}	b^{0}	b^{0++}	Seq.	y	y^{++}	y*	y^{*++}	y^{0}	y^{0++}	#
1	164.0706	82.5389					Y							14
2	**277.1547**	139.0810					I	1512.7210	756.8641	1495.6945	748.3509	1494.7104	747.8589	13
3	428.1486	214.5779					C	***1399.6369***	700.3221	1382.6104	691.8088	1381.6264	691.3168	12
4	557.1912	279.0992			539.1806	270.0940	E	1248.6430	624.8251	1231.6165	616.3119	1230.6325	615.8199	11
5	671.2341	336.1207	654.2076	327.6074	653.2236	327.1154	N	***1119.6004***	560.3039	1102.5739	551.7906	1101.5899	551.2986	10
6	799.2927	400.1500	782.2661	391.6367	781.2821	391.1447	Q	1005.5575	503.2824	988.5310	494.7691	987.5469	494.2771	9
7	914.3196	457.6635	897.2931	449.1502	896.3091	448.6582	D	***877.4989***	439.2531	860.4724	430.7398	859.4884	430.2478	8
8	1001.3517	501.1795	984.3251	492.6662	983.3411	492.1742	S	762.4720	381.7396	745.4454	373.2264	744.4614	372.7343	7
9	1114.4357	557.7215	1097.4092	549.2082	1096.4252	548.7162	I	***675.4400***	338.2236	658.4134	329.7103	657.4294	329.2183	6
10	1201.4678	601.2375	1184.4412	592.7242	1183.4572	592.2322	S	***562.3559***	281.6816	545.3293	273.1683	544.3453	272.6763	5
11	1288.4998	644.7535	1271.4732	636.2403	1270.4892	635.7482	S	475.3239	238.1656	458.2973	229.6523	457.3133	229.1603	4
12	1416.5948	708.8010	1399.5682	700.2877	1398.5842	699.7957	K	388.2918	194.6496	371.2653	186.1363			3
13	1529.6788	765.3430	1512.6523	756.8298	1511.6682	756.3378	L	***260.1969***	130.6021	243.1703	122.0888			2
14							K	***147.1128***	74.0600	130.0863	65.5468			1

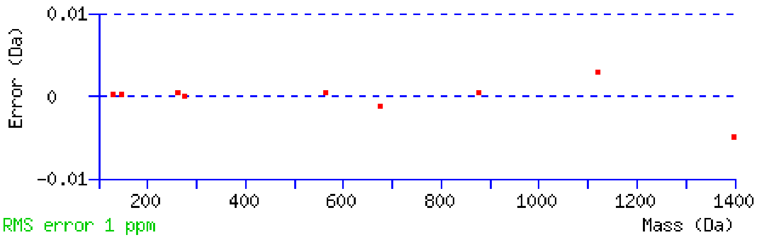

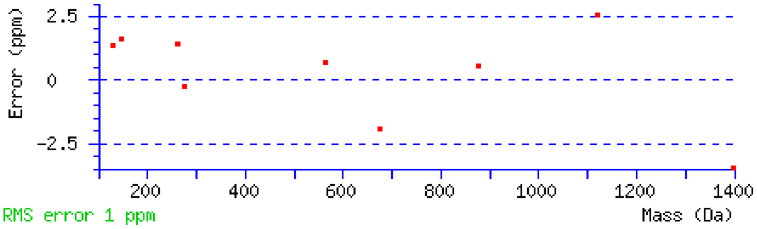

NCBI **BLAST** search of YICENQDSISSKLK
(Parameters: blastp, nr protein database, expect=20000, no filter, PAM30)
Other BLAST web gateways

All matches to this query

Score	Mr(calc):	Delta	Sequence
43.0	1674.7770	-0.0062	YICENQDSISSKLK
28.3	1674.7770	-0.0062	YICENQDSISSKLK

**Mascot:** http://www.matrixscience.com/

Mascot Search Results

Peptide View

MS/MS Fragmentation of **LKECCEKPLLEKSHCIAEVENDEMPADLPSLAADFVESK**
Found in **sp|P02768|ALBU_HUMAN**, Serum albumin OS=Homo sapiens GN=ALB PE=1 SV=2

Match to Query 15813: 4521.998736 from(1131.506960,4+) intensity(631575.0000) scans(15961) rtinseconds(2876) index(13474)
Title: 150808_TTSH_Patient_Plasma_60_Spectrum030164_scans__15961_RTINSECONDS=2876
Data file L:\\Ard_TTSH\\T1D\\T150808_TTSH_Patient_Plasma_60.mgf

Click mouse within plot area to zoom in by factor of two about that point
Or, Plot from 200 to 3000 Da Full range
Label all possible matches ○ Label matches used for scoring ⊙

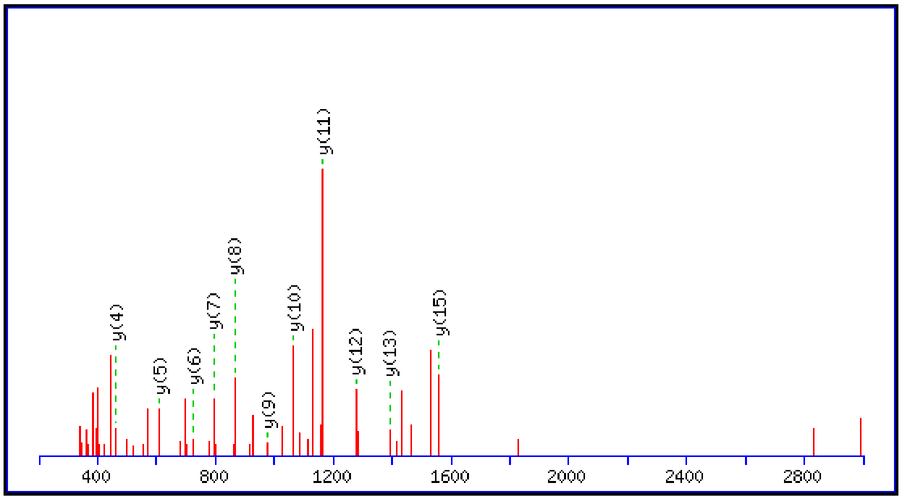

Monoisotopic mass of neutral peptide Mr(calc): 4521.9906
Variable modifications:
C4 : 4Trioxidation (CMWY)
C5 : 4Trioxidation (CMWY)
C15 : 4Trioxidation (CMWY)
M24 : 4Trioxidation (CMWY)
Ions Score: 42 **Expect:** 0.037
Matches : 11/448 fragment ions using 29 most intense peaks (help)

#	b	b^{++}	b*	b^{*++}	b^{0}	b^{0++}	Seq.	y	y^{++}	y*	y^{*++}	y^{0}	y^{0++}	#
1	114.0913	57.5493					L							39
2	242.1863	121.5968	225.1598	113.0835			K	4409.9139	2205.4606	4392.8874	2196.9473	4391.9034	2196.4553	38
3	371.2289	186.1181	354.2023	177.6048	353.2183	177.1128	E	4281.8190	2141.4131	4264.7924	2132.8998	4263.8084	2132.4078	37
4	522.2228	261.6151	505.1963	253.1018	504.2123	252.6098	C	4152.7764	2076.8918	4135.7498	2068.3785	4134.7658	2067.8865	36
5	673.2168	337.1120	656.1902	328.5987	655.2062	328.1067	C	4001.7824	2001.3949	3984.7559	1992.8816	3983.7719	1992.3896	35
6	802.2593	401.6333	785.2328	393.1200	784.2488	392.6280	E	3850.7885	1925.8979	3833.7620	1917.3846	3832.7779	1916.8926	34
7	930.3543	465.6808	913.3278	457.1675	912.3437	456.6755	K	3721.7459	1861.3766	3704.7194	1852.8633	3703.7353	1852.3713	33
8	1027.4071	514.2072	1010.3805	505.6939	1009.3965	505.2019	P	3593.6510	1797.3291	3576.6244	1788.8158	3575.6404	1788.3238	32
9	1140.4911	570.7492	1123.4646	562.2359	1122.4806	561.7439	L	3496.5982	1748.8027	3479.5716	1740.2895	3478.5876	1739.7974	31
10	1253.5752	627.2912	1236.5487	618.7780	1235.5646	618.2860	L	3383.5141	1692.2607	3366.4876	1683.7474	3365.5036	1683.2554	30
11	1382.6178	691.8125	1365.5912	683.2993	1364.6072	682.8073	E	3270.4301	1635.7187	3253.4035	1627.2054	3252.4195	1626.7134	29
12	1510.7128	755.8600	1493.6862	747.3467	1492.7022	746.8547	K	3141.3875	1571.1974	3124.3609	1562.6841	3123.3769	1562.1921	28
13	1597.7448	799.3760	1580.7182	790.8628	1579.7342	790.3707	S	3013.2925	1507.1499	2996.2660	1498.6366	2995.2819	1498.1446	27
14	1734.8037	867.9055	1717.7771	859.3922	1716.7931	858.9002	H	2926.2605	1463.6339	2909.2339	1455.1206	2908.2499	1454.6286	26
15	1885.7976	943.4025	1868.7711	934.8892	1867.7871	934.3972	C	2789.2016	1395.1044	2772.1750	1386.5911	2771.1910	1386.0991	25
16	1998.8817	999.9445	1981.8551	991.4312	1980.8711	990.9392	I	2638.2076	1319.6075	2621.1811	1311.0942	2620.1971	1310.6022	24
17	2069.9188	1035.4630	2052.8923	1026.9498	2051.9082	1026.4578	A	2525.1236	1263.0654	2508.0970	1254.5521	2507.1130	1254.0601	23

**18**	2198.9614	1099.9843	2181.9348	1091.4711	2180.9508	1090.9791	**E**	2454.0865	1227.5469	2437.0599	1219.0336	2436.0759	1218.5416	**22**
**19**	2298.0298	1149.5185	2281.0033	1141.0053	2280.0192	1140.5133	**V**	2325.0439	1163.0256	2308.0173	1154.5123	2307.0333	1154.0203	**21**
**20**	2427.0724	1214.0398	2410.0459	1205.5266	2409.0618	1205.0346	**E**	2225.9754	1113.4914	2208.9489	1104.9781	2207.9649	1104.4861	**20**
**21**	2541.1153	1271.0613	2524.0888	1262.5480	2523.1048	1262.0560	**N**	2096.9329	1048.9701	2079.9063	1040.4568	2078.9223	1039.9648	**19**
**22**	2656.1423	1328.5748	2639.1157	1320.0615	2638.1317	1319.5695	**D**	1982.8899	991.9486	1965.8634	983.4353	1964.8794	982.9433	**18**
**23**	2785.1849	1393.0961	2768.1583	1384.5828	2767.1743	1384.0908	**E**	1867.8630	934.4351	1850.8364	925.9219	1849.8524	925.4298	**17**
**24**	2964.2101	1482.6087	2947.1835	1474.0954	2946.1995	1473.6034	**M**	1738.8204	869.9138	1721.7938	861.4006	1720.8098	860.9086	**16**
**25**	3061.2629	1531.1351	3044.2363	1522.6218	3043.2523	1522.1298	**P**	***1559.7952***	780.4012	1542.7686	771.8879	1541.7846	771.3959	**15**
**26**	3132.3000	1566.6536	3115.2734	1558.1404	3114.2894	1557.6483	**A**	1462.7424	731.8748	1445.7159	723.3616	1444.7318	722.8696	**14**
**27**	3247.3269	1624.1671	3230.3004	1615.6538	3229.3164	1615.1618	**D**	***1391.7053***	696.3563	1374.6787	687.8430	1373.6947	687.3510	**13**
**28**	3360.4110	1680.7091	3343.3844	1672.1959	3342.4004	1671.7038	**L**	***1276.6783***	638.8428	1259.6518	630.3295	1258.6678	629.8375	**12**
**29**	3457.4637	1729.2355	3440.4372	1720.7222	3439.4532	1720.2302	**P**	***1163.5943***	582.3008	1146.5677	573.7875	1145.5837	573.2955	**11**
**30**	3544.4958	1772.7515	3527.4692	1764.2383	3526.4852	1763.7462	**S**	***1066.5415***	533.7744	1049.5150	525.2611	1048.5310	524.7691	**10**
**31**	3657.5798	1829.2936	3640.5533	1820.7803	3639.5693	1820.2883	**L**	***979.5095***	490.2584	962.4829	481.7451	961.4989	481.2531	**9**
**32**	3728.6170	1864.8121	3711.5904	1856.2988	3710.6064	1855.8068	**A**	***866.4254***	433.7163	849.3989	425.2031	848.4149	424.7111	**8**
**33**	3799.6541	1900.3307	3782.6275	1891.8174	3781.6435	1891.3254	**A**	***795.3883***	398.1978	778.3618	389.6845	777.3777	389.1925	**7**
**34**	3914.6810	1957.8441	3897.6545	1949.3309	3896.6704	1948.8389	**D**	***724.3512***	362.6792	707.3246	354.1660	706.3406	353.6740	**6**
**35**	4061.7494	2031.3783	4044.7229	2022.8651	4043.7389	2022.3731	**F**	***609.3243***	305.1658	592.2977	296.6525	591.3137	296.1605	**5**
**36**	4160.8178	2080.9126	4143.7913	2072.3993	4142.8073	2071.9073	**V**	***462.2558***	231.6316	445.2293	223.1183	444.2453	222.6263	**4**
**37**	4289.8604	2145.4339	4272.8339	2136.9206	4271.8499	2136.4286	**E**	363.1874	182.0974	346.1609	173.5841	345.1769	173.0921	**3**
**38**	4376.8925	2188.9499	4359.8659	2180.4366	4358.8819	2179.9446	**S**	234.1448	117.5761	217.1183	109.0628	216.1343	108.5708	**2**
**39**							**K**	147.1128	74.0600	130.0863	65.5468			**1**

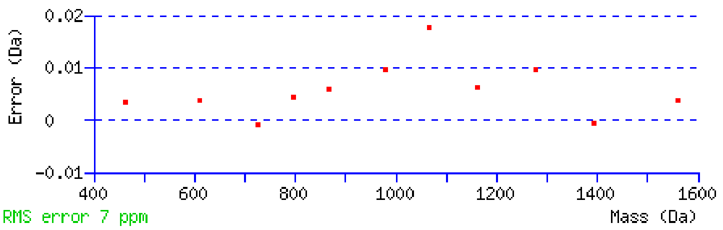

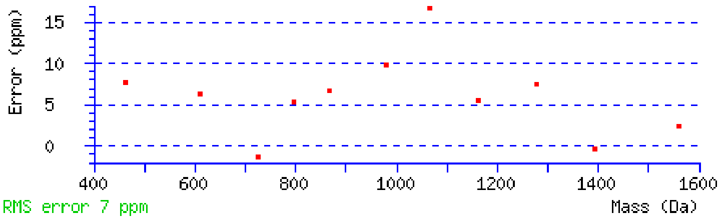

NCBI **BLAST** search of LKECCEKPLLEKSHCIAEVENDEMPADLPSLAADFVESK
(Parameters: blastp, nr protein database, expect=20000, no filter, PAM30)
Other BLAST web gateways

All matches to this query

Score	Mr(calc):	Delta	Sequence
42.0	4521.9906	0.0081	LKECCEKPLLEKSHCIAEVENDEMPADLPSLAADFVESK

Mascot: http://www.matrixscience.com/

Mascot Search Results

Peptide View

MS/MS Fragmentation of **CASLQKFGER**
Found in **sp|P02768|ALBU_HUMAN**, Serum albumin OS=Homo sapiens GN=ALB PE=1 SV=2

Match to Query 2198: 1185.543928 from(593.779240,2+) intensity(666292.0625) scans(6344) rtinseconds(1191) index(5218)
Title: 150808_TTSH_Patient_Plasma_61_Spectrum022177_scans__6344_RTINSECONDS=1191
Data file L:\\Ard_TTSH\\T1D\\T150808_TTSH_Patient_Plasma_61.mgf

Click mouse within plot area to zoom in by factor of two about that point
Or, Plot from 0 to 1200 Da Full range
Label all possible matches ○ Label matches used for scoring ⊙

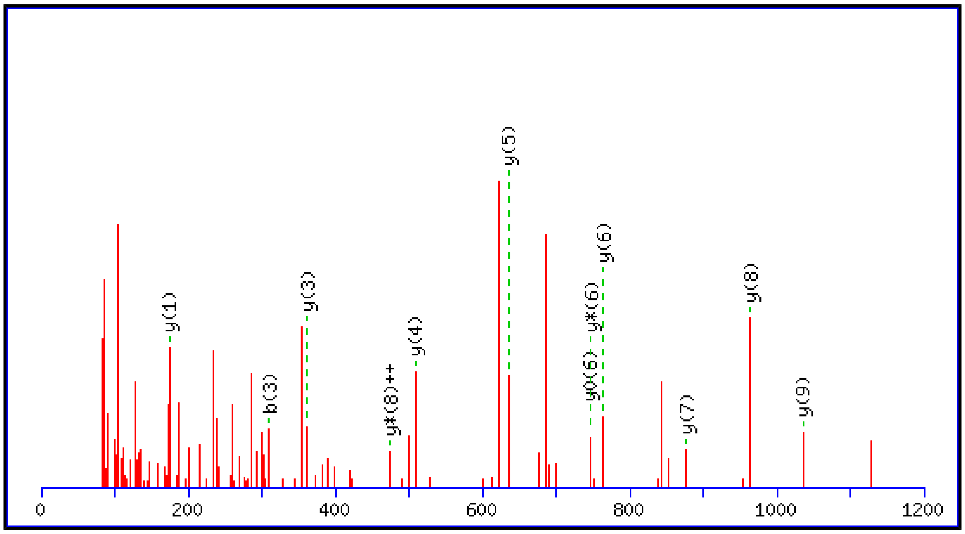


```
Monoisotopic mass of neutral peptide Mr(calc): 1185.5448
Variable modifications:
C1     : 4Trioxidation (CMWY)
Ions Score: 36  Expect: 0.034
Matches : 12/94 fragment ions using 36 most intense peaks   (help)
```

#	b	b^{++}	b*	b^{*++}	b^{0}	b^{0++}	Seq.	y	y^{++}	y*	y^{*++}	y^{0}	y^{0++}	#
1	152.0012	76.5042					C							10
2	223.0383	112.0228					A	***1035.5582***	518.2827	1018.5316	509.7694	1017.5476	509.2774	9
3	**310.0703**	155.5388			292.0598	146.5335	S	***964.5211***	482.7642	947.4945	474.2509	946.5105	473.7589	8
4	423.1544	212.0808			405.1438	203.0756	L	***877.4890***	439.2482	860.4625	430.7349	859.4785	430.2429	7
5	551.2130	276.1101	534.1864	267.5969	533.2024	267.1049	Q	***764.4050***	382.7061	747.3784	374.1928	746.3944	373.7008	6
6	679.3080	340.1576	662.2814	331.6443	661.2974	331.1523	K	***636.3464***	318.6768	619.3198	310.1636	618.3358	309.6715	5
7	826.3764	413.6918	809.3498	405.1785	808.3658	404.6865	F	***508.2514***	254.6293	491.2249	246.1161	490.2409	245.6241	4
8	883.3978	442.2026	866.3713	433.6893	865.3873	433.1973	G	***361.1830***	181.0951	344.1565	172.5819	343.1724	172.0899	3
9	1012.4404	506.7238	995.4139	498.2106	994.4299	497.7186	E	304.1615	152.5844	287.1350	144.0711	286.1510	143.5791	2
10							R	***175.1190***	88.0631	158.0924	79.5498			1

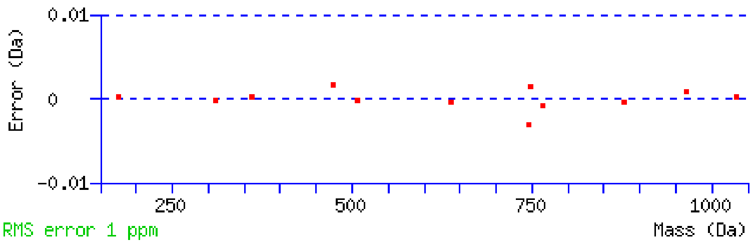

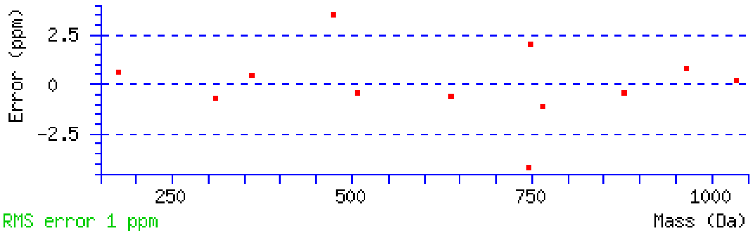

NCBI **BLAST** search of CASLQKFGER
(Parameters: blastp, nr protein database, expect=20000, no filter, PAM30)
Other BLAST web gateways

All matches to this query

Score	Mr(calc):	Delta	Sequence
36.3	1185.5448	-0.0009	CASLQKFGER

**Mascot:** http://www.matrixscience.com/
