# Supplementary material for: Serum albumin cysteine trioxidation is a potential oxidative stress biomarker of type 2 diabetes mellitus
Source: Sci Rep. 2020 Apr 15;10:6475. doi: 10.1038/s41598-020-62341-z (PMC7160123; doi:10.1038/s41598-020-62341-z)
Supplement: Supplementary file 3 — Supplementary Information 3. [file 41598_2020_62341_MOESM3_ESM.pdf]

Serum albumin cysteine trioxidation is a potential oxidative stress biomarker of type 2 diabetes mellitus

Selvam Paramasivan, Sunil S Adav, SoFong Cam Ngan, Rinkoo Dalan, Melvin Khee-Shing Leow, Hee Hwa Ho, Siu Kwan Sze*

ALB_K.ALVLIAFAQYLQQC#PFEDHVK.L_103.07
ALB_K.ALVLIAFAQYLQQC#PFEDHVK.L_101.62
ALB_K.ALVLIAFAQYLQQC#PFEDHVK.L_95.42
ALB_K.ALVLIAFAQYLQQC#PFEDHVK.L_95.14
ALB_K.ALVLIAFAQYLQQC#PFEDHVK.L_94.99
ALB_K.ALVLIAFAQYLQQC#PFEDHVK.L_93.03
ALB_K.ALVLIAFAQYLQQC#PFEDHVK.L_86.66
ALB_K.ALVLIAFAQYLQQC#PFEDHVK.L_82.14
ALB_K.ALVLIAFAQYLQQC#PFEDHVK.L_78.88
ALB_K.ALVLIAFAQYLQQC#PFEDHVK.L_78.80
ALB_K.ALVLIAFAQYLQQC#PFEDHVK.L_74.73
ALB_K.ALVLIAFAQYLQQC#PFEDHVK.L_74.13
ALB_K.ALVLIAFAQYLQQC#PFEDHVK.L_68.08
ALB_K.ALVLIAFAQYLQQC#PFEDHVK.L_67.30
ALB_K.ALVLIAFAQYLQQC#PFEDHVK.L_66.52
ALB_K.ALVLIAFAQYLQQC#PFEDHVK.L_65.28
ALB_K.ALVLIAFAQYLQQC#PFEDHVK.L_64.65
ALB_K.ALVLIAFAQYLQQC#PFEDHVK.L_64.14
ALB_K.ALVLIAFAQYLQQC#PFEDHVK.L_63.15
ALB_K.ALVLIAFAQYLQQC#PFEDHVK.L_62.71
ALB_K.ALVLIAFAQYLQQC#PFEDHVK.L_132.63
ALB_K.ALVLIAFAQYLQQC#PFEDHVK.L_132.62
ALB_K.ALVLIAFAQYLQQC#PFEDHVK.L_130.40
ALB_K.ALVLIAFAQYLQQC#PFEDHVK.L_127.52
ALB_K.ALVLIAFAQYLQQC#PFEDHVK.L_123.28
ALB_K.ALVLIAFAQYLQQC#PFEDHVK.L_120.50
ALB_K.ALVLIAFAQYLQQC#PFEDHVK.L_120.25
ALB_K.ALVLIAFAQYLQQC#PFEDHVK.L_120.19
ALB_K.ALVLIAFAQYLQQC#PFEDHVK.L_120.19
ALB_K.ALVLIAFAQYLQQC#PFEDHVK.L_120.17
ALB_K.ALVLIAFAQYLQQC#PFEDHVK.L_119.99
ALB_K.ALVLIAFAQYLQQC#PFEDHVK.L_119.98
ALB_K.ALVLIAFAQYLQQC#PFEDHVK.L_119.77
ALB_K.ALVLIAFAQYLQQC#PFEDHVK.L_119.56
ALB_K.ALVLIAFAQYLQQC#PFEDHVK.L_117.84
ALB_K.ALVLIAFAQYLQQC#PFEDHVK.L_117.81
ALB_K.ALVLIAFAQYLQQC#PFEDHVK.L_117.74
ALB_K.ALVLIAFAQYLQQC#PFEDHVK.L_117.73
ALB_K.ALVLIAFAQYLQQC#PFEDHVK.L_117.38
ALB_K.ALVLIAFAQYLQQC#PFEDHVK.L_117.23
ALB_R.RPC#FSALEVDETYVPK.E_92.53
ALB_R.RPC#FSALEVDETYVPK.E_90.06
ALB_R.RPC#FSALEVDETYVPK.E_86.40
ALB_R.RPC#FSALEVDETYVPK.E_84.39
ALB_R.RPC#FSALEVDETYVPK.E_82.43
ALB_R.RPC#FSALEVDETYVPK.E_81.58
ALB_R.RPC#FSALEVDETYVPK.E_81.28
ALB_R.RPC#FSALEVDETYVPK.E_81.26
ALB_R.RPC#FSALEVDETYVPK.E_78.93
ALB_R.RPC#FSALEVDETYVPK.E_78.79
ALB_R.RPC#FSALEVDETYVPK.E_77.56
ALB_R.RPC#FSALEVDETYVPK.E_76.73
ALB_R.RPC#FSALEVDETYVPK.E_75.68

ALB_R.RPC#FSALEVDETYVPK.E_75.43
ALB_R.RPC#FSALEVDETYVPK.E_74.00
ALB_R.RPC#FSALEVDETYVPK.E_73.41
ALB_R.RPC#FSALEVDETYVPK.E_72.48
ALB_R.RPC#FSALEVDETYVPK.E_72.48
ALB_R.RPC#FSALEVDETYVPK.E_71.85
ALB_R.RPC#FSALEVDETYVPK.E_71.71
ALB_R.RPC#FSALEVDETYVPK.E_93.48
ALB_R.RPC#FSALEVDETYVPK.E_89.47
ALB_R.RPC#FSALEVDETYVPK.E_88.87
ALB_R.RPC#FSALEVDETYVPK.E_88.79
ALB_R.RPC#FSALEVDETYVPK.E_88.65
ALB_R.RPC#FSALEVDETYVPK.E_88.21
ALB_R.RPC#FSALEVDETYVPK.E_87.23
ALB_R.RPC#FSALEVDETYVPK.E_87.15
tr|B2RBS8|B2RBS8_HUMAN_R.RPC#FSALEVDETYVPK.E_86.97
ALB_R.RPC#FSALEVDETYVPK.E_86.89
ALB_R.RPC#FSALEVDETYVPK.E_86.76
ALB_R.RPC#FSALEVDETYVPK.E_86.57
ALB_R.RPC#FSALEVDETYVPK.E_86.42
ALB_R.RPC#FSALEVDETYVPK.E_86.09
ALB_R.RPC#FSALEVDETYVPK.E_86.02
ALB_R.RPC#FSALEVDETYVPK.E_85.83
ALB_R.RPC#FSALEVDETYVPK.E_85.49
ALB_R.RPC#FSALEVDETYVPK.E_85.03
ALB_R.RPC#FSALEVDETYVPK.E_84.70
ALB_R.RPC#FSALEVDETYVPK.E_84.66
ALB_K.YIC#ENQDSISSK.L_75.01
ALB_K.YIC#ENQDSISSK.L_74.57
ALB_K.YIC#ENQDSISSK.L_74.55
ALB_K.YIC#ENQDSISSK.L_71.98
ALB_K.YIC#ENQDSISSK.L_71.81
ALB_K.YIC#ENQDSISSK.L_71.57
ALB_K.YIC#ENQDSISSK.L_71.50
ALB_K.YIC#ENQDSISSK.L_71.22
ALB_K.YIC#ENQDSISSK.L_70.45
ALB_K.YIC#ENQDSISSK.L_70.28
tr|F6KPG5|F6KPG5_HUMAN_K.YIC#ENQDSISSK.L_70.04
ALB_K.YIC#ENQDSISSK.L_69.90
ALB_K.YIC#ENQDSISSK.L_69.43
ALB_K.YIC#ENQDSISSK.L_69.41
ALB_K.YIC#ENQDSISSK.L_68.55
ALB_K.YIC#ENQDSISSK.L_68.36
ALB_K.YIC#ENQDSISSK.L_67.46
ALB_K.YIC#ENQDSISSK.L_67.22
ALB_K.YIC#ENQDSISSK.L_66.71
tr|F6KPG5|F6KPG5_HUMAN_K.YIC#ENQDSISSK.L_66.29
ALB_K.YIC#ENQDSISSK.L_96.98
tr|B2RBS8|B2RBS8_HUMAN_K.Y#IC#ENQDSISSK.L_96.46
ALB_K.YIC#ENQDSISSK.L_94.33
ALB_K.YIC#ENQDSISSK.L_94.01
ALB_K.YIC#ENQDSISSK.L_94.01
tr|B2RBS8|B2RBS8_HUMAN_K.YIC#ENQDSISSK.L_93.96
ALB_K.YIC#ENQDSISSK.L_93.89

ALB_K.YIC#ENQDSISSK.L_93.29
ALB_K.YIC#ENQDSISSK.L_92.38
ALB_K.YIC#ENQDSISSK.L_91.86
ALB_K.YIC#ENQDSISSK.L_91.83
ALB_K.YIC#ENQDSISSK.L_91.70
ALB_K.YIC#ENQDSISSK.L_91.68
ALB_K.YIC#ENQDSISSK.L_91.36
ALB_K.YIC#ENQDSISSK.L_91.20
tr|B2RBS8|B2RBS8_HUMAN_K.YIC#ENQDSISSK.L_90.79
ALB_K.YIC#ENQDSISSK.L_90.70
ALB_K.YIC#ENQDSISSK.L_90.62
ALB_K.YIC#ENQDSISSK.L_90.38
ALB_K.YIC#ENQDSISSK.L_90.27

Mascot Search Results

Peptide View

MS/MS Fragmentation of **ALVLIAFAQYLQQCPFEDHVK**
Found in **sp|P02768|ALBU_HUMAN**, Serum albumin OS=Homo sapiens GN=ALB PE=1 SV=2

Match to Query 12476: 2480.239608 from(1241.127080,2+) intensity(10307705.0000) scans(18076) rtinseconds(3116) index(15668)
Title: 150818_TTSH_Patient_Plasma_37_Spectrum034070_scans__18076_RTINSECONDS=3116
Data file L:\\Ard_TTSH\\T1D\\T150818_TTSH_Patient_Plasma_37.mgf

Click mouse within plot area to zoom in by factor of two about that point

Or, to Da

Label all possible matches ○ Label matches used for scoring ⊙

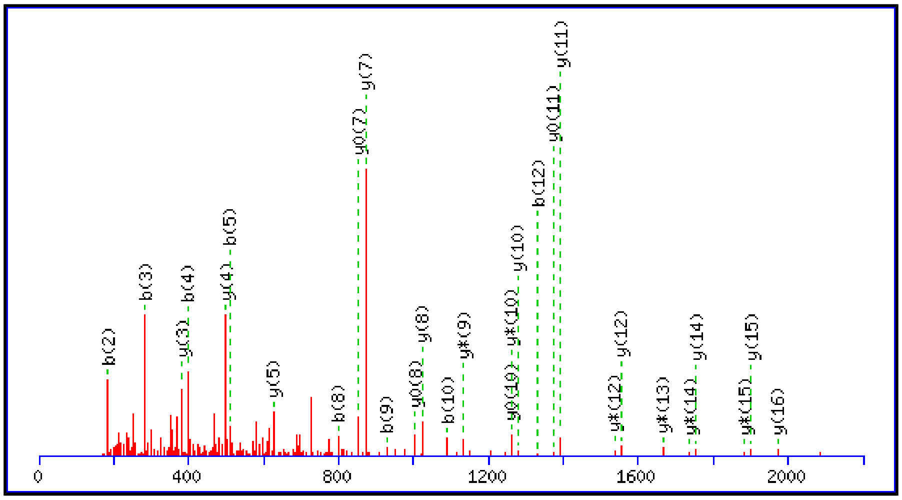

Monoisotopic mass of neutral peptide Mr(calc): 2480.2410
Variable modifications:
C14 : 4Trioxidation (CMWY)
Ions Score: 103 **Expect:** 1.9e-008
Matches : 29/186 fragment ions using 35 most intense peaks (help)

#	b	b^{++}	b*	b^{*++}	b^{0}	b^{0++}	Seq.	y	y^{++}	y*	y^{*++}	y^{0}	y^{0++}	#
1	72.0444	36.5258					A							21
2	***185.1285***	93.0679					L	2410.2111	1205.6092	2393.1846	1197.0959	2392.2006	1196.6039	20
3	***284.1969***	142.6021					V	2297.1271	1149.0672	2280.1005	1140.5539	2279.1165	1140.0619	19
4	***397.2809***	199.1441					L	2198.0587	1099.5330	2181.0321	1091.0197	2180.0481	1090.5277	18
5	***510.3650***	255.6861					I	2084.9746	1042.9909	2067.9481	1034.4777	2066.9640	1033.9857	17
6	581.4021	291.2047					A	***1971.8905***	986.4489	1954.8640	977.9356	1953.8800	977.4436	16
7	728.4705	364.7389					F	***1900.8534***	950.9304	1883.8269	942.4171	1882.8429	941.9251	15
8	***799.5076***	400.2575					A	***1753.7850***	877.3961	1736.7585	868.8829	1735.7744	868.3909	14
9	***927.5662***	464.2867	910.5397	455.7735			Q	1682.7479	841.8776	1665.7214	833.3643	1664.7373	832.8723	13
10	***1090.6295***	545.8184	1073.6030	537.3051			Y	***1554.6893***	777.8483	1537.6628	769.3350	1536.6788	768.8430	12
11	1203.7136	602.3604	1186.6871	593.8472			L	***1391.6260***	696.3166	1374.5994	687.8034	1373.6154	687.3114	11
12	***1331.7722***	666.3897	1314.7456	657.8765			Q	***1278.5419***	639.7746	1261.5154	631.2613	1260.5314	630.7693	10
13	1459.8308	730.4190	1442.8042	721.9057			Q	1150.4834	575.7453	1133.4568	567.2320	1132.4728	566.7400	9
14	1610.8247	805.9160	1593.7981	797.4027			C	***1022.4248***	511.7160	1005.3982	503.2027	1004.4142	502.7107	8
15	1707.8775	854.4424	1690.8509	845.9291			P	***871.4308***	436.2191	854.4043	427.7058	853.4203	427.2138	7
16	1854.9459	927.9766	1837.9193	919.4633			F	774.3781	387.6927	757.3515	379.1794	756.3675	378.6874	6
17	1983.9885	992.4979	1966.9619	983.9846	1965.9779	983.4926	E	***627.3097***	314.1585	610.2831	305.6452	609.2991	305.1532	5
18	2099.0154	1050.0113	2081.9889	1041.4981	2081.0048	1041.0061	D	***498.2671***	249.6372	481.2405	241.1239	480.2565	240.6319	4
19	2236.0743	1118.5408	2219.0478	1110.0275	2218.0638	1109.5355	H	***383.2401***	192.1237	366.2136	183.6104			3

**20**	2335.1427	1168.0750	2318.1162	1159.5617	2317.1322	1159.0697	**V**	246.1812	123.5942	229.1547	115.0810			**2**
**21**							**K**	147.1128	74.0600	130.0863	65.5468			**1**

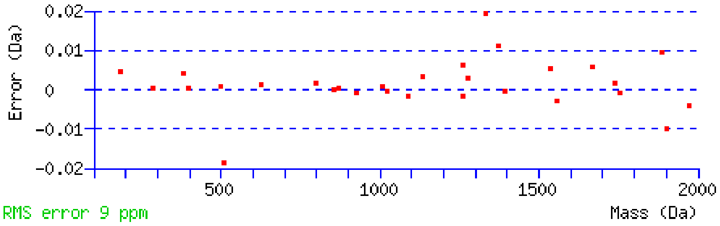

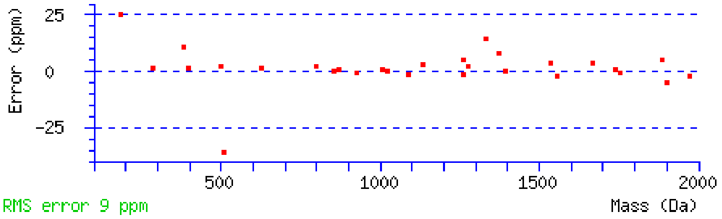

NCBI **BLAST** search of ALVLIAFAQYLQQCPFEDHVK
(Parameters: blastp, nr protein database, expect=20000, no filter, PAM30)
Other BLAST web gateways

All matches to this query

Score	Mr(calc):	Delta	Sequence
103.1	2480.2410	-0.0014	ALVLIAFAQYLQQCPFEDHVK
46.6	2480.2410	-0.0014	ALVLIAFAQYLQQCPFEDHVK

Mascot Search Results

Peptide View

MS/MS Fragmentation of **ALVLIAFAQYLQQCPFEDHVK**
Found in **sp|P02768|ALBU_HUMAN**, Serum albumin OS=Homo sapiens GN=ALB PE=1 SV=2

Match to Query 12094: 2480.241308 from(1241.127930,2+) intensity(3101732.2500) scans(18014) rtinseconds(3112) index(15391)
Title: 150818_TTSH_Patient_Plasma_36_Spectrum033647_scans__18014_RTINSECONDS=3112
Data file L:\\Ard_TTSH\\T1D\\T150818_TTSH_Patient_Plasma_36.mgf

Click mouse within plot area to zoom in by factor of two about that point
Or, Plot from 0 to 2200 Da Full range
Label all possible matches ○ Label matches used for scoring ⊙

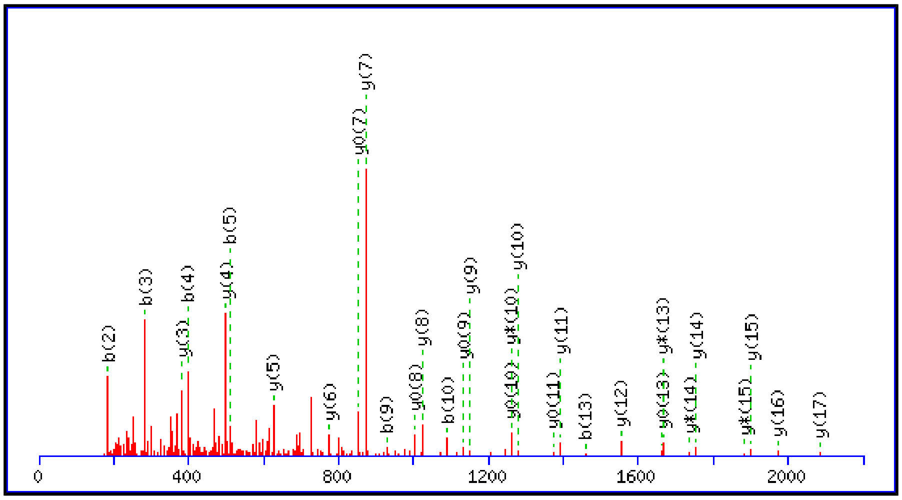

Monoisotopic mass of neutral peptide Mr(calc): 2480.2410
Variable modifications:
C14 : 4Trioxidation (CMWY)
Ions Score: 102 **Expect:** 2.7e-008
Matches : 31/186 fragment ions using 46 most intense peaks (help)

#	b	b^{++}	b*	b^{*++}	b^0	b^{0++}	Seq.	y	y^{++}	y*	y^{*++}	y^0	y^{0++}	#
1	72.0444	36.5258					A							21
2	**185.1285**	93.0679					L	2410.2111	1205.6092	2393.1846	1197.0959	2392.2006	1196.6039	20
3	**284.1969**	142.6021					V	2297.1271	1149.0672	2280.1005	1140.5539	2279.1165	1140.0619	19
4	**397.2809**	199.1441					L	2198.0587	1099.5330	2181.0321	1091.0197	2180.0481	1090.5277	18
5	**510.3650**	255.6861					I	***2084.9746***	1042.9909	2067.9481	1034.4777	2066.9640	1033.9857	17
6	581.4021	291.2047					A	***1971.8905***	986.4489	1954.8640	977.9356	1953.8800	977.4436	16
7	728.4705	364.7389					F	***1900.8534***	950.9304	1883.8269	942.4171	1882.8429	941.9251	15
8	799.5076	400.2575					A	***1753.7850***	877.3961	1736.7585	868.8829	1735.7744	868.3909	14
9	**927.5662**	464.2867	910.5397	455.7735			Q	1682.7479	841.8776	1665.7214	833.3643	1664.7373	832.8723	13
10	**1090.6295**	545.8184	1073.6030	537.3051			Y	***1554.6893***	777.8483	1537.6628	769.3350	1536.6788	768.8430	12
11	1203.7136	602.3604	1186.6871	593.8472			L	***1391.6260***	696.3166	1374.5994	687.8034	1373.6154	687.3114	11
12	1331.7722	666.3897	1314.7456	657.8765			Q	***1278.5419***	639.7746	1261.5154	631.2613	1260.5314	630.7693	10
13	**1459.8308**	730.4190	1442.8042	721.9057			Q	***1150.4834***	575.7453	1133.4568	567.2320	1132.4728	566.7400	9
14	1610.8247	805.9160	1593.7981	797.4027			C	***1022.4248***	511.7160	1005.3982	503.2027	1004.4142	502.7107	8
15	1707.8775	854.4424	1690.8509	845.9291			P	***871.4308***	436.2191	854.4043	427.7058	853.4203	427.2138	7
16	1854.9459	927.9766	1837.9193	919.4633			F	***774.3781***	387.6927	757.3515	379.1794	756.3675	378.6874	6
17	1983.9885	992.4979	1966.9619	983.9846	1965.9779	983.4926	E	***627.3097***	314.1585	610.2831	305.6452	609.2991	305.1532	5
18	2099.0154	1050.0113	2081.9889	1041.4981	2081.0048	1041.0061	D	***498.2671***	249.6372	481.2405	241.1239	480.2565	240.6319	4
19	2236.0743	1118.5408	2219.0478	1110.0275	2218.0638	1109.5355	H	***383.2401***	192.1237	366.2136	183.6104			3

**20**	2335.1427	1168.0750	2318.1162	1159.5617	2317.1322	1159.0697	**V**	246.1812	123.5942	229.1547	115.0810			**2**
**21**							**K**	147.1128	74.0600	130.0863	65.5468			**1**

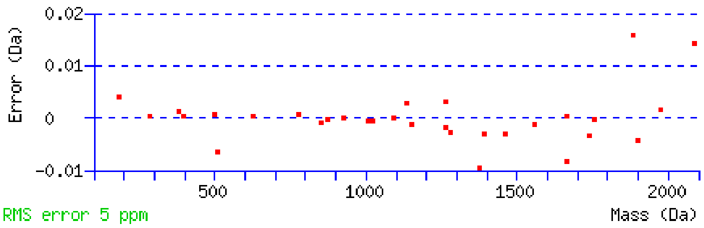

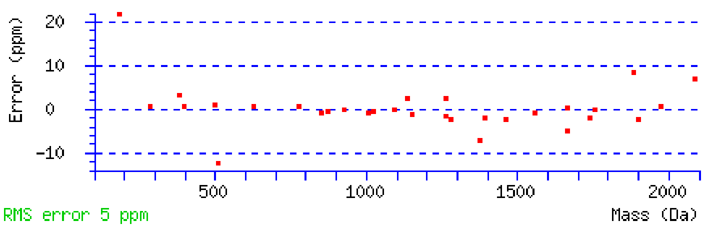

NCBI **BLAST** search of ALVLIAFAQYLQQCPFEDHVK
(Parameters: blastp, nr protein database, expect=20000, no filter, PAM30)
Other BLAST web gateways

All matches to this query

Score	Mr(calc):	Delta	Sequence
101.6	2480.2410	0.0003	ALVLIAFAQYLQQCPFEDHVK
46.4	2480.2410	0.0003	ALVLIAFAQYLQQCPFEDHVK

Mascot: http://www.matrixscience.com/

Mascot Search Results

Peptide View

MS/MS Fragmentation of **ALVLIAFAQYLQQCPFEDHVK**
Found in **sp|P02768|ALBU_HUMAN**, Serum albumin OS=Homo sapiens GN=ALB PE=1 SV=2

Match to Query 12462: 2480.241308 from(1241.127930,2+) intensity(11724308.0000) scans(18053) rtinseconds(3114) index(15647)
Title: 150818_TTSH_Patient_Plasma_38_Spectrum034038_scans__18053_RTINSECONDS=3114
Data file L:\\Ard_TTSH\\T1D\\T150818_TTSH_Patient_Plasma_38.mgf

Click mouse within plot area to zoom in by factor of two about that point
Or, [Plot from] [0] to [2200] Da [Full range]
Label all possible matches ○ Label matches used for scoring ⦿

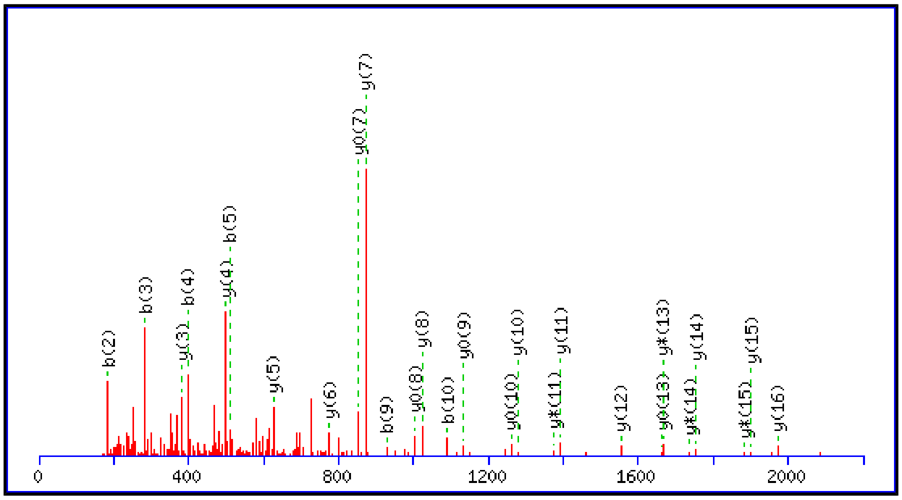

Monoisotopic mass of neutral peptide Mr(calc): 2480.2410
Variable modifications:
C14 : 4Trioxidation (CMWY)
Ions Score: 95 **Expect:** 1.1e-007
Matches : 27/186 fragment ions using 34 most intense peaks (help)

#	b	b^{++}	b*	b^{*++}	b^{0}	b^{0++}	Seq.	y	y^{++}	y*	y^{*++}	y^{0}	y^{0++}	#
1	72.0444	36.5258					A							21
2	**185.1285**	93.0679					L	2410.2111	1205.6092	2393.1846	1197.0959	2392.2006	1196.6039	20
3	**284.1969**	142.6021					V	2297.1271	1149.0672	2280.1005	1140.5539	2279.1165	1140.0619	19
4	**397.2809**	199.1441					L	2198.0587	1099.5330	2181.0321	1091.0197	2180.0481	1090.5277	18
5	**510.3650**	255.6861					I	2084.9746	1042.9909	2067.9481	1034.4777	2066.9640	1033.9857	17
6	581.4021	291.2047					A	***1971.8905***	986.4489	1954.8640	977.9356	1953.8800	977.4436	16
7	728.4705	364.7389					F	***1900.8534***	950.9304	1883.8269	942.4171	1882.8429	941.9251	15
8	799.5076	400.2575					A	***1753.7850***	877.3961	1736.7585	868.8829	1735.7744	868.3909	14
9	**927.5662**	464.2867	910.5397	455.7735			Q	1682.7479	841.8776	1665.7214	833.3643	1664.7373	832.8723	13
10	**1090.6295**	545.8184	1073.6030	537.3051			Y	***1554.6893***	777.8483	1537.6628	769.3350	1536.6788	768.8430	12
11	1203.7136	602.3604	1186.6871	593.8472			L	***1391.6260***	696.3166	1374.5994	687.8034	1373.6154	687.3114	11
12	1331.7722	666.3897	1314.7456	657.8765			Q	***1278.5419***	639.7746	1261.5154	631.2613	1260.5314	630.7693	10
13	1459.8308	730.4190	1442.8042	721.9057			Q	1150.4834	575.7453	1133.4568	567.2320	1132.4728	566.7400	9
14	1610.8247	805.9160	1593.7981	797.4027			C	***1022.4248***	511.7160	1005.3982	503.2027	1004.4142	502.7107	8
15	1707.8775	854.4424	1690.8509	845.9291			P	***871.4308***	436.2191	854.4043	427.7058	853.4203	427.2138	7
16	1854.9459	927.9766	1837.9193	919.4633			F	***774.3781***	387.6927	757.3515	379.1794	756.3675	378.6874	6
17	1983.9885	992.4979	1966.9619	983.9846	1965.9779	983.4926	E	***627.3097***	314.1585	610.2831	305.6452	609.2991	305.1532	5
18	2099.0154	1050.0113	2081.9889	1041.4981	2081.0048	1041.0061	D	***498.2671***	249.6372	481.2405	241.1239	480.2565	240.6319	4
19	2236.0743	1118.5408	2219.0478	1110.0275	2218.0638	1109.5355	H	***383.2401***	192.1237	366.2136	183.6104			3

**20**	2335.1427	1168.0750	2318.1162	1159.5617	2317.1322	1159.0697	**V**	246.1812	123.5942	229.1547	115.0810			**2**
**21**							**K**	147.1128	74.0600	130.0863	65.5468			**1**

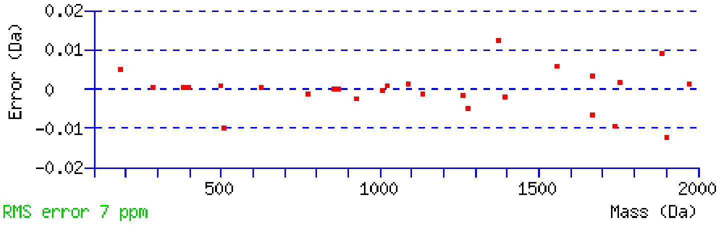

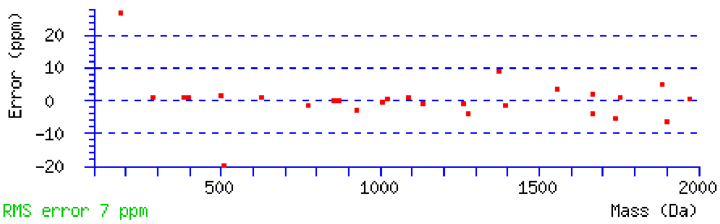

NCBI **BLAST** search of ALVLIAFAQYLQQCPFEDHVK
(Parameters: blastp, nr protein database, expect=20000, no filter, PAM30)
Other BLAST web gateways

All matches to this query

Score	Mr(calc):	Delta	Sequence
95.4	2480.2410	0.0003	ALVLIAFAQYLQQCPFEDHVK
48.3	2480.2410	0.0003	ALVLIAFAQYLQQCPFEDHVK

Mascot: http://www.matrixscience.com/

Mascot Search Results

Peptide View

MS/MS Fragmentation of **ALVLIAFAQYLQQCPFEDHVK**
Found in **sp|P02768|ALBU_HUMAN**, Serum albumin OS=Homo sapiens GN=ALB PE=1 SV=2

Match to Query 12026: 2480.239348 from(1241.126950,2+) intensity(11786250.0000) scans(18024) rtinseconds(3116) index(15416)
Title: 150818_TTSH_Patient_Plasma_35_Spectrum033688_scans__18024_RTINSECONDS=3116
Data file L:\\Ard_TTSH\\T1D\\T150818_TTSH_Patient_Plasma_35.mgf

Click mouse within plot area to zoom in by factor of two about that point
Or, Plot from 100 to 2000 Da Full range
Label all possible matches ○ Label matches used for scoring ⦿

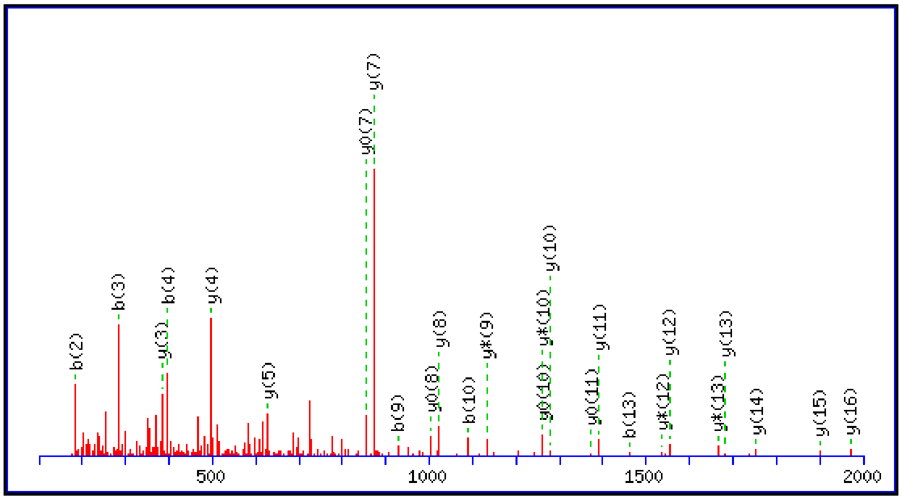

Monoisotopic mass of neutral peptide Mr(calc): 2480.2410
Variable modifications:
C14 : 4Trioxidation (CMWY)
Ions Score: 95 **Expect:** 1.2e-007
Matches : 26/186 fragment ions using 33 most intense peaks (help)

#	b	b++	b*	b*++	b0	b0++	Seq.	y	y++	y*	y*++	y0	y0++	#
1	72.0444	36.5258					A							21
2	**185.1285**	93.0679					L	2410.2111	1205.6092	2393.1846	1197.0959	2392.2006	1196.6039	20
3	**284.1969**	142.6021					V	2297.1271	1149.0672	2280.1005	1140.5539	2279.1165	1140.0619	19
4	**397.2809**	199.1441					L	2198.0587	1099.5330	2181.0321	1091.0197	2180.0481	1090.5277	18
5	510.3650	255.6861					I	2084.9746	1042.9909	2067.9481	1034.4777	2066.9640	1033.9857	17
6	581.4021	291.2047					A	***1971.8905***	986.4489	1954.8640	977.9356	1953.8800	977.4436	16
7	728.4705	364.7389					F	***1900.8534***	950.9304	1883.8269	942.4171	1882.8429	941.9251	15
8	799.5076	400.2575					A	***1753.7850***	877.3961	1736.7585	868.8829	1735.7744	868.3909	14
9	**927.5662**	464.2867	910.5397	455.7735			Q	***1682.7479***	841.8776	1665.7214	833.3643	1664.7373	832.8723	13
10	**1090.6295**	545.8184	1073.6030	537.3051			Y	***1554.6893***	777.8483	1537.6628	769.3350	1536.6788	768.8430	12
11	1203.7136	602.3604	1186.6871	593.8472			L	***1391.6260***	696.3166	1374.5994	687.8034	1373.6154	687.3114	11
12	1331.7722	666.3897	1314.7456	657.8765			Q	***1278.5419***	639.7746	1261.5154	631.2613	1260.5314	630.7693	10
13	**1459.8308**	730.4190	1442.8042	721.9057			Q	1150.4834	575.7453	1133.4568	567.2320	1132.4728	566.7400	9
14	1610.8247	805.9160	1593.7981	797.4027			C	***1022.4248***	511.7160	1005.3982	503.2027	1004.4142	502.7107	8
15	1707.8775	854.4424	1690.8509	845.9291			P	***871.4308***	436.2191	854.4043	427.7058	853.4203	427.2138	7
16	1854.9459	927.9766	1837.9193	919.4633			F	774.3781	387.6927	757.3515	379.1794	756.3675	378.6874	6
17	1983.9885	992.4979	1966.9619	983.9846	1965.9779	983.4926	E	***627.3097***	314.1585	610.2831	305.6452	609.2991	305.1532	5
18	2099.0154	1050.0113	2081.9889	1041.4981	2081.0048	1041.0061	D	***498.2671***	249.6372	481.2405	241.1239	480.2565	240.6319	4
19	2236.0743	1118.5408	2219.0478	1110.0275	2218.0638	1109.5355	H	***383.2401***	192.1237	366.2136	183.6104			3

**20**	2335.1427	1168.0750	2318.1162	1159.5617	2317.1322	1159.0697	**V**	246.1812	123.5942	229.1547	115.0810			**2**
**21**							**K**	147.1128	74.0600	130.0863	65.5468			**1**

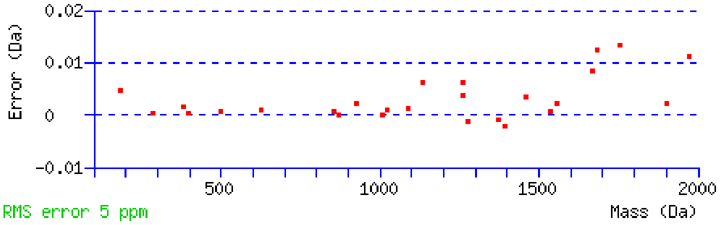

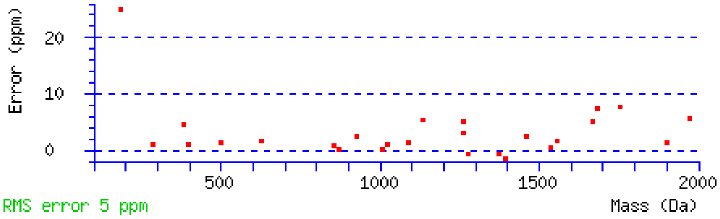

NCBI **BLAST** search of ALVLIAFAQYLQQCPFEDHVK
(Parameters: blastp, nr protein database, expect=20000, no filter, PAM30)
Other BLAST web gateways

All matches to this query

Score	Mr(calc):	Delta	Sequence
95.1	2480.2410	-0.0016	ALVLIAFAQYLQQCPFEDHVK
42.6	2480.2410	-0.0016	ALVLIAFAQYLQQCPFEDHVK

Mascot: http://www.matrixscience.com/

MATRIX SCIENCE Mascot Search Results

Peptide View

MS/MS Fragmentation of **ALVLIAFAQYLQQCPFEDHVK**
Found in **sp|P02768|ALBU_HUMAN**, Serum albumin OS=Homo sapiens GN=ALB PE=1 SV=2

Match to Query 11946: 2480.236188 from(1241.125370,2+) intensity(11179564.0000) scans(17967) rtinseconds(3113) index(15229)
Title: 150818_TTSH_Patient_Plasma_34_Spectrum033269_scans__17967_RTINSECONDS=3113
Data file L:\\Ard_TTSH\\T1D\\T150818_TTSH_Patient_Plasma_34.mgf

Click mouse within plot area to zoom in by factor of two about that point

Or, to Da

Label all possible matches ○ Label matches used for scoring ⦿

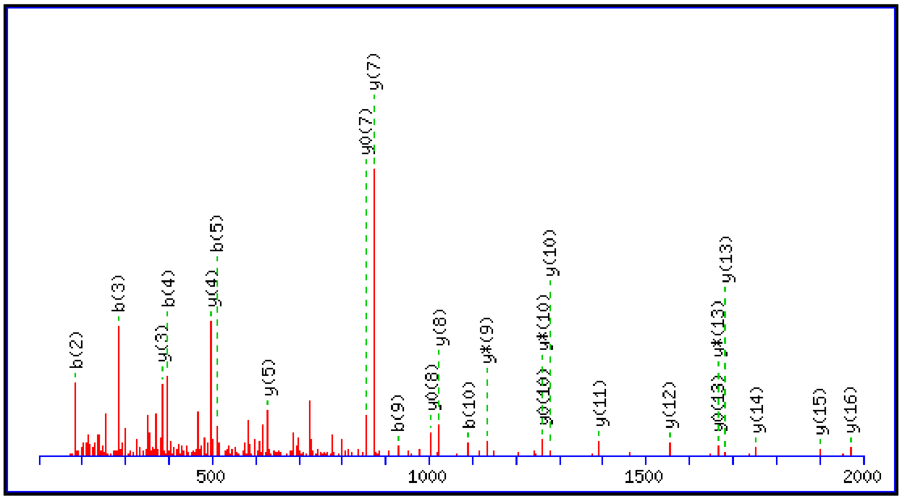

Monoisotopic mass of neutral peptide Mr(calc): 2480.2410
Variable modifications:
C14 : 4Trioxidation (CMWY)
Ions Score: 95 **Expect:** 1.3e-007
Matches : 25/186 fragment ions using 32 most intense peaks (help)

#	b	b++	b*	b*++	b0	b0++	Seq.	y	y++	y*	y*++	y0	y0++	#
1	72.0444	36.5258					A							21
2	**185.1285**	93.0679					L	2410.2111	1205.6092	2393.1846	1197.0959	2392.2006	1196.6039	20
3	**284.1969**	142.6021					V	2297.1271	1149.0672	2280.1005	1140.5539	2279.1165	1140.0619	19
4	**397.2809**	199.1441					L	2198.0587	1099.5330	2181.0321	1091.0197	2180.0481	1090.5277	18
5	**510.3650**	255.6861					I	2084.9746	1042.9909	2067.9481	1034.4777	2066.9640	1033.9857	17
6	581.4021	291.2047					A	***1971.8905***	986.4489	1954.8640	977.9356	1953.8800	977.4436	16
7	728.4705	364.7389					F	***1900.8534***	950.9304	1883.8269	942.4171	1882.8429	941.9251	15
8	799.5076	400.2575					A	***1753.7850***	877.3961	1736.7585	868.8829	1735.7744	868.3909	14
9	**927.5662**	464.2867	910.5397	455.7735			Q	***1682.7479***	841.8776	1665.7214	833.3643	1664.7373	832.8723	13
10	**1090.6295**	545.8184	1073.6030	537.3051			Y	***1554.6893***	777.8483	1537.6628	769.3350	1536.6788	768.8430	12
11	1203.7136	602.3604	1186.6871	593.8472			L	***1391.6260***	696.3166	1374.5994	687.8034	1373.6154	687.3114	11
12	1331.7722	666.3897	1314.7456	657.8765			Q	***1278.5419***	639.7746	1261.5154	631.2613	1260.5314	630.7693	10
13	1459.8308	730.4190	1442.8042	721.9057			Q	1150.4834	575.7453	1133.4568	567.2320	1132.4728	566.7400	9
14	1610.8247	805.9160	1593.7981	797.4027			C	***1022.4248***	511.7160	1005.3982	503.2027	1004.4142	502.7107	8
15	1707.8775	854.4424	1690.8509	845.9291			P	***871.4308***	436.2191	854.4043	427.7058	853.4203	427.2138	7
16	1854.9459	927.9766	1837.9193	919.4633			F	774.3781	387.6927	757.3515	379.1794	756.3675	378.6874	6
17	1983.9885	992.4979	1966.9619	983.9846	1965.9779	983.4926	E	***627.3097***	314.1585	610.2831	305.6452	609.2991	305.1532	5
18	2099.0154	1050.0113	2081.9889	1041.4981	2081.0048	1041.0061	D	***498.2671***	249.6372	481.2405	241.1239	480.2565	240.6319	4
19	2236.0743	1118.5408	2219.0478	1110.0275	2218.0638	1109.5355	H	***383.2401***	192.1237	366.2136	183.6104			3

**20**	2335.1427	1168.0750	2318.1162	1159.5617	2317.1322	1159.0697	**V**	246.1812	123.5942	229.1547	115.0810			**2**
**21**							**K**	147.1128	74.0600	130.0863	65.5468			**1**

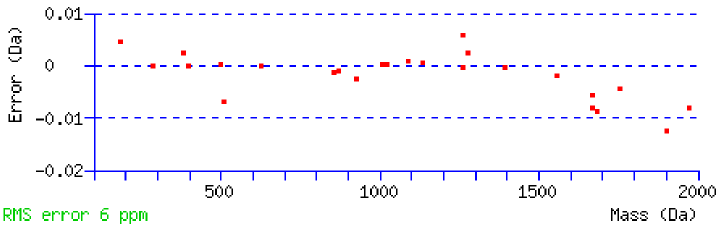

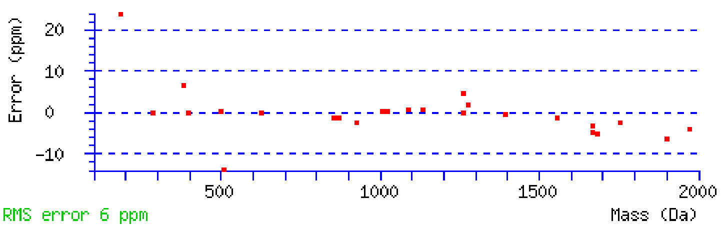

NCBI **BLAST** search of ALVLIAFAQYLQQCPFEDHVK
(Parameters: blastp, nr protein database, expect=20000, no filter, PAM30)
Other BLAST web gateways

All matches to this query

Score	Mr(calc):	Delta	Sequence
95.0	2480.2410	-0.0048	ALVLIAFAQYLQQCPFEDHVK
47.4	2480.2410	-0.0048	ALVLIAFAQYLQQCPFEDHVK

Mascot: http://www.matrixscience.com/

Mascot Search Results

Peptide View

MS/MS Fragmentation of **ALVLIAFAQYLQQCPFEDHVK**
Found in **sp|P02768|ALBU_HUMAN**, Serum albumin OS=Homo sapiens GN=ALB PE=1 SV=2

Match to Query 12603: 2480.239608 from(1241.127080,2+) intensity(4052403.7500) scans(18061) rtinseconds(3115) index(15631)
Title: 150818_TTSH_Patient_Plasma_39_Spectrum034111_scans__18061_RTINSECONDS=3115
Data file L:\\Ard_TTSH\\T1D\\T150818_TTSH_Patient_Plasma_39.mgf

Click mouse within plot area to zoom in by factor of two about that point
Or, Plot from 0 to 2200 Da Full range
Label all possible matches ○ Label matches used for scoring ⊙

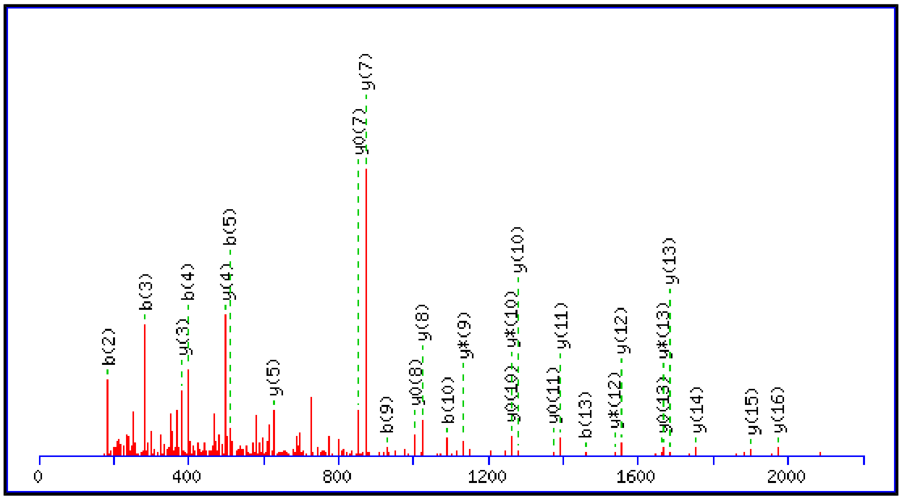

Monoisotopic mass of neutral peptide Mr(calc): 2480.2410
Variable modifications:
C14 : 4Trioxidation (CMWY)
Ions Score: 93 **Expect:** 2e-007
Matches : 28/186 fragment ions using 36 most intense peaks (help)

#	b	b^{++}	b^{*}	b^{*++}	b^{0}	b^{0++}	Seq.	y	y^{++}	y^{*}	y^{*++}	y^{0}	y^{0++}	#
1	72.0444	36.5258					A							21
2	**185.1285**	93.0679					L	2410.2111	1205.6092	2393.1846	1197.0959	2392.2006	1196.6039	20
3	**284.1969**	142.6021					V	2297.1271	1149.0672	2280.1005	1140.5539	2279.1165	1140.0619	19
4	**397.2809**	199.1441					L	2198.0587	1099.5330	2181.0321	1091.0197	2180.0481	1090.5277	18
5	**510.3650**	255.6861					I	2084.9746	1042.9909	2067.9481	1034.4777	2066.9640	1033.9857	17
6	581.4021	291.2047					A	***1971.8905***	986.4489	1954.8640	977.9356	1953.8800	977.4436	16
7	728.4705	364.7389					F	***1900.8534***	950.9304	1883.8269	942.4171	1882.8429	941.9251	15
8	799.5076	400.2575					A	***1753.7850***	877.3961	1736.7585	868.8829	1735.7744	868.3909	14
9	**927.5662**	464.2867	910.5397	455.7735			Q	***1682.7479***	841.8776	1665.7214	833.3643	1664.7373	832.8723	13
10	**1090.6295**	545.8184	1073.6030	537.3051			Y	***1554.6893***	777.8483	1537.6628	769.3350	1536.6788	768.8430	12
11	1203.7136	602.3604	1186.6871	593.8472			L	***1391.6260***	696.3166	1374.5994	687.8034	1373.6154	687.3114	11
12	1331.7722	666.3897	1314.7456	657.8765			Q	***1278.5419***	639.7746	1261.5154	631.2613	1260.5314	630.7693	10
13	**1459.8308**	730.4190	1442.8042	721.9057			Q	1150.4834	575.7453	1133.4568	567.2320	1132.4728	566.7400	9
14	1610.8247	805.9160	1593.7981	797.4027			C	***1022.4248***	511.7160	1005.3982	503.2027	1004.4142	502.7107	8
15	1707.8775	854.4424	1690.8509	845.9291			P	***871.4308***	436.2191	854.4043	427.7058	853.4203	427.2138	7
16	1854.9459	927.9766	1837.9193	919.4633			F	774.3781	387.6927	757.3515	379.1794	756.3675	378.6874	6
17	1983.9885	992.4979	1966.9619	983.9846	1965.9779	983.4926	E	***627.3097***	314.1585	610.2831	305.6452	609.2991	305.1532	5
18	2099.0154	1050.0113	2081.9889	1041.4981	2081.0048	1041.0061	D	***498.2671***	249.6372	481.2405	241.1239	480.2565	240.6319	4
19	2236.0743	1118.5408	2219.0478	1110.0275	2218.0638	1109.5355	H	***383.2401***	192.1237	366.2136	183.6104			3

**20**	2335.1427	1168.0750	2318.1162	1159.5617	2317.1322	1159.0697	**V**	246.1812	123.5942	229.1547	115.0810			**2**
**21**							**K**	147.1128	74.0600	130.0863	65.5468			**1**

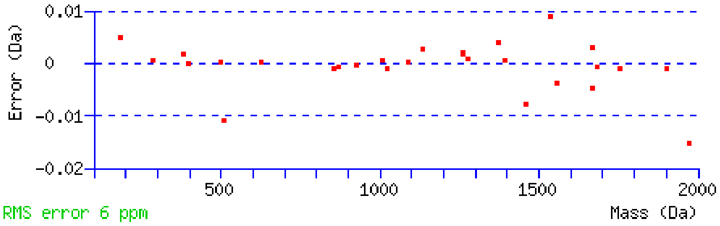

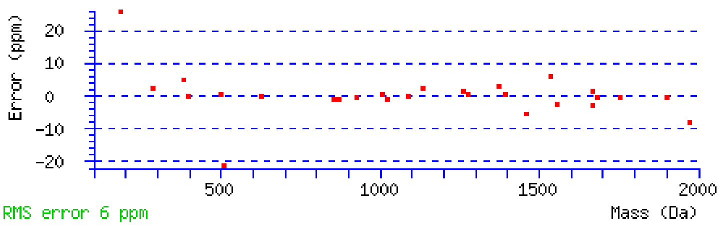

NCBI **BLAST** search of ALVLIAFAQYLQQCPFEDHVK
(Parameters: blastp, nr protein database, expect=20000, no filter, PAM30)
Other BLAST web gateways

All matches to this query

Score	Mr(calc):	Delta	Sequence
93.0	2480.2410	-0.0014	ALVLIAFAQYLQQCPFEDHVK
43.9	2480.2410	-0.0014	ALVLIAFAQYLQQCPFEDHVK

Mascot Search Results

Peptide View

MS/MS Fragmentation of **ALVLIAFAQYLQQCPFEDHVK**
Found in **sp|P02768|ALBU_HUMAN**, Serum albumin OS=Homo sapiens GN=ALB PE=1 SV=2

Match to Query 12027: 2480.242452 from(827.754760,3+) intensity(2998150.5000) scans(18020) rtinseconds(3115) index(15413)
Title: 150818_TTSH_Patient_Plasma_35_Spectrum033685_scans__18020_RTINSECONDS=3115
Data file L:\\Ard_TTSH\\T1D\\T150818_TTSH_Patient_Plasma_35.mgf

Click mouse within plot area to zoom in by factor of two about that point
Or, Plot from 100 to 2000 Da Full range
Label all possible matches ○ Label matches used for scoring ⊙

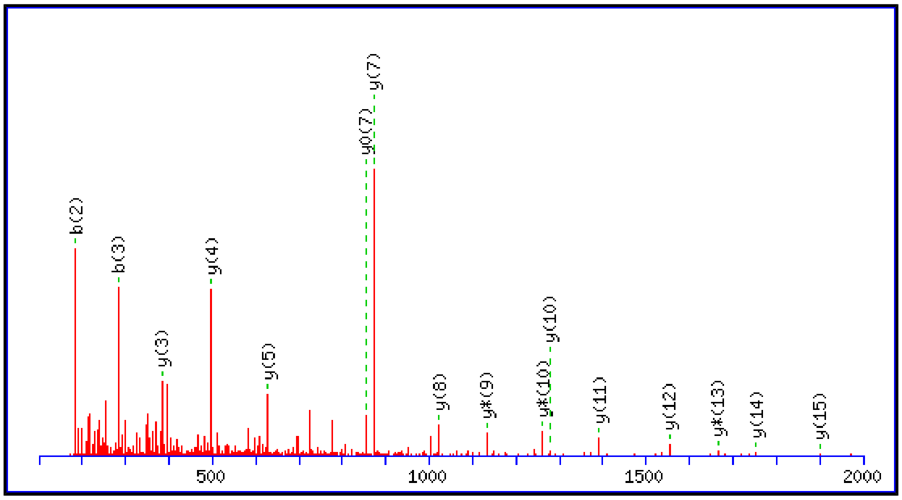

Monoisotopic mass of neutral peptide Mr(calc): 2480.2410
Variable modifications:
C14 : 4Trioxidation (CMWY)
Ions Score: 87 **Expect:** 8.3e-007
Matches : 16/186 fragment ions using 18 most intense peaks (help)

#	b	b++	b*	b*++	b0	b0++	Seq.	y	y++	y*	y*++	y0	y0++	#
1	72.0444	36.5258					A							21
2	**185.1285**	93.0679					L	2410.2111	1205.6092	2393.1846	1197.0959	2392.2006	1196.6039	20
3	**284.1969**	142.6021					V	2297.1271	1149.0672	2280.1005	1140.5539	2279.1165	1140.0619	19
4	397.2809	199.1441					L	2198.0587	1099.5330	2181.0321	1091.0197	2180.0481	1090.5277	18
5	510.3650	255.6861					I	2084.9746	1042.9909	2067.9481	1034.4777	2066.9640	1033.9857	17
6	581.4021	291.2047					A	1971.8905	986.4489	1954.8640	977.9356	1953.8800	977.4436	16
7	728.4705	364.7389					F	***1900.8534***	950.9304	1883.8269	942.4171	1882.8429	941.9251	15
8	799.5076	400.2575					A	***1753.7850***	877.3961	1736.7585	868.8829	1735.7744	868.3909	14
9	927.5662	464.2867	910.5397	455.7735			Q	1682.7479	841.8776	1665.7214	833.3643	1664.7373	832.8723	13
10	1090.6295	545.8184	1073.6030	537.3051			Y	***1554.6893***	777.8483	1537.6628	769.3350	1536.6788	768.8430	12
11	1203.7136	602.3604	1186.6871	593.8472			L	***1391.6260***	696.3166	1374.5994	687.8034	1373.6154	687.3114	11
12	1331.7722	666.3897	1314.7456	657.8765			Q	***1278.5419***	639.7746	1261.5154	631.2613	1260.5314	630.7693	10
13	1459.8308	730.4190	1442.8042	721.9057			Q	1150.4834	575.7453	1133.4568	567.2320	1132.4728	566.7400	9
14	1610.8247	805.9160	1593.7981	797.4027			C	***1022.4248***	511.7160	1005.3982	503.2027	1004.4142	502.7107	8
15	1707.8775	854.4424	1690.8509	845.9291			P	***871.4308***	436.2191	854.4043	427.7058	853.4203	427.2138	7
16	1854.9459	927.9766	1837.9193	919.4633			F	774.3781	387.6927	757.3515	379.1794	756.3675	378.6874	6
17	1983.9885	992.4979	1966.9619	983.9846	1965.9779	983.4926	E	***627.3097***	314.1585	610.2831	305.6452	609.2991	305.1532	5
18	2099.0154	1050.0113	2081.9889	1041.4981	2081.0048	1041.0061	D	***498.2671***	249.6372	481.2405	241.1239	480.2565	240.6319	4
19	2236.0743	1118.5408	2219.0478	1110.0275	2218.0638	1109.5355	H	***383.2401***	192.1237	366.2136	183.6104			3

**20**	2335.1427	1168.0750	2318.1162	1159.5617	2317.1322	1159.0697	**V**	246.1812	123.5942	229.1547	115.0810			**2**
**21**							**K**	147.1128	74.0600	130.0863	65.5468			**1**

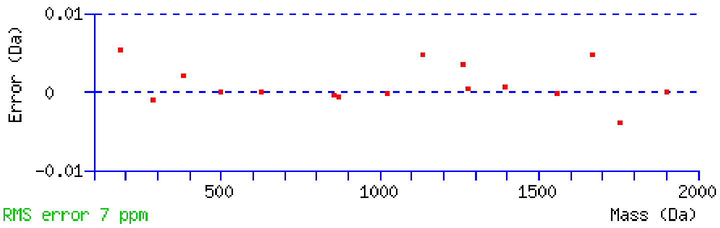

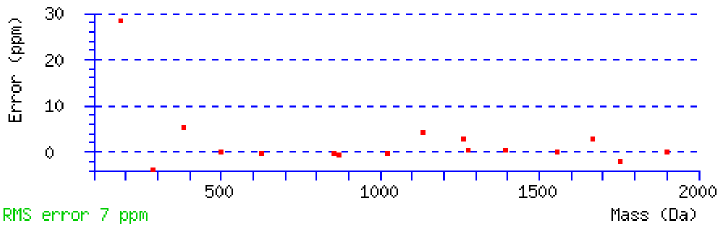

NCBI **BLAST** search of ALVLIAFAQYLQQCPFEDHVK
(Parameters: blastp, nr protein database, expect=20000, no filter, PAM30)
Other BLAST web gateways

All matches to this query

Score	Mr(calc):	Delta	Sequence
86.7	2480.2410	0.0015	ALVLIAFAQYLQQCPFEDHVK
39.0	2480.2410	0.0015	ALVLIAFAQYLQQCPFEDHVK

Mascot: http://www.matrixscience.com/

{MATRIX SCIENCE} Mascot Search Results

Peptide View

MS/MS Fragmentation of **ALVLIAFAQYLQQCPFEDHVK**
Found in **sp|P02768|ALBU_HUMAN**, Serum albumin OS=Homo sapiens GN=ALB PE=1 SV=2

Match to Query 10889: 2480.236668 from(1241.125610,2+) intensity(929313.1250) scans(16228) rtinseconds(3170) index(12991)
Title: 150801_TTSH_Patient_Plasma_76_Spectrum028152_scans__16228_RTINSECONDS=3170
Data file L:\\Ard_TTSH\\T1D\\T150801_TTSH_Patient_Plasma_76.mgf

Click mouse within plot area to zoom in by factor of two about that point

Or, to Da

Label all possible matches ○ Label matches used for scoring ⊙

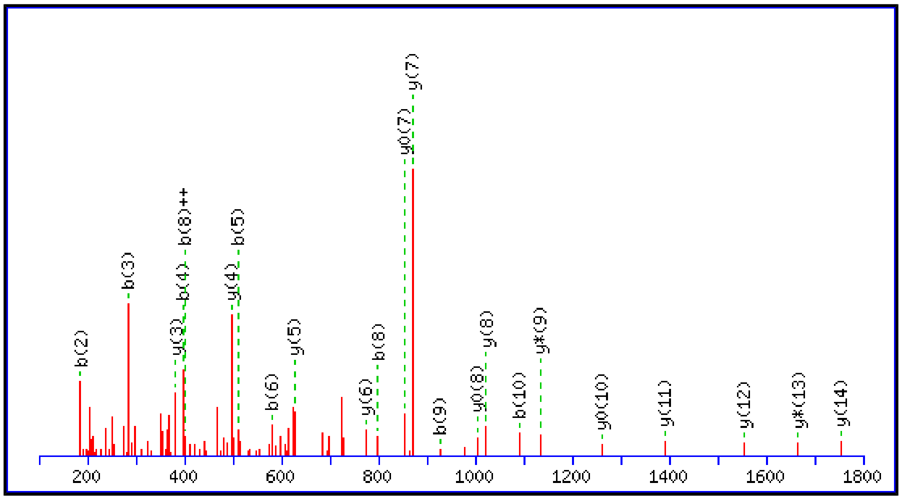

Monoisotopic mass of neutral peptide Mr(calc): 2480.2410
Variable modifications:
C14 : 4Trioxidation (CMWY)
Ions Score: 82 **Expect:** 2.4e-006
Matches : 23/186 fragment ions using 32 most intense peaks (help)

#	b	b++	b*	b*++	b0	b0++	Seq.	y	y++	y*	y*++	y0	y0++	#
1	72.0444	36.5258					A							21
2	***185.1285***	93.0679					L	2410.2111	1205.6092	2393.1846	1197.0959	2392.2006	1196.6039	20
3	***284.1969***	142.6021					V	2297.1271	1149.0672	2280.1005	1140.5539	2279.1165	1140.0619	19
4	***397.2809***	199.1441					L	2198.0587	1099.5330	2181.0321	1091.0197	2180.0481	1090.5277	18
5	***510.3650***	255.6861					I	2084.9746	1042.9909	2067.9481	1034.4777	2066.9640	1033.9857	17
6	***581.4021***	291.2047					A	1971.8905	986.4489	1954.8640	977.9356	1953.8800	977.4436	16
7	728.4705	364.7389					F	1900.8534	950.9304	1883.8269	942.4171	1882.8429	941.9251	15
8	***799.5076***	**400.2575**					A	***1753.7850***	877.3961	1736.7585	868.8829	1735.7744	868.3909	14
9	***927.5662***	464.2867	910.5397	455.7735			Q	1682.7479	841.8776	1665.7214	833.3643	1664.7373	832.8723	13
10	***1090.6295***	545.8184	1073.6030	537.3051			Y	***1554.6893***	777.8483	1537.6628	769.3350	1536.6788	768.8430	12
11	1203.7136	602.3604	1186.6871	593.8472			L	***1391.6260***	696.3166	1374.5994	687.8034	1373.6154	687.3114	11
12	1331.7722	666.3897	1314.7456	657.8765			Q	1278.5419	639.7746	1261.5154	631.2613	1260.5314	630.7693	10
13	1459.8308	730.4190	1442.8042	721.9057			Q	1150.4834	575.7453	1133.4568	567.2320	1132.4728	566.7400	9
14	1610.8247	805.9160	1593.7981	797.4027			C	***1022.4248***	511.7160	1005.3982	503.2027	1004.4142	502.7107	8
15	1707.8775	854.4424	1690.8509	845.9291			P	***871.4308***	436.2191	854.4043	427.7058	853.4203	427.2138	7
16	1854.9459	927.9766	1837.9193	919.4633			F	***774.3781***	387.6927	757.3515	379.1794	756.3675	378.6874	6
17	1983.9885	992.4979	1966.9619	983.9846	1965.9779	983.4926	E	***627.3097***	314.1585	610.2831	305.6452	609.2991	305.1532	5
18	2099.0154	1050.0113	2081.9889	1041.4981	2081.0048	1041.0061	D	***498.2671***	249.6372	481.2405	241.1239	480.2565	240.6319	4
19	2236.0743	1118.5408	2219.0478	1110.0275	2218.0638	1109.5355	H	***383.2401***	192.1237	366.2136	183.6104			3

**20**	2335.1427	1168.0750	2318.1162	1159.5617	2317.1322	1159.0697	**V**	246.1812	123.5942	229.1547	115.0810			**2**
**21**							**K**	147.1128	74.0600	130.0863	65.5468			**1**

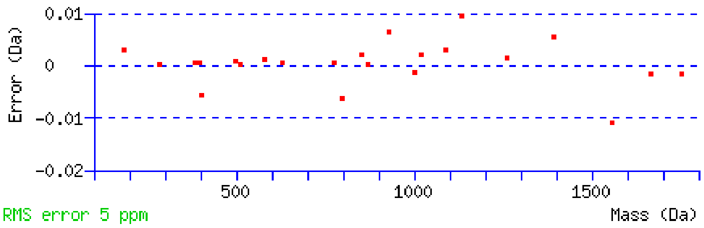

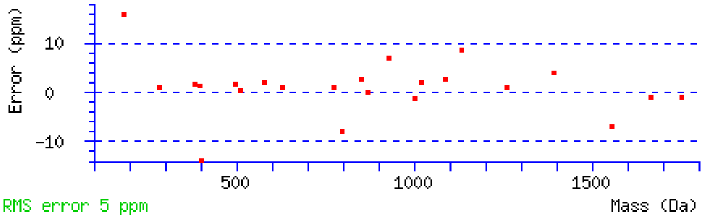

NCBI **BLAST** search of ALVLIAFAQYLQQCPFEDHVK
(Parameters: blastp, nr protein database, expect=20000, no filter, PAM30)
Other BLAST web gateways

All matches to this query

Score	Mr(calc):	Delta	Sequence
82.1	2480.2410	-0.0043	ALVLIAFAQYLQQCPFEDHVK
50.9	2480.2410	-0.0043	ALVLIAFAQYLQQCPFEDHVK
1.6	2479.2549	0.9817	ALVILAKGAEEMETVIPVDVMR

Mascot Search Results

Peptide View

MS/MS Fragmentation of **ALVLIAFAQYLQQCPFEDHVK**
Found in **sp|P02768|ALBU_HUMAN**, Serum albumin OS=Homo sapiens GN=ALB PE=1 SV=2

Match to Query 11407: 2480.239902 from(827.753910,3+) intensity(2010529.6250) scans(16469) rtinseconds(3149) index(13374)
Title: 150801_TTSH_Patient_Plasma_74_Spectrum028924_scans__16469_RTINSECONDS=3149
Data file L:\\Ard_TTSH\\T1D\\T150801_TTSH_Patient_Plasma_74.mgf

Click mouse within plot area to zoom in by factor of two about that point
Or, Plot from 100 to 2000 Da Full range
Label all possible matches ○ Label matches used for scoring ⊙

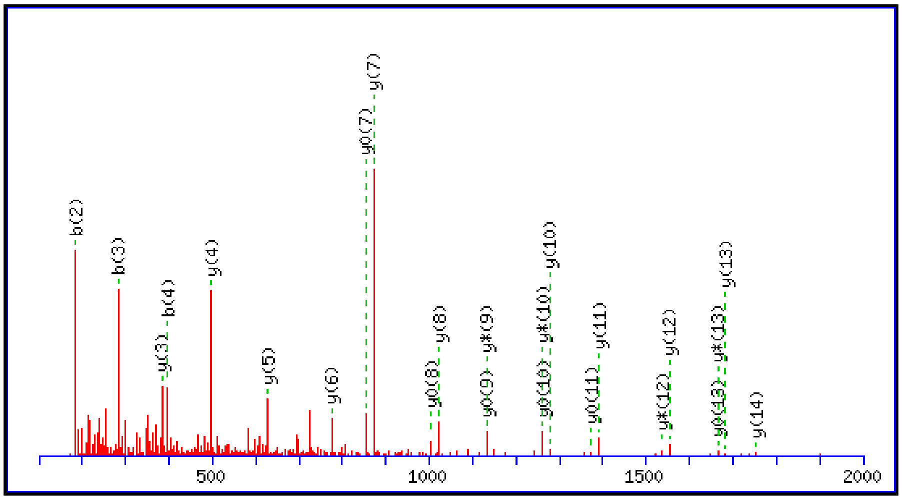

Monoisotopic mass of neutral peptide Mr(calc): 2480.2410
Variable modifications:
C14 : 4Trioxidation (CMWY)
Ions Score: 79 **Expect:** 5.1e-006
Matches : 24/186 fragment ions using 33 most intense peaks (help)

#	b	b^{++}	b*	b^{*++}	b^{0}	b^{0++}	Seq.	y	y^{++}	y*	y^{*++}	y^{0}	y^{0++}	#
1	72.0444	36.5258					A							21
2	**185.1285**	93.0679					L	2410.2111	1205.6092	2393.1846	1197.0959	2392.2006	1196.6039	20
3	**284.1969**	142.6021					V	2297.1271	1149.0672	2280.1005	1140.5539	2279.1165	1140.0619	19
4	**397.2809**	199.1441					L	2198.0587	1099.5330	2181.0321	1091.0197	2180.0481	1090.5277	18
5	510.3650	255.6861					I	2084.9746	1042.9909	2067.9481	1034.4777	2066.9640	1033.9857	17
6	581.4021	291.2047					A	1971.8905	986.4489	1954.8640	977.9356	1953.8800	977.4436	16
7	728.4705	364.7389					F	1900.8534	950.9304	1883.8269	942.4171	1882.8429	941.9251	15
8	799.5076	400.2575					A	**1753.7850**	877.3961	1736.7585	868.8829	1735.7744	868.3909	14
9	927.5662	464.2867	910.5397	455.7735			Q	**1682.7479**	841.8776	1665.7214	833.3643	1664.7373	832.8723	13
10	1090.6295	545.8184	1073.6030	537.3051			Y	**1554.6893**	777.8483	1537.6628	769.3350	1536.6788	768.8430	12
11	1203.7136	602.3604	1186.6871	593.8472			L	**1391.6260**	696.3166	1374.5994	687.8034	1373.6154	687.3114	11
12	1331.7722	666.3897	1314.7456	657.8765			Q	**1278.5419**	639.7746	1261.5154	631.2613	1260.5314	630.7693	10
13	1459.8308	730.4190	1442.8042	721.9057			Q	1150.4834	575.7453	1133.4568	567.2320	1132.4728	566.7400	9
14	1610.8247	805.9160	1593.7981	797.4027			C	**1022.4248**	511.7160	1005.3982	503.2027	1004.4142	502.7107	8
15	1707.8775	854.4424	1690.8509	845.9291			P	**871.4308**	436.2191	854.4043	427.7058	853.4203	427.2138	7
16	1854.9459	927.9766	1837.9193	919.4633			F	**774.3781**	387.6927	757.3515	379.1794	756.3675	378.6874	6
17	1983.9885	992.4979	1966.9619	983.9846	1965.9779	983.4926	E	**627.3097**	314.1585	610.2831	305.6452	609.2991	305.1532	5
18	2099.0154	1050.0113	2081.9889	1041.4981	2081.0048	1041.0061	D	**498.2671**	249.6372	481.2405	241.1239	480.2565	240.6319	4
19	2236.0743	1118.5408	2219.0478	1110.0275	2218.0638	1109.5355	H	**383.2401**	192.1237	366.2136	183.6104			3

**20**	2335.1427	1168.0750	2318.1162	1159.5617	2317.1322	1159.0697	**V**	246.1812	123.5942	229.1547	115.0810			**2**
**21**							**K**	147.1128	74.0600	130.0863	65.5468			**1**

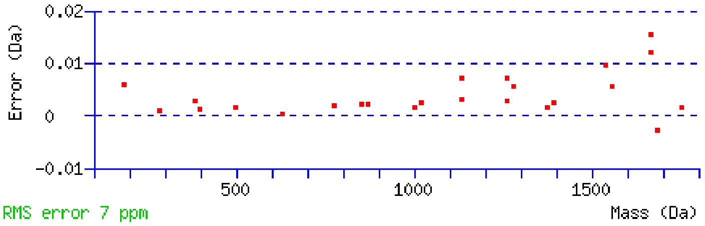

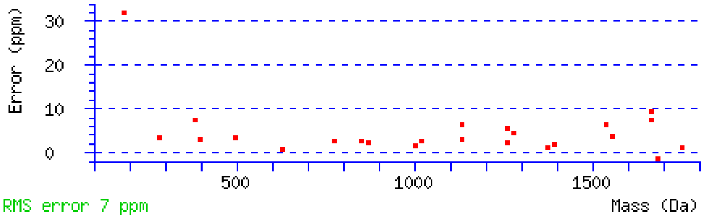

NCBI **BLAST** search of ALVLIAFAQYLQQCPFEDHVK
(Parameters: blastp, nr protein database, expect=20000, no filter, PAM30)
Other BLAST web gateways

All matches to this query

Score	Mr(calc):	Delta	Sequence
78.9	2480.2410	-0.0011	ALVLIAFAQYLQQCPFEDHVK
33.5	2480.2410	-0.0011	ALVLIAFAQYLQQCPFEDHVK

Mascot: http://www.matrixscience.com/

Mascot Search Results

Peptide View

MS/MS Fragmentation of **ALVLIAFAQYLQQCPFEDHVK**
Found in **sp|P02768|ALBU_HUMAN**, Serum albumin OS=Homo sapiens GN=ALB PE=1 SV=2

Match to Query 12475: 2480.238792 from(827.753540,3+) intensity(6294169.0000) scans(18070) rtinseconds(3115) index(15664)
Title: 150818_TTSH_Patient_Plasma_37_Spectrum034066_scans__18070_RTINSECONDS=3115
Data file L:\\Ard_TTSH\\T1D\\T150818_TTSH_Patient_Plasma_37.mgf

Click mouse within plot area to zoom in by factor of two about that point

Or, to Da

Label all possible matches ○ Label matches used for scoring ⦿

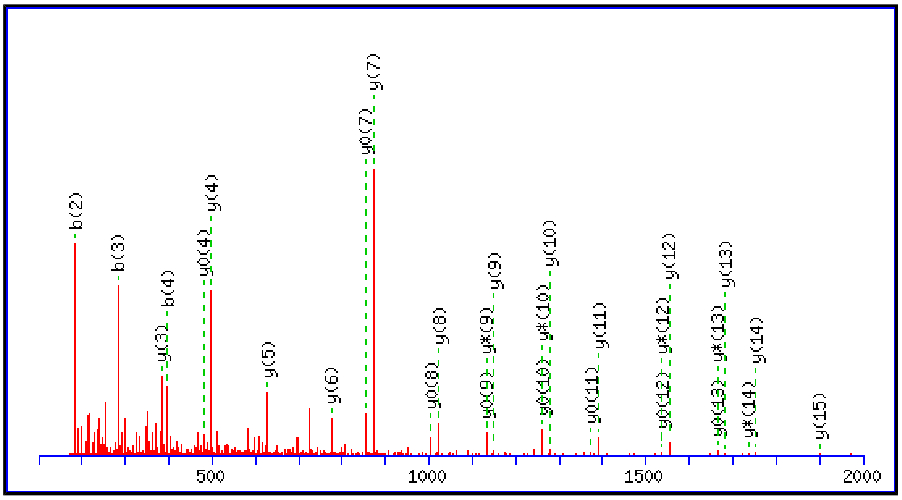

Monoisotopic mass of neutral peptide Mr(calc): 2480.2410
Variable modifications:
C14 : 4Trioxidation (CMWY)
Ions Score: 79 **Expect:** 5.2e-006
Matches : 29/186 fragment ions using 51 most intense peaks (help)

#	b	b^{++}	b*	b^{*++}	b^0	b^{0++}	Seq.	y	y^{++}	y*	y^{*++}	y^0	y^{0++}	#
1	72.0444	36.5258					A							21
2	**185.1285**	93.0679					L	2410.2111	1205.6092	2393.1846	1197.0959	2392.2006	1196.6039	20
3	**284.1969**	142.6021					V	2297.1271	1149.0672	2280.1005	1140.5539	2279.1165	1140.0619	19
4	**397.2809**	199.1441					L	2198.0587	1099.5330	2181.0321	1091.0197	2180.0481	1090.5277	18
5	510.3650	255.6861					I	2084.9746	1042.9909	2067.9481	1034.4777	2066.9640	1033.9857	17
6	581.4021	291.2047					A	1971.8905	986.4489	1954.8640	977.9356	1953.8800	977.4436	16
7	728.4705	364.7389					F	***1900.8534***	950.9304	1883.8269	942.4171	1882.8429	941.9251	15
8	799.5076	400.2575					A	***1753.7850***	877.3961	1736.7585	868.8829	1735.7744	868.3909	14
9	927.5662	464.2867	910.5397	455.7735			Q	***1682.7479***	841.8776	1665.7214	833.3643	1664.7373	832.8723	13
10	1090.6295	545.8184	1073.6030	537.3051			Y	***1554.6893***	777.8483	1537.6628	769.3350	1536.6788	768.8430	12
11	1203.7136	602.3604	1186.6871	593.8472			L	***1391.6260***	696.3166	1374.5994	687.8034	1373.6154	687.3114	11
12	1331.7722	666.3897	1314.7456	657.8765			Q	***1278.5419***	639.7746	1261.5154	631.2613	1260.5314	630.7693	10
13	1459.8308	730.4190	1442.8042	721.9057			Q	***1150.4834***	575.7453	1133.4568	567.2320	1132.4728	566.7400	9
14	1610.8247	805.9160	1593.7981	797.4027			C	***1022.4248***	511.7160	1005.3982	503.2027	1004.4142	502.7107	8
15	1707.8775	854.4424	1690.8509	845.9291			P	***871.4308***	436.2191	854.4043	427.7058	853.4203	427.2138	7
16	1854.9459	927.9766	1837.9193	919.4633			F	***774.3781***	387.6927	757.3515	379.1794	756.3675	378.6874	6
17	1983.9885	992.4979	1966.9619	983.9846	1965.9779	983.4926	E	***627.3097***	314.1585	610.2831	305.6452	609.2991	305.1532	5
18	2099.0154	1050.0113	2081.9889	1041.4981	2081.0048	1041.0061	D	***498.2671***	249.6372	481.2405	241.1239	480.2565	240.6319	4
19	2236.0743	1118.5408	2219.0478	1110.0275	2218.0638	1109.5355	H	***383.2401***	192.1237	366.2136	183.6104			3

**20**	2335.1427	1168.0750	2318.1162	1159.5617	2317.1322	1159.0697	**V**	246.1812	123.5942	229.1547	115.0810			**2**
**21**							**K**	147.1128	74.0600	130.0863	65.5468			**1**

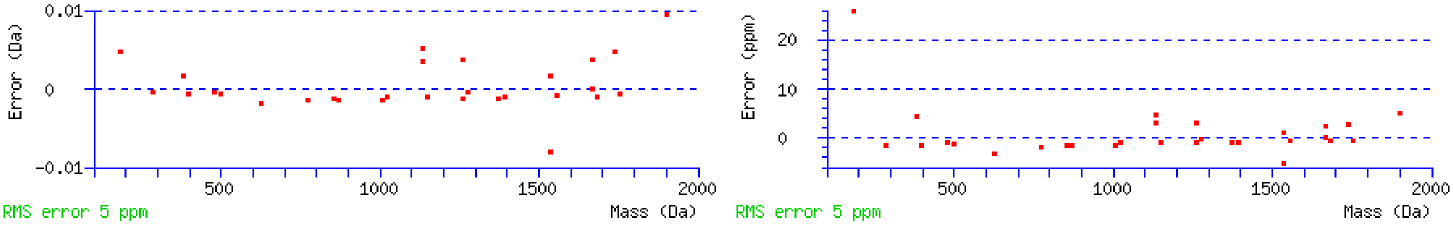

NCBI **BLAST** search of ALVLIAFAQYLQQCPFEDHVK
(Parameters: blastp, nr protein database, expect=20000, no filter, PAM30)
Other BLAST web gateways

All matches to this query

Score	Mr(calc):	Delta	Sequence
78.8	2480.2410	-0.0022	ALVLIAFAQYLQQCPFEDHVK
32.9	2480.2410	-0.0022	ALVLIAFAQYLQQCPFEDHVK

**Mascot:** http://www.matrixscience.com/

Mascot Search Results

Peptide View

MS/MS Fragmentation of **ALVLIAFAQYLQQCPFEDHVK**
Found in **sp|P02768|ALBU_HUMAN**, Serum albumin OS=Homo sapiens GN=ALB PE=1 SV=2

Match to Query 11572: 2480.231292 from(827.751040,3+) intensity(1218234.5000) scans(17233) rtinseconds(3127) index(14551)
Title: 150825_TTSH_Patient_Plasma_62_Spectrum031156_scans__17233_RTINSECONDS=3127
Data file L:\\Ard_TTSH\\T1D\\T150825_TTSH_Patient_Plasma_62.mgf

Click mouse within plot area to zoom in by factor of two about that point
Or, to Da
Label all possible matches ○ Label matches used for scoring ⊙

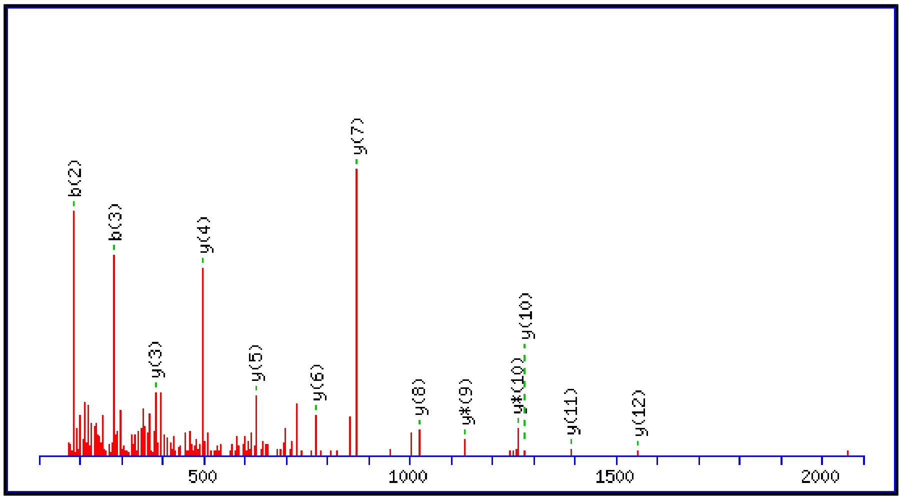

Monoisotopic mass of neutral peptide Mr(calc): 2480.2410
Variable modifications:
C14 : 4Trioxidation (CMWY)
Ions Score: 75 **Expect:** 1.4e-005
Matches : 13/186 fragment ions using 15 most intense peaks (help)

#	b	b++	b*	b*++	b0	b0++	Seq.	y	y++	y*	y*++	y0	y0++	#
1	72.0444	36.5258					A							21
2	185.1285	93.0679					L	2410.2111	1205.6092	2393.1846	1197.0959	2392.2006	1196.6039	20
3	284.1969	142.6021					V	2297.1271	1149.0672	2280.1005	1140.5539	2279.1165	1140.0619	19
4	397.2809	199.1441					L	2198.0587	1099.5330	2181.0321	1091.0197	2180.0481	1090.5277	18
5	510.3650	255.6861					I	2084.9746	1042.9909	2067.9481	1034.4777	2066.9640	1033.9857	17
6	581.4021	291.2047					A	1971.8905	986.4489	1954.8640	977.9356	1953.8800	977.4436	16
7	728.4705	364.7389					F	1900.8534	950.9304	1883.8269	942.4171	1882.8429	941.9251	15
8	799.5076	400.2575					A	1753.7850	877.3961	1736.7585	868.8829	1735.7744	868.3909	14
9	927.5662	464.2867	910.5397	455.7735			Q	1682.7479	841.8776	1665.7214	833.3643	1664.7373	832.8723	13
10	1090.6295	545.8184	1073.6030	537.3051			Y	1554.6893	777.8483	1537.6628	769.3350	1536.6788	768.8430	12
11	1203.7136	602.3604	1186.6871	593.8472			L	1391.6260	696.3166	1374.5994	687.8034	1373.6154	687.3114	11
12	1331.7722	666.3897	1314.7456	657.8765			Q	1278.5419	639.7746	1261.5154	631.2613	1260.5314	630.7693	10
13	1459.8308	730.4190	1442.8042	721.9057			Q	1150.4834	575.7453	1133.4568	567.2320	1132.4728	566.7400	9
14	1610.8247	805.9160	1593.7981	797.4027			C	1022.4248	511.7160	1005.3982	503.2027	1004.4142	502.7107	8
15	1707.8775	854.4424	1690.8509	845.9291			P	871.4308	436.2191	854.4043	427.7058	853.4203	427.2138	7
16	1854.9459	927.9766	1837.9193	919.4633			F	774.3781	387.6927	757.3515	379.1794	756.3675	378.6874	6
17	1983.9885	992.4979	1966.9619	983.9846	1965.9779	983.4926	E	627.3097	314.1585	610.2831	305.6452	609.2991	305.1532	5
18	2099.0154	1050.0113	2081.9889	1041.4981	2081.0048	1041.0061	D	498.2671	249.6372	481.2405	241.1239	480.2565	240.6319	4
19	2236.0743	1118.5408	2219.0478	1110.0275	2218.0638	1109.5355	H	383.2401	192.1237	366.2136	183.6104			3

**20**	2335.1427	1168.0750	2318.1162	1159.5617	2317.1322	1159.0697	**V**	246.1812	123.5942	229.1547	115.0810			**2**
**21**							**K**	147.1128	74.0600	130.0863	65.5468			**1**

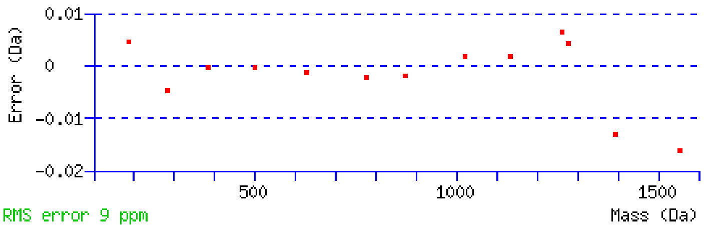

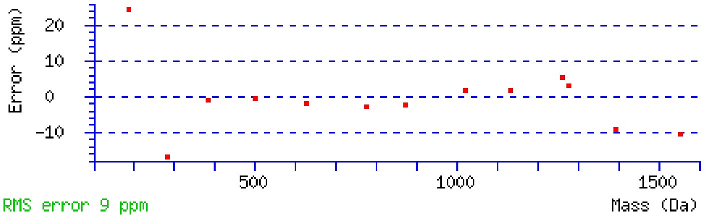

NCBI **BLAST** search of ALVLIAFAQYLQQCPFEDHVK
(Parameters: blastp, nr protein database, expect=20000, no filter, PAM30)
Other BLAST web gateways

All matches to this query

Score	Mr(calc):	Delta	Sequence
74.7	2480.2410	-0.0097	ALVLIAFAQYLQQCPFEDHVK
29.5	2480.2410	-0.0097	ALVLIAFAQYLQQCPFEDHVK

**Mascot:** http://www.matrixscience.com/

Mascot Search Results

Peptide View

MS/MS Fragmentation of **ALVLIAFAQYLQQCPFEDHVK**
Found in **sp|P02768|ALBU_HUMAN**, Serum albumin OS=Homo sapiens GN=ALB PE=1 SV=2

Match to Query 11572: 2480.231292 from(827.751040,3+) intensity(1218234.5000) scans(17233) rtinseconds(3127) index(14551)
Title: N50825_TTSH_Patient_Plasma_62_Spectrum014789_scans__17233_RTINSECONDS=3127
Data file L:\\Ard_TTSH\\TN1D\\TN50825_TTSH_Patient_Plasma_62.mgf

Click mouse within plot area to zoom in by factor of two about that point

Or, to Da

Label all possible matches Label matches used for scoring

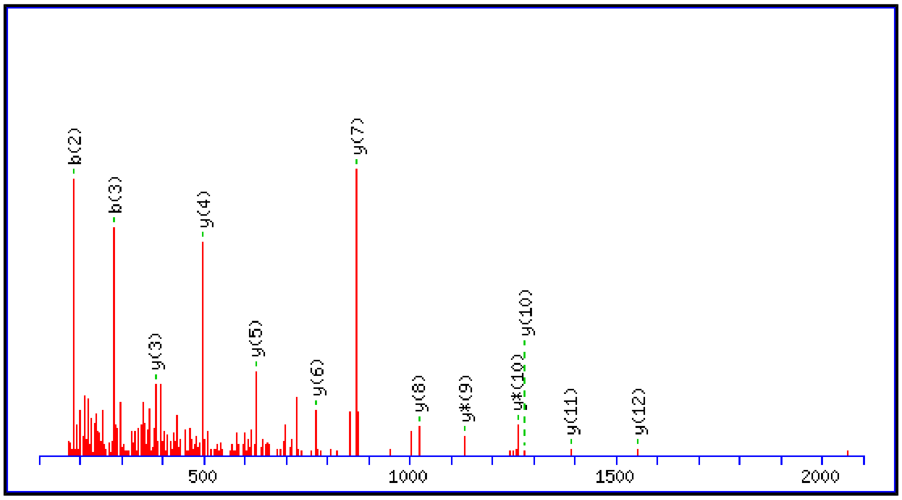

Monoisotopic mass of neutral peptide Mr(calc): 2480.2410
Variable modifications:
C14 : 4Trioxidation (CMWY)
Ions Score: 74 **Expect:** 1.6e-005
Matches : 13/186 fragment ions using 15 most intense peaks (help)

#	b	b++	b*	b*++	b0	b0++	Seq.	y	y++	y*	y*++	y0	y0++	#
1	72.0444	36.5258					A							21
2	**185.1285**	93.0679					L	2410.2111	1205.6092	2393.1846	1197.0959	2392.2006	1196.6039	20
3	**284.1969**	142.6021					V	2297.1271	1149.0672	2280.1005	1140.5539	2279.1165	1140.0619	19
4	397.2809	199.1441					L	2198.0587	1099.5330	2181.0321	1091.0197	2180.0481	1090.5277	18
5	510.3650	255.6861					I	2084.9746	1042.9909	2067.9481	1034.4777	2066.9640	1033.9857	17
6	581.4021	291.2047					A	1971.8905	986.4489	1954.8640	977.9356	1953.8800	977.4436	16
7	728.4705	364.7389					F	1900.8534	950.9304	1883.8269	942.4171	1882.8429	941.9251	15
8	799.5076	400.2575					A	1753.7850	877.3961	1736.7585	868.8829	1735.7744	868.3909	14
9	927.5662	464.2867	910.5397	455.7735			Q	1682.7479	841.8776	1665.7214	833.3643	1664.7373	832.8723	13
10	1090.6295	545.8184	1073.6030	537.3051			Y	***1554.6893***	777.8483	1537.6628	769.3350	1536.6788	768.8430	12
11	1203.7136	602.3604	1186.6871	593.8472			L	***1391.6260***	696.3166	1374.5994	687.8034	1373.6154	687.3114	11
12	1331.7722	666.3897	1314.7456	657.8765			Q	***1278.5419***	639.7746	1261.5154	631.2613	1260.5314	630.7693	10
13	1459.8308	730.4190	1442.8042	721.9057			Q	1150.4834	575.7453	1133.4568	567.2320	1132.4728	566.7400	9
14	1610.8247	805.9160	1593.7981	797.4027			C	***1022.4248***	511.7160	1005.3982	503.2027	1004.4142	502.7107	8
15	1707.8775	854.4424	1690.8509	845.9291			P	***871.4308***	436.2191	854.4043	427.7058	853.4203	427.2138	7
16	1854.9459	927.9766	1837.9193	919.4633			F	***774.3781***	387.6927	757.3515	379.1794	756.3675	378.6874	6
17	1983.9885	992.4979	1966.9619	983.9846	1965.9779	983.4926	E	***627.3097***	314.1585	610.2831	305.6452	609.2991	305.1532	5
18	2099.0154	1050.0113	2081.9889	1041.4981	2081.0048	1041.0061	D	***498.2671***	249.6372	481.2405	241.1239	480.2565	240.6319	4
19	2236.0743	1118.5408	2219.0478	1110.0275	2218.0638	1109.5355	H	***383.2401***	192.1237	366.2136	183.6104			3

**20**	2335.1427	1168.0750	2318.1162	1159.5617	2317.1322	1159.0697	**V**	246.1812	123.5942	229.1547	115.0810			**2**
**21**							**K**	147.1128	74.0600	130.0863	65.5468			**1**

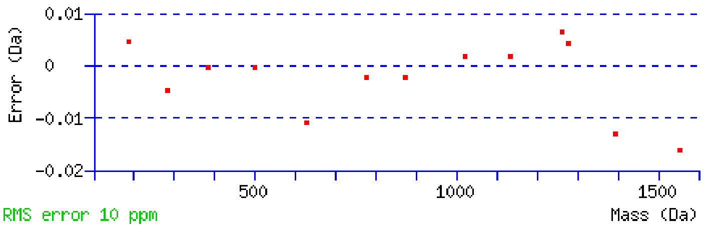

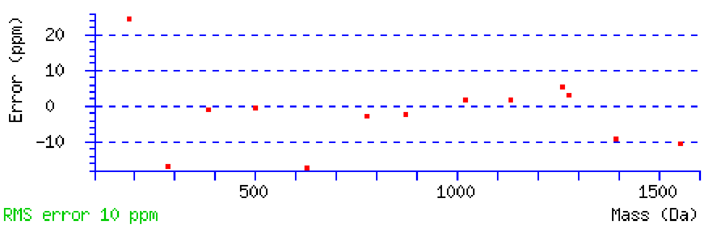

NCBI **BLAST** search of ALVLIAFAQYLQQCPFEDHVK
(Parameters: blastp, nr protein database, expect=20000, no filter, PAM30)
Other BLAST web gateways

All matches to this query

Score	Mr(calc):	Delta	Sequence
74.1	2480.2410	-0.0097	ALVLIAFAQYLQQCPFEDHVK
28.9	2480.2410	-0.0097	ALVLIAFAQYLQQCPFEDHVK

Mascot: http://www.matrixscience.com/

Mascot Search Results

Peptide View

MS/MS Fragmentation of **ALVLIAFAQYLQQCPFEDHVK**
Found in **sp|P02768|ALBU_HUMAN**, Serum albumin OS=Homo sapiens GN=ALB PE=1 SV=2

Match to Query 11533: 2480.240982 from(827.754270,3+) intensity(815731.8125) scans(17117) rtinseconds(3088) index(14415)
Title: 150801_TTSH_Patient_Plasma_37_Spectrum031329_scans__17117_RTINSECONDS=3088
Data file L:\\Ard_TTSH\\T1D\\T150801_TTSH_Patient_Plasma_37.mgf

Click mouse within plot area to zoom in by factor of two about that point

Or, to Da

Label all possible matches ○ Label matches used for scoring ⊙

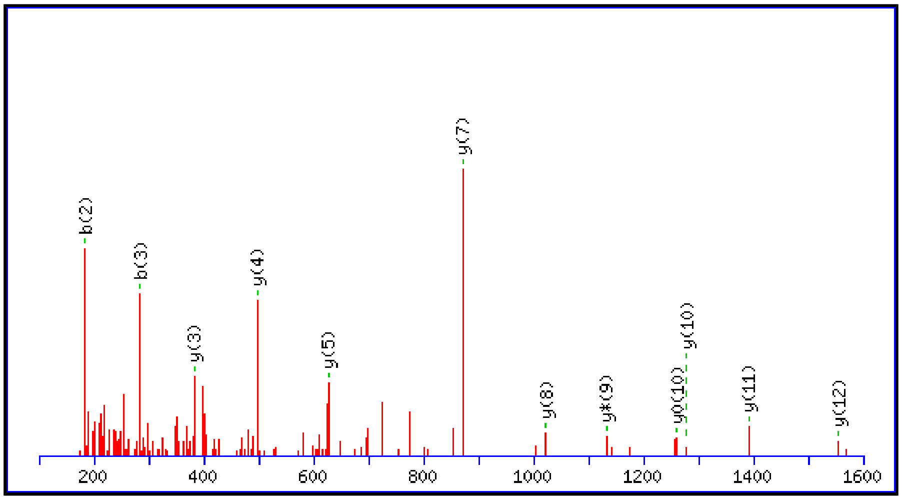

Monoisotopic mass of neutral peptide Mr(calc): 2480.2410
Variable modifications:
C14 : 4Trioxidation (CMWY)
Ions Score: 68 **Expect:** 6e-005
Matches : 12/186 fragment ions using 13 most intense peaks (help)

#	b	b^{++}	b*	b^{*++}	b^{0}	b^{0++}	Seq.	y	y^{++}	y*	y^{*++}	y^{0}	y^{0++}	#
1	72.0444	36.5258					A							21
2	**185.1285**	93.0679					L	2410.2111	1205.6092	2393.1846	1197.0959	2392.2006	1196.6039	20
3	**284.1969**	142.6021					V	2297.1271	1149.0672	2280.1005	1140.5539	2279.1165	1140.0619	19
4	397.2809	199.1441					L	2198.0587	1099.5330	2181.0321	1091.0197	2180.0481	1090.5277	18
5	510.3650	255.6861					I	2084.9746	1042.9909	2067.9481	1034.4777	2066.9640	1033.9857	17
6	581.4021	291.2047					A	1971.8905	986.4489	1954.8640	977.9356	1953.8800	977.4436	16
7	728.4705	364.7389					F	1900.8534	950.9304	1883.8269	942.4171	1882.8429	941.9251	15
8	799.5076	400.2575					A	1753.7850	877.3961	1736.7585	868.8829	1735.7744	868.3909	14
9	927.5662	464.2867	910.5397	455.7735			Q	1682.7479	841.8776	1665.7214	833.3643	1664.7373	832.8723	13
10	1090.6295	545.8184	1073.6030	537.3051			Y	***1554.6893***	777.8483	1537.6628	769.3350	1536.6788	768.8430	12
11	1203.7136	602.3604	1186.6871	593.8472			L	***1391.6260***	696.3166	1374.5994	687.8034	1373.6154	687.3114	11
12	1331.7722	666.3897	1314.7456	657.8765			Q	***1278.5419***	639.7746	1261.5154	631.2613	1260.5314	630.7693	10
13	1459.8308	730.4190	1442.8042	721.9057			Q	1150.4834	575.7453	1133.4568	567.2320	1132.4728	566.7400	9
14	1610.8247	805.9160	1593.7981	797.4027			C	***1022.4248***	511.7160	1005.3982	503.2027	1004.4142	502.7107	8
15	1707.8775	854.4424	1690.8509	845.9291			P	***871.4308***	436.2191	854.4043	427.7058	853.4203	427.2138	7
16	1854.9459	927.9766	1837.9193	919.4633			F	774.3781	387.6927	757.3515	379.1794	756.3675	378.6874	6
17	1983.9885	992.4979	1966.9619	983.9846	1965.9779	983.4926	E	***627.3097***	314.1585	610.2831	305.6452	609.2991	305.1532	5
18	2099.0154	1050.0113	2081.9889	1041.4981	2081.0048	1041.0061	D	***498.2671***	249.6372	481.2405	241.1239	480.2565	240.6319	4
19	2236.0743	1118.5408	2219.0478	1110.0275	2218.0638	1109.5355	H	***383.2401***	192.1237	366.2136	183.6104			3

**20**	2335.1427	1168.0750	2318.1162	1159.5617	2317.1322	1159.0697	**V**	246.1812	123.5942	229.1547	115.0810			**2**
**21**							**K**	147.1128	74.0600	130.0863	65.5468			**1**

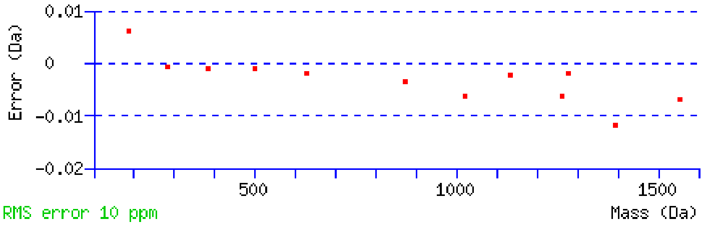

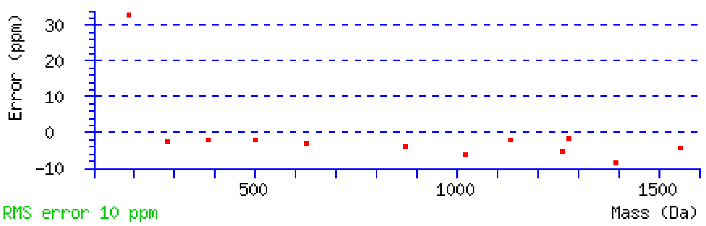

NCBI **BLAST** search of ALVLIAFAQYLQQCPFEDHVK
(Parameters: blastp, nr protein database, expect=20000, no filter, PAM30)
Other BLAST web gateways

All matches to this query

Score	Mr(calc):	Delta	Sequence
68.1	2480.2410	0.0000	ALVLIAFAQYLQQCPFEDHVK
25.5	2480.2410	0.0000	ALVLIAFAQYLQQCPFEDHVK

Mascot Search Results

Peptide View

MS/MS Fragmentation of **ALVLIAFAQYLQQCPFEDHVK**
Found in **sp|P02768|ALBU_HUMAN**, Serum albumin OS=Homo sapiens GN=ALB PE=1 SV=2

Match to Query 11533: 2480.240982 from(827.754270,3+) intensity(815731.8125) scans(17117) rtinseconds(3088) index(14415)
Title: N50801_TTSH_Patient_Plasma_37_Spectrum014850_scans__17117_RTINSECONDS=3088
Data file L:\\Ard_TTSH\\TN1D\\TN50801_TTSH_Patient_Plasma_37.mgf

Click mouse within plot area to zoom in by factor of two about that point

Or, to Da

Label all possible matches Label matches used for scoring

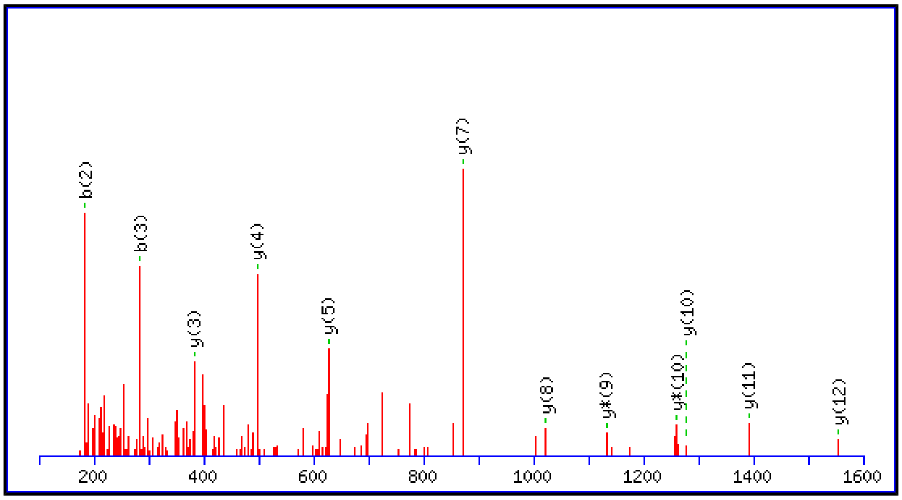

Monoisotopic mass of neutral peptide Mr(calc): 2480.2410
Variable modifications:
C14 : 4Trioxidation (CMWY)
Ions Score: 67 **Expect:** 7.2e-005
Matches : 12/186 fragment ions using 13 most intense peaks (help)

#	b	b^{++}	b*	b^{*++}	b^0	b^{0++}	Seq.	y	y^{++}	y*	y^{*++}	y^0	y^{0++}	#
1	72.0444	36.5258					A							21
2	**185.1285**	93.0679					L	2410.2111	1205.6092	2393.1846	1197.0959	2392.2006	1196.6039	20
3	**284.1969**	142.6021					V	2297.1271	1149.0672	2280.1005	1140.5539	2279.1165	1140.0619	19
4	397.2809	199.1441					L	2198.0587	1099.5330	2181.0321	1091.0197	2180.0481	1090.5277	18
5	510.3650	255.6861					I	2084.9746	1042.9909	2067.9481	1034.4777	2066.9640	1033.9857	17
6	581.4021	291.2047					A	1971.8905	986.4489	1954.8640	977.9356	1953.8800	977.4436	16
7	728.4705	364.7389					F	1900.8534	950.9304	1883.8269	942.4171	1882.8429	941.9251	15
8	799.5076	400.2575					A	1753.7850	877.3961	1736.7585	868.8829	1735.7744	868.3909	14
9	927.5662	464.2867	910.5397	455.7735			Q	1682.7479	841.8776	1665.7214	833.3643	1664.7373	832.8723	13
10	1090.6295	545.8184	1073.6030	537.3051			Y	***1554.6893***	777.8483	1537.6628	769.3350	1536.6788	768.8430	12
11	1203.7136	602.3604	1186.6871	593.8472			L	***1391.6260***	696.3166	1374.5994	687.8034	1373.6154	687.3114	11
12	1331.7722	666.3897	1314.7456	657.8765			Q	***1278.5419***	639.7746	1261.5154	631.2613	1260.5314	630.7693	10
13	1459.8308	730.4190	1442.8042	721.9057			Q	1150.4834	575.7453	1133.4568	567.2320	1132.4728	566.7400	9
14	1610.8247	805.9160	1593.7981	797.4027			C	***1022.4248***	511.7160	1005.3982	503.2027	1004.4142	502.7107	8
15	1707.8775	854.4424	1690.8509	845.9291			P	***871.4308***	436.2191	854.4043	427.7058	853.4203	427.2138	7
16	1854.9459	927.9766	1837.9193	919.4633			F	774.3781	387.6927	757.3515	379.1794	756.3675	378.6874	6
17	1983.9885	992.4979	1966.9619	983.9846	1965.9779	983.4926	E	***627.3097***	314.1585	610.2831	305.6452	609.2991	305.1532	5
18	2099.0154	1050.0113	2081.9889	1041.4981	2081.0048	1041.0061	D	***498.2671***	249.6372	481.2405	241.1239	480.2565	240.6319	4
19	2236.0743	1118.5408	2219.0478	1110.0275	2218.0638	1109.5355	H	***383.2401***	192.1237	366.2136	183.6104			3

**20**	2335.1427	1168.0750	2318.1162	1159.5617	2317.1322	1159.0697	**V**	246.1812	123.5942	229.1547	115.0810			**2**
**21**							**K**	147.1128	74.0600	130.0863	65.5468			**1**

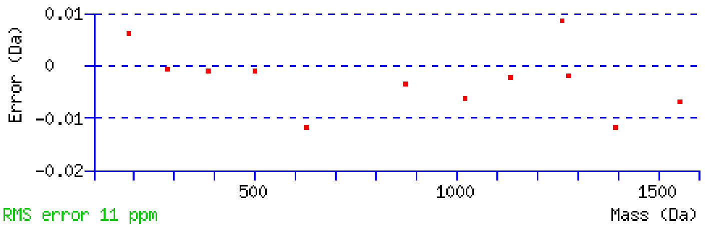

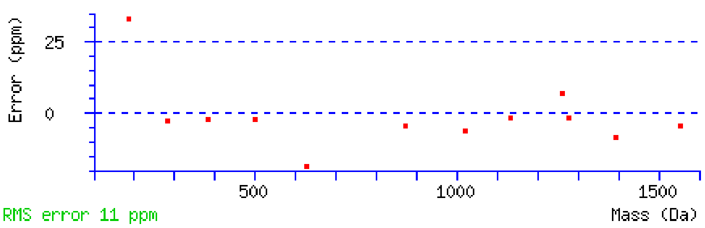

NCBI **BLAST** search of ALVLIAFAQYLQQCPFEDHVK
(Parameters: blastp, nr protein database, expect=20000, no filter, PAM30)
Other BLAST web gateways

All matches to this query

Score	Mr(calc):	Delta	Sequence
67.3	2480.2410	0.0000	ALVLIAFAQYLQQCPFEDHVK
22.3	2480.2410	0.0000	ALVLIAFAQYLQQCPFEDHVK

Mascot: http://www.matrixscience.com/

MATRIX SCIENCE Mascot Search Results

Peptide View

MS/MS Fragmentation of **ALVLIAFAQYLQQCPFEDHVK**
Found in **sp|P02768|ALBU_HUMAN**, Serum albumin OS=Homo sapiens GN=ALB PE=1 SV=2

Match to Query 12475: 2480.238792 from(827.753540,3+) intensity(6294169.0000) scans(18070) rtinseconds(3115) index(15664)
Title: N50818_TTSH_Patient_Plasma_37_Spectrum016281_scans__18070_RTINSECONDS=3115
Data file L:\\Ard_TTSH\\TN1D\\TN50818_TTSH_Patient_Plasma_37.mgf

Click mouse within plot area to zoom in by factor of two about that point

Or, to Da

Label all possible matches ☐ Label matches used for scoring ☐

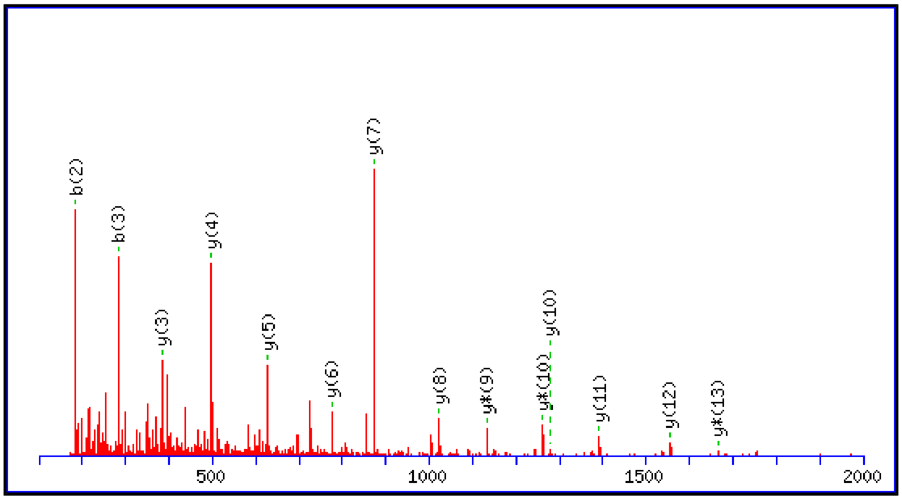

Monoisotopic mass of neutral peptide Mr(calc): 2480.2410
Variable modifications:
C14 : 4Trioxidation (CMWY)
Ions Score: 67 **Expect:** 8.7e-005
Matches : 14/186 fragment ions using 18 most intense peaks (help)

#	b	b++	b*	b*++	b0	b0++	Seq.	y	y++	y*	y*++	y0	y0++	#
1	72.0444	36.5258					A							21
2	**185.1285**	93.0679					L	2410.2111	1205.6092	2393.1846	1197.0959	2392.2006	1196.6039	20
3	**284.1969**	142.6021					V	2297.1271	1149.0672	2280.1005	1140.5539	2279.1165	1140.0619	19
4	397.2809	199.1441					L	2198.0587	1099.5330	2181.0321	1091.0197	2180.0481	1090.5277	18
5	510.3650	255.6861					I	2084.9746	1042.9909	2067.9481	1034.4777	2066.9640	1033.9857	17
6	581.4021	291.2047					A	1971.8905	986.4489	1954.8640	977.9356	1953.8800	977.4436	16
7	728.4705	364.7389					F	1900.8534	950.9304	1883.8269	942.4171	1882.8429	941.9251	15
8	799.5076	400.2575					A	1753.7850	877.3961	1736.7585	868.8829	1735.7744	868.3909	14
9	927.5662	464.2867	910.5397	455.7735			Q	1682.7479	841.8776	1665.7214	833.3643	1664.7373	832.8723	13
10	1090.6295	545.8184	1073.6030	537.3051			Y	***1554.6893***	777.8483	1537.6628	769.3350	1536.6788	768.8430	12
11	1203.7136	602.3604	1186.6871	593.8472			L	***1391.6260***	696.3166	1374.5994	687.8034	1373.6154	687.3114	11
12	1331.7722	666.3897	1314.7456	657.8765			Q	***1278.5419***	639.7746	1261.5154	631.2613	1260.5314	630.7693	10
13	1459.8308	730.4190	1442.8042	721.9057			Q	1150.4834	575.7453	1133.4568	567.2320	1132.4728	566.7400	9
14	1610.8247	805.9160	1593.7981	797.4027			C	***1022.4248***	511.7160	1005.3982	503.2027	1004.4142	502.7107	8
15	1707.8775	854.4424	1690.8509	845.9291			P	***871.4308***	436.2191	854.4043	427.7058	853.4203	427.2138	7
16	1854.9459	927.9766	1837.9193	919.4633			F	***774.3781***	387.6927	757.3515	379.1794	756.3675	378.6874	6
17	1983.9885	992.4979	1966.9619	983.9846	1965.9779	983.4926	E	***627.3097***	314.1585	610.2831	305.6452	609.2991	305.1532	5
18	2099.0154	1050.0113	2081.9889	1041.4981	2081.0048	1041.0061	D	***498.2671***	249.6372	481.2405	241.1239	480.2565	240.6319	4
19	2236.0743	1118.5408	2219.0478	1110.0275	2218.0638	1109.5355	H	***383.2401***	192.1237	366.2136	183.6104			3

**20**	2335.1427	1168.0750	2318.1162	1159.5617	2317.1322	1159.0697	**V**	246.1812	123.5942	229.1547	115.0810			**2**
**21**							**K**	147.1128	74.0600	130.0863	65.5468			**1**

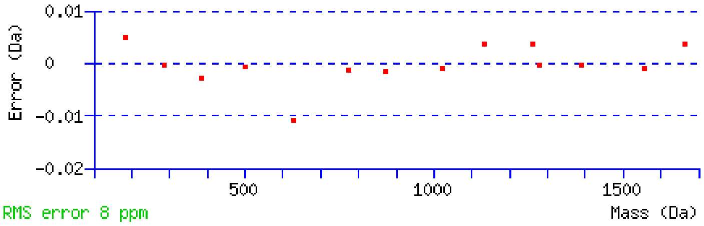

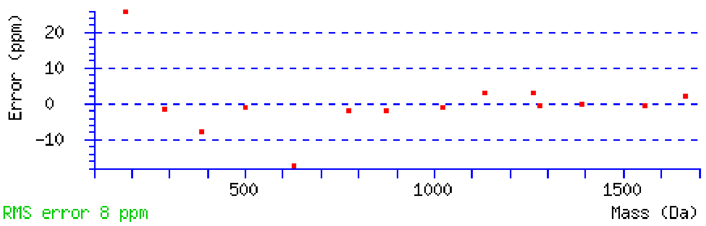

NCBI **BLAST** search of ALVLIAFAQYLQQCPFEDHVK
(Parameters: blastp, nr protein database, expect=20000, no filter, PAM30)
Other BLAST web gateways

All matches to this query

Score	Mr(calc):	Delta	Sequence
66.5	2480.2410	-0.0022	ALVLIAFAQYLQQCPFEDHVK
27.5	2480.2410	-0.0022	ALVLIAFAQYLQQCPFEDHVK

Mascot: http://www.matrixscience.com/

Mascot Search Results

Peptide View

MS/MS Fragmentation of **ALVLIAFAQYLQQCPFEDHVK**
Found in **sp|P02768|ALBU_HUMAN**, Serum albumin OS=Homo sapiens GN=ALB PE=1 SV=2

Match to Query 12476: 2480.239608 from(1241.127080,2+) intensity(10307705.0000) scans(18076) rtinseconds(3116) index(15668)
Title: N50818_TTSH_Patient_Plasma_37_Spectrum016286_scans__18076_RTINSECONDS=3116
Data file L:\\Ard_TTSH\\TN1D\\TN50818_TTSH_Patient_Plasma_37.mgf

Click mouse within plot area to zoom in by factor of two about that point

Or, to Da

Label all possible matches Label matches used for scoring

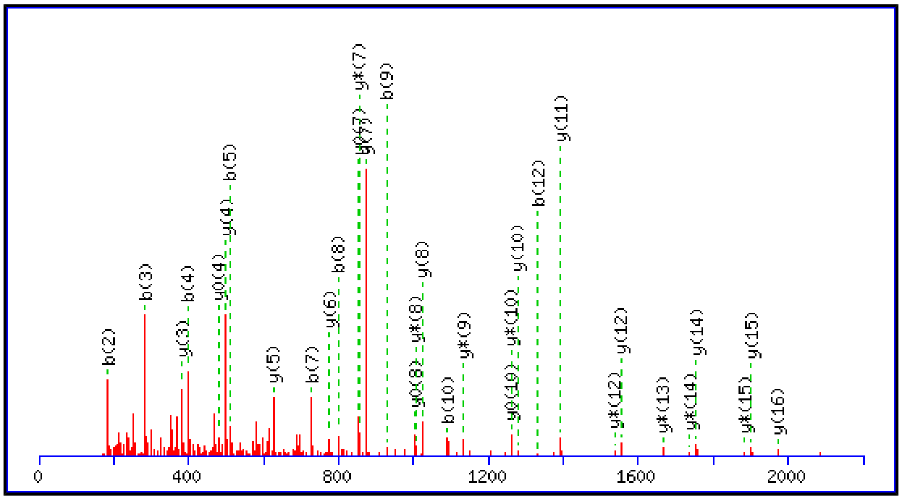

Monoisotopic mass of neutral peptide Mr(calc): 2480.2410
Variable modifications:
C14 : 4Trioxidation (CMWY)
Ions Score: 65 **Expect:** 0.00012
Matches : 33/186 fragment ions using 71 most intense peaks (help)

#	b	b^{++}	b*	b^{*++}	b^0	b^{0++}	Seq.	y	y^{++}	y*	y^{*++}	y^0	y^{0++}	#
1	72.0444	36.5258					A							21
2	***185.1285***	93.0679					L	2410.2111	1205.6092	2393.1846	1197.0959	2392.2006	1196.6039	20
3	***284.1969***	142.6021					V	2297.1271	1149.0672	2280.1005	1140.5539	2279.1165	1140.0619	19
4	***397.2809***	199.1441					L	2198.0587	1099.5330	2181.0321	1091.0197	2180.0481	1090.5277	18
5	***510.3650***	255.6861					I	2084.9746	1042.9909	2067.9481	1034.4777	2066.9640	1033.9857	17
6	581.4021	291.2047					A	***1971.8905***	986.4489	1954.8640	977.9356	1953.8800	977.4436	16
7	***728.4705***	364.7389					F	***1900.8534***	950.9304	1883.8269	942.4171	1882.8429	941.9251	15
8	***799.5076***	400.2575					A	***1753.7850***	877.3961	1736.7585	868.8829	1735.7744	868.3909	14
9	***927.5662***	464.2867	910.5397	455.7735			Q	1682.7479	841.8776	1665.7214	833.3643	1664.7373	832.8723	13
10	***1090.6295***	545.8184	1073.6030	537.3051			Y	***1554.6893***	777.8483	1537.6628	769.3350	1536.6788	768.8430	12
11	1203.7136	602.3604	1186.6871	593.8472			L	***1391.6260***	696.3166	1374.5994	687.8034	1373.6154	687.3114	11
12	***1331.7722***	666.3897	1314.7456	657.8765			Q	***1278.5419***	639.7746	1261.5154	631.2613	1260.5314	630.7693	10
13	1459.8308	730.4190	1442.8042	721.9057			Q	1150.4834	575.7453	1133.4568	567.2320	1132.4728	566.7400	9
14	1610.8247	805.9160	1593.7981	797.4027			C	***1022.4248***	511.7160	1005.3982	503.2027	1004.4142	502.7107	8
15	1707.8775	854.4424	1690.8509	845.9291			P	***871.4308***	436.2191	854.4043	427.7058	853.4203	427.2138	7
16	1854.9459	927.9766	1837.9193	919.4633			F	***774.3781***	387.6927	757.3515	379.1794	756.3675	378.6874	6
17	1983.9885	992.4979	1966.9619	983.9846	1965.9779	983.4926	E	***627.3097***	314.1585	610.2831	305.6452	609.2991	305.1532	5
18	2099.0154	1050.0113	2081.9889	1041.4981	2081.0048	1041.0061	D	***498.2671***	249.6372	481.2405	241.1239	480.2565	240.6319	4
19	2236.0743	1118.5408	2219.0478	1110.0275	2218.0638	1109.5355	H	***383.2401***	192.1237	366.2136	183.6104			3

**20**	2335.1427	1168.0750	2318.1162	1159.5617	2317.1322	1159.0697	**V**	246.1812	123.5942	229.1547	115.0810			**2**
**21**							**K**	147.1128	74.0600	130.0863	65.5468			**1**

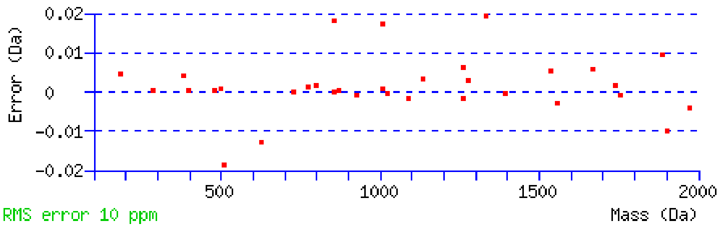

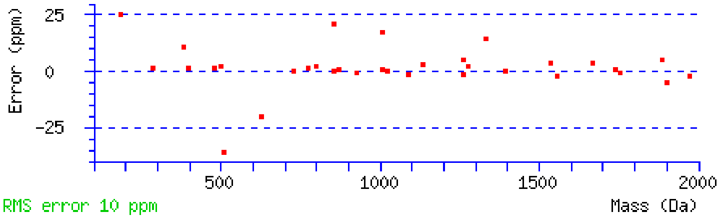

NCBI **BLAST** search of ALVLIAFAQYLQQCPFEDHVK
(Parameters: blastp, nr protein database, expect=20000, no filter, PAM30)
Other BLAST web gateways

All matches to this query

Score	Mr(calc):	Delta	Sequence
65.3	2480.2410	-0.0014	ALVLIAFAQYLQQCPFEDHVK
29.3	2480.2410	-0.0014	ALVLIAFAQYLQQCPFEDHVK

MATRIX SCIENCE Mascot Search Results

Peptide View

MS/MS Fragmentation of **ALVLIAFAQYLQQCPFEDHVK**
Found in **sp|P02768|ALBU_HUMAN**, Serum albumin OS=Homo sapiens GN=ALB PE=1 SV=2

Match to Query 10890: 2480.239722 from(827.753850,3+) intensity(10857396.0000) scans(16243) rtinseconds(3173) index(13005)
Title: 150801_TTSH_Patient_Plasma_76_Spectrum028166_scans__16243_RTINSECONDS=3173
Data file L:\\Ard_TTSH\\T1D\\T150801_TTSH_Patient_Plasma_76.mgf

Click mouse within plot area to zoom in by factor of two about that point
Or, [Plot from] 100 to 1600 Da [Full range]
Label all possible matches ○ Label matches used for scoring ⊙

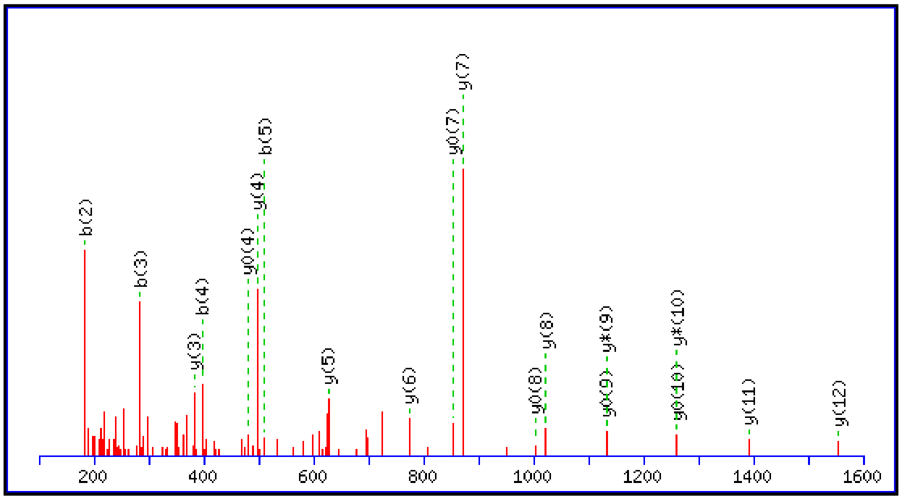

Monoisotopic mass of neutral peptide Mr(calc): 2480.2410
Variable modifications:
C14 : 4Trioxidation (CMWY)
Ions Score: 65 **Expect:** 0.00013
Matches : 19/186 fragment ions using 23 most intense peaks (help)

#	b	b++	b*	b*++	b0	b0++	Seq.	y	y++	y*	y*++	y0	y0++	#
1	72.0444	36.5258					A							21
2	***185.1285***	93.0679					L	2410.2111	1205.6092	2393.1846	1197.0959	2392.2006	1196.6039	20
3	***284.1969***	142.6021					V	2297.1271	1149.0672	2280.1005	1140.5539	2279.1165	1140.0619	19
4	***397.2809***	199.1441					L	2198.0587	1099.5330	2181.0321	1091.0197	2180.0481	1090.5277	18
5	***510.3650***	255.6861					I	2084.9746	1042.9909	2067.9481	1034.4777	2066.9640	1033.9857	17
6	581.4021	291.2047					A	1971.8905	986.4489	1954.8640	977.9356	1953.8800	977.4436	16
7	728.4705	364.7389					F	1900.8534	950.9304	1883.8269	942.4171	1882.8429	941.9251	15
8	799.5076	400.2575					A	1753.7850	877.3961	1736.7585	868.8829	1735.7744	868.3909	14
9	927.5662	464.2867	910.5397	455.7735			Q	1682.7479	841.8776	1665.7214	833.3643	1664.7373	832.8723	13
10	1090.6295	545.8184	1073.6030	537.3051			Y	***1554.6893***	777.8483	1537.6628	769.3350	1536.6788	768.8430	12
11	1203.7136	602.3604	1186.6871	593.8472			L	***1391.6260***	696.3166	1374.5994	687.8034	1373.6154	687.3114	11
12	1331.7722	666.3897	1314.7456	657.8765			Q	1278.5419	639.7746	1261.5154	631.2613	1260.5314	630.7693	10
13	1459.8308	730.4190	1442.8042	721.9057			Q	1150.4834	575.7453	1133.4568	567.2320	1132.4728	566.7400	9
14	1610.8247	805.9160	1593.7981	797.4027			C	***1022.4248***	511.7160	1005.3982	503.2027	1004.4142	502.7107	8
15	1707.8775	854.4424	1690.8509	845.9291			P	***871.4308***	436.2191	854.4043	427.7058	853.4203	427.2138	7
16	1854.9459	927.9766	1837.9193	919.4633			F	***774.3781***	387.6927	757.3515	379.1794	756.3675	378.6874	6
17	1983.9885	992.4979	1966.9619	983.9846	1965.9779	983.4926	E	***627.3097***	314.1585	610.2831	305.6452	609.2991	305.1532	5
18	2099.0154	1050.0113	2081.9889	1041.4981	2081.0048	1041.0061	D	***498.2671***	249.6372	481.2405	241.1239	480.2565	240.6319	4
19	2236.0743	1118.5408	2219.0478	1110.0275	2218.0638	1109.5355	H	***383.2401***	192.1237	366.2136	183.6104			3

**20**	2335.1427	1168.0750	2318.1162	1159.5617	2317.1322	1159.0697	**V**	246.1812	123.5942	229.1547	115.0810			**2**
**21**							**K**	147.1128	74.0600	130.0863	65.5468			**1**

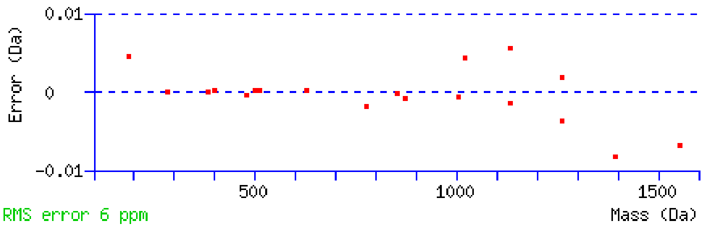

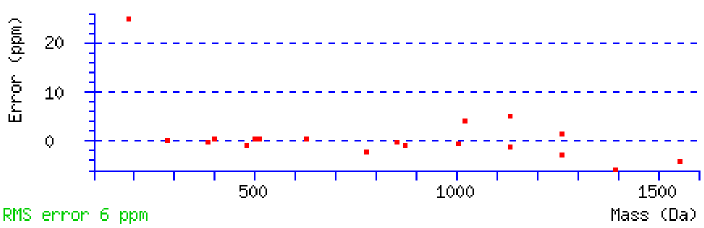

NCBI **BLAST** search of ALVLIAFAQYLQQCPFEDHVK
(Parameters: blastp, nr protein database, expect=20000, no filter, PAM30)
Other BLAST web gateways

All matches to this query

Score	Mr(calc):	Delta	Sequence
64.7	2480.2410	-0.0012	ALVLIAFAQYLQQCPFEDHVK
34.0	2480.2410	-0.0012	ALVLIAFAQYLQQCPFEDHVK
1.9	2479.2549	0.9848	ALVILAKGAEEMETVIPVDVMR

Mascot: http://www.matrixscience.com/

Mascot Search Results

Peptide View

MS/MS Fragmentation of **ALVLIAFAQYLQQCPFEDHVK**
Found in **sp|P02768|ALBU_HUMAN**, Serum albumin OS=Homo sapiens GN=ALB PE=1 SV=2

Match to Query 11946: 2480.236188 from(1241.125370,2+) intensity(11179564.0000) scans(17967) rtinseconds(3113) index(15229)
Title: N50818_TTSH_Patient_Plasma_34_Spectrum015961_scans__17967_RTINSECONDS=3113
Data file L:\\Ard_TTSH\\TN1D\\TN50818_TTSH_Patient_Plasma_34.mgf

Click mouse within plot area to zoom in by factor of two about that point

Or, to Da

Label all possible matches ☐ Label matches used for scoring ☐

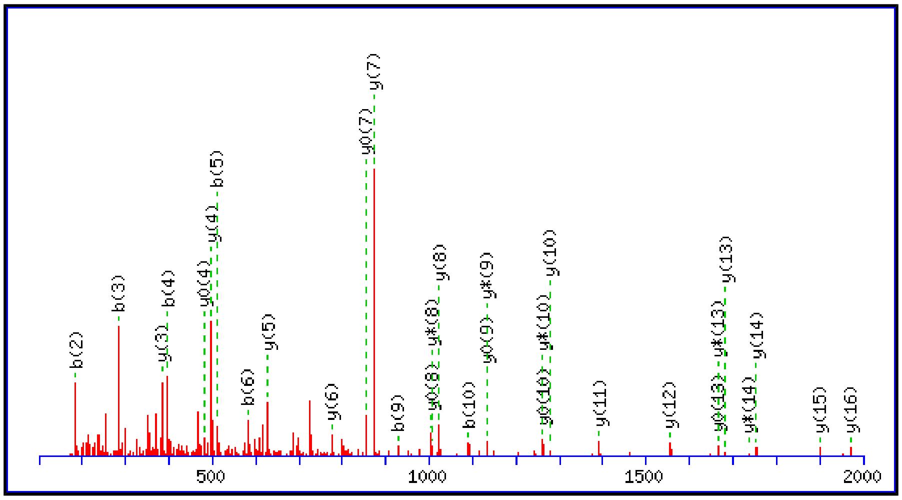

Monoisotopic mass of neutral peptide Mr(calc): 2480.2410
Variable modifications:
C14 : 4Trioxidation (CMWY)
Ions Score: 64 **Expect:** 0.00016
Matches : 31/186 fragment ions using 66 most intense peaks (help)

#	b	b++	b*	b*++	b0	b0++	Seq.	y	y++	y*	y*++	y0	y0++	#
1	72.0444	36.5258					A							21
2	**185.1285**	93.0679					L	2410.2111	1205.6092	2393.1846	1197.0959	2392.2006	1196.6039	20
3	**284.1969**	142.6021					V	2297.1271	1149.0672	2280.1005	1140.5539	2279.1165	1140.0619	19
4	**397.2809**	199.1441					L	2198.0587	1099.5330	2181.0321	1091.0197	2180.0481	1090.5277	18
5	**510.3650**	255.6861					I	2084.9746	1042.9909	2067.9481	1034.4777	2066.9640	1033.9857	17
6	**581.4021**	291.2047					A	***1971.8905***	986.4489	1954.8640	977.9356	1953.8800	977.4436	16
7	728.4705	364.7389					F	***1900.8534***	950.9304	1883.8269	942.4171	1882.8429	941.9251	15
8	799.5076	400.2575					A	***1753.7850***	877.3961	1736.7585	868.8829	1735.7744	868.3909	14
9	**927.5662**	464.2867	910.5397	455.7735			Q	***1682.7479***	841.8776	1665.7214	833.3643	1664.7373	832.8723	13
10	**1090.6295**	545.8184	1073.6030	537.3051			Y	***1554.6893***	777.8483	1537.6628	769.3350	1536.6788	768.8430	12
11	1203.7136	602.3604	1186.6871	593.8472			L	***1391.6260***	696.3166	1374.5994	687.8034	1373.6154	687.3114	11
12	1331.7722	666.3897	1314.7456	657.8765			Q	***1278.5419***	639.7746	1261.5154	631.2613	1260.5314	630.7693	10
13	1459.8308	730.4190	1442.8042	721.9057			Q	1150.4834	575.7453	1133.4568	567.2320	1132.4728	566.7400	9
14	1610.8247	805.9160	1593.7981	797.4027			C	***1022.4248***	511.7160	1005.3982	503.2027	1004.4142	502.7107	8
15	1707.8775	854.4424	1690.8509	845.9291			P	***871.4308***	436.2191	854.4043	427.7058	853.4203	427.2138	7
16	1854.9459	927.9766	1837.9193	919.4633			F	***774.3781***	387.6927	757.3515	379.1794	756.3675	378.6874	6
17	1983.9885	992.4979	1966.9619	983.9846	1965.9779	983.4926	E	***627.3097***	314.1585	610.2831	305.6452	609.2991	305.1532	5
18	2099.0154	1050.0113	2081.9889	1041.4981	2081.0048	1041.0061	D	***498.2671***	249.6372	481.2405	241.1239	480.2565	240.6319	4
19	2236.0743	1118.5408	2219.0478	1110.0275	2218.0638	1109.5355	H	***383.2401***	192.1237	366.2136	183.6104			3

**20**	2335.1427	1168.0750	2318.1162	1159.5617	2317.1322	1159.0697	**V**	246.1812	123.5942	229.1547	115.0810			**2**
**21**							**K**	147.1128	74.0600	130.0863	65.5468			**1**

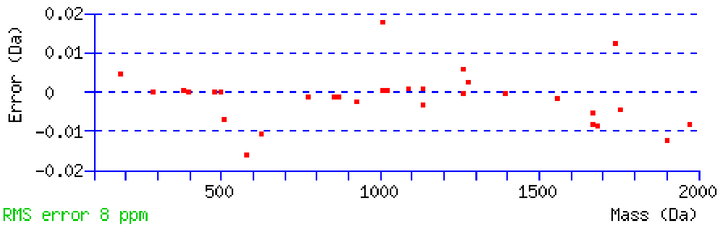

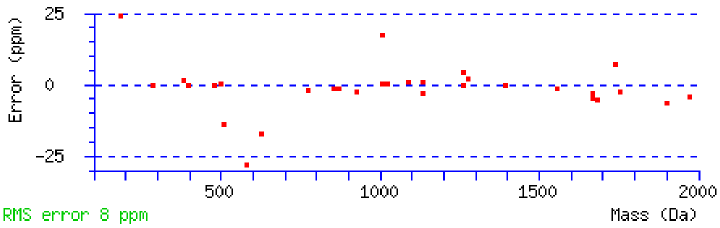

NCBI **BLAST** search of ALVLIAFAQYLQQCPFEDHVK
(Parameters: blastp, nr protein database, expect=20000, no filter, PAM30)
Other BLAST web gateways

All matches to this query

Score	Mr(calc):	Delta	Sequence
64.1	2480.2410	-0.0048	ALVLIAFAQYLQQCPFEDHVK
30.3	2480.2410	-0.0048	ALVLIAFAQYLQQCPFEDHVK

Mascot: http://www.matrixscience.com/

MATRIX SCIENCE Mascot Search Results

Peptide View

MS/MS Fragmentation of **ALVLIAFAQYLQQCPFEDHVK**
Found in **sp|P02768|ALBU_HUMAN**, Serum albumin OS=Homo sapiens GN=ALB PE=1 SV=2

Match to Query 11573: 2480.234968 from(1241.124760,2+) intensity(1543558.2500) scans(17247) rtinseconds(3129) index(14564)
Title: 150825_TTSH_Patient_Plasma_62_Spectrum031169_scans__17247_RTINSECONDS=3129
Data file L:\\Ard_TTSH\\T1D\\T150825_TTSH_Patient_Plasma_62.mgf

Click mouse within plot area to zoom in by factor of two about that point
Or, Plot from 100 to 1700 Da Full range
Label all possible matches ○ Label matches used for scoring ⦿

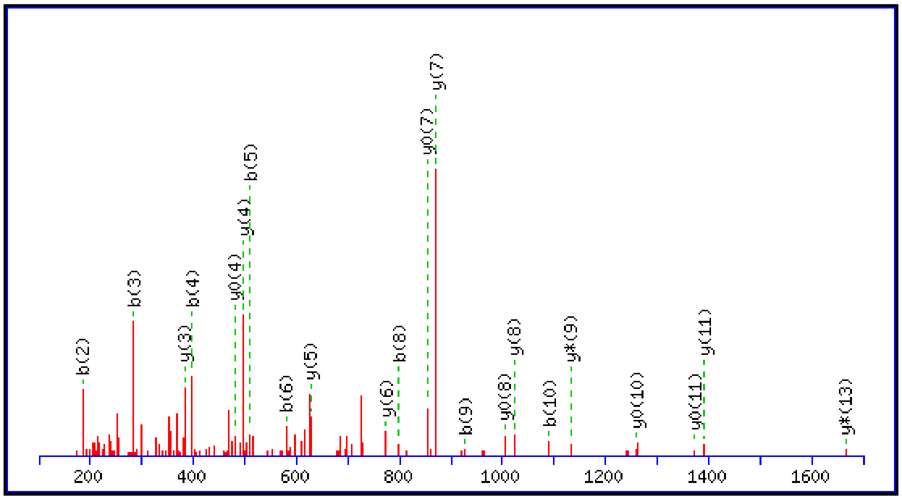

Monoisotopic mass of neutral peptide Mr(calc): 2480.2410
Variable modifications:
C14 : 4Trioxidation (CMWY)
Ions Score: 63 **Expect:** 0.0002
Matches : 22/186 fragment ions using 35 most intense peaks (help)

#	b	b++	b*	b*++	b0	b0++	Seq.	y	y++	y*	y*++	y0	y0++	#
1	72.0444	36.5258					A							21
2	**185.1285**	93.0679					L	2410.2111	1205.6092	2393.1846	1197.0959	2392.2006	1196.6039	20
3	**284.1969**	142.6021					V	2297.1271	1149.0672	2280.1005	1140.5539	2279.1165	1140.0619	19
4	**397.2809**	199.1441					L	2198.0587	1099.5330	2181.0321	1091.0197	2180.0481	1090.5277	18
5	**510.3650**	255.6861					I	2084.9746	1042.9909	2067.9481	1034.4777	2066.9640	1033.9857	17
6	**581.4021**	291.2047					A	1971.8905	986.4489	1954.8640	977.9356	1953.8800	977.4436	16
7	728.4705	364.7389					F	1900.8534	950.9304	1883.8269	942.4171	1882.8429	941.9251	15
8	**799.5076**	400.2575					A	1753.7850	877.3961	1736.7585	868.8829	1735.7744	868.3909	14
9	**927.5662**	464.2867	910.5397	455.7735			Q	1682.7479	841.8776	1665.7214	833.3643	1664.7373	832.8723	13
10	**1090.6295**	545.8184	1073.6030	537.3051			Y	1554.6893	777.8483	1537.6628	769.3350	1536.6788	768.8430	12
11	1203.7136	602.3604	1186.6871	593.8472			L	**1391.6260**	696.3166	1374.5994	687.8034	1373.6154	687.3114	11
12	1331.7722	666.3897	1314.7456	657.8765			Q	1278.5419	639.7746	1261.5154	631.2613	1260.5314	630.7693	10
13	1459.8308	730.4190	1442.8042	721.9057			Q	1150.4834	575.7453	1133.4568	567.2320	1132.4728	566.7400	9
14	1610.8247	805.9160	1593.7981	797.4027			C	**1022.4248**	511.7160	1005.3982	503.2027	1004.4142	502.7107	8
15	1707.8775	854.4424	1690.8509	845.9291			P	**871.4308**	436.2191	854.4043	427.7058	853.4203	427.2138	7
16	1854.9459	927.9766	1837.9193	919.4633			F	**774.3781**	387.6927	757.3515	379.1794	756.3675	378.6874	6
17	1983.9885	992.4979	1966.9619	983.9846	1965.9779	983.4926	E	**627.3097**	314.1585	610.2831	305.6452	609.2991	305.1532	5
18	2099.0154	1050.0113	2081.9889	1041.4981	2081.0048	1041.0061	D	**498.2671**	249.6372	481.2405	241.1239	480.2565	240.6319	4
19	2236.0743	1118.5408	2219.0478	1110.0275	2218.0638	1109.5355	H	**383.2401**	192.1237	366.2136	183.6104			3

**20**	2335.1427	1168.0750	2318.1162	1159.5617	2317.1322	1159.0697	**V**	246.1812	123.5942	229.1547	115.0810			**2**
**21**							**K**	147.1128	74.0600	130.0863	65.5468			**1**

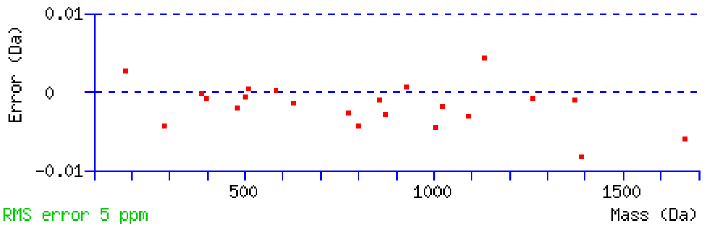

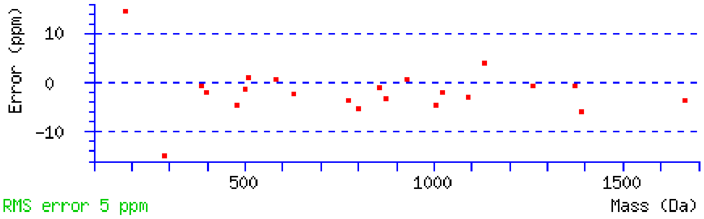

NCBI **BLAST** search of ALVLIAFAQYLQQCPFEDHVK
(Parameters: blastp, nr protein database, expect=20000, no filter, PAM30)
Other BLAST web gateways

All matches to this query

Score	Mr(calc):	Delta	Sequence
63.1	2480.2410	-0.0060	ALVLIAFAQYLQQCPFEDHVK
35.4	2480.2410	-0.0060	ALVLIAFAQYLQQCPFEDHVK
0.7	2479.2549	0.9800	ALVILAKGAEEMETVIPVDVMR

Mascot: http://www.matrixscience.com/

Mascot Search Results

Peptide View

MS/MS Fragmentation of **ALVLIAFAQYLQQCPFEDHVK**
Found in **sp|P02768|ALBU_HUMAN**, Serum albumin OS=Homo sapiens GN=ALB PE=1 SV=2

Match to Query 10889: 2480.236668 from(1241.125610,2+) intensity(929313.1250) scans(16228) rtinseconds(3170) index(12991)
Title: N50801_TTSH_Patient_Plasma_76_Spectrum013341_scans__16228_RTINSECONDS=3170
Data file L:\\Ard_TTSH\\TN1D\\TN50801_TTSH_Patient_Plasma_76.mgf

Click mouse within plot area to zoom in by factor of two about that point

Or, to Da

Label all possible matches Label matches used for scoring

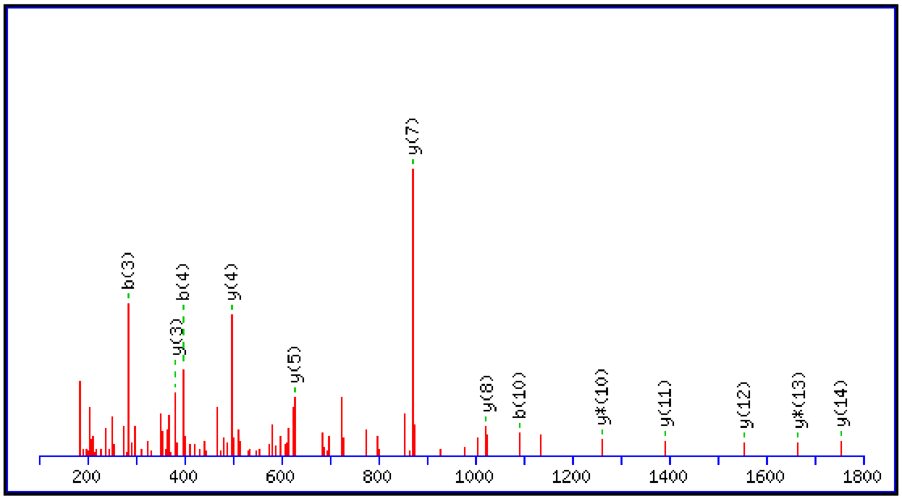

Monoisotopic mass of neutral peptide Mr(calc): 2480.2410
Variable modifications:
C14 : 4Trioxidation (CMWY)
Ions Score: 63 **Expect:** 0.00021
Matches : 13/186 fragment ions using 15 most intense peaks (help)

#	b	b^{++}	b*	b^{*++}	b^0	b^{0++}	Seq.	y	y^{++}	y*	y^{*++}	y^0	y^{0++}	#
1	72.0444	36.5258					A							21
2	185.1285	93.0679					L	2410.2111	1205.6092	2393.1846	1197.0959	2392.2006	1196.6039	20
3	**284.1969**	142.6021					V	2297.1271	1149.0672	2280.1005	1140.5539	2279.1165	1140.0619	19
4	**397.2809**	199.1441					L	2198.0587	1099.5330	2181.0321	1091.0197	2180.0481	1090.5277	18
5	510.3650	255.6861					I	2084.9746	1042.9909	2067.9481	1034.4777	2066.9640	1033.9857	17
6	581.4021	291.2047					A	1971.8905	986.4489	1954.8640	977.9356	1953.8800	977.4436	16
7	728.4705	364.7389					F	1900.8534	950.9304	1883.8269	942.4171	1882.8429	941.9251	15
8	799.5076	400.2575					A	***1753.7850***	877.3961	1736.7585	868.8829	1735.7744	868.3909	14
9	927.5662	464.2867	910.5397	455.7735			Q	1682.7479	841.8776	1665.7214	833.3643	1664.7373	832.8723	13
10	**1090.6295**	545.8184	1073.6030	537.3051			Y	***1554.6893***	777.8483	1537.6628	769.3350	1536.6788	768.8430	12
11	1203.7136	602.3604	1186.6871	593.8472			L	***1391.6260***	696.3166	1374.5994	687.8034	1373.6154	687.3114	11
12	1331.7722	666.3897	1314.7456	657.8765			Q	1278.5419	639.7746	1261.5154	631.2613	1260.5314	630.7693	10
13	1459.8308	730.4190	1442.8042	721.9057			Q	1150.4834	575.7453	1133.4568	567.2320	1132.4728	566.7400	9
14	1610.8247	805.9160	1593.7981	797.4027			C	***1022.4248***	511.7160	1005.3982	503.2027	1004.4142	502.7107	8
15	1707.8775	854.4424	1690.8509	845.9291			P	***871.4308***	436.2191	854.4043	427.7058	853.4203	427.2138	7
16	1854.9459	927.9766	1837.9193	919.4633			F	774.3781	387.6927	757.3515	379.1794	756.3675	378.6874	6
17	1983.9885	992.4979	1966.9619	983.9846	1965.9779	983.4926	E	***627.3097***	314.1585	610.2831	305.6452	609.2991	305.1532	5
18	2099.0154	1050.0113	2081.9889	1041.4981	2081.0048	1041.0061	D	***498.2671***	249.6372	481.2405	241.1239	480.2565	240.6319	4
19	2236.0743	1118.5408	2219.0478	1110.0275	2218.0638	1109.5355	H	***383.2401***	192.1237	366.2136	183.6104			3

**20**	2335.1427	1168.0750	2318.1162	1159.5617	2317.1322	1159.0697	**V**	246.1812	123.5942	229.1547	115.0810			**2**
**21**							**K**	147.1128	74.0600	130.0863	65.5468			**1**

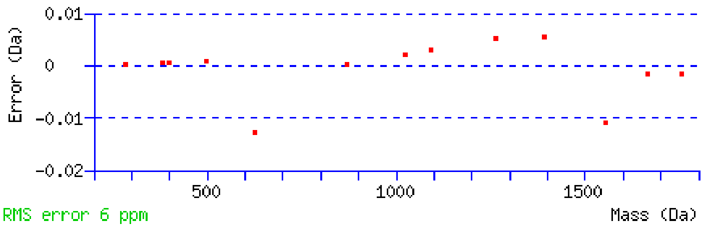

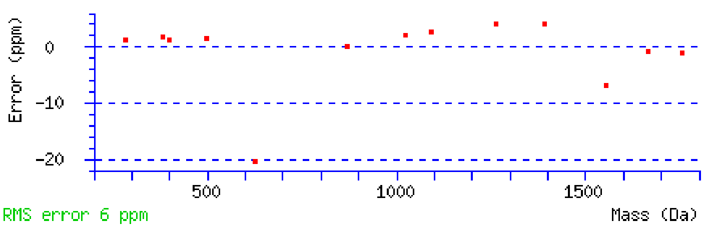

NCBI **BLAST** search of ALVLIAFAQYLQQCPFEDHVK
(Parameters: blastp, nr protein database, expect=20000, no filter, PAM30)
Other BLAST web gateways

All matches to this query

Score	Mr(calc):	Delta	Sequence
62.7	2480.2410	-0.0043	ALVLIAFAQYLQQCPFEDHVK
30.7	2480.2410	-0.0043	ALVLIAFAQYLQQCPFEDHVK

Mascot: http://www.matrixscience.com/

MATRIX SCIENCE Mascot Search Results

Peptide View

MS/MS Fragmentation of **ALVLIAFAQYLQQCPFEDHVK**
Found in **sp|P02768|ALBU_HUMAN**, Serum albumin OS=Homo sapiens GN=ALB PE=1 SV=2

Match to Query 12171: 2489.271012 from(830.764280,3+) intensity(11636153.0000) scans(17479) rtinseconds(3115) index(14772)
Title: 150825_TTSH_Patient_Plasma_75_Spectrum031822_scans__17479_RTINSECONDS=3115
Data file L:\\Ard_TTSH\\T1D\\T150825_TTSH_Patient_Plasma_75.mgf

Click mouse within plot area to zoom in by factor of two about that point
Or, Plot from 0 to 2400 Da Full range
Label all possible matches ○ Label matches used for scoring ⊙

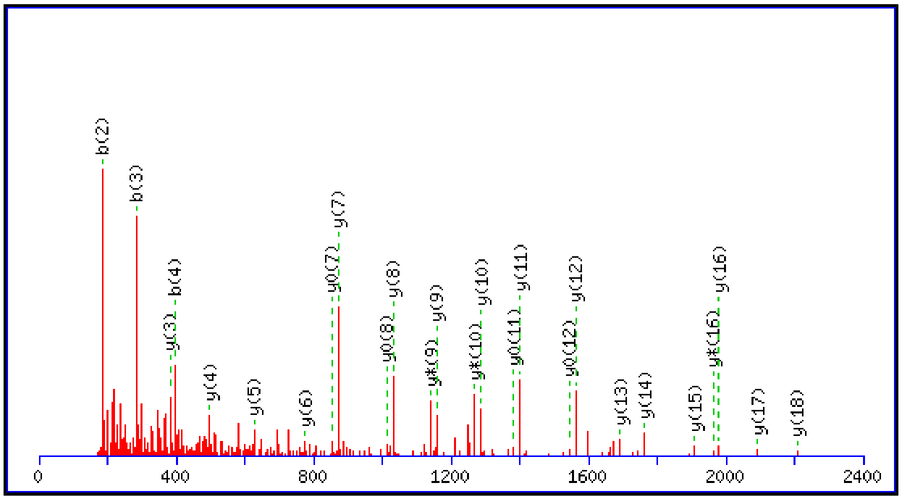

Monoisotopic mass of neutral peptide Mr(calc): 2489.2777
Variable modifications:
C14 : Carbamidomethyl (C)
Ions Score: 133 **Expect:** 1.9e-011
Matches : 26/186 fragment ions using 37 most intense peaks (help)

#	b	b^{++}	b*	b^{*++}	b^0	b^{0++}	Seq.	y	y^{++}	y*	y^{*++}	y^0	y^{0++}	#
1	72.0444	36.5258					A							21
2	**185.1285**	93.0679					L	2419.2479	1210.1276	2402.2213	1201.6143	2401.2373	1201.1223	20
3	**284.1969**	142.6021					V	2306.1638	1153.5855	2289.1373	1145.0723	2288.1532	1144.5803	19
4	**397.2809**	199.1441					L	***2207.0954***	1104.0513	2190.0688	1095.5381	2189.0848	1095.0461	18
5	510.3650	255.6861					I	***2094.0113***	1047.5093	2076.9848	1038.9960	2076.0008	1038.5040	17
6	581.4021	291.2047					A	***1980.9273***	990.9673	1963.9007	982.4540	1962.9167	981.9620	16
7	728.4705	364.7389					F	***1909.8901***	955.4487	1892.8636	946.9354	1891.8796	946.4434	15
8	799.5076	400.2575					A	***1762.8217***	881.9145	1745.7952	873.4012	1744.8112	872.9092	14
9	927.5662	464.2867	910.5397	455.7735			Q	***1691.7846***	846.3959	1674.7581	837.8827	1673.7741	837.3907	13
10	1090.6295	545.8184	1073.6030	537.3051			Y	***1563.7260***	782.3667	1546.6995	773.8534	1545.7155	773.3614	12
11	1203.7136	602.3604	1186.6871	593.8472			L	***1400.6627***	700.8350	1383.6362	692.3217	1382.6521	691.8297	11
12	1331.7722	666.3897	1314.7456	657.8765			Q	***1287.5786***	644.2930	1270.5521	635.7797	1269.5681	635.2877	10
13	1459.8308	730.4190	1442.8042	721.9057			Q	***1159.5201***	580.2637	1142.4935	571.7504	1141.5095	571.2584	9
14	1619.8614	810.4343	1602.8349	801.9211			C	***1031.4615***	516.2344	1014.4349	507.7211	1013.4509	507.2291	8
15	1716.9142	858.9607	1699.8876	850.4475			P	***871.4308***	436.2191	854.4043	427.7058	853.4203	427.2138	7
16	1863.9826	932.4949	1846.9560	923.9817			F	***774.3781***	387.6927	757.3515	379.1794	756.3675	378.6874	6
17	1993.0252	997.0162	1975.9986	988.5030	1975.0146	988.0109	E	***627.3097***	314.1585	610.2831	305.6452	609.2991	305.1532	5
18	2108.0521	1054.5297	2091.0256	1046.0164	2090.0416	1045.5244	D	***498.2671***	249.6372	481.2405	241.1239	480.2565	240.6319	4
19	2245.1110	1123.0592	2228.0845	1114.5459	2227.1005	1114.0539	H	***383.2401***	192.1237	366.2136	183.6104			3

**20**	2344.1795	1172.5934	2327.1529	1164.0801	2326.1689	1163.5881	**V**	246.1812	123.5942	229.1547	115.0810			**2**
**21**							**K**	147.1128	74.0600	130.0863	65.5468			**1**

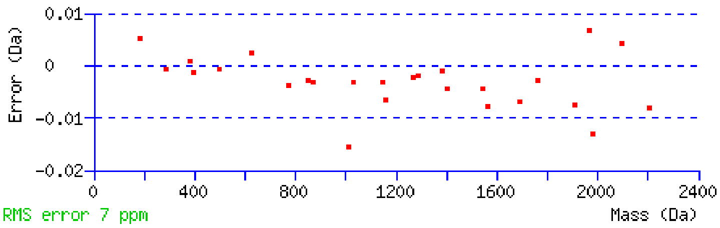

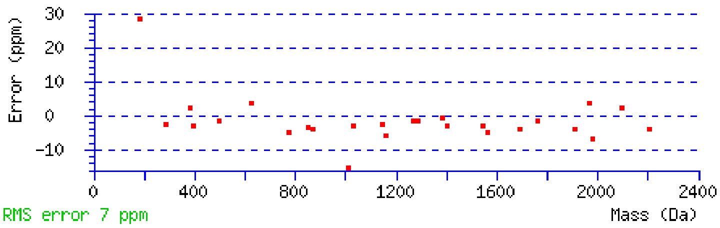

NCBI **BLAST** search of ALVLIAFAQYLQQCPFEDHVK
(Parameters: blastp, nr protein database, expect=20000, no filter, PAM30)
Other BLAST web gateways

All matches to this query

Score	Mr(calc):	Delta	Sequence
132.6	2489.2777	-0.0067	ALVLIAFAQYLQQCPFEDHVK

Mascot: http://www.matrixscience.com/

Mascot Search Results

Peptide View

MS/MS Fragmentation of **ALVLIAFAQYLQQCPFEDHVK**
Found in **sp|P02768|ALBU_HUMAN**, Serum albumin OS=Homo sapiens GN=ALB PE=1 SV=2

Match to Query 12408: 2489.273952 from(830.765260,3+) intensity(9069588.0000) scans(17608) rtinseconds(3102) index(15291)
Title: 150825_TTSH_Patient_Plasma_11_Spectrum033004_scans__17608_RTINSECONDS=3102
Data file L:\\Ard_TTSH\\T1D\\T150825_TTSH_Patient_Plasma_11.mgf

Click mouse within plot area to zoom in by factor of two about that point
Or, [Plot from] [0] to [2400] Da [Full range]
Label all possible matches ○ Label matches used for scoring ⊙

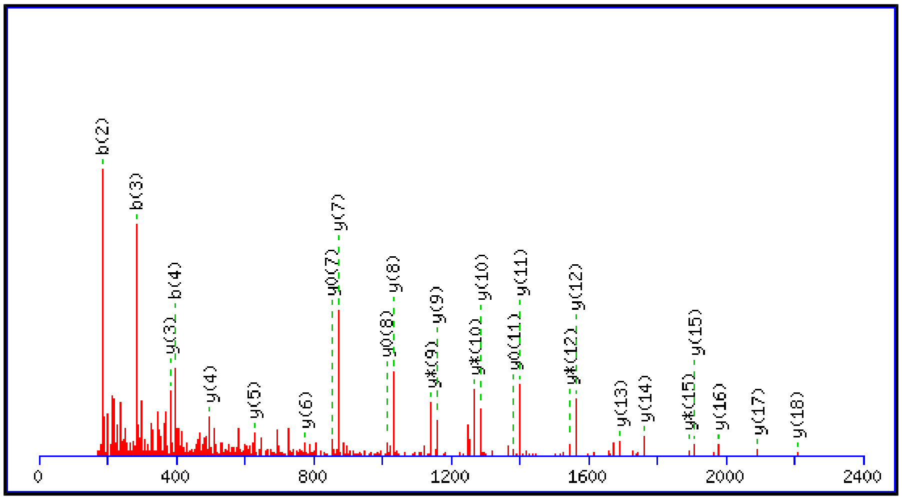

Monoisotopic mass of neutral peptide Mr(calc): 2489.2777
Variable modifications:
C14 : Carbamidomethyl (C)
Ions Score: 133 **Expect:** 1.9e-011
Matches : 26/186 fragment ions using 37 most intense peaks (help)

#	b	b^{++}	b*	b^{*++}	b^0	b^{0++}	Seq.	y	y^{++}	y*	y^{*++}	y^0	y^{0++}	#
1	72.0444	36.5258					A							21
2	**185.1285**	93.0679					L	2419.2479	1210.1276	2402.2213	1201.6143	2401.2373	1201.1223	20
3	**284.1969**	142.6021					V	2306.1638	1153.5855	2289.1373	1145.0723	2288.1532	1144.5803	19
4	**397.2809**	199.1441					L	***2207.0954***	1104.0513	2190.0688	1095.5381	2189.0848	1095.0461	18
5	510.3650	255.6861					I	***2094.0113***	1047.5093	2076.9848	1038.9960	2076.0008	1038.5040	17
6	581.4021	291.2047					A	***1980.9273***	990.9673	1963.9007	982.4540	1962.9167	981.9620	16
7	728.4705	364.7389					F	***1909.8901***	955.4487	1892.8636	946.9354	1891.8796	946.4434	15
8	799.5076	400.2575					A	***1762.8217***	881.9145	1745.7952	873.4012	1744.8112	872.9092	14
9	927.5662	464.2867	910.5397	455.7735			Q	***1691.7846***	846.3959	1674.7581	837.8827	1673.7741	837.3907	13
10	1090.6295	545.8184	1073.6030	537.3051			Y	***1563.7260***	782.3667	1546.6995	773.8534	1545.7155	773.3614	12
11	1203.7136	602.3604	1186.6871	593.8472			L	***1400.6627***	700.8350	1383.6362	692.3217	1382.6521	691.8297	11
12	1331.7722	666.3897	1314.7456	657.8765			Q	***1287.5786***	644.2930	1270.5521	635.7797	1269.5681	635.2877	10
13	1459.8308	730.4190	1442.8042	721.9057			Q	***1159.5201***	580.2637	1142.4935	571.7504	1141.5095	571.2584	9
14	1619.8614	810.4343	1602.8349	801.9211			C	***1031.4615***	516.2344	1014.4349	507.7211	1013.4509	507.2291	8
15	1716.9142	858.9607	1699.8876	850.4475			P	***871.4308***	436.2191	854.4043	427.7058	853.4203	427.2138	7
16	1863.9826	932.4949	1846.9560	923.9817			F	***774.3781***	387.6927	757.3515	379.1794	756.3675	378.6874	6
17	1993.0252	997.0162	1975.9986	988.5030	1975.0146	988.0109	E	***627.3097***	314.1585	610.2831	305.6452	609.2991	305.1532	5
18	2108.0521	1054.5297	2091.0256	1046.0164	2090.0416	1045.5244	D	***498.2671***	249.6372	481.2405	241.1239	480.2565	240.6319	4
19	2245.1110	1123.0592	2228.0845	1114.5459	2227.1005	1114.0539	H	***383.2401***	192.1237	366.2136	183.6104			3

**20**	2344.1795	1172.5934	2327.1529	1164.0801	2326.1689	1163.5881	**V**	246.1812	123.5942	229.1547	115.0810			**2**
**21**							**K**	147.1128	74.0600	130.0863	65.5468			**1**

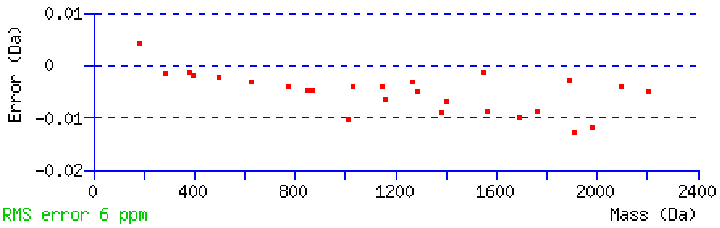

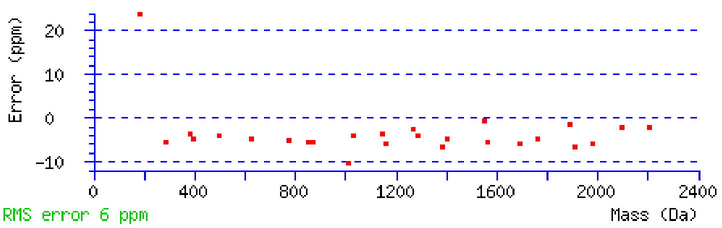

NCBI **BLAST** search of ALVLIAFAQYLQQCPFEDHVK
(Parameters: blastp, nr protein database, expect=20000, no filter, PAM30)
Other BLAST web gateways

All matches to this query

Score	Mr(calc):	Delta	Sequence
132.6	2489.2777	-0.0037	ALVLIAFAQYLQQCPFEDHVK

Mascot: http://www.matrixscience.com/

{MATRIX SCIENCE} Mascot Search Results

Peptide View

MS/MS Fragmentation of **ALVLIAFAQYLQQCPFEDHVK**
Found in **sp|P02768|ALBU_HUMAN**, Serum albumin OS=Homo sapiens GN=ALB PE=1 SV=2

Match to Query 10909: 2489.277432 from(830.766420,3+) intensity(14765883.0000) scans(16135) rtinseconds(3154) index(12910)
Title: 150801_TTSH_Patient_Plasma_76_Spectrum028071_scans__16135_RTINSECONDS=3154
Data file L:\\Ard_TTSH\\T1D\\T150801_TTSH_Patient_Plasma_76.mgf

Click mouse within plot area to zoom in by factor of two about that point
Or, [Plot from] [0] to [2400] Da [Full range]
Label all possible matches ○ Label matches used for scoring ⊙

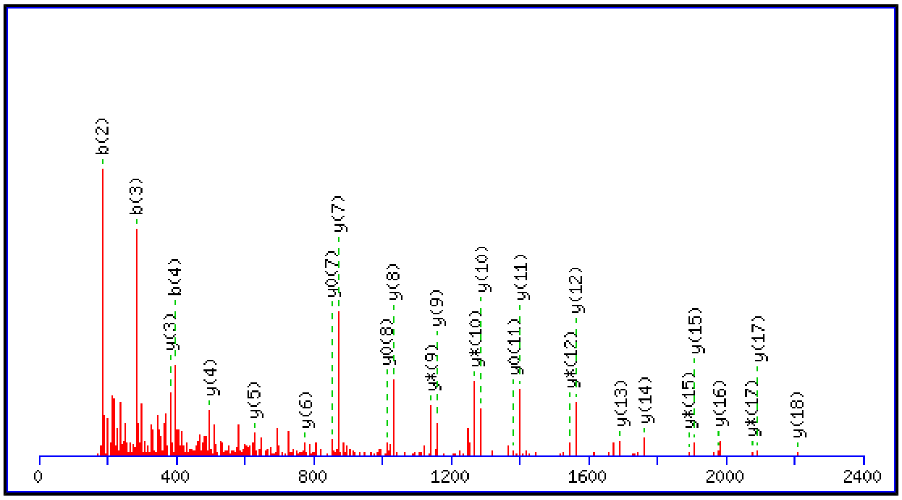

Monoisotopic mass of neutral peptide Mr(calc): 2489.2777
Variable modifications:
C14 : Carbamidomethyl (C)
Ions Score: 130 **Expect:** 3e-011
Matches : 27/186 fragment ions using 39 most intense peaks (help)

#	b	b^{++}	b^{*}	b^{*++}	b^{0}	b^{0++}	Seq.	y	y^{++}	y^{*}	y^{*++}	y^{0}	y^{0++}	#
1	72.0444	36.5258					A							21
2	185.1285	93.0679					L	2419.2479	1210.1276	2402.2213	1201.6143	2401.2373	1201.1223	20
3	284.1969	142.6021					V	2306.1638	1153.5855	2289.1373	1145.0723	2288.1532	1144.5803	19
4	397.2809	199.1441					L	2207.0954	1104.0513	2190.0688	1095.5381	2189.0848	1095.0461	18
5	510.3650	255.6861					I	2094.0113	1047.5093	2076.9848	1038.9960	2076.0008	1038.5040	17
6	581.4021	291.2047					A	1980.9273	990.9673	1963.9007	982.4540	1962.9167	981.9620	16
7	728.4705	364.7389					F	1909.8901	955.4487	1892.8636	946.9354	1891.8796	946.4434	15
8	799.5076	400.2575					A	1762.8217	881.9145	1745.7952	873.4012	1744.8112	872.9092	14
9	927.5662	464.2867	910.5397	455.7735			Q	1691.7846	846.3959	1674.7581	837.8827	1673.7741	837.3907	13
10	1090.6295	545.8184	1073.6030	537.3051			Y	1563.7260	782.3667	1546.6995	773.8534	1545.7155	773.3614	12
11	1203.7136	602.3604	1186.6871	593.8472			L	1400.6627	700.8350	1383.6362	692.3217	1382.6521	691.8297	11
12	1331.7722	666.3897	1314.7456	657.8765			Q	1287.5786	644.2930	1270.5521	635.7797	1269.5681	635.2877	10
13	1459.8308	730.4190	1442.8042	721.9057			Q	1159.5201	580.2637	1142.4935	571.7504	1141.5095	571.2584	9
14	1619.8614	810.4343	1602.8349	801.9211			C	1031.4615	516.2344	1014.4349	507.7211	1013.4509	507.2291	8
15	1716.9142	858.9607	1699.8876	850.4475			P	871.4308	436.2191	854.4043	427.7058	853.4203	427.2138	7
16	1863.9826	932.4949	1846.9560	923.9817			F	774.3781	387.6927	757.3515	379.1794	756.3675	378.6874	6
17	1993.0252	997.0162	1975.9986	988.5030	1975.0146	988.0109	E	627.3097	314.1585	610.2831	305.6452	609.2991	305.1532	5
18	2108.0521	1054.5297	2091.0256	1046.0164	2090.0416	1045.5244	D	498.2671	249.6372	481.2405	241.1239	480.2565	240.6319	4
19	2245.1110	1123.0592	2228.0845	1114.5459	2227.1005	1114.0539	H	383.2401	192.1237	366.2136	183.6104			3

20	2344.1795	1172.5934	2327.1529	1164.0801	2326.1689	1163.5881	V	246.1812	123.5942	229.1547	115.0810			2
21							K	147.1128	74.0600	130.0863	65.5468			1

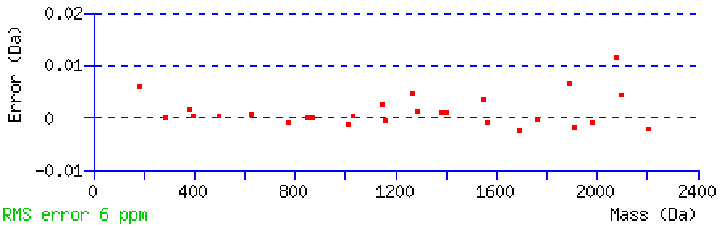

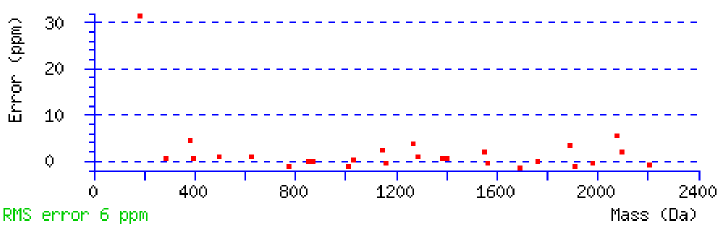

NCBI **BLAST** search of ALVLIAFAQYLQQCPFEDHVK
(Parameters: blastp, nr protein database, expect=20000, no filter, PAM30)
Other BLAST web gateways

All matches to this query

Score	Mr(calc):	Delta	Sequence
130.4	2489.2777	-0.0003	ALVLIAFAQYLQQCPFEDHVK

Mascot: http://www.matrixscience.com/

Mascot Search Results

Peptide View

MS/MS Fragmentation of **ALVLIAFAQYLQQCPFEDHVK**
Found in **sp|P02768|ALBU_HUMAN**, Serum albumin OS=Homo sapiens GN=ALB PE=1 SV=2

Match to Query 13299: 2489.278902 from(830.766910,3+) intensity(9852977.0000) scans(17903) rtinseconds(3134) index(15476)
Title: 150818_TTSH_Patient_Plasma_21_Spectrum033459_scans__17903_RTINSECONDS=3134
Data file L:\\Ard_TTSH\\T1D\\T150818_TTSH_Patient_Plasma_21.mgf

Click mouse within plot area to zoom in by factor of two about that point
Or, Plot from 0 to 2400 Da Full range
Label all possible matches ○ Label matches used for scoring ⊙

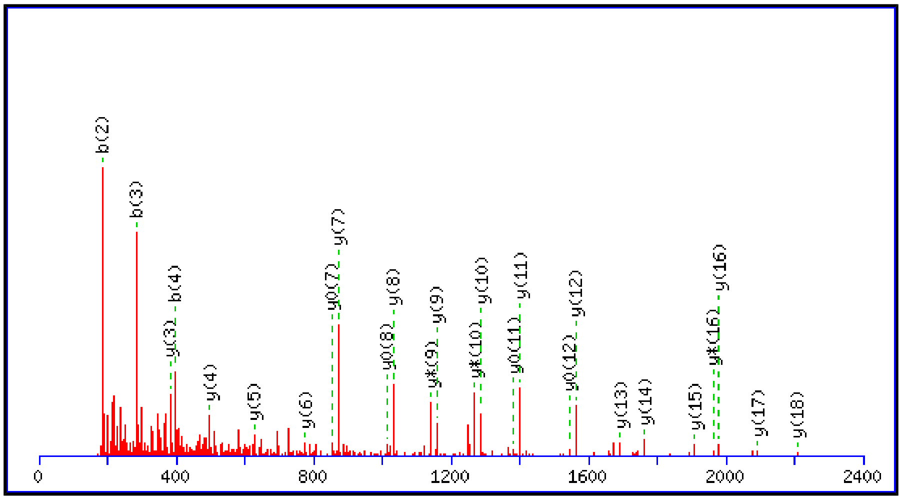

Monoisotopic mass of neutral peptide Mr(calc): 2489.2777
Variable modifications:
C14 : Carbamidomethyl (C)
Ions Score: 128 **Expect:** 6.6e-011
Matches : 26/186 fragment ions using 39 most intense peaks (help)

#	b	b++	b*	b*++	b0	b0++	Seq.	y	y++	y*	y*++	y0	y0++	#
1	72.0444	36.5258					A							21
2	**185.1285**	93.0679					L	2419.2479	1210.1276	2402.2213	1201.6143	2401.2373	1201.1223	20
3	**284.1969**	142.6021					V	2306.1638	1153.5855	2289.1373	1145.0723	2288.1532	1144.5803	19
4	**397.2809**	199.1441					L	***2207.0954***	1104.0513	2190.0688	1095.5381	2189.0848	1095.0461	18
5	510.3650	255.6861					I	***2094.0113***	1047.5093	2076.9848	1038.9960	2076.0008	1038.5040	17
6	581.4021	291.2047					A	***1980.9273***	990.9673	1963.9007	982.4540	1962.9167	981.9620	16
7	728.4705	364.7389					F	***1909.8901***	955.4487	1892.8636	946.9354	1891.8796	946.4434	15
8	799.5076	400.2575					A	***1762.8217***	881.9145	1745.7952	873.4012	1744.8112	872.9092	14
9	927.5662	464.2867	910.5397	455.7735			Q	***1691.7846***	846.3959	1674.7581	837.8827	1673.7741	837.3907	13
10	1090.6295	545.8184	1073.6030	537.3051			Y	***1563.7260***	782.3667	1546.6995	773.8534	1545.7155	773.3614	12
11	1203.7136	602.3604	1186.6871	593.8472			L	***1400.6627***	700.8350	1383.6362	692.3217	1382.6521	691.8297	11
12	1331.7722	666.3897	1314.7456	657.8765			Q	***1287.5786***	644.2930	1270.5521	635.7797	1269.5681	635.2877	10
13	1459.8308	730.4190	1442.8042	721.9057			Q	***1159.5201***	580.2637	1142.4935	571.7504	1141.5095	571.2584	9
14	1619.8614	810.4343	1602.8349	801.9211			C	***1031.4615***	516.2344	1014.4349	507.7211	1013.4509	507.2291	8
15	1716.9142	858.9607	1699.8876	850.4475			P	***871.4308***	436.2191	854.4043	427.7058	853.4203	427.2138	7
16	1863.9826	932.4949	1846.9560	923.9817			F	***774.3781***	387.6927	757.3515	379.1794	756.3675	378.6874	6
17	1993.0252	997.0162	1975.9986	988.5030	1975.0146	988.0109	E	***627.3097***	314.1585	610.2831	305.6452	609.2991	305.1532	5
18	2108.0521	1054.5297	2091.0256	1046.0164	2090.0416	1045.5244	D	***498.2671***	249.6372	481.2405	241.1239	480.2565	240.6319	4
19	2245.1110	1123.0592	2228.0845	1114.5459	2227.1005	1114.0539	H	***383.2401***	192.1237	366.2136	183.6104			3

**20**	2344.1795	1172.5934	2327.1529	1164.0801	2326.1689	1163.5881	**V**	246.1812	123.5942	229.1547	115.0810			**2**
**21**							**K**	147.1128	74.0600	130.0863	65.5468			**1**

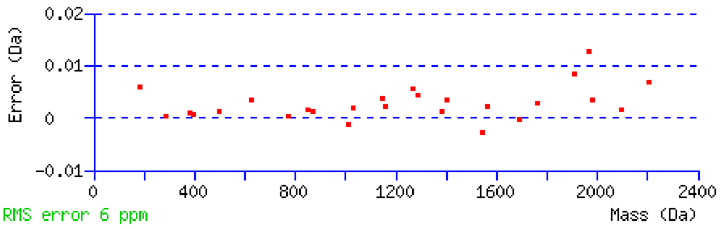

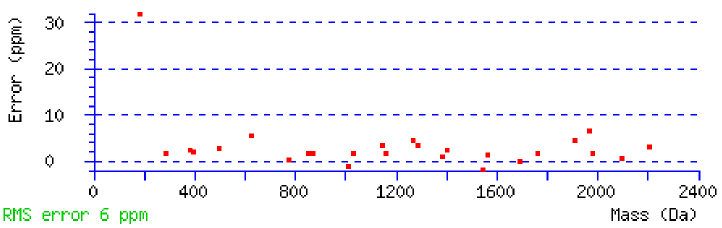

NCBI **BLAST** search of ALVLIAFAQYLQQCPFEDHVK
(Parameters: blastp, nr protein database, expect=20000, no filter, PAM30)
Other BLAST web gateways

All matches to this query

Score	Mr(calc):	Delta	Sequence
127.5	2489.2777	0.0012	ALVLIAFAQYLQQCPFEDHVK

Mascot: http://www.matrixscience.com/

MATRIX SCIENCE Mascot Search Results

Peptide View

MS/MS Fragmentation of **ALVLIAFAQYLQQCPFEDHVK**
Found in **sp|P02768|ALBU_HUMAN**, Serum albumin OS=Homo sapiens GN=ALB PE=1 SV=2

Match to Query 13094: 2489.278332 from(830.766720,3+) intensity(15139741.0000) scans(18246) rtinseconds(3182) index(16014)
Title: 150825_TTSH_Patient_Plasma_15_Spectrum034026_scans__18246_RTINSECONDS=3182
Data file L:\\Ard_TTSH\\T1D\\T150825_TTSH_Patient_Plasma_15.mgf

Click mouse within plot area to zoom in by factor of two about that point
Or, [Plot from] 0 to 2600 Da [Full range]
Label all possible matches ○ Label matches used for scoring ⦿

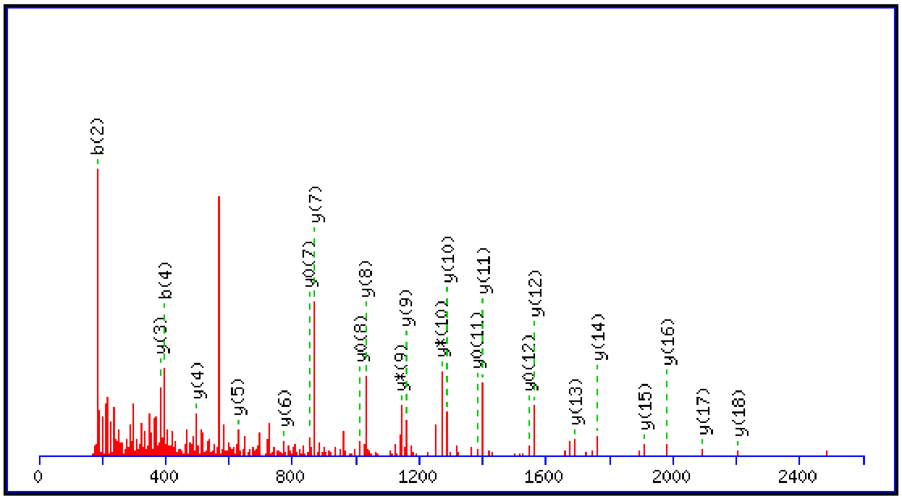

Monoisotopic mass of neutral peptide Mr(calc): 2489.2777
Variable modifications:
C14 : Carbamidomethyl (C)
Ions Score: 123 **Expect:** 1.8e-010
Matches : 24/186 fragment ions using 38 most intense peaks (help)

#	b	b^{++}	b*	b^{*++}	b^0	b^{0++}	Seq.	y	y^{++}	y*	y^{*++}	y^0	y^{0++}	#
1	72.0444	36.5258					A							21
2	**185.1285**	93.0679					L	2419.2479	1210.1276	2402.2213	1201.6143	2401.2373	1201.1223	20
3	284.1969	142.6021					V	2306.1638	1153.5855	2289.1373	1145.0723	2288.1532	1144.5803	19
4	**397.2809**	199.1441					L	***2207.0954***	1104.0513	2190.0688	1095.5381	2189.0848	1095.0461	18
5	510.3650	255.6861					I	***2094.0113***	1047.5093	2076.9848	1038.9960	2076.0008	1038.5040	17
6	581.4021	291.2047					A	***1980.9273***	990.9673	1963.9007	982.4540	1962.9167	981.9620	16
7	728.4705	364.7389					F	***1909.8901***	955.4487	1892.8636	946.9354	1891.8796	946.4434	15
8	799.5076	400.2575					A	***1762.8217***	881.9145	1745.7952	873.4012	1744.8112	872.9092	14
9	927.5662	464.2867	910.5397	455.7735			Q	***1691.7846***	846.3959	1674.7581	837.8827	1673.7741	837.3907	13
10	1090.6295	545.8184	1073.6030	537.3051			Y	***1563.7260***	782.3667	1546.6995	773.8534	1545.7155	773.3614	12
11	1203.7136	602.3604	1186.6871	593.8472			L	***1400.6627***	700.8350	1383.6362	692.3217	1382.6521	691.8297	11
12	1331.7722	666.3897	1314.7456	657.8765			Q	***1287.5786***	644.2930	1270.5521	635.7797	1269.5681	635.2877	10
13	1459.8308	730.4190	1442.8042	721.9057			Q	***1159.5201***	580.2637	1142.4935	571.7504	1141.5095	571.2584	9
14	1619.8614	810.4343	1602.8349	801.9211			C	***1031.4615***	516.2344	1014.4349	507.7211	1013.4509	507.2291	8
15	1716.9142	858.9607	1699.8876	850.4475			P	***871.4308***	436.2191	854.4043	427.7058	853.4203	427.2138	7
16	1863.9826	932.4949	1846.9560	923.9817			F	***774.3781***	387.6927	757.3515	379.1794	756.3675	378.6874	6
17	1993.0252	997.0162	1975.9986	988.5030	1975.0146	988.0109	E	***627.3097***	314.1585	610.2831	305.6452	609.2991	305.1532	5
18	2108.0521	1054.5297	2091.0256	1046.0164	2090.0416	1045.5244	D	***498.2671***	249.6372	481.2405	241.1239	480.2565	240.6319	4
19	2245.1110	1123.0592	2228.0845	1114.5459	2227.1005	1114.0539	H	***383.2401***	192.1237	366.2136	183.6104			3

**20**	2344.1795	1172.5934	2327.1529	1164.0801	2326.1689	1163.5881	**V**	246.1812	123.5942	229.1547	115.0810			**2**
**21**							**K**	147.1128	74.0600	130.0863	65.5468			**1**

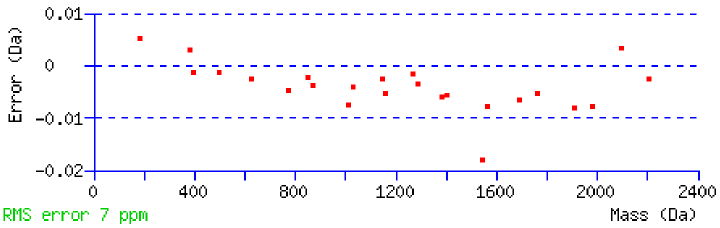

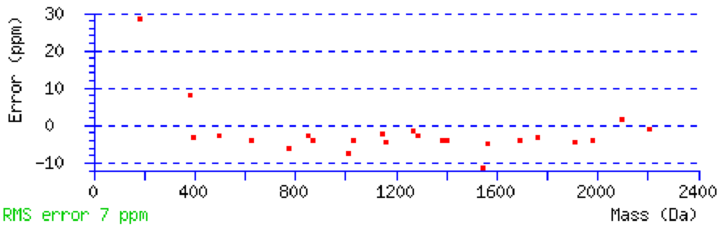

NCBI **BLAST** search of ALVLIAFAQYLQQCPFEDHVK
(Parameters: blastp, nr protein database, expect=20000, no filter, PAM30)
Other BLAST web gateways

All matches to this query

Score	Mr(calc):	Delta	Sequence
123.3	2489.2777	0.0006	ALVLIAFAQYLQQCPFEDHVK

Mascot: http://www.matrixscience.com/

Mascot Search Results

Peptide View

MS/MS Fragmentation of **ALVLIAFAQYLQQCPFEDHVK**
Found in **sp|P02768|ALBU_HUMAN**, Serum albumin OS=Homo sapiens GN=ALB PE=1 SV=2

Match to Query 11212: 2489.275962 from(830.765930,3+) intensity(2619101.5000) scans(735) rtinseconds(132) index(611)
Title: 150818_TTSH_Patient_Plasma_24_Spectrum019129_scans__735_RTINSECONDS=132
Data file L:\\Ard_TTSH\\T1D\\T150818_TTSH_Patient_Plasma_24.mgf

Click mouse within plot area to zoom in by factor of two about that point
Or, Plot from 0 to 2400 Da Full range
Label all possible matches ○ Label matches used for scoring ⦿

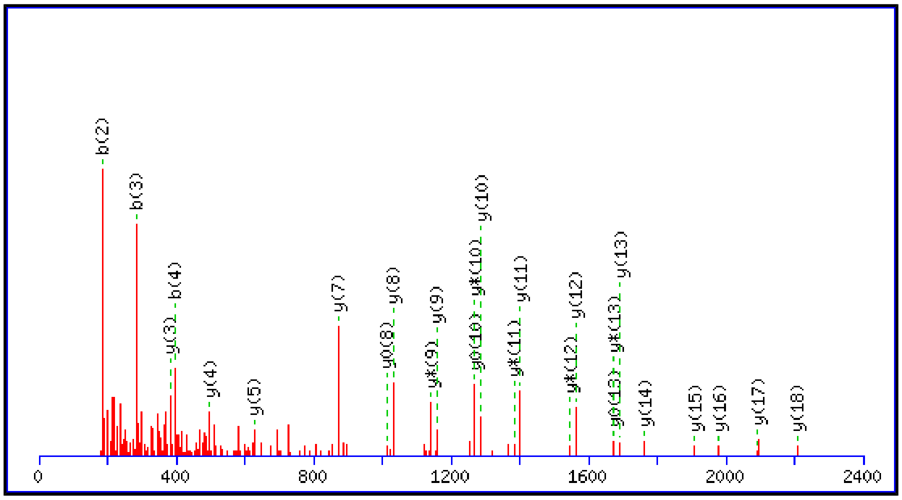

Monoisotopic mass of neutral peptide Mr(calc): 2489.2777
Variable modifications:
C14 : Carbamidomethyl (C)
Ions Score: 121 **Expect:** 3e-010
Matches : 26/186 fragment ions using 37 most intense peaks (help)

#	b	b^{++}	b*	b^{*++}	b^{0}	b^{0++}	Seq.	y	y^{++}	y*	y^{*++}	y^{0}	y^{0++}	#
1	72.0444	36.5258					A							21
2	**185.1285**	93.0679					L	2419.2479	1210.1276	2402.2213	1201.6143	2401.2373	1201.1223	20
3	**284.1969**	142.6021					V	2306.1638	1153.5855	2289.1373	1145.0723	2288.1532	1144.5803	19
4	**397.2809**	199.1441					L	***2207.0954***	1104.0513	2190.0688	1095.5381	2189.0848	1095.0461	18
5	510.3650	255.6861					I	***2094.0113***	1047.5093	2076.9848	1038.9960	2076.0008	1038.5040	17
6	581.4021	291.2047					A	***1980.9273***	990.9673	1963.9007	982.4540	1962.9167	981.9620	16
7	728.4705	364.7389					F	***1909.8901***	955.4487	1892.8636	946.9354	1891.8796	946.4434	15
8	799.5076	400.2575					A	***1762.8217***	881.9145	1745.7952	873.4012	1744.8112	872.9092	14
9	927.5662	464.2867	910.5397	455.7735			Q	***1691.7846***	846.3959	1674.7581	837.8827	1673.7741	837.3907	13
10	1090.6295	545.8184	1073.6030	537.3051			Y	***1563.7260***	782.3667	1546.6995	773.8534	1545.7155	773.3614	12
11	1203.7136	602.3604	1186.6871	593.8472			L	***1400.6627***	700.8350	1383.6362	692.3217	1382.6521	691.8297	11
12	1331.7722	666.3897	1314.7456	657.8765			Q	***1287.5786***	644.2930	1270.5521	635.7797	1269.5681	635.2877	10
13	1459.8308	730.4190	1442.8042	721.9057			Q	***1159.5201***	580.2637	1142.4935	571.7504	1141.5095	571.2584	9
14	1619.8614	810.4343	1602.8349	801.9211			C	***1031.4615***	516.2344	1014.4349	507.7211	1013.4509	507.2291	8
15	1716.9142	858.9607	1699.8876	850.4475			P	***871.4308***	436.2191	854.4043	427.7058	853.4203	427.2138	7
16	1863.9826	932.4949	1846.9560	923.9817			F	774.3781	387.6927	757.3515	379.1794	756.3675	378.6874	6
17	1993.0252	997.0162	1975.9986	988.5030	1975.0146	988.0109	E	***627.3097***	314.1585	610.2831	305.6452	609.2991	305.1532	5
18	2108.0521	1054.5297	2091.0256	1046.0164	2090.0416	1045.5244	D	***498.2671***	249.6372	481.2405	241.1239	480.2565	240.6319	4
19	2245.1110	1123.0592	2228.0845	1114.5459	2227.1005	1114.0539	H	***383.2401***	192.1237	366.2136	183.6104			3

**20**	2344.1795	1172.5934	2327.1529	1164.0801	2326.1689	1163.5881	**V**	246.1812	123.5942	229.1547	115.0810			**2**
**21**							**K**	147.1128	74.0600	130.0863	65.5468			**1**

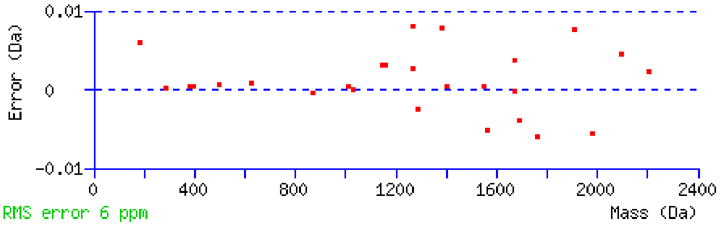

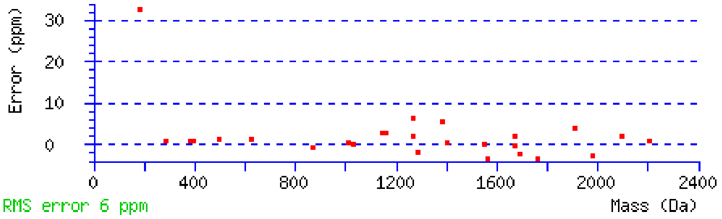

NCBI **BLAST** search of ALVLIAFAQYLQQCPFEDHVK
(Parameters: blastp, nr protein database, expect=20000, no filter, PAM30)
Other BLAST web gateways

All matches to this query

Score	Mr(calc):	Delta	Sequence
120.5	2489.2777	-0.0017	ALVLIAFAQYLQQCPFEDHVK

Mascot: http://www.matrixscience.com/

MATRIX SCIENCE Mascot Search Results

Peptide View

MS/MS Fragmentation of **ALVLIAFAQYLQQCPFEDHVK**
Found in **sp|P02768|ALBU_HUMAN**, Serum albumin OS=Homo sapiens GN=ALB PE=1 SV=2

Match to Query 11678: 2489.277252 from(830.766360,3+) intensity(79334032.0000) scans(17667) rtinseconds(3159) index(15027)
Title: 150825_TTSH_Patient_Plasma_12_Spectrum032309_scans__17667_RTINSECONDS=3159
Data file L:\\Ard_TTSH\\T1D\\T150825_TTSH_Patient_Plasma_12.mgf

Click mouse within plot area to zoom in by factor of two about that point
Or, [Plot from] 0 to 2400 Da [Full range]
Label all possible matches ○ Label matches used for scoring ⊙

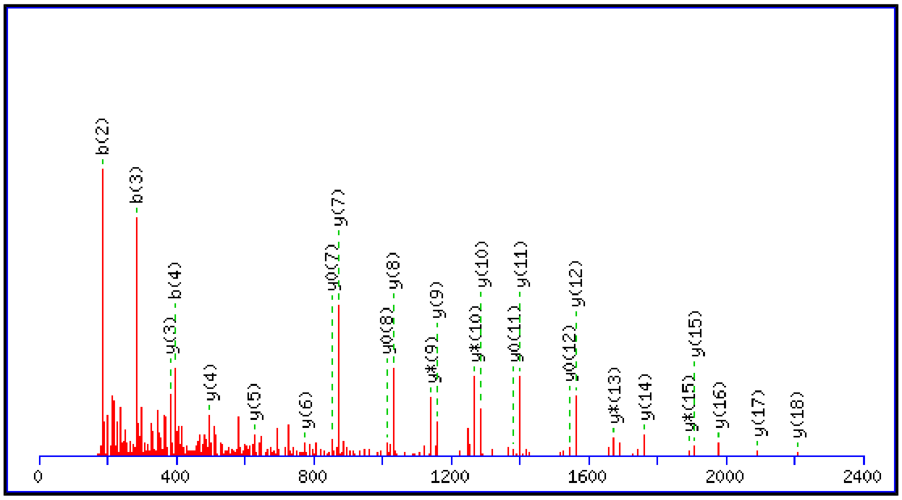

Monoisotopic mass of neutral peptide Mr(calc): 2489.2777
Variable modifications:
C14 : Carbamidomethyl (C)
Ions Score: 120 **Expect:** 3.1e-010
Matches : 26/186 fragment ions using 37 most intense peaks (help)

#	b	b++	b*	b*++	b0	b0++	Seq.	y	y++	y*	y*++	y0	y0++	#
1	72.0444	36.5258					A							21
2	**185.1285**	93.0679					L	2419.2479	1210.1276	2402.2213	1201.6143	2401.2373	1201.1223	20
3	**284.1969**	142.6021					V	2306.1638	1153.5855	2289.1373	1145.0723	2288.1532	1144.5803	19
4	**397.2809**	199.1441					L	***2207.0954***	1104.0513	2190.0688	1095.5381	2189.0848	1095.0461	18
5	510.3650	255.6861					I	***2094.0113***	1047.5093	2076.9848	1038.9960	2076.0008	1038.5040	17
6	581.4021	291.2047					A	***1980.9273***	990.9673	1963.9007	982.4540	1962.9167	981.9620	16
7	728.4705	364.7389					F	***1909.8901***	955.4487	1892.8636	946.9354	1891.8796	946.4434	15
8	799.5076	400.2575					A	***1762.8217***	881.9145	1745.7952	873.4012	1744.8112	872.9092	14
9	927.5662	464.2867	910.5397	455.7735			Q	1691.7846	846.3959	1674.7581	837.8827	1673.7741	837.3907	13
10	1090.6295	545.8184	1073.6030	537.3051			Y	***1563.7260***	782.3667	1546.6995	773.8534	1545.7155	773.3614	12
11	1203.7136	602.3604	1186.6871	593.8472			L	***1400.6627***	700.8350	1383.6362	692.3217	1382.6521	691.8297	11
12	1331.7722	666.3897	1314.7456	657.8765			Q	***1287.5786***	644.2930	1270.5521	635.7797	1269.5681	635.2877	10
13	1459.8308	730.4190	1442.8042	721.9057			Q	***1159.5201***	580.2637	1142.4935	571.7504	1141.5095	571.2584	9
14	1619.8614	810.4343	1602.8349	801.9211			C	***1031.4615***	516.2344	1014.4349	507.7211	1013.4509	507.2291	8
15	1716.9142	858.9607	1699.8876	850.4475			P	***871.4308***	436.2191	854.4043	427.7058	853.4203	427.2138	7
16	1863.9826	932.4949	1846.9560	923.9817			F	***774.3781***	387.6927	757.3515	379.1794	756.3675	378.6874	6
17	1993.0252	997.0162	1975.9986	988.5030	1975.0146	988.0109	E	***627.3097***	314.1585	610.2831	305.6452	609.2991	305.1532	5
18	2108.0521	1054.5297	2091.0256	1046.0164	2090.0416	1045.5244	D	***498.2671***	249.6372	481.2405	241.1239	480.2565	240.6319	4
19	2245.1110	1123.0592	2228.0845	1114.5459	2227.1005	1114.0539	H	***383.2401***	192.1237	366.2136	183.6104			3

**20**	2344.1795	1172.5934	2327.1529	1164.0801	2326.1689	1163.5881	**V**	246.1812	123.5942	229.1547	115.0810			**2**
**21**							**K**	147.1128	74.0600	130.0863	65.5468			**1**

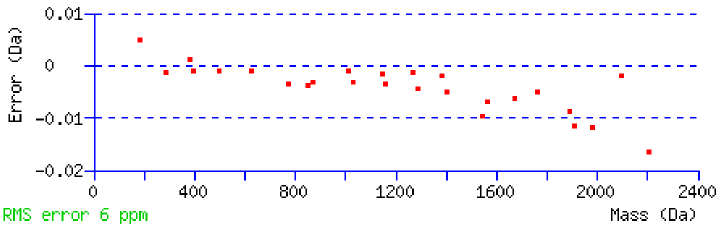

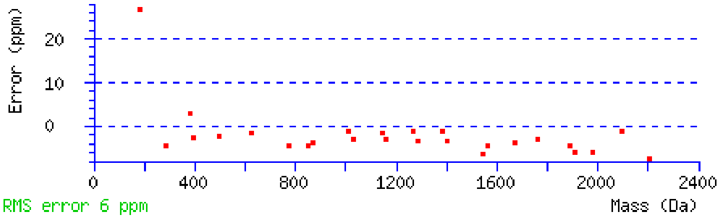

NCBI **BLAST** search of ALVLIAFAQYLQQCPFEDHVK
(Parameters: blastp, nr protein database, expect=20000, no filter, PAM30)
Other BLAST web gateways

All matches to this query

Score	Mr(calc):	Delta	Sequence
120.3	2489.2777	-0.0004	ALVLIAFAQYLQQCPFEDHVK

Mascot: http://www.matrixscience.com/

{MATRIX SCIENCE} Mascot Search Results

Peptide View

MS/MS Fragmentation of **ALVLIAFAQYLQQCPFEDHVK**
Found in **sp|P02768|ALBU_HUMAN**, Serum albumin OS=Homo sapiens GN=ALB PE=1 SV=2

Match to Query 11692: 2489.273592 from(830.765140,3+) intensity(4482005.0000) scans(16956) rtinseconds(2988) index(14512)
Title: 150801_TTSH_Patient_Plasma_45_Spectrum031938_scans__16956_RTINSECONDS=2988
Data file L:\\Ard_TTSH\\T1D\\T150801_TTSH_Patient_Plasma_45.mgf

Click mouse within plot area to zoom in by factor of two about that point
Or, [Plot from] [0] to [2400] Da [Full range]
Label all possible matches ○ Label matches used for scoring ⊙

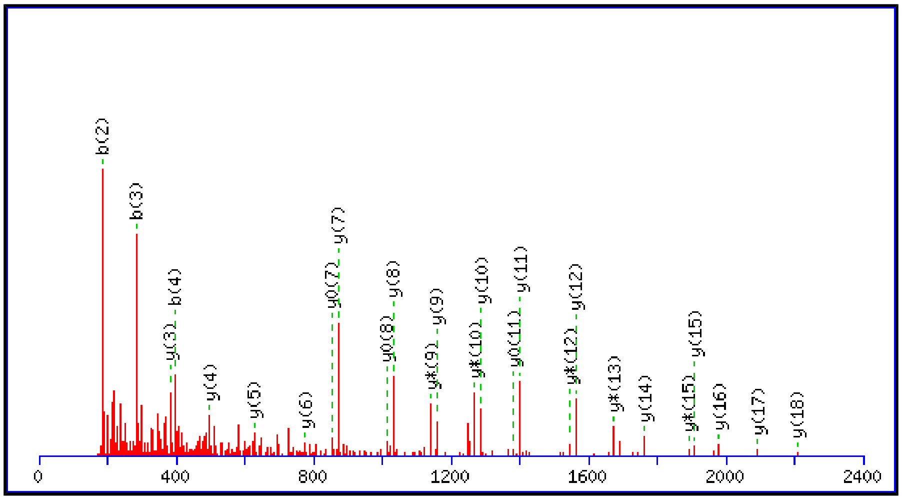

Monoisotopic mass of neutral peptide Mr(calc): 2489.2777
Variable modifications:
C14 : Carbamidomethyl (C)
Ions Score: 120 **Expect:** 3.3e-010
Matches : 26/186 fragment ions using 37 most intense peaks (help)

#	b	b++	b*	b*++	b0	b0++	Seq.	y	y++	y*	y*++	y0	y0++	#
1	72.0444	36.5258					A							21
2	**185.1285**	93.0679					L	2419.2479	1210.1276	2402.2213	1201.6143	2401.2373	1201.1223	20
3	**284.1969**	142.6021					V	2306.1638	1153.5855	2289.1373	1145.0723	2288.1532	1144.5803	19
4	**397.2809**	199.1441					L	***2207.0954***	1104.0513	2190.0688	1095.5381	2189.0848	1095.0461	18
5	510.3650	255.6861					I	***2094.0113***	1047.5093	2076.9848	1038.9960	2076.0008	1038.5040	17
6	581.4021	291.2047					A	***1980.9273***	990.9673	1963.9007	982.4540	1962.9167	981.9620	16
7	728.4705	364.7389					F	***1909.8901***	955.4487	1892.8636	946.9354	1891.8796	946.4434	15
8	799.5076	400.2575					A	***1762.8217***	881.9145	1745.7952	873.4012	1744.8112	872.9092	14
9	927.5662	464.2867	910.5397	455.7735			Q	1691.7846	846.3959	1674.7581	837.8827	1673.7741	837.3907	13
10	1090.6295	545.8184	1073.6030	537.3051			Y	***1563.7260***	782.3667	1546.6995	773.8534	1545.7155	773.3614	12
11	1203.7136	602.3604	1186.6871	593.8472			L	***1400.6627***	700.8350	1383.6362	692.3217	1382.6521	691.8297	11
12	1331.7722	666.3897	1314.7456	657.8765			Q	***1287.5786***	644.2930	1270.5521	635.7797	1269.5681	635.2877	10
13	1459.8308	730.4190	1442.8042	721.9057			Q	***1159.5201***	580.2637	1142.4935	571.7504	1141.5095	571.2584	9
14	1619.8614	810.4343	1602.8349	801.9211			C	***1031.4615***	516.2344	1014.4349	507.7211	1013.4509	507.2291	8
15	1716.9142	858.9607	1699.8876	850.4475			P	***871.4308***	436.2191	854.4043	427.7058	853.4203	427.2138	7
16	1863.9826	932.4949	1846.9560	923.9817			F	***774.3781***	387.6927	757.3515	379.1794	756.3675	378.6874	6
17	1993.0252	997.0162	1975.9986	988.5030	1975.0146	988.0109	E	***627.3097***	314.1585	610.2831	305.6452	609.2991	305.1532	5
18	2108.0521	1054.5297	2091.0256	1046.0164	2090.0416	1045.5244	D	***498.2671***	249.6372	481.2405	241.1239	480.2565	240.6319	4
19	2245.1110	1123.0592	2228.0845	1114.5459	2227.1005	1114.0539	H	***383.2401***	192.1237	366.2136	183.6104			3

**20**	2344.1795	1172.5934	2327.1529	1164.0801	2326.1689	1163.5881	**V**	246.1812	123.5942	229.1547	115.0810			**2**
**21**							**K**	147.1128	74.0600	130.0863	65.5468			**1**

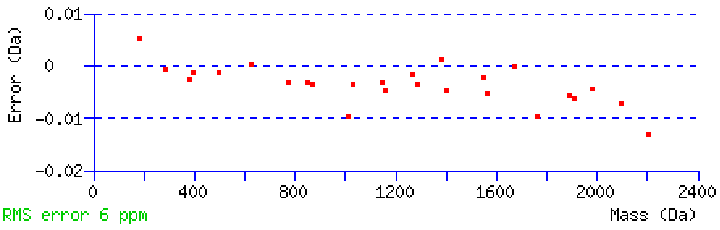

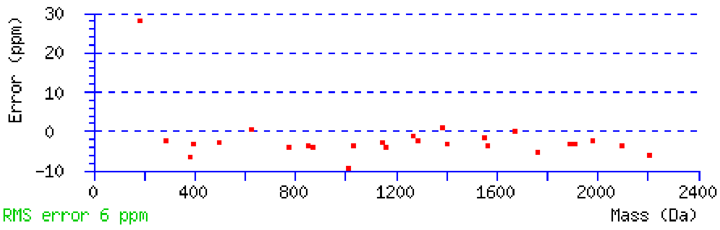

NCBI **BLAST** search of [ALVLIAFAQYLQQCPFEDHVK]
(Parameters: blastp, nr protein database, expect=20000, no filter, PAM30)
Other BLAST [web gateways]

All matches to this query

Score	Mr(calc):	Delta	Sequence
120.2	2489.2777	-0.0041	ALVLIAFAQYLQQCPFEDHVK

Mascot: http://www.matrixscience.com/

Mascot Search Results

Peptide View

MS/MS Fragmentation of **ALVLIAFAQYLQQCPFEDHVK**
Found in **sp|P02768|ALBU_HUMAN**, Serum albumin OS=Homo sapiens GN=ALB PE=1 SV=2

Match to Query 12498: 2489.275782 from(830.765870,3+) intensity(14878000.0000) scans(17639) rtinseconds(3039) index(15292)
Title: 150818_TTSH_Patient_Plasma_40_Spectrum033900_scans__17639_RTINSECONDS=3039
Data file L:\\Ard_TTSH\\T1D\\T150818_TTSH_Patient_Plasma_40.mgf

Click mouse within plot area to zoom in by factor of two about that point
Or, Plot from 0 to 2400 Da Full range
Label all possible matches ○ Label matches used for scoring ⦿

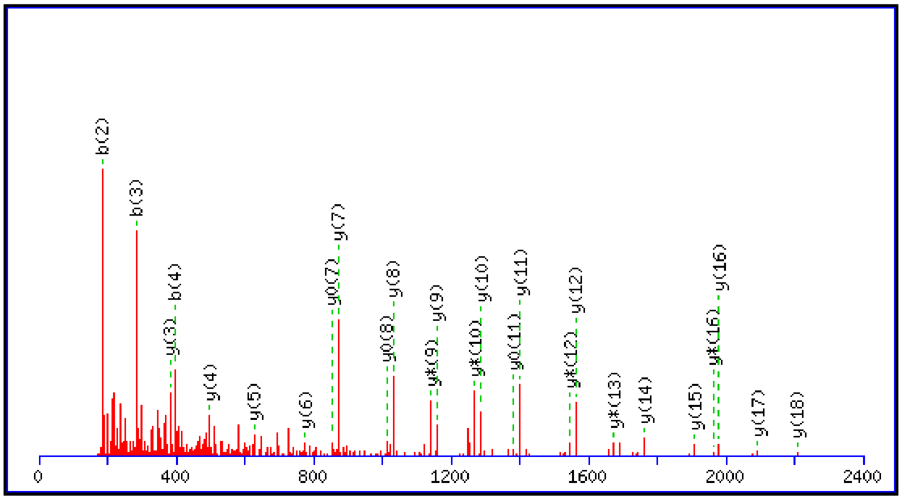

Monoisotopic mass of neutral peptide Mr(calc): 2489.2777
Variable modifications:
C14 : Carbamidomethyl (C)
Ions Score: 120 **Expect:** 3.2e-010
Matches : 26/186 fragment ions using 37 most intense peaks (help)

#	b	b++	b*	b*++	b0	b0++	Seq.	y	y++	y*	y*++	y0	y0++	#
1	72.0444	36.5258					A							21
2	**185.1285**	93.0679					L	2419.2479	1210.1276	2402.2213	1201.6143	2401.2373	1201.1223	20
3	**284.1969**	142.6021					V	2306.1638	1153.5855	2289.1373	1145.0723	2288.1532	1144.5803	19
4	**397.2809**	199.1441					L	***2207.0954***	1104.0513	2190.0688	1095.5381	2189.0848	1095.0461	18
5	510.3650	255.6861					I	***2094.0113***	1047.5093	2076.9848	1038.9960	2076.0008	1038.5040	17
6	581.4021	291.2047					A	***1980.9273***	990.9673	1963.9007	982.4540	1962.9167	981.9620	16
7	728.4705	364.7389					F	***1909.8901***	955.4487	1892.8636	946.9354	1891.8796	946.4434	15
8	799.5076	400.2575					A	***1762.8217***	881.9145	1745.7952	873.4012	1744.8112	872.9092	14
9	927.5662	464.2867	910.5397	455.7735			Q	1691.7846	846.3959	1674.7581	837.8827	1673.7741	837.3907	13
10	1090.6295	545.8184	1073.6030	537.3051			Y	***1563.7260***	782.3667	1546.6995	773.8534	1545.7155	773.3614	12
11	1203.7136	602.3604	1186.6871	593.8472			L	***1400.6627***	700.8350	1383.6362	692.3217	1382.6521	691.8297	11
12	1331.7722	666.3897	1314.7456	657.8765			Q	***1287.5786***	644.2930	1270.5521	635.7797	1269.5681	635.2877	10
13	1459.8308	730.4190	1442.8042	721.9057			Q	***1159.5201***	580.2637	1142.4935	571.7504	1141.5095	571.2584	9
14	1619.8614	810.4343	1602.8349	801.9211			C	***1031.4615***	516.2344	1014.4349	507.7211	1013.4509	507.2291	8
15	1716.9142	858.9607	1699.8876	850.4475			P	***871.4308***	436.2191	854.4043	427.7058	853.4203	427.2138	7
16	1863.9826	932.4949	1846.9560	923.9817			F	***774.3781***	387.6927	757.3515	379.1794	756.3675	378.6874	6
17	1993.0252	997.0162	1975.9986	988.5030	1975.0146	988.0109	E	***627.3097***	314.1585	610.2831	305.6452	609.2991	305.1532	5
18	2108.0521	1054.5297	2091.0256	1046.0164	2090.0416	1045.5244	D	***498.2671***	249.6372	481.2405	241.1239	480.2565	240.6319	4
19	2245.1110	1123.0592	2228.0845	1114.5459	2227.1005	1114.0539	H	***383.2401***	192.1237	366.2136	183.6104			3

**20**	2344.1795	1172.5934	2327.1529	1164.0801	2326.1689	1163.5881	**V**	246.1812	123.5942	229.1547	115.0810			**2**
**21**							**K**	147.1128	74.0600	130.0863	65.5468			**1**

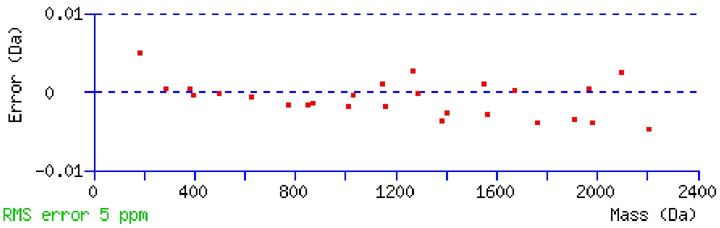

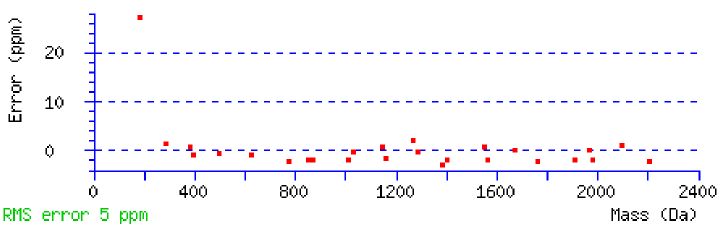

NCBI **BLAST** search of ALVLIAFAQYLQQCPFEDHVK
(Parameters: blastp, nr protein database, expect=20000, no filter, PAM30)
Other BLAST web gateways

All matches to this query

Score	Mr(calc):	Delta	Sequence
120.2	2489.2777	-0.0019	ALVLIAFAQYLQQCPFEDHVK

Mascot: http://www.matrixscience.com/

Mascot Search Results

Peptide View

MS/MS Fragmentation of **ALVLIAFAQYLQQCPFEDHVK**
Found in **sp|P02768|ALBU_HUMAN**, Serum albumin OS=Homo sapiens GN=ALB PE=1 SV=2

Match to Query 12607: 2489.282202 from(830.768010,3+) intensity(24298394.0000) scans(17684) rtinseconds(3071) index(15140)
Title: 150818_TTSH_Patient_Plasma_51_Spectrum033411_scans__17684_RTINSECONDS=3071
Data file L:\\Ard_TTSH\\T1D\\T150818_TTSH_Patient_Plasma_51.mgf

Click mouse within plot area to zoom in by factor of two about that point
Or, Plot from 0 to 2400 Da Full range
Label all possible matches ○ Label matches used for scoring ⊙

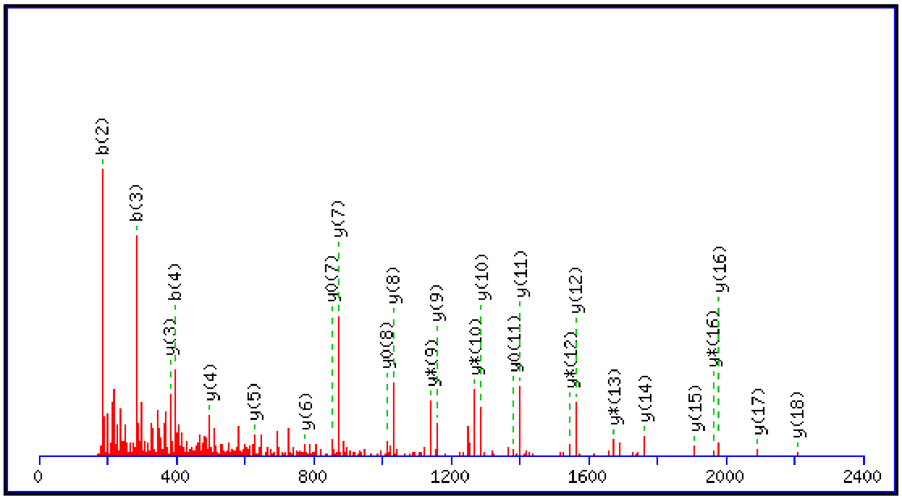

Monoisotopic mass of neutral peptide Mr(calc): 2489.2777
Variable modifications:
C14 : Carbamidomethyl (C)
Ions Score: 120 **Expect:** 3.5e-010
Matches : 26/186 fragment ions using 37 most intense peaks (help)

#	b	b^{++}	b*	b^{*++}	b^0	b^{0++}	Seq.	y	y^{++}	y*	y^{*++}	y^0	y^{0++}	#
1	72.0444	36.5258					A							21
2	**185.1285**	93.0679					L	2419.2479	1210.1276	2402.2213	1201.6143	2401.2373	1201.1223	20
3	**284.1969**	142.6021					V	2306.1638	1153.5855	2289.1373	1145.0723	2288.1532	1144.5803	19
4	**397.2809**	199.1441					L	***2207.0954***	1104.0513	2190.0688	1095.5381	2189.0848	1095.0461	18
5	510.3650	255.6861					I	***2094.0113***	1047.5093	2076.9848	1038.9960	2076.0008	1038.5040	17
6	581.4021	291.2047					A	***1980.9273***	990.9673	1963.9007	982.4540	1962.9167	981.9620	16
7	728.4705	364.7389					F	***1909.8901***	955.4487	1892.8636	946.9354	1891.8796	946.4434	15
8	799.5076	400.2575					A	***1762.8217***	881.9145	1745.7952	873.4012	1744.8112	872.9092	14
9	927.5662	464.2867	910.5397	455.7735			Q	1691.7846	846.3959	1674.7581	837.8827	1673.7741	837.3907	13
10	1090.6295	545.8184	1073.6030	537.3051			Y	***1563.7260***	782.3667	1546.6995	773.8534	1545.7155	773.3614	12
11	1203.7136	602.3604	1186.6871	593.8472			L	***1400.6627***	700.8350	1383.6362	692.3217	1382.6521	691.8297	11
12	1331.7722	666.3897	1314.7456	657.8765			Q	***1287.5786***	644.2930	1270.5521	635.7797	1269.5681	635.2877	10
13	1459.8308	730.4190	1442.8042	721.9057			Q	***1159.5201***	580.2637	1142.4935	571.7504	1141.5095	571.2584	9
14	1619.8614	810.4343	1602.8349	801.9211			C	***1031.4615***	516.2344	1014.4349	507.7211	1013.4509	507.2291	8
15	1716.9142	858.9607	1699.8876	850.4475			P	***871.4308***	436.2191	854.4043	427.7058	853.4203	427.2138	7
16	1863.9826	932.4949	1846.9560	923.9817			F	***774.3781***	387.6927	757.3515	379.1794	756.3675	378.6874	6
17	1993.0252	997.0162	1975.9986	988.5030	1975.0146	988.0109	E	***627.3097***	314.1585	610.2831	305.6452	609.2991	305.1532	5
18	2108.0521	1054.5297	2091.0256	1046.0164	2090.0416	1045.5244	D	***498.2671***	249.6372	481.2405	241.1239	480.2565	240.6319	4
19	2245.1110	1123.0592	2228.0845	1114.5459	2227.1005	1114.0539	H	***383.2401***	192.1237	366.2136	183.6104			3

**20**	2344.1795	1172.5934	2327.1529	1164.0801	2326.1689	1163.5881	**V**	246.1812	123.5942	229.1547	115.0810			**2**
**21**							**K**	147.1128	74.0600	130.0863	65.5468			**1**

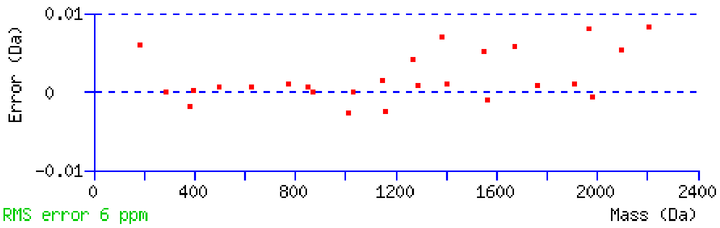

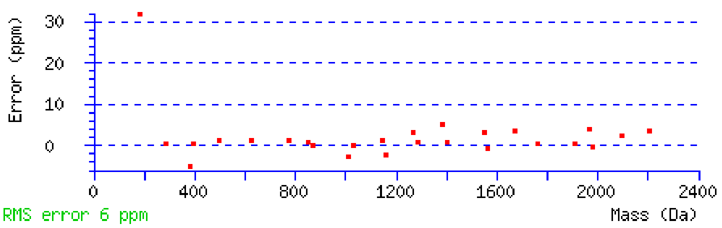

NCBI **BLAST** search of ALVLIAFAQYLQQCPFEDHVK
(Parameters: blastp, nr protein database, expect=20000, no filter, PAM30)
Other BLAST web gateways

All matches to this query

Score	Mr(calc):	Delta	Sequence
120.2	2489.2777	0.0045	ALVLIAFAQYLQQCPFEDHVK

Mascot: http://www.matrixscience.com/

Mascot Search Results

Peptide View

MS/MS Fragmentation of **ALVLIAFAQYLQQCPFEDHVK**
Found in **sp|P02768|ALBU_HUMAN**, Serum albumin OS=Homo sapiens GN=ALB PE=1 SV=2

Match to Query 13093: 2489.278332 from(830.766720,3+) intensity(88157432.0000) scans(17873) rtinseconds(3119) index(15704)
Title: 150825_TTSH_Patient_Plasma_15_Spectrum033716_scans__17873_RTINSECONDS=3119
Data file L:\\Ard_TTSH\\T1D\\T150825_TTSH_Patient_Plasma_15.mgf

Click mouse within plot area to zoom in by factor of two about that point
Or, Plot from 0 to 2400 Da Full range
Label all possible matches ○ Label matches used for scoring ⦿

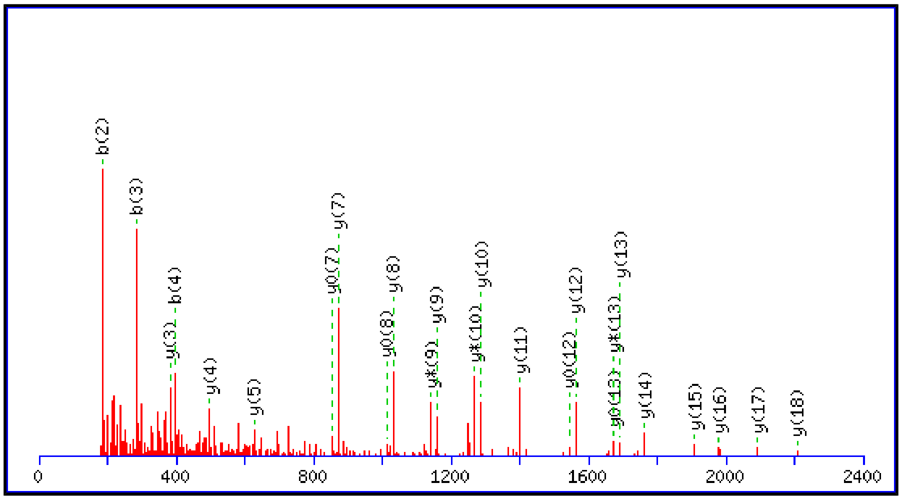

Monoisotopic mass of neutral peptide Mr(calc): 2489.2777
Variable modifications:
C14 : Carbamidomethyl (C)
Ions Score: 120 **Expect:** 3.8e-010
Matches : 25/186 fragment ions using 36 most intense peaks (help)

#	b	b^{++}	b*	b^{*++}	b^{0}	b^{0++}	Seq.	y	y^{++}	y*	y^{*++}	y^{0}	y^{0++}	#
1	72.0444	36.5258					A							21
2	**185.1285**	93.0679					L	2419.2479	1210.1276	2402.2213	1201.6143	2401.2373	1201.1223	20
3	**284.1969**	142.6021					V	2306.1638	1153.5855	2289.1373	1145.0723	2288.1532	1144.5803	19
4	**397.2809**	199.1441					L	***2207.0954***	1104.0513	2190.0688	1095.5381	2189.0848	1095.0461	18
5	510.3650	255.6861					I	***2094.0113***	1047.5093	2076.9848	1038.9960	2076.0008	1038.5040	17
6	581.4021	291.2047					A	***1980.9273***	990.9673	1963.9007	982.4540	1962.9167	981.9620	16
7	728.4705	364.7389					F	***1909.8901***	955.4487	1892.8636	946.9354	1891.8796	946.4434	15
8	799.5076	400.2575					A	***1762.8217***	881.9145	1745.7952	873.4012	1744.8112	872.9092	14
9	927.5662	464.2867	910.5397	455.7735			Q	***1691.7846***	846.3959	1674.7581	837.8827	1673.7741	837.3907	13
10	1090.6295	545.8184	1073.6030	537.3051			Y	***1563.7260***	782.3667	1546.6995	773.8534	1545.7155	773.3614	12
11	1203.7136	602.3604	1186.6871	593.8472			L	***1400.6627***	700.8350	1383.6362	692.3217	1382.6521	691.8297	11
12	1331.7722	666.3897	1314.7456	657.8765			Q	***1287.5786***	644.2930	1270.5521	635.7797	1269.5681	635.2877	10
13	1459.8308	730.4190	1442.8042	721.9057			Q	***1159.5201***	580.2637	1142.4935	571.7504	1141.5095	571.2584	9
14	1619.8614	810.4343	1602.8349	801.9211			C	***1031.4615***	516.2344	1014.4349	507.7211	1013.4509	507.2291	8
15	1716.9142	858.9607	1699.8876	850.4475			P	***871.4308***	436.2191	854.4043	427.7058	853.4203	427.2138	7
16	1863.9826	932.4949	1846.9560	923.9817			F	774.3781	387.6927	757.3515	379.1794	756.3675	378.6874	6
17	1993.0252	997.0162	1975.9986	988.5030	1975.0146	988.0109	E	***627.3097***	314.1585	610.2831	305.6452	609.2991	305.1532	5
18	2108.0521	1054.5297	2091.0256	1046.0164	2090.0416	1045.5244	D	***498.2671***	249.6372	481.2405	241.1239	480.2565	240.6319	4
19	2245.1110	1123.0592	2228.0845	1114.5459	2227.1005	1114.0539	H	***383.2401***	192.1237	366.2136	183.6104			3

**20**	2344.1795	1172.5934	2327.1529	1164.0801	2326.1689	1163.5881	**V**	246.1812	123.5942	229.1547	115.0810			**2**
**21**							**K**	147.1128	74.0600	130.0863	65.5468			**1**

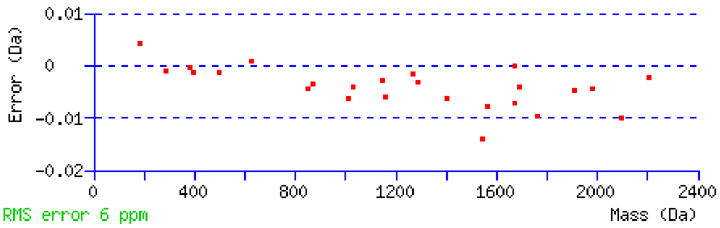

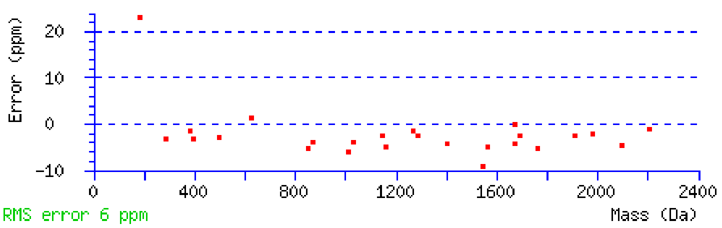

NCBI **BLAST** search of ALVLIAFAQYLQQCPFEDHVK
(Parameters: blastp, nr protein database, expect=20000, no filter, PAM30)
Other BLAST web gateways

All matches to this query

Score	Mr(calc):	Delta	Sequence
120.0	2489.2777	0.0006	ALVLIAFAQYLQQCPFEDHVK

**Mascot:** http://www.matrixscience.com/

Mascot Search Results

Peptide View

MS/MS Fragmentation of **ALVLIAFAQYLQQCPFEDHVK**
Found in **sp|P02768|ALBU_HUMAN**, Serum albumin OS=Homo sapiens GN=ALB PE=1 SV=2

Match to Query 11628: 2489.273202 from(830.765010,3+) intensity(396126816.0000) scans(17736) rtinseconds(3219) index(14772)
Title: 150825_TTSH_Patient_Plasma_19_Spectrum031689_scans__17736_RTINSECONDS=3219
Data file L:\\Ard_TTSH\\T1D\\T150825_TTSH_Patient_Plasma_19.mgf

Click mouse within plot area to zoom in by factor of two about that point
Or, Plot from 0 to 2400 Da Full range
Label all possible matches ○ Label matches used for scoring ⊙

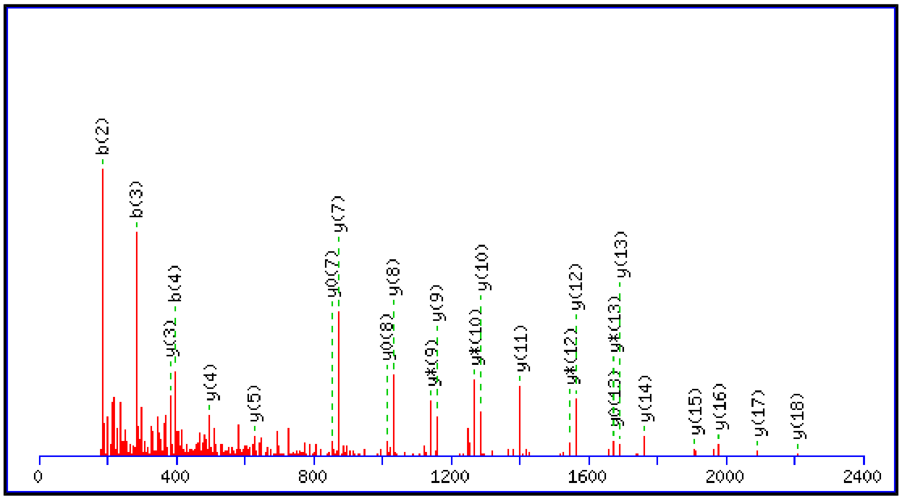

Monoisotopic mass of neutral peptide Mr(calc): 2489.2777
Variable modifications:
C14 : Carbamidomethyl (C)
Ions Score: 120 **Expect:** 3.4e-010
Matches : 25/186 fragment ions using 36 most intense peaks (help)

#	b	b++	b*	b*++	b0	b0++	Seq.	y	y++	y*	y*++	y0	y0++	#
1	72.0444	36.5258					A							21
2	**185.1285**	93.0679					L	2419.2479	1210.1276	2402.2213	1201.6143	2401.2373	1201.1223	20
3	**284.1969**	142.6021					V	2306.1638	1153.5855	2289.1373	1145.0723	2288.1532	1144.5803	19
4	**397.2809**	199.1441					L	***2207.0954***	1104.0513	2190.0688	1095.5381	2189.0848	1095.0461	18
5	510.3650	255.6861					I	***2094.0113***	1047.5093	2076.9848	1038.9960	2076.0008	1038.5040	17
6	581.4021	291.2047					A	***1980.9273***	990.9673	1963.9007	982.4540	1962.9167	981.9620	16
7	728.4705	364.7389					F	***1909.8901***	955.4487	1892.8636	946.9354	1891.8796	946.4434	15
8	799.5076	400.2575					A	***1762.8217***	881.9145	1745.7952	873.4012	1744.8112	872.9092	14
9	927.5662	464.2867	910.5397	455.7735			Q	***1691.7846***	846.3959	1674.7581	837.8827	1673.7741	837.3907	13
10	1090.6295	545.8184	1073.6030	537.3051			Y	***1563.7260***	782.3667	1546.6995	773.8534	1545.7155	773.3614	12
11	1203.7136	602.3604	1186.6871	593.8472			L	***1400.6627***	700.8350	1383.6362	692.3217	1382.6521	691.8297	11
12	1331.7722	666.3897	1314.7456	657.8765			Q	***1287.5786***	644.2930	1270.5521	635.7797	1269.5681	635.2877	10
13	1459.8308	730.4190	1442.8042	721.9057			Q	***1159.5201***	580.2637	1142.4935	571.7504	1141.5095	571.2584	9
14	1619.8614	810.4343	1602.8349	801.9211			C	***1031.4615***	516.2344	1014.4349	507.7211	1013.4509	507.2291	8
15	1716.9142	858.9607	1699.8876	850.4475			P	***871.4308***	436.2191	854.4043	427.7058	853.4203	427.2138	7
16	1863.9826	932.4949	1846.9560	923.9817			F	774.3781	387.6927	757.3515	379.1794	756.3675	378.6874	6
17	1993.0252	997.0162	1975.9986	988.5030	1975.0146	988.0109	E	***627.3097***	314.1585	610.2831	305.6452	609.2991	305.1532	5
18	2108.0521	1054.5297	2091.0256	1046.0164	2090.0416	1045.5244	D	***498.2671***	249.6372	481.2405	241.1239	480.2565	240.6319	4
19	2245.1110	1123.0592	2228.0845	1114.5459	2227.1005	1114.0539	H	***383.2401***	192.1237	366.2136	183.6104			3

**20**	2344.1795	1172.5934	2327.1529	1164.0801	2326.1689	1163.5881	**V**	246.1812	123.5942	229.1547	115.0810			**2**
**21**							**K**	147.1128	74.0600	130.0863	65.5468			**1**

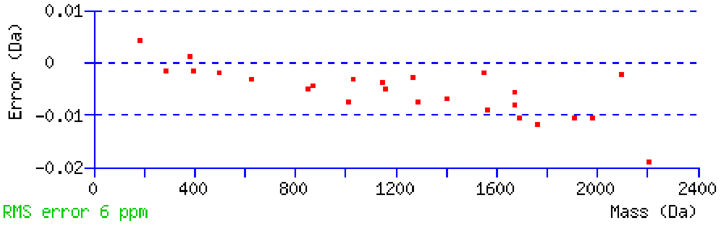

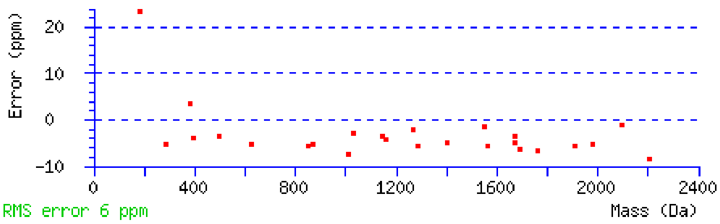

NCBI **BLAST** search of ALVLIAFAQYLQQCPFEDHVK
(Parameters: blastp, nr protein database, expect=20000, no filter, PAM30)
Other BLAST web gateways

All matches to this query

Score	Mr(calc):	Delta	Sequence
120.0	2489.2777	-0.0045	ALVLIAFAQYLQQCPFEDHVK

**Mascot:** http://www.matrixscience.com/

Mascot Search Results

Peptide View

MS/MS Fragmentation of **ALVLIAFAQYLQQCPFEDHVK**
Found in **sp|P02768|ALBU_HUMAN**, Serum albumin OS=Homo sapiens GN=ALB PE=1 SV=2

Match to Query 11136: 2489.274672 from(830.765500,3+) intensity(14852766.0000) scans(18323) rtinseconds(3192) index(15309)
Title: 150818_TTSH_Patient_Plasma_23_Spectrum033671_scans__18323_RTINSECONDS=3192
Data file L:\\Ard_TTSH\\T1D\\T150818_TTSH_Patient_Plasma_23.mgf

Click mouse within plot area to zoom in by factor of two about that point
Or, Plot from 0 to 2400 Da Full range
Label all possible matches ○ Label matches used for scoring ⊙

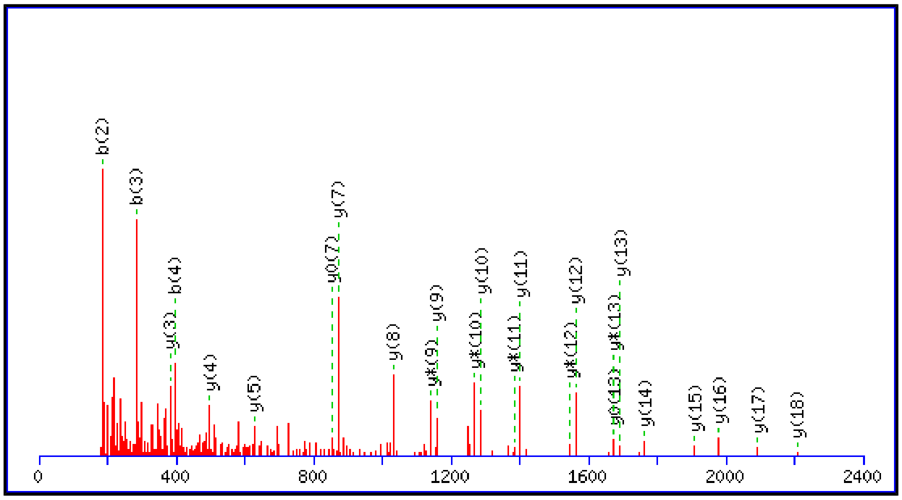

Monoisotopic mass of neutral peptide Mr(calc): 2489.2777
Variable modifications:
C14 : Carbamidomethyl (C)
Ions Score: 120 **Expect:** 3.6e-010
Matches : 25/186 fragment ions using 36 most intense peaks (help)

#	b	b^{++}	b*	b^{*++}	b^0	b^{0++}	Seq.	y	y^{++}	y*	y^{*++}	y^0	y^{0++}	#
1	72.0444	36.5258					A							21
2	**185.1285**	93.0679					L	2419.2479	1210.1276	2402.2213	1201.6143	2401.2373	1201.1223	20
3	**284.1969**	142.6021					V	2306.1638	1153.5855	2289.1373	1145.0723	2288.1532	1144.5803	19
4	**397.2809**	199.1441					L	***2207.0954***	1104.0513	2190.0688	1095.5381	2189.0848	1095.0461	18
5	510.3650	255.6861					I	***2094.0113***	1047.5093	2076.9848	1038.9960	2076.0008	1038.5040	17
6	581.4021	291.2047					A	***1980.9273***	990.9673	1963.9007	982.4540	1962.9167	981.9620	16
7	728.4705	364.7389					F	***1909.8901***	955.4487	1892.8636	946.9354	1891.8796	946.4434	15
8	799.5076	400.2575					A	***1762.8217***	881.9145	1745.7952	873.4012	1744.8112	872.9092	14
9	927.5662	464.2867	910.5397	455.7735			Q	***1691.7846***	846.3959	1674.7581	837.8827	1673.7741	837.3907	13
10	1090.6295	545.8184	1073.6030	537.3051			Y	***1563.7260***	782.3667	1546.6995	773.8534	1545.7155	773.3614	12
11	1203.7136	602.3604	1186.6871	593.8472			L	***1400.6627***	700.8350	1383.6362	692.3217	1382.6521	691.8297	11
12	1331.7722	666.3897	1314.7456	657.8765			Q	***1287.5786***	644.2930	1270.5521	635.7797	1269.5681	635.2877	10
13	1459.8308	730.4190	1442.8042	721.9057			Q	***1159.5201***	580.2637	1142.4935	571.7504	1141.5095	571.2584	9
14	1619.8614	810.4343	1602.8349	801.9211			C	***1031.4615***	516.2344	1014.4349	507.7211	1013.4509	507.2291	8
15	1716.9142	858.9607	1699.8876	850.4475			P	***871.4308***	436.2191	854.4043	427.7058	853.4203	427.2138	7
16	1863.9826	932.4949	1846.9560	923.9817			F	774.3781	387.6927	757.3515	379.1794	756.3675	378.6874	6
17	1993.0252	997.0162	1975.9986	988.5030	1975.0146	988.0109	E	***627.3097***	314.1585	610.2831	305.6452	609.2991	305.1532	5
18	2108.0521	1054.5297	2091.0256	1046.0164	2090.0416	1045.5244	D	***498.2671***	249.6372	481.2405	241.1239	480.2565	240.6319	4
19	2245.1110	1123.0592	2228.0845	1114.5459	2227.1005	1114.0539	H	***383.2401***	192.1237	366.2136	183.6104			3

**20**	2344.1795	1172.5934	2327.1529	1164.0801	2326.1689	1163.5881	**V**	246.1812	123.5942	229.1547	115.0810			**2**
**21**							**K**	147.1128	74.0600	130.0863	65.5468			**1**

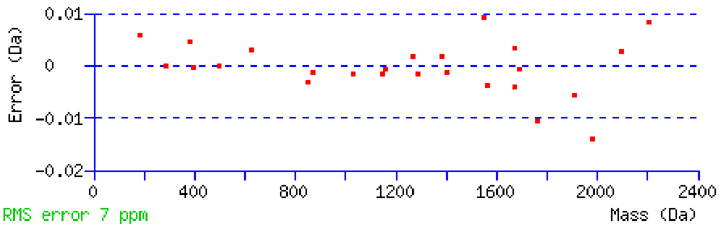

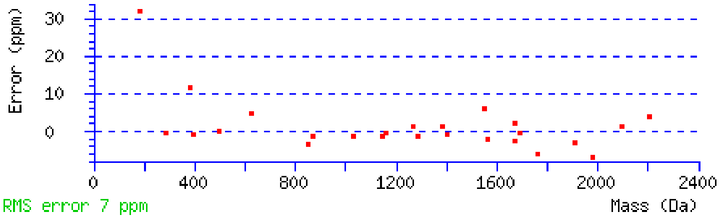

NCBI **BLAST** search of ALVLIAFAQYLQQCPFEDHVK
(Parameters: blastp, nr protein database, expect=20000, no filter, PAM30)
Other BLAST web gateways

All matches to this query

Score	Mr(calc):	Delta	Sequence
119.8	2489.2777	-0.0030	ALVLIAFAQYLQQCPFEDHVK

Mascot: http://www.matrixscience.com/

{MATRIX}{SCIENCE} Mascot Search Results

Peptide View

MS/MS Fragmentation of **ALVLIAFAQYLQQCPFEDHVK**
Found in **sp|P02768|ALBU_HUMAN**, Serum albumin OS=Homo sapiens GN=ALB PE=1 SV=2

Match to Query 12501: 2489.276502 from(830.766110,3+) intensity(1701140.6250) scans(19303) rtinseconds(3376) index(16759)
Title: 150818_TTSH_Patient_Plasma_93_Spectrum034764_scans__19303_RTINSECONDS=3376
Data file L:\\Ard_TTSH\\T1D\\T150818_TTSH_Patient_Plasma_93.mgf

Click mouse within plot area to zoom in by factor of two about that point
Or, [Plot from] [0] to [2200] Da [Full range]
Label all possible matches ○ Label matches used for scoring ⊙

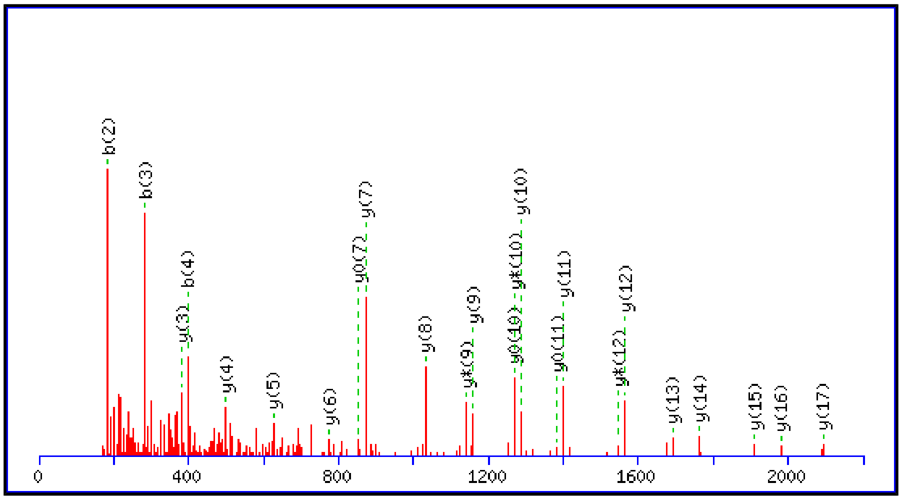

Monoisotopic mass of neutral peptide Mr(calc): 2489.2777
Variable modifications:
C14 : Carbamidomethyl (C)
Ions Score: 120 **Expect:** 3.7e-010
Matches : 24/186 fragment ions using 35 most intense peaks (help)

#	b	b^{++}	b*	b^{*++}	b^0	b^{0++}	Seq.	y	y^{++}	y*	y^{*++}	y^0	y^{0++}	#
1	72.0444	36.5258					A							21
2	**185.1285**	93.0679					L	2419.2479	1210.1276	2402.2213	1201.6143	2401.2373	1201.1223	20
3	**284.1969**	142.6021					V	2306.1638	1153.5855	2289.1373	1145.0723	2288.1532	1144.5803	19
4	**397.2809**	199.1441					L	2207.0954	1104.0513	2190.0688	1095.5381	2189.0848	1095.0461	18
5	510.3650	255.6861					I	***2094.0113***	1047.5093	2076.9848	1038.9960	2076.0008	1038.5040	17
6	581.4021	291.2047					A	***1980.9273***	990.9673	1963.9007	982.4540	1962.9167	981.9620	16
7	728.4705	364.7389					F	***1909.8901***	955.4487	1892.8636	946.9354	1891.8796	946.4434	15
8	799.5076	400.2575					A	***1762.8217***	881.9145	1745.7952	873.4012	1744.8112	872.9092	14
9	927.5662	464.2867	910.5397	455.7735			Q	***1691.7846***	846.3959	1674.7581	837.8827	1673.7741	837.3907	13
10	1090.6295	545.8184	1073.6030	537.3051			Y	***1563.7260***	782.3667	1546.6995	773.8534	1545.7155	773.3614	12
11	1203.7136	602.3604	1186.6871	593.8472			L	***1400.6627***	700.8350	1383.6362	692.3217	1382.6521	691.8297	11
12	1331.7722	666.3897	1314.7456	657.8765			Q	***1287.5786***	644.2930	1270.5521	635.7797	1269.5681	635.2877	10
13	1459.8308	730.4190	1442.8042	721.9057			Q	***1159.5201***	580.2637	1142.4935	571.7504	1141.5095	571.2584	9
14	1619.8614	810.4343	1602.8349	801.9211			C	***1031.4615***	516.2344	1014.4349	507.7211	1013.4509	507.2291	8
15	1716.9142	858.9607	1699.8876	850.4475			P	***871.4308***	436.2191	854.4043	427.7058	853.4203	427.2138	7
16	1863.9826	932.4949	1846.9560	923.9817			F	***774.3781***	387.6927	757.3515	379.1794	756.3675	378.6874	6
17	1993.0252	997.0162	1975.9986	988.5030	1975.0146	988.0109	E	***627.3097***	314.1585	610.2831	305.6452	609.2991	305.1532	5
18	2108.0521	1054.5297	2091.0256	1046.0164	2090.0416	1045.5244	D	***498.2671***	249.6372	481.2405	241.1239	480.2565	240.6319	4
19	2245.1110	1123.0592	2228.0845	1114.5459	2227.1005	1114.0539	H	***383.2401***	192.1237	366.2136	183.6104			3

**20**	2344.1795	1172.5934	2327.1529	1164.0801	2326.1689	1163.5881	**V**	246.1812	123.5942	229.1547	115.0810			**2**
**21**							**K**	147.1128	74.0600	130.0863	65.5468			**1**

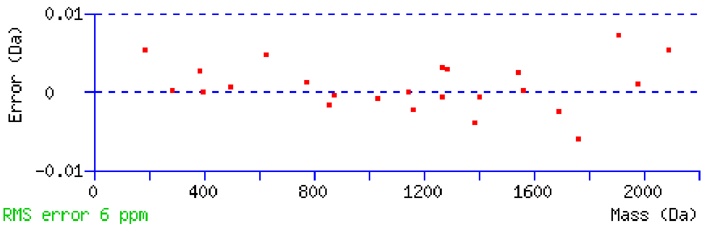

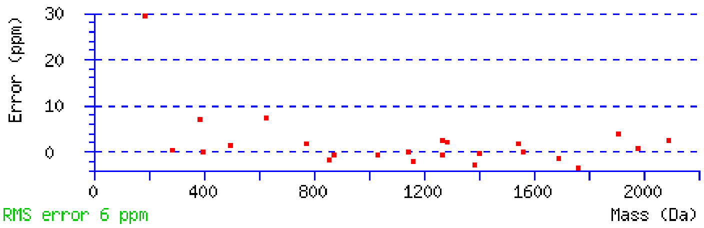

NCBI **BLAST** search of ALVLIAFAQYLQQCPFEDHVK
(Parameters: blastp, nr protein database, expect=20000, no filter, PAM30)
Other BLAST web gateways

All matches to this query

Score	Mr(calc):	Delta	Sequence
119.6	2489.2777	-0.0012	ALVLIAFAQYLQQCPFEDHVK

Mascot: http://www.matrixscience.com/

Mascot Search Results

Peptide View

MS/MS Fragmentation of **ALVLIAFAQYLQQCPFEDHVK**
Found in **sp|P02768|ALBU_HUMAN**, Serum albumin OS=Homo sapiens GN=ALB PE=1 SV=2

Match to Query 11653: 2489.273022 from(830.764950,3+) intensity(304908384.0000) scans(18014) rtinseconds(3110) index(15393)
Title: 150818_TTSH_Patient_Plasma_91_Spectrum033515_scans__18014_RTINSECONDS=3110
Data file L:\\Ard_TTSH\\T1D\\T150818_TTSH_Patient_Plasma_91.mgf

Click mouse within plot area to zoom in by factor of two about that point
Or, [Plot from] 0 to 2400 Da [Full range]
Label all possible matches ○ Label matches used for scoring ⦿

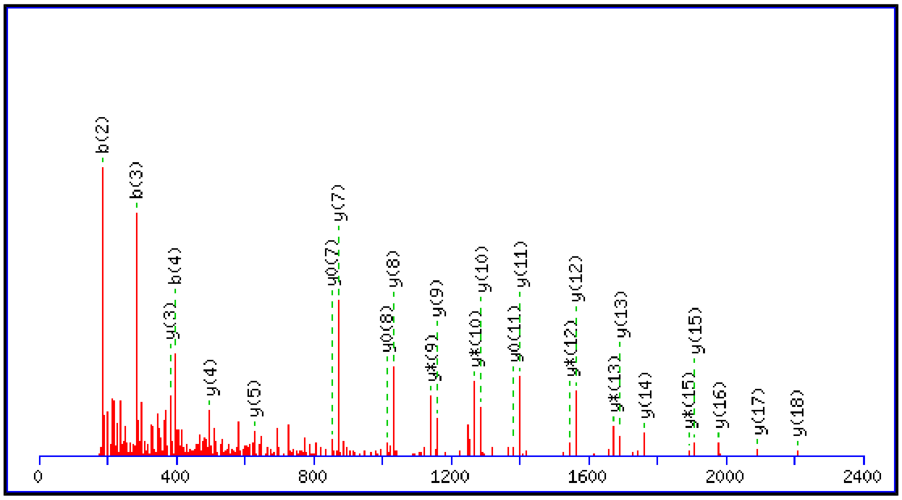

Monoisotopic mass of neutral peptide Mr(calc): 2489.2777
Variable modifications:
C14 : Carbamidomethyl (C)
Ions Score: 118 **Expect:** 5.6e-010
Matches : 26/186 fragment ions using 38 most intense peaks (help)

#	b	b^{++}	b*	b^{*++}	b^0	b^{0++}	Seq.	y	y^{++}	y*	y^{*++}	y^0	y^{0++}	#
1	72.0444	36.5258					A							21
2	185.1285	93.0679					L	2419.2479	1210.1276	2402.2213	1201.6143	2401.2373	1201.1223	20
3	284.1969	142.6021					V	2306.1638	1153.5855	2289.1373	1145.0723	2288.1532	1144.5803	19
4	397.2809	199.1441					L	2207.0954	1104.0513	2190.0688	1095.5381	2189.0848	1095.0461	18
5	510.3650	255.6861					I	2094.0113	1047.5093	2076.9848	1038.9960	2076.0008	1038.5040	17
6	581.4021	291.2047					A	1980.9273	990.9673	1963.9007	982.4540	1962.9167	981.9620	16
7	728.4705	364.7389					F	1909.8901	955.4487	1892.8636	946.9354	1891.8796	946.4434	15
8	799.5076	400.2575					A	1762.8217	881.9145	1745.7952	873.4012	1744.8112	872.9092	14
9	927.5662	464.2867	910.5397	455.7735			Q	1691.7846	846.3959	1674.7581	837.8827	1673.7741	837.3907	13
10	1090.6295	545.8184	1073.6030	537.3051			Y	1563.7260	782.3667	1546.6995	773.8534	1545.7155	773.3614	12
11	1203.7136	602.3604	1186.6871	593.8472			L	1400.6627	700.8350	1383.6362	692.3217	1382.6521	691.8297	11
12	1331.7722	666.3897	1314.7456	657.8765			Q	1287.5786	644.2930	1270.5521	635.7797	1269.5681	635.2877	10
13	1459.8308	730.4190	1442.8042	721.9057			Q	1159.5201	580.2637	1142.4935	571.7504	1141.5095	571.2584	9
14	1619.8614	810.4343	1602.8349	801.9211			C	1031.4615	516.2344	1014.4349	507.7211	1013.4509	507.2291	8
15	1716.9142	858.9607	1699.8876	850.4475			P	871.4308	436.2191	854.4043	427.7058	853.4203	427.2138	7
16	1863.9826	932.4949	1846.9560	923.9817			F	774.3781	387.6927	757.3515	379.1794	756.3675	378.6874	6
17	1993.0252	997.0162	1975.9986	988.5030	1975.0146	988.0109	E	627.3097	314.1585	610.2831	305.6452	609.2991	305.1532	5
18	2108.0521	1054.5297	2091.0256	1046.0164	2090.0416	1045.5244	D	498.2671	249.6372	481.2405	241.1239	480.2565	240.6319	4
19	2245.1110	1123.0592	2228.0845	1114.5459	2227.1005	1114.0539	H	383.2401	192.1237	366.2136	183.6104			3

**20**	2344.1795	1172.5934	2327.1529	1164.0801	2326.1689	1163.5881	**V**	246.1812	123.5942	229.1547	115.0810			**2**
**21**							**K**	147.1128	74.0600	130.0863	65.5468			**1**

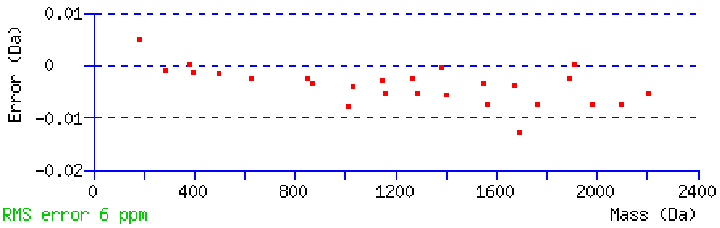

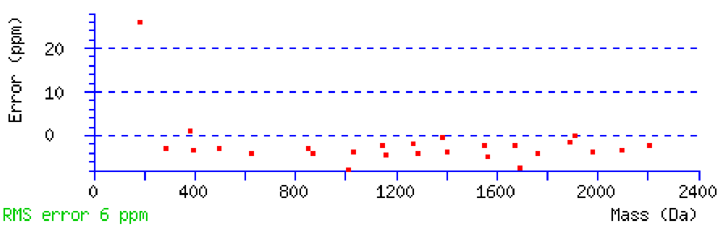

NCBI **BLAST** search of ALVLIAFAQYLQQCPFEDHVK
(Parameters: blastp, nr protein database, expect=20000, no filter, PAM30)
Other BLAST web gateways

All matches to this query

Score	Mr(calc):	Delta	Sequence
117.8	2489.2777	-0.0047	ALVLIAFAQYLQQCPFEDHVK

Mascot: http://www.matrixscience.com/

Mascot Search Results

Peptide View

MS/MS Fragmentation of **ALVLIAFAQYLQQCPFEDHVK**
Found in **sp|P02768|ALBU_HUMAN**, Serum albumin OS=Homo sapiens GN=ALB PE=1 SV=2

Match to Query 12409: 2489.279262 from(830.767030,3+) intensity(14321033.0000) scans(17692) rtinseconds(3046) index(15350)
Title: 150818_TTSH_Patient_Plasma_41_Spectrum033982_scans__17692_RTINSECONDS=3046
Data file L:\\Ard_TTSH\\T1D\\T150818_TTSH_Patient_Plasma_41.mgf

Click mouse within plot area to zoom in by factor of two about that point
Or, Plot from 0 to 2400 Da Full range
Label all possible matches ○ Label matches used for scoring ⊙

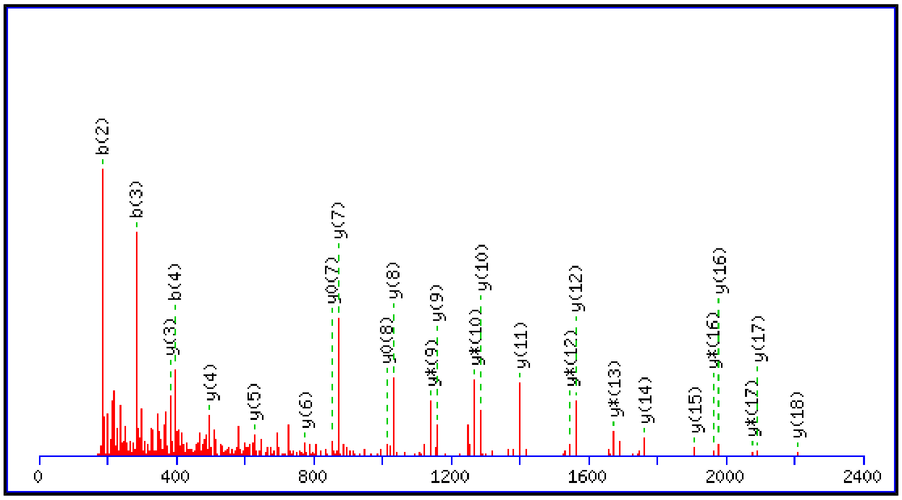

Monoisotopic mass of neutral peptide Mr(calc): 2489.2777
Variable modifications:
C14 : Carbamidomethyl (C)
Ions Score: 118 **Expect:** 6.2e-010
Matches : 26/186 fragment ions using 38 most intense peaks (help)

#	b	b^{++}	b*	b^{*++}	b^0	b^{0++}	Seq.	y	y^{++}	y*	y^{*++}	y^0	y^{0++}	#
1	72.0444	36.5258					A							21
2	**185.1285**	93.0679					L	2419.2479	1210.1276	2402.2213	1201.6143	2401.2373	1201.1223	20
3	**284.1969**	142.6021					V	2306.1638	1153.5855	2289.1373	1145.0723	2288.1532	1144.5803	19
4	**397.2809**	199.1441					L	***2207.0954***	1104.0513	2190.0688	1095.5381	2189.0848	1095.0461	18
5	510.3650	255.6861					I	***2094.0113***	1047.5093	2076.9848	1038.9960	2076.0008	1038.5040	17
6	581.4021	291.2047					A	***1980.9273***	990.9673	1963.9007	982.4540	1962.9167	981.9620	16
7	728.4705	364.7389					F	***1909.8901***	955.4487	1892.8636	946.9354	1891.8796	946.4434	15
8	799.5076	400.2575					A	***1762.8217***	881.9145	1745.7952	873.4012	1744.8112	872.9092	14
9	927.5662	464.2867	910.5397	455.7735			Q	1691.7846	846.3959	1674.7581	837.8827	1673.7741	837.3907	13
10	1090.6295	545.8184	1073.6030	537.3051			Y	***1563.7260***	782.3667	1546.6995	773.8534	1545.7155	773.3614	12
11	1203.7136	602.3604	1186.6871	593.8472			L	***1400.6627***	700.8350	1383.6362	692.3217	1382.6521	691.8297	11
12	1331.7722	666.3897	1314.7456	657.8765			Q	***1287.5786***	644.2930	1270.5521	635.7797	1269.5681	635.2877	10
13	1459.8308	730.4190	1442.8042	721.9057			Q	***1159.5201***	580.2637	1142.4935	571.7504	1141.5095	571.2584	9
14	1619.8614	810.4343	1602.8349	801.9211			C	***1031.4615***	516.2344	1014.4349	507.7211	1013.4509	507.2291	8
15	1716.9142	858.9607	1699.8876	850.4475			P	***871.4308***	436.2191	854.4043	427.7058	853.4203	427.2138	7
16	1863.9826	932.4949	1846.9560	923.9817			F	***774.3781***	387.6927	757.3515	379.1794	756.3675	378.6874	6
17	1993.0252	997.0162	1975.9986	988.5030	1975.0146	988.0109	E	***627.3097***	314.1585	610.2831	305.6452	609.2991	305.1532	5
18	2108.0521	1054.5297	2091.0256	1046.0164	2090.0416	1045.5244	D	***498.2671***	249.6372	481.2405	241.1239	480.2565	240.6319	4
19	2245.1110	1123.0592	2228.0845	1114.5459	2227.1005	1114.0539	H	***383.2401***	192.1237	366.2136	183.6104			3

**20**	2344.1795	1172.5934	2327.1529	1164.0801	2326.1689	1163.5881	**V**	246.1812	123.5942	229.1547	115.0810			**2**
**21**							**K**	147.1128	74.0600	130.0863	65.5468			**1**

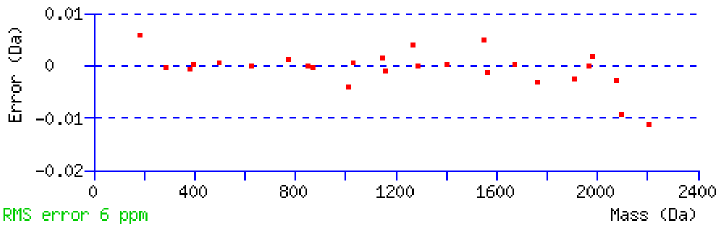

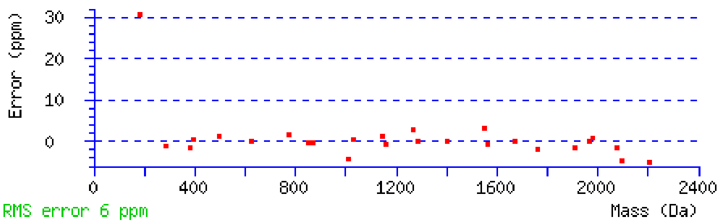

NCBI **BLAST** search of ALVLIAFAQYLQQCPFEDHVK
(Parameters: blastp, nr protein database, expect=20000, no filter, PAM30)
Other BLAST web gateways

All matches to this query

Score	Mr(calc):	Delta	Sequence
117.8	2489.2777	0.0016	ALVLIAFAQYLQQCPFEDHVK

Mascot: http://www.matrixscience.com/

Mascot Search Results

Peptide View

MS/MS Fragmentation of **ALVLIAFAQYLQQCPFEDHVK**
Found in **sp|P02768|ALBU_HUMAN**, Serum albumin OS=Homo sapiens GN=ALB PE=1 SV=2

Match to Query 12050: 2489.279442 from(830.767090,3+) intensity(31867814.0000) scans(19025) rtinseconds(3383) index(16218)
Title: 150818_TTSH_Patient_Plasma_94_Spectrum033687_scans__19025_RTINSECONDS=3383
Data file L:\\Ard_TTSH\\T1D\\T150818_TTSH_Patient_Plasma_94.mgf

Click mouse within plot area to zoom in by factor of two about that point
Or, Plot from 0 to 2400 Da Full range
Label all possible matches ○ Label matches used for scoring ⦿

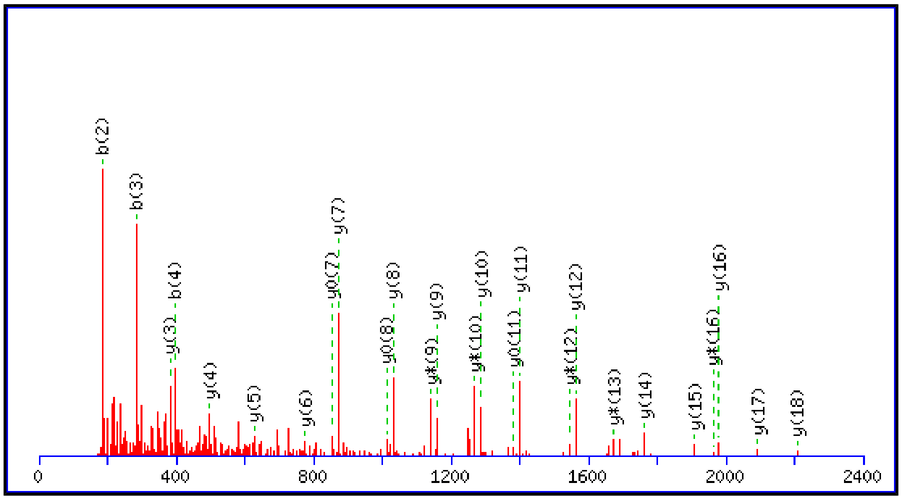

Monoisotopic mass of neutral peptide Mr(calc): 2489.2777
Variable modifications:
C14 : Carbamidomethyl (C)
Ions Score: 118 **Expect:** 6.3e-010
Matches : 26/186 fragment ions using 38 most intense peaks (help)

#	b	b^{++}	b*	b^{*++}	b^0	b^{0++}	Seq.	y	y^{++}	y*	y^{*++}	y^0	y^{0++}	#
1	72.0444	36.5258					A							21
2	**185.1285**	93.0679					L	2419.2479	1210.1276	2402.2213	1201.6143	2401.2373	1201.1223	20
3	**284.1969**	142.6021					V	2306.1638	1153.5855	2289.1373	1145.0723	2288.1532	1144.5803	19
4	**397.2809**	199.1441					L	***2207.0954***	1104.0513	2190.0688	1095.5381	2189.0848	1095.0461	18
5	510.3650	255.6861					I	***2094.0113***	1047.5093	2076.9848	1038.9960	2076.0008	1038.5040	17
6	581.4021	291.2047					A	***1980.9273***	990.9673	1963.9007	982.4540	1962.9167	981.9620	16
7	728.4705	364.7389					F	***1909.8901***	955.4487	1892.8636	946.9354	1891.8796	946.4434	15
8	799.5076	400.2575					A	***1762.8217***	881.9145	1745.7952	873.4012	1744.8112	872.9092	14
9	927.5662	464.2867	910.5397	455.7735			Q	1691.7846	846.3959	1674.7581	837.8827	1673.7741	837.3907	13
10	1090.6295	545.8184	1073.6030	537.3051			Y	***1563.7260***	782.3667	1546.6995	773.8534	1545.7155	773.3614	12
11	1203.7136	602.3604	1186.6871	593.8472			L	***1400.6627***	700.8350	1383.6362	692.3217	1382.6521	691.8297	11
12	1331.7722	666.3897	1314.7456	657.8765			Q	***1287.5786***	644.2930	1270.5521	635.7797	1269.5681	635.2877	10
13	1459.8308	730.4190	1442.8042	721.9057			Q	***1159.5201***	580.2637	1142.4935	571.7504	1141.5095	571.2584	9
14	1619.8614	810.4343	1602.8349	801.9211			C	***1031.4615***	516.2344	1014.4349	507.7211	1013.4509	507.2291	8
15	1716.9142	858.9607	1699.8876	850.4475			P	***871.4308***	436.2191	854.4043	427.7058	853.4203	427.2138	7
16	1863.9826	932.4949	1846.9560	923.9817			F	***774.3781***	387.6927	757.3515	379.1794	756.3675	378.6874	6
17	1993.0252	997.0162	1975.9986	988.5030	1975.0146	988.0109	E	***627.3097***	314.1585	610.2831	305.6452	609.2991	305.1532	5
18	2108.0521	1054.5297	2091.0256	1046.0164	2090.0416	1045.5244	D	***498.2671***	249.6372	481.2405	241.1239	480.2565	240.6319	4
19	2245.1110	1123.0592	2228.0845	1114.5459	2227.1005	1114.0539	H	***383.2401***	192.1237	366.2136	183.6104			3

**20**	2344.1795	1172.5934	2327.1529	1164.0801	2326.1689	1163.5881	**V**	246.1812	123.5942	229.1547	115.0810			**2**
**21**							**K**	147.1128	74.0600	130.0863	65.5468			**1**

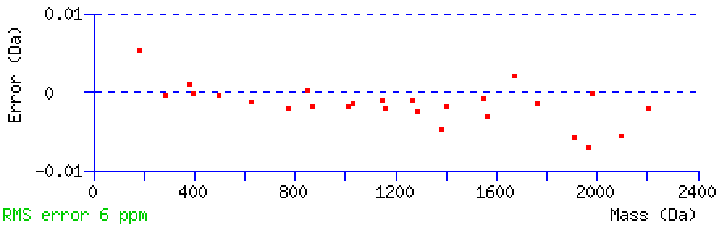

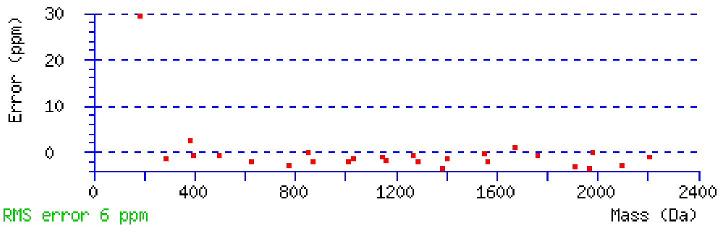

NCBI **BLAST** search of ALVLIAFAQYLQQCPFEDHVK
(Parameters: blastp, nr protein database, expect=20000, no filter, PAM30)
Other BLAST web gateways

All matches to this query

Score	Mr(calc):	Delta	Sequence
117.7	2489.2777	0.0017	ALVLIAFAQYLQQCPFEDHVK

**Mascot:** http://www.matrixscience.com/

Mascot Search Results

Peptide View

MS/MS Fragmentation of **ALVLIAFAQYLQQCPFEDHVK**
Found in **sp|P02768|ALBU_HUMAN**, Serum albumin OS=Homo sapiens GN=ALB PE=1 SV=2

Match to Query 12403: 2489.277252 from(830.766360,3+) intensity(96682992.0000) scans(17787) rtinseconds(3111) index(15520)
Title: 150818_TTSH_Patient_Plasma_92_Spectrum033433_scans__17787_RTINSECONDS=3111
Data file L:\\Ard_TTSH\\T1D\\T150818_TTSH_Patient_Plasma_92.mgf

Click mouse within plot area to zoom in by factor of two about that point
Or, [Plot from] [0] to [2400] Da [Full range]
Label all possible matches ○ Label matches used for scoring ⊙

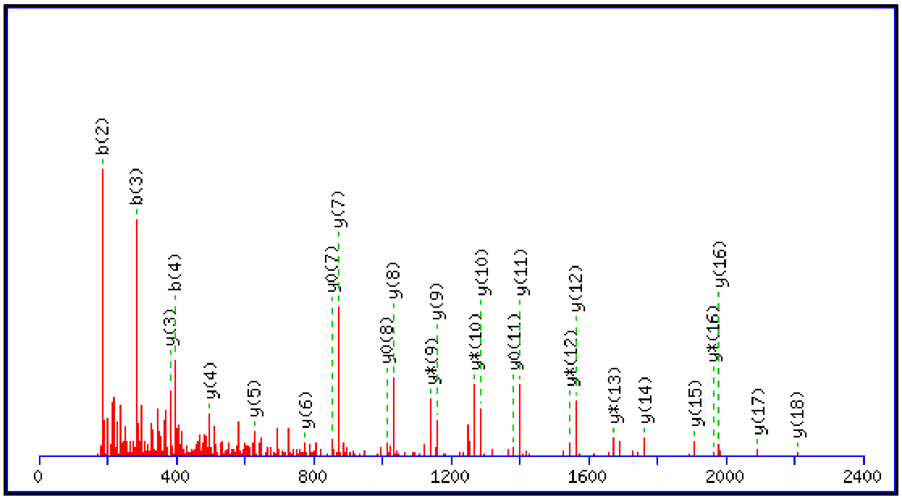

Monoisotopic mass of neutral peptide Mr(calc): 2489.2777
Variable modifications:
C14 : Carbamidomethyl (C)
Ions Score: 118 **Expect:** 5.5e-010
Matches : 26/186 fragment ions using 38 most intense peaks (help)

#	b	b^{++}	b*	b^{*++}	b^0	b^{0++}	Seq.	y	y^{++}	y*	y^{*++}	y^0	y^{0++}	#
1	72.0444	36.5258					A							21
2	**185.1285**	93.0679					L	2419.2479	1210.1276	2402.2213	1201.6143	2401.2373	1201.1223	20
3	**284.1969**	142.6021					V	2306.1638	1153.5855	2289.1373	1145.0723	2288.1532	1144.5803	19
4	**397.2809**	199.1441					L	***2207.0954***	1104.0513	2190.0688	1095.5381	2189.0848	1095.0461	18
5	510.3650	255.6861					I	***2094.0113***	1047.5093	2076.9848	1038.9960	2076.0008	1038.5040	17
6	581.4021	291.2047					A	***1980.9273***	990.9673	1963.9007	982.4540	1962.9167	981.9620	16
7	728.4705	364.7389					F	***1909.8901***	955.4487	1892.8636	946.9354	1891.8796	946.4434	15
8	799.5076	400.2575					A	***1762.8217***	881.9145	1745.7952	873.4012	1744.8112	872.9092	14
9	927.5662	464.2867	910.5397	455.7735			Q	1691.7846	846.3959	1674.7581	837.8827	1673.7741	837.3907	13
10	1090.6295	545.8184	1073.6030	537.3051			Y	***1563.7260***	782.3667	1546.6995	773.8534	1545.7155	773.3614	12
11	1203.7136	602.3604	1186.6871	593.8472			L	***1400.6627***	700.8350	1383.6362	692.3217	1382.6521	691.8297	11
12	1331.7722	666.3897	1314.7456	657.8765			Q	***1287.5786***	644.2930	1270.5521	635.7797	1269.5681	635.2877	10
13	1459.8308	730.4190	1442.8042	721.9057			Q	***1159.5201***	580.2637	1142.4935	571.7504	1141.5095	571.2584	9
14	1619.8614	810.4343	1602.8349	801.9211			C	***1031.4615***	516.2344	1014.4349	507.7211	1013.4509	507.2291	8
15	1716.9142	858.9607	1699.8876	850.4475			P	***871.4308***	436.2191	854.4043	427.7058	853.4203	427.2138	7
16	1863.9826	932.4949	1846.9560	923.9817			F	***774.3781***	387.6927	757.3515	379.1794	756.3675	378.6874	6
17	1993.0252	997.0162	1975.9986	988.5030	1975.0146	988.0109	E	***627.3097***	314.1585	610.2831	305.6452	609.2991	305.1532	5
18	2108.0521	1054.5297	2091.0256	1046.0164	2090.0416	1045.5244	D	***498.2671***	249.6372	481.2405	241.1239	480.2565	240.6319	4
19	2245.1110	1123.0592	2228.0845	1114.5459	2227.1005	1114.0539	H	***383.2401***	192.1237	366.2136	183.6104			3

**20**	2344.1795	1172.5934	2327.1529	1164.0801	2326.1689	1163.5881	**V**	246.1812	123.5942	229.1547	115.0810			**2**
**21**							**K**	147.1128	74.0600	130.0863	65.5468			**1**

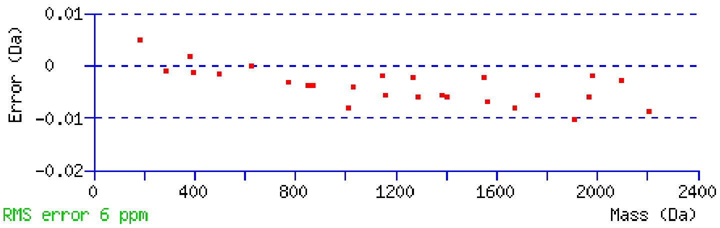

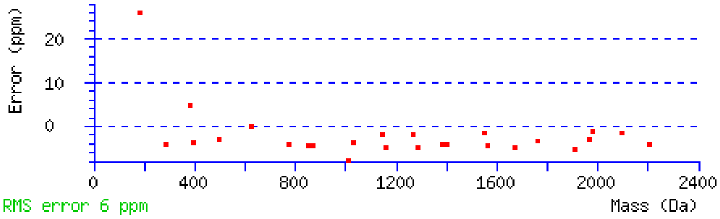

NCBI **BLAST** search of ALVLIAFAQYLQQCPFEDHVK
(Parameters: blastp, nr protein database, expect=20000, no filter, PAM30)
Other BLAST web gateways

All matches to this query

Score	Mr(calc):	Delta	Sequence
117.7	2489.2777	-0.0004	ALVLIAFAQYLQQCPFEDHVK

Mascot: http://www.matrixscience.com/

Mascot Search Results

Peptide View

MS/MS Fragmentation of **ALVLIAFAQYLQQCPFEDHVK**
Found in **sp|P02768|ALBU_HUMAN**, Serum albumin OS=Homo sapiens GN=ALB PE=1 SV=2

Match to Query 12173: 2489.276142 from(830.765990,3+) intensity(57659252.0000) scans(17171) rtinseconds(3027) index(14778)
Title: 150818_TTSH_Patient_Plasma_95_Spectrum032436_scans__17171_RTINSECONDS=3027
Data file L:\\Ard_TTSH\\T1D\\T150818_TTSH_Patient_Plasma_95.mgf

Click mouse within plot area to zoom in by factor of two about that point
Or, [Plot from] [0] to [2400] Da [Full range]
Label all possible matches ○ Label matches used for scoring ⊙

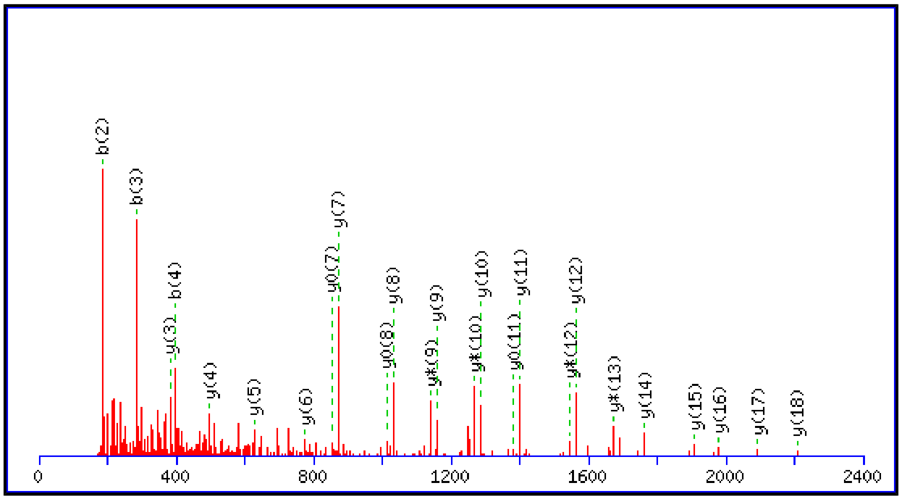

Monoisotopic mass of neutral peptide Mr(calc): 2489.2777
Variable modifications:
C14 : Carbamidomethyl (C)
Ions Score: 117 **Expect:** 6e-010
Matches : 25/186 fragment ions using 37 most intense peaks (help)

#	b	b++	b*	b*++	b0	b0++	Seq.	y	y++	y*	y*++	y0	y0++	#
1	72.0444	36.5258					A							21
2	**185.1285**	93.0679					L	2419.2479	1210.1276	2402.2213	1201.6143	2401.2373	1201.1223	20
3	**284.1969**	142.6021					V	2306.1638	1153.5855	2289.1373	1145.0723	2288.1532	1144.5803	19
4	**397.2809**	199.1441					L	***2207.0954***	1104.0513	2190.0688	1095.5381	2189.0848	1095.0461	18
5	510.3650	255.6861					I	***2094.0113***	1047.5093	2076.9848	1038.9960	2076.0008	1038.5040	17
6	581.4021	291.2047					A	***1980.9273***	990.9673	1963.9007	982.4540	1962.9167	981.9620	16
7	728.4705	364.7389					F	***1909.8901***	955.4487	1892.8636	946.9354	1891.8796	946.4434	15
8	799.5076	400.2575					A	***1762.8217***	881.9145	1745.7952	873.4012	1744.8112	872.9092	14
9	927.5662	464.2867	910.5397	455.7735			Q	1691.7846	846.3959	1674.7581	837.8827	1673.7741	837.3907	13
10	1090.6295	545.8184	1073.6030	537.3051			Y	***1563.7260***	782.3667	1546.6995	773.8534	1545.7155	773.3614	12
11	1203.7136	602.3604	1186.6871	593.8472			L	***1400.6627***	700.8350	1383.6362	692.3217	1382.6521	691.8297	11
12	1331.7722	666.3897	1314.7456	657.8765			Q	***1287.5786***	644.2930	1270.5521	635.7797	1269.5681	635.2877	10
13	1459.8308	730.4190	1442.8042	721.9057			Q	***1159.5201***	580.2637	1142.4935	571.7504	1141.5095	571.2584	9
14	1619.8614	810.4343	1602.8349	801.9211			C	***1031.4615***	516.2344	1014.4349	507.7211	1013.4509	507.2291	8
15	1716.9142	858.9607	1699.8876	850.4475			P	***871.4308***	436.2191	854.4043	427.7058	853.4203	427.2138	7
16	1863.9826	932.4949	1846.9560	923.9817			F	***774.3781***	387.6927	757.3515	379.1794	756.3675	378.6874	6
17	1993.0252	997.0162	1975.9986	988.5030	1975.0146	988.0109	E	***627.3097***	314.1585	610.2831	305.6452	609.2991	305.1532	5
18	2108.0521	1054.5297	2091.0256	1046.0164	2090.0416	1045.5244	D	***498.2671***	249.6372	481.2405	241.1239	480.2565	240.6319	4
19	2245.1110	1123.0592	2228.0845	1114.5459	2227.1005	1114.0539	H	***383.2401***	192.1237	366.2136	183.6104			3

**20**	2344.1795	1172.5934	2327.1529	1164.0801	2326.1689	1163.5881	**V**	246.1812	123.5942	229.1547	115.0810			**2**
**21**							**K**	147.1128	74.0600	130.0863	65.5468			**1**

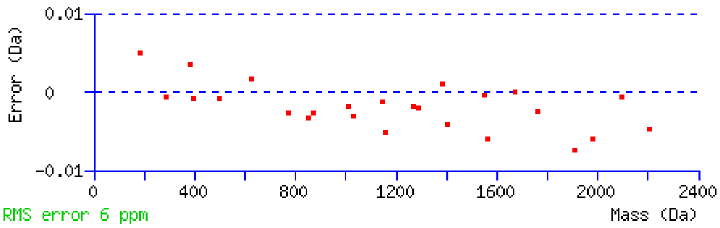

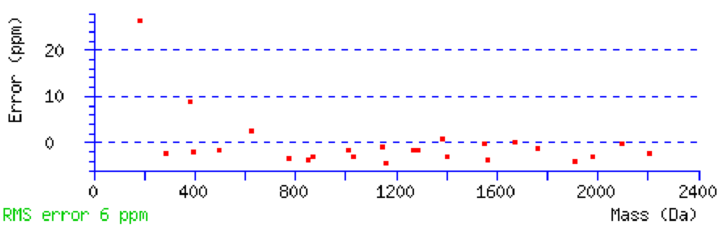

NCBI **BLAST** search of ALVLIAFAQYLQQCPFEDHVK
(Parameters: blastp, nr protein database, expect=20000, no filter, PAM30)
Other BLAST web gateways

All matches to this query

Score	Mr(calc):	Delta	Sequence
117.4	2489.2777	-0.0016	ALVLIAFAQYLQQCPFEDHVK

**Mascot:** http://www.matrixscience.com/

Mascot Search Results

Peptide View

MS/MS Fragmentation of **ALVLIAFAQYLQQCPFEDHVK**
Found in **sp|P02768|ALBU_HUMAN**, Serum albumin OS=Homo sapiens GN=ALB PE=1 SV=2

Match to Query 11578: 2489.270292 from(830.764040,3+) intensity(7863925.0000) scans(14516) rtinseconds(2671) index(12130)
Title: 150825_TTSH_Patient_Plasma_20_Spectrum029121_scans__14516_RTINSECONDS=2671
Data file L:\\Ard_TTSH\\T1D\\T150825_TTSH_Patient_Plasma_20.mgf

Click mouse within plot area to zoom in by factor of two about that point
Or, [Plot from] [0] to [2400] Da [Full range]
Label all possible matches ○ Label matches used for scoring ⊙

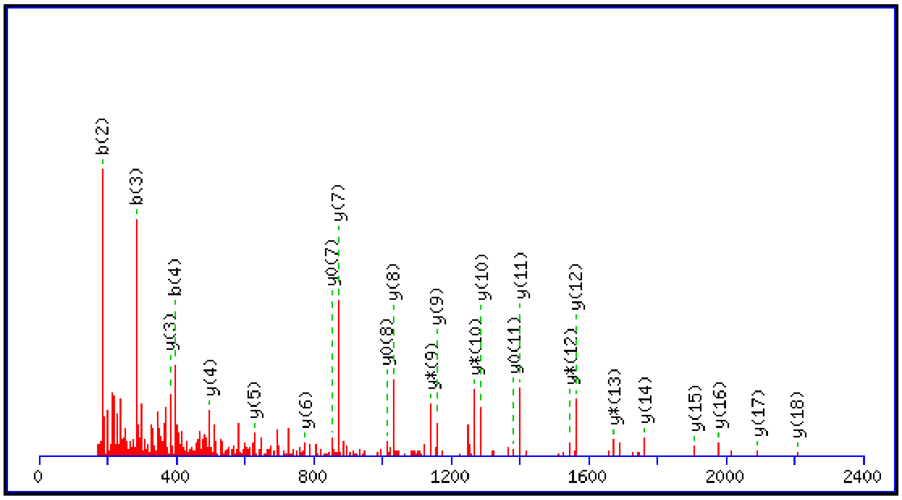


```
Monoisotopic mass of neutral peptide Mr(calc): 2489.2777
Variable modifications:
C14     : Carbamidomethyl (C)
Ions Score: 117  Expect: 6.5e-010
Matches : 25/186 fragment ions using 37 most intense peaks   (help)
```

#	b	b^{++}	b*	b*$^{++}$	b^{0}	b^{0++}	Seq.	y	y^{++}	y*	y*$^{++}$	y^{0}	y^{0++}	#
1	72.0444	36.5258					A							21
2	**185.1285**	93.0679					L	2419.2479	1210.1276	2402.2213	1201.6143	2401.2373	1201.1223	20
3	**284.1969**	142.6021					V	2306.1638	1153.5855	2289.1373	1145.0723	2288.1532	1144.5803	19
4	**397.2809**	199.1441					L	***2207.0954***	1104.0513	2190.0688	1095.5381	2189.0848	1095.0461	18
5	510.3650	255.6861					I	***2094.0113***	1047.5093	2076.9848	1038.9960	2076.0008	1038.5040	17
6	581.4021	291.2047					A	***1980.9273***	990.9673	1963.9007	982.4540	1962.9167	981.9620	16
7	728.4705	364.7389					F	***1909.8901***	955.4487	1892.8636	946.9354	1891.8796	946.4434	15
8	799.5076	400.2575					A	***1762.8217***	881.9145	1745.7952	873.4012	1744.8112	872.9092	14
9	927.5662	464.2867	910.5397	455.7735			Q	1691.7846	846.3959	1674.7581	837.8827	1673.7741	837.3907	13
10	1090.6295	545.8184	1073.6030	537.3051			Y	***1563.7260***	782.3667	1546.6995	773.8534	1545.7155	773.3614	12
11	1203.7136	602.3604	1186.6871	593.8472			L	***1400.6627***	700.8350	1383.6362	692.3217	1382.6521	691.8297	11
12	1331.7722	666.3897	1314.7456	657.8765			Q	***1287.5786***	644.2930	1270.5521	635.7797	1269.5681	635.2877	10
13	1459.8308	730.4190	1442.8042	721.9057			Q	***1159.5201***	580.2637	1142.4935	571.7504	1141.5095	571.2584	9
14	1619.8614	810.4343	1602.8349	801.9211			C	***1031.4615***	516.2344	1014.4349	507.7211	1013.4509	507.2291	8
15	1716.9142	858.9607	1699.8876	850.4475			P	***871.4308***	436.2191	854.4043	427.7058	853.4203	427.2138	7
16	1863.9826	932.4949	1846.9560	923.9817			F	***774.3781***	387.6927	757.3515	379.1794	756.3675	378.6874	6
17	1993.0252	997.0162	1975.9986	988.5030	1975.0146	988.0109	E	***627.3097***	314.1585	610.2831	305.6452	609.2991	305.1532	5
18	2108.0521	1054.5297	2091.0256	1046.0164	2090.0416	1045.5244	D	***498.2671***	249.6372	481.2405	241.1239	480.2565	240.6319	4
19	2245.1110	1123.0592	2228.0845	1114.5459	2227.1005	1114.0539	H	***383.2401***	192.1237	366.2136	183.6104			3

**20**	2344.1795	1172.5934	2327.1529	1164.0801	2326.1689	1163.5881	**V**	246.1812	123.5942	229.1547	115.0810			**2**
**21**							**K**	147.1128	74.0600	130.0863	65.5468			**1**

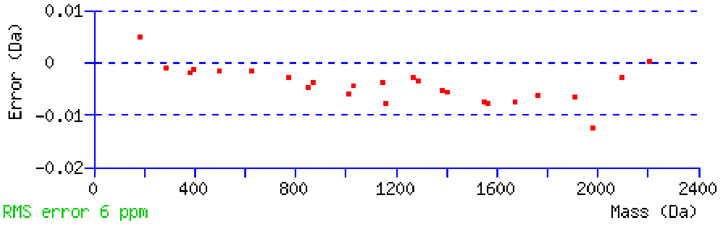

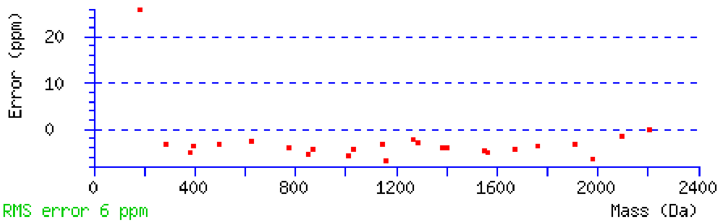

NCBI **BLAST** search of ALVLIAFAQYLQQCPFEDHVK
(Parameters: blastp, nr protein database, expect=20000, no filter, PAM30)
Other BLAST web gateways

All matches to this query

Score	Mr(calc):	Delta	Sequence
117.2	2489.2777	-0.0074	ALVLIAFAQYLQQCPFEDHVK

**Mascot:** http://www.matrixscience.com/

MATRIX SCIENCE Mascot Search Results

Peptide View

MS/MS Fragmentation of **RPCFSALEVDETYVPK**
Found in **sp|P02768|ALBU_HUMAN**, Serum albumin OS=Homo sapiens GN=ALB PE=1 SV=2

Match to Query 11558: 1900.886688 from(951.450620,2+) intensity(56712968.0000) scans(14692) rtinseconds(2639) index(12737)
Title: 150818_TTSH_Patient_Plasma_76_Spectrum029881_scans__14692_RTINSECONDS=2639
Data file L:\\Ard_TTSH\\T1D\\T150818_TTSH_Patient_Plasma_76.mgf

Click mouse within plot area to zoom in by factor of two about that point
Or, to Da
Label all possible matches ○ Label matches used for scoring ⦿

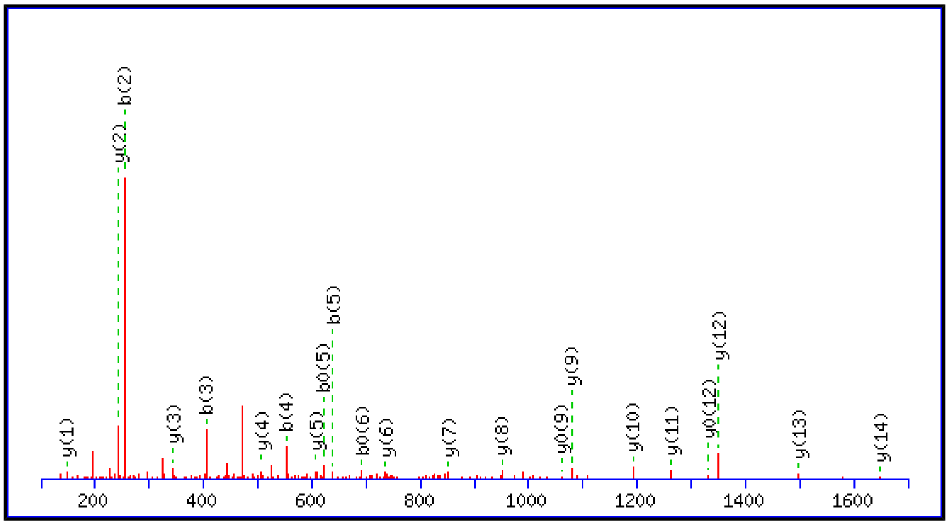

Monoisotopic mass of neutral peptide Mr(calc): 1900.8877
Variable modifications:
C3 : 4Trioxidation (CMWY)
Ions Score: 93 **Expect:** 2.1e-007
Matches : 22/164 fragment ions using 47 most intense peaks (help)

#	b	b^{++}	b*	b^{*++}	b^0	b^{0++}	Seq.	y	y^{++}	y*	y^{*++}	y^0	y^{0++}	#
1	157.1084	79.0578	140.0818	70.5446			R							16
2	**254.1612**	127.5842	237.1346	119.0709			P	1745.7938	873.4006	1728.7673	864.8873	1727.7833	864.3953	15
3	**405.1551**	203.0812	388.1285	194.5679			C	***1648.7411***	824.8742	1631.7145	816.3609	1630.7305	815.8689	14
4	**552.2235**	276.6154	535.1969	268.1021			F	***1497.7472***	749.3772	1480.7206	740.8639	1479.7366	740.3719	13
5	**639.2555**	320.1314	622.2290	311.6181	621.2450	311.1261	S	***1350.6787***	675.8430	1333.6522	667.3297	1332.6682	666.8377	12
6	710.2926	355.6500	693.2661	347.1367	692.2821	346.6447	A	***1263.6467***	632.3270	1246.6202	623.8137	1245.6361	623.3217	11
7	823.3767	412.1920	806.3502	403.6787	805.3661	403.1867	L	***1192.6096***	596.8084	1175.5830	588.2952	1174.5990	587.8032	10
8	952.4193	476.7133	935.3927	468.2000	934.4087	467.7080	E	***1079.5255***	540.2664	1062.4990	531.7531	1061.5150	531.2611	9
9	1051.4877	526.2475	1034.4612	517.7342	1033.4771	517.2422	V	***950.4829***	475.7451	933.4564	467.2318	932.4724	466.7398	8
10	1166.5147	583.7610	1149.4881	575.2477	1148.5041	574.7557	D	***851.4145***	426.2109	834.3880	417.6976	833.4040	417.2056	7
11	1295.5572	648.2823	1278.5307	639.7690	1277.5467	639.2770	E	***736.3876***	368.6974	719.3610	360.1842	718.3770	359.6921	6
12	1396.6049	698.8061	1379.5784	690.2928	1378.5944	689.8008	T	***607.3450***	304.1761	590.3184	295.6629	589.3344	295.1709	5
13	1559.6683	780.3378	1542.6417	771.8245	1541.6577	771.3325	Y	***506.2973***	253.6523	489.2708	245.1390			4
14	1658.7367	829.8720	1641.7101	821.3587	1640.7261	820.8667	V	***343.2340***	172.1206	326.2074	163.6074			3
15	1755.7894	878.3984	1738.7629	869.8851	1737.7789	869.3931	P	***244.1656***	122.5864	227.1390	114.0731			2
16							K	***147.1128***	74.0600	130.0863	65.5468			1

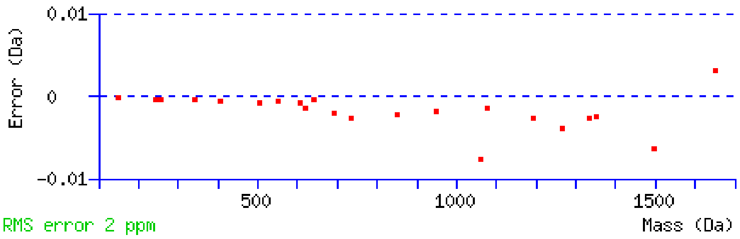

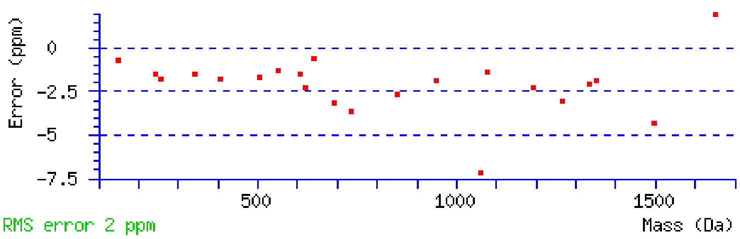

NCBI **BLAST** search of RPCFSALEVDETYVPK
(Parameters: blastp, nr protein database, expect=20000, no filter, PAM30)
Other BLAST web gateways

All matches to this query

Score	Mr(calc):	Delta	Sequence
92.5	1900.8877	-0.0010	RPCFSALEVDETYVPK
1.9	1900.8877	-0.0010	RPCFSALEVDETYVPK

**Mascot:** http://www.matrixscience.com/

Mascot Search Results

Peptide View

MS/MS Fragmentation of **RPCFSALEVDETYVPK**
Found in **sp|P02768|ALBU_HUMAN**, Serum albumin OS=Homo sapiens GN=ALB PE=1 SV=2

Match to Query 9159: 1900.886328 from(951.450440,2+) intensity(5756728.5000) scans(14772) rtinseconds(2668) index(12443)
Title: 150808_TTSH_Patient_Plasma_77_Spectrum029067_scans__14772_RTINSECONDS=2668
Data file L:\\Ard_TTSH\\T1D\\T150808_TTSH_Patient_Plasma_77.mgf

Click mouse within plot area to zoom in by factor of two about that point
Or, Plot from 100 to 1500 Da Full range
Label all possible matches ○ Label matches used for scoring ⦿

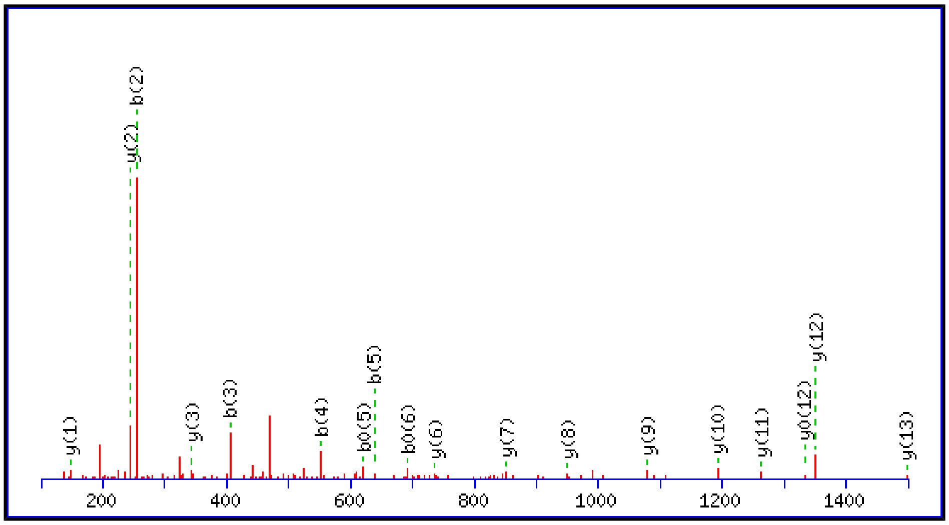

Monoisotopic mass of neutral peptide Mr(calc): 1900.8877
Variable modifications:
C3 : 4Trioxidation (CMWY)
Ions Score: 90 **Expect:** 3.7e-007
Matches : 18/164 fragment ions using 25 most intense peaks (help)

#	b	b^{++}	b*	b^{*++}	b^0	b^{0++}	Seq.	y	y^{++}	y*	y^{*++}	y^0	y^{0++}	#
1	157.1084	79.0578	140.0818	70.5446			R							16
2	254.1612	127.5842	237.1346	119.0709			P	1745.7938	873.4006	1728.7673	864.8873	1727.7833	864.3953	15
3	405.1551	203.0812	388.1285	194.5679			C	1648.7411	824.8742	1631.7145	816.3609	1630.7305	815.8689	14
4	552.2235	276.6154	535.1969	268.1021			F	1497.7472	749.3772	1480.7206	740.8639	1479.7366	740.3719	13
5	639.2555	320.1314	622.2290	311.6181	621.2450	311.1261	S	1350.6787	675.8430	1333.6522	667.3297	1332.6682	666.8377	12
6	710.2926	355.6500	693.2661	347.1367	692.2821	346.6447	A	1263.6467	632.3270	1246.6202	623.8137	1245.6361	623.3217	11
7	823.3767	412.1920	806.3502	403.6787	805.3661	403.1867	L	1192.6096	596.8084	1175.5830	588.2952	1174.5990	587.8032	10
8	952.4193	476.7133	935.3927	468.2000	934.4087	467.7080	E	1079.5255	540.2664	1062.4990	531.7531	1061.5150	531.2611	9
9	1051.4877	526.2475	1034.4612	517.7342	1033.4771	517.2422	V	950.4829	475.7451	933.4564	467.2318	932.4724	466.7398	8
10	1166.5147	583.7610	1149.4881	575.2477	1148.5041	574.7557	D	851.4145	426.2109	834.3880	417.6976	833.4040	417.2056	7
11	1295.5572	648.2823	1278.5307	639.7690	1277.5467	639.2770	E	736.3876	368.6974	719.3610	360.1842	718.3770	359.6921	6
12	1396.6049	698.8061	1379.5784	690.2928	1378.5944	689.8008	T	607.3450	304.1761	590.3184	295.6629	589.3344	295.1709	5
13	1559.6683	780.3378	1542.6417	771.8245	1541.6577	771.3325	Y	506.2973	253.6523	489.2708	245.1390			4
14	1658.7367	829.8720	1641.7101	821.3587	1640.7261	820.8667	V	343.2340	172.1206	326.2074	163.6074			3
15	1755.7894	878.3984	1738.7629	869.8851	1737.7789	869.3931	P	244.1656	122.5864	227.1390	114.0731			2
16							K	147.1128	74.0600	130.0863	65.5468			1

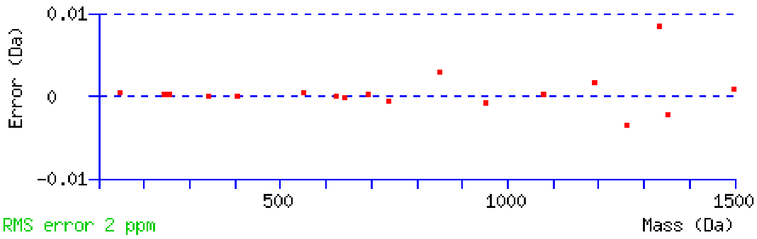

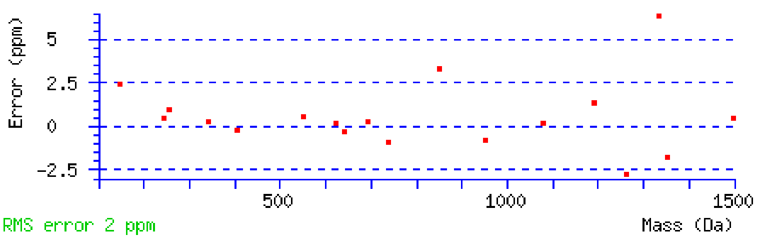

NCBI **BLAST** search of RPCFSALEVDETYVPK
(Parameters: blastp, nr protein database, expect=20000, no filter, PAM30)
Other BLAST web gateways

All matches to this query

Score	Mr(calc):	Delta	Sequence
90.1	1900.8877	-0.0013	RPCFSALEVDETYVPK
1.7	1900.8877	-0.0013	RPCFSALEVDETYVPK

**Mascot:** http://www.matrixscience.com/

Mascot Search Results

Peptide View

MS/MS Fragmentation of **RPCFSALEVDETYVPK**
Found in **sp|P02768|ALBU_HUMAN**, Serum albumin OS=Homo sapiens GN=ALB PE=1 SV=2

Match to Query 9524: 1900.885968 from(951.450260,2+) intensity(4913686.5000) scans(14768) rtinseconds(2665) index(12477)
Title: 150808_TTSH_Patient_Plasma_78_Spectrum029085_scans__14768_RTINSECONDS=2665
Data file L:\\Ard_TTSH\\T1D\\T150808_TTSH_Patient_Plasma_78.mgf

Click mouse within plot area to zoom in by factor of two about that point
Or, Plot from 100 to 1500 Da Full range
Label all possible matches ○ Label matches used for scoring ⊙

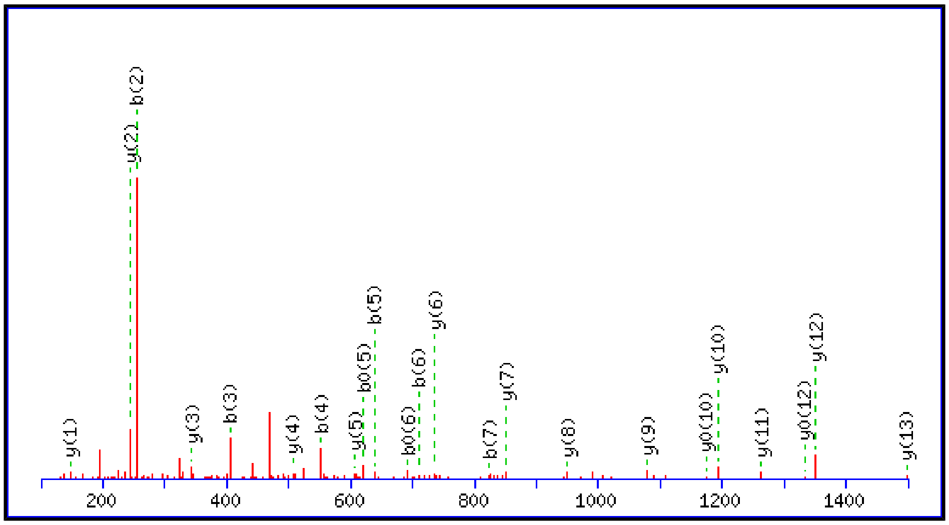

Monoisotopic mass of neutral peptide Mr(calc): 1900.8877
Variable modifications:
C3 : 4Trioxidation (CMWY)
Ions Score: 86 **Expect:** 8.7e-007
Matches : 23/164 fragment ions using 45 most intense peaks (help)

#	b	b^{++}	b*	b^{*++}	b^0	b^{0++}	Seq.	y	y^{++}	y*	y^{*++}	y^0	y^{0++}	#
1	157.1084	79.0578	140.0818	70.5446			R							16
2	**254.1612**	127.5842	237.1346	119.0709			P	1745.7938	873.4006	1728.7673	864.8873	1727.7833	864.3953	15
3	**405.1551**	203.0812	388.1285	194.5679			C	1648.7411	824.8742	1631.7145	816.3609	1630.7305	815.8689	14
4	**552.2235**	276.6154	535.1969	268.1021			F	***1497.7472***	749.3772	1480.7206	740.8639	1479.7366	740.3719	13
5	**639.2555**	320.1314	622.2290	311.6181	621.2450	311.1261	S	***1350.6787***	675.8430	1333.6522	667.3297	1332.6682	666.8377	12
6	**710.2926**	355.6500	693.2661	347.1367	692.2821	346.6447	A	***1263.6467***	632.3270	1246.6202	623.8137	1245.6361	623.3217	11
7	**823.3767**	412.1920	806.3502	403.6787	805.3661	403.1867	L	***1192.6096***	596.8084	1175.5830	588.2952	1174.5990	587.8032	10
8	952.4193	476.7133	935.3927	468.2000	934.4087	467.7080	E	***1079.5255***	540.2664	1062.4990	531.7531	1061.5150	531.2611	9
9	1051.4877	526.2475	1034.4612	517.7342	1033.4771	517.2422	V	***950.4829***	475.7451	933.4564	467.2318	932.4724	466.7398	8
10	1166.5147	583.7610	1149.4881	575.2477	1148.5041	574.7557	D	***851.4145***	426.2109	834.3880	417.6976	833.4040	417.2056	7
11	1295.5572	648.2823	1278.5307	639.7690	1277.5467	639.2770	E	***736.3876***	368.6974	719.3610	360.1842	718.3770	359.6921	6
12	1396.6049	698.8061	1379.5784	690.2928	1378.5944	689.8008	T	***607.3450***	304.1761	590.3184	295.6629	589.3344	295.1709	5
13	1559.6683	780.3378	1542.6417	771.8245	1541.6577	771.3325	Y	***506.2973***	253.6523	489.2708	245.1390			4
14	1658.7367	829.8720	1641.7101	821.3587	1640.7261	820.8667	V	***343.2340***	172.1206	326.2074	163.6074			3
15	1755.7894	878.3984	1738.7629	869.8851	1737.7789	869.3931	P	***244.1656***	122.5864	227.1390	114.0731			2
16							K	***147.1128***	74.0600	130.0863	65.5468			1

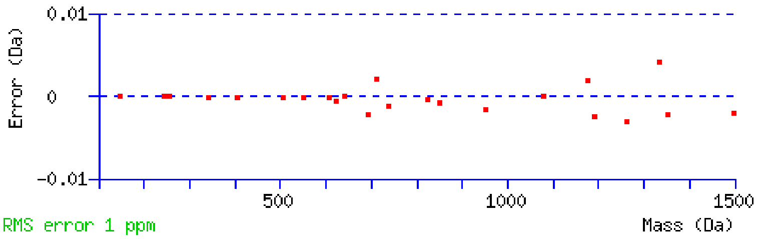

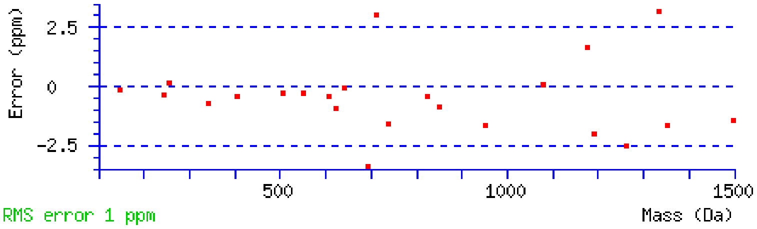

NCBI **BLAST** search of RPCFSALEVDETYVPK
(Parameters: blastp, nr protein database, expect=20000, no filter, PAM30)
Other BLAST web gateways

All matches to this query

Score	Mr(calc):	Delta	Sequence
86.4	1900.8877	-0.0017	RPCFSALEVDETYVPK
0.4	1900.8877	-0.0017	RPCFSALEVDETYVPK

Mascot Search Results

Peptide View

MS/MS Fragmentation of **RPCFSALEVDETYVPK**
Found in **sp|P02768|ALBU_HUMAN**, Serum albumin OS=Homo sapiens GN=ALB PE=1 SV=2

Match to Query 9322: 1900.884988 from(951.449770,2+) intensity(23967612.0000) scans(14646) rtinseconds(2977) index(11679)
Title: 150801_TTSH_Patient_Plasma_88_Spectrum026357_scans__14646_RTINSECONDS=2977
Data file L:\\Ard_TTSH\\T1D\\T150801_TTSH_Patient_Plasma_88.mgf

Click mouse within plot area to zoom in by factor of two about that point
Or, Plot from 100 to 1500 Da Full range
Label all possible matches ○ Label matches used for scoring ⊙

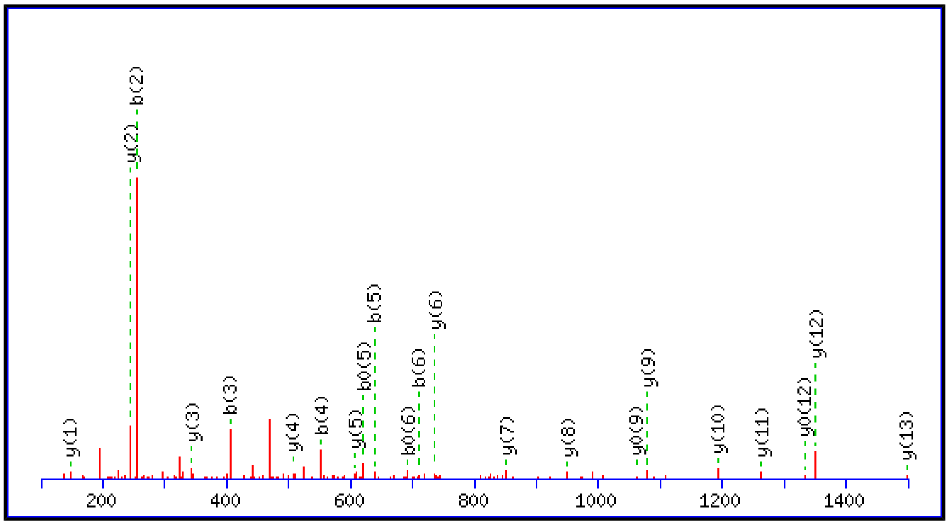

Monoisotopic mass of neutral peptide Mr(calc): 1900.8877
Variable modifications:
C3 : 4Trioxidation (CMWY)
Ions Score: 84 **Expect:** 1.3e-006
Matches : 22/164 fragment ions using 45 most intense peaks (help)

#	b	b^{++}	b*	b^{*++}	b^0	b^{0++}	Seq.	y	y^{++}	y*	y^{*++}	y^0	y^{0++}	#
1	157.1084	79.0578	140.0818	70.5446			R							16
2	**254.1612**	127.5842	237.1346	119.0709			P	1745.7938	873.4006	1728.7673	864.8873	1727.7833	864.3953	15
3	**405.1551**	203.0812	388.1285	194.5679			C	1648.7411	824.8742	1631.7145	816.3609	1630.7305	815.8689	14
4	**552.2235**	276.6154	535.1969	268.1021			F	***1497.7472***	749.3772	1480.7206	740.8639	1479.7366	740.3719	13
5	**639.2555**	320.1314	622.2290	311.6181	621.2450	311.1261	S	***1350.6787***	675.8430	1333.6522	667.3297	1332.6682	666.8377	12
6	**710.2926**	355.6500	693.2661	347.1367	692.2821	346.6447	A	***1263.6467***	632.3270	1246.6202	623.8137	1245.6361	623.3217	11
7	823.3767	412.1920	806.3502	403.6787	805.3661	403.1867	L	***1192.6096***	596.8084	1175.5830	588.2952	1174.5990	587.8032	10
8	952.4193	476.7133	935.3927	468.2000	934.4087	467.7080	E	***1079.5255***	540.2664	1062.4990	531.7531	1061.5150	531.2611	9
9	1051.4877	526.2475	1034.4612	517.7342	1033.4771	517.2422	V	***950.4829***	475.7451	933.4564	467.2318	932.4724	466.7398	8
10	1166.5147	583.7610	1149.4881	575.2477	1148.5041	574.7557	D	***851.4145***	426.2109	834.3880	417.6976	833.4040	417.2056	7
11	1295.5572	648.2823	1278.5307	639.7690	1277.5467	639.2770	E	***736.3876***	368.6974	719.3610	360.1842	718.3770	359.6921	6
12	1396.6049	698.8061	1379.5784	690.2928	1378.5944	689.8008	T	***607.3450***	304.1761	590.3184	295.6629	589.3344	295.1709	5
13	1559.6683	780.3378	1542.6417	771.8245	1541.6577	771.3325	Y	***506.2973***	253.6523	489.2708	245.1390			4
14	1658.7367	829.8720	1641.7101	821.3587	1640.7261	820.8667	V	***343.2340***	172.1206	326.2074	163.6074			3
15	1755.7894	878.3984	1738.7629	869.8851	1737.7789	869.3931	P	***244.1656***	122.5864	227.1390	114.0731			2
16							K	***147.1128***	74.0600	130.0863	65.5468			1

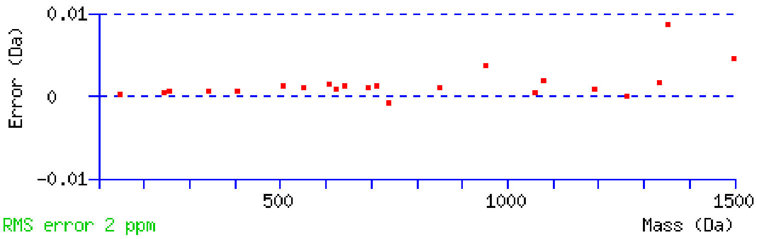

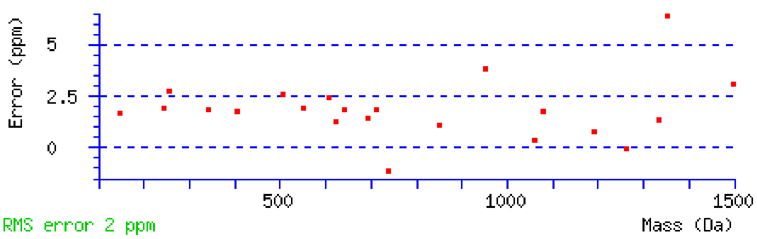

NCBI **BLAST** search of RPCFSALEVDETYVPK
(Parameters: blastp, nr protein database, expect=20000, no filter, PAM30)
Other BLAST web gateways

All matches to this query

Score	Mr(calc):	Delta	Sequence
84.4	1900.8877	-0.0027	RPCFSALEVDETYVPK
0.5	1900.8877	-0.0027	RPCFSALEVDETYVPK

Mascot: http://www.matrixscience.com/

MATRIX SCIENCE Mascot Search Results

Peptide View

MS/MS Fragmentation of **RPCFSALEVDETYVPK**
Found in **sp|P02768|ALBU_HUMAN**, Serum albumin OS=Homo sapiens GN=ALB PE=1 SV=2

Match to Query 11475: 1900.886328 from(951.450440,2+) intensity(64443140.0000) scans(14713) rtinseconds(2640) index(12761)
Title: 150818_TTSH_Patient_Plasma_77_Spectrum029898_scans__14713_RTINSECONDS=2640
Data file L:\\Ard_TTSH\\T1D\\T150818_TTSH_Patient_Plasma_77.mgf

Click mouse within plot area to zoom in by factor of two about that point
Or, [Plot from] 100 to 1500 Da [Full range]
Label all possible matches ○ Label matches used for scoring ⊙

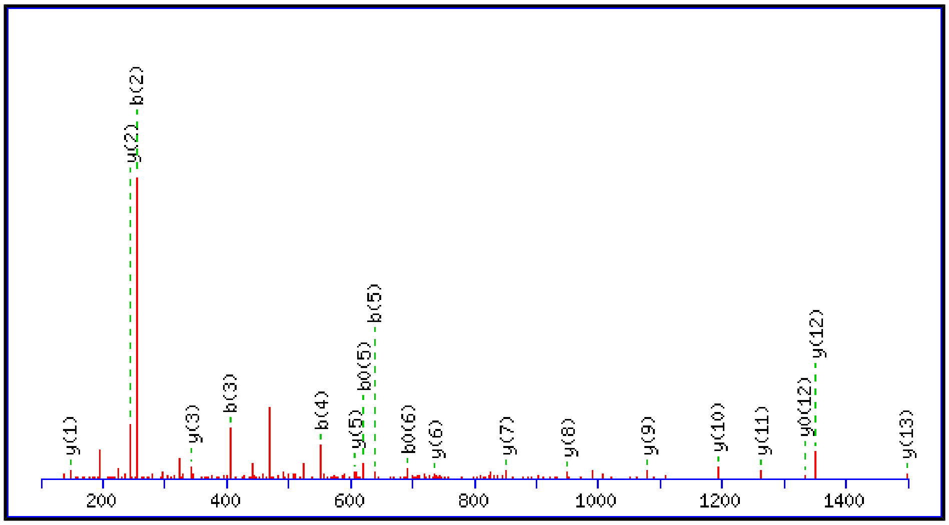

Monoisotopic mass of neutral peptide Mr(calc): 1900.8877
Variable modifications:
C3 : 4Trioxidation (CMWY)
Ions Score: 82 **Expect:** 2.2e-006
Matches : 19/164 fragment ions using 35 most intense peaks (help)

#	b	b^{++}	b^{*}	b^{*++}	b^{0}	b^{0++}	Seq.	y	y^{++}	y^{*}	y^{*++}	y^{0}	y^{0++}	#
1	157.1084	79.0578	140.0818	70.5446			R							16
2	**254.1612**	127.5842	237.1346	119.0709			P	1745.7938	873.4006	1728.7673	864.8873	1727.7833	864.3953	15
3	**405.1551**	203.0812	388.1285	194.5679			C	1648.7411	824.8742	1631.7145	816.3609	1630.7305	815.8689	14
4	**552.2235**	276.6154	535.1969	268.1021			F	***1497.7472***	749.3772	1480.7206	740.8639	1479.7366	740.3719	13
5	**639.2555**	320.1314	622.2290	311.6181	621.2450	311.1261	S	***1350.6787***	675.8430	1333.6522	667.3297	1332.6682	666.8377	12
6	710.2926	355.6500	693.2661	347.1367	692.2821	346.6447	A	***1263.6467***	632.3270	1246.6202	623.8137	1245.6361	623.3217	11
7	823.3767	412.1920	806.3502	403.6787	805.3661	403.1867	L	***1192.6096***	596.8084	1175.5830	588.2952	1174.5990	587.8032	10
8	952.4193	476.7133	935.3927	468.2000	934.4087	467.7080	E	***1079.5255***	540.2664	1062.4990	531.7531	1061.5150	531.2611	9
9	1051.4877	526.2475	1034.4612	517.7342	1033.4771	517.2422	V	***950.4829***	475.7451	933.4564	467.2318	932.4724	466.7398	8
10	1166.5147	583.7610	1149.4881	575.2477	1148.5041	574.7557	D	***851.4145***	426.2109	834.3880	417.6976	833.4040	417.2056	7
11	1295.5572	648.2823	1278.5307	639.7690	1277.5467	639.2770	E	***736.3876***	368.6974	719.3610	360.1842	718.3770	359.6921	6
12	1396.6049	698.8061	1379.5784	690.2928	1378.5944	689.8008	T	***607.3450***	304.1761	590.3184	295.6629	589.3344	295.1709	5
13	1559.6683	780.3378	1542.6417	771.8245	1541.6577	771.3325	Y	506.2973	253.6523	489.2708	245.1390			4
14	1658.7367	829.8720	1641.7101	821.3587	1640.7261	820.8667	V	***343.2340***	172.1206	326.2074	163.6074			3
15	1755.7894	878.3984	1738.7629	869.8851	1737.7789	869.3931	P	***244.1656***	122.5864	227.1390	114.0731			2
16							K	***147.1128***	74.0600	130.0863	65.5468			1

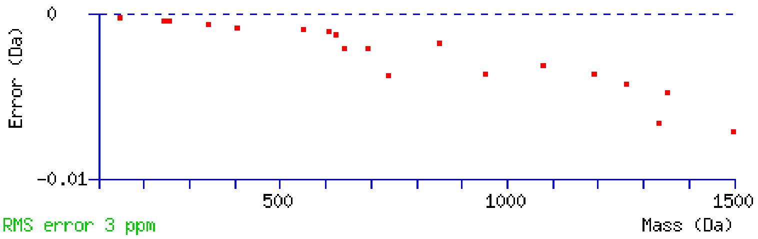

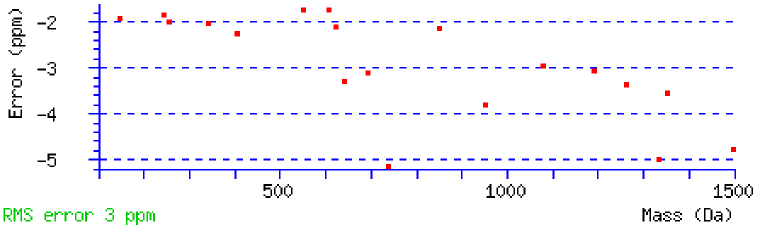

NCBI **BLAST** search of RPCFSALEVDETYVPK
(Parameters: blastp, nr protein database, expect=20000, no filter, PAM30)
Other BLAST web gateways

All matches to this query

Score	Mr(calc):	Delta	Sequence
82.4	1900.8877	-0.0013	RPCFSALEVDETYVPK
0.2	1900.8877	-0.0013	RPCFSALEVDETYVPK

Mascot: http://www.matrixscience.com/

Mascot Search Results

Peptide View

MS/MS Fragmentation of **RPCFSALEVDETYVPK**
Found in **sp|P02768|ALBU_HUMAN**, Serum albumin OS=Homo sapiens GN=ALB PE=1 SV=2

Match to Query 11826: 1900.885348 from(951.449950,2+) intensity(607340.1250) scans(15213) rtinseconds(2816) index(12787)
Title: 150808_TTSH_Patient_Plasma_24_Spectrum028563_scans__15213_RTINSECONDS=2816
Data file L:\\Ard_TTSH\\T1D\\T150808_TTSH_Patient_Plasma_24.mgf

Click mouse within plot area to zoom in by factor of two about that point
Or, Plot from 100 to 1400 Da Full range
Label all possible matches ○ Label matches used for scoring ⊙

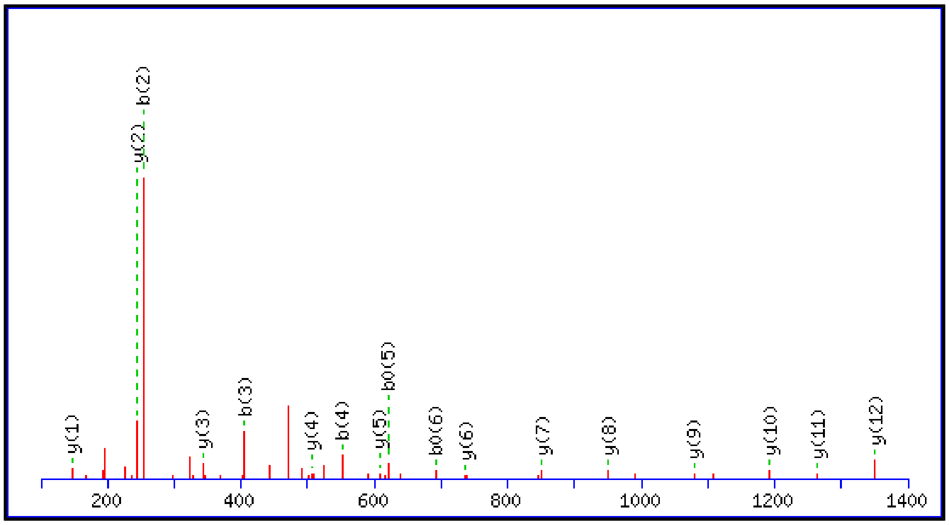

Monoisotopic mass of neutral peptide Mr(calc): 1900.8877
Variable modifications:
C3 : 4Trioxidation (CMWY)
Ions Score: 82 **Expect:** 2.6e-006
Matches : 17/164 fragment ions using 33 most intense peaks (help)

#	b	b^{++}	b*	b^{*++}	b^{0}	b^{0++}	Seq.	y	y^{++}	y*	y^{*++}	y^{0}	y^{0++}	#
1	157.1084	79.0578	140.0818	70.5446			R							16
2	**254.1612**	127.5842	237.1346	119.0709			P	1745.7938	873.4006	1728.7673	864.8873	1727.7833	864.3953	15
3	**405.1551**	203.0812	388.1285	194.5679			C	1648.7411	824.8742	1631.7145	816.3609	1630.7305	815.8689	14
4	**552.2235**	276.6154	535.1969	268.1021			F	1497.7472	749.3772	1480.7206	740.8639	1479.7366	740.3719	13
5	639.2555	320.1314	622.2290	311.6181	621.2450	311.1261	S	***1350.6787***	675.8430	1333.6522	667.3297	1332.6682	666.8377	12
6	710.2926	355.6500	693.2661	347.1367	692.2821	346.6447	A	***1263.6467***	632.3270	1246.6202	623.8137	1245.6361	623.3217	11
7	823.3767	412.1920	806.3502	403.6787	805.3661	403.1867	L	***1192.6096***	596.8084	1175.5830	588.2952	1174.5990	587.8032	10
8	952.4193	476.7133	935.3927	468.2000	934.4087	467.7080	E	***1079.5255***	540.2664	1062.4990	531.7531	1061.5150	531.2611	9
9	1051.4877	526.2475	1034.4612	517.7342	1033.4771	517.2422	V	***950.4829***	475.7451	933.4564	467.2318	932.4724	466.7398	8
10	1166.5147	583.7610	1149.4881	575.2477	1148.5041	574.7557	D	***851.4145***	426.2109	834.3880	417.6976	833.4040	417.2056	7
11	1295.5572	648.2823	1278.5307	639.7690	1277.5467	639.2770	E	***736.3876***	368.6974	719.3610	360.1842	718.3770	359.6921	6
12	1396.6049	698.8061	1379.5784	690.2928	1378.5944	689.8008	T	***607.3450***	304.1761	590.3184	295.6629	589.3344	295.1709	5
13	1559.6683	780.3378	1542.6417	771.8245	1541.6577	771.3325	Y	***506.2973***	253.6523	489.2708	245.1390			4
14	1658.7367	829.8720	1641.7101	821.3587	1640.7261	820.8667	V	***343.2340***	172.1206	326.2074	163.6074			3
15	1755.7894	878.3984	1738.7629	869.8851	1737.7789	869.3931	P	***244.1656***	122.5864	227.1390	114.0731			2
16							K	***147.1128***	74.0600	130.0863	65.5468			1

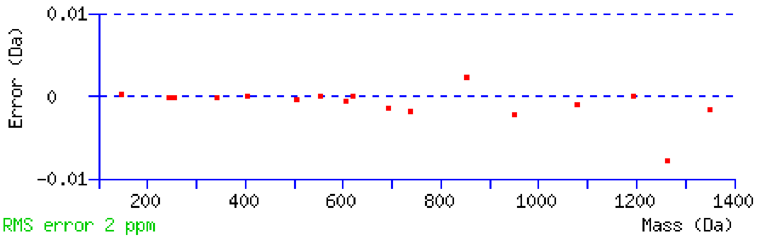

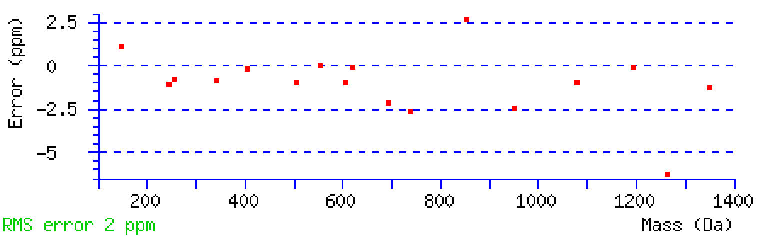

NCBI **BLAST** search of RPCFSALEVDETYVPK
(Parameters: blastp, nr protein database, expect=20000, no filter, PAM30)
Other BLAST web gateways

All matches to this query

Score	Mr(calc):	Delta	Sequence
81.6	1900.8877	-0.0023	RPCFSALEVDETYVPK
1.2	1900.8877	-0.0023	RPCFSALEVDETYVPK

Mascot: http://www.matrixscience.com/

Mascot Search Results

Peptide View

MS/MS Fragmentation of **RPCFSALEVDETYVPK**
Found in **sp|P02768|ALBU_HUMAN**, Serum albumin OS=Homo sapiens GN=ALB PE=1 SV=2

Match to Query 9672: 1900.888028 from(951.451290,2+) intensity(1073899.3750) scans(13450) rtinseconds(2427) index(11081)
Title: 150825_TTSH_Patient_Plasma_08_Spectrum028382_scans__13450_RTINSECONDS=2427
Data file L:\\Ard_TTSH\\T1D\\T150825_TTSH_Patient_Plasma_08.mgf

Click mouse within plot area to zoom in by factor of two about that point
Or, Plot from 100 to 1500 Da Full range
Label all possible matches ○ Label matches used for scoring ⊙

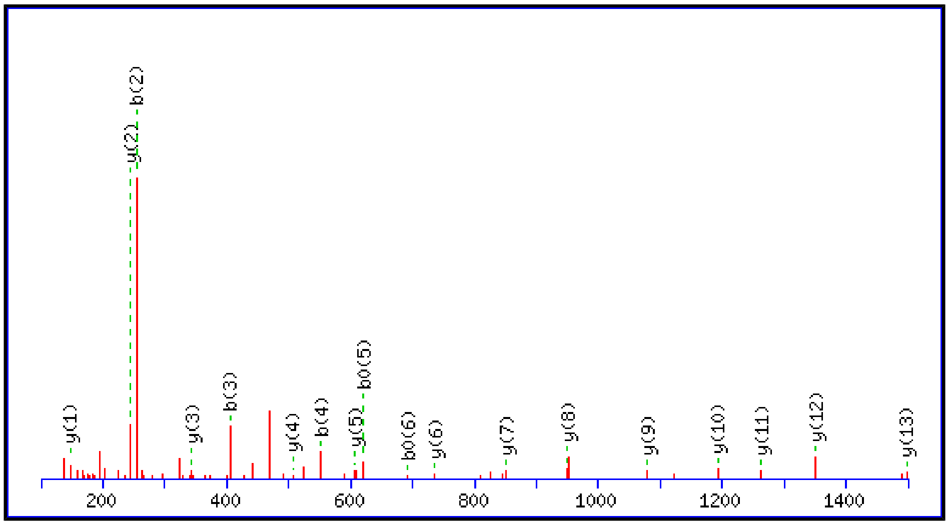

Monoisotopic mass of neutral peptide Mr(calc): 1900.8877
Variable modifications:
C3 : 4Trioxidation (CMWY)
Ions Score: 81 **Expect:** 2.9e-006
Matches : 18/164 fragment ions using 41 most intense peaks (help)

#	b	b^{++}	b*	b^{*++}	b^0	b^{0++}	Seq.	y	y^{++}	y*	y^{*++}	y^0	y^{0++}	#
1	157.1084	79.0578	140.0818	70.5446			R							16
2	**254.1612**	127.5842	237.1346	119.0709			P	1745.7938	873.4006	1728.7673	864.8873	1727.7833	864.3953	15
3	**405.1551**	203.0812	388.1285	194.5679			C	1648.7411	824.8742	1631.7145	816.3609	1630.7305	815.8689	14
4	**552.2235**	276.6154	535.1969	268.1021			F	***1497.7472***	749.3772	1480.7206	740.8639	1479.7366	740.3719	13
5	639.2555	320.1314	622.2290	311.6181	621.2450	311.1261	S	***1350.6787***	675.8430	1333.6522	667.3297	1332.6682	666.8377	12
6	710.2926	355.6500	693.2661	347.1367	692.2821	346.6447	A	***1263.6467***	632.3270	1246.6202	623.8137	1245.6361	623.3217	11
7	823.3767	412.1920	806.3502	403.6787	805.3661	403.1867	L	***1192.6096***	596.8084	1175.5830	588.2952	1174.5990	587.8032	10
8	952.4193	476.7133	935.3927	468.2000	934.4087	467.7080	E	***1079.5255***	540.2664	1062.4990	531.7531	1061.5150	531.2611	9
9	1051.4877	526.2475	1034.4612	517.7342	1033.4771	517.2422	V	***950.4829***	475.7451	933.4564	467.2318	932.4724	466.7398	8
10	1166.5147	583.7610	1149.4881	575.2477	1148.5041	574.7557	D	***851.4145***	426.2109	834.3880	417.6976	833.4040	417.2056	7
11	1295.5572	648.2823	1278.5307	639.7690	1277.5467	639.2770	E	***736.3876***	368.6974	719.3610	360.1842	718.3770	359.6921	6
12	1396.6049	698.8061	1379.5784	690.2928	1378.5944	689.8008	T	***607.3450***	304.1761	590.3184	295.6629	589.3344	295.1709	5
13	1559.6683	780.3378	1542.6417	771.8245	1541.6577	771.3325	Y	***506.2973***	253.6523	489.2708	245.1390			4
14	1658.7367	829.8720	1641.7101	821.3587	1640.7261	820.8667	V	***343.2340***	172.1206	326.2074	163.6074			3
15	1755.7894	878.3984	1738.7629	869.8851	1737.7789	869.3931	P	***244.1656***	122.5864	227.1390	114.0731			2
16							K	***147.1128***	74.0600	130.0863	65.5468			1

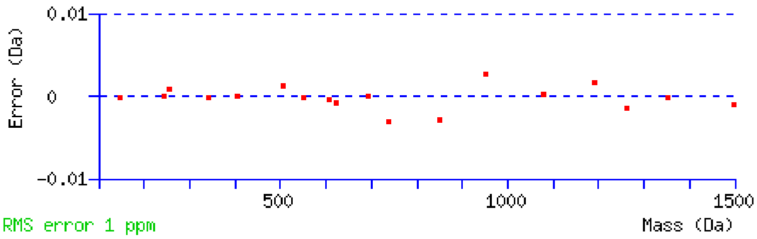

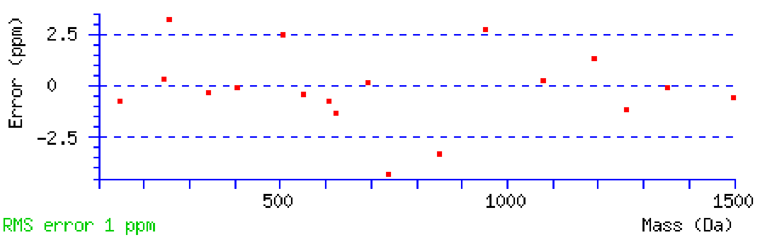

NCBI **BLAST** search of RPCFSALEVDETYVPK
(Parameters: blastp, nr protein database, expect=20000, no filter, PAM30)
Other BLAST web gateways

All matches to this query

Score	Mr(calc):	Delta	Sequence
81.3	1900.8877	0.0004	RPCFSALEVDETYVPK
0.8	1900.8877	0.0004	RPCFSALEVDETYVPK

Mascot: http://www.matrixscience.com/

Mascot Search Results

Peptide View

MS/MS Fragmentation of **RPCFSALEVDETYVPK**
Found in **sp|P02768|ALBU_HUMAN**, Serum albumin OS=Homo sapiens GN=ALB PE=1 SV=2

Match to Query 9875: 1900.887428 from(951.450990,2+) intensity(2719322.7500) scans(13940) rtinseconds(2454) index(12269)
Title: 150818_TTSH_Patient_Plasma_89_Spectrum029962_scans__13940_RTINSECONDS=2454
Data file L:\\Ard_TTSH\\T1D\\T150818_TTSH_Patient_Plasma_89.mgf

Click mouse within plot area to zoom in by factor of two about that point
Or, Plot from 100 to 1500 Da Full range
Label all possible matches ○ Label matches used for scoring ⦿

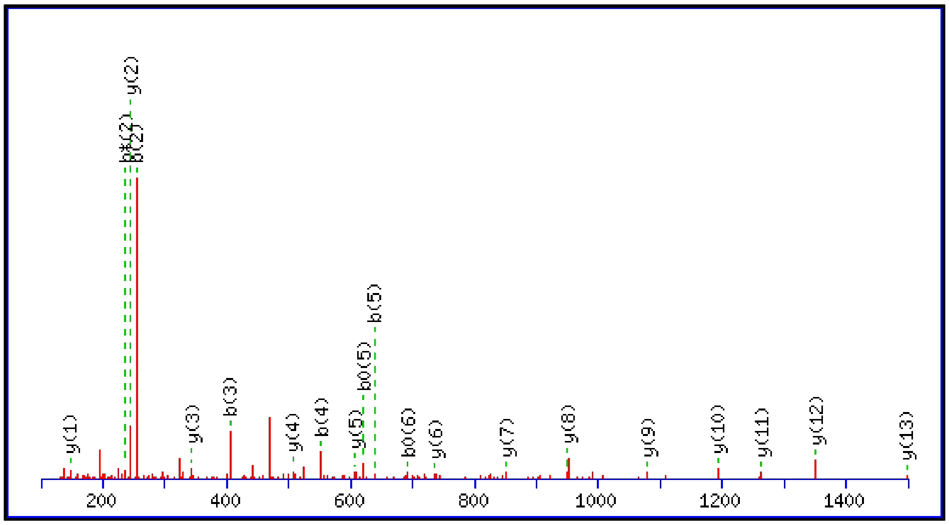

Monoisotopic mass of neutral peptide Mr(calc): 1900.8877
Variable modifications:
C3 : 4Trioxidation (CMWY)
Ions Score: 81 **Expect:** 2.8e-006
Matches : 20/164 fragment ions using 44 most intense peaks (help)

#	b	b^{++}	b*	b^{*++}	b^0	b^{0++}	Seq.	y	y^{++}	y*	y^{*++}	y^0	y^{0++}	#
1	157.1084	79.0578	140.0818	70.5446			R							16
2	**254.1612**	127.5842	237.1346	119.0709			P	1745.7938	873.4006	1728.7673	864.8873	1727.7833	864.3953	15
3	**405.1551**	203.0812	388.1285	194.5679			C	1648.7411	824.8742	1631.7145	816.3609	1630.7305	815.8689	14
4	**552.2235**	276.6154	535.1969	268.1021			F	***1497.7472***	749.3772	1480.7206	740.8639	1479.7366	740.3719	13
5	**639.2555**	320.1314	622.2290	311.6181	621.2450	311.1261	S	***1350.6787***	675.8430	1333.6522	667.3297	1332.6682	666.8377	12
6	710.2926	355.6500	693.2661	347.1367	692.2821	346.6447	A	***1263.6467***	632.3270	1246.6202	623.8137	1245.6361	623.3217	11
7	823.3767	412.1920	806.3502	403.6787	805.3661	403.1867	L	***1192.6096***	596.8084	1175.5830	588.2952	1174.5990	587.8032	10
8	952.4193	476.7133	935.3927	468.2000	934.4087	467.7080	E	***1079.5255***	540.2664	1062.4990	531.7531	1061.5150	531.2611	9
9	1051.4877	526.2475	1034.4612	517.7342	1033.4771	517.2422	V	***950.4829***	475.7451	933.4564	467.2318	932.4724	466.7398	8
10	1166.5147	583.7610	1149.4881	575.2477	1148.5041	574.7557	D	***851.4145***	426.2109	834.3880	417.6976	833.4040	417.2056	7
11	1295.5572	648.2823	1278.5307	639.7690	1277.5467	639.2770	E	***736.3876***	368.6974	719.3610	360.1842	718.3770	359.6921	6
12	1396.6049	698.8061	1379.5784	690.2928	1378.5944	689.8008	T	***607.3450***	304.1761	590.3184	295.6629	589.3344	295.1709	5
13	1559.6683	780.3378	1542.6417	771.8245	1541.6577	771.3325	Y	***506.2973***	253.6523	489.2708	245.1390			4
14	1658.7367	829.8720	1641.7101	821.3587	1640.7261	820.8667	V	***343.2340***	172.1206	326.2074	163.6074			3
15	1755.7894	878.3984	1738.7629	869.8851	1737.7789	869.3931	P	***244.1656***	122.5864	227.1390	114.0731			2
16							K	***147.1128***	74.0600	130.0863	65.5468			1

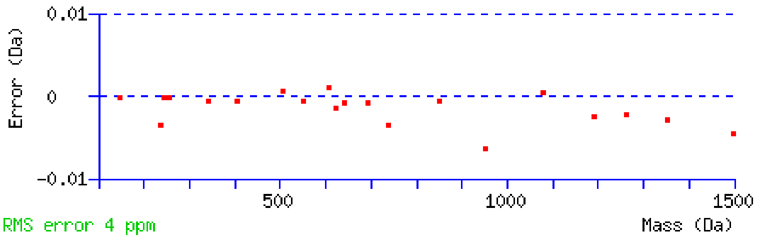

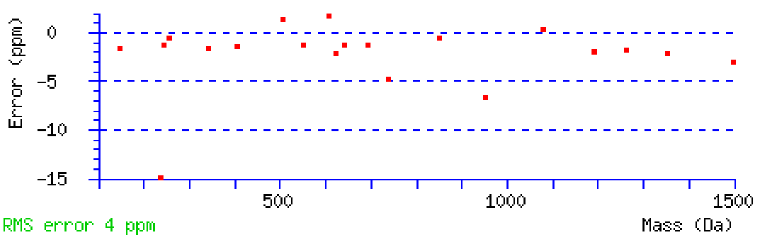

NCBI **BLAST** search of RPCFSALEVDETYVPK
(Parameters: blastp, nr protein database, expect=20000, no filter, PAM30)
Other BLAST web gateways

All matches to this query

Score	Mr(calc):	Delta	Sequence
81.3	1900.8877	-0.0002	RPCFSALEVDETYVPK

Mascot: http://www.matrixscience.com/

Mascot Search Results

Peptide View

MS/MS Fragmentation of **RPCFSALEVDETYVPK**
Found in **sp|P02768|ALBU_HUMAN**, Serum albumin OS=Homo sapiens GN=ALB PE=1 SV=2

Match to Query 9197: 1900.886808 from(951.450680,2+) intensity(25738310.0000) scans(14276) rtinseconds(2487) index(12358)
Title: 150818_TTSH_Patient_Plasma_44_Spectrum030771_scans__14276_RTINSECONDS=2487
Data file L:\\Ard_TTSH\\T1D\\T150818_TTSH_Patient_Plasma_44.mgf

Click mouse within plot area to zoom in by factor of two about that point
Or, Plot from 100 to 1600 Da Full range
Label all possible matches ○ Label matches used for scoring ⊙

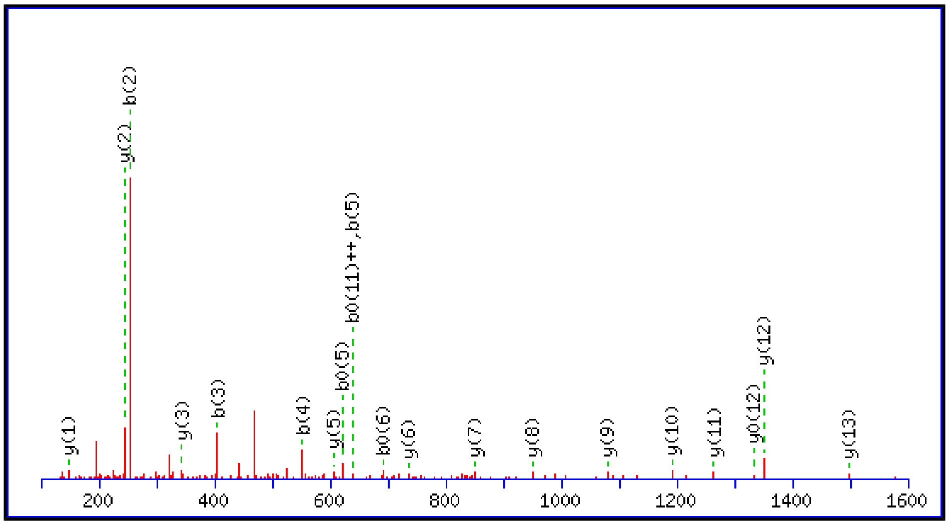

Monoisotopic mass of neutral peptide Mr(calc): 1900.8877
Variable modifications:
C3 : 4Trioxidation (CMWY)
Ions Score: 79 **Expect:** 4.9e-006
Matches : 20/164 fragment ions using 37 most intense peaks (help)

#	b	b++	b*	b*++	b0	b0++	Seq.	y	y++	y*	y*++	y0	y0++	#
1	157.1084	79.0578	140.0818	70.5446			R							16
2	**254.1612**	127.5842	237.1346	119.0709			P	1745.7938	873.4006	1728.7673	864.8873	1727.7833	864.3953	15
3	**405.1551**	203.0812	388.1285	194.5679			C	1648.7411	824.8742	1631.7145	816.3609	1630.7305	815.8689	14
4	**552.2235**	276.6154	535.1969	268.1021			F	***1497.7472***	749.3772	1480.7206	740.8639	1479.7366	740.3719	13
5	**639.2555**	320.1314	622.2290	311.6181	621.2450	311.1261	S	***1350.6787***	675.8430	1333.6522	667.3297	1332.6682	666.8377	12
6	710.2926	355.6500	693.2661	347.1367	692.2821	346.6447	A	***1263.6467***	632.3270	1246.6202	623.8137	1245.6361	623.3217	11
7	823.3767	412.1920	806.3502	403.6787	805.3661	403.1867	L	***1192.6096***	596.8084	1175.5830	588.2952	1174.5990	587.8032	10
8	952.4193	476.7133	935.3927	468.2000	934.4087	467.7080	E	***1079.5255***	540.2664	1062.4990	531.7531	1061.5150	531.2611	9
9	1051.4877	526.2475	1034.4612	517.7342	1033.4771	517.2422	V	***950.4829***	475.7451	933.4564	467.2318	932.4724	466.7398	8
10	1166.5147	583.7610	1149.4881	575.2477	1148.5041	574.7557	D	***851.4145***	426.2109	834.3880	417.6976	833.4040	417.2056	7
11	1295.5572	648.2823	1278.5307	639.7690	1277.5467	639.2770	E	***736.3876***	368.6974	719.3610	360.1842	718.3770	359.6921	6
12	1396.6049	698.8061	1379.5784	690.2928	1378.5944	689.8008	T	***607.3450***	304.1761	590.3184	295.6629	589.3344	295.1709	5
13	1559.6683	780.3378	1542.6417	771.8245	1541.6577	771.3325	Y	506.2973	253.6523	489.2708	245.1390			4
14	1658.7367	829.8720	1641.7101	821.3587	1640.7261	820.8667	V	***343.2340***	172.1206	326.2074	163.6074			3
15	1755.7894	878.3984	1738.7629	869.8851	1737.7789	869.3931	P	***244.1656***	122.5864	227.1390	114.0731			2
16							K	***147.1128***	74.0600	130.0863	65.5468			1

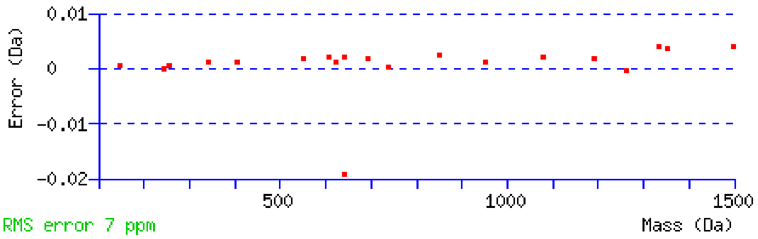

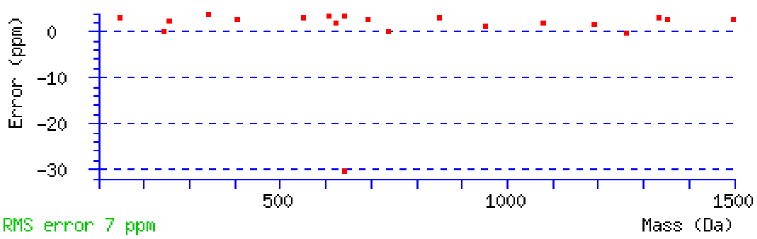

NCBI **BLAST** search of RPCFSALEVDETYVPK
(Parameters: blastp, nr protein database, expect=20000, no filter, PAM30)
Other BLAST web gateways

All matches to this query

Score	Mr(calc):	Delta	Sequence
78.9	1900.8877	-0.0009	RPCFSALEVDETYVPK
0.2	1900.8877	-0.0009	RPCFSALEVDETYVPK

**Mascot:** http://www.matrixscience.com/

Mascot Search Results

Peptide View

MS/MS Fragmentation of **RPCFSALEVDETYVPK**
Found in **sp|P02768|ALBU_HUMAN**, Serum albumin OS=Homo sapiens GN=ALB PE=1 SV=2

Match to Query 9132: 1900.885588 from(951.450070,2+) intensity(28522432.0000) scans(13252) rtinseconds(2737) index(10250)
Title: 150808_TTSH_Patient_Plasma_21_Spectrum024115_scans__13252_RTINSECONDS=2737
Data file L:\\Ard_TTSH\\T1D\\T150808_TTSH_Patient_Plasma_21.mgf

Click mouse within plot area to zoom in by factor of two about that point
Or, Plot from 100 to 1900 Da Full range
Label all possible matches ○ Label matches used for scoring ⊙

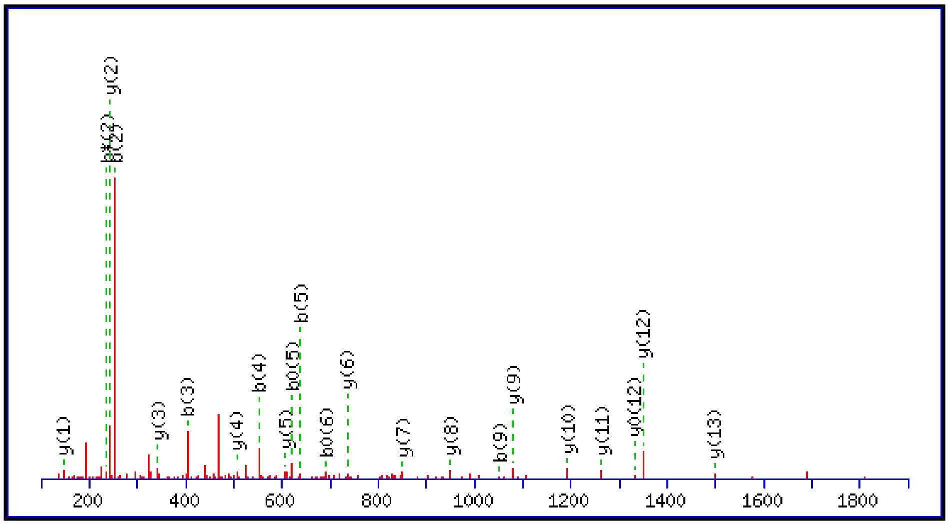

Monoisotopic mass of neutral peptide Mr(calc): 1900.8877
Variable modifications:
C3 : 4Trioxidation (CMWY)
Ions Score: 79 **Expect:** 5e-006
Matches : 22/164 fragment ions using 49 most intense peaks (help)

#	b	b++	b*	b*++	b0	b0++	Seq.	y	y++	y*	y*++	y0	y0++	#
1	157.1084	79.0578	140.0818	70.5446			R							16
2	**254.1612**	127.5842	237.1346	119.0709			P	1745.7938	873.4006	1728.7673	864.8873	1727.7833	864.3953	15
3	**405.1551**	203.0812	388.1285	194.5679			C	1648.7411	824.8742	1631.7145	816.3609	1630.7305	815.8689	14
4	**552.2235**	276.6154	535.1969	268.1021			F	***1497.7472***	749.3772	1480.7206	740.8639	1479.7366	740.3719	13
5	**639.2555**	320.1314	622.2290	311.6181	621.2450	311.1261	S	***1350.6787***	675.8430	1333.6522	667.3297	1332.6682	666.8377	12
6	710.2926	355.6500	693.2661	347.1367	692.2821	346.6447	A	***1263.6467***	632.3270	1246.6202	623.8137	1245.6361	623.3217	11
7	823.3767	412.1920	806.3502	403.6787	805.3661	403.1867	L	***1192.6096***	596.8084	1175.5830	588.2952	1174.5990	587.8032	10
8	952.4193	476.7133	935.3927	468.2000	934.4087	467.7080	E	***1079.5255***	540.2664	1062.4990	531.7531	1061.5150	531.2611	9
9	**1051.4877**	526.2475	1034.4612	517.7342	1033.4771	517.2422	V	***950.4829***	475.7451	933.4564	467.2318	932.4724	466.7398	8
10	1166.5147	583.7610	1149.4881	575.2477	1148.5041	574.7557	D	***851.4145***	426.2109	834.3880	417.6976	833.4040	417.2056	7
11	1295.5572	648.2823	1278.5307	639.7690	1277.5467	639.2770	E	***736.3876***	368.6974	719.3610	360.1842	718.3770	359.6921	6
12	1396.6049	698.8061	1379.5784	690.2928	1378.5944	689.8008	T	***607.3450***	304.1761	590.3184	295.6629	589.3344	295.1709	5
13	1559.6683	780.3378	1542.6417	771.8245	1541.6577	771.3325	Y	***506.2973***	253.6523	489.2708	245.1390			4
14	1658.7367	829.8720	1641.7101	821.3587	1640.7261	820.8667	V	***343.2340***	172.1206	326.2074	163.6074			3
15	1755.7894	878.3984	1738.7629	869.8851	1737.7789	869.3931	P	***244.1656***	122.5864	227.1390	114.0731			2
16							K	***147.1128***	74.0600	130.0863	65.5468			1

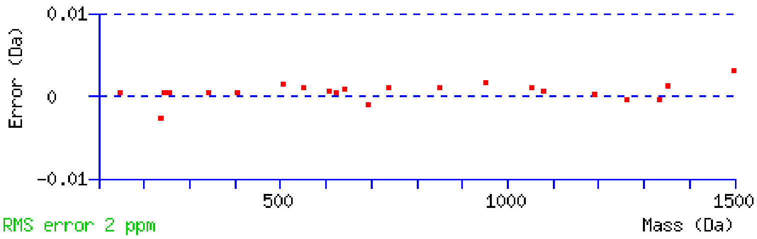

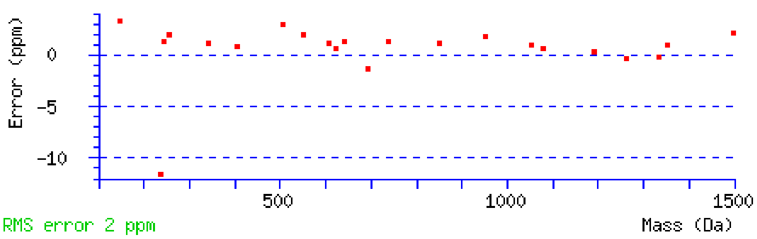

NCBI **BLAST** search of RPCFSALEVDETYVPK
(Parameters: blastp, nr protein database, expect=20000, no filter, PAM30)
Other BLAST web gateways

All matches to this query

Score	Mr(calc):	Delta	Sequence
78.8	1900.8877	-0.0021	RPCFSALEVDETYVPK

Mascot Search Results

Peptide View

MS/MS Fragmentation of **RPCFSALEVDETYVPK**
Found in **sp|P02768|ALBU_HUMAN**, Serum albumin OS=Homo sapiens GN=ALB PE=1 SV=2

Match to Query 8958: 1900.884608 from(951.449580,2+) intensity(4951795.5000) scans(14516) rtinseconds(3008) index(11332)
Title: 150801_TTSH_Patient_Plasma_86_Spectrum025554_scans__14516_RTINSECONDS=3008
Data file L:\\Ard_TTSH\\T1D\\T150801_TTSH_Patient_Plasma_86.mgf

Click mouse within plot area to zoom in by factor of two about that point
Or, Plot from 100 to 1900 Da Full range
Label all possible matches ○ Label matches used for scoring ⊙

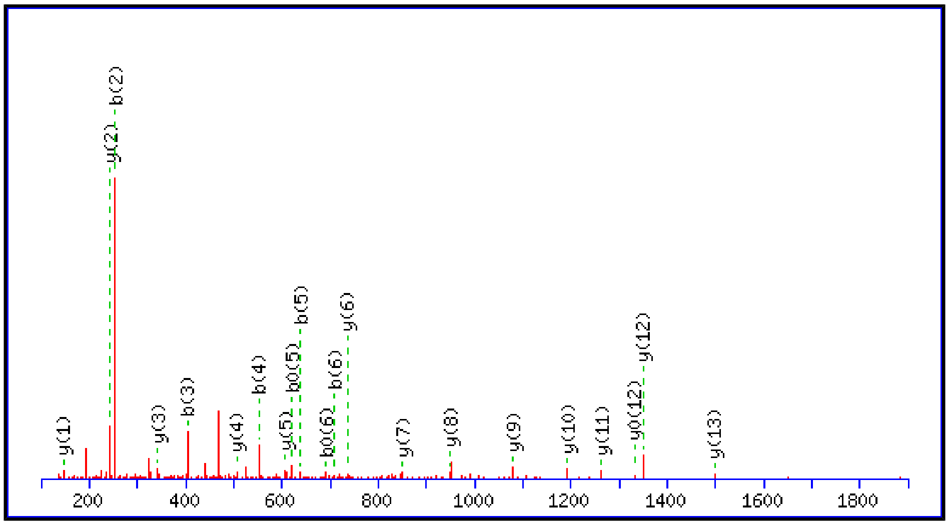

Monoisotopic mass of neutral peptide Mr(calc): 1900.8877
Variable modifications:
C3 : 4Trioxidation (CMWY)
Ions Score: 78 **Expect:** 6.5e-006
Matches : 21/164 fragment ions using 50 most intense peaks (help)

#	b	b^{++}	b*	b^{*++}	b^{0}	b^{0++}	Seq.	y	y^{++}	y*	y^{*++}	y^{0}	y^{0++}	#
1	157.1084	79.0578	140.0818	70.5446			R							16
2	**254.1612**	127.5842	237.1346	119.0709			P	1745.7938	873.4006	1728.7673	864.8873	1727.7833	864.3953	15
3	**405.1551**	203.0812	388.1285	194.5679			C	1648.7411	824.8742	1631.7145	816.3609	1630.7305	815.8689	14
4	**552.2235**	276.6154	535.1969	268.1021			F	***1497.7472***	749.3772	1480.7206	740.8639	1479.7366	740.3719	13
5	**639.2555**	320.1314	622.2290	311.6181	621.2450	311.1261	S	***1350.6787***	675.8430	1333.6522	667.3297	1332.6682	666.8377	12
6	**710.2926**	355.6500	693.2661	347.1367	692.2821	346.6447	A	***1263.6467***	632.3270	1246.6202	623.8137	1245.6361	623.3217	11
7	823.3767	412.1920	806.3502	403.6787	805.3661	403.1867	L	***1192.6096***	596.8084	1175.5830	588.2952	1174.5990	587.8032	10
8	952.4193	476.7133	935.3927	468.2000	934.4087	467.7080	E	***1079.5255***	540.2664	1062.4990	531.7531	1061.5150	531.2611	9
9	1051.4877	526.2475	1034.4612	517.7342	1033.4771	517.2422	V	***950.4829***	475.7451	933.4564	467.2318	932.4724	466.7398	8
10	1166.5147	583.7610	1149.4881	575.2477	1148.5041	574.7557	D	***851.4145***	426.2109	834.3880	417.6976	833.4040	417.2056	7
11	1295.5572	648.2823	1278.5307	639.7690	1277.5467	639.2770	E	***736.3876***	368.6974	719.3610	360.1842	718.3770	359.6921	6
12	1396.6049	698.8061	1379.5784	690.2928	1378.5944	689.8008	T	***607.3450***	304.1761	590.3184	295.6629	589.3344	295.1709	5
13	1559.6683	780.3378	1542.6417	771.8245	1541.6577	771.3325	Y	***506.2973***	253.6523	489.2708	245.1390			4
14	1658.7367	829.8720	1641.7101	821.3587	1640.7261	820.8667	V	***343.2340***	172.1206	326.2074	163.6074			3
15	1755.7894	878.3984	1738.7629	869.8851	1737.7789	869.3931	P	***244.1656***	122.5864	227.1390	114.0731			2
16							K	***147.1128***	74.0600	130.0863	65.5468			1

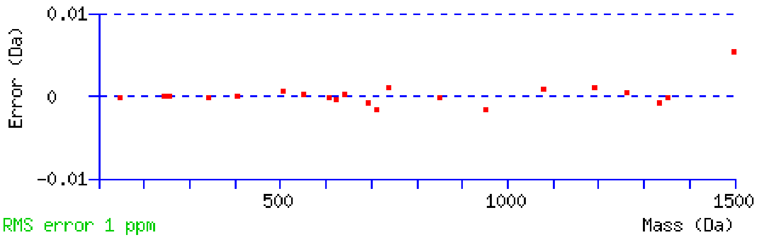

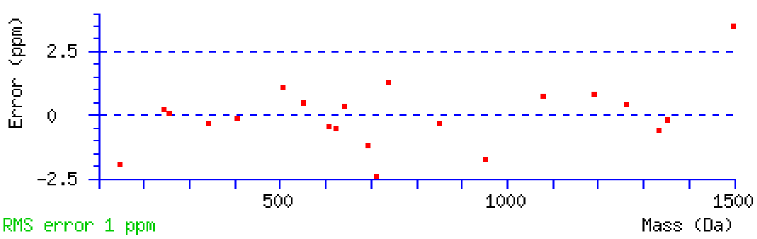

NCBI **BLAST** search of RPCFSALEVDETYVPK
(Parameters: blastp, nr protein database, expect=20000, no filter, PAM30)
Other BLAST web gateways

All matches to this query

Score	Mr(calc):	Delta	Sequence
77.6	1900.8877	-0.0031	RPCFSALEVDETYVPK
0.1	1900.8877	-0.0031	RPCFSALEVDETYVPK

Mascot Search Results

Peptide View

MS/MS Fragmentation of **RPCFSALEVDETYVPK**
Found in **sp|P02768|ALBU_HUMAN**, Serum albumin OS=Homo sapiens GN=ALB PE=1 SV=2

Match to Query 9184: 1900.886208 from(951.450380,2+) intensity(96305720.0000) scans(14260) rtinseconds(2487) index(12376)
Title: 150818_TTSH_Patient_Plasma_45_Spectrum030808_scans__14260_RTINSECONDS=2487
Data file L:\\Ard_TTSH\\T1D\\T150818_TTSH_Patient_Plasma_45.mgf

Click mouse within plot area to zoom in by factor of two about that point
Or, Plot from 100 to 1600 Da Full range
Label all possible matches ○ Label matches used for scoring ⊙

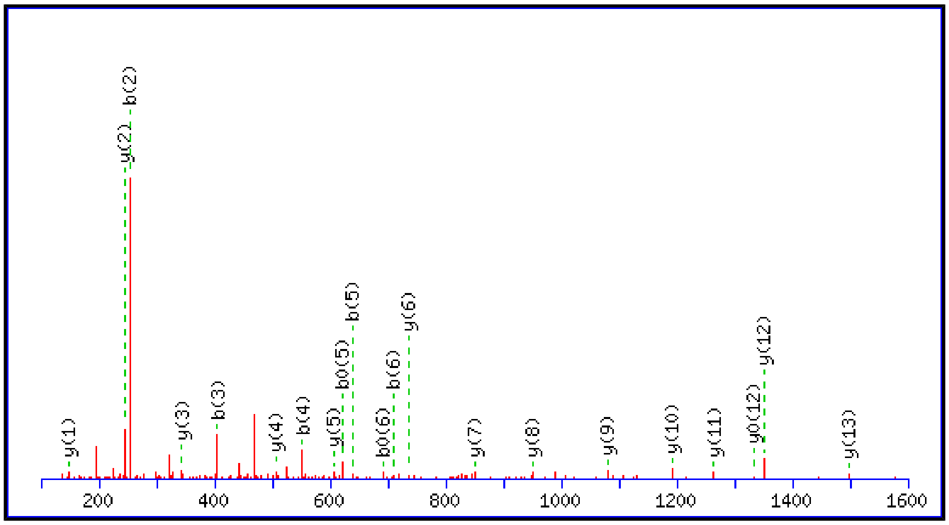

Monoisotopic mass of neutral peptide Mr(calc): 1900.8877
Variable modifications:
C3 : 4Trioxidation (CMWY)
Ions Score: 77 **Expect:** 8.1e-006
Matches : 21/164 fragment ions using 49 most intense peaks (help)

#	b	b^{++}	b*	b^{*++}	b^0	b^{0++}	Seq.	y	y^{++}	y*	y^{*++}	y^0	y^{0++}	#
1	157.1084	79.0578	140.0818	70.5446			R							16
2	**254.1612**	127.5842	237.1346	119.0709			P	1745.7938	873.4006	1728.7673	864.8873	1727.7833	864.3953	15
3	**405.1551**	203.0812	388.1285	194.5679			C	1648.7411	824.8742	1631.7145	816.3609	1630.7305	815.8689	14
4	**552.2235**	276.6154	535.1969	268.1021			F	***1497.7472***	749.3772	1480.7206	740.8639	1479.7366	740.3719	13
5	**639.2555**	320.1314	622.2290	311.6181	621.2450	311.1261	S	***1350.6787***	675.8430	1333.6522	667.3297	1332.6682	666.8377	12
6	**710.2926**	355.6500	693.2661	347.1367	692.2821	346.6447	A	***1263.6467***	632.3270	1246.6202	623.8137	1245.6361	623.3217	11
7	823.3767	412.1920	806.3502	403.6787	805.3661	403.1867	L	***1192.6096***	596.8084	1175.5830	588.2952	1174.5990	587.8032	10
8	952.4193	476.7133	935.3927	468.2000	934.4087	467.7080	E	***1079.5255***	540.2664	1062.4990	531.7531	1061.5150	531.2611	9
9	1051.4877	526.2475	1034.4612	517.7342	1033.4771	517.2422	V	***950.4829***	475.7451	933.4564	467.2318	932.4724	466.7398	8
10	1166.5147	583.7610	1149.4881	575.2477	1148.5041	574.7557	D	***851.4145***	426.2109	834.3880	417.6976	833.4040	417.2056	7
11	1295.5572	648.2823	1278.5307	639.7690	1277.5467	639.2770	E	***736.3876***	368.6974	719.3610	360.1842	718.3770	359.6921	6
12	1396.6049	698.8061	1379.5784	690.2928	1378.5944	689.8008	T	***607.3450***	304.1761	590.3184	295.6629	589.3344	295.1709	5
13	1559.6683	780.3378	1542.6417	771.8245	1541.6577	771.3325	Y	***506.2973***	253.6523	489.2708	245.1390			4
14	1658.7367	829.8720	1641.7101	821.3587	1640.7261	820.8667	V	***343.2340***	172.1206	326.2074	163.6074			3
15	1755.7894	878.3984	1738.7629	869.8851	1737.7789	869.3931	P	***244.1656***	122.5864	227.1390	114.0731			2
16							K	***147.1128***	74.0600	130.0863	65.5468			1

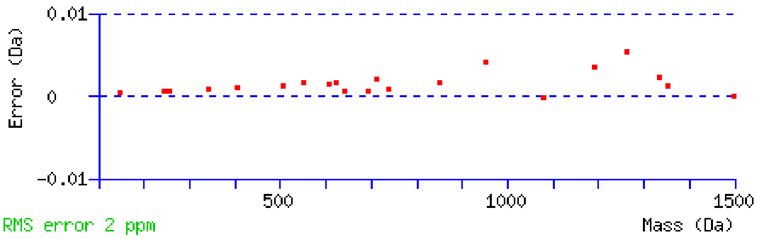

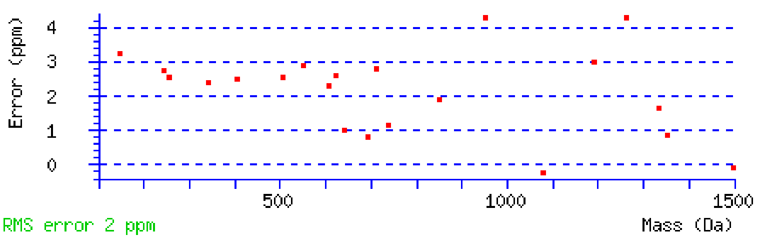

NCBI **BLAST** search of RPCFSALEVDETYVPK
(Parameters: blastp, nr protein database, expect=20000, no filter, PAM30)
Other BLAST web gateways

All matches to this query

Score	Mr(calc):	Delta	Sequence
76.7	1900.8877	-0.0015	RPCFSALEVDETYVPK

**Mascot:** http://www.matrixscience.com/

Mascot Search Results

Peptide View

MS/MS Fragmentation of **RPCFSALEVDETYVPK**
Found in **sp|P02768|ALBU_HUMAN**, Serum albumin OS=Homo sapiens GN=ALB PE=1 SV=2

Match to Query 10135: 1900.885968 from(951.450260,2+) intensity(4437039.0000) scans(15051) rtinseconds(2751) index(12709)
Title: 150808_TTSH_Patient_Plasma_48_Spectrum029275_scans__15051_RTINSECONDS=2751
Data file L:\\Ard_TTSH\\T1D\\T150808_TTSH_Patient_Plasma_48.mgf

Click mouse within plot area to zoom in by factor of two about that point
Or, [Plot from] [100] to [1700] Da [Full range]
Label all possible matches ○ Label matches used for scoring ⊙

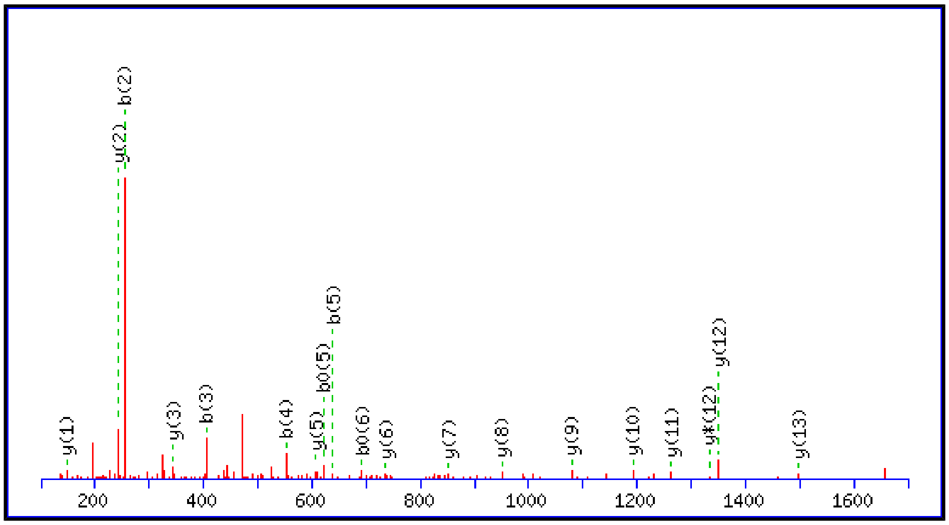

Monoisotopic mass of neutral peptide Mr(calc): 1900.8877
Variable modifications:
C3 : 4Trioxidation (CMWY)
Ions Score: 76 **Expect:** 1e-005
Matches : 19/164 fragment ions using 39 most intense peaks (help)

#	b	b++	b*	b*++	b0	b0++	Seq.	y	y++	y*	y*++	y0	y0++	#
1	157.1084	79.0578	140.0818	70.5446			R							16
2	**254.1612**	127.5842	237.1346	119.0709			P	1745.7938	873.4006	1728.7673	864.8873	1727.7833	864.3953	15
3	**405.1551**	203.0812	388.1285	194.5679			C	1648.7411	824.8742	1631.7145	816.3609	1630.7305	815.8689	14
4	**552.2235**	276.6154	535.1969	268.1021			F	***1497.7472***	749.3772	1480.7206	740.8639	1479.7366	740.3719	13
5	**639.2555**	320.1314	622.2290	311.6181	621.2450	311.1261	S	***1350.6787***	675.8430	1333.6522	667.3297	1332.6682	666.8377	12
6	710.2926	355.6500	693.2661	347.1367	692.2821	346.6447	A	***1263.6467***	632.3270	1246.6202	623.8137	1245.6361	623.3217	11
7	823.3767	412.1920	806.3502	403.6787	805.3661	403.1867	L	***1192.6096***	596.8084	1175.5830	588.2952	1174.5990	587.8032	10
8	952.4193	476.7133	935.3927	468.2000	934.4087	467.7080	E	***1079.5255***	540.2664	1062.4990	531.7531	1061.5150	531.2611	9
9	1051.4877	526.2475	1034.4612	517.7342	1033.4771	517.2422	V	***950.4829***	475.7451	933.4564	467.2318	932.4724	466.7398	8
10	1166.5147	583.7610	1149.4881	575.2477	1148.5041	574.7557	D	***851.4145***	426.2109	834.3880	417.6976	833.4040	417.2056	7
11	1295.5572	648.2823	1278.5307	639.7690	1277.5467	639.2770	E	***736.3876***	368.6974	719.3610	360.1842	718.3770	359.6921	6
12	1396.6049	698.8061	1379.5784	690.2928	1378.5944	689.8008	T	***607.3450***	304.1761	590.3184	295.6629	589.3344	295.1709	5
13	1559.6683	780.3378	1542.6417	771.8245	1541.6577	771.3325	Y	506.2973	253.6523	489.2708	245.1390			4
14	1658.7367	829.8720	1641.7101	821.3587	1640.7261	820.8667	V	***343.2340***	172.1206	326.2074	163.6074			3
15	1755.7894	878.3984	1738.7629	869.8851	1737.7789	869.3931	P	***244.1656***	122.5864	227.1390	114.0731			2
16							K	***147.1128***	74.0600	130.0863	65.5468			1

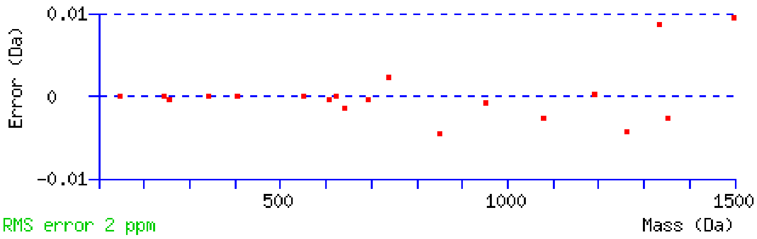

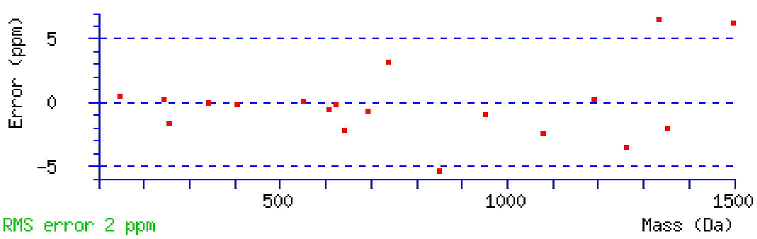

NCBI **BLAST** search of RPCFSALEVDETYVPK
(Parameters: blastp, nr protein database, expect=20000, no filter, PAM30)
Other BLAST web gateways

All matches to this query

Score	Mr(calc):	Delta	Sequence
75.7	1900.8877	-0.0017	RPCFSALEVDETYVPK
0.0	1900.8877	-0.0017	RPCFSALEVDETYVPK

**Mascot:** http://www.matrixscience.com/

Mascot Search Results

Peptide View

MS/MS Fragmentation of **RPCFSALEVDETYVPK**
Found in **sp|P02768|ALBU_HUMAN**, Serum albumin OS=Homo sapiens GN=ALB PE=1 SV=2

Match to Query 11362: 1900.886208 from(951.450380,2+) intensity(6710048.5000) scans(14770) rtinseconds(2662) index(12785)
Title: 150818_TTSH_Patient_Plasma_78_Spectrum029865_scans__14770_RTINSECONDS=2662
Data file L:\\Ard_TTSH\\T1D\\T150818_TTSH_Patient_Plasma_78.mgf

Click mouse within plot area to zoom in by factor of two about that point
Or, Plot from 100 to 1500 Da Full range
Label all possible matches ○ Label matches used for scoring ⊙

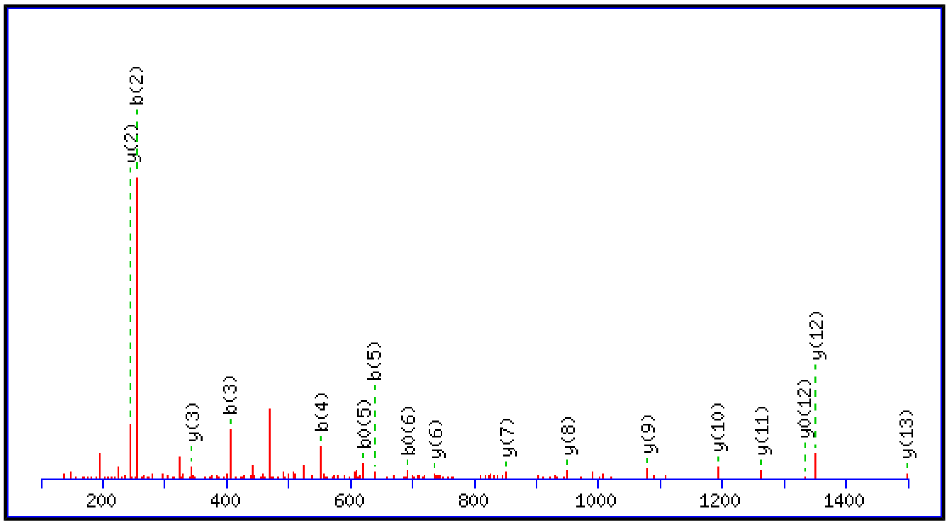

Monoisotopic mass of neutral peptide Mr(calc): 1900.8877
Variable modifications:
C3 : 4Trioxidation (CMWY)
Ions Score: 75 **Expect:** 1.1e-005
Matches : 17/164 fragment ions using 25 most intense peaks (help)

#	b	b^{++}	b*	b^{*++}	b^0	b^{0++}	Seq.	y	y^{++}	y*	y^{*++}	y^0	y^{0++}	#
1	157.1084	79.0578	140.0818	70.5446			R							16
2	**254.1612**	127.5842	237.1346	119.0709			P	1745.7938	873.4006	1728.7673	864.8873	1727.7833	864.3953	15
3	**405.1551**	203.0812	388.1285	194.5679			C	1648.7411	824.8742	1631.7145	816.3609	1630.7305	815.8689	14
4	**552.2235**	276.6154	535.1969	268.1021			F	***1497.7472***	749.3772	1480.7206	740.8639	1479.7366	740.3719	13
5	**639.2555**	320.1314	622.2290	311.6181	621.2450	311.1261	S	***1350.6787***	675.8430	1333.6522	667.3297	1332.6682	666.8377	12
6	710.2926	355.6500	693.2661	347.1367	692.2821	346.6447	A	***1263.6467***	632.3270	1246.6202	623.8137	1245.6361	623.3217	11
7	823.3767	412.1920	806.3502	403.6787	805.3661	403.1867	L	***1192.6096***	596.8084	1175.5830	588.2952	1174.5990	587.8032	10
8	952.4193	476.7133	935.3927	468.2000	934.4087	467.7080	E	***1079.5255***	540.2664	1062.4990	531.7531	1061.5150	531.2611	9
9	1051.4877	526.2475	1034.4612	517.7342	1033.4771	517.2422	V	***950.4829***	475.7451	933.4564	467.2318	932.4724	466.7398	8
10	1166.5147	583.7610	1149.4881	575.2477	1148.5041	574.7557	D	***851.4145***	426.2109	834.3880	417.6976	833.4040	417.2056	7
11	1295.5572	648.2823	1278.5307	639.7690	1277.5467	639.2770	E	***736.3876***	368.6974	719.3610	360.1842	718.3770	359.6921	6
12	1396.6049	698.8061	1379.5784	690.2928	1378.5944	689.8008	T	607.3450	304.1761	590.3184	295.6629	589.3344	295.1709	5
13	1559.6683	780.3378	1542.6417	771.8245	1541.6577	771.3325	Y	506.2973	253.6523	489.2708	245.1390			4
14	1658.7367	829.8720	1641.7101	821.3587	1640.7261	820.8667	V	***343.2340***	172.1206	326.2074	163.6074			3
15	1755.7894	878.3984	1738.7629	869.8851	1737.7789	869.3931	P	***244.1656***	122.5864	227.1390	114.0731			2
16							K	147.1128	74.0600	130.0863	65.5468			1

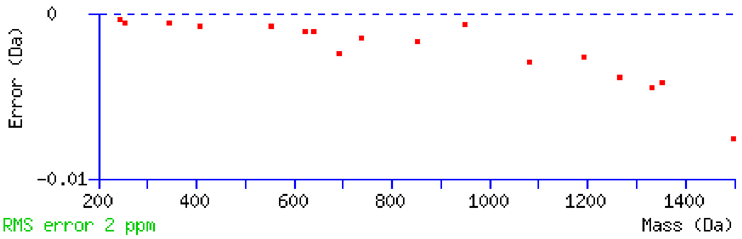

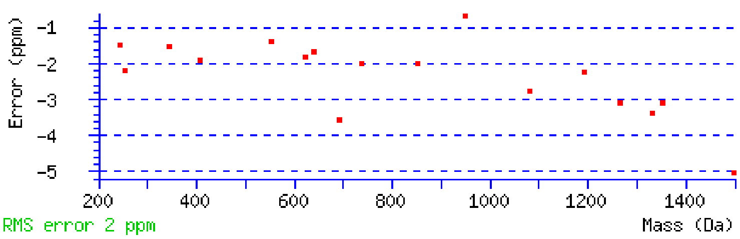

NCBI **BLAST** search of RPCFSALEVDETYVPK
(Parameters: blastp, nr protein database, expect=20000, no filter, PAM30)
Other BLAST web gateways

All matches to this query

Score	Mr(calc):	Delta	Sequence
75.4	1900.8877	-0.0015	RPCFSALEVDETYVPK
0.3	1900.8877	-0.0015	RPCFSALEVDETYVPK

Mascot: http://www.matrixscience.com/

{MATRIX SCIENCE} Mascot Search Results

Peptide View

MS/MS Fragmentation of **RPCFSALEVDETYVPK**
Found in **sp|P02768|ALBU_HUMAN**, Serum albumin OS=Homo sapiens GN=ALB PE=1 SV=2

Match to Query 8779: 1900.885968 from(951.450260,2+) intensity(11274031.0000) scans(13659) rtinseconds(2749) index(10760)
Title: 150808_TTSH_Patient_Plasma_47_Spectrum025196_scans__13659_RTINSECONDS=2749
Data file L:\\Ard_TTSH\\T1D\\T150808_TTSH_Patient_Plasma_47.mgf

Click mouse within plot area to zoom in by factor of two about that point
Or, [Plot from] 100 to 1500 Da [Full range]
Label all possible matches ○ Label matches used for scoring ⊙

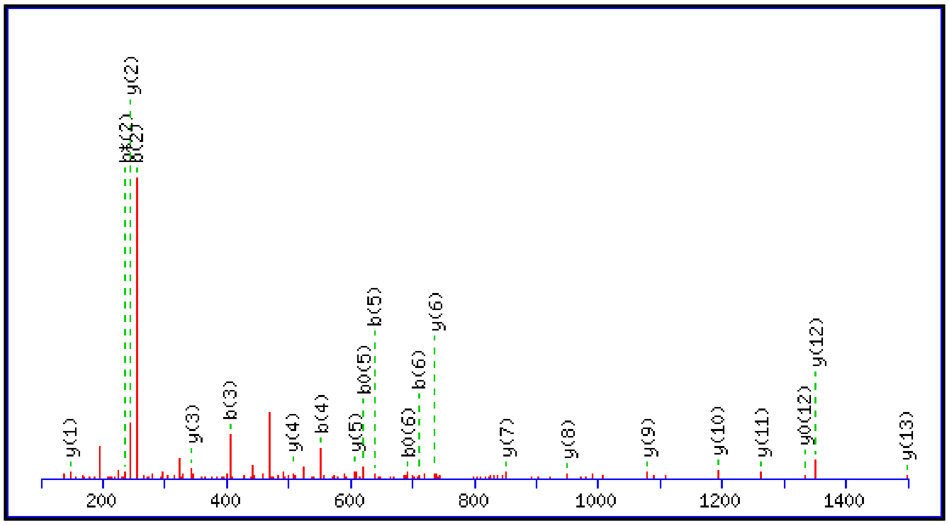

Monoisotopic mass of neutral peptide Mr(calc): 1900.8877
Variable modifications:
C3 : 4Trioxidation (CMWY)
Ions Score: 74 **Expect:** 1.5e-005
Matches : 22/164 fragment ions using 53 most intense peaks (help)

#	b	b^{++}	b*	b^{*++}	b^0	b^{0++}	Seq.	y	y^{++}	y*	y^{*++}	y^0	y^{0++}	#
1	157.1084	79.0578	140.0818	70.5446			R							16
2	**254.1612**	127.5842	237.1346	119.0709			P	1745.7938	873.4006	1728.7673	864.8873	1727.7833	864.3953	15
3	**405.1551**	203.0812	388.1285	194.5679			C	1648.7411	824.8742	1631.7145	816.3609	1630.7305	815.8689	14
4	**552.2235**	276.6154	535.1969	268.1021			F	***1497.7472***	749.3772	1480.7206	740.8639	1479.7366	740.3719	13
5	**639.2555**	320.1314	622.2290	311.6181	621.2450	311.1261	S	***1350.6787***	675.8430	1333.6522	667.3297	1332.6682	666.8377	12
6	**710.2926**	355.6500	693.2661	347.1367	692.2821	346.6447	A	***1263.6467***	632.3270	1246.6202	623.8137	1245.6361	623.3217	11
7	823.3767	412.1920	806.3502	403.6787	805.3661	403.1867	L	***1192.6096***	596.8084	1175.5830	588.2952	1174.5990	587.8032	10
8	952.4193	476.7133	935.3927	468.2000	934.4087	467.7080	E	***1079.5255***	540.2664	1062.4990	531.7531	1061.5150	531.2611	9
9	1051.4877	526.2475	1034.4612	517.7342	1033.4771	517.2422	V	***950.4829***	475.7451	933.4564	467.2318	932.4724	466.7398	8
10	1166.5147	583.7610	1149.4881	575.2477	1148.5041	574.7557	D	***851.4145***	426.2109	834.3880	417.6976	833.4040	417.2056	7
11	1295.5572	648.2823	1278.5307	639.7690	1277.5467	639.2770	E	***736.3876***	368.6974	719.3610	360.1842	718.3770	359.6921	6
12	1396.6049	698.8061	1379.5784	690.2928	1378.5944	689.8008	T	***607.3450***	304.1761	590.3184	295.6629	589.3344	295.1709	5
13	1559.6683	780.3378	1542.6417	771.8245	1541.6577	771.3325	Y	***506.2973***	253.6523	489.2708	245.1390			4
14	1658.7367	829.8720	1641.7101	821.3587	1640.7261	820.8667	V	***343.2340***	172.1206	326.2074	163.6074			3
15	1755.7894	878.3984	1738.7629	869.8851	1737.7789	869.3931	P	***244.1656***	122.5864	227.1390	114.0731			2
16							K	***147.1128***	74.0600	130.0863	65.5468			1

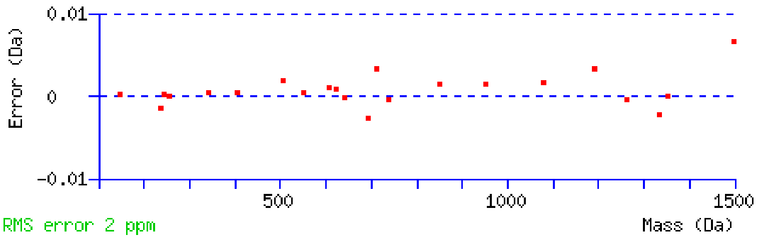

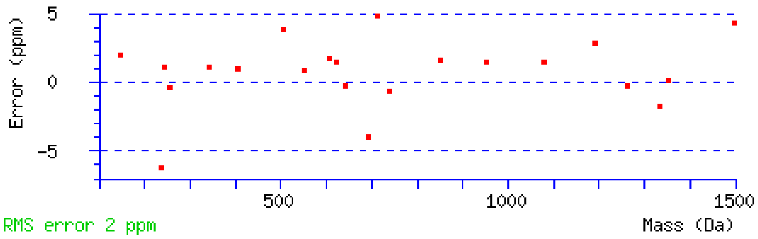

NCBI **BLAST** search of RPCFSALEVDETYVPK
(Parameters: blastp, nr protein database, expect=20000, no filter, PAM30)
Other BLAST web gateways

All matches to this query

Score	Mr(calc):	Delta	Sequence
74.0	1900.8877	-0.0017	RPCFSALEVDETYVPK

Mascot Search Results

Peptide View

MS/MS Fragmentation of **RPCFSALEVDETYVPK**
Found in **sp|P02768|ALBU_HUMAN**, Serum albumin OS=Homo sapiens GN=ALB PE=1 SV=2

Match to Query 11558: 1900.886688 from(951.450620,2+) intensity(56712968.0000) scans(14692) rtinseconds(2639) index(12737)
Title: N50818_TTSH_Patient_Plasma_76_Spectrum012875_scans__14692_RTINSECONDS=2639
Data file L:\\Ard_TTSH\\TN1D\\TN50818_TTSH_Patient_Plasma_76.mgf

Click mouse within plot area to zoom in by factor of two about that point
Or, to Da
Label all possible matches Label matches used for scoring

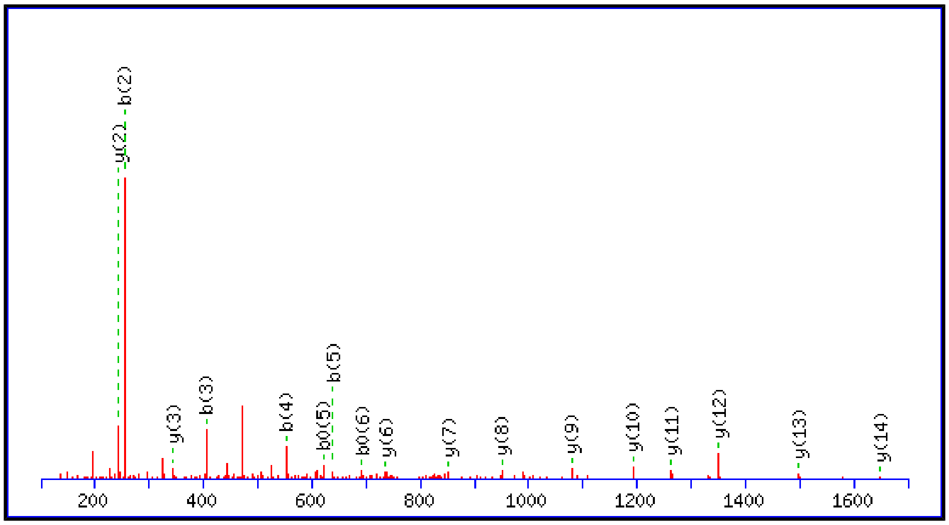

Monoisotopic mass of neutral peptide Mr(calc): 1900.8877
Variable modifications:
C3 : 4Trioxidation (CMWY)
Ions Score: 73 **Expect:** 1.7e-005
Matches : 17/164 fragment ions using 31 most intense peaks (help)

#	b	b^{++}	b*	b^{*++}	b^{0}	b^{0++}	Seq.	y	y^{++}	y*	y^{*++}	y^{0}	y^{0++}	#
1	157.1084	79.0578	140.0818	70.5446			R							16
2	**254.1612**	127.5842	237.1346	119.0709			P	1745.7938	873.4006	1728.7673	864.8873	1727.7833	864.3953	15
3	**405.1551**	203.0812	388.1285	194.5679			C	***1648.7411***	824.8742	1631.7145	816.3609	1630.7305	815.8689	14
4	**552.2235**	276.6154	535.1969	268.1021			F	***1497.7472***	749.3772	1480.7206	740.8639	1479.7366	740.3719	13
5	**639.2555**	320.1314	622.2290	311.6181	621.2450	311.1261	S	***1350.6787***	675.8430	1333.6522	667.3297	1332.6682	666.8377	12
6	710.2926	355.6500	693.2661	347.1367	692.2821	346.6447	A	***1263.6467***	632.3270	1246.6202	623.8137	1245.6361	623.3217	11
7	823.3767	412.1920	806.3502	403.6787	805.3661	403.1867	L	***1192.6096***	596.8084	1175.5830	588.2952	1174.5990	587.8032	10
8	952.4193	476.7133	935.3927	468.2000	934.4087	467.7080	E	***1079.5255***	540.2664	1062.4990	531.7531	1061.5150	531.2611	9
9	1051.4877	526.2475	1034.4612	517.7342	1033.4771	517.2422	V	***950.4829***	475.7451	933.4564	467.2318	932.4724	466.7398	8
10	1166.5147	583.7610	1149.4881	575.2477	1148.5041	574.7557	D	***851.4145***	426.2109	834.3880	417.6976	833.4040	417.2056	7
11	1295.5572	648.2823	1278.5307	639.7690	1277.5467	639.2770	E	***736.3876***	368.6974	719.3610	360.1842	718.3770	359.6921	6
12	1396.6049	698.8061	1379.5784	690.2928	1378.5944	689.8008	T	607.3450	304.1761	590.3184	295.6629	589.3344	295.1709	5
13	1559.6683	780.3378	1542.6417	771.8245	1541.6577	771.3325	Y	506.2973	253.6523	489.2708	245.1390			4
14	1658.7367	829.8720	1641.7101	821.3587	1640.7261	820.8667	V	***343.2340***	172.1206	326.2074	163.6074			3
15	1755.7894	878.3984	1738.7629	869.8851	1737.7789	869.3931	P	***244.1656***	122.5864	227.1390	114.0731			2
16							K	147.1128	74.0600	130.0863	65.5468			1

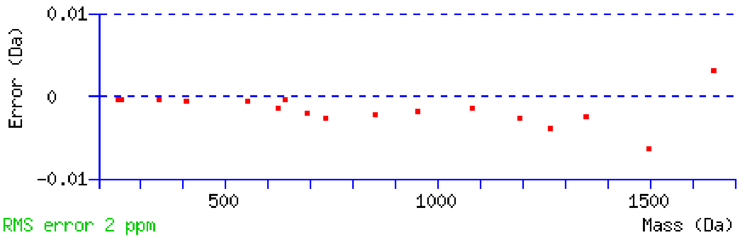

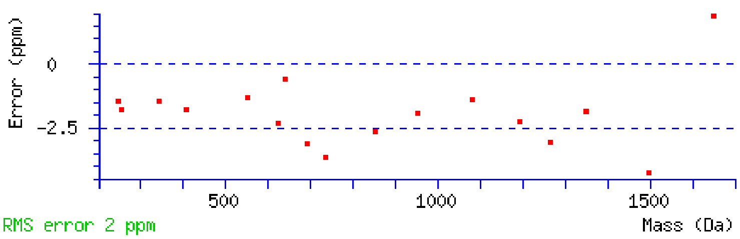

NCBI **BLAST** search of RPCFSALEVDETYVPK
(Parameters: blastp, nr protein database, expect=20000, no filter, PAM30)
Other BLAST web gateways

All matches to this query

Score	Mr(calc):	Delta	Sequence
73.4	1900.8877	-0.0010	RPCFSALEVDETYVPK
0.5	1900.8877	-0.0010	RPCFSALEVDETYVPK

Mascot Search Results

Peptide View

MS/MS Fragmentation of **RPCFSALEVDETYVPK**
Found in **sp|P02768|ALBU_HUMAN**, Serum albumin OS=Homo sapiens GN=ALB PE=1 SV=2

Match to Query 8923: 1900.883148 from(951.448850,2+) intensity(1127290.6250) scans(13623) rtinseconds(2383) index(11907)
Title: 150801_TTSH_Patient_Plasma_48_Spectrum029381_scans__13623_RTINSECONDS=2383
Data file L:\\Ard_TTSH\\T1D\\T150801_TTSH_Patient_Plasma_48.mgf

Click mouse within plot area to zoom in by factor of two about that point
Or, Plot from 100 to 1400 Da Full range
Label all possible matches ○ Label matches used for scoring ⊙

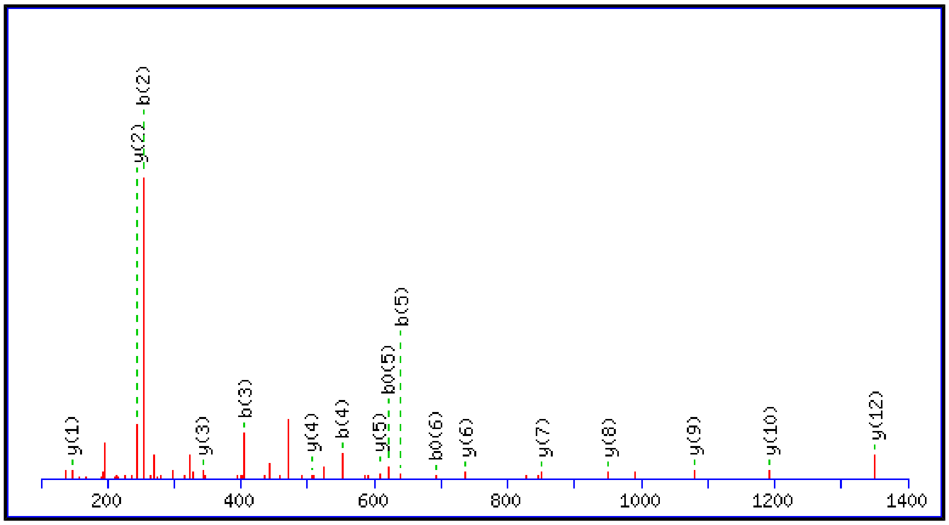

Monoisotopic mass of neutral peptide Mr(calc): 1900.8877
Variable modifications:
C3 : 4Trioxidation (CMWY)
Ions Score: 72 **Expect:** 2.1e-005
Matches : 17/164 fragment ions using 32 most intense peaks (help)

#	b	b++	b*	b*++	b0	b0++	Seq.	y	y++	y*	y*++	y0	y0++	#
1	157.1084	79.0578	140.0818	70.5446			R							16
2	**254.1612**	127.5842	237.1346	119.0709			P	1745.7938	873.4006	1728.7673	864.8873	1727.7833	864.3953	15
3	**405.1551**	203.0812	388.1285	194.5679			C	1648.7411	824.8742	1631.7145	816.3609	1630.7305	815.8689	14
4	**552.2235**	276.6154	535.1969	268.1021			F	1497.7472	749.3772	1480.7206	740.8639	1479.7366	740.3719	13
5	**639.2555**	320.1314	622.2290	311.6181	621.2450	311.1261	S	***1350.6787***	675.8430	1333.6522	667.3297	1332.6682	666.8377	12
6	710.2926	355.6500	693.2661	347.1367	692.2821	346.6447	A	1263.6467	632.3270	1246.6202	623.8137	1245.6361	623.3217	11
7	823.3767	412.1920	806.3502	403.6787	805.3661	403.1867	L	***1192.6096***	596.8084	1175.5830	588.2952	1174.5990	587.8032	10
8	952.4193	476.7133	935.3927	468.2000	934.4087	467.7080	E	***1079.5255***	540.2664	1062.4990	531.7531	1061.5150	531.2611	9
9	1051.4877	526.2475	1034.4612	517.7342	1033.4771	517.2422	V	***950.4829***	475.7451	933.4564	467.2318	932.4724	466.7398	8
10	1166.5147	583.7610	1149.4881	575.2477	1148.5041	574.7557	D	***851.4145***	426.2109	834.3880	417.6976	833.4040	417.2056	7
11	1295.5572	648.2823	1278.5307	639.7690	1277.5467	639.2770	E	***736.3876***	368.6974	719.3610	360.1842	718.3770	359.6921	6
12	1396.6049	698.8061	1379.5784	690.2928	1378.5944	689.8008	T	***607.3450***	304.1761	590.3184	295.6629	589.3344	295.1709	5
13	1559.6683	780.3378	1542.6417	771.8245	1541.6577	771.3325	Y	***506.2973***	253.6523	489.2708	245.1390			4
14	1658.7367	829.8720	1641.7101	821.3587	1640.7261	820.8667	V	***343.2340***	172.1206	326.2074	163.6074			3
15	1755.7894	878.3984	1738.7629	869.8851	1737.7789	869.3931	P	***244.1656***	122.5864	227.1390	114.0731			2
16							K	***147.1128***	74.0600	130.0863	65.5468			1

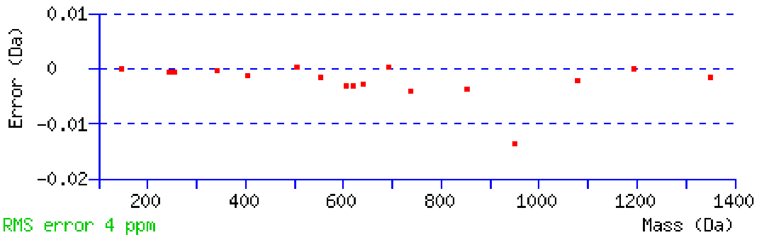

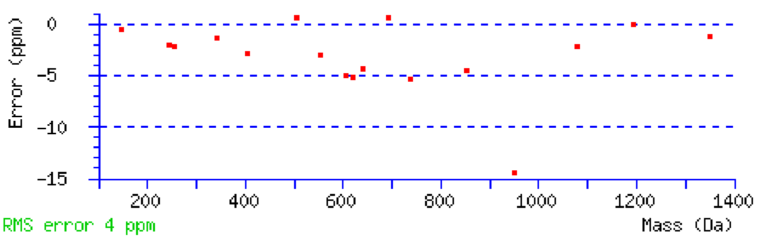

NCBI **BLAST** search of RPCFSALEVDETYVPK
(Parameters: blastp, nr protein database, expect=20000, no filter, PAM30)
Other BLAST web gateways

All matches to this query

Score	Mr(calc):	Delta	Sequence
72.5	1900.8877	-0.0045	RPCFSALEVDETYVPK
1.8	1900.8877	-0.0045	RPCFSALEVDETYVPK

Mascot: http://www.matrixscience.com/

Mascot Search Results

Peptide View

MS/MS Fragmentation of **RPCFSALEVDETYVPK**
Found in **sp|P02768|ALBU_HUMAN**, Serum albumin OS=Homo sapiens GN=ALB PE=1 SV=2

Match to Query 9352: 1900.889868 from(951.452210,2+) intensity(1108798.7500) scans(13509) rtinseconds(2472) index(11629)
Title: 150825_TTSH_Patient_Plasma_02_Spectrum028388_scans__13509_RTINSECONDS=2472
Data file L:\\Ard_TTSH\\T1D\\T150825_TTSH_Patient_Plasma_02.mgf

Click mouse within plot area to zoom in by factor of two about that point
Or, Plot from 100 to 1700 Da Full range
Label all possible matches ○ Label matches used for scoring ⊙

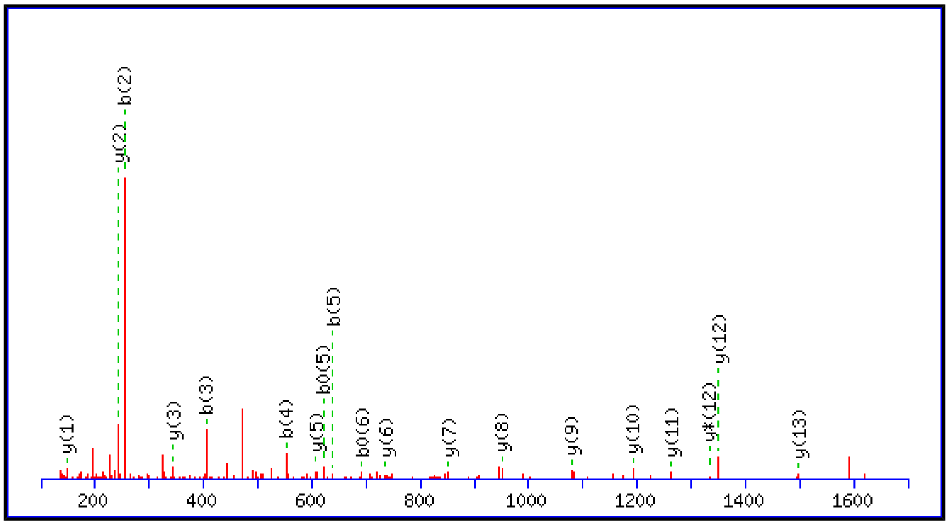

Monoisotopic mass of neutral peptide Mr(calc): 1900.8877
Variable modifications:
C3 : 4Trioxidation (CMWY)
Ions Score: 72 **Expect:** 2.2e-005
Matches : 19/164 fragment ions using 41 most intense peaks (help)

#	b	b^{++}	b*	b^{*++}	b^0	b^{0++}	Seq.	y	y^{++}	y*	y^{*++}	y^0	y^{0++}	#
1	157.1084	79.0578	140.0818	70.5446			R							16
2	**254.1612**	127.5842	237.1346	119.0709			P	1745.7938	873.4006	1728.7673	864.8873	1727.7833	864.3953	15
3	**405.1551**	203.0812	388.1285	194.5679			C	1648.7411	824.8742	1631.7145	816.3609	1630.7305	815.8689	14
4	**552.2235**	276.6154	535.1969	268.1021			F	***1497.7472***	749.3772	1480.7206	740.8639	1479.7366	740.3719	13
5	**639.2555**	320.1314	622.2290	311.6181	621.2450	311.1261	S	***1350.6787***	675.8430	1333.6522	667.3297	1332.6682	666.8377	12
6	710.2926	355.6500	693.2661	347.1367	692.2821	346.6447	A	***1263.6467***	632.3270	1246.6202	623.8137	1245.6361	623.3217	11
7	823.3767	412.1920	806.3502	403.6787	805.3661	403.1867	L	***1192.6096***	596.8084	1175.5830	588.2952	1174.5990	587.8032	10
8	952.4193	476.7133	935.3927	468.2000	934.4087	467.7080	E	***1079.5255***	540.2664	1062.4990	531.7531	1061.5150	531.2611	9
9	1051.4877	526.2475	1034.4612	517.7342	1033.4771	517.2422	V	***950.4829***	475.7451	933.4564	467.2318	932.4724	466.7398	8
10	1166.5147	583.7610	1149.4881	575.2477	1148.5041	574.7557	D	***851.4145***	426.2109	834.3880	417.6976	833.4040	417.2056	7
11	1295.5572	648.2823	1278.5307	639.7690	1277.5467	639.2770	E	***736.3876***	368.6974	719.3610	360.1842	718.3770	359.6921	6
12	1396.6049	698.8061	1379.5784	690.2928	1378.5944	689.8008	T	***607.3450***	304.1761	590.3184	295.6629	589.3344	295.1709	5
13	1559.6683	780.3378	1542.6417	771.8245	1541.6577	771.3325	Y	506.2973	253.6523	489.2708	245.1390			4
14	1658.7367	829.8720	1641.7101	821.3587	1640.7261	820.8667	V	***343.2340***	172.1206	326.2074	163.6074			3
15	1755.7894	878.3984	1738.7629	869.8851	1737.7789	869.3931	P	***244.1656***	122.5864	227.1390	114.0731			2
16							K	***147.1128***	74.0600	130.0863	65.5468			1

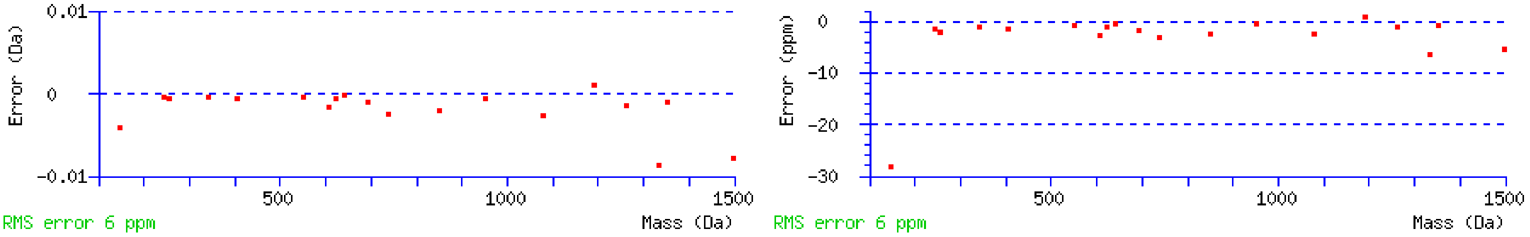

NCBI **BLAST** search of RPCFSALEVDETYVPK
(Parameters: blastp, nr protein database, expect=20000, no filter, PAM30)
Other BLAST web gateways

All matches to this query

Score	Mr(calc):	Delta	Sequence
72.5	1900.8877	0.0022	RPCFSALEVDETYVPK

{MATRIX SCIENCE} Mascot Search Results

Peptide View

MS/MS Fragmentation of **RPCFSALEVDETYVPK**
Found in **sp|P02768|ALBU_HUMAN**, Serum albumin OS=Homo sapiens GN=ALB PE=1 SV=2

Match to Query 8690: 1900.885468 from(951.450010,2+) intensity(2222695.0000) scans(13364) rtinseconds(2373) index(11437)
Title: 150801_TTSH_Patient_Plasma_45_Spectrum028863_scans__13364_RTINSECONDS=2373
Data file L:\\Ard_TTSH\\T1D\\T150801_TTSH_Patient_Plasma_45.mgf

Click mouse within plot area to zoom in by factor of two about that point
Or, Plot from 100 to 1500 Da Full range
Label all possible matches ○ Label matches used for scoring ⦿

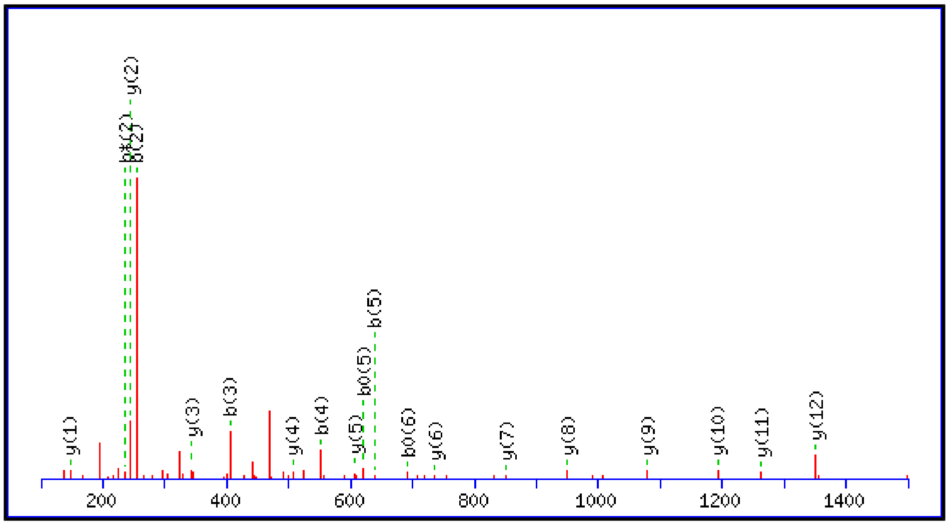


```
Monoisotopic mass of neutral peptide Mr(calc): 1900.8877
Variable modifications:
C3     : 4Trioxidation (CMWY)
Ions Score: 72  Expect: 2.4e-005
Matches : 19/164 fragment ions using 42 most intense peaks   (help)
```

#	b	b^{++}	b*	b^{*++}	b^0	b^{0++}	Seq.	y	y^{++}	y*	y^{*++}	y^0	y^{0++}	#
1	157.1084	79.0578	140.0818	70.5446			R							16
2	**254.1612**	127.5842	237.1346	119.0709			P	1745.7938	873.4006	1728.7673	864.8873	1727.7833	864.3953	15
3	**405.1551**	203.0812	388.1285	194.5679			C	1648.7411	824.8742	1631.7145	816.3609	1630.7305	815.8689	14
4	**552.2235**	276.6154	535.1969	268.1021			F	1497.7472	749.3772	1480.7206	740.8639	1479.7366	740.3719	13
5	**639.2555**	320.1314	622.2290	311.6181	621.2450	311.1261	S	***1350.6787***	675.8430	1333.6522	667.3297	1332.6682	666.8377	12
6	710.2926	355.6500	693.2661	347.1367	692.2821	346.6447	A	***1263.6467***	632.3270	1246.6202	623.8137	1245.6361	623.3217	11
7	823.3767	412.1920	806.3502	403.6787	805.3661	403.1867	L	***1192.6096***	596.8084	1175.5830	588.2952	1174.5990	587.8032	10
8	952.4193	476.7133	935.3927	468.2000	934.4087	467.7080	E	***1079.5255***	540.2664	1062.4990	531.7531	1061.5150	531.2611	9
9	1051.4877	526.2475	1034.4612	517.7342	1033.4771	517.2422	V	***950.4829***	475.7451	933.4564	467.2318	932.4724	466.7398	8
10	1166.5147	583.7610	1149.4881	575.2477	1148.5041	574.7557	D	***851.4145***	426.2109	834.3880	417.6976	833.4040	417.2056	7
11	1295.5572	648.2823	1278.5307	639.7690	1277.5467	639.2770	E	***736.3876***	368.6974	719.3610	360.1842	718.3770	359.6921	6
12	1396.6049	698.8061	1379.5784	690.2928	1378.5944	689.8008	T	***607.3450***	304.1761	590.3184	295.6629	589.3344	295.1709	5
13	1559.6683	780.3378	1542.6417	771.8245	1541.6577	771.3325	Y	***506.2973***	253.6523	489.2708	245.1390			4
14	1658.7367	829.8720	1641.7101	821.3587	1640.7261	820.8667	V	***343.2340***	172.1206	326.2074	163.6074			3
15	1755.7894	878.3984	1738.7629	869.8851	1737.7789	869.3931	P	***244.1656***	122.5864	227.1390	114.0731			2
16							K	***147.1128***	74.0600	130.0863	65.5468			1

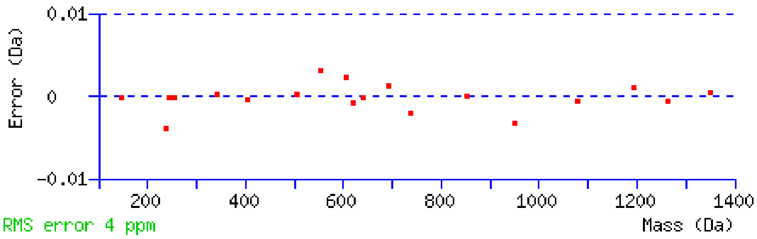

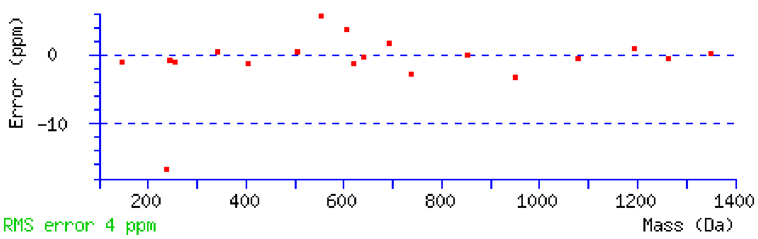

NCBI **BLAST** search of RPCFSALEVDETYVPK
(Parameters: blastp, nr protein database, expect=20000, no filter, PAM30)
Other BLAST web gateways

All matches to this query

Score	Mr(calc):	Delta	Sequence
71.8	1900.8877	-0.0022	RPCFSALEVDETYVPK

Mascot: http://www.matrixscience.com/

{MATRIX SCIENCE} Mascot Search Results

Peptide View

MS/MS Fragmentation of **RPCFSALEVDETYVPK**
Found in **sp|P02768|ALBU_HUMAN**, Serum albumin OS=Homo sapiens GN=ALB PE=1 SV=2

Match to Query 8982: 1900.887428 from(951.450990,2+) intensity(1071329.2500) scans(13014) rtinseconds(2730) index(9904)
Title: 150808_TTSH_Patient_Plasma_19_Spectrum023431_scans__13014_RTINSECONDS=2730
Data file L:\\Ard_TTSH\\T1D\\T150808_TTSH_Patient_Plasma_19.mgf

Click mouse within plot area to zoom in by factor of two about that point
Or, Plot from 100 to 1500 Da Full range
Label all possible matches ○ Label matches used for scoring ⦿

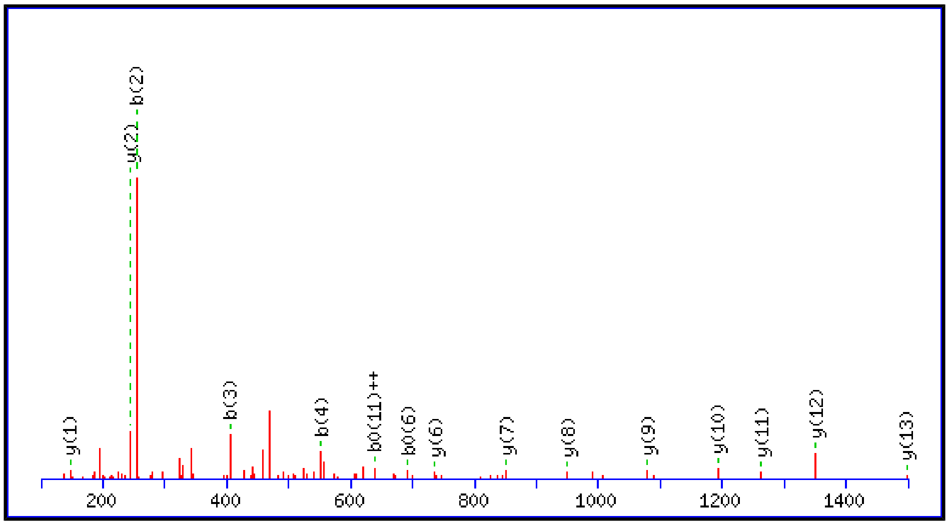

Monoisotopic mass of neutral peptide Mr(calc): 1900.8877
Variable modifications:
C3 : 4Trioxidation (CMWY)
Ions Score: 72 **Expect:** 2.6e-005
Matches : 15/164 fragment ions using 24 most intense peaks (help)

#	b	b^{++}	b*	b^{*++}	b^0	b^{0++}	Seq.	y	y^{++}	y*	y^{*++}	y^0	y^{0++}	#
1	157.1084	79.0578	140.0818	70.5446			R							16
2	**254.1612**	127.5842	237.1346	119.0709			P	1745.7938	873.4006	1728.7673	864.8873	1727.7833	864.3953	15
3	**405.1551**	203.0812	388.1285	194.5679			C	1648.7411	824.8742	1631.7145	816.3609	1630.7305	815.8689	14
4	**552.2235**	276.6154	535.1969	268.1021			F	***1497.7472***	749.3772	1480.7206	740.8639	1479.7366	740.3719	13
5	639.2555	320.1314	622.2290	311.6181	621.2450	311.1261	S	***1350.6787***	675.8430	1333.6522	667.3297	1332.6682	666.8377	12
6	710.2926	355.6500	693.2661	347.1367	692.2821	346.6447	A	***1263.6467***	632.3270	1246.6202	623.8137	1245.6361	623.3217	11
7	823.3767	412.1920	806.3502	403.6787	805.3661	403.1867	L	***1192.6096***	596.8084	1175.5830	588.2952	1174.5990	587.8032	10
8	952.4193	476.7133	935.3927	468.2000	934.4087	467.7080	E	***1079.5255***	540.2664	1062.4990	531.7531	1061.5150	531.2611	9
9	1051.4877	526.2475	1034.4612	517.7342	1033.4771	517.2422	V	***950.4829***	475.7451	933.4564	467.2318	932.4724	466.7398	8
10	1166.5147	583.7610	1149.4881	575.2477	1148.5041	574.7557	D	***851.4145***	426.2109	834.3880	417.6976	833.4040	417.2056	7
11	1295.5572	648.2823	1278.5307	639.7690	1277.5467	639.2770	E	***736.3876***	368.6974	719.3610	360.1842	718.3770	359.6921	6
12	1396.6049	698.8061	1379.5784	690.2928	1378.5944	689.8008	T	607.3450	304.1761	590.3184	295.6629	589.3344	295.1709	5
13	1559.6683	780.3378	1542.6417	771.8245	1541.6577	771.3325	Y	506.2973	253.6523	489.2708	245.1390			4
14	1658.7367	829.8720	1641.7101	821.3587	1640.7261	820.8667	V	343.2340	172.1206	326.2074	163.6074			3
15	1755.7894	878.3984	1738.7629	869.8851	1737.7789	869.3931	P	***244.1656***	122.5864	227.1390	114.0731			2
16							K	***147.1128***	74.0600	130.0863	65.5468			1

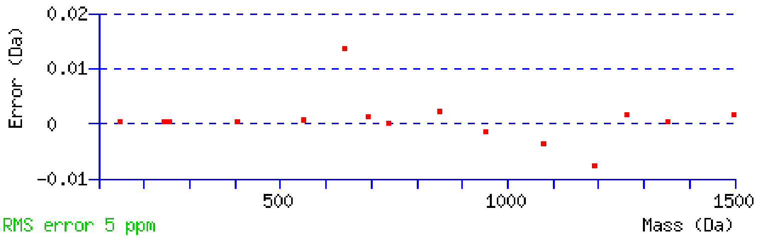

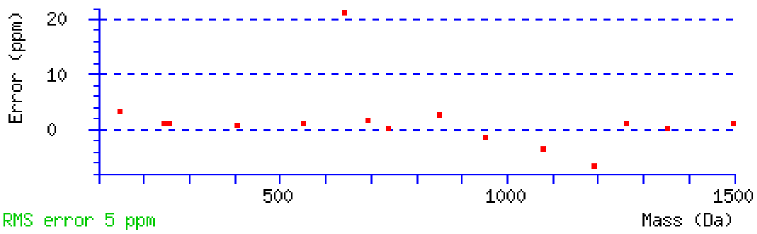

NCBI **BLAST** search of RPCFSALEVDETYVPK
(Parameters: blastp, nr protein database, expect=20000, no filter, PAM30)
Other BLAST web gateways

All matches to this query

Score	Mr(calc):	Delta	Sequence
71.7	1900.8877	-0.0002	RPCFSALEVDETYVPK

Mascot: http://www.matrixscience.com/

Mascot Search Results

Peptide View

MS/MS Fragmentation of **RPCFSALEVDETYVPK**
Found in **sp|P02768|ALBU_HUMAN**, Serum albumin OS=Homo sapiens GN=ALB PE=1 SV=2

Match to Query 8301: 1909.924288 from(955.969420,2+) intensity(3288620.7500) scans(11727) rtinseconds(2159) index(9800)
Title: 150801_TTSH_Patient_Plasma_37_Spectrum026714_scans__11727_RTINSECONDS=2159
Data file L:\\Ard_TTSH\\T1D\\T150801_TTSH_Patient_Plasma_37.mgf

Click mouse within plot area to zoom in by factor of two about that point
Or, Plot from 100 to 1700 Da Full range
Label all possible matches ○ Label matches used for scoring ⊙

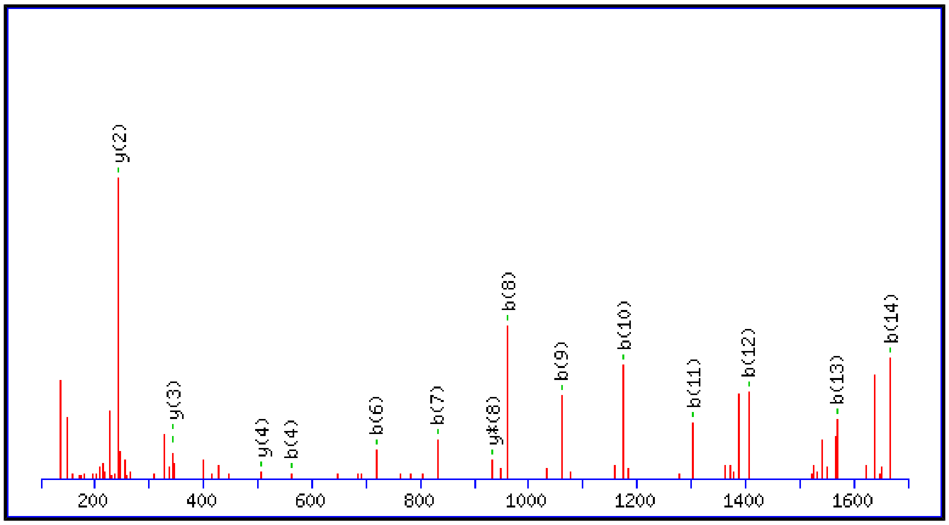

Monoisotopic mass of neutral peptide Mr(calc): 1909.9244
Variable modifications:
C3 : Carbamidomethyl (C)
Ions Score: 93 **Expect:** 1.6e-007
Matches : 14/164 fragment ions using 16 most intense peaks (help)

#	b	b^{++}	b*	b^{*++}	b^0	b^{0++}	Seq.	y	y^{++}	y*	y^{*++}	y^0	y^{0++}	#
1	157.1084	79.0578	140.0818	70.5446			R							16
2	254.1612	127.5842	237.1346	119.0709			P	1754.8306	877.9189	1737.8040	869.4056	1736.8200	868.9136	15
3	414.1918	207.5995	397.1653	199.0863			C	1657.7778	829.3925	1640.7513	820.8793	1639.7672	820.3873	14
4	***561.2602***	281.1337	544.2337	272.6205			F	1497.7472	749.3772	1480.7206	740.8639	1479.7366	740.3719	13
5	648.2922	324.6498	631.2657	316.1365	630.2817	315.6445	S	1350.6787	675.8430	1333.6522	667.3297	1332.6682	666.8377	12
6	***719.3294***	360.1683	702.3028	351.6550	701.3188	351.1630	A	1263.6467	632.3270	1246.6202	623.8137	1245.6361	623.3217	11
7	***832.4134***	416.7103	815.3869	408.1971	814.4029	407.7051	L	1192.6096	596.8084	1175.5830	588.2952	1174.5990	587.8032	10
8	***961.4560***	481.2316	944.4295	472.7184	943.4454	472.2264	E	1079.5255	540.2664	1062.4990	531.7531	1061.5150	531.2611	9
9	***1060.5244***	530.7659	1043.4979	522.2526	1042.5139	521.7606	V	950.4829	475.7451	933.4564	467.2318	932.4724	466.7398	8
10	***1175.5514***	588.2793	1158.5248	579.7660	1157.5408	579.2740	D	851.4145	426.2109	834.3880	417.6976	833.4040	417.2056	7
11	***1304.5940***	652.8006	1287.5674	644.2873	1286.5834	643.7953	E	736.3876	368.6974	719.3610	360.1842	718.3770	359.6921	6
12	***1405.6416***	703.3245	1388.6151	694.8112	1387.6311	694.3192	T	607.3450	304.1761	590.3184	295.6629	589.3344	295.1709	5
13	***1568.7050***	784.8561	1551.6784	776.3428	1550.6944	775.8508	Y	**506.2973**	253.6523	489.2708	245.1390			4
14	***1667.7734***	834.3903	1650.7468	825.8771	1649.7628	825.3850	V	**343.2340**	172.1206	326.2074	163.6074			3
15	1764.8261	882.9167	1747.7996	874.4034	1746.8156	873.9114	P	**244.1656**	122.5864	227.1390	114.0731			2
16							K	147.1128	74.0600	130.0863	65.5468			1

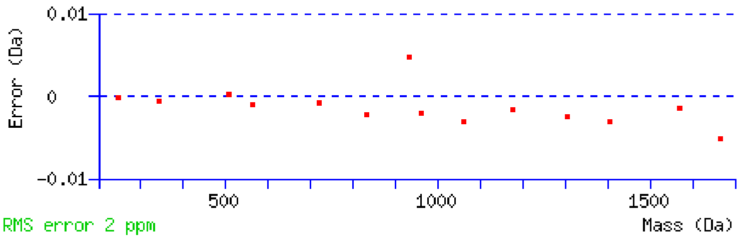

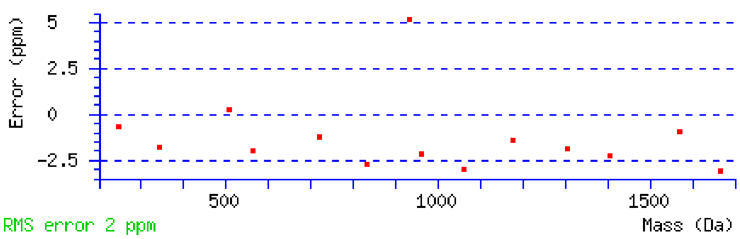

NCBI **BLAST** search of RPCFSALEVDETYVPK
(Parameters: blastp, nr protein database, expect=20000, no filter, PAM30)
Other BLAST web gateways

All matches to this query

Score	Mr(calc):	Delta	Sequence
93.5	1909.9244	-0.0001	RPCFSALEVDETYVPK

Mascot Search Results

Peptide View

MS/MS Fragmentation of **RPCFSALEVDETYVPK**
Found in **sp|P02768|ALBU_HUMAN**, Serum albumin OS=Homo sapiens GN=ALB PE=1 SV=2

Match to Query 9794: 1909.922948 from(955.968750,2+) intensity(91294160.0000) scans(13964) rtinseconds(2511) index(11966)
Title: 150808_TTSH_Patient_Plasma_38_Spectrum029412_scans__13964_RTINSECONDS=2511
Data file L:\\Ard_TTSH\\T1D\\T150808_TTSH_Patient_Plasma_38.mgf

Click mouse within plot area to zoom in by factor of two about that point
Or, Plot from 100 to 1900 Da Full range
Label all possible matches ○ Label matches used for scoring ⊙

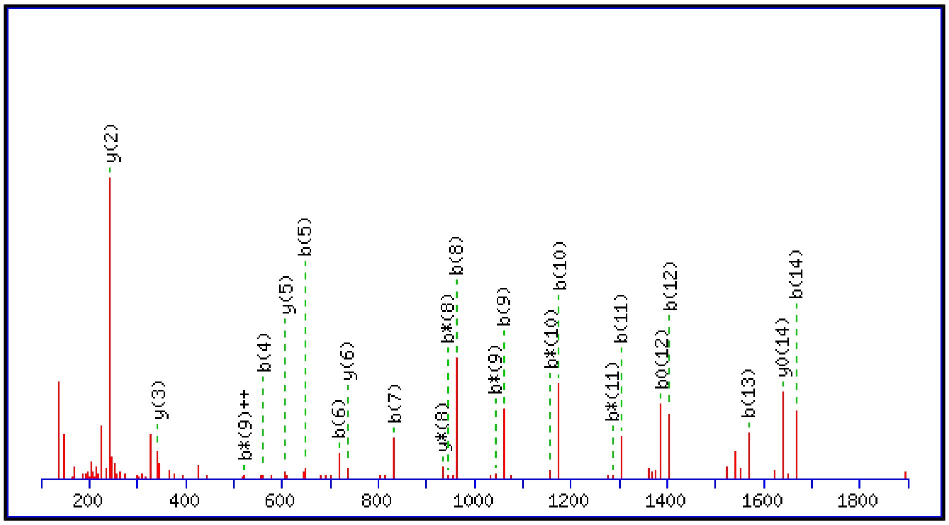

Monoisotopic mass of neutral peptide Mr(calc): 1909.9244
Variable modifications:
C3 : Carbamidomethyl (C)
Ions Score: 89 **Expect:** 4.2e-007
Matches : 23/164 fragment ions using 32 most intense peaks (help)

#	b	b^{++}	b*	b^{*++}	b^0	b^{0++}	Seq.	y	y^{++}	y*	y^{*++}	y^0	y^{0++}	#
1	157.1084	79.0578	140.0818	70.5446			R							16
2	254.1612	127.5842	237.1346	119.0709			P	1754.8306	877.9189	1737.8040	869.4056	1736.8200	868.9136	15
3	414.1918	207.5995	397.1653	199.0863			C	1657.7778	829.3925	1640.7513	820.8793	1639.7672	820.3873	14
4	***561.2602***	281.1337	544.2337	272.6205			F	1497.7472	749.3772	1480.7206	740.8639	1479.7366	740.3719	13
5	***648.2922***	324.6498	631.2657	316.1365	630.2817	315.6445	S	1350.6787	675.8430	1333.6522	667.3297	1332.6682	666.8377	12
6	***719.3294***	360.1683	702.3028	351.6550	701.3188	351.1630	A	1263.6467	632.3270	1246.6202	623.8137	1245.6361	623.3217	11
7	***832.4134***	416.7103	815.3869	408.1971	814.4029	407.7051	L	1192.6096	596.8084	1175.5830	588.2952	1174.5990	587.8032	10
8	***961.4560***	481.2316	944.4295	472.7184	943.4454	472.2264	E	1079.5255	540.2664	1062.4990	531.7531	1061.5150	531.2611	9
9	***1060.5244***	530.7659	1043.4979	522.2526	1042.5139	521.7606	V	950.4829	475.7451	933.4564	467.2318	932.4724	466.7398	8
10	***1175.5514***	588.2793	1158.5248	579.7660	1157.5408	579.2740	D	851.4145	426.2109	834.3880	417.6976	833.4040	417.2056	7
11	***1304.5940***	652.8006	1287.5674	644.2873	1286.5834	643.7953	E	**736.3876**	368.6974	719.3610	360.1842	718.3770	359.6921	6
12	***1405.6416***	703.3245	1388.6151	694.8112	1387.6311	694.3192	T	**607.3450**	304.1761	590.3184	295.6629	589.3344	295.1709	5
13	***1568.7050***	784.8561	1551.6784	776.3428	1550.6944	775.8508	Y	506.2973	253.6523	489.2708	245.1390			4
14	***1667.7734***	834.3903	1650.7468	825.8771	1649.7628	825.3850	V	**343.2340**	172.1206	326.2074	163.6074			3
15	1764.8261	882.9167	1747.7996	874.4034	1746.8156	873.9114	P	**244.1656**	122.5864	227.1390	114.0731			2
16							K	147.1128	74.0600	130.0863	65.5468			1

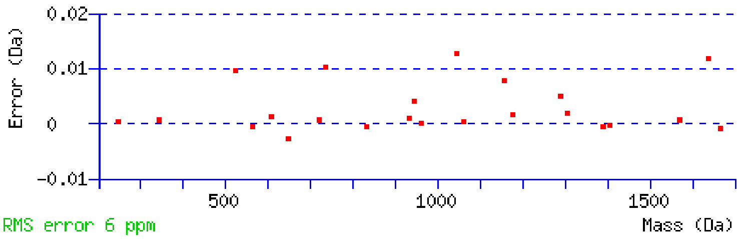

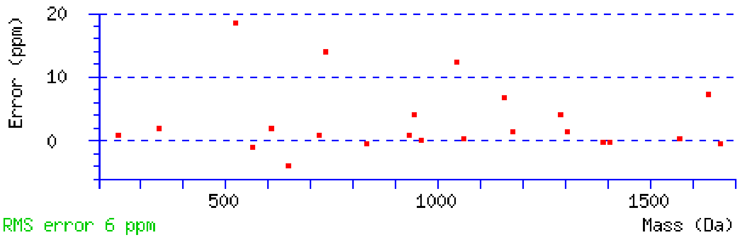

NCBI **BLAST** search of RPCFSALEVDETYVPK
(Parameters: blastp, nr protein database, expect=20000, no filter, PAM30)
Other BLAST web gateways

All matches to this query

Score	Mr(calc):	Delta	Sequence
89.5	1909.9244	-0.0014	RPCFSALEVDETYVPK

Mascot: http://www.matrixscience.com/

Mascot Search Results

Peptide View

MS/MS Fragmentation of **RPCFSALEVDETYVPK**
Found in **sp|P02768|ALBU_HUMAN**, Serum albumin OS=Homo sapiens GN=ALB PE=1 SV=2

Match to Query 8453: 1909.921216 from(478.487580,4+) intensity(20581500.0000) scans(10031) rtinseconds(1875) index(8287)
Title: 150825_TTSH_Patient_Plasma_62_Spectrum024892_scans__10031_RTINSECONDS=1875
Data file L:\\Ard_TTSH\\T1D\\T150825_TTSH_Patient_Plasma_62.mgf

Click mouse within plot area to zoom in by factor of two about that point
Or, Plot from 100 to 1900 Da Full range
Label all possible matches ○ Label matches used for scoring ⊙

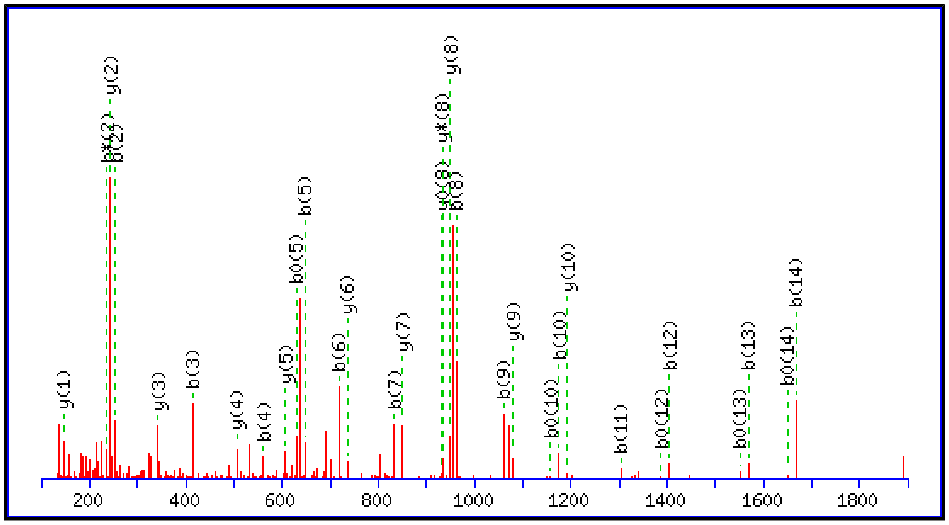

Monoisotopic mass of neutral peptide Mr(calc): 1909.9244
Variable modifications:
C3 : Carbamidomethyl (C)
Ions Score: 89 **Expect:** 4.9e-007
Matches : 31/164 fragment ions using 55 most intense peaks (help)

#	b	b^{++}	b*	b^{*++}	b^0	b^{0++}	Seq.	y	y^{++}	y*	y^{*++}	y^0	y^{0++}	#
1	157.1084	79.0578	140.0818	70.5446			R							16
2	254.1612	127.5842	237.1346	119.0709			P	1754.8306	877.9189	1737.8040	869.4056	1736.8200	868.9136	15
3	414.1918	207.5995	397.1653	199.0863			C	1657.7778	829.3925	1640.7513	820.8793	1639.7672	820.3873	14
4	561.2602	281.1337	544.2337	272.6205			F	1497.7472	749.3772	1480.7206	740.8639	1479.7366	740.3719	13
5	648.2922	324.6498	631.2657	316.1365	630.2817	315.6445	S	1350.6787	675.8430	1333.6522	667.3297	1332.6682	666.8377	12
6	719.3294	360.1683	702.3028	351.6550	701.3188	351.1630	A	1263.6467	632.3270	1246.6202	623.8137	1245.6361	623.3217	11
7	832.4134	416.7103	815.3869	408.1971	814.4029	407.7051	L	1192.6096	596.8084	1175.5830	588.2952	1174.5990	587.8032	10
8	961.4560	481.2316	944.4295	472.7184	943.4454	472.2264	E	1079.5255	540.2664	1062.4990	531.7531	1061.5150	531.2611	9
9	1060.5244	530.7659	1043.4979	522.2526	1042.5139	521.7606	V	950.4829	475.7451	933.4564	467.2318	932.4724	466.7398	8
10	1175.5514	588.2793	1158.5248	579.7660	1157.5408	579.2740	D	851.4145	426.2109	834.3880	417.6976	833.4040	417.2056	7
11	1304.5940	652.8006	1287.5674	644.2873	1286.5834	643.7953	E	736.3876	368.6974	719.3610	360.1842	718.3770	359.6921	6
12	1405.6416	703.3245	1388.6151	694.8112	1387.6311	694.3192	T	607.3450	304.1761	590.3184	295.6629	589.3344	295.1709	5
13	1568.7050	784.8561	1551.6784	776.3428	1550.6944	775.8508	Y	506.2973	253.6523	489.2708	245.1390			4
14	1667.7734	834.3903	1650.7468	825.8771	1649.7628	825.3850	V	343.2340	172.1206	326.2074	163.6074			3
15	1764.8261	882.9167	1747.7996	874.4034	1746.8156	873.9114	P	244.1656	122.5864	227.1390	114.0731			2
16							K	147.1128	74.0600	130.0863	65.5468			1

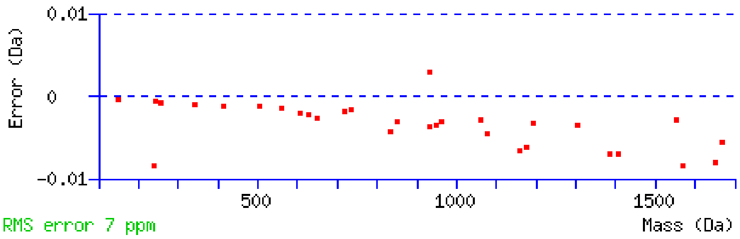

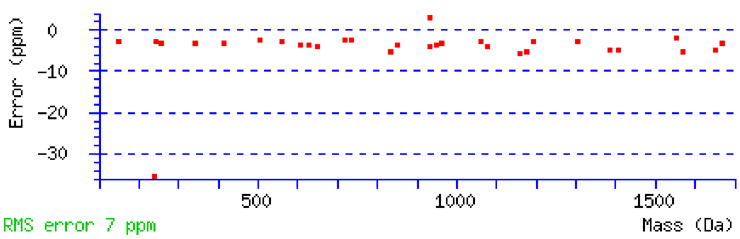

NCBI **BLAST** search of RPCFSALEVDETYVPK
(Parameters: blastp, nr protein database, expect=20000, no filter, PAM30)
Other BLAST web gateways

All matches to this query

Score	Mr(calc):	Delta	Sequence
88.9	1909.9244	-0.0032	RPCFSALEVDETYVPK

Mascot: http://www.matrixscience.com/

Mascot Search Results

Peptide View

MS/MS Fragmentation of **RPCFSALEVDETYVPK**
Found in **sp|P02768|ALBU_HUMAN**, Serum albumin OS=Homo sapiens GN=ALB PE=1 SV=2

Match to Query 12404: 1909.923068 from(955.968810,2+) intensity(2128626.5000) scans(16550) rtinseconds(3005) index(14309)
Title: 150818_TTSH_Patient_Plasma_69_Spectrum030981_scans__16550_RTINSECONDS=3005
Data file L:\\Ard_TTSH\\T1D\\T150818_TTSH_Patient_Plasma_69.mgf

Click mouse within plot area to zoom in by factor of two about that point
Or, Plot from 100 to 1700 Da Full range
Label all possible matches ○ Label matches used for scoring ⦿

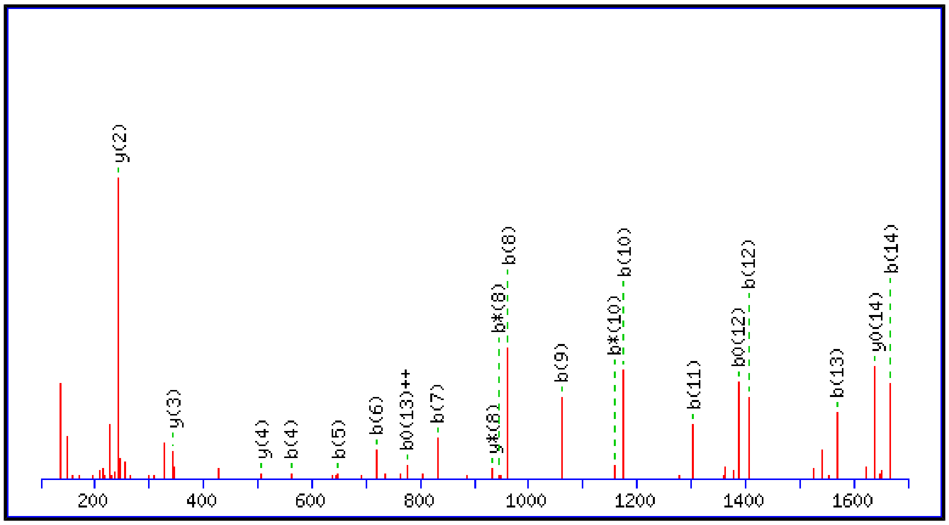

Monoisotopic mass of neutral peptide Mr(calc): 1909.9244
Variable modifications:
C3 : Carbamidomethyl (C)
Ions Score: 89 **Expect:** 4.9e-007
Matches : 20/164 fragment ions using 28 most intense peaks (help)

#	b	b^{++}	b*	b^{*++}	b^{0}	b^{0++}	Seq.	y	y^{++}	y*	y^{*++}	y^{0}	y^{0++}	#
1	157.1084	79.0578	140.0818	70.5446			R							16
2	254.1612	127.5842	237.1346	119.0709			P	1754.8306	877.9189	1737.8040	869.4056	1736.8200	868.9136	15
3	414.1918	207.5995	397.1653	199.0863			C	1657.7778	829.3925	1640.7513	820.8793	1639.7672	820.3873	14
4	***561.2602***	281.1337	544.2337	272.6205			F	1497.7472	749.3772	1480.7206	740.8639	1479.7366	740.3719	13
5	***648.2922***	324.6498	631.2657	316.1365	630.2817	315.6445	S	1350.6787	675.8430	1333.6522	667.3297	1332.6682	666.8377	12
6	***719.3294***	360.1683	702.3028	351.6550	701.3188	351.1630	A	1263.6467	632.3270	1246.6202	623.8137	1245.6361	623.3217	11
7	***832.4134***	416.7103	815.3869	408.1971	814.4029	407.7051	L	1192.6096	596.8084	1175.5830	588.2952	1174.5990	587.8032	10
8	***961.4560***	481.2316	944.4295	472.7184	943.4454	472.2264	E	1079.5255	540.2664	1062.4990	531.7531	1061.5150	531.2611	9
9	***1060.5244***	530.7659	1043.4979	522.2526	1042.5139	521.7606	V	950.4829	475.7451	933.4564	467.2318	932.4724	466.7398	8
10	***1175.5514***	588.2793	1158.5248	579.7660	1157.5408	579.2740	D	851.4145	426.2109	834.3880	417.6976	833.4040	417.2056	7
11	***1304.5940***	652.8006	1287.5674	644.2873	1286.5834	643.7953	E	736.3876	368.6974	719.3610	360.1842	718.3770	359.6921	6
12	***1405.6416***	703.3245	1388.6151	694.8112	1387.6311	694.3192	T	607.3450	304.1761	590.3184	295.6629	589.3344	295.1709	5
13	***1568.7050***	784.8561	1551.6784	776.3428	1550.6944	775.8508	Y	**506.2973**	253.6523	489.2708	245.1390			4
14	***1667.7734***	834.3903	1650.7468	825.8771	1649.7628	825.3850	V	**343.2340**	172.1206	326.2074	163.6074			3
15	1764.8261	882.9167	1747.7996	874.4034	1746.8156	873.9114	P	**244.1656**	122.5864	227.1390	114.0731			2
16							K	147.1128	74.0600	130.0863	65.5468			1

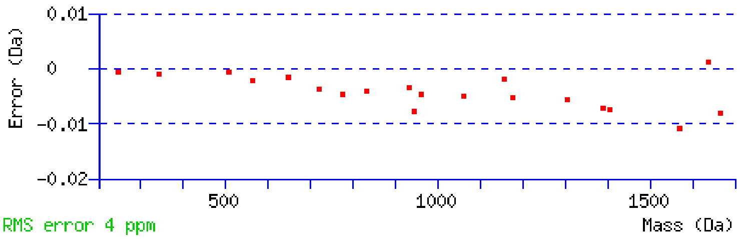

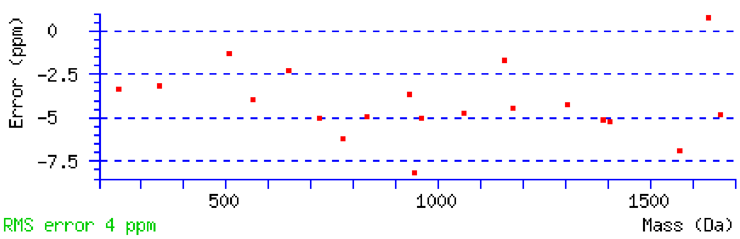

NCBI **BLAST** search of RPCFSALEVDETYVPK
(Parameters: blastp, nr protein database, expect=20000, no filter, PAM30)
Other BLAST web gateways

All matches to this query

Score	Mr(calc):	Delta	Sequence
88.8	1909.9244	-0.0013	RPCFSALEVDETYVPK

Mascot Search Results

Peptide View

MS/MS Fragmentation of **RPCFSALEVDETYVPK**
Found in **sp|P02768|ALBU_HUMAN**, Serum albumin OS=Homo sapiens GN=ALB PE=1 SV=2

Match to Query 9240: 1909.923688 from(955.969120,2+) intensity(885665.5625) scans(13379) rtinseconds(2428) index(11308)
Title: 150808_TTSH_Patient_Plasma_73_Spectrum027773_scans__13379_RTINSECONDS=2428
Data file L:\\Ard_TTSH\\T1D\\T150808_TTSH_Patient_Plasma_73.mgf

Click mouse within plot area to zoom in by factor of two about that point
Or, Plot from 100 to 1700 Da Full range
Label all possible matches ○ Label matches used for scoring ⦿

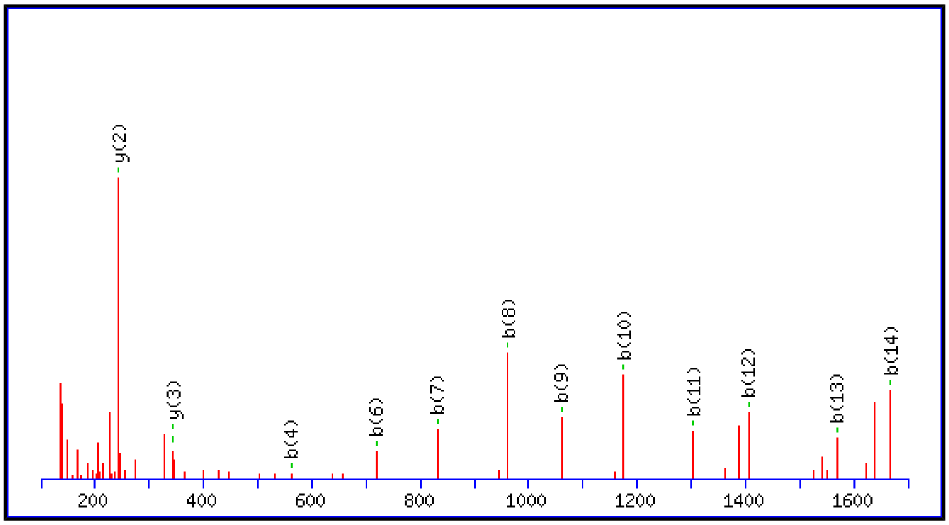

Monoisotopic mass of neutral peptide Mr(calc): 1909.9244
Variable modifications:
C3 : Carbamidomethyl (C)
Ions Score: 89 **Expect:** 5e-007
Matches : 12/164 fragment ions using 15 most intense peaks (help)

#	b	b^{++}	b*	b*$^{++}$	b^{0}	b^{0++}	Seq.	y	y^{++}	y*	y*$^{++}$	y^{0}	y^{0++}	#
1	157.1084	79.0578	140.0818	70.5446			R							16
2	254.1612	127.5842	237.1346	119.0709			P	1754.8306	877.9189	1737.8040	869.4056	1736.8200	868.9136	15
3	414.1918	207.5995	397.1653	199.0863			C	1657.7778	829.3925	1640.7513	820.8793	1639.7672	820.3873	14
4	***561.2602***	281.1337	544.2337	272.6205			F	1497.7472	749.3772	1480.7206	740.8639	1479.7366	740.3719	13
5	648.2922	324.6498	631.2657	316.1365	630.2817	315.6445	S	1350.6787	675.8430	1333.6522	667.3297	1332.6682	666.8377	12
6	***719.3294***	360.1683	702.3028	351.6550	701.3188	351.1630	A	1263.6467	632.3270	1246.6202	623.8137	1245.6361	623.3217	11
7	***832.4134***	416.7103	815.3869	408.1971	814.4029	407.7051	L	1192.6096	596.8084	1175.5830	588.2952	1174.5990	587.8032	10
8	***961.4560***	481.2316	944.4295	472.7184	943.4454	472.2264	E	1079.5255	540.2664	1062.4990	531.7531	1061.5150	531.2611	9
9	***1060.5244***	530.7659	1043.4979	522.2526	1042.5139	521.7606	V	950.4829	475.7451	933.4564	467.2318	932.4724	466.7398	8
10	***1175.5514***	588.2793	1158.5248	579.7660	1157.5408	579.2740	D	851.4145	426.2109	834.3880	417.6976	833.4040	417.2056	7
11	***1304.5940***	652.8006	1287.5674	644.2873	1286.5834	643.7953	E	736.3876	368.6974	719.3610	360.1842	718.3770	359.6921	6
12	***1405.6416***	703.3245	1388.6151	694.8112	1387.6311	694.3192	T	607.3450	304.1761	590.3184	295.6629	589.3344	295.1709	5
13	***1568.7050***	784.8561	1551.6784	776.3428	1550.6944	775.8508	Y	506.2973	253.6523	489.2708	245.1390			4
14	***1667.7734***	834.3903	1650.7468	825.8771	1649.7628	825.3850	V	**343.2340**	172.1206	326.2074	163.6074			3
15	1764.8261	882.9167	1747.7996	874.4034	1746.8156	873.9114	P	**244.1656**	122.5864	227.1390	114.0731			2
16							K	147.1128	74.0600	130.0863	65.5468			1

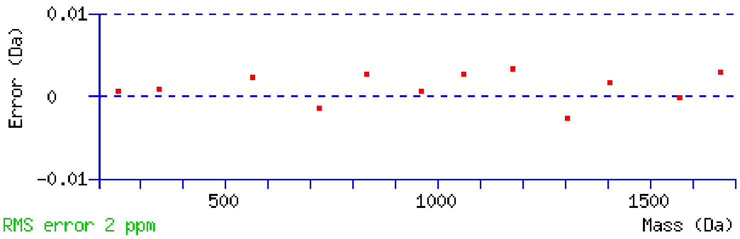

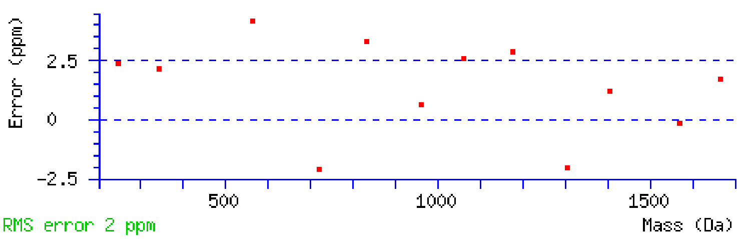

NCBI **BLAST** search of RPCFSALEVDETYVPK
(Parameters: blastp, nr protein database, expect=20000, no filter, PAM30)
Other BLAST web gateways

All matches to this query

Score	Mr(calc):	Delta	Sequence
88.7	1909.9244	-0.0007	RPCFSALEVDETYVPK

Mascot: http://www.matrixscience.com/

Mascot Search Results

Peptide View

MS/MS Fragmentation of **RPCFSALEVDETYVPK**
Found in **sp|P02768|ALBU_HUMAN**, Serum albumin OS=Homo sapiens GN=ALB PE=1 SV=2

Match to Query 8695: 1909.924856 from(478.488490,4+) intensity(7296998.5000) scans(10970) rtinseconds(1945) index(9447)
Title: 150801_TTSH_Patient_Plasma_46_Spectrum026785_scans__10970_RTINSECONDS=1945
Data file L:\\Ard_TTSH\\T1D\\T150801_TTSH_Patient_Plasma_46.mgf

Click mouse within plot area to zoom in by factor of two about that point
Or, Plot from 100 to 1400 Da Full range
Label all possible matches ○ Label matches used for scoring ⊙

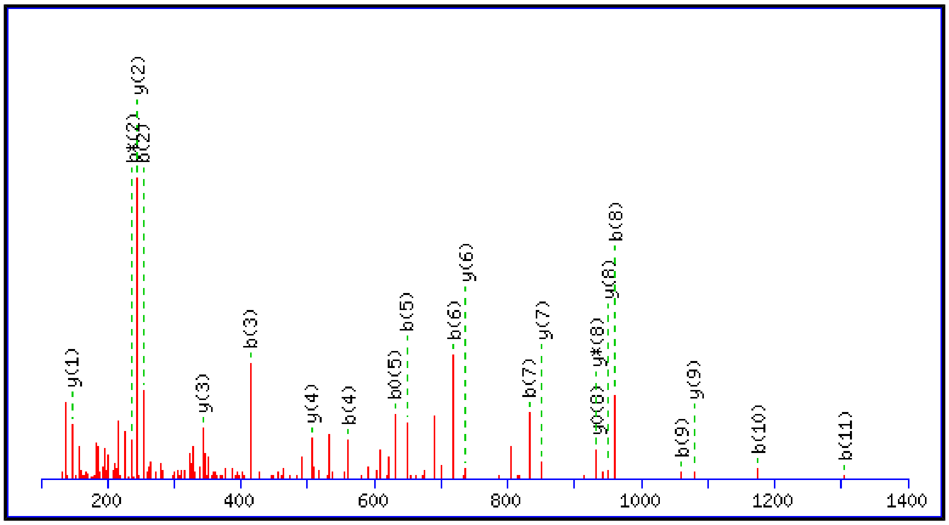

Monoisotopic mass of neutral peptide Mr(calc): 1909.9244
Variable modifications:
C3 : Carbamidomethyl (C)
Ions Score: 88 **Expect:** 5.7e-007
Matches : 22/164 fragment ions using 31 most intense peaks (help)

#	b	b^{++}	b*	b^{*++}	b^{0}	b^{0++}	Seq.	y	y^{++}	y*	y^{*++}	y^{0}	y^{0++}	#
1	157.1084	79.0578	140.0818	70.5446			R							16
2	***254.1612***	127.5842	237.1346	119.0709			P	1754.8306	877.9189	1737.8040	869.4056	1736.8200	868.9136	15
3	***414.1918***	207.5995	397.1653	199.0863			C	1657.7778	829.3925	1640.7513	820.8793	1639.7672	820.3873	14
4	***561.2602***	281.1337	544.2337	272.6205			F	1497.7472	749.3772	1480.7206	740.8639	1479.7366	740.3719	13
5	***648.2922***	324.6498	631.2657	316.1365	630.2817	315.6445	S	1350.6787	675.8430	1333.6522	667.3297	1332.6682	666.8377	12
6	***719.3294***	360.1683	702.3028	351.6550	701.3188	351.1630	A	1263.6467	632.3270	1246.6202	623.8137	1245.6361	623.3217	11
7	***832.4134***	416.7103	815.3869	408.1971	814.4029	407.7051	L	1192.6096	596.8084	1175.5830	588.2952	1174.5990	587.8032	10
8	***961.4560***	481.2316	944.4295	472.7184	943.4454	472.2264	E	***1079.5255***	540.2664	1062.4990	531.7531	1061.5150	531.2611	9
9	***1060.5244***	530.7659	1043.4979	522.2526	1042.5139	521.7606	V	***950.4829***	475.7451	933.4564	467.2318	932.4724	466.7398	8
10	***1175.5514***	588.2793	1158.5248	579.7660	1157.5408	579.2740	D	***851.4145***	426.2109	834.3880	417.6976	833.4040	417.2056	7
11	***1304.5940***	652.8006	1287.5674	644.2873	1286.5834	643.7953	E	***736.3876***	368.6974	719.3610	360.1842	718.3770	359.6921	6
12	1405.6416	703.3245	1388.6151	694.8112	1387.6311	694.3192	T	607.3450	304.1761	590.3184	295.6629	589.3344	295.1709	5
13	1568.7050	784.8561	1551.6784	776.3428	1550.6944	775.8508	Y	***506.2973***	253.6523	489.2708	245.1390			4
14	1667.7734	834.3903	1650.7468	825.8771	1649.7628	825.3850	V	***343.2340***	172.1206	326.2074	163.6074			3
15	1764.8261	882.9167	1747.7996	874.4034	1746.8156	873.9114	P	***244.1656***	122.5864	227.1390	114.0731			2
16							K	***147.1128***	74.0600	130.0863	65.5468			1

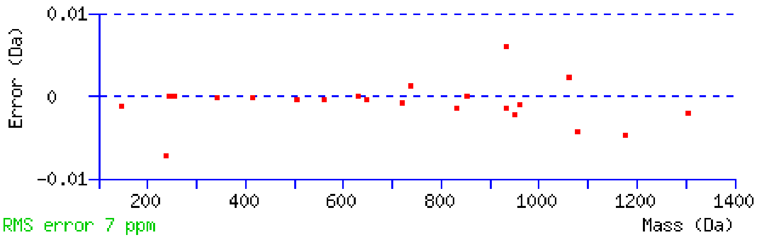

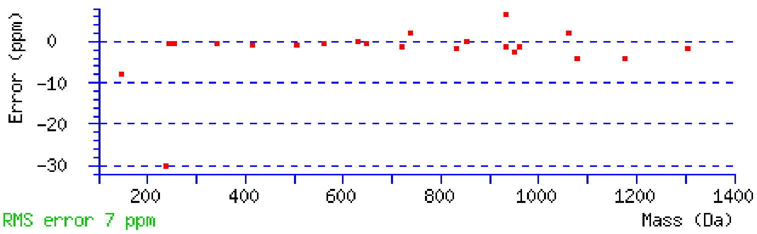

NCBI **BLAST** search of RPCFSALEVDETYVPK
(Parameters: blastp, nr protein database, expect=20000, no filter, PAM30)
Other BLAST web gateways

All matches to this query

Score	Mr(calc):	Delta	Sequence
88.2	1909.9244	0.0005	RPCFSALEVDETYVPK

Mascot Search Results

Peptide View

MS/MS Fragmentation of **RPCFSALEVDETYVPK**
Found in **sp|P02768|ALBU_HUMAN**, Serum albumin OS=Homo sapiens GN=ALB PE=1 SV=2

Match to Query 8181: 1909.918788 from(955.966670,2+) intensity(981982400.0000) scans(10516) rtinseconds(1984) index(8552)
Title: 150825_TTSH_Patient_Plasma_64_Spectrum025406_scans__10516_RTINSECONDS=1984
Data file L:\\Ard_TTSH\\T1D\\T150825_TTSH_Patient_Plasma_64.mgf

Click mouse within plot area to zoom in by factor of two about that point
Or, [Plot from] 100 to 1900 Da [Full range]
Label all possible matches ○ Label matches used for scoring ⊙

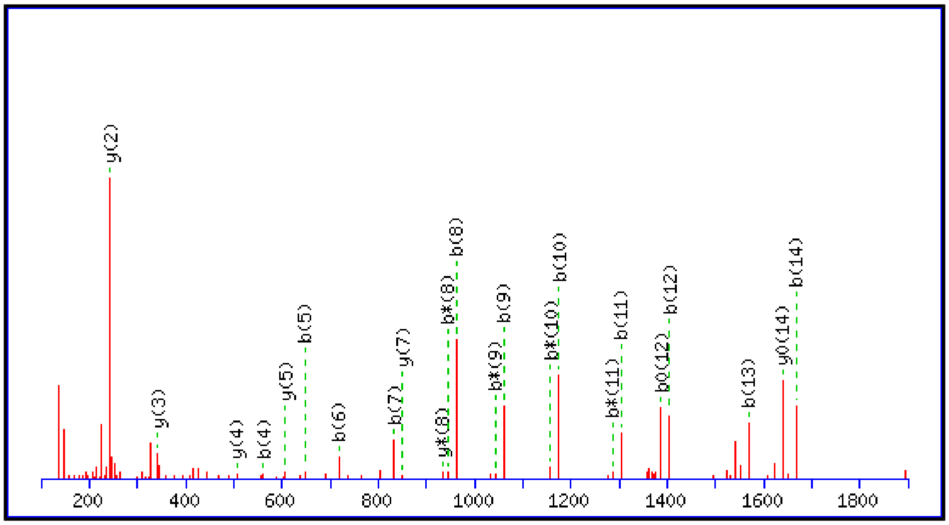

Monoisotopic mass of neutral peptide Mr(calc): 1909.9244
Variable modifications:
C3 : Carbamidomethyl (C)
Ions Score: 87 **Expect:** 6.6e-007
Matches : 23/164 fragment ions using 33 most intense peaks (help)

#	b	b^{++}	b^{*}	b^{*++}	b^{0}	b^{0++}	Seq.	y	y^{++}	y^{*}	y^{*++}	y^{0}	y^{0++}	#
1	157.1084	79.0578	140.0818	70.5446			R							16
2	254.1612	127.5842	237.1346	119.0709			P	1754.8306	877.9189	1737.8040	869.4056	1736.8200	868.9136	15
3	414.1918	207.5995	397.1653	199.0863			C	1657.7778	829.3925	1640.7513	820.8793	1639.7672	820.3873	14
4	***561.2602***	281.1337	544.2337	272.6205			F	1497.7472	749.3772	1480.7206	740.8639	1479.7366	740.3719	13
5	***648.2922***	324.6498	631.2657	316.1365	630.2817	315.6445	S	1350.6787	675.8430	1333.6522	667.3297	1332.6682	666.8377	12
6	***719.3294***	360.1683	702.3028	351.6550	701.3188	351.1630	A	1263.6467	632.3270	1246.6202	623.8137	1245.6361	623.3217	11
7	***832.4134***	416.7103	815.3869	408.1971	814.4029	407.7051	L	1192.6096	596.8084	1175.5830	588.2952	1174.5990	587.8032	10
8	***961.4560***	481.2316	944.4295	472.7184	943.4454	472.2264	E	1079.5255	540.2664	1062.4990	531.7531	1061.5150	531.2611	9
9	***1060.5244***	530.7659	1043.4979	522.2526	1042.5139	521.7606	V	950.4829	475.7451	933.4564	467.2318	932.4724	466.7398	8
10	***1175.5514***	588.2793	1158.5248	579.7660	1157.5408	579.2740	D	**851.4145**	426.2109	834.3880	417.6976	833.4040	417.2056	7
11	***1304.5940***	652.8006	1287.5674	644.2873	1286.5834	643.7953	E	736.3876	368.6974	719.3610	360.1842	718.3770	359.6921	6
12	***1405.6416***	703.3245	1388.6151	694.8112	1387.6311	694.3192	T	**607.3450**	304.1761	590.3184	295.6629	589.3344	295.1709	5
13	***1568.7050***	784.8561	1551.6784	776.3428	1550.6944	775.8508	Y	**506.2973**	253.6523	489.2708	245.1390			4
14	***1667.7734***	834.3903	1650.7468	825.8771	1649.7628	825.3850	V	**343.2340**	172.1206	326.2074	163.6074			3
15	1764.8261	882.9167	1747.7996	874.4034	1746.8156	873.9114	P	**244.1656**	122.5864	227.1390	114.0731			2
16							K	147.1128	74.0600	130.0863	65.5468			1

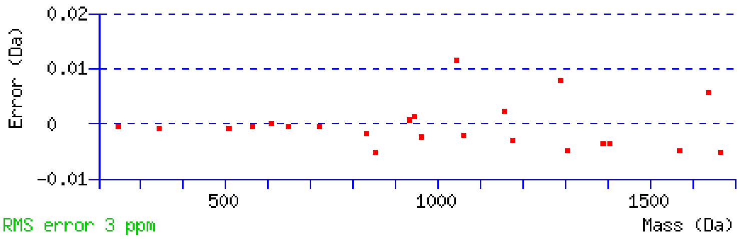

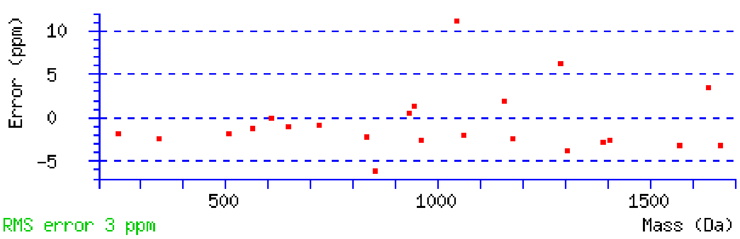

NCBI **BLAST** search of RPCFSALEVDETYVPK
(Parameters: blastp, nr protein database, expect=20000, no filter, PAM30)
Other BLAST web gateways

All matches to this query

Score	Mr(calc):	Delta	Sequence
87.2	1909.9244	-0.0056	RPCFSALEVDETYVPK

Mascot Search Results

Peptide View

MS/MS Fragmentation of **RPCFSALEVDETYVPK**
Found in **sp|P02768|ALBU_HUMAN**, Serum albumin OS=Homo sapiens GN=ALB PE=1 SV=2

Match to Query 9934: 1909.924168 from(955.969360,2+) intensity(11801648.0000) scans(15224) rtinseconds(2764) index(13102)
Title: 150818_TTSH_Patient_Plasma_84_Spectrum029624_scans__15224_RTINSECONDS=2764
Data file L:\\Ard_TTSH\\T1D\\T150818_TTSH_Patient_Plasma_84.mgf

Click mouse within plot area to zoom in by factor of two about that point
Or, Plot from 100 to 1900 Da Full range
Label all possible matches ○ Label matches used for scoring ⊙

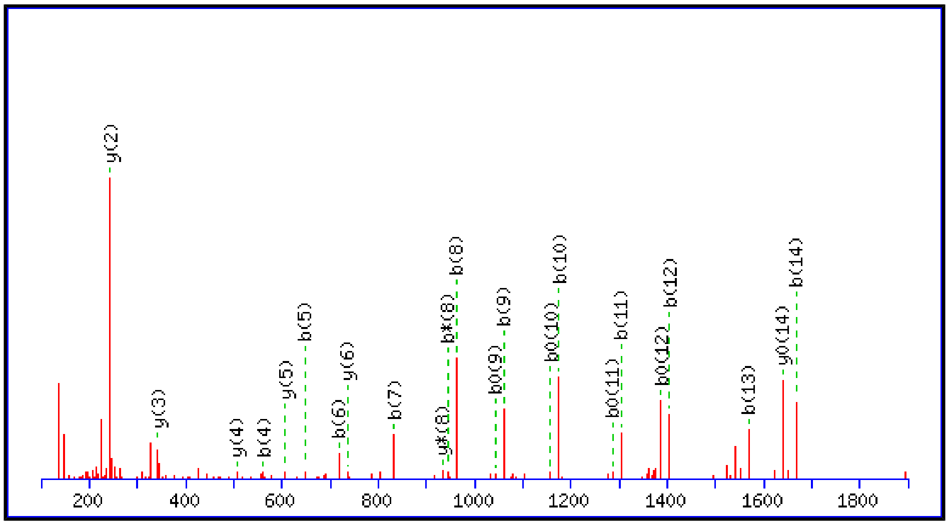

Monoisotopic mass of neutral peptide Mr(calc): 1909.9244
Variable modifications:
C3 : Carbamidomethyl (C)
Ions Score: 87 **Expect:** 7.1e-007
Matches : 23/164 fragment ions using 33 most intense peaks (help)

#	b	b^{++}	b*	b^{*++}	b^{0}	b^{0++}	Seq.	y	y^{++}	y*	y^{*++}	y^{0}	y^{0++}	#
1	157.1084	79.0578	140.0818	70.5446			R							16
2	254.1612	127.5842	237.1346	119.0709			P	1754.8306	877.9189	1737.8040	869.4056	1736.8200	868.9136	15
3	414.1918	207.5995	397.1653	199.0863			C	1657.7778	829.3925	1640.7513	820.8793	1639.7672	820.3873	14
4	561.2602	281.1337	544.2337	272.6205			F	1497.7472	749.3772	1480.7206	740.8639	1479.7366	740.3719	13
5	648.2922	324.6498	631.2657	316.1365	630.2817	315.6445	S	1350.6787	675.8430	1333.6522	667.3297	1332.6682	666.8377	12
6	719.3294	360.1683	702.3028	351.6550	701.3188	351.1630	A	1263.6467	632.3270	1246.6202	623.8137	1245.6361	623.3217	11
7	832.4134	416.7103	815.3869	408.1971	814.4029	407.7051	L	1192.6096	596.8084	1175.5830	588.2952	1174.5990	587.8032	10
8	961.4560	481.2316	944.4295	472.7184	943.4454	472.2264	E	1079.5255	540.2664	1062.4990	531.7531	1061.5150	531.2611	9
9	1060.5244	530.7659	1043.4979	522.2526	1042.5139	521.7606	V	950.4829	475.7451	933.4564	467.2318	932.4724	466.7398	8
10	1175.5514	588.2793	1158.5248	579.7660	1157.5408	579.2740	D	851.4145	426.2109	834.3880	417.6976	833.4040	417.2056	7
11	1304.5940	652.8006	1287.5674	644.2873	1286.5834	643.7953	E	736.3876	368.6974	719.3610	360.1842	718.3770	359.6921	6
12	1405.6416	703.3245	1388.6151	694.8112	1387.6311	694.3192	T	607.3450	304.1761	590.3184	295.6629	589.3344	295.1709	5
13	1568.7050	784.8561	1551.6784	776.3428	1550.6944	775.8508	Y	506.2973	253.6523	489.2708	245.1390			4
14	1667.7734	834.3903	1650.7468	825.8771	1649.7628	825.3850	V	343.2340	172.1206	326.2074	163.6074			3
15	1764.8261	882.9167	1747.7996	874.4034	1746.8156	873.9114	P	244.1656	122.5864	227.1390	114.0731			2
16							K	147.1128	74.0600	130.0863	65.5468			1

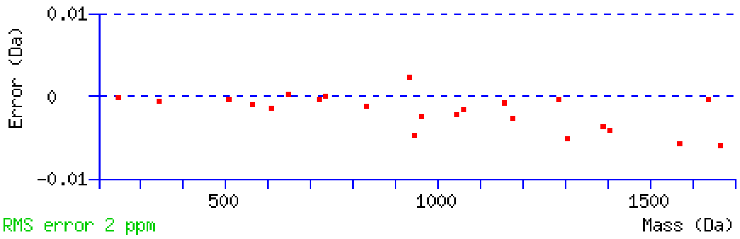

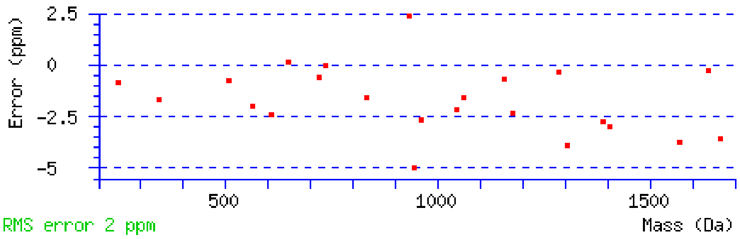

NCBI **BLAST** search of RPCFSALEVDETYVPK
(Parameters: blastp, nr protein database, expect=20000, no filter, PAM30)
Other BLAST web gateways

All matches to this query

Score	Mr(calc):	Delta	Sequence
87.2	1909.9244	-0.0002	RPCFSALEVDETYVPK

Mascot Search Results

Peptide View

MS/MS Fragmentation of **RPCFSALEVDETYVPK**
Found in **sp|P02768|ALBU_HUMAN**, Serum albumin OS=Homo sapiens GN=ALB PE=1 SV=2

Match to Query 8947: 1909.923568 from(955.969060,2+) intensity(5068370.5000) scans(12430) rtinseconds(2528) index(9443)
Title: 150808_TTSH_Patient_Plasma_37_Spectrum023965_scans__12430_RTINSECONDS=2528
Data file L:\\Ard_TTSH\\T1D\\T150808_TTSH_Patient_Plasma_37.mgf

Click mouse within plot area to zoom in by factor of two about that point
Or, [Plot from] 100 to 1900 Da [Full range]
Label all possible matches ○ Label matches used for scoring ⊙

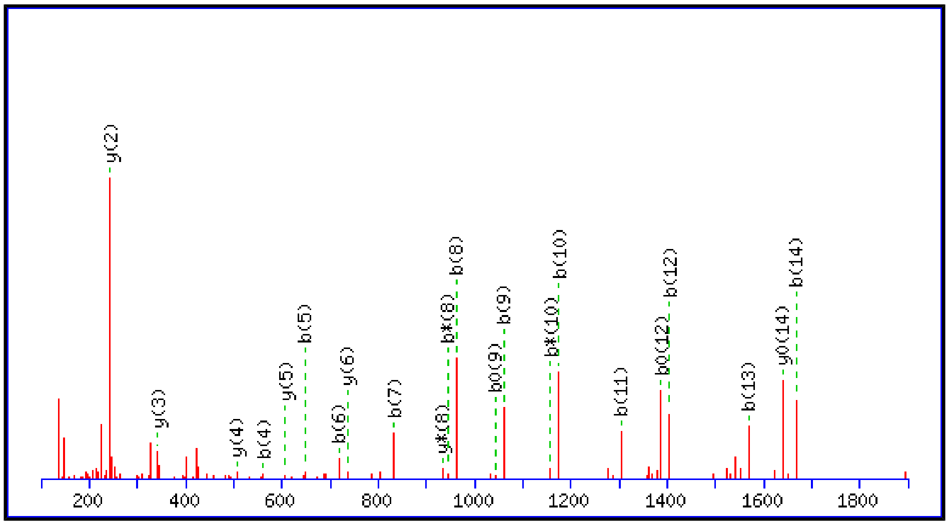

Monoisotopic mass of neutral peptide Mr(calc): 1909.9244
Variable modifications:
C3 : Carbamidomethyl (C)
Ions Score: 87 **Expect:** 7.4e-007
Matches : 22/164 fragment ions using 32 most intense peaks (help)

#	b	b^{++}	b*	b^{*++}	b^0	b^{0++}	Seq.	y	y^{++}	y*	y^{*++}	y^0	y^{0++}	#
1	157.1084	79.0578	140.0818	70.5446			R							16
2	254.1612	127.5842	237.1346	119.0709			P	1754.8306	877.9189	1737.8040	869.4056	1736.8200	868.9136	15
3	414.1918	207.5995	397.1653	199.0863			C	1657.7778	829.3925	1640.7513	820.8793	1639.7672	820.3873	14
4	***561.2602***	281.1337	544.2337	272.6205			F	1497.7472	749.3772	1480.7206	740.8639	1479.7366	740.3719	13
5	***648.2922***	324.6498	631.2657	316.1365	630.2817	315.6445	S	1350.6787	675.8430	1333.6522	667.3297	1332.6682	666.8377	12
6	***719.3294***	360.1683	702.3028	351.6550	701.3188	351.1630	A	1263.6467	632.3270	1246.6202	623.8137	1245.6361	623.3217	11
7	***832.4134***	416.7103	815.3869	408.1971	814.4029	407.7051	L	1192.6096	596.8084	1175.5830	588.2952	1174.5990	587.8032	10
8	***961.4560***	481.2316	944.4295	472.7184	943.4454	472.2264	E	1079.5255	540.2664	1062.4990	531.7531	1061.5150	531.2611	9
9	***1060.5244***	530.7659	1043.4979	522.2526	1042.5139	521.7606	V	950.4829	475.7451	933.4564	467.2318	932.4724	466.7398	8
10	***1175.5514***	588.2793	1158.5248	579.7660	1157.5408	579.2740	D	851.4145	426.2109	834.3880	417.6976	833.4040	417.2056	7
11	***1304.5940***	652.8006	1287.5674	644.2873	1286.5834	643.7953	E	**736.3876**	368.6974	719.3610	360.1842	718.3770	359.6921	6
12	***1405.6416***	703.3245	1388.6151	694.8112	1387.6311	694.3192	T	**607.3450**	304.1761	590.3184	295.6629	589.3344	295.1709	5
13	***1568.7050***	784.8561	1551.6784	776.3428	1550.6944	775.8508	Y	**506.2973**	253.6523	489.2708	245.1390			4
14	***1667.7734***	834.3903	1650.7468	825.8771	1649.7628	825.3850	V	**343.2340**	172.1206	326.2074	163.6074			3
15	1764.8261	882.9167	1747.7996	874.4034	1746.8156	873.9114	P	**244.1656**	122.5864	227.1390	114.0731			2
16							K	147.1128	74.0600	130.0863	65.5468			1

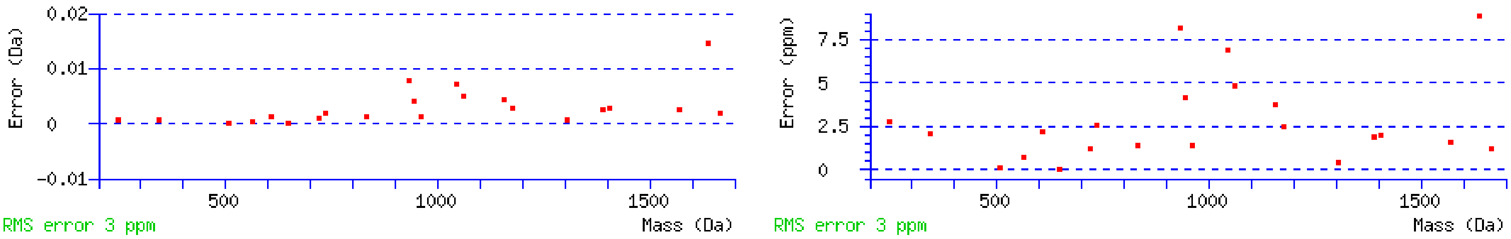

NCBI **BLAST** search of RPCFSALEVDETYVPK
(Parameters: blastp, nr protein database, expect=20000, no filter, PAM30)
Other BLAST web gateways

All matches to this query

Score	Mr(calc):	Delta	Sequence
87.0	1909.9244	-0.0008	RPCFSALEVDETYVPK

Mascot: http://www.matrixscience.com/

MATRIX SCIENCE Mascot Search Results

Peptide View

MS/MS Fragmentation of **RPCFSALEVDETYVPK**
Found in **sp|P02768|ALBU_HUMAN**, Serum albumin OS=Homo sapiens GN=ALB PE=1 SV=2

Match to Query 8698: 1909.925628 from(955.970090,2+) intensity(315650752.0000) scans(11209) rtinseconds(1986) index(9648)
Title: 150801_TTSH_Patient_Plasma_46_Spectrum026986_scans__11209_RTINSECONDS=1986
Data file L:\\Ard_TTSH\\T1D\\T150801_TTSH_Patient_Plasma_46.mgf

Click mouse within plot area to zoom in by factor of two about that point
Or, [Plot from] 100 to 1900 Da [Full range]
Label all possible matches ○ Label matches used for scoring ⊙

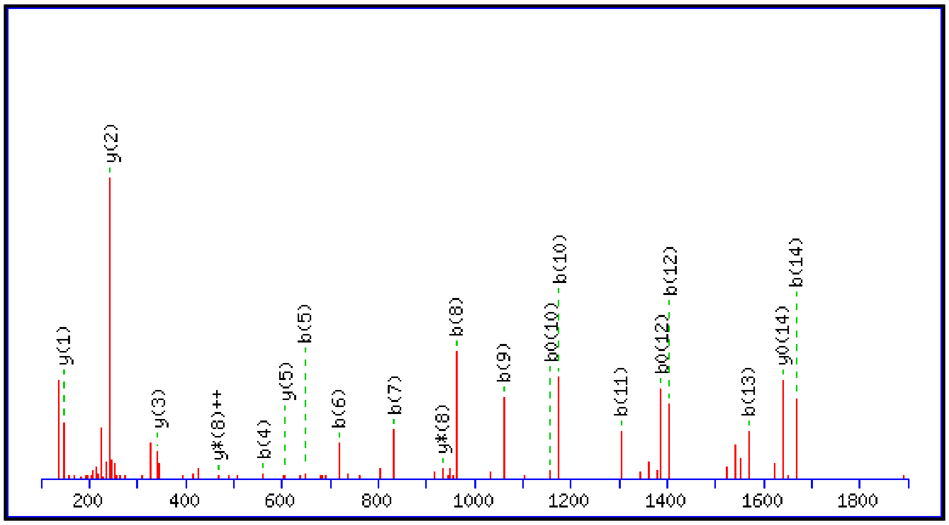

Monoisotopic mass of neutral peptide Mr(calc): 1909.9244
Variable modifications:
C3 : Carbamidomethyl (C)
Ions Score: 87 **Expect:** 7.7e-007
Matches : 20/164 fragment ions using 31 most intense peaks (help)

#	b	b^{++}	b*	b^{*++}	b^{0}	b^{0++}	Seq.	y	y^{++}	y*	y^{*++}	y^{0}	y^{0++}	#
1	157.1084	79.0578	140.0818	70.5446			R							16
2	254.1612	127.5842	237.1346	119.0709			P	1754.8306	877.9189	1737.8040	869.4056	1736.8200	868.9136	15
3	414.1918	207.5995	397.1653	199.0863			C	1657.7778	829.3925	1640.7513	820.8793	1639.7672	820.3873	14
4	***561.2602***	281.1337	544.2337	272.6205			F	1497.7472	749.3772	1480.7206	740.8639	1479.7366	740.3719	13
5	***648.2922***	324.6498	631.2657	316.1365	630.2817	315.6445	S	1350.6787	675.8430	1333.6522	667.3297	1332.6682	666.8377	12
6	***719.3294***	360.1683	702.3028	351.6550	701.3188	351.1630	A	1263.6467	632.3270	1246.6202	623.8137	1245.6361	623.3217	11
7	***832.4134***	416.7103	815.3869	408.1971	814.4029	407.7051	L	1192.6096	596.8084	1175.5830	588.2952	1174.5990	587.8032	10
8	***961.4560***	481.2316	944.4295	472.7184	943.4454	472.2264	E	1079.5255	540.2664	1062.4990	531.7531	1061.5150	531.2611	9
9	***1060.5244***	530.7659	1043.4979	522.2526	1042.5139	521.7606	V	950.4829	475.7451	933.4564	467.2318	932.4724	466.7398	8
10	***1175.5514***	588.2793	1158.5248	579.7660	1157.5408	579.2740	D	851.4145	426.2109	834.3880	417.6976	833.4040	417.2056	7
11	***1304.5940***	652.8006	1287.5674	644.2873	1286.5834	643.7953	E	736.3876	368.6974	719.3610	360.1842	718.3770	359.6921	6
12	***1405.6416***	703.3245	1388.6151	694.8112	1387.6311	694.3192	T	**607.3450**	304.1761	590.3184	295.6629	589.3344	295.1709	5
13	***1568.7050***	784.8561	1551.6784	776.3428	1550.6944	775.8508	Y	506.2973	253.6523	489.2708	245.1390			4
14	***1667.7734***	834.3903	1650.7468	825.8771	1649.7628	825.3850	V	**343.2340**	172.1206	326.2074	163.6074			3
15	1764.8261	882.9167	1747.7996	874.4034	1746.8156	873.9114	P	**244.1656**	122.5864	227.1390	114.0731			2
16							K	**147.1128**	74.0600	130.0863	65.5468			1

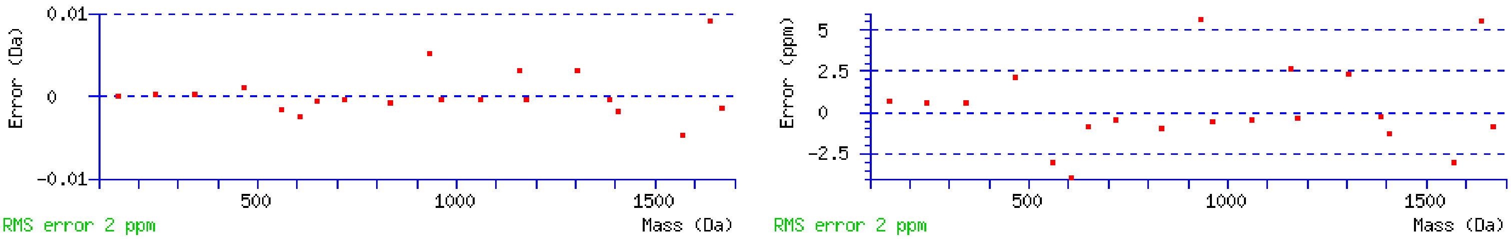

NCBI **BLAST** search of RPCFSALEVDETYVPK
(Parameters: blastp, nr protein database, expect=20000, no filter, PAM30)
Other BLAST web gateways

All matches to this query

Score	Mr(calc):	Delta	Sequence
86.9	1909.9244	0.0012	RPCFSALEVDETYVPK
0.4	1909.9396	-0.0140	QLYEMVLTYNFHQPK

Mascot: http://www.matrixscience.com/

Mascot Search Results

Peptide View

MS/MS Fragmentation of **RPCFSALEVDETYVPK**
Found in **sp|P02768|ALBU_HUMAN**, Serum albumin OS=Homo sapiens GN=ALB PE=1 SV=2

Match to Query 5449: 1909.911968 from(955.963260,2+) intensity(1611566592.0000) scans(8997) rtinseconds(1699) index(7434)
Title: 150825_TTSH_Patient_Plasma_60_Spectrum024310_scans__8997_RTINSECONDS=1699
Data file L:\\Ard_TTSH\\T1D\\T150825_TTSH_Patient_Plasma_60.mgf

Click mouse within plot area to zoom in by factor of two about that point
Or, [Plot from] 100 to 1700 Da [Full range]
Label all possible matches ○ Label matches used for scoring ⊙

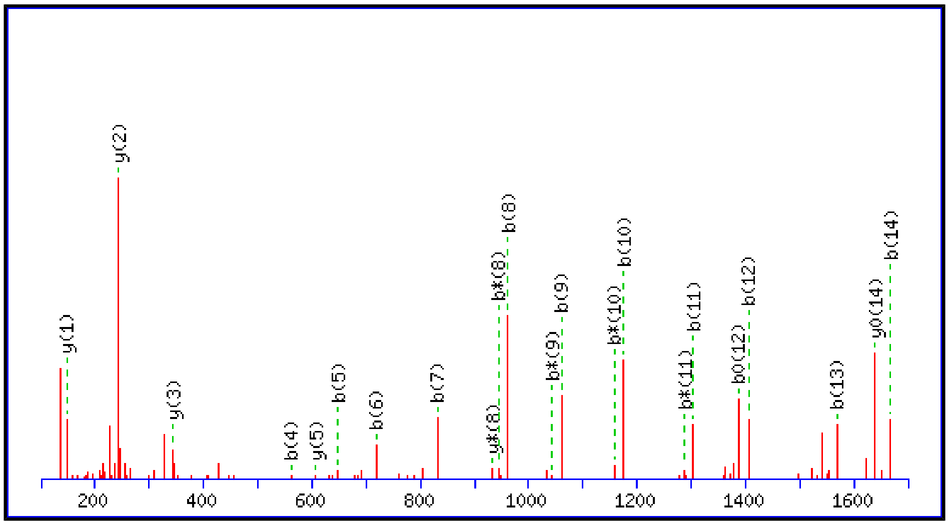

Monoisotopic mass of neutral peptide Mr(calc): 1909.9244
Variable modifications:
C3 : Carbamidomethyl (C)
Ions Score: 87 **Expect:** 7.2e-007
Matches : 22/164 fragment ions using 31 most intense peaks (help)

#	b	b^{++}	b*	b^{*++}	b^0	b^{0++}	Seq.	y	y^{++}	y*	y^{*++}	y^0	y^{0++}	#
1	157.1084	79.0578	140.0818	70.5446			R							16
2	254.1612	127.5842	237.1346	119.0709			P	1754.8306	877.9189	1737.8040	869.4056	1736.8200	868.9136	15
3	414.1918	207.5995	397.1653	199.0863			C	1657.7778	829.3925	1640.7513	820.8793	1639.7672	820.3873	14
4	***561.2602***	281.1337	544.2337	272.6205			F	1497.7472	749.3772	1480.7206	740.8639	1479.7366	740.3719	13
5	***648.2922***	324.6498	631.2657	316.1365	630.2817	315.6445	S	1350.6787	675.8430	1333.6522	667.3297	1332.6682	666.8377	12
6	***719.3294***	360.1683	702.3028	351.6550	701.3188	351.1630	A	1263.6467	632.3270	1246.6202	623.8137	1245.6361	623.3217	11
7	***832.4134***	416.7103	815.3869	408.1971	814.4029	407.7051	L	1192.6096	596.8084	1175.5830	588.2952	1174.5990	587.8032	10
8	***961.4560***	481.2316	944.4295	472.7184	943.4454	472.2264	E	1079.5255	540.2664	1062.4990	531.7531	1061.5150	531.2611	9
9	***1060.5244***	530.7659	1043.4979	522.2526	1042.5139	521.7606	V	950.4829	475.7451	933.4564	467.2318	932.4724	466.7398	8
10	***1175.5514***	588.2793	1158.5248	579.7660	1157.5408	579.2740	D	851.4145	426.2109	834.3880	417.6976	833.4040	417.2056	7
11	***1304.5940***	652.8006	1287.5674	644.2873	1286.5834	643.7953	E	736.3876	368.6974	719.3610	360.1842	718.3770	359.6921	6
12	***1405.6416***	703.3245	1388.6151	694.8112	1387.6311	694.3192	T	**607.3450**	304.1761	590.3184	295.6629	589.3344	295.1709	5
13	***1568.7050***	784.8561	1551.6784	776.3428	1550.6944	775.8508	Y	506.2973	253.6523	489.2708	245.1390			4
14	***1667.7734***	834.3903	1650.7468	825.8771	1649.7628	825.3850	V	**343.2340**	172.1206	326.2074	163.6074			3
15	1764.8261	882.9167	1747.7996	874.4034	1746.8156	873.9114	P	**244.1656**	122.5864	227.1390	114.0731			2
16							K	**147.1128**	74.0600	130.0863	65.5468			1

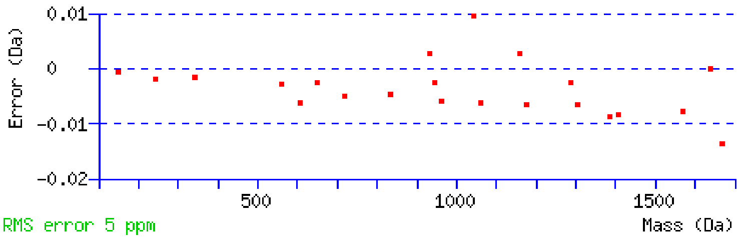

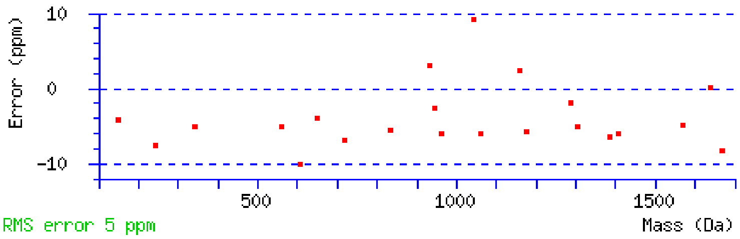

NCBI **BLAST** search of RPCFSALEVDETYVPK
(Parameters: blastp, nr protein database, expect=20000, no filter, PAM30)
Other BLAST web gateways

All matches to this query

Score	Mr(calc):	Delta	Sequence
86.8	1909.9244	-0.0124	RPCFSALEVDETYVPK

**Mascot:** http://www.matrixscience.com/

Mascot Search Results

Peptide View

MS/MS Fragmentation of **RPCFSALEVDETYVPK**
Found in **sp|P02768|ALBU_HUMAN**, Serum albumin OS=Homo sapiens GN=ALB PE=1 SV=2

Match to Query 8877: 1909.923068 from(955.968810,2+) intensity(16190750.0000) scans(12407) rtinseconds(2574) index(9293)
Title: 150808_TTSH_Patient_Plasma_07_Spectrum023319_scans__12407_RTINSECONDS=2574
Data file L:\\Ard_TTSH\\T1D\\T150808_TTSH_Patient_Plasma_07.mgf

Click mouse within plot area to zoom in by factor of two about that point
Or, Plot from 100 to 1900 Da Full range
Label all possible matches ○ Label matches used for scoring ⊙

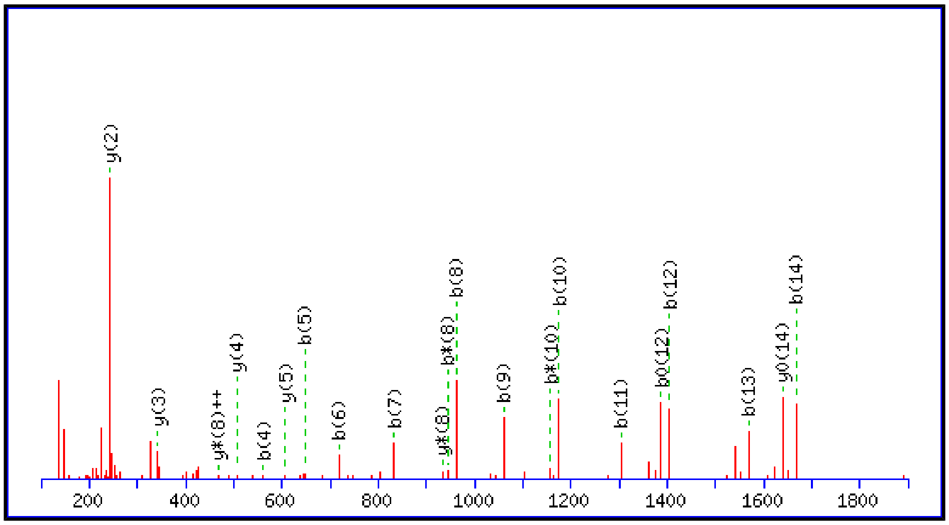


```
Monoisotopic mass of neutral peptide Mr(calc): 1909.9244
Variable modifications:
C3     : Carbamidomethyl (C)
Ions Score: 87  Expect: 8.2e-007
Matches : 21/164 fragment ions using 31 most intense peaks   (help)
```

#	b	b^{++}	b*	b^{*++}	b^0	b^{0++}	Seq.	y	y^{++}	y*	y^{*++}	y^0	y^{0++}	#
1	157.1084	79.0578	140.0818	70.5446			R							16
2	254.1612	127.5842	237.1346	119.0709			P	1754.8306	877.9189	1737.8040	869.4056	1736.8200	868.9136	15
3	414.1918	207.5995	397.1653	199.0863			C	1657.7778	829.3925	1640.7513	820.8793	1639.7672	820.3873	14
4	561.2602	281.1337	544.2337	272.6205			F	1497.7472	749.3772	1480.7206	740.8639	1479.7366	740.3719	13
5	648.2922	324.6498	631.2657	316.1365	630.2817	315.6445	S	1350.6787	675.8430	1333.6522	667.3297	1332.6682	666.8377	12
6	719.3294	360.1683	702.3028	351.6550	701.3188	351.1630	A	1263.6467	632.3270	1246.6202	623.8137	1245.6361	623.3217	11
7	832.4134	416.7103	815.3869	408.1971	814.4029	407.7051	L	1192.6096	596.8084	1175.5830	588.2952	1174.5990	587.8032	10
8	961.4560	481.2316	944.4295	472.7184	943.4454	472.2264	E	1079.5255	540.2664	1062.4990	531.7531	1061.5150	531.2611	9
9	1060.5244	530.7659	1043.4979	522.2526	1042.5139	521.7606	V	950.4829	475.7451	933.4564	467.2318	932.4724	466.7398	8
10	1175.5514	588.2793	1158.5248	579.7660	1157.5408	579.2740	D	851.4145	426.2109	834.3880	417.6976	833.4040	417.2056	7
11	1304.5940	652.8006	1287.5674	644.2873	1286.5834	643.7953	E	736.3876	368.6974	719.3610	360.1842	718.3770	359.6921	6
12	1405.6416	703.3245	1388.6151	694.8112	1387.6311	694.3192	T	607.3450	304.1761	590.3184	295.6629	589.3344	295.1709	5
13	1568.7050	784.8561	1551.6784	776.3428	1550.6944	775.8508	Y	506.2973	253.6523	489.2708	245.1390			4
14	1667.7734	834.3903	1650.7468	825.8771	1649.7628	825.3850	V	343.2340	172.1206	326.2074	163.6074			3
15	1764.8261	882.9167	1747.7996	874.4034	1746.8156	873.9114	P	244.1656	122.5864	227.1390	114.0731			2
16							K	147.1128	74.0600	130.0863	65.5468			1

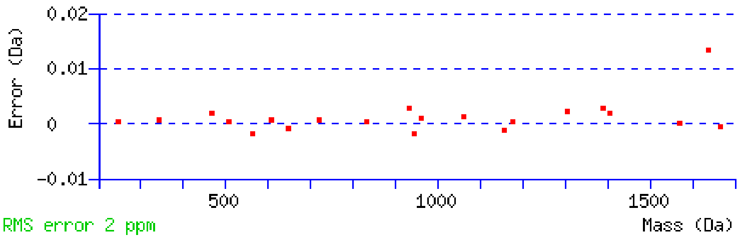

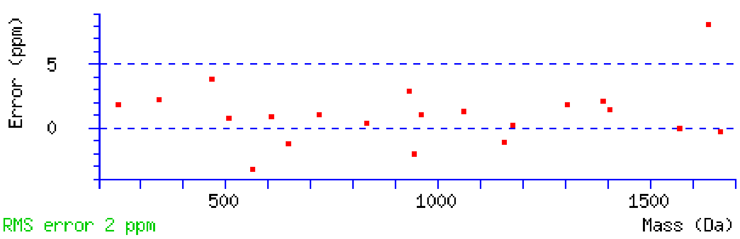

NCBI **BLAST** search of RPCFSALEVDETYVPK
(Parameters: blastp, nr protein database, expect=20000, no filter, PAM30)
Other BLAST web gateways

All matches to this query

Score	Mr(calc):	Delta	Sequence
86.6	1909.9244	-0.0013	RPCFSALEVDETYVPK

Mascot Search Results

Peptide View

MS/MS Fragmentation of **RPCFSALEVDETYVPK**
Found in **sp|P02768|ALBU_HUMAN**, Serum albumin OS=Homo sapiens GN=ALB PE=1 SV=2

Match to Query 5458: 1909.913188 from(955.963870,2+) intensity(1715585024.0000) scans(9158) rtinseconds(1730) index(7560)
Title: 150825_TTSH_Patient_Plasma_60_Spectrum024436_scans__9158_RTINSECONDS=1730
Data file L:\\Ard_TTSH\\T1D\\T150825_TTSH_Patient_Plasma_60.mgf

Click mouse within plot area to zoom in by factor of two about that point
Or, [Plot from] 100 to 1700 Da [Full range]
Label all possible matches ○ Label matches used for scoring ⊙

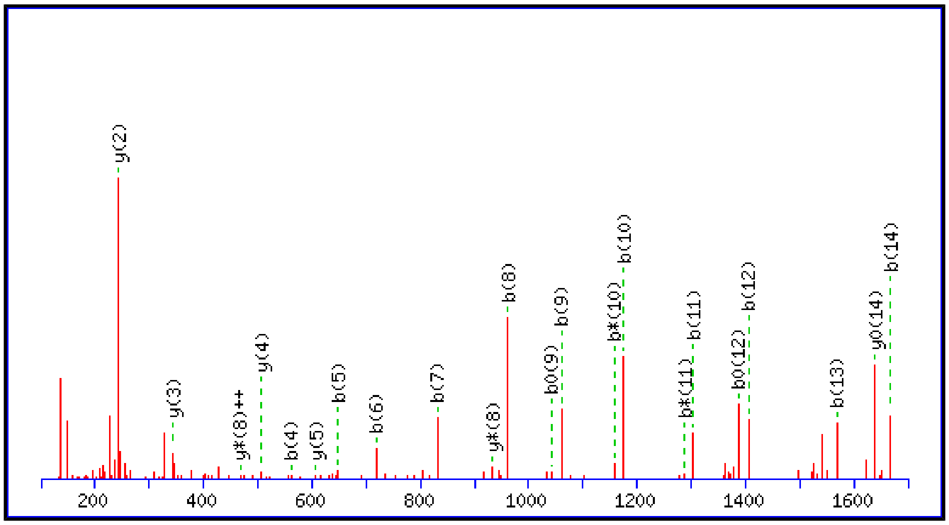

Monoisotopic mass of neutral peptide Mr(calc): 1909.9244
Variable modifications:
C3 : Carbamidomethyl (C)
Ions Score: 86 **Expect:** 7.9e-007
Matches : 22/164 fragment ions using 31 most intense peaks (help)

#	b	b^{++}	b*	b^{*++}	b^0	b^{0++}	Seq.	y	y^{++}	y*	y^{*++}	y^0	y^{0++}	#
1	157.1084	79.0578	140.0818	70.5446			R							16
2	254.1612	127.5842	237.1346	119.0709			P	1754.8306	877.9189	1737.8040	869.4056	1736.8200	868.9136	15
3	414.1918	207.5995	397.1653	199.0863			C	1657.7778	829.3925	1640.7513	820.8793	1639.7672	820.3873	14
4	***561.2602***	281.1337	544.2337	272.6205			F	1497.7472	749.3772	1480.7206	740.8639	1479.7366	740.3719	13
5	***648.2922***	324.6498	631.2657	316.1365	630.2817	315.6445	S	1350.6787	675.8430	1333.6522	667.3297	1332.6682	666.8377	12
6	***719.3294***	360.1683	702.3028	351.6550	701.3188	351.1630	A	1263.6467	632.3270	1246.6202	623.8137	1245.6361	623.3217	11
7	***832.4134***	416.7103	815.3869	408.1971	814.4029	407.7051	L	1192.6096	596.8084	1175.5830	588.2952	1174.5990	587.8032	10
8	***961.4560***	481.2316	944.4295	472.7184	943.4454	472.2264	E	1079.5255	540.2664	1062.4990	531.7531	1061.5150	531.2611	9
9	***1060.5244***	530.7659	1043.4979	522.2526	1042.5139	521.7606	V	950.4829	475.7451	933.4564	467.2318	932.4724	466.7398	8
10	***1175.5514***	588.2793	1158.5248	579.7660	1157.5408	579.2740	D	851.4145	426.2109	834.3880	417.6976	833.4040	417.2056	7
11	***1304.5940***	652.8006	1287.5674	644.2873	1286.5834	643.7953	E	736.3876	368.6974	719.3610	360.1842	718.3770	359.6921	6
12	***1405.6416***	703.3245	1388.6151	694.8112	1387.6311	694.3192	T	**607.3450**	304.1761	590.3184	295.6629	589.3344	295.1709	5
13	***1568.7050***	784.8561	1551.6784	776.3428	1550.6944	775.8508	Y	**506.2973**	253.6523	489.2708	245.1390			4
14	***1667.7734***	834.3903	1650.7468	825.8771	1649.7628	825.3850	V	**343.2340**	172.1206	326.2074	163.6074			3
15	1764.8261	882.9167	1747.7996	874.4034	1746.8156	873.9114	P	**244.1656**	122.5864	227.1390	114.0731			2
16							K	147.1128	74.0600	130.0863	65.5468			1

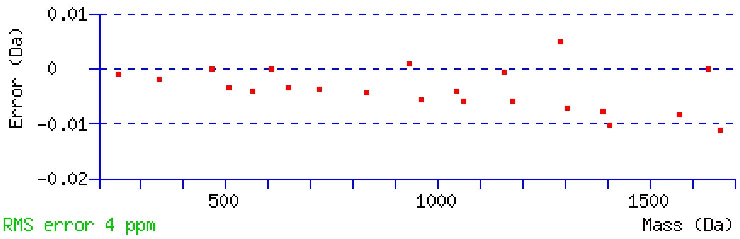

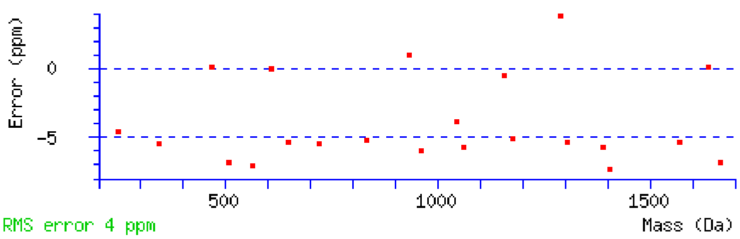

NCBI **BLAST** search of RPCFSALEVDETYVPK
(Parameters: blastp, nr protein database, expect=20000, no filter, PAM30)
Other BLAST web gateways

All matches to this query

Score	Mr(calc):	Delta	Sequence
86.4	1909.9244	-0.0112	RPCFSALEVDETYVPK

Mascot: http://www.matrixscience.com/

Mascot Search Results

Peptide View

MS/MS Fragmentation of **RPCFSALEVDETYVPK**
Found in **sp|P02768|ALBU_HUMAN**, Serum albumin OS=Homo sapiens GN=ALB PE=1 SV=2

Match to Query 9865: 1909.922828 from(955.968690,2+) intensity(3210293.0000) scans(13455) rtinseconds(2534) index(11155)
Title: 150808_TTSH_Patient_Plasma_32_Spectrum027558_scans__13455_RTINSECONDS=2534
Data file L:\\Ard_TTSH\\T1D\\T150808_TTSH_Patient_Plasma_32.mgf

Click mouse within plot area to zoom in by factor of two about that point
Or, Plot from 100 to 1700 Da Full range
Label all possible matches ○ Label matches used for scoring ⊙

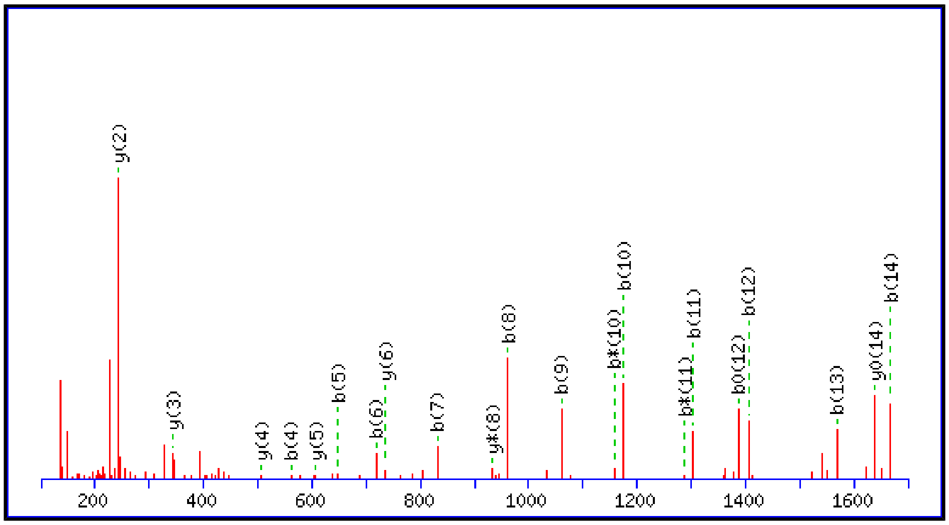

Monoisotopic mass of neutral peptide Mr(calc): 1909.9244
Variable modifications:
C3 : Carbamidomethyl (C)
Ions Score: 86 **Expect:** 9.1e-007
Matches : 21/164 fragment ions using 30 most intense peaks (help)

#	b	b^{++}	b*	b^{*++}	b^{0}	b^{0++}	Seq.	y	y^{++}	y*	y^{*++}	y^{0}	y^{0++}	#
1	157.1084	79.0578	140.0818	70.5446			R							16
2	254.1612	127.5842	237.1346	119.0709			P	1754.8306	877.9189	1737.8040	869.4056	1736.8200	868.9136	15
3	414.1918	207.5995	397.1653	199.0863			C	1657.7778	829.3925	1640.7513	820.8793	1639.7672	820.3873	14
4	***561.2602***	281.1337	544.2337	272.6205			F	1497.7472	749.3772	1480.7206	740.8639	1479.7366	740.3719	13
5	***648.2922***	324.6498	631.2657	316.1365	630.2817	315.6445	S	1350.6787	675.8430	1333.6522	667.3297	1332.6682	666.8377	12
6	***719.3294***	360.1683	702.3028	351.6550	701.3188	351.1630	A	1263.6467	632.3270	1246.6202	623.8137	1245.6361	623.3217	11
7	***832.4134***	416.7103	815.3869	408.1971	814.4029	407.7051	L	1192.6096	596.8084	1175.5830	588.2952	1174.5990	587.8032	10
8	***961.4560***	481.2316	944.4295	472.7184	943.4454	472.2264	E	1079.5255	540.2664	1062.4990	531.7531	1061.5150	531.2611	9
9	***1060.5244***	530.7659	1043.4979	522.2526	1042.5139	521.7606	V	950.4829	475.7451	933.4564	467.2318	932.4724	466.7398	8
10	***1175.5514***	588.2793	1158.5248	579.7660	1157.5408	579.2740	D	851.4145	426.2109	834.3880	417.6976	833.4040	417.2056	7
11	***1304.5940***	652.8006	1287.5674	644.2873	1286.5834	643.7953	E	**736.3876**	368.6974	719.3610	360.1842	718.3770	359.6921	6
12	***1405.6416***	703.3245	1388.6151	694.8112	1387.6311	694.3192	T	**607.3450**	304.1761	590.3184	295.6629	589.3344	295.1709	5
13	***1568.7050***	784.8561	1551.6784	776.3428	1550.6944	775.8508	Y	**506.2973**	253.6523	489.2708	245.1390			4
14	***1667.7734***	834.3903	1650.7468	825.8771	1649.7628	825.3850	V	**343.2340**	172.1206	326.2074	163.6074			3
15	1764.8261	882.9167	1747.7996	874.4034	1746.8156	873.9114	P	**244.1656**	122.5864	227.1390	114.0731			2
16							K	147.1128	74.0600	130.0863	65.5468			1

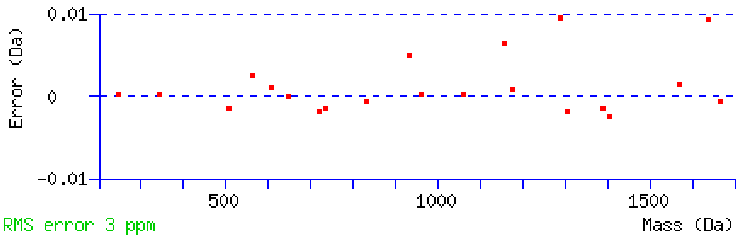

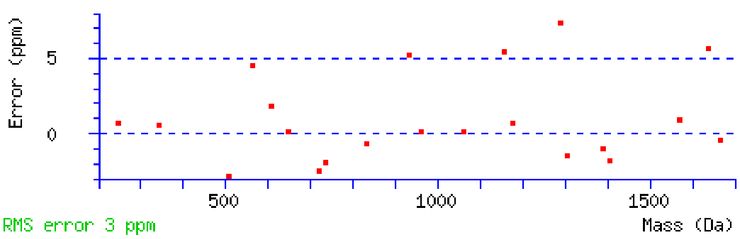

NCBI **BLAST** search of RPCFSALEVDETYVPK
(Parameters: blastp, nr protein database, expect=20000, no filter, PAM30)
Other BLAST web gateways

All matches to this query

Score	Mr(calc):	Delta	Sequence
86.1	1909.9244	-0.0016	RPCFSALEVDETYVPK

Mascot: http://www.matrixscience.com/

Mascot Search Results

Peptide View

MS/MS Fragmentation of **RPCFSALEVDETYVPK**
Found in **sp|P02768|ALBU_HUMAN**, Serum albumin OS=Homo sapiens GN=ALB PE=1 SV=2

Match to Query 8652: 1909.921608 from(955.968080,2+) intensity(374158240.0000) scans(12325) rtinseconds(2150) index(10562)
Title: 150818_TTSH_Patient_Plasma_36_Spectrum028818_scans__12325_RTINSECONDS=2150
Data file L:\\Ard_TTSH\\T1D\\T150818_TTSH_Patient_Plasma_36.mgf

Click mouse within plot area to zoom in by factor of two about that point
Or, Plot from 100 to 1700 Da Full range
Label all possible matches ○ Label matches used for scoring ⦿

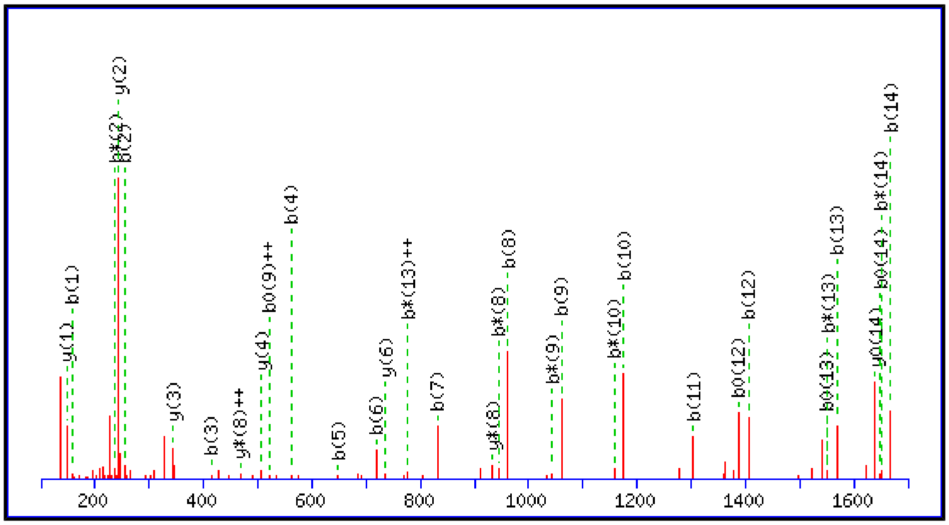

Monoisotopic mass of neutral peptide Mr(calc): 1909.9244
Variable modifications:
C3 : Carbamidomethyl (C)
Ions Score: 86 **Expect:** 9.5e-007
Matches : 33/164 fragment ions using 69 most intense peaks (help)

#	b	b^{++}	b*	b^{*++}	b^{0}	b^{0++}	Seq.	y	y^{++}	y*	y^{*++}	y^{0}	y^{0++}	#
1	157.1084	79.0578	140.0818	70.5446			R							16
2	254.1612	127.5842	237.1346	119.0709			P	1754.8306	877.9189	1737.8040	869.4056	1736.8200	868.9136	15
3	414.1918	207.5995	397.1653	199.0863			C	1657.7778	829.3925	1640.7513	820.8793	1639.7672	820.3873	14
4	561.2602	281.1337	544.2337	272.6205			F	1497.7472	749.3772	1480.7206	740.8639	1479.7366	740.3719	13
5	648.2922	324.6498	631.2657	316.1365	630.2817	315.6445	S	1350.6787	675.8430	1333.6522	667.3297	1332.6682	666.8377	12
6	719.3294	360.1683	702.3028	351.6550	701.3188	351.1630	A	1263.6467	632.3270	1246.6202	623.8137	1245.6361	623.3217	11
7	832.4134	416.7103	815.3869	408.1971	814.4029	407.7051	L	1192.6096	596.8084	1175.5830	588.2952	1174.5990	587.8032	10
8	961.4560	481.2316	944.4295	472.7184	943.4454	472.2264	E	1079.5255	540.2664	1062.4990	531.7531	1061.5150	531.2611	9
9	1060.5244	530.7659	1043.4979	522.2526	1042.5139	521.7606	V	950.4829	475.7451	933.4564	467.2318	932.4724	466.7398	8
10	1175.5514	588.2793	1158.5248	579.7660	1157.5408	579.2740	D	851.4145	426.2109	834.3880	417.6976	833.4040	417.2056	7
11	1304.5940	652.8006	1287.5674	644.2873	1286.5834	643.7953	E	736.3876	368.6974	719.3610	360.1842	718.3770	359.6921	6
12	1405.6416	703.3245	1388.6151	694.8112	1387.6311	694.3192	T	607.3450	304.1761	590.3184	295.6629	589.3344	295.1709	5
13	1568.7050	784.8561	1551.6784	776.3428	1550.6944	775.8508	Y	506.2973	253.6523	489.2708	245.1390			4
14	1667.7734	834.3903	1650.7468	825.8771	1649.7628	825.3850	V	343.2340	172.1206	326.2074	163.6074			3
15	1764.8261	882.9167	1747.7996	874.4034	1746.8156	873.9114	P	244.1656	122.5864	227.1390	114.0731			2
16							K	147.1128	74.0600	130.0863	65.5468			1

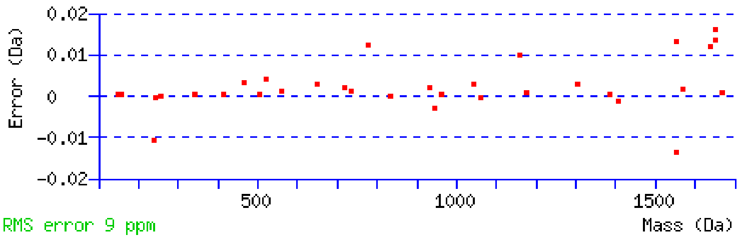

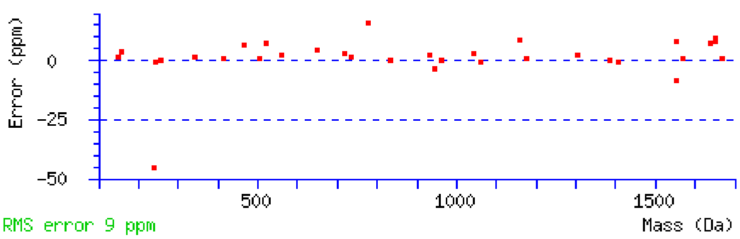

NCBI **BLAST** search of RPCFSALEVDETYVPK
(Parameters: blastp, nr protein database, expect=20000, no filter, PAM30)
Other BLAST web gateways

All matches to this query

Score	Mr(calc):	Delta	Sequence
86.0	1909.9244	-0.0028	RPCFSALEVDETYVPK

Mascot Search Results

Peptide View

MS/MS Fragmentation of **RPCFSALEVDETYVPK**
Found in **sp|P02768|ALBU_HUMAN**, Serum albumin OS=Homo sapiens GN=ALB PE=1 SV=2

Match to Query 8583: 1909.922588 from(955.968570,2+) intensity(590127.3125) scans(11666) rtinseconds(2488) index(8859)
Title: 150808_TTSH_Patient_Plasma_15_Spectrum022289_scans__11666_RTINSECONDS=2488
Data file L:\\Ard_TTSH\\T1D\\T150808_TTSH_Patient_Plasma_15.mgf

Click mouse within plot area to zoom in by factor of two about that point
Or, Plot from 100 to 1700 Da Full range
Label all possible matches ○ Label matches used for scoring ⊙

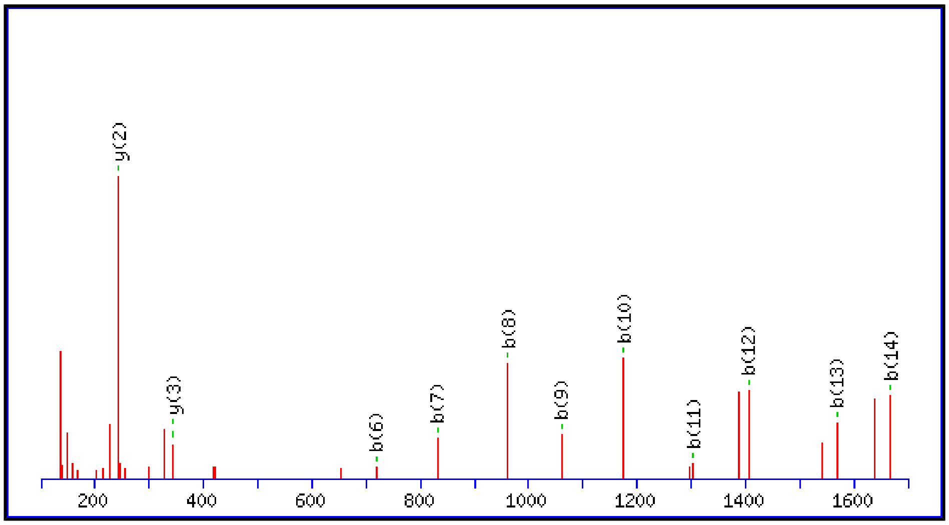

Monoisotopic mass of neutral peptide Mr(calc): 1909.9244
Variable modifications:
C3 : Carbamidomethyl (C)
Ions Score: 86 **Expect:** 9.6e-007
Matches : 11/164 fragment ions using 12 most intense peaks (help)

#	b	b^{++}	b*	b^{*++}	b^0	b^{0++}	Seq.	y	y^{++}	y*	y^{*++}	y^0	y^{0++}	#
1	157.1084	79.0578	140.0818	70.5446			R							16
2	254.1612	127.5842	237.1346	119.0709			P	1754.8306	877.9189	1737.8040	869.4056	1736.8200	868.9136	15
3	414.1918	207.5995	397.1653	199.0863			C	1657.7778	829.3925	1640.7513	820.8793	1639.7672	820.3873	14
4	561.2602	281.1337	544.2337	272.6205			F	1497.7472	749.3772	1480.7206	740.8639	1479.7366	740.3719	13
5	648.2922	324.6498	631.2657	316.1365	630.2817	315.6445	S	1350.6787	675.8430	1333.6522	667.3297	1332.6682	666.8377	12
6	***719.3294***	360.1683	702.3028	351.6550	701.3188	351.1630	A	1263.6467	632.3270	1246.6202	623.8137	1245.6361	623.3217	11
7	***832.4134***	416.7103	815.3869	408.1971	814.4029	407.7051	L	1192.6096	596.8084	1175.5830	588.2952	1174.5990	587.8032	10
8	***961.4560***	481.2316	944.4295	472.7184	943.4454	472.2264	E	1079.5255	540.2664	1062.4990	531.7531	1061.5150	531.2611	9
9	***1060.5244***	530.7659	1043.4979	522.2526	1042.5139	521.7606	V	950.4829	475.7451	933.4564	467.2318	932.4724	466.7398	8
10	***1175.5514***	588.2793	1158.5248	579.7660	1157.5408	579.2740	D	851.4145	426.2109	834.3880	417.6976	833.4040	417.2056	7
11	***1304.5940***	652.8006	1287.5674	644.2873	1286.5834	643.7953	E	736.3876	368.6974	719.3610	360.1842	718.3770	359.6921	6
12	***1405.6416***	703.3245	1388.6151	694.8112	1387.6311	694.3192	T	607.3450	304.1761	590.3184	295.6629	589.3344	295.1709	5
13	***1568.7050***	784.8561	1551.6784	776.3428	1550.6944	775.8508	Y	506.2973	253.6523	489.2708	245.1390			4
14	***1667.7734***	834.3903	1650.7468	825.8771	1649.7628	825.3850	V	**343.2340**	172.1206	326.2074	163.6074			3
15	1764.8261	882.9167	1747.7996	874.4034	1746.8156	873.9114	P	**244.1656**	122.5864	227.1390	114.0731			2
16							K	147.1128	74.0600	130.0863	65.5468			1

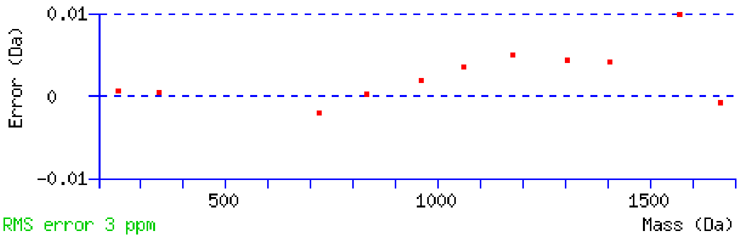

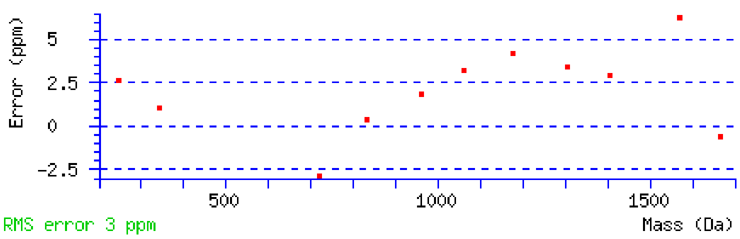

NCBI **BLAST** search of RPCFSALEVDETYVPK
(Parameters: blastp, nr protein database, expect=20000, no filter, PAM30)
Other BLAST web gateways

All matches to this query

Score	Mr(calc):	Delta	Sequence
85.8	1909.9244	-0.0018	RPCFSALEVDETYVPK

MATRIX SCIENCE Mascot Search Results

Peptide View

MS/MS Fragmentation of **RPCFSALEVDETYVPK**
Found in **sp|P02768|ALBU_HUMAN**, Serum albumin OS=Homo sapiens GN=ALB PE=1 SV=2

Match to Query 5510: 1909.918056 from(478.486790,4+) intensity(22104204.0000) scans(9875) rtinseconds(1864) index(8123)
Title: 150825_TTSH_Patient_Plasma_60_Spectrum024999_scans__9875_RTINSECONDS=1864
Data file L:\\Ard_TTSH\\T1D\\T150825_TTSH_Patient_Plasma_60.mgf

Click mouse within plot area to zoom in by factor of two about that point
Or, Plot from 100 to 1400 Da Full range
Label all possible matches ○ Label matches used for scoring ⦿

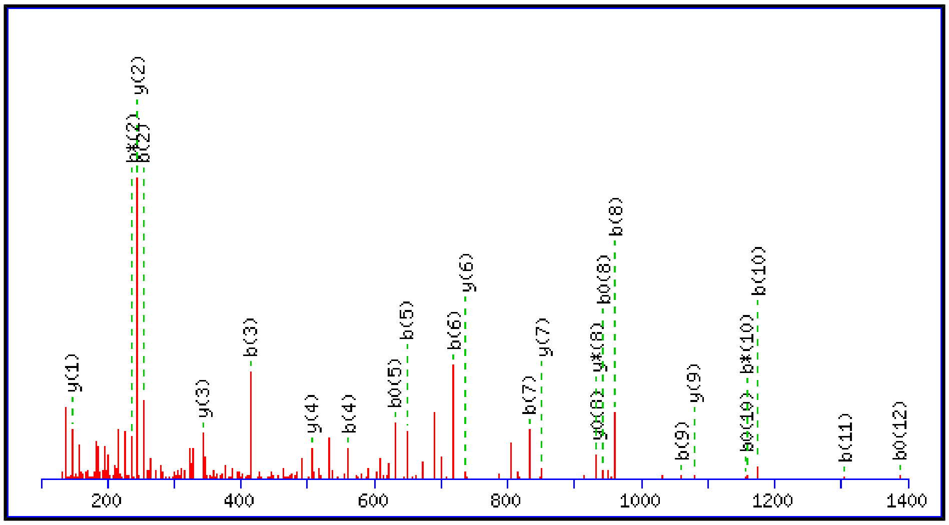

Monoisotopic mass of neutral peptide Mr(calc): 1909.9244
Variable modifications:
C3 : Carbamidomethyl (C)
Ions Score: 85 **Expect:** 1e-006
Matches : 25/164 fragment ions using 34 most intense peaks (help)

#	b	b^{++}	b*	b^{*++}	b^0	b^{0++}	Seq.	y	y^{++}	y*	y^{*++}	y^0	y^{0++}	#
1	157.1084	79.0578	140.0818	70.5446			R							16
2	254.1612	127.5842	237.1346	119.0709			P	1754.8306	877.9189	1737.8040	869.4056	1736.8200	868.9136	15
3	414.1918	207.5995	397.1653	199.0863			C	1657.7778	829.3925	1640.7513	820.8793	1639.7672	820.3873	14
4	561.2602	281.1337	544.2337	272.6205			F	1497.7472	749.3772	1480.7206	740.8639	1479.7366	740.3719	13
5	648.2922	324.6498	631.2657	316.1365	630.2817	315.6445	S	1350.6787	675.8430	1333.6522	667.3297	1332.6682	666.8377	12
6	719.3294	360.1683	702.3028	351.6550	701.3188	351.1630	A	1263.6467	632.3270	1246.6202	623.8137	1245.6361	623.3217	11
7	832.4134	416.7103	815.3869	408.1971	814.4029	407.7051	L	1192.6096	596.8084	1175.5830	588.2952	1174.5990	587.8032	10
8	961.4560	481.2316	944.4295	472.7184	943.4454	472.2264	E	1079.5255	540.2664	1062.4990	531.7531	1061.5150	531.2611	9
9	1060.5244	530.7659	1043.4979	522.2526	1042.5139	521.7606	V	950.4829	475.7451	933.4564	467.2318	932.4724	466.7398	8
10	1175.5514	588.2793	1158.5248	579.7660	1157.5408	579.2740	D	851.4145	426.2109	834.3880	417.6976	833.4040	417.2056	7
11	1304.5940	652.8006	1287.5674	644.2873	1286.5834	643.7953	E	736.3876	368.6974	719.3610	360.1842	718.3770	359.6921	6
12	1405.6416	703.3245	1388.6151	694.8112	1387.6311	694.3192	T	607.3450	304.1761	590.3184	295.6629	589.3344	295.1709	5
13	1568.7050	784.8561	1551.6784	776.3428	1550.6944	775.8508	Y	506.2973	253.6523	489.2708	245.1390			4
14	1667.7734	834.3903	1650.7468	825.8771	1649.7628	825.3850	V	343.2340	172.1206	326.2074	163.6074			3
15	1764.8261	882.9167	1747.7996	874.4034	1746.8156	873.9114	P	244.1656	122.5864	227.1390	114.0731			2
16							K	147.1128	74.0600	130.0863	65.5468			1

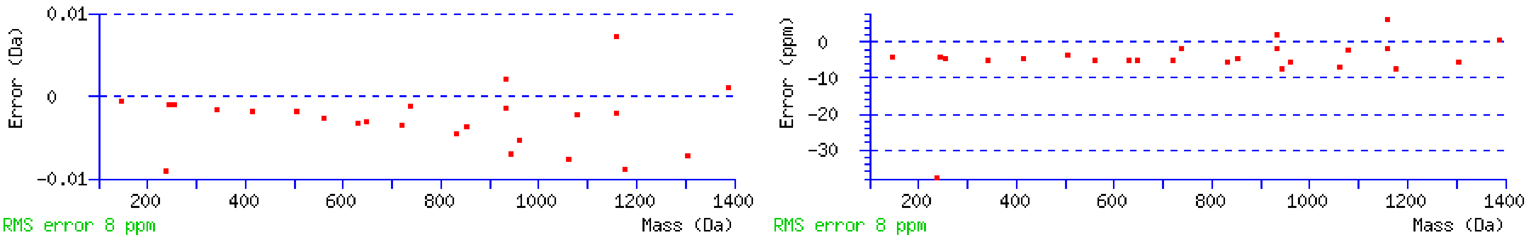

NCBI **BLAST** search of RPCFSALEVDETYVPK
(Parameters: blastp, nr protein database, expect=20000, no filter, PAM30)
Other BLAST web gateways

All matches to this query

Score	Mr(calc):	Delta	Sequence
85.5	1909.9244	-0.0063	RPCFSALEVDETYVPK

Mascot Search Results

Peptide View

MS/MS Fragmentation of **RPCFSALEVDETYVPK**
Found in **sp|P02768|ALBU_HUMAN**, Serum albumin OS=Homo sapiens GN=ALB PE=1 SV=2

Match to Query 9521: 1909.923068 from(955.968810,2+) intensity(9679715.0000) scans(11543) rtinseconds(2097) index(9943)
Title: 150801_TTSH_Patient_Plasma_50_Spectrum026734_scans__11543_RTINSECONDS=2097
Data file L:\\Ard_TTSH\\T1D\\T150801_TTSH_Patient_Plasma_50.mgf

Click mouse within plot area to zoom in by factor of two about that point
Or, Plot from 100 to 1900 Da Full range
Label all possible matches ○ Label matches used for scoring ⊙

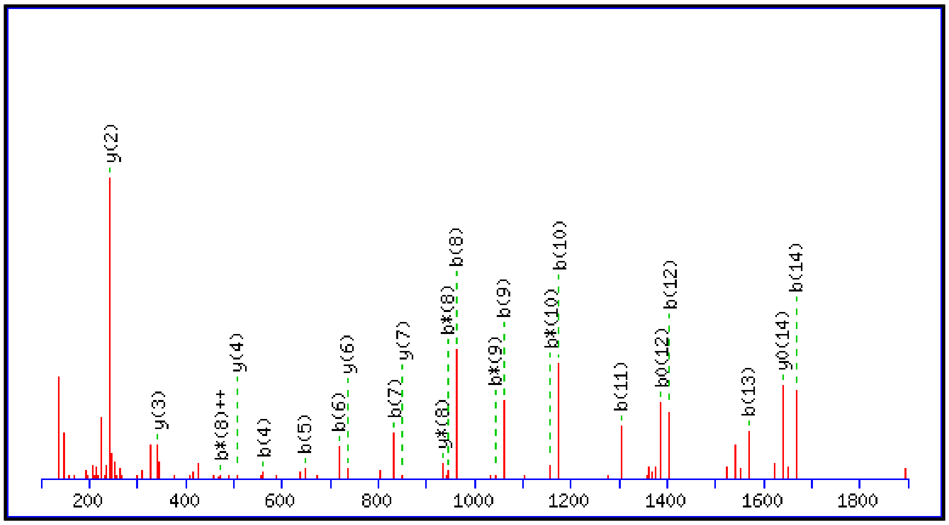


```
Monoisotopic mass of neutral peptide Mr(calc): 1909.9244
Variable modifications:
C3     : Carbamidomethyl (C)
Ions Score: 85  Expect: 1.2e-006
Matches : 23/164 fragment ions using 34 most intense peaks   (help)
```

#	b	b^{++}	b*	b^{*++}	b^0	b^{0++}	Seq.	y	y^{++}	y*	y^{*++}	y^0	y^{0++}	#
1	157.1084	79.0578	140.0818	70.5446			R							16
2	254.1612	127.5842	237.1346	119.0709			P	1754.8306	877.9189	1737.8040	869.4056	1736.8200	868.9136	15
3	414.1918	207.5995	397.1653	199.0863			C	1657.7778	829.3925	1640.7513	820.8793	1639.7672	820.3873	14
4	***561.2602***	281.1337	544.2337	272.6205			F	1497.7472	749.3772	1480.7206	740.8639	1479.7366	740.3719	13
5	***648.2922***	324.6498	631.2657	316.1365	630.2817	315.6445	S	1350.6787	675.8430	1333.6522	667.3297	1332.6682	666.8377	12
6	***719.3294***	360.1683	702.3028	351.6550	701.3188	351.1630	A	1263.6467	632.3270	1246.6202	623.8137	1245.6361	623.3217	11
7	***832.4134***	416.7103	815.3869	408.1971	814.4029	407.7051	L	1192.6096	596.8084	1175.5830	588.2952	1174.5990	587.8032	10
8	***961.4560***	481.2316	944.4295	472.7184	943.4454	472.2264	E	1079.5255	540.2664	1062.4990	531.7531	1061.5150	531.2611	9
9	***1060.5244***	530.7659	1043.4979	522.2526	1042.5139	521.7606	V	950.4829	475.7451	933.4564	467.2318	932.4724	466.7398	8
10	***1175.5514***	588.2793	1158.5248	579.7660	1157.5408	579.2740	D	**851.4145**	426.2109	834.3880	417.6976	833.4040	417.2056	7
11	***1304.5940***	652.8006	1287.5674	644.2873	1286.5834	643.7953	E	**736.3876**	368.6974	719.3610	360.1842	718.3770	359.6921	6
12	***1405.6416***	703.3245	1388.6151	694.8112	1387.6311	694.3192	T	607.3450	304.1761	590.3184	295.6629	589.3344	295.1709	5
13	***1568.7050***	784.8561	1551.6784	776.3428	1550.6944	775.8508	Y	**506.2973**	253.6523	489.2708	245.1390			4
14	***1667.7734***	834.3903	1650.7468	825.8771	1649.7628	825.3850	V	**343.2340**	172.1206	326.2074	163.6074			3
15	1764.8261	882.9167	1747.7996	874.4034	1746.8156	873.9114	P	**244.1656**	122.5864	227.1390	114.0731			2
16							K	147.1128	74.0600	130.0863	65.5468			1

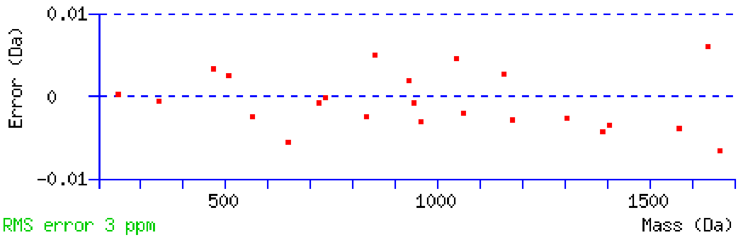

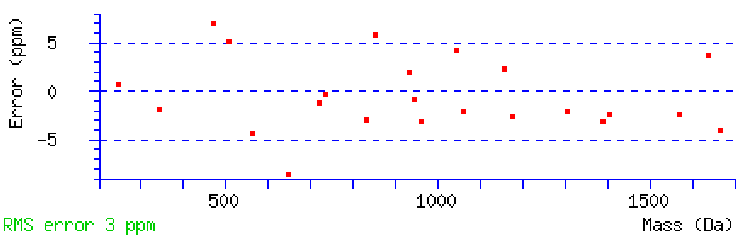

NCBI **BLAST** search of RPCFSALEVDETYVPK
(Parameters: blastp, nr protein database, expect=20000, no filter, PAM30)
Other BLAST web gateways

All matches to this query

Score	Mr(calc):	Delta	Sequence
85.0	1909.9244	-0.0013	RPCFSALEVDETYVPK

**Mascot:** http://www.matrixscience.com/

Mascot Search Results

Peptide View

MS/MS Fragmentation of **RPCFSALEVDETYVPK**
Found in **sp|P02768|ALBU_HUMAN**, Serum albumin OS=Homo sapiens GN=ALB PE=1 SV=2

Match to Query 9578: 1909.923068 from(955.968810,2+) intensity(1061850624.0000) scans(13395) rtinseconds(2369) index(11621)
Title: 150808_TTSH_Patient_Plasma_69_Spectrum029444_scans__13395_RTINSECONDS=2369
Data file L:\\Ard_TTSH\\T1D\\T150808_TTSH_Patient_Plasma_69.mgf

Click mouse within plot area to zoom in by factor of two about that point
Or, Plot from 100 to 1900 Da Full range
Label all possible matches ○ Label matches used for scoring ⊙

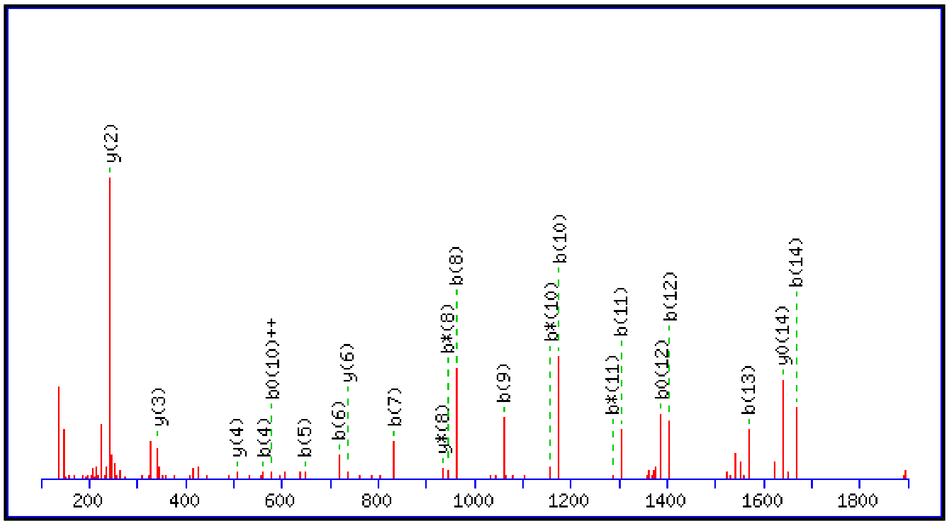


```
Monoisotopic mass of neutral peptide Mr(calc): 1909.9244
Variable modifications:
C3     : Carbamidomethyl (C)
Ions Score: 85  Expect: 1.3e-006
Matches : 22/164 fragment ions using 33 most intense peaks   (help)
```

#	b	b^{++}	b*	b^{*++}	b^0	b^{0++}	Seq.	y	y^{++}	y*	y^{*++}	y^0	y^{0++}	#
1	157.1084	79.0578	140.0818	70.5446			R							16
2	254.1612	127.5842	237.1346	119.0709			P	1754.8306	877.9189	1737.8040	869.4056	1736.8200	868.9136	15
3	414.1918	207.5995	397.1653	199.0863			C	1657.7778	829.3925	1640.7513	820.8793	1639.7672	820.3873	14
4	***561.2602***	281.1337	544.2337	272.6205			F	1497.7472	749.3772	1480.7206	740.8639	1479.7366	740.3719	13
5	***648.2922***	324.6498	631.2657	316.1365	630.2817	315.6445	S	1350.6787	675.8430	1333.6522	667.3297	1332.6682	666.8377	12
6	***719.3294***	360.1683	702.3028	351.6550	701.3188	351.1630	A	1263.6467	632.3270	1246.6202	623.8137	1245.6361	623.3217	11
7	***832.4134***	416.7103	815.3869	408.1971	814.4029	407.7051	L	1192.6096	596.8084	1175.5830	588.2952	1174.5990	587.8032	10
8	***961.4560***	481.2316	944.4295	472.7184	943.4454	472.2264	E	1079.5255	540.2664	1062.4990	531.7531	1061.5150	531.2611	9
9	***1060.5244***	530.7659	1043.4979	522.2526	1042.5139	521.7606	V	950.4829	475.7451	933.4564	467.2318	932.4724	466.7398	8
10	***1175.5514***	588.2793	1158.5248	579.7660	1157.5408	579.2740	D	851.4145	426.2109	834.3880	417.6976	833.4040	417.2056	7
11	***1304.5940***	652.8006	1287.5674	644.2873	1286.5834	643.7953	E	**736.3876**	368.6974	719.3610	360.1842	718.3770	359.6921	6
12	***1405.6416***	703.3245	1388.6151	694.8112	1387.6311	694.3192	T	607.3450	304.1761	590.3184	295.6629	589.3344	295.1709	5
13	***1568.7050***	784.8561	1551.6784	776.3428	1550.6944	775.8508	Y	**506.2973**	253.6523	489.2708	245.1390			4
14	***1667.7734***	834.3903	1650.7468	825.8771	1649.7628	825.3850	V	**343.2340**	172.1206	326.2074	163.6074			3
15	1764.8261	882.9167	1747.7996	874.4034	1746.8156	873.9114	P	**244.1656**	122.5864	227.1390	114.0731			2
16							K	147.1128	74.0600	130.0863	65.5468			1

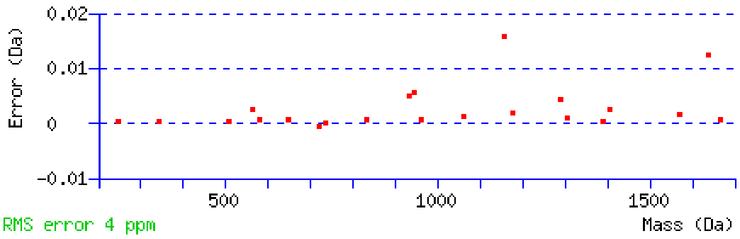

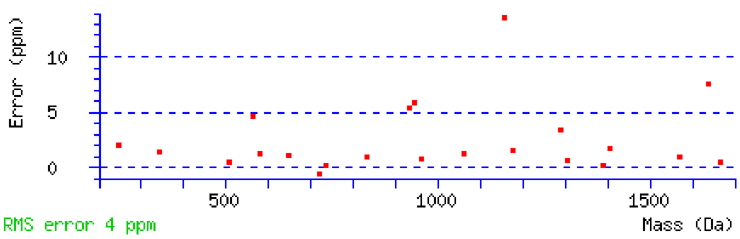

NCBI **BLAST** search of RPCFSALEVDETYVPK
(Parameters: blastp, nr protein database, expect=20000, no filter, PAM30)
Other BLAST web gateways

All matches to this query

Score	Mr(calc):	Delta	Sequence
84.7	1909.9244	-0.0013	RPCFSALEVDETYVPK

**Mascot:** http://www.matrixscience.com/

Mascot Search Results

Peptide View

MS/MS Fragmentation of **RPCFSALEVDETYVPK**
Found in **sp|P02768|ALBU_HUMAN**, Serum albumin OS=Homo sapiens GN=ALB PE=1 SV=2

Match to Query 9014: 1909.923188 from(955.968870,2+) intensity(476162144.0000) scans(11293) rtinseconds(1995) index(9798)
Title: 150801_TTSH_Patient_Plasma_49_Spectrum027300_scans__11293_RTINSECONDS=1995
Data file L:\\Ard_TTSH\\T1D\\T150801_TTSH_Patient_Plasma_49.mgf

Click mouse within plot area to zoom in by factor of two about that point
Or, Plot from 100 to 1900 Da Full range
Label all possible matches ○ Label matches used for scoring ⦿

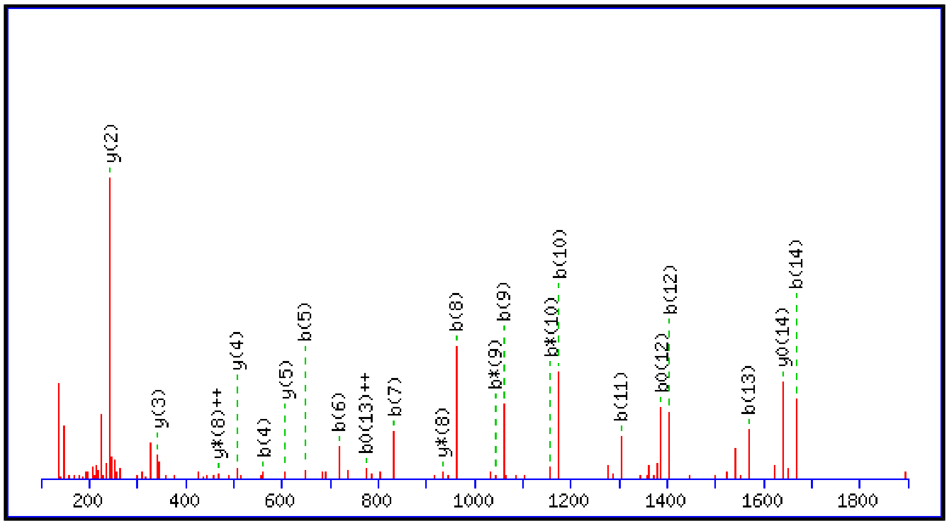

Monoisotopic mass of neutral peptide Mr(calc): 1909.9244
Variable modifications:
C3 : Carbamidomethyl (C)
Ions Score: 85 **Expect:** 1.3e-006
Matches : 22/164 fragment ions using 33 most intense peaks (help)

#	b	b++	b*	b*++	b0	b0++	Seq.	y	y++	y*	y*++	y0	y0++	#
1	157.1084	79.0578	140.0818	70.5446			R							16
2	254.1612	127.5842	237.1346	119.0709			P	1754.8306	877.9189	1737.8040	869.4056	1736.8200	868.9136	15
3	414.1918	207.5995	397.1653	199.0863			C	1657.7778	829.3925	1640.7513	820.8793	1639.7672	820.3873	14
4	561.2602	281.1337	544.2337	272.6205			F	1497.7472	749.3772	1480.7206	740.8639	1479.7366	740.3719	13
5	648.2922	324.6498	631.2657	316.1365	630.2817	315.6445	S	1350.6787	675.8430	1333.6522	667.3297	1332.6682	666.8377	12
6	719.3294	360.1683	702.3028	351.6550	701.3188	351.1630	A	1263.6467	632.3270	1246.6202	623.8137	1245.6361	623.3217	11
7	832.4134	416.7103	815.3869	408.1971	814.4029	407.7051	L	1192.6096	596.8084	1175.5830	588.2952	1174.5990	587.8032	10
8	961.4560	481.2316	944.4295	472.7184	943.4454	472.2264	E	1079.5255	540.2664	1062.4990	531.7531	1061.5150	531.2611	9
9	1060.5244	530.7659	1043.4979	522.2526	1042.5139	521.7606	V	950.4829	475.7451	933.4564	467.2318	932.4724	466.7398	8
10	1175.5514	588.2793	1158.5248	579.7660	1157.5408	579.2740	D	851.4145	426.2109	834.3880	417.6976	833.4040	417.2056	7
11	1304.5940	652.8006	1287.5674	644.2873	1286.5834	643.7953	E	736.3876	368.6974	719.3610	360.1842	718.3770	359.6921	6
12	1405.6416	703.3245	1388.6151	694.8112	1387.6311	694.3192	T	607.3450	304.1761	590.3184	295.6629	589.3344	295.1709	5
13	1568.7050	784.8561	1551.6784	776.3428	1550.6944	775.8508	Y	506.2973	253.6523	489.2708	245.1390			4
14	1667.7734	834.3903	1650.7468	825.8771	1649.7628	825.3850	V	343.2340	172.1206	326.2074	163.6074			3
15	1764.8261	882.9167	1747.7996	874.4034	1746.8156	873.9114	P	244.1656	122.5864	227.1390	114.0731			2
16							K	147.1128	74.0600	130.0863	65.5468			1

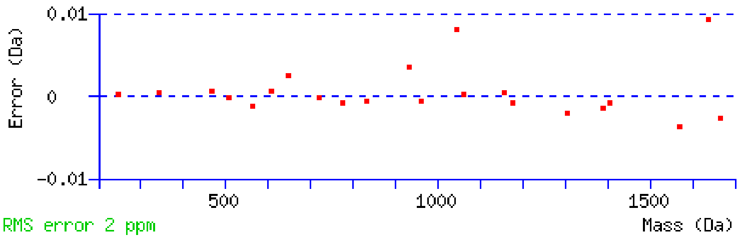

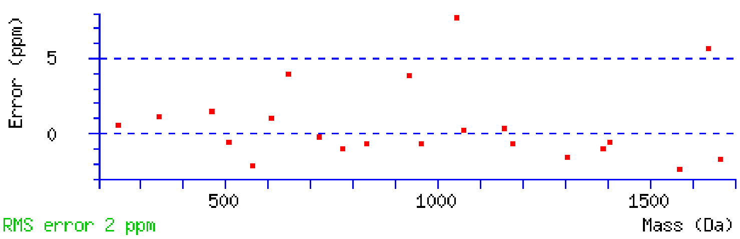

NCBI **BLAST** search of RPCFSALEVDETYVPK
(Parameters: blastp, nr protein database, expect=20000, no filter, PAM30)
Other BLAST web gateways

All matches to this query

Score	Mr(calc):	Delta	Sequence
84.7	1909.9244	-0.0012	RPCFSALEVDETYVPK

Mascot: http://www.matrixscience.com/

Mascot Search Results

Peptide View

MS/MS Fragmentation of **YICENQDSISSK**
Found in **sp|P02768|ALBU_HUMAN**, Serum albumin OS=Homo sapiens GN=ALB PE=1 SV=2

Match to Query 5504: 1433.598368 from(717.806460,2+) intensity(5163122.5000) scans(7564) rtinseconds(1480) index(5860)
Title: 150808_TTSH_Patient_Plasma_54_Spectrum021590_scans__7564_RTINSECONDS=1480
Data file L:\\Ard_TTSH\\T1D\\T150808_TTSH_Patient_Plasma_54.mgf

Click mouse within plot area to zoom in by factor of two about that point

Or, to Da

Label all possible matches ○ Label matches used for scoring ⊙

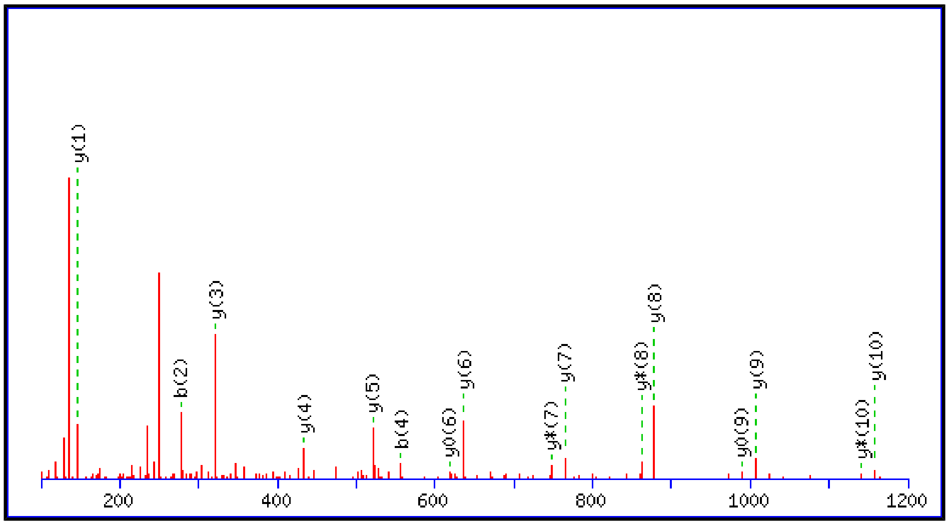

Monoisotopic mass of neutral peptide Mr(calc): 1433.5980
Variable modifications:
C3 : 4Trioxidation (CMWY)
Ions Score: 75 **Expect:** 6.1e-006
Matches : 16/116 fragment ions using 22 most intense peaks (help)

#	b	b^{++}	b*	b*$^{++}$	b^{0}	b^{0++}	Seq.	y	y^{++}	y*	y*$^{++}$	y^{0}	y^{0++}	#
1	164.0706	82.5389					Y							12
2	**277.1547**	139.0810					I	1271.5420	636.2746	1254.5154	627.7614	1253.5314	627.2693	11
3	428.1486	214.5779					C	***1158.4579***	579.7326	1141.4314	571.2193	1140.4474	570.7273	10
4	**557.1912**	279.0992			539.1806	270.0940	E	***1007.4640***	504.2356	990.4374	495.7224	989.4534	495.2304	9
5	671.2341	336.1207	654.2076	327.6074	653.2236	327.1154	N	***878.4214***	439.7143	861.3949	431.2011	860.4108	430.7091	8
6	799.2927	400.1500	782.2661	391.6367	781.2821	391.1447	Q	***764.3785***	382.6929	747.3519	374.1796	746.3679	373.6876	7
7	914.3196	457.6635	897.2931	449.1502	896.3091	448.6582	D	***636.3199***	318.6636	619.2933	310.1503	618.3093	309.6583	6
8	1001.3517	501.1795	984.3251	492.6662	983.3411	492.1742	S	***521.2930***	261.1501	504.2664	252.6368	503.2824	252.1448	5
9	1114.4357	557.7215	1097.4092	549.2082	1096.4252	548.7162	I	***434.2609***	217.6341	417.2344	209.1208	416.2504	208.6288	4
10	1201.4678	601.2375	1184.4412	592.7242	1183.4572	592.2322	S	***321.1769***	161.0921	304.1503	152.5788	303.1663	152.0868	3
11	1288.4998	644.7535	1271.4732	636.2403	1270.4892	635.7482	S	234.1448	117.5761	217.1183	109.0628	216.1343	108.5708	2
12							K	***147.1128***	74.0600	130.0863	65.5468			1

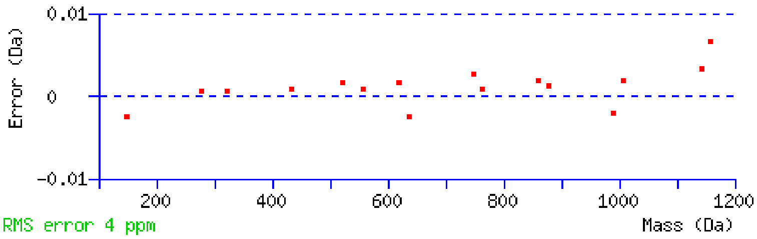

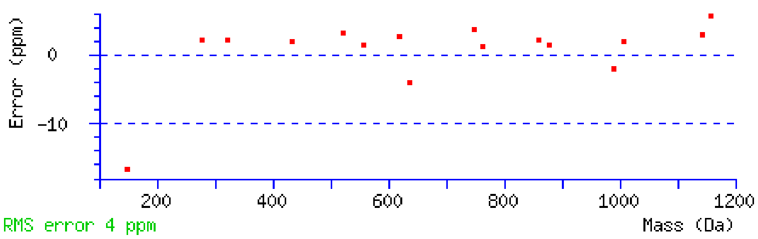

NCBI **BLAST** search of YICENQDSISSK
(Parameters: blastp, nr protein database, expect=20000, no filter, PAM30)
Other BLAST web gateways

All matches to this query

Score	Mr(calc):	Delta	Sequence
75.0	1433.5980	0.0004	YICENQDSISSK
54.9	1433.5980	0.0004	YICENQDSISSK
0.4	1431.6041	1.9942	XEESENEFYIK

Mascot: http://www.matrixscience.com/

Mascot Search Results

Peptide View

MS/MS Fragmentation of **YICENQDSISSK**
Found in **sp|P02768|ALBU_HUMAN**, Serum albumin OS=Homo sapiens GN=ALB PE=1 SV=2

Match to Query 5504: 1433.598368 from(717.806460,2+) intensity(5163122.5000) scans(7564) rtinseconds(1480) index(5860)
Title: N50808_TTSH_Patient_Plasma_54_Spectrum006226_scans__7564_RTINSECONDS=1480
Data file L:\\Ard_TTSH\\TN1D\\TN50808_TTSH_Patient_Plasma_54.mgf

Click mouse within plot area to zoom in by factor of two about that point
Or, to Da
Label all possible matches Label matches used for scoring

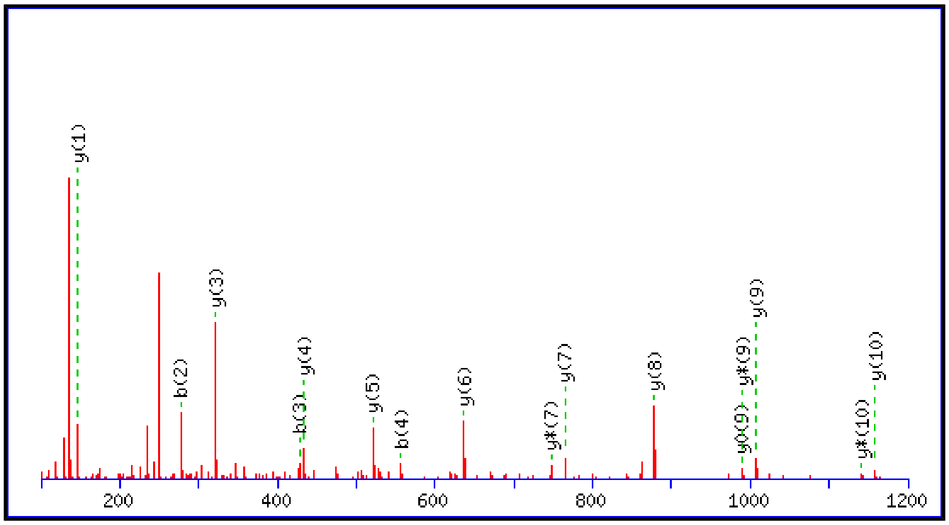

Monoisotopic mass of neutral peptide Mr(calc): 1433.5980
Variable modifications:
C3 : 4Trioxidation (CMWY)
Ions Score: 75 **Expect:** 6.7e-006
Matches : 16/116 fragment ions using 22 most intense peaks (help)

#	b	b++	b*	b*++	b0	b0++	Seq.	y	y++	y*	y*++	y0	y0++	#
1	164.0706	82.5389					Y							12
2	**277.1547**	139.0810					I	1271.5420	636.2746	1254.5154	627.7614	1253.5314	627.2693	11
3	**428.1486**	214.5779					C	***1158.4579***	579.7326	1141.4314	571.2193	1140.4474	570.7273	10
4	**557.1912**	279.0992			539.1806	270.0940	E	***1007.4640***	504.2356	990.4374	495.7224	989.4534	495.2304	9
5	671.2341	336.1207	654.2076	327.6074	653.2236	327.1154	N	***878.4214***	439.7143	861.3949	431.2011	860.4108	430.7091	8
6	799.2927	400.1500	782.2661	391.6367	781.2821	391.1447	Q	***764.3785***	382.6929	747.3519	374.1796	746.3679	373.6876	7
7	914.3196	457.6635	897.2931	449.1502	896.3091	448.6582	D	***636.3199***	318.6636	619.2933	310.1503	618.3093	309.6583	6
8	1001.3517	501.1795	984.3251	492.6662	983.3411	492.1742	S	***521.2930***	261.1501	504.2664	252.6368	503.2824	252.1448	5
9	1114.4357	557.7215	1097.4092	549.2082	1096.4252	548.7162	I	***434.2609***	217.6341	417.2344	209.1208	416.2504	208.6288	4
10	1201.4678	601.2375	1184.4412	592.7242	1183.4572	592.2322	S	***321.1769***	161.0921	304.1503	152.5788	303.1663	152.0868	3
11	1288.4998	644.7535	1271.4732	636.2403	1270.4892	635.7482	S	234.1448	117.5761	217.1183	109.0628	216.1343	108.5708	2
12							K	***147.1128***	74.0600	130.0863	65.5468			1

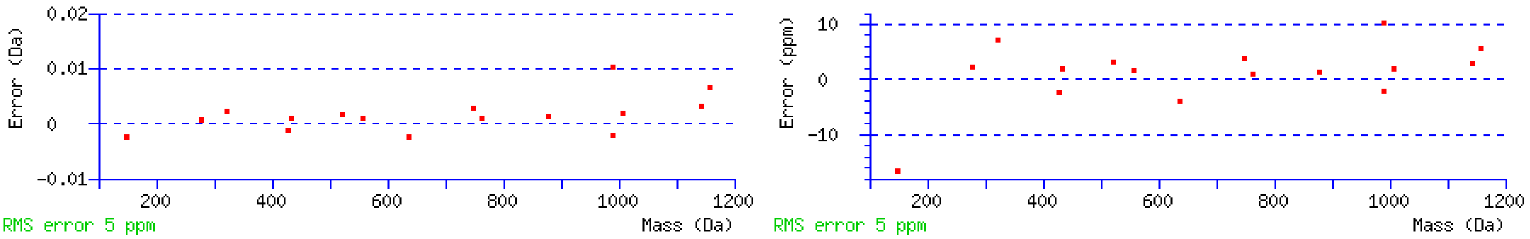

NCBI **BLAST** search of YICENQDSISSK
(Parameters: blastp, nr protein database, expect=20000, no filter, PAM30)
Other BLAST web gateways

All matches to this query

Score	Mr(calc):	Delta	Sequence
74.6	1433.5980	0.0004	YICENQDSISSK
54.5	1433.5980	0.0004	YICENQDSISSK
0.5	1431.6041	1.9942	XEESENEFYIK

Mascot: http://www.matrixscience.com/

Mascot Search Results

Peptide View

MS/MS Fragmentation of **YICENQDSISSK**
Found in **sp|P02768|ALBU_HUMAN**, Serum albumin OS=Homo sapiens GN=ALB PE=1 SV=2

Match to Query 5434: 1433.597268 from(717.805910,2+) intensity(22906332.0000) scans(6374) rtinseconds(1190) index(5116)
Title: N50808_TTSH_Patient_Plasma_84_Spectrum005462_scans__6374_RTINSECONDS=1190
Data file L:\\Ard_TTSH\\TN1D\\TN50808_TTSH_Patient_Plasma_84.mgf

Click mouse within plot area to zoom in by factor of two about that point
Or, to Da
Label all possible matches ○ Label matches used for scoring ⊙

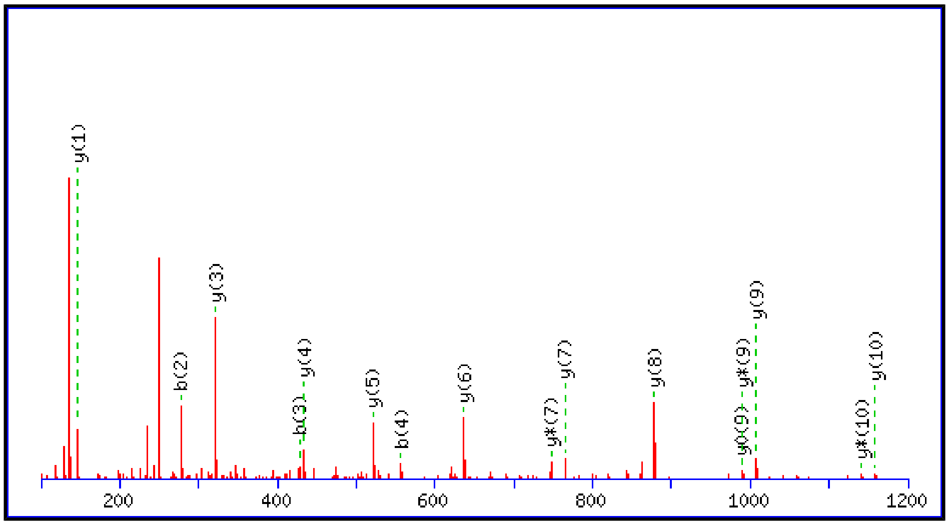

Monoisotopic mass of neutral peptide Mr(calc): 1433.5980
Variable modifications:
C3 : 4Trioxidation (CMWY)
Ions Score: 75 **Expect:** 6.7e-006
Matches : 16/116 fragment ions using 22 most intense peaks (help)

#	b	b^{++}	b*	b^{*++}	b^0	b^{0++}	Seq.	y	y^{++}	y*	y^{*++}	y^0	y^{0++}	#
1	164.0706	82.5389					Y							12
2	**277.1547**	139.0810					I	1271.5420	636.2746	1254.5154	627.7614	1253.5314	627.2693	11
3	**428.1486**	214.5779					C	***1158.4579***	579.7326	1141.4314	571.2193	1140.4474	570.7273	10
4	**557.1912**	279.0992			539.1806	270.0940	E	***1007.4640***	504.2356	990.4374	495.7224	989.4534	495.2304	9
5	671.2341	336.1207	654.2076	327.6074	653.2236	327.1154	N	***878.4214***	439.7143	861.3949	431.2011	860.4108	430.7091	8
6	799.2927	400.1500	782.2661	391.6367	781.2821	391.1447	Q	***764.3785***	382.6929	747.3519	374.1796	746.3679	373.6876	7
7	914.3196	457.6635	897.2931	449.1502	896.3091	448.6582	D	***636.3199***	318.6636	619.2933	310.1503	618.3093	309.6583	6
8	1001.3517	501.1795	984.3251	492.6662	983.3411	492.1742	S	***521.2930***	261.1501	504.2664	252.6368	503.2824	252.1448	5
9	1114.4357	557.7215	1097.4092	549.2082	1096.4252	548.7162	I	***434.2609***	217.6341	417.2344	209.1208	416.2504	208.6288	4
10	1201.4678	601.2375	1184.4412	592.7242	1183.4572	592.2322	S	***321.1769***	161.0921	304.1503	152.5788	303.1663	152.0868	3
11	1288.4998	644.7535	1271.4732	636.2403	1270.4892	635.7482	S	234.1448	117.5761	217.1183	109.0628	216.1343	108.5708	2
12							K	***147.1128***	74.0600	130.0863	65.5468			1

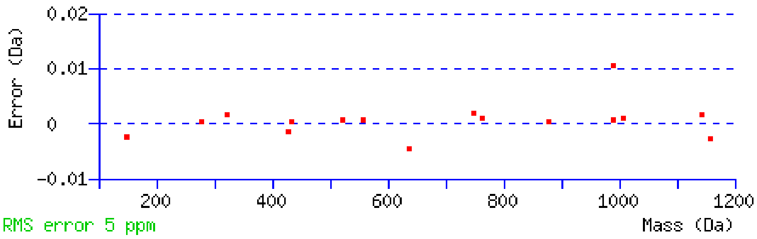

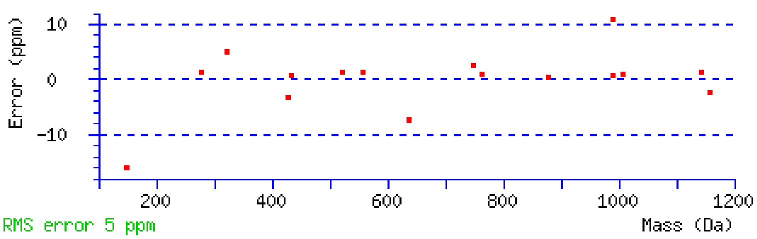

NCBI **BLAST** search of YICENQDSISSK
(Parameters: blastp, nr protein database, expect=20000, no filter, PAM30)
Other BLAST web gateways

All matches to this query

Score	Mr(calc):	Delta	Sequence
74.5	1433.5980	-0.0007	YICENQDSISSK
54.6	1433.5980	-0.0007	YICENQDSISSK

**Mascot:** http://www.matrixscience.com/

Mascot Search Results

Peptide View

MS/MS Fragmentation of **YICENQDSISSK**
Found in **sp|P02768|ALBU_HUMAN**, Serum albumin OS=Homo sapiens GN=ALB PE=1 SV=2

Match to Query 3147: 1433.595068 from(717.804810,2+) intensity(7719326.5000) scans(3428) rtinseconds(786) index(2193)
Title: N50825_TTSH_Patient_Plasma_48_Spectrum002425_scans__3428_RTINSECONDS=786
Data file L:\\Ard_TTSH\\TN1D\\TN50825_TTSH_Patient_Plasma_48.mgf

Click mouse within plot area to zoom in by factor of two about that point

Or, to Da

Label all possible matches ○ Label matches used for scoring ⦿

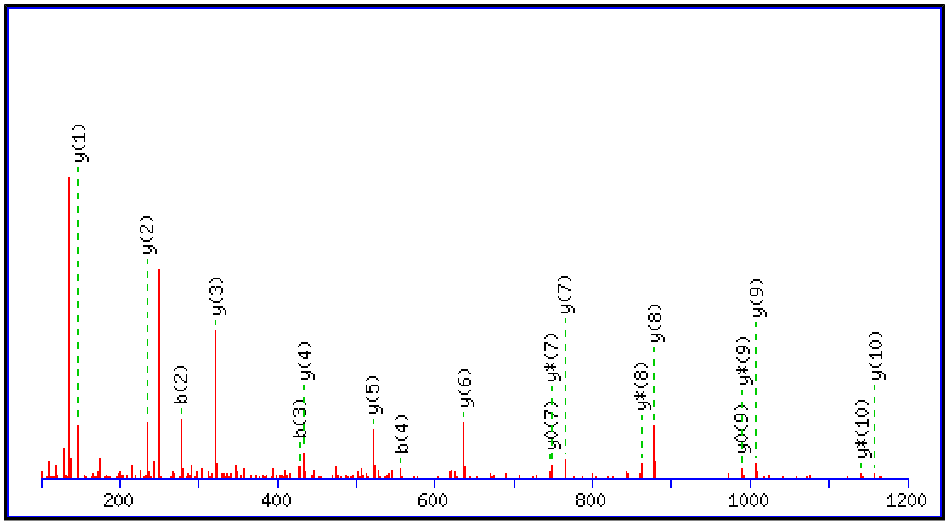

Monoisotopic mass of neutral peptide Mr(calc): 1433.5980
Variable modifications:
C3 : 4Trioxidation (CMWY)
Ions Score: 72 **Expect:** 1.2e-005
Matches : 19/116 fragment ions using 33 most intense peaks (help)

#	b	b^{++}	b*	b^{*++}	b^{0}	b^{0++}	Seq.	y	y^{++}	y*	y^{*++}	y^{0}	y^{0++}	#
1	164.0706	82.5389					Y							12
2	**277.1547**	139.0810					I	1271.5420	636.2746	1254.5154	627.7614	1253.5314	627.2693	11
3	**428.1486**	214.5779					C	***1158.4579***	579.7326	1141.4314	571.2193	1140.4474	570.7273	10
4	**557.1912**	279.0992			539.1806	270.0940	E	***1007.4640***	504.2356	990.4374	495.7224	989.4534	495.2304	9
5	671.2341	336.1207	654.2076	327.6074	653.2236	327.1154	N	***878.4214***	439.7143	861.3949	431.2011	860.4108	430.7091	8
6	799.2927	400.1500	782.2661	391.6367	781.2821	391.1447	Q	***764.3785***	382.6929	747.3519	374.1796	746.3679	373.6876	7
7	914.3196	457.6635	897.2931	449.1502	896.3091	448.6582	D	***636.3199***	318.6636	619.2933	310.1503	618.3093	309.6583	6
8	1001.3517	501.1795	984.3251	492.6662	983.3411	492.1742	S	***521.2930***	261.1501	504.2664	252.6368	503.2824	252.1448	5
9	1114.4357	557.7215	1097.4092	549.2082	1096.4252	548.7162	I	***434.2609***	217.6341	417.2344	209.1208	416.2504	208.6288	4
10	1201.4678	601.2375	1184.4412	592.7242	1183.4572	592.2322	S	***321.1769***	161.0921	304.1503	152.5788	303.1663	152.0868	3
11	1288.4998	644.7535	1271.4732	636.2403	1270.4892	635.7482	S	***234.1448***	117.5761	217.1183	109.0628	216.1343	108.5708	2
12							K	***147.1128***	74.0600	130.0863	65.5468			1

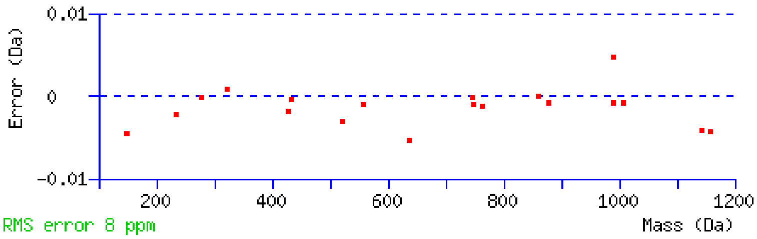

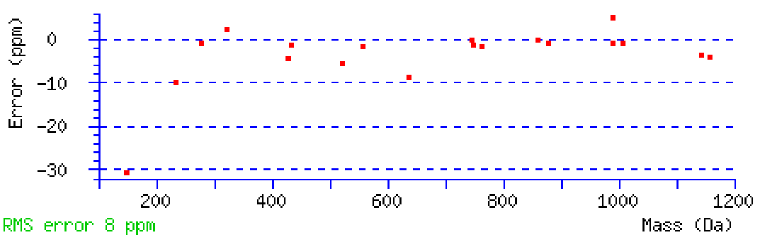

NCBI **BLAST** search of YICENQDSISSK
(Parameters: blastp, nr protein database, expect=20000, no filter, PAM30)
Other BLAST web gateways

All matches to this query

Score	Mr(calc):	Delta	Sequence
72.0	1433.5980	-0.0029	YICENQDSISSK
54.2	1433.5980	-0.0029	YICENQDSISSK

Mascot: http://www.matrixscience.com/

Mascot Search Results

Peptide View

MS/MS Fragmentation of **YICENQDSISSK**
Found in **sp|P02768|ALBU_HUMAN**, Serum albumin OS=Homo sapiens GN=ALB PE=1 SV=2

Match to Query 5256: 1433.598128 from(717.806340,2+) intensity(1691816.5000) scans(4885) rtinseconds(915) index(3656)
Title: N50801_TTSH_Patient_Plasma_52_Spectrum003935_scans__4885_RTINSECONDS=915
Data file L:\\Ard_TTSH\\TN1D\\TN50801_TTSH_Patient_Plasma_52.mgf

Click mouse within plot area to zoom in by factor of two about that point
Or, to Da
Label all possible matches ○ Label matches used for scoring ⊙

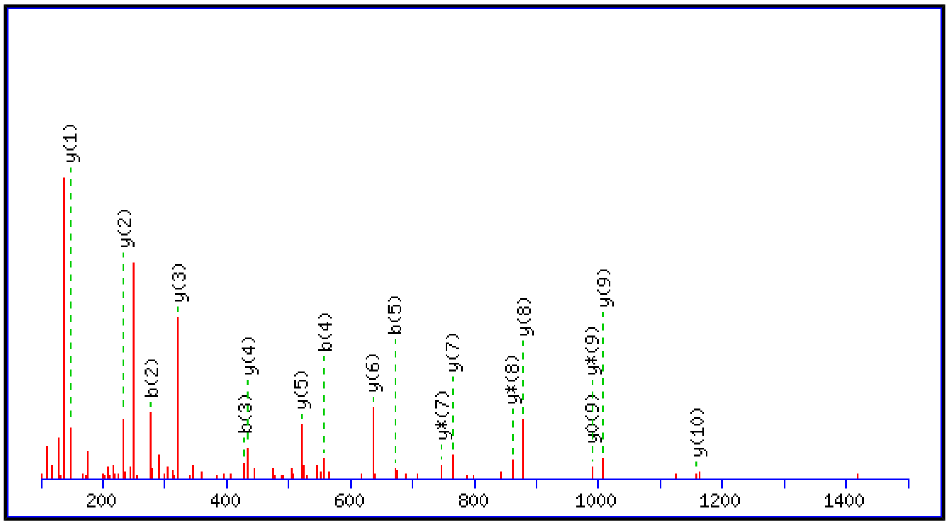

Monoisotopic mass of neutral peptide Mr(calc): 1433.5980
Variable modifications:
C3 : 4Trioxidation (CMWY)
Ions Score: 72 **Expect:** 1.3e-005
Matches : 18/116 fragment ions using 32 most intense peaks (help)

#	b	b^{++}	b*	b^{*++}	b^{0}	b^{0++}	Seq.	y	y^{++}	y*	y^{*++}	y^{0}	y^{0++}	#
1	164.0706	82.5389					Y							12
2	**277.1547**	139.0810					I	1271.5420	636.2746	1254.5154	627.7614	1253.5314	627.2693	11
3	**428.1486**	214.5779					C	***1158.4579***	579.7326	1141.4314	571.2193	1140.4474	570.7273	10
4	**557.1912**	279.0992			539.1806	270.0940	E	***1007.4640***	504.2356	990.4374	495.7224	989.4534	495.2304	9
5	**671.2341**	336.1207	654.2076	327.6074	653.2236	327.1154	N	***878.4214***	439.7143	861.3949	431.2011	860.4108	430.7091	8
6	799.2927	400.1500	782.2661	391.6367	781.2821	391.1447	Q	***764.3785***	382.6929	747.3519	374.1796	746.3679	373.6876	7
7	914.3196	457.6635	897.2931	449.1502	896.3091	448.6582	D	***636.3199***	318.6636	619.2933	310.1503	618.3093	309.6583	6
8	1001.3517	501.1795	984.3251	492.6662	983.3411	492.1742	S	***521.2930***	261.1501	504.2664	252.6368	503.2824	252.1448	5
9	1114.4357	557.7215	1097.4092	549.2082	1096.4252	548.7162	I	***434.2609***	217.6341	417.2344	209.1208	416.2504	208.6288	4
10	1201.4678	601.2375	1184.4412	592.7242	1183.4572	592.2322	S	***321.1769***	161.0921	304.1503	152.5788	303.1663	152.0868	3
11	1288.4998	644.7535	1271.4732	636.2403	1270.4892	635.7482	S	***234.1448***	117.5761	217.1183	109.0628	216.1343	108.5708	2
12							K	***147.1128***	74.0600	130.0863	65.5468			1

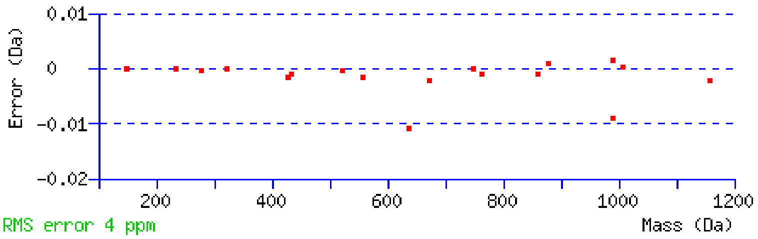

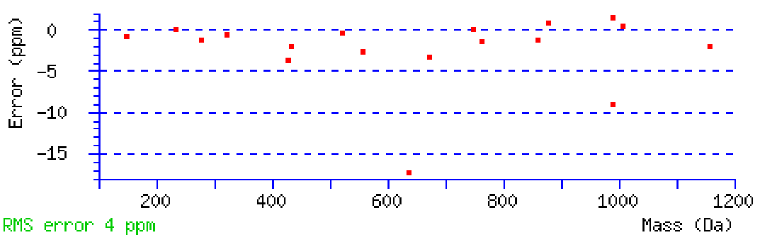

NCBI **BLAST** search of YICENQDSISSK
(Parameters: blastp, nr protein database, expect=20000, no filter, PAM30)
Other BLAST web gateways

All matches to this query

Score	Mr(calc):	Delta	Sequence
71.8	1433.5980	0.0001	YICENQDSISSK
55.7	1433.5980	0.0001	YICENQDSISSK

Mascot: http://www.matrixscience.com/

Mascot Search Results

Peptide View

MS/MS Fragmentation of **YICENQDSISSK**
Found in **sp|P02768|ALBU_HUMAN**, Serum albumin OS=Homo sapiens GN=ALB PE=1 SV=2

Match to Query 5500: 1433.598248 from(717.806400,2+) intensity(3903312.7500) scans(7707) rtinseconds(1527) index(5943)
Title: 150808_TTSH_Patient_Plasma_52_Spectrum021587_scans__7707_RTINSECONDS=1527
Data file L:\\Ard_TTSH\\T1D\\T150808_TTSH_Patient_Plasma_52.mgf

Click mouse within plot area to zoom in by factor of two about that point

Or, to Da

Label all possible matches ○ Label matches used for scoring ⊙

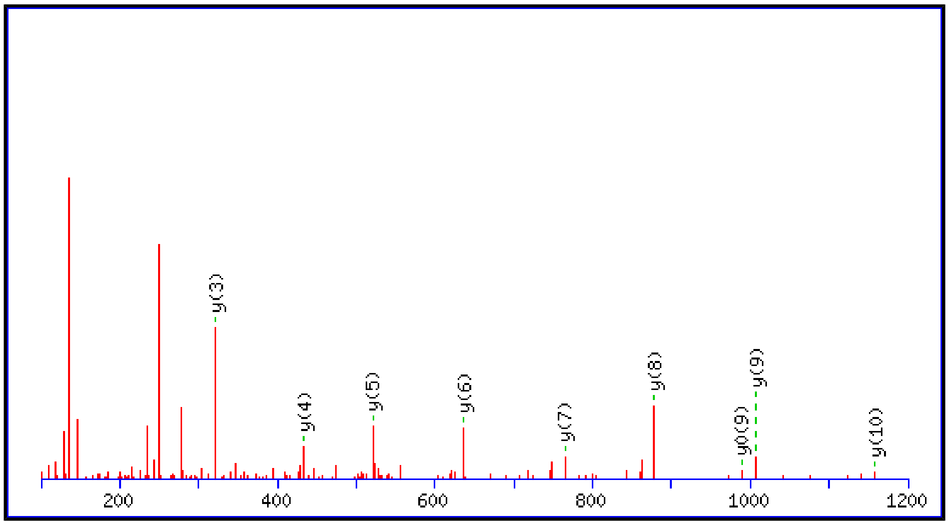

Monoisotopic mass of neutral peptide Mr(calc): 1433.5980
Variable modifications:
C3 : 4Trioxidation (CMWY)
Ions Score: 72 **Expect:** 1.3e-005
Matches : 9/116 fragment ions using 11 most intense peaks (help)

#	b	b^{++}	b*	b^{*++}	b^{0}	b^{0++}	Seq.	y	y^{++}	y*	y^{*++}	y^{0}	y^{0++}	#
1	164.0706	82.5389					Y							12
2	277.1547	139.0810					I	1271.5420	636.2746	1254.5154	627.7614	1253.5314	627.2693	11
3	428.1486	214.5779					C	***1158.4579***	579.7326	1141.4314	571.2193	1140.4474	570.7273	10
4	557.1912	279.0992			539.1806	270.0940	E	***1007.4640***	504.2356	990.4374	495.7224	989.4534	495.2304	9
5	671.2341	336.1207	654.2076	327.6074	653.2236	327.1154	N	***878.4214***	439.7143	861.3949	431.2011	860.4108	430.7091	8
6	799.2927	400.1500	782.2661	391.6367	781.2821	391.1447	Q	***764.3785***	382.6929	747.3519	374.1796	746.3679	373.6876	7
7	914.3196	457.6635	897.2931	449.1502	896.3091	448.6582	D	***636.3199***	318.6636	619.2933	310.1503	618.3093	309.6583	6
8	1001.3517	501.1795	984.3251	492.6662	983.3411	492.1742	S	***521.2930***	261.1501	504.2664	252.6368	503.2824	252.1448	5
9	1114.4357	557.7215	1097.4092	549.2082	1096.4252	548.7162	I	***434.2609***	217.6341	417.2344	209.1208	416.2504	208.6288	4
10	1201.4678	601.2375	1184.4412	592.7242	1183.4572	592.2322	S	***321.1769***	161.0921	304.1503	152.5788	303.1663	152.0868	3
11	1288.4998	644.7535	1271.4732	636.2403	1270.4892	635.7482	S	234.1448	117.5761	217.1183	109.0628	216.1343	108.5708	2
12							K	147.1128	74.0600	130.0863	65.5468			1

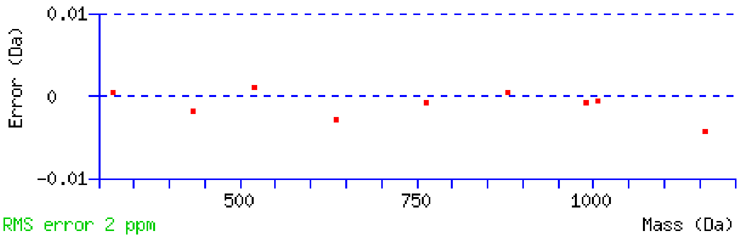

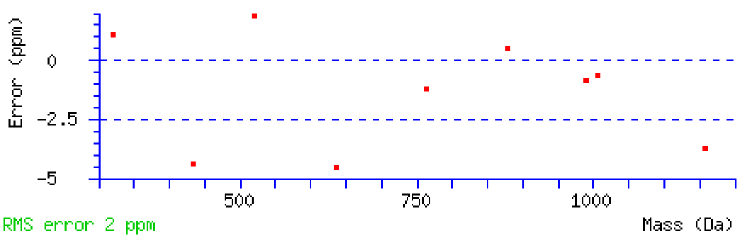

NCBI **BLAST** search of YICENQDSISSK
(Parameters: blastp, nr protein database, expect=20000, no filter, PAM30)
Other BLAST web gateways

All matches to this query

Score	Mr(calc):	Delta	Sequence
71.6	1433.5980	0.0002	YICENQDSISSK
54.8	1433.5980	0.0002	YICENQDSISSK
1.8	1432.5850	1.0133	EKPYKCEECEK

Mascot: http://www.matrixscience.com/

Mascot Search Results

Peptide View

MS/MS Fragmentation of **YICENQDSISSK**
Found in **sp|P02768|ALBU_HUMAN**, Serum albumin OS=Homo sapiens GN=ALB PE=1 SV=2

Match to Query 5256: 1433.598128 from(717.806340,2+) intensity(1691816.5000) scans(4885) rtinseconds(915) index(3656)
Title: 150801_TTSH_Patient_Plasma_52_Spectrum018880_scans__4885_RTINSECONDS=915
Data file L:\\Ard_TTSH\\T1D\\T150801_TTSH_Patient_Plasma_52.mgf

Click mouse within plot area to zoom in by factor of two about that point
Or, to Da
Label all possible matches Label matches used for scoring

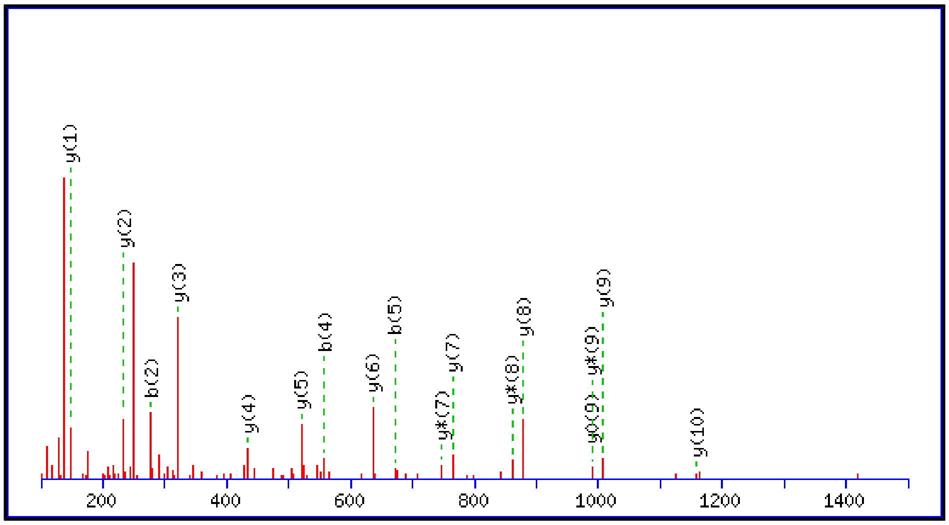

Monoisotopic mass of neutral peptide Mr(calc): 1433.5980
Variable modifications:
C3 : 4Trioxidation (CMWY)
Ions Score: 72 **Expect:** 1.4e-005
Matches : 17/116 fragment ions using 31 most intense peaks (help)

#	b	b^{++}	b*	b^{*++}	b^0	b^{0++}	Seq.	y	y^{++}	y*	y^{*++}	y^0	y^{0++}	#
1	164.0706	82.5389					Y							12
2	**277.1547**	139.0810					I	1271.5420	636.2746	1254.5154	627.7614	1253.5314	627.2693	11
3	428.1486	214.5779					C	***1158.4579***	579.7326	1141.4314	571.2193	1140.4474	570.7273	10
4	**557.1912**	279.0992			539.1806	270.0940	E	***1007.4640***	504.2356	990.4374	495.7224	989.4534	495.2304	9
5	**671.2341**	336.1207	654.2076	327.6074	653.2236	327.1154	N	***878.4214***	439.7143	861.3949	431.2011	860.4108	430.7091	8
6	799.2927	400.1500	782.2661	391.6367	781.2821	391.1447	Q	***764.3785***	382.6929	747.3519	374.1796	746.3679	373.6876	7
7	914.3196	457.6635	897.2931	449.1502	896.3091	448.6582	D	***636.3199***	318.6636	619.2933	310.1503	618.3093	309.6583	6
8	1001.3517	501.1795	984.3251	492.6662	983.3411	492.1742	S	***521.2930***	261.1501	504.2664	252.6368	503.2824	252.1448	5
9	1114.4357	557.7215	1097.4092	549.2082	1096.4252	548.7162	I	***434.2609***	217.6341	417.2344	209.1208	416.2504	208.6288	4
10	1201.4678	601.2375	1184.4412	592.7242	1183.4572	592.2322	S	***321.1769***	161.0921	304.1503	152.5788	303.1663	152.0868	3
11	1288.4998	644.7535	1271.4732	636.2403	1270.4892	635.7482	S	***234.1448***	117.5761	217.1183	109.0628	216.1343	108.5708	2
12							K	***147.1128***	74.0600	130.0863	65.5468			1

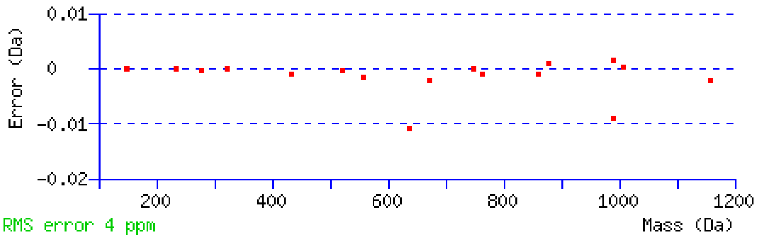

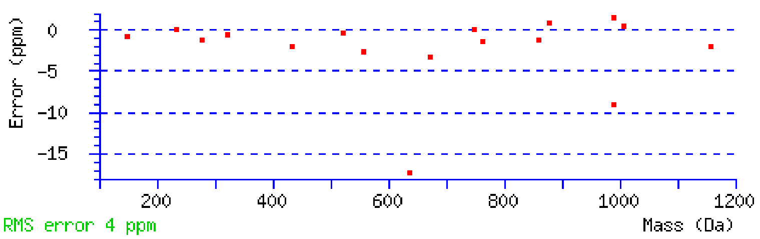

NCBI **BLAST** search of YICENQDSISSK
(Parameters: blastp, nr protein database, expect=20000, no filter, PAM30)
Other BLAST web gateways

All matches to this query

Score	Mr(calc):	Delta	Sequence
71.5	1433.5980	0.0001	YICENQDSISSK
57.0	1433.5980	0.0001	YICENQDSISSK

**Mascot:** http://www.matrixscience.com/

Mascot Search Results

Peptide View

MS/MS Fragmentation of **YICENQDSISSK**
Found in **sp|P02768|ALBU_HUMAN**, Serum albumin OS=Homo sapiens GN=ALB PE=1 SV=2

Match to Query 5500: 1433.598248 from(717.806400,2+) intensity(3903312.7500) scans(7707) rtinseconds(1527) index(5943)
Title: N50808_TTSH_Patient_Plasma_52_Spectrum006286_scans__7707_RTINSECONDS=1527
Data file L:\\Ard_TTSH\\TN1D\\TN50808_TTSH_Patient_Plasma_52.mgf

Click mouse within plot area to zoom in by factor of two about that point
Or, to Da
Label all possible matches Label matches used for scoring

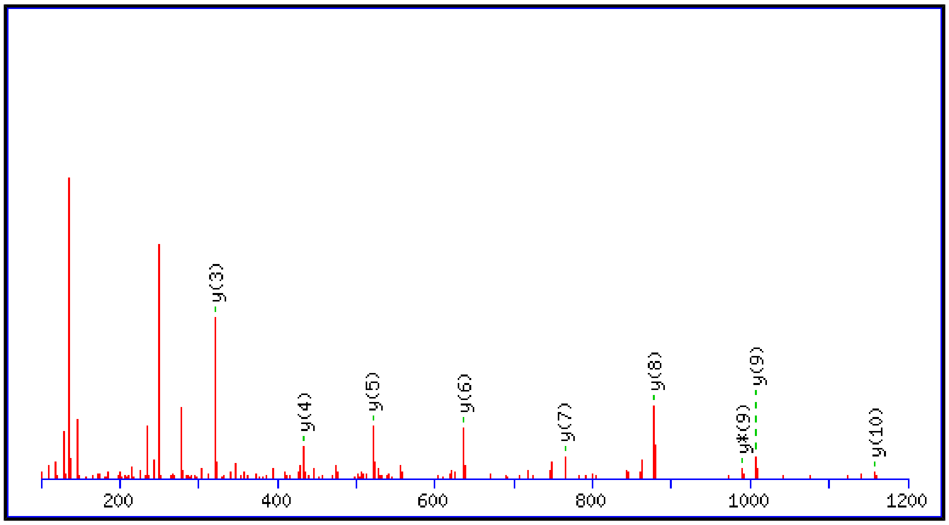

Monoisotopic mass of neutral peptide Mr(calc): 1433.5980
Variable modifications:
C3 : 4Trioxidation (CMWY)
Ions Score: 71 **Expect:** 1.5e-005
Matches : 9/116 fragment ions using 11 most intense peaks (help)

#	b	b^{++}	b*	b*$^{++}$	b^{0}	b^{0++}	Seq.	y	y^{++}	y*	y*$^{++}$	y^{0}	y^{0++}	#
1	164.0706	82.5389					Y							12
2	277.1547	139.0810					I	1271.5420	636.2746	1254.5154	627.7614	1253.5314	627.2693	11
3	428.1486	214.5779					C	***1158.4579***	579.7326	1141.4314	571.2193	1140.4474	570.7273	10
4	557.1912	279.0992			539.1806	270.0940	E	***1007.4640***	504.2356	990.4374	495.7224	989.4534	495.2304	9
5	671.2341	336.1207	654.2076	327.6074	653.2236	327.1154	N	***878.4214***	439.7143	861.3949	431.2011	860.4108	430.7091	8
6	799.2927	400.1500	782.2661	391.6367	781.2821	391.1447	Q	***764.3785***	382.6929	747.3519	374.1796	746.3679	373.6876	7
7	914.3196	457.6635	897.2931	449.1502	896.3091	448.6582	D	***636.3199***	318.6636	619.2933	310.1503	618.3093	309.6583	6
8	1001.3517	501.1795	984.3251	492.6662	983.3411	492.1742	S	***521.2930***	261.1501	504.2664	252.6368	503.2824	252.1448	5
9	1114.4357	557.7215	1097.4092	549.2082	1096.4252	548.7162	I	***434.2609***	217.6341	417.2344	209.1208	416.2504	208.6288	4
10	1201.4678	601.2375	1184.4412	592.7242	1183.4572	592.2322	S	***321.1769***	161.0921	304.1503	152.5788	303.1663	152.0868	3
11	1288.4998	644.7535	1271.4732	636.2403	1270.4892	635.7482	S	234.1448	117.5761	217.1183	109.0628	216.1343	108.5708	2
12							K	147.1128	74.0600	130.0863	65.5468			1

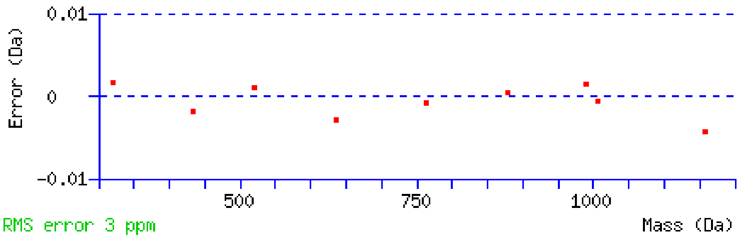

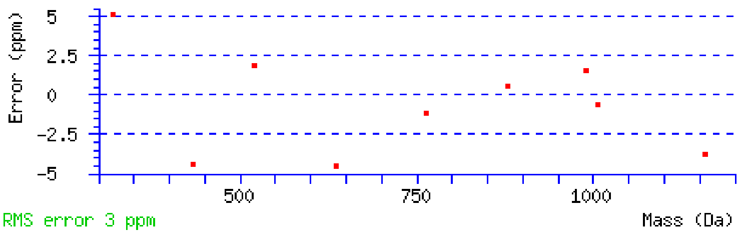

NCBI **BLAST** search of YICENQDSISSK
(Parameters: blastp, nr protein database, expect=20000, no filter, PAM30)
Other BLAST web gateways

All matches to this query

Score	Mr(calc):	Delta	Sequence
71.2	1433.5980	0.0002	YICENQDSISSK
54.4	1433.5980	0.0002	YICENQDSISSK
1.4	1432.5850	1.0133	EKPYKCEECEK

Mascot Search Results

Peptide View

MS/MS Fragmentation of **YICENQDSISSK**
Found in **sp|P02768|ALBU_HUMAN**, Serum albumin OS=Homo sapiens GN=ALB PE=1 SV=2

Match to Query 5434: 1433.597268 from(717.805910,2+) intensity(22906332.0000) scans(6374) rtinseconds(1190) index(5116)
Title: 150808_TTSH_Patient_Plasma_84_Spectrum021381_scans__6374_RTINSECONDS=1190
Data file L:\\Ard_TTSH\\T1D\\T150808_TTSH_Patient_Plasma_84.mgf

Click mouse within plot area to zoom in by factor of two about that point

Or, to Da

Label all possible matches Label matches used for scoring

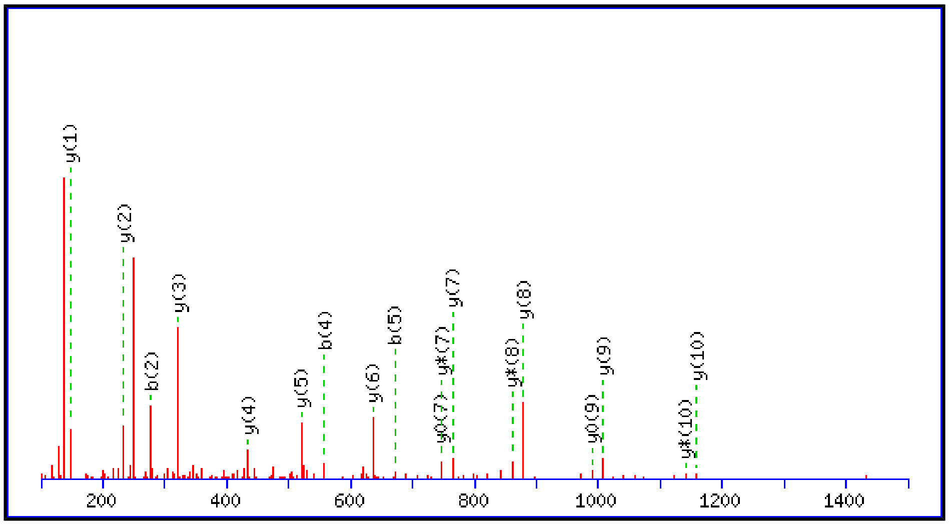

Monoisotopic mass of neutral peptide Mr(calc): 1433.5980
Variable modifications:
C3 : 4Trioxidation (CMWY)
Ions Score: 70 **Expect:** 1.7e-005
Matches : 18/116 fragment ions using 33 most intense peaks (help)

#	b	b^{++}	b*	b*$^{++}$	b^{0}	b^{0++}	Seq.	y	y^{++}	y*	y*$^{++}$	y^{0}	y^{0++}	#
1	164.0706	82.5389					Y							12
2	**277.1547**	139.0810					I	1271.5420	636.2746	1254.5154	627.7614	1253.5314	627.2693	11
3	428.1486	214.5779					C	***1158.4579***	579.7326	1141.4314	571.2193	1140.4474	570.7273	10
4	**557.1912**	279.0992			539.1806	270.0940	E	***1007.4640***	504.2356	990.4374	495.7224	989.4534	495.2304	9
5	**671.2341**	336.1207	654.2076	327.6074	653.2236	327.1154	N	***878.4214***	439.7143	861.3949	431.2011	860.4108	430.7091	8
6	799.2927	400.1500	782.2661	391.6367	781.2821	391.1447	Q	***764.3785***	382.6929	747.3519	374.1796	746.3679	373.6876	7
7	914.3196	457.6635	897.2931	449.1502	896.3091	448.6582	D	***636.3199***	318.6636	619.2933	310.1503	618.3093	309.6583	6
8	1001.3517	501.1795	984.3251	492.6662	983.3411	492.1742	S	***521.2930***	261.1501	504.2664	252.6368	503.2824	252.1448	5
9	1114.4357	557.7215	1097.4092	549.2082	1096.4252	548.7162	I	***434.2609***	217.6341	417.2344	209.1208	416.2504	208.6288	4
10	1201.4678	601.2375	1184.4412	592.7242	1183.4572	592.2322	S	***321.1769***	161.0921	304.1503	152.5788	303.1663	152.0868	3
11	1288.4998	644.7535	1271.4732	636.2403	1270.4892	635.7482	S	***234.1448***	117.5761	217.1183	109.0628	216.1343	108.5708	2
12							K	***147.1128***	74.0600	130.0863	65.5468			1

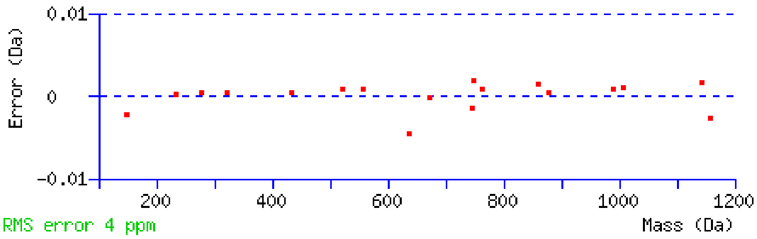

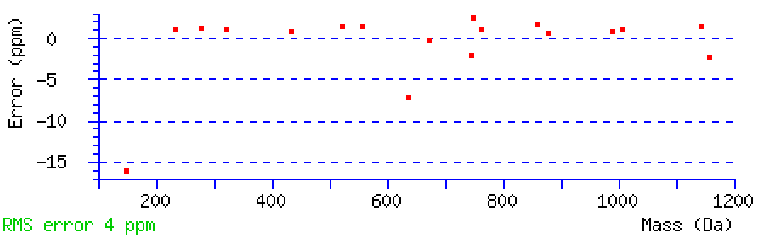

NCBI **BLAST** search of YICENQDSISSK
(Parameters: blastp, nr protein database, expect=20000, no filter, PAM30)
Other BLAST web gateways

All matches to this query

Score	Mr(calc):	Delta	Sequence
70.5	1433.5980	-0.0007	YICENQDSISSK
52.6	1433.5980	-0.0007	YICENQDSISSK

**Mascot:** http://www.matrixscience.com/

Mascot Search Results

Peptide View

MS/MS Fragmentation of **YICENQDSISSK**
Found in **sp|P02768|ALBU_HUMAN**, Serum albumin OS=Homo sapiens GN=ALB PE=1 SV=2

Match to Query 5442: 1433.597748 from(717.806150,2+) intensity(3784288.7500) scans(6419) rtinseconds(1211) index(5112)
Title: 150808_TTSH_Patient_Plasma_82_Spectrum021378_scans__6419_RTINSECONDS=1211
Data file L:\\Ard_TTSH\\T1D\\T150808_TTSH_Patient_Plasma_82.mgf

Click mouse within plot area to zoom in by factor of two about that point

Or, to Da

Label all possible matches ○ Label matches used for scoring ⊙

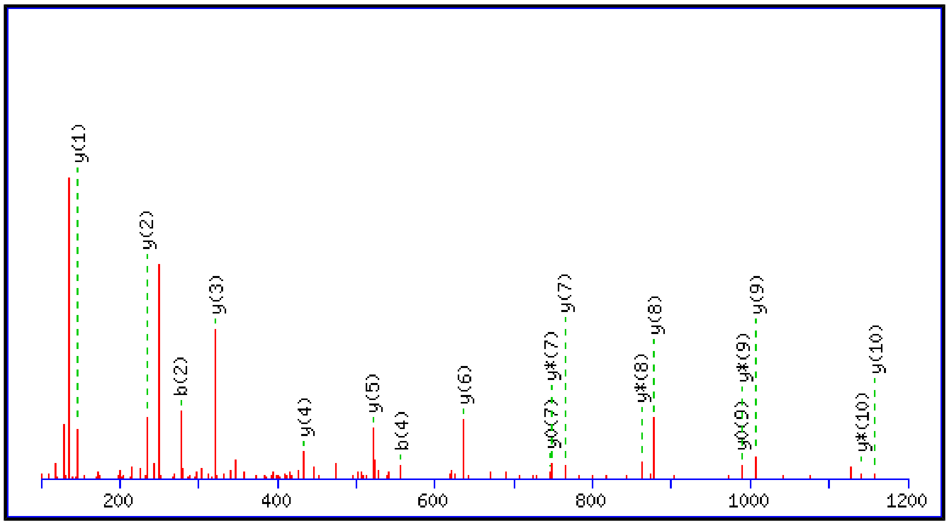


```
Monoisotopic mass of neutral peptide Mr(calc): 1433.5980
Variable modifications:
C3     : 4Trioxidation (CMWY)
Ions Score: 70  Expect: 1.8e-005
Matches : 18/116 fragment ions using 33 most intense peaks   (help)
```

#	b	b^{++}	b*	b^{*++}	b^0	b^{0++}	Seq.	y	y^{++}	y*	y^{*++}	y^0	y^{0++}	#
1	164.0706	82.5389					Y							12
2	277.1547	139.0810					I	1271.5420	636.2746	1254.5154	627.7614	1253.5314	627.2693	11
3	428.1486	214.5779					C	1158.4579	579.7326	1141.4314	571.2193	1140.4474	570.7273	10
4	557.1912	279.0992			539.1806	270.0940	E	1007.4640	504.2356	990.4374	495.7224	989.4534	495.2304	9
5	671.2341	336.1207	654.2076	327.6074	653.2236	327.1154	N	878.4214	439.7143	861.3949	431.2011	860.4108	430.7091	8
6	799.2927	400.1500	782.2661	391.6367	781.2821	391.1447	Q	764.3785	382.6929	747.3519	374.1796	746.3679	373.6876	7
7	914.3196	457.6635	897.2931	449.1502	896.3091	448.6582	D	636.3199	318.6636	619.2933	310.1503	618.3093	309.6583	6
8	1001.3517	501.1795	984.3251	492.6662	983.3411	492.1742	S	521.2930	261.1501	504.2664	252.6368	503.2824	252.1448	5
9	1114.4357	557.7215	1097.4092	549.2082	1096.4252	548.7162	I	434.2609	217.6341	417.2344	209.1208	416.2504	208.6288	4
10	1201.4678	601.2375	1184.4412	592.7242	1183.4572	592.2322	S	321.1769	161.0921	304.1503	152.5788	303.1663	152.0868	3
11	1288.4998	644.7535	1271.4732	636.2403	1270.4892	635.7482	S	234.1448	117.5761	217.1183	109.0628	216.1343	108.5708	2
12							K	147.1128	74.0600	130.0863	65.5468			1

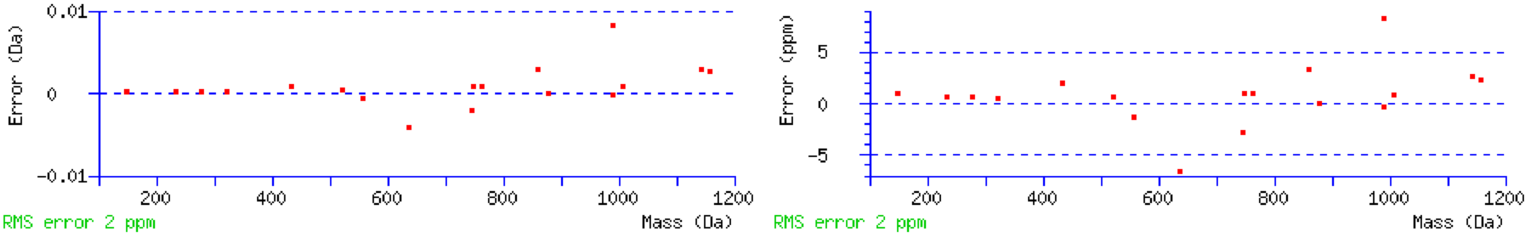

NCBI **BLAST** search of YICENQDSISSK
(Parameters: blastp, nr protein database, expect=20000, no filter, PAM30)
Other BLAST web gateways

All matches to this query

Score	Mr(calc):	Delta	Sequence
70.3	1433.5980	-0.0003	YICENQDSISSK
52.4	1433.5980	-0.0003	YICENQDSISSK

Mascot Search Results

Peptide View

MS/MS Fragmentation of **YICENQDSISSK**
Found in **sp|P02768|ALBU_HUMAN**, Serum albumin OS=Homo sapiens GN=ALB PE=1 SV=2

Match to Query 5139: 1433.596168 from(717.805360,2+) intensity(830344.1875) scans(5423) rtinseconds(1272) index(3451)
Title: 150801_TTSH_Patient_Plasma_66_Spectrum017429_scans__5423_RTINSECONDS=1272
Data file L:\\Ard_TTSH\\T1D\\T150801_TTSH_Patient_Plasma_66.mgf

Click mouse within plot area to zoom in by factor of two about that point
Or, to Da
Label all possible matches ○ Label matches used for scoring ⊙

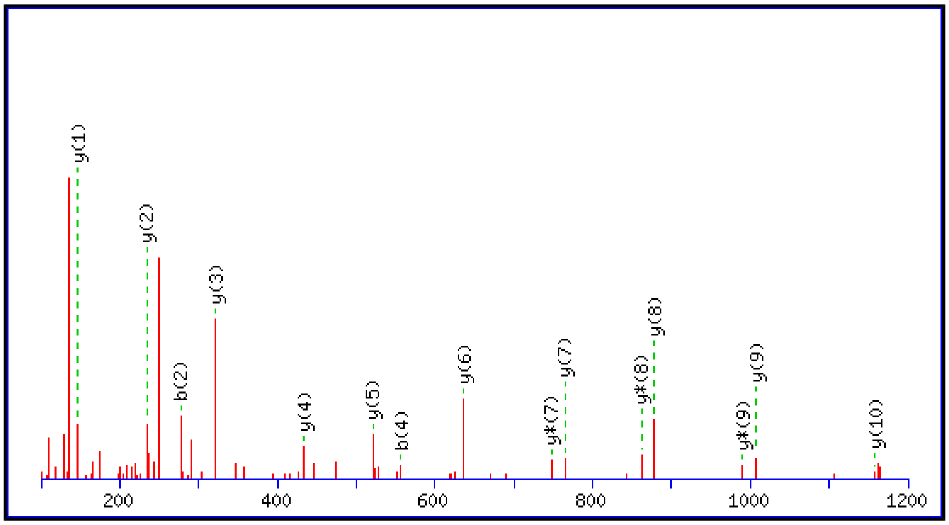

Monoisotopic mass of neutral peptide Mr(calc): 1433.5980
Variable modifications:
C3 : 4Trioxidation (CMWY)
Ions Score: 70 **Expect:** 1.9e-005
Matches : 15/116 fragment ions using 29 most intense peaks (help)

#	b	b^{++}	b*	b^{*++}	b^0	b^{0++}	Seq.	y	y^{++}	y*	y^{*++}	y^0	y^{0++}	#
1	164.0706	82.5389					Y							12
2	277.1547	139.0810					I	1271.5420	636.2746	1254.5154	627.7614	1253.5314	627.2693	11
3	428.1486	214.5779					C	1158.4579	579.7326	1141.4314	571.2193	1140.4474	570.7273	10
4	557.1912	279.0992			539.1806	270.0940	E	1007.4640	504.2356	990.4374	495.7224	989.4534	495.2304	9
5	671.2341	336.1207	654.2076	327.6074	653.2236	327.1154	N	878.4214	439.7143	861.3949	431.2011	860.4108	430.7091	8
6	799.2927	400.1500	782.2661	391.6367	781.2821	391.1447	Q	764.3785	382.6929	747.3519	374.1796	746.3679	373.6876	7
7	914.3196	457.6635	897.2931	449.1502	896.3091	448.6582	D	636.3199	318.6636	619.2933	310.1503	618.3093	309.6583	6
8	1001.3517	501.1795	984.3251	492.6662	983.3411	492.1742	S	521.2930	261.1501	504.2664	252.6368	503.2824	252.1448	5
9	1114.4357	557.7215	1097.4092	549.2082	1096.4252	548.7162	I	434.2609	217.6341	417.2344	209.1208	416.2504	208.6288	4
10	1201.4678	601.2375	1184.4412	592.7242	1183.4572	592.2322	S	321.1769	161.0921	304.1503	152.5788	303.1663	152.0868	3
11	1288.4998	644.7535	1271.4732	636.2403	1270.4892	635.7482	S	234.1448	117.5761	217.1183	109.0628	216.1343	108.5708	2
12							K	147.1128	74.0600	130.0863	65.5468			1

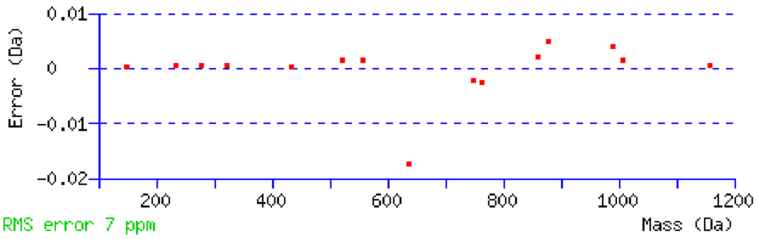

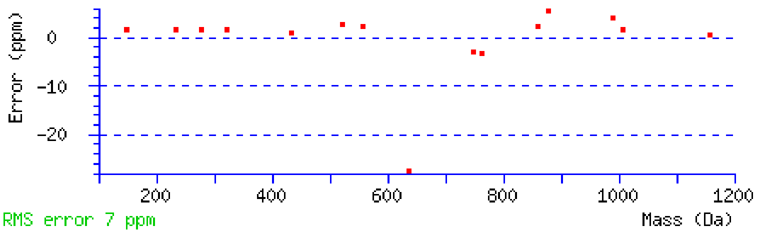

NCBI **BLAST** search of YICENQDSISSK
(Parameters: blastp, nr protein database, expect=20000, no filter, PAM30)
Other BLAST web gateways

All matches to this query

Score	Mr(calc):	Delta	Sequence
70.0	1433.5980	-0.0018	YICENQDSISSK
55.8	1433.5980	-0.0018	YICENQDSISSK

Mascot: http://www.matrixscience.com/

Mascot Search Results

Peptide View

MS/MS Fragmentation of **YICENQDSISSK**
Found in **sp|P02768|ALBU_HUMAN**, Serum albumin OS=Homo sapiens GN=ALB PE=1 SV=2

Match to Query 5442: 1433.597748 from(717.806150,2+) intensity(3784288.7500) scans(6419) rtinseconds(1211) index(5112)
Title: N50808_TTSH_Patient_Plasma_82_Spectrum005470_scans__6419_RTINSECONDS=1211
Data file L:\\Ard_TTSH\\TN1D\\TN50808_TTSH_Patient_Plasma_82.mgf

Click mouse within plot area to zoom in by factor of two about that point
Or, to Da
Label all possible matches Label matches used for scoring

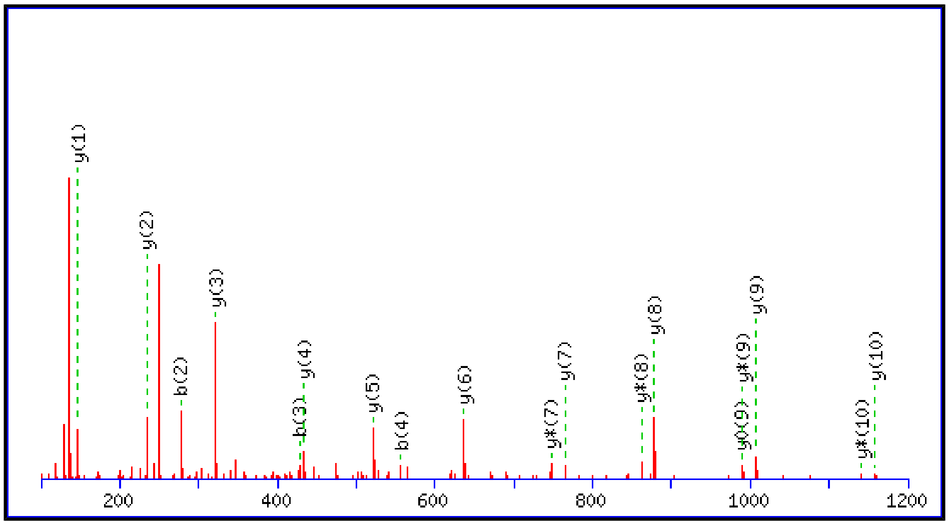

Monoisotopic mass of neutral peptide Mr(calc): 1433.5980
Variable modifications:
C3 : 4Trioxidation (CMWY)
Ions Score: 70 **Expect:** 9.7e-006
Matches : 18/116 fragment ions using 33 most intense peaks (help)

#	b	b^{++}	b*	b^{*++}	b^{0}	b^{0++}	Seq.	y	y^{++}	y*	y^{*++}	y^{0}	y^{0++}	#
1	164.0706	82.5389					Y							12
2	**277.1547**	139.0810					I	1271.5420	636.2746	1254.5154	627.7614	1253.5314	627.2693	11
3	**428.1486**	214.5779					C	***1158.4579***	579.7326	1141.4314	571.2193	1140.4474	570.7273	10
4	**557.1912**	279.0992			539.1806	270.0940	E	***1007.4640***	504.2356	990.4374	495.7224	989.4534	495.2304	9
5	671.2341	336.1207	654.2076	327.6074	653.2236	327.1154	N	***878.4214***	439.7143	861.3949	431.2011	860.4108	430.7091	8
6	799.2927	400.1500	782.2661	391.6367	781.2821	391.1447	Q	***764.3785***	382.6929	747.3519	374.1796	746.3679	373.6876	7
7	914.3196	457.6635	897.2931	449.1502	896.3091	448.6582	D	***636.3199***	318.6636	619.2933	310.1503	618.3093	309.6583	6
8	1001.3517	501.1795	984.3251	492.6662	983.3411	492.1742	S	***521.2930***	261.1501	504.2664	252.6368	503.2824	252.1448	5
9	1114.4357	557.7215	1097.4092	549.2082	1096.4252	548.7162	I	***434.2609***	217.6341	417.2344	209.1208	416.2504	208.6288	4
10	1201.4678	601.2375	1184.4412	592.7242	1183.4572	592.2322	S	***321.1769***	161.0921	304.1503	152.5788	303.1663	152.0868	3
11	1288.4998	644.7535	1271.4732	636.2403	1270.4892	635.7482	S	***234.1448***	117.5761	217.1183	109.0628	216.1343	108.5708	2
12							K	***147.1128***	74.0600	130.0863	65.5468			1

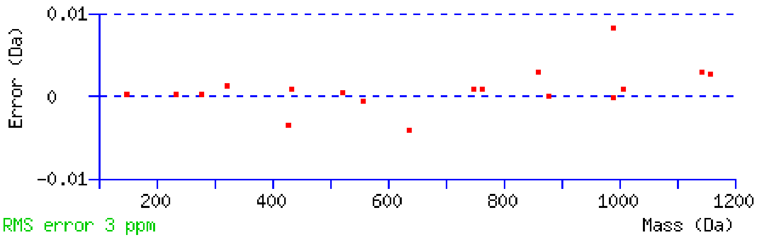

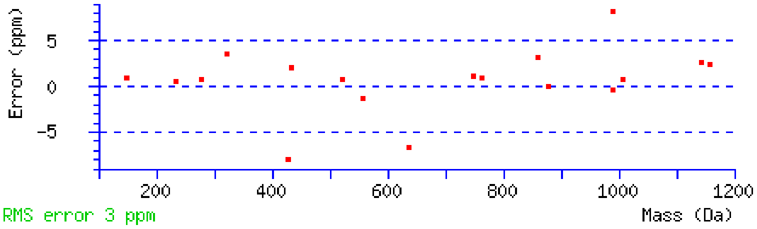

NCBI **BLAST** search of YICENQDSISSK
(Parameters: blastp, nr protein database, expect=20000, no filter, PAM30)
Other BLAST web gateways

All matches to this query

Score	Mr(calc):	Delta	Sequence
69.9	1433.5980	-0.0003	YICENQDSISSK
52.0	1433.5980	-0.0003	YICENQDSISSK

Mascot: http://www.matrixscience.com/

Mascot Search Results

Peptide View

MS/MS Fragmentation of **YICENQDSISSK**
Found in **sp|P02768|ALBU_HUMAN**, Serum albumin OS=Homo sapiens GN=ALB PE=1 SV=2

Match to Query 3147: 1433.595068 from(717.804810,2+) intensity(7719326.5000) scans(3428) rtinseconds(786) index(2193)
Title: 150825_TTSH_Patient_Plasma_48_Spectrum018484_scans__3428_RTINSECONDS=786
Data file L:\\Ard_TTSH\\T1D\\T150825_TTSH_Patient_Plasma_48.mgf

Click mouse within plot area to zoom in by factor of two about that point
Or, to Da
Label all possible matches Label matches used for scoring

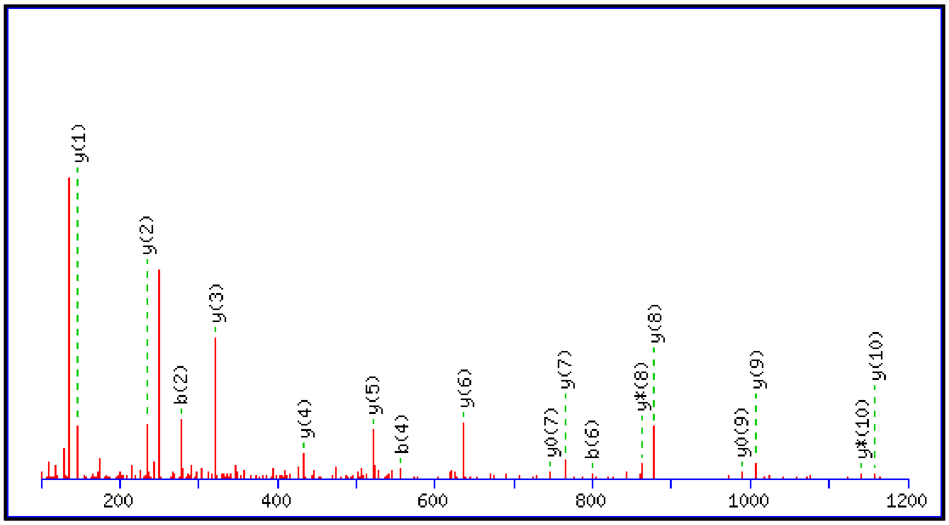

Monoisotopic mass of neutral peptide Mr(calc): 1433.5980
Variable modifications:
C3 : 4Trioxidation (CMWY)
Ions Score: 69 **Expect:** 2.2e-005
Matches : 17/116 fragment ions using 32 most intense peaks (help)

#	b	b^{++}	b*	b^{*++}	b^0	b^{0++}	Seq.	y	y^{++}	y*	y^{*++}	y^0	y^{0++}	#
1	164.0706	82.5389					Y							12
2	277.1547	139.0810					I	1271.5420	636.2746	1254.5154	627.7614	1253.5314	627.2693	11
3	428.1486	214.5779					C	1158.4579	579.7326	1141.4314	571.2193	1140.4474	570.7273	10
4	557.1912	279.0992			539.1806	270.0940	E	1007.4640	504.2356	990.4374	495.7224	989.4534	495.2304	9
5	671.2341	336.1207	654.2076	327.6074	653.2236	327.1154	N	878.4214	439.7143	861.3949	431.2011	860.4108	430.7091	8
6	799.2927	400.1500	782.2661	391.6367	781.2821	391.1447	Q	764.3785	382.6929	747.3519	374.1796	746.3679	373.6876	7
7	914.3196	457.6635	897.2931	449.1502	896.3091	448.6582	D	636.3199	318.6636	619.2933	310.1503	618.3093	309.6583	6
8	1001.3517	501.1795	984.3251	492.6662	983.3411	492.1742	S	521.2930	261.1501	504.2664	252.6368	503.2824	252.1448	5
9	1114.4357	557.7215	1097.4092	549.2082	1096.4252	548.7162	I	434.2609	217.6341	417.2344	209.1208	416.2504	208.6288	4
10	1201.4678	601.2375	1184.4412	592.7242	1183.4572	592.2322	S	321.1769	161.0921	304.1503	152.5788	303.1663	152.0868	3
11	1288.4998	644.7535	1271.4732	636.2403	1270.4892	635.7482	S	234.1448	117.5761	217.1183	109.0628	216.1343	108.5708	2
12							K	147.1128	74.0600	130.0863	65.5468			1

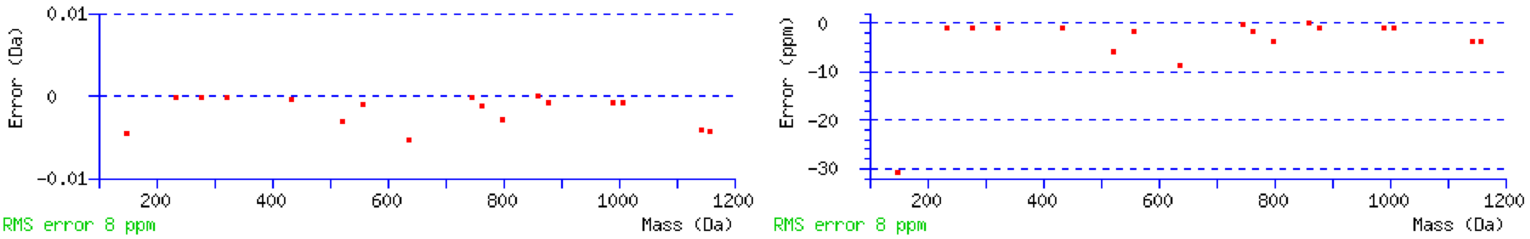

NCBI **BLAST** search of YICENQDSISSK
(Parameters: blastp, nr protein database, expect=20000, no filter, PAM30)
Other BLAST web gateways

All matches to this query

Score	Mr(calc):	Delta	Sequence
69.4	1433.5980	-0.0029	YICENQDSISSK
54.5	1433.5980	-0.0029	YICENQDSISSK

Mascot Search Results

Peptide View

MS/MS Fragmentation of **YICENQDSISSK**
Found in **sp|P02768|ALBU_HUMAN**, Serum albumin OS=Homo sapiens GN=ALB PE=1 SV=2

Match to Query 4911: 1433.595808 from(717.805180,2+) intensity(725976.6875) scans(7417) rtinseconds(1446) index(5907)
Title: 150808_TTSH_Patient_Plasma_50_Spectrum022008_scans__7417_RTINSECONDS=1446
Data file L:\\Ard_TTSH\\T1D\\T150808_TTSH_Patient_Plasma_50.mgf

Click mouse within plot area to zoom in by factor of two about that point
Or, Plot from 100 to 1050 Da Full range
Label all possible matches ○ Label matches used for scoring ⦿

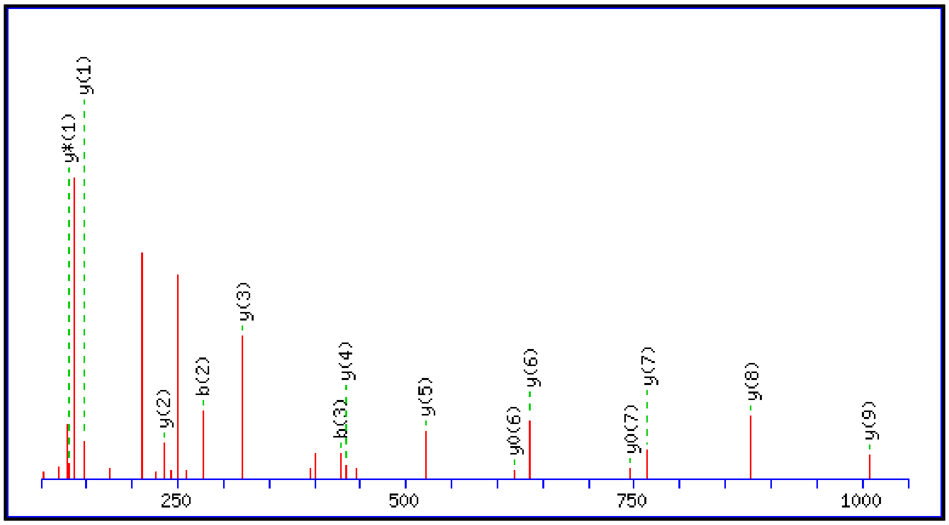

Monoisotopic mass of neutral peptide Mr(calc): 1433.5980
Variable modifications:
C3 : 4Trioxidation (CMWY)
Ions Score: 69 **Expect:** 2.2e-005
Matches : 14/116 fragment ions using 22 most intense peaks (help)

#	b	b^{++}	b*	b^{*++}	b^{0}	b^{0++}	Seq.	y	y^{++}	y*	y^{*++}	y^{0}	y^{0++}	#
1	164.0706	82.5389					Y							12
2	**277.1547**	139.0810					I	1271.5420	636.2746	1254.5154	627.7614	1253.5314	627.2693	11
3	**428.1486**	214.5779					C	1158.4579	579.7326	1141.4314	571.2193	1140.4474	570.7273	10
4	557.1912	279.0992			539.1806	270.0940	E	***1007.4640***	504.2356	990.4374	495.7224	989.4534	495.2304	9
5	671.2341	336.1207	654.2076	327.6074	653.2236	327.1154	N	***878.4214***	439.7143	861.3949	431.2011	860.4108	430.7091	8
6	799.2927	400.1500	782.2661	391.6367	781.2821	391.1447	Q	***764.3785***	382.6929	747.3519	374.1796	746.3679	373.6876	7
7	914.3196	457.6635	897.2931	449.1502	896.3091	448.6582	D	***636.3199***	318.6636	619.2933	310.1503	618.3093	309.6583	6
8	1001.3517	501.1795	984.3251	492.6662	983.3411	492.1742	S	***521.2930***	261.1501	504.2664	252.6368	503.2824	252.1448	5
9	1114.4357	557.7215	1097.4092	549.2082	1096.4252	548.7162	I	***434.2609***	217.6341	417.2344	209.1208	416.2504	208.6288	4
10	1201.4678	601.2375	1184.4412	592.7242	1183.4572	592.2322	S	***321.1769***	161.0921	304.1503	152.5788	303.1663	152.0868	3
11	1288.4998	644.7535	1271.4732	636.2403	1270.4892	635.7482	S	***234.1448***	117.5761	217.1183	109.0628	216.1343	108.5708	2
12							K	***147.1128***	74.0600	130.0863	65.5468			1

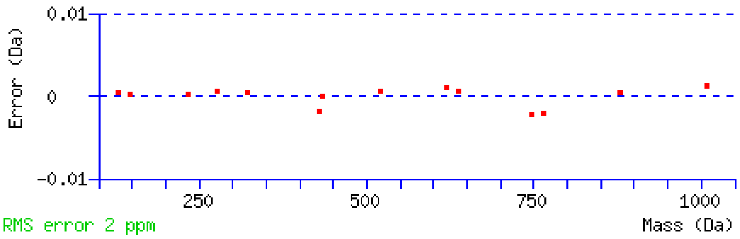

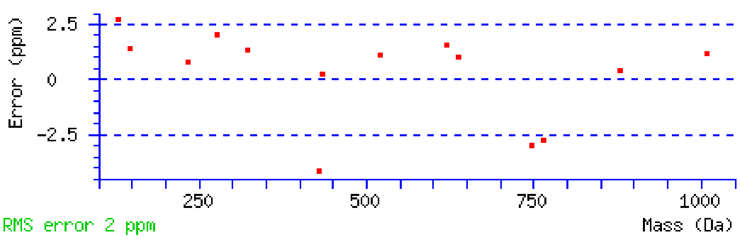

NCBI **BLAST** search of YICENQDSISSK
(Parameters: blastp, nr protein database, expect=20000, no filter, PAM30)
Other BLAST web gateways

All matches to this query

Score	Mr(calc):	Delta	Sequence
69.4	1433.5980	-0.0022	YICENQDSISSK
66.1	1433.5980	-0.0022	YICENQDSISSK

Mascot Search Results

Peptide View

MS/MS Fragmentation of **YICENQDSISSK**
Found in **sp|P02768|ALBU_HUMAN**, Serum albumin OS=Homo sapiens GN=ALB PE=1 SV=2

Match to Query 5231: 1433.601408 from(717.807980,2+) intensity(1594404.3750) scans(4818) rtinseconds(908) index(3592)
Title: N50801_TTSH_Patient_Plasma_53_Spectrum003867_scans__4818_RTINSECONDS=908
Data file L:\\Ard_TTSH\\TN1D\\TN50801_TTSH_Patient_Plasma_53.mgf

Click mouse within plot area to zoom in by factor of two about that point
Or, Plot from 100 to 1200 Da Full range
Label all possible matches ○ Label matches used for scoring ⦿

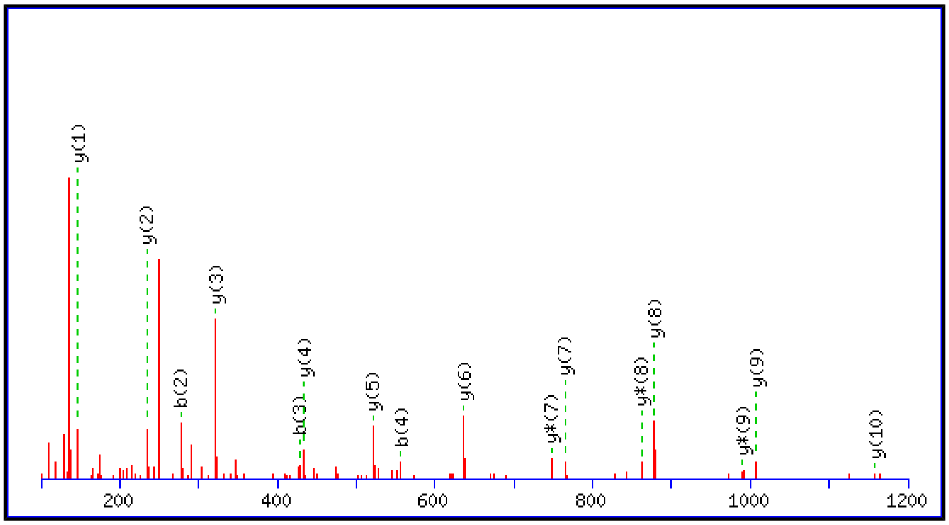

Monoisotopic mass of neutral peptide Mr(calc): 1433.5980
Variable modifications:
C3 : 4Trioxidation (CMWY)
Ions Score: 69 **Expect:** 2.8e-005
Matches : 16/116 fragment ions using 31 most intense peaks (help)

#	b	b^{++}	b*	b^{*++}	b^0	b^{0++}	Seq.	y	y^{++}	y*	y^{*++}	y^0	y^{0++}	#
1	164.0706	82.5389					Y							12
2	**277.1547**	139.0810					I	1271.5420	636.2746	1254.5154	627.7614	1253.5314	627.2693	11
3	**428.1486**	214.5779					C	***1158.4579***	579.7326	1141.4314	571.2193	1140.4474	570.7273	10
4	**557.1912**	279.0992			539.1806	270.0940	E	***1007.4640***	504.2356	990.4374	495.7224	989.4534	495.2304	9
5	671.2341	336.1207	654.2076	327.6074	653.2236	327.1154	N	***878.4214***	439.7143	861.3949	431.2011	860.4108	430.7091	8
6	799.2927	400.1500	782.2661	391.6367	781.2821	391.1447	Q	***764.3785***	382.6929	747.3519	374.1796	746.3679	373.6876	7
7	914.3196	457.6635	897.2931	449.1502	896.3091	448.6582	D	***636.3199***	318.6636	619.2933	310.1503	618.3093	309.6583	6
8	1001.3517	501.1795	984.3251	492.6662	983.3411	492.1742	S	***521.2930***	261.1501	504.2664	252.6368	503.2824	252.1448	5
9	1114.4357	557.7215	1097.4092	549.2082	1096.4252	548.7162	I	***434.2609***	217.6341	417.2344	209.1208	416.2504	208.6288	4
10	1201.4678	601.2375	1184.4412	592.7242	1183.4572	592.2322	S	***321.1769***	161.0921	304.1503	152.5788	303.1663	152.0868	3
11	1288.4998	644.7535	1271.4732	636.2403	1270.4892	635.7482	S	***234.1448***	117.5761	217.1183	109.0628	216.1343	108.5708	2
12							K	***147.1128***	74.0600	130.0863	65.5468			1

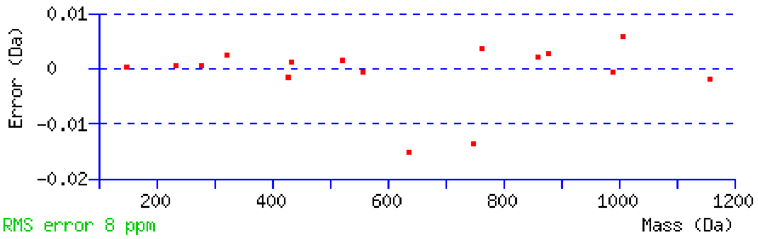

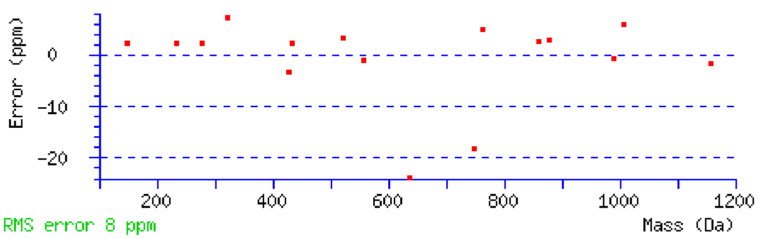

NCBI **BLAST** search of YICENQDSISSK
(Parameters: blastp, nr protein database, expect=20000, no filter, PAM30)
Other BLAST web gateways

All matches to this query

Score	Mr(calc):	Delta	Sequence
68.5	1433.5980	0.0034	YICENQDSISSK
53.2	1433.5980	0.0034	YICENQDSISSK

Mascot: http://www.matrixscience.com/

MATRIX SCIENCE Mascot Search Results

Peptide View

MS/MS Fragmentation of **YICENQDSISSK**
Found in **sp|P02768|ALBU_HUMAN**, Serum albumin OS=Homo sapiens GN=ALB PE=1 SV=2

Match to Query 4045: 1433.597028 from(717.805790,2+) intensity(816902.9375) scans(6325) rtinseconds(1506) index(4042)
Title: 150808_TTSH_Patient_Plasma_53_Spectrum017537_scans__6325_RTINSECONDS=1506
Data file L:\\Ard_TTSH\\T1D\\T150808_TTSH_Patient_Plasma_53.mgf

Click mouse within plot area to zoom in by factor of two about that point
Or, Plot from 100 to 1050 Da Full range
Label all possible matches ○ Label matches used for scoring ⊙

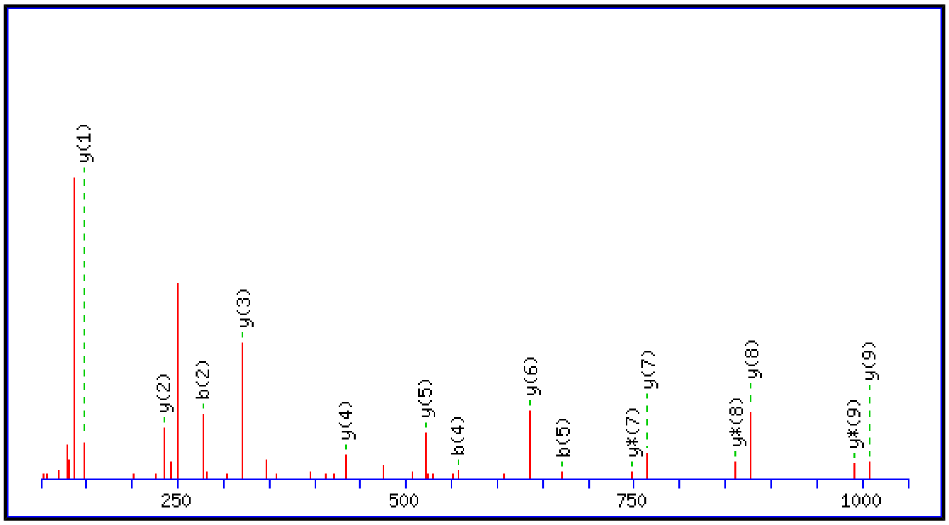

Monoisotopic mass of neutral peptide Mr(calc): 1433.5980
Variable modifications:
C3 : 4Trioxidation (CMWY)
Ions Score: 68 **Expect:** 2.8e-005
Matches : 15/116 fragment ions using 24 most intense peaks (help)

#	b	b^{++}	b*	b^{*++}	b^0	b^{0++}	Seq.	y	y^{++}	y*	y^{*++}	y^0	y^{0++}	#
1	164.0706	82.5389					Y							12
2	**277.1547**	139.0810					I	1271.5420	636.2746	1254.5154	627.7614	1253.5314	627.2693	11
3	428.1486	214.5779					C	1158.4579	579.7326	1141.4314	571.2193	1140.4474	570.7273	10
4	**557.1912**	279.0992			539.1806	270.0940	E	***1007.4640***	504.2356	990.4374	495.7224	989.4534	495.2304	9
5	**671.2341**	336.1207	654.2076	327.6074	653.2236	327.1154	N	***878.4214***	439.7143	861.3949	431.2011	860.4108	430.7091	8
6	799.2927	400.1500	782.2661	391.6367	781.2821	391.1447	Q	***764.3785***	382.6929	747.3519	374.1796	746.3679	373.6876	7
7	914.3196	457.6635	897.2931	449.1502	896.3091	448.6582	D	***636.3199***	318.6636	619.2933	310.1503	618.3093	309.6583	6
8	1001.3517	501.1795	984.3251	492.6662	983.3411	492.1742	S	***521.2930***	261.1501	504.2664	252.6368	503.2824	252.1448	5
9	1114.4357	557.7215	1097.4092	549.2082	1096.4252	548.7162	I	***434.2609***	217.6341	417.2344	209.1208	416.2504	208.6288	4
10	1201.4678	601.2375	1184.4412	592.7242	1183.4572	592.2322	S	***321.1769***	161.0921	304.1503	152.5788	303.1663	152.0868	3
11	1288.4998	644.7535	1271.4732	636.2403	1270.4892	635.7482	S	***234.1448***	117.5761	217.1183	109.0628	216.1343	108.5708	2
12							K	***147.1128***	74.0600	130.0863	65.5468			1

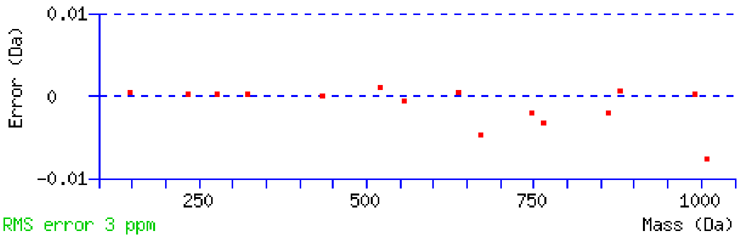

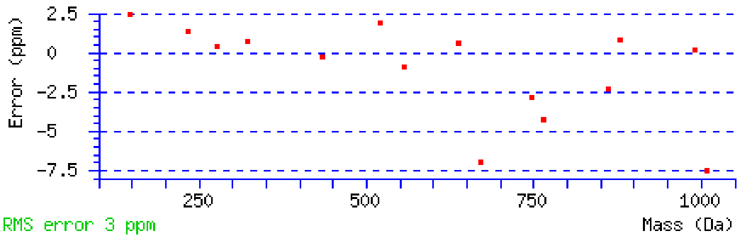

NCBI **BLAST** search of YICENQDSISSK
(Parameters: blastp, nr protein database, expect=20000, no filter, PAM30)
Other BLAST web gateways

All matches to this query

Score	Mr(calc):	Delta	Sequence
68.4	1433.5980	-0.0010	YICENQDSISSK
65.3	1433.5980	-0.0010	YICENQDSISSK
1.2	1431.6041	1.9929	XEESENEFYIK

Mascot Search Results

Peptide View

MS/MS Fragmentation of **YICENQDSISSK**
Found in **sp|P02768|ALBU_HUMAN**, Serum albumin OS=Homo sapiens GN=ALB PE=1 SV=2

Match to Query 3113: 1433.596168 from(717.805360,2+) intensity(5413672.5000) scans(3501) rtinseconds(768) index(2297)
Title: 150825_TTSH_Patient_Plasma_49_Spectrum018652_scans__3501_RTINSECONDS=768
Data file L:\\Ard_TTSH\\T1D\\T150825_TTSH_Patient_Plasma_49.mgf

Click mouse within plot area to zoom in by factor of two about that point

Or, to Da

Label all possible matches ○ Label matches used for scoring ⊙

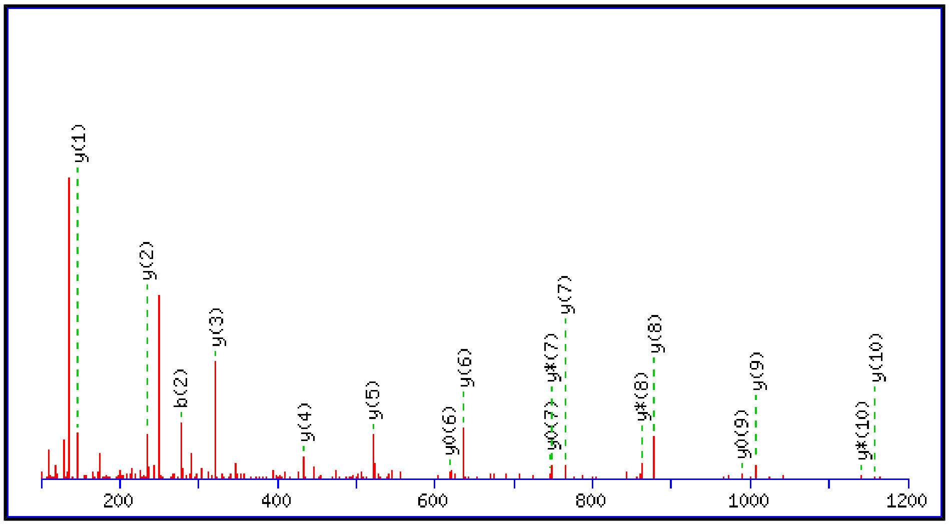

Monoisotopic mass of neutral peptide Mr(calc): 1433.5980
Variable modifications:
C3 : 4Trioxidation (CMWY)
Ions Score: 67 **Expect:** 3.4e-005
Matches : 17/116 fragment ions using 33 most intense peaks (help)

#	b	b++	b*	b*++	b0	b0++	Seq.	y	y++	y*	y*++	y0	y0++	#
1	164.0706	82.5389					Y							12
2	**277.1547**	139.0810					I	1271.5420	636.2746	1254.5154	627.7614	1253.5314	627.2693	11
3	428.1486	214.5779					C	***1158.4579***	579.7326	1141.4314	571.2193	1140.4474	570.7273	10
4	557.1912	279.0992			539.1806	270.0940	E	***1007.4640***	504.2356	990.4374	495.7224	989.4534	495.2304	9
5	671.2341	336.1207	654.2076	327.6074	653.2236	327.1154	N	***878.4214***	439.7143	861.3949	431.2011	860.4108	430.7091	8
6	799.2927	400.1500	782.2661	391.6367	781.2821	391.1447	Q	***764.3785***	382.6929	747.3519	374.1796	746.3679	373.6876	7
7	914.3196	457.6635	897.2931	449.1502	896.3091	448.6582	D	***636.3199***	318.6636	619.2933	310.1503	618.3093	309.6583	6
8	1001.3517	501.1795	984.3251	492.6662	983.3411	492.1742	S	***521.2930***	261.1501	504.2664	252.6368	503.2824	252.1448	5
9	1114.4357	557.7215	1097.4092	549.2082	1096.4252	548.7162	I	***434.2609***	217.6341	417.2344	209.1208	416.2504	208.6288	4
10	1201.4678	601.2375	1184.4412	592.7242	1183.4572	592.2322	S	***321.1769***	161.0921	304.1503	152.5788	303.1663	152.0868	3
11	1288.4998	644.7535	1271.4732	636.2403	1270.4892	635.7482	S	***234.1448***	117.5761	217.1183	109.0628	216.1343	108.5708	2
12							K	***147.1128***	74.0600	130.0863	65.5468			1

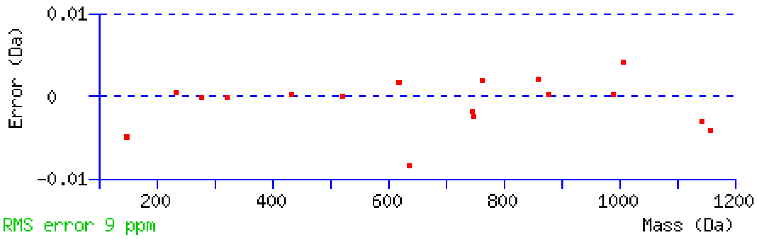

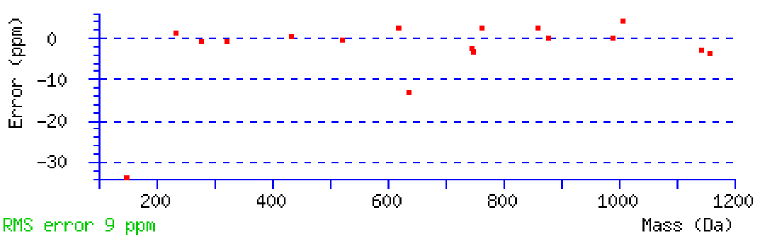

NCBI **BLAST** search of YICENQDSISSK
(Parameters: blastp, nr protein database, expect=20000, no filter, PAM30)
Other BLAST web gateways

All matches to this query

Score	Mr(calc):	Delta	Sequence
67.5	1433.5980	-0.0018	YICENQDSISSK
49.7	1433.5980	-0.0018	YICENQDSISSK

Mascot Search Results

Peptide View

MS/MS Fragmentation of **YICENQDSISSK**
Found in **sp|P02768|ALBU_HUMAN**, Serum albumin OS=Homo sapiens GN=ALB PE=1 SV=2

Match to Query 3113: 1433.596168 from(717.805360,2+) intensity(5413672.5000) scans(3501) rtinseconds(768) index(2297)
Title: N50825_TTSH_Patient_Plasma_49_Spectrum002599_scans__3501_RTINSECONDS=768
Data file L:\\Ard_TTSH\\TN1D\\TN50825_TTSH_Patient_Plasma_49.mgf

Click mouse within plot area to zoom in by factor of two about that point
Or, to Da
Label all possible matches Label matches used for scoring

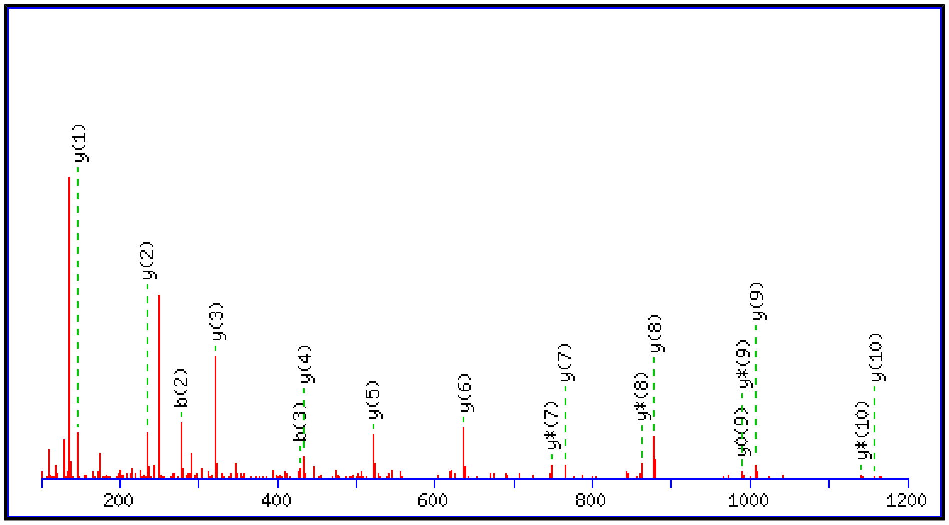

Monoisotopic mass of neutral peptide Mr(calc): 1433.5980
Variable modifications:
C3 : 4Trioxidation (CMWY)
Ions Score: 67 **Expect:** 3.6e-005
Matches : 17/116 fragment ions using 33 most intense peaks (help)

#	b	b^{++}	b*	b^{*++}	b^{0}	b^{0++}	Seq.	y	y^{++}	y*	y^{*++}	y^{0}	y^{0++}	#
1	164.0706	82.5389					Y							12
2	**277.1547**	139.0810					I	1271.5420	636.2746	1254.5154	627.7614	1253.5314	627.2693	11
3	**428.1486**	214.5779					C	***1158.4579***	579.7326	1141.4314	571.2193	1140.4474	570.7273	10
4	557.1912	279.0992			539.1806	270.0940	E	***1007.4640***	504.2356	990.4374	495.7224	989.4534	495.2304	9
5	671.2341	336.1207	654.2076	327.6074	653.2236	327.1154	N	***878.4214***	439.7143	861.3949	431.2011	860.4108	430.7091	8
6	799.2927	400.1500	782.2661	391.6367	781.2821	391.1447	Q	***764.3785***	382.6929	747.3519	374.1796	746.3679	373.6876	7
7	914.3196	457.6635	897.2931	449.1502	896.3091	448.6582	D	***636.3199***	318.6636	619.2933	310.1503	618.3093	309.6583	6
8	1001.3517	501.1795	984.3251	492.6662	983.3411	492.1742	S	***521.2930***	261.1501	504.2664	252.6368	503.2824	252.1448	5
9	1114.4357	557.7215	1097.4092	549.2082	1096.4252	548.7162	I	***434.2609***	217.6341	417.2344	209.1208	416.2504	208.6288	4
10	1201.4678	601.2375	1184.4412	592.7242	1183.4572	592.2322	S	***321.1769***	161.0921	304.1503	152.5788	303.1663	152.0868	3
11	1288.4998	644.7535	1271.4732	636.2403	1270.4892	635.7482	S	***234.1448***	117.5761	217.1183	109.0628	216.1343	108.5708	2
12							K	***147.1128***	74.0600	130.0863	65.5468			1

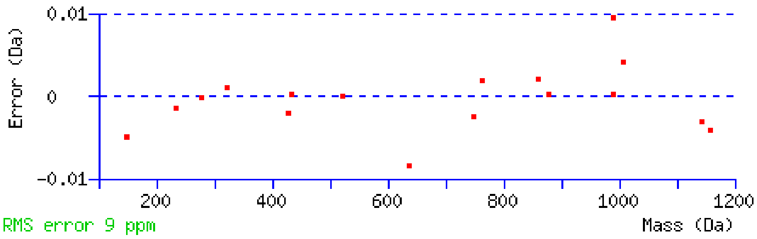

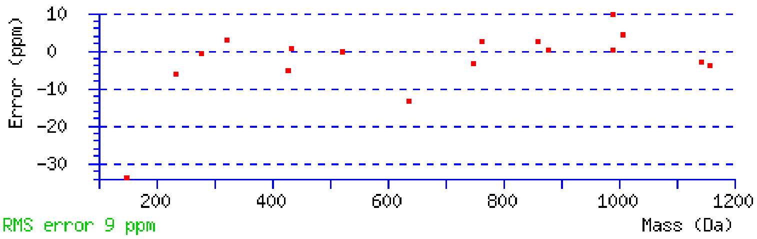

NCBI **BLAST** search of YICENQDSISSK
(Parameters: blastp, nr protein database, expect=20000, no filter, PAM30)
Other BLAST web gateways

All matches to this query

Score	Mr(calc):	Delta	Sequence
67.2	1433.5980	-0.0018	YICENQDSISSK
49.5	1433.5980	-0.0018	YICENQDSISSK

Mascot: http://www.matrixscience.com/

Mascot Search Results

Peptide View

MS/MS Fragmentation of **YICENQDSISSK**
Found in **sp|P02768|ALBU_HUMAN**, Serum albumin OS=Homo sapiens GN=ALB PE=1 SV=2

Match to Query 6506: 1433.596168 from(717.805360,2+) intensity(1540714.3750) scans(4764) rtinseconds(903) index(4095)
Title: 150818_TTSH_Patient_Plasma_79_Spectrum021262_scans__4764_RTINSECONDS=903
Data file L:\\Ard_TTSH\\T1D\\T150818_TTSH_Patient_Plasma_79.mgf

Click mouse within plot area to zoom in by factor of two about that point
Or, Plot from 100 to 1200 Da Full range
Label all possible matches ○ Label matches used for scoring ⊙

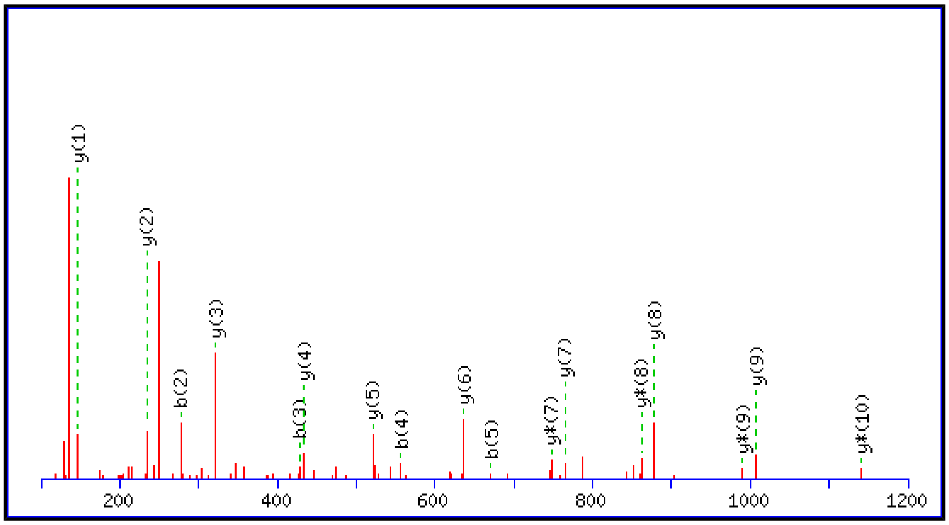

Monoisotopic mass of neutral peptide Mr(calc): 1433.5980
Variable modifications:
C3 : 4Trioxidation (CMWY)
Ions Score: 67 **Expect:** 4e-005
Matches : 17/116 fragment ions using 27 most intense peaks (help)

#	b	b^{++}	b*	b^{*++}	b^0	b^{0++}	Seq.	y	y^{++}	y*	y^{*++}	y^0	y^{0++}	#
1	164.0706	82.5389					Y							12
2	**277.1547**	139.0810					I	1271.5420	636.2746	1254.5154	627.7614	1253.5314	627.2693	11
3	**428.1486**	214.5779					C	1158.4579	579.7326	1141.4314	571.2193	1140.4474	570.7273	10
4	**557.1912**	279.0992			539.1806	270.0940	E	***1007.4640***	504.2356	990.4374	495.7224	989.4534	495.2304	9
5	**671.2341**	336.1207	654.2076	327.6074	653.2236	327.1154	N	***878.4214***	439.7143	861.3949	431.2011	860.4108	430.7091	8
6	799.2927	400.1500	782.2661	391.6367	781.2821	391.1447	Q	***764.3785***	382.6929	747.3519	374.1796	746.3679	373.6876	7
7	914.3196	457.6635	897.2931	449.1502	896.3091	448.6582	D	***636.3199***	318.6636	619.2933	310.1503	618.3093	309.6583	6
8	1001.3517	501.1795	984.3251	492.6662	983.3411	492.1742	S	***521.2930***	261.1501	504.2664	252.6368	503.2824	252.1448	5
9	1114.4357	557.7215	1097.4092	549.2082	1096.4252	548.7162	I	***434.2609***	217.6341	417.2344	209.1208	416.2504	208.6288	4
10	1201.4678	601.2375	1184.4412	592.7242	1183.4572	592.2322	S	***321.1769***	161.0921	304.1503	152.5788	303.1663	152.0868	3
11	1288.4998	644.7535	1271.4732	636.2403	1270.4892	635.7482	S	***234.1448***	117.5761	217.1183	109.0628	216.1343	108.5708	2
12							K	***147.1128***	74.0600	130.0863	65.5468			1

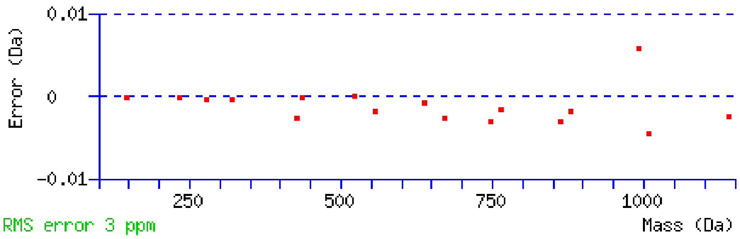

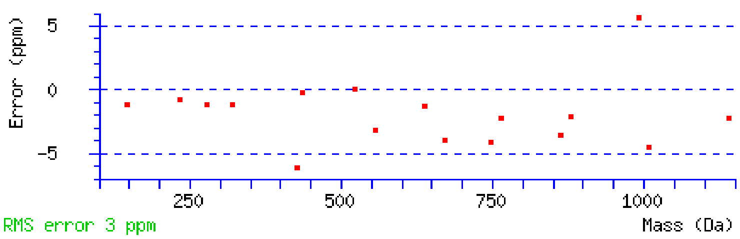

NCBI **BLAST** search of YICENQDSISSK
(Parameters: blastp, nr protein database, expect=20000, no filter, PAM30)
Other BLAST web gateways

All matches to this query

Score	Mr(calc):	Delta	Sequence
66.7	1433.5980	-0.0018	YICENQDSISSK
61.6	1433.5980	-0.0018	YICENQDSISSK

Mascot: http://www.matrixscience.com/

Mascot Search Results

Peptide View

MS/MS Fragmentation of **YICENQDSISSK**
Found in **sp|P02768|ALBU_HUMAN**, Serum albumin OS=Homo sapiens GN=ALB PE=1 SV=2

Match to Query 5139: 1433.596168 from(717.805360,2+) intensity(830344.1875) scans(5423) rtinseconds(1272) index(3451)
Title: N50801_TTSH_Patient_Plasma_66_Spectrum003676_scans__5423_RTINSECONDS=1272
Data file L:\\Ard_TTSH\\TN1D\\TN50801_TTSH_Patient_Plasma_66.mgf

Click mouse within plot area to zoom in by factor of two about that point

Or, to Da

Label all possible matches Label matches used for scoring

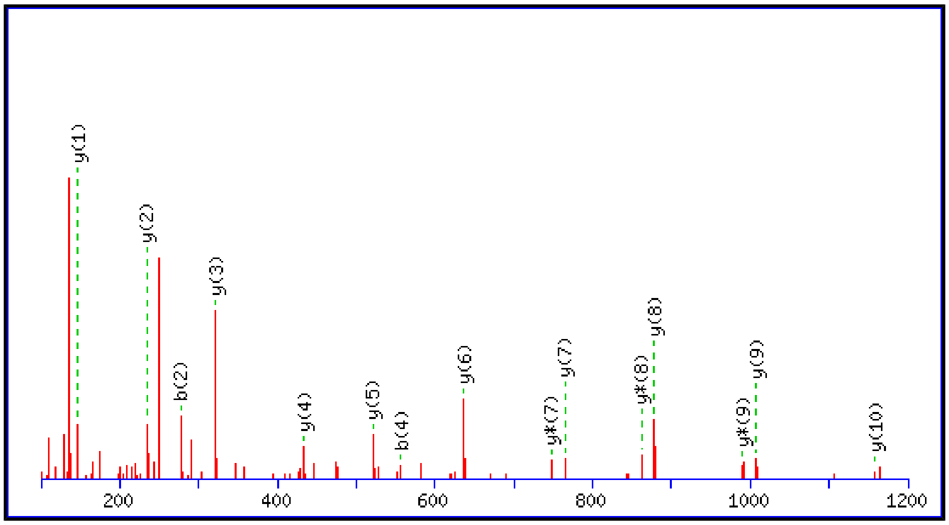

Monoisotopic mass of neutral peptide Mr(calc): 1433.5980
Variable modifications:
C3 : 4Trioxidation (CMWY)
Ions Score: 66 **Expect:** 4.4e-005
Matches : 15/116 fragment ions using 31 most intense peaks (help)

#	b	b^{++}	b*	b*$^{++}$	b^{0}	b^{0++}	Seq.	y	y^{++}	y*	y*$^{++}$	y^{0}	y^{0++}	#
1	164.0706	82.5389					Y							12
2	277.1547	139.0810					I	1271.5420	636.2746	1254.5154	627.7614	1253.5314	627.2693	11
3	428.1486	214.5779					C	1158.4579	579.7326	1141.4314	571.2193	1140.4474	570.7273	10
4	557.1912	279.0992			539.1806	270.0940	E	1007.4640	504.2356	990.4374	495.7224	989.4534	495.2304	9
5	671.2341	336.1207	654.2076	327.6074	653.2236	327.1154	N	878.4214	439.7143	861.3949	431.2011	860.4108	430.7091	8
6	799.2927	400.1500	782.2661	391.6367	781.2821	391.1447	Q	764.3785	382.6929	747.3519	374.1796	746.3679	373.6876	7
7	914.3196	457.6635	897.2931	449.1502	896.3091	448.6582	D	636.3199	318.6636	619.2933	310.1503	618.3093	309.6583	6
8	1001.3517	501.1795	984.3251	492.6662	983.3411	492.1742	S	521.2930	261.1501	504.2664	252.6368	503.2824	252.1448	5
9	1114.4357	557.7215	1097.4092	549.2082	1096.4252	548.7162	I	434.2609	217.6341	417.2344	209.1208	416.2504	208.6288	4
10	1201.4678	601.2375	1184.4412	592.7242	1183.4572	592.2322	S	321.1769	161.0921	304.1503	152.5788	303.1663	152.0868	3
11	1288.4998	644.7535	1271.4732	636.2403	1270.4892	635.7482	S	234.1448	117.5761	217.1183	109.0628	216.1343	108.5708	2
12							K	147.1128	74.0600	130.0863	65.5468			1

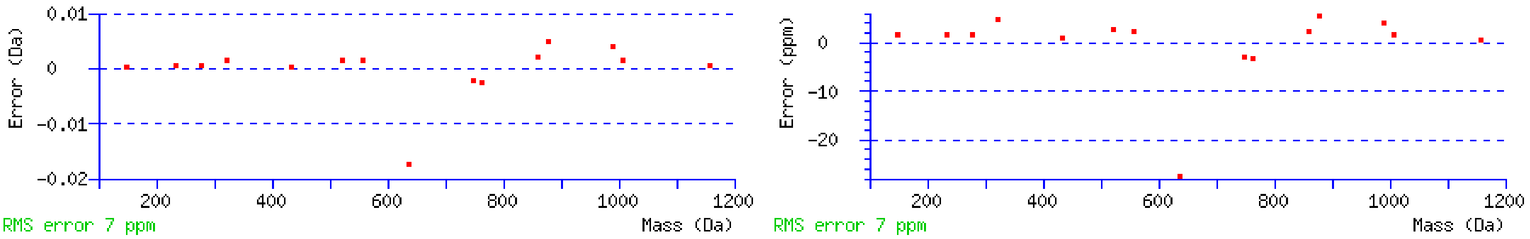

NCBI **BLAST** search of YICENQDSISSK
(Parameters: blastp, nr protein database, expect=20000, no filter, PAM30)
Other BLAST web gateways

All matches to this query

Score	Mr(calc):	Delta	Sequence
66.3	1433.5980	-0.0018	YICENQDSISSK
50.5	1433.5980	-0.0018	YICENQDSISSK

Mascot Search Results

Peptide View

MS/MS Fragmentation of **YICENQDSISSK**
Found in **sp|P02768|ALBU_HUMAN**, Serum albumin OS=Homo sapiens GN=ALB PE=1 SV=2

Match to Query 4653: 1442.635108 from(722.324830,2+) intensity(19177242.0000) scans(4583) rtinseconds(991) index(3380)
Title: 150808_TTSH_Patient_Plasma_42_Spectrum018912_scans__4583_RTINSECONDS=991
Data file L:\\Ard_TTSH\\T1D\\T150808_TTSH_Patient_Plasma_42.mgf

Click mouse within plot area to zoom in by factor of two about that point
Or, Plot from 100 to 1300 Da Full range
Label all possible matches ○ Label matches used for scoring ⊙

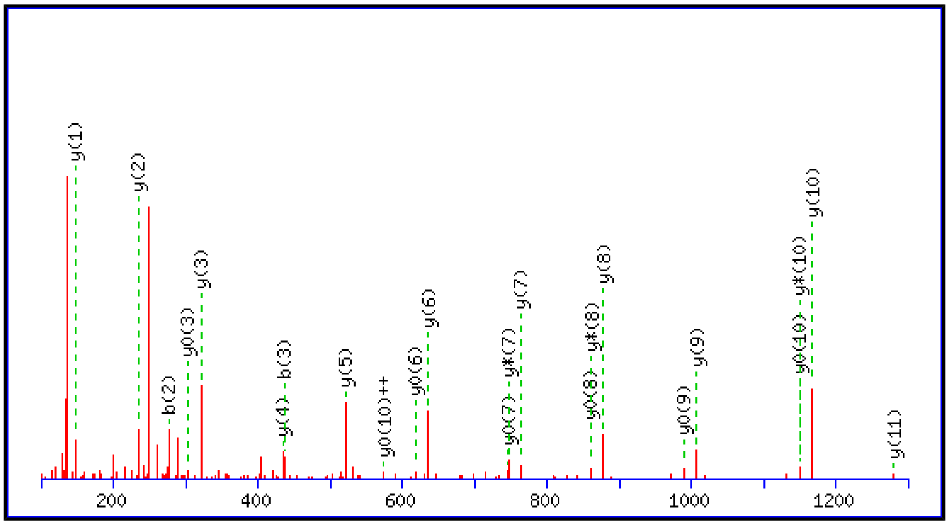

Monoisotopic mass of neutral peptide Mr(calc): 1442.6347
Variable modifications:
C3 : Carbamidomethyl (C)
Ions Score: 97 **Expect:** 4.5e-008
Matches : 23/116 fragment ions using 32 most intense peaks (help)

#	b	b^{++}	b*	b^{*++}	b^0	b^{0++}	Seq.	y	y^{++}	y*	y^{*++}	y^0	y^{0++}	#
1	164.0706	82.5389					Y							12
2	277.1547	139.0810					I	1280.5787	640.7930	1263.5522	632.2797	1262.5681	631.7877	11
3	437.1853	219.0963					C	1167.4946	584.2510	1150.4681	575.7377	1149.4841	575.2457	10
4	566.2279	283.6176			548.2173	274.6123	E	1007.4640	504.2356	990.4374	495.7224	989.4534	495.2304	9
5	680.2708	340.6391	663.2443	332.1258	662.2603	331.6338	N	878.4214	439.7143	861.3949	431.2011	860.4108	430.7091	8
6	808.3294	404.6683	791.3029	396.1551	790.3189	395.6631	Q	764.3785	382.6929	747.3519	374.1796	746.3679	373.6876	7
7	923.3564	462.1818	906.3298	453.6685	905.3458	453.1765	D	636.3199	318.6636	619.2933	310.1503	618.3093	309.6583	6
8	1010.3884	505.6978	993.3618	497.1846	992.3778	496.6925	S	521.2930	261.1501	504.2664	252.6368	503.2824	252.1448	5
9	1123.4725	562.2399	1106.4459	553.7266	1105.4619	553.2346	I	434.2609	217.6341	417.2344	209.1208	416.2504	208.6288	4
10	1210.5045	605.7559	1193.4779	597.2426	1192.4939	596.7506	S	321.1769	161.0921	304.1503	152.5788	303.1663	152.0868	3
11	1297.5365	649.2719	1280.5100	640.7586	1279.5259	640.2666	S	234.1448	117.5761	217.1183	109.0628	216.1343	108.5708	2
12							K	147.1128	74.0600	130.0863	65.5468			1

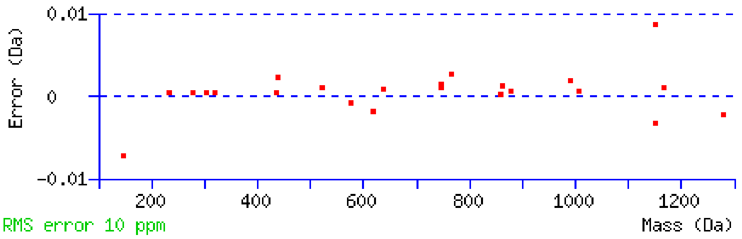

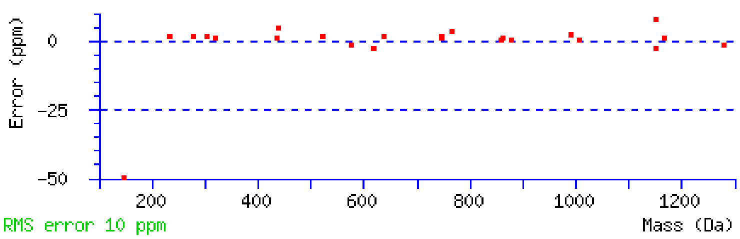

NCBI **BLAST** search of YICENQDSISSK
(Parameters: blastp, nr protein database, expect=20000, no filter, PAM30)
Other BLAST web gateways

All matches to this query

Score	Mr(calc):	Delta	Sequence
97.0	1442.6347	0.0004	YICENQDSISSK
2.9	1441.6217	1.0134	EKPYKCEECEK
1.7	1442.6282	0.0069	YLCMATNAAGTDR

Mascot: http://www.matrixscience.com/

Mascot Search Results

Peptide View

MS/MS Fragmentation of **YICENQDSISSK**
Found in **sp|P02768|ALBU_HUMAN**, Serum albumin OS=Homo sapiens GN=ALB PE=1 SV=2

Match to Query 6822: 1490.617768 from(746.316160,2+) intensity(1596442.5000) scans(7958) rtinseconds(1539) index(6458)
Title: 150801_TTSH_Patient_Plasma_81_Spectrum022576_scans__7958_RTINSECONDS=1539
Data file L:\\Ard_TTSH\\T1D\\T150801_TTSH_Patient_Plasma_81.mgf

Click mouse within plot area to zoom in by factor of two about that point
Or, Plot from 100 to 1200 Da Full range
Label all possible matches ○ Label matches used for scoring ⊙

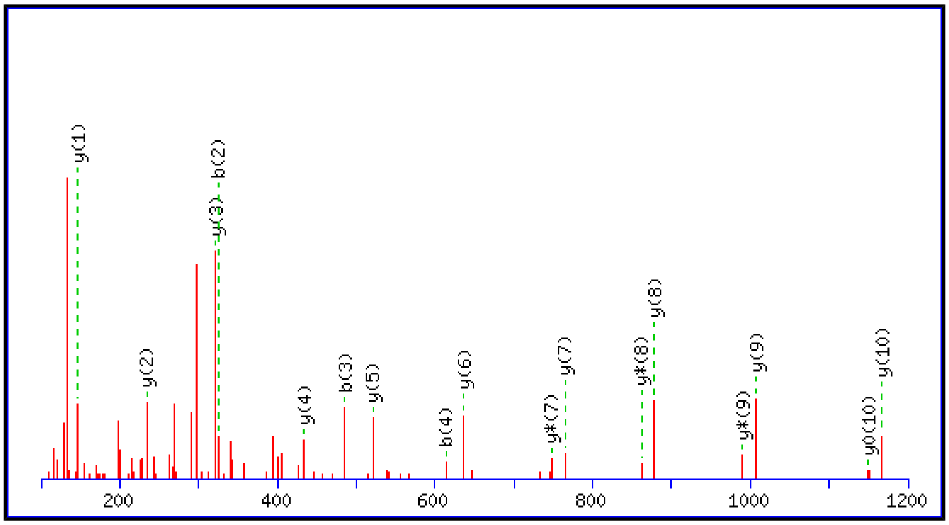

Monoisotopic mass of neutral peptide Mr(calc): 1490.6195
Variable modifications:
Y1 : 4Trioxidation (CMWY)
C3 : Carbamidomethyl (C)
Ions Score: 96 **Expect:** 3.7e-008
Matches : 17/116 fragment ions using 20 most intense peaks (help)

#	b	b^{++}	b*	b^{*++}	b^{0}	b^{0++}	Seq.	y	y^{++}	y*	y^{*++}	y^{0}	y^{0++}	#
1	212.0553	106.5313					Y							12
2	**325.1394**	163.0733					I	1280.5787	640.7930	1263.5522	632.2797	1262.5681	631.7877	11
3	**485.1701**	243.0887					C	***1167.4946***	584.2510	1150.4681	575.7377	1149.4841	575.2457	10
4	**614.2127**	307.6100			596.2021	298.6047	E	***1007.4640***	504.2356	990.4374	495.7224	989.4534	495.2304	9
5	728.2556	364.6314	711.2290	356.1182	710.2450	355.6261	N	***878.4214***	439.7143	861.3949	431.2011	860.4108	430.7091	8
6	856.3142	428.6607	839.2876	420.1474	838.3036	419.6554	Q	***764.3785***	382.6929	747.3519	374.1796	746.3679	373.6876	7
7	971.3411	486.1742	954.3146	477.6609	953.3305	477.1689	D	***636.3199***	318.6636	619.2933	310.1503	618.3093	309.6583	6
8	1058.3731	529.6902	1041.3466	521.1769	1040.3626	520.6849	S	***521.2930***	261.1501	504.2664	252.6368	503.2824	252.1448	5
9	1171.4572	586.2322	1154.4306	577.7190	1153.4466	577.2270	I	***434.2609***	217.6341	417.2344	209.1208	416.2504	208.6288	4
10	1258.4892	629.7482	1241.4627	621.2350	1240.4787	620.7430	S	***321.1769***	161.0921	304.1503	152.5788	303.1663	152.0868	3
11	1345.5213	673.2643	1328.4947	664.7510	1327.5107	664.2590	S	***234.1448***	117.5761	217.1183	109.0628	216.1343	108.5708	2
12							K	***147.1128***	74.0600	130.0863	65.5468			1

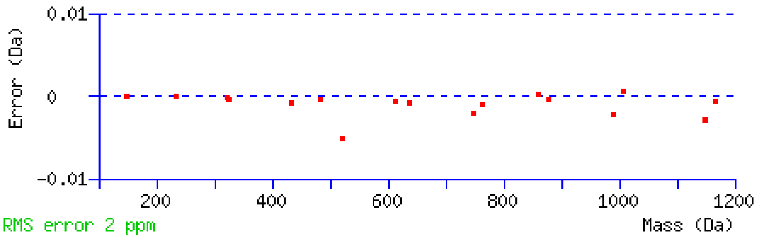

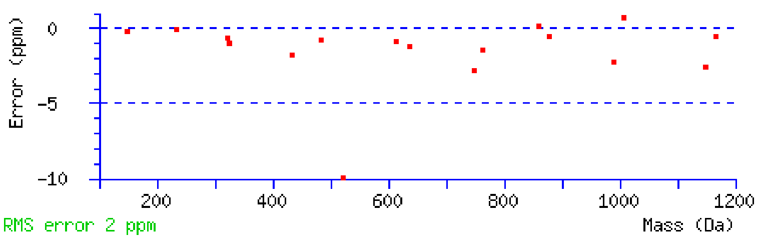

NCBI **BLAST** search of YICENQDSISSK
(Parameters: blastp, nr protein database, expect=20000, no filter, PAM30)
Other BLAST web gateways

All matches to this query

Score	Mr(calc):	Delta	Sequence
96.5	1490.6195	-0.0017	YICENQDSISSK

**Mascot:** http://www.matrixscience.com/

Mascot Search Results

Peptide View

MS/MS Fragmentation of **YICENQDSISSK**
Found in **sp|P02768|ALBU_HUMAN**, Serum albumin OS=Homo sapiens GN=ALB PE=1 SV=2

Match to Query 4351: 1442.634748 from(722.324650,2+) intensity(5483187.5000) scans(3271) rtinseconds(857) index(1871)
Title: 150801_TTSH_Patient_Plasma_82_Spectrum016704_scans__3271_RTINSECONDS=857
Data file L:\\Ard_TTSH\\T1D\\T150801_TTSH_Patient_Plasma_82.mgf

Click mouse within plot area to zoom in by factor of two about that point
Or, Plot from 100 to 1300 Da Full range
Label all possible matches ○ Label matches used for scoring ⊙

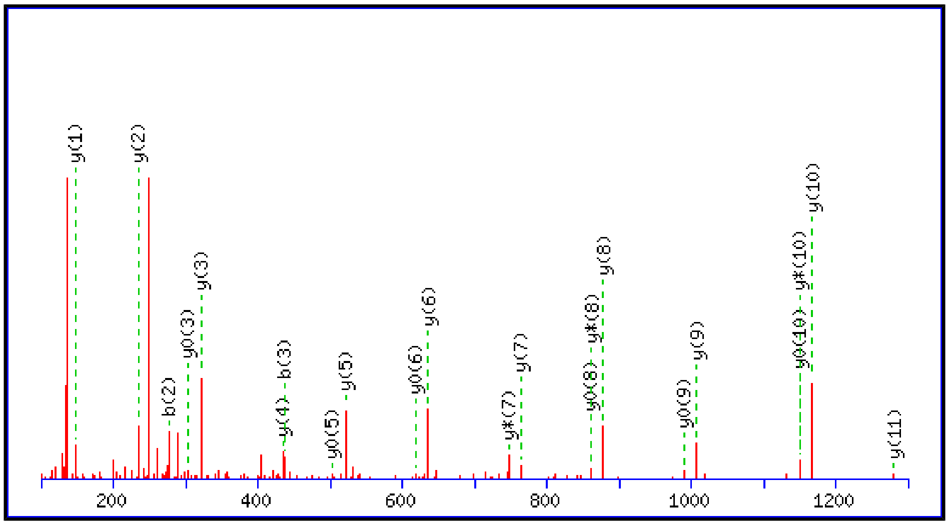

Monoisotopic mass of neutral peptide Mr(calc): 1442.6347
Variable modifications:
C3 : Carbamidomethyl (C)
Ions Score: 94 **Expect:** 8.2e-008
Matches : 22/116 fragment ions using 32 most intense peaks (help)

#	b	b^{++}	b*	b^{*++}	b^{0}	b^{0++}	Seq.	y	y^{++}	y*	y^{*++}	y^{0}	y^{0++}	#
1	164.0706	82.5389					Y							12
2	277.1547	139.0810					I	1280.5787	640.7930	1263.5522	632.2797	1262.5681	631.7877	11
3	437.1853	219.0963					C	1167.4946	584.2510	1150.4681	575.7377	1149.4841	575.2457	10
4	566.2279	283.6176			548.2173	274.6123	E	1007.4640	504.2356	990.4374	495.7224	989.4534	495.2304	9
5	680.2708	340.6391	663.2443	332.1258	662.2603	331.6338	N	878.4214	439.7143	861.3949	431.2011	860.4108	430.7091	8
6	808.3294	404.6683	791.3029	396.1551	790.3189	395.6631	Q	764.3785	382.6929	747.3519	374.1796	746.3679	373.6876	7
7	923.3564	462.1818	906.3298	453.6685	905.3458	453.1765	D	636.3199	318.6636	619.2933	310.1503	618.3093	309.6583	6
8	1010.3884	505.6978	993.3618	497.1846	992.3778	496.6925	S	521.2930	261.1501	504.2664	252.6368	503.2824	252.1448	5
9	1123.4725	562.2399	1106.4459	553.7266	1105.4619	553.2346	I	434.2609	217.6341	417.2344	209.1208	416.2504	208.6288	4
10	1210.5045	605.7559	1193.4779	597.2426	1192.4939	596.7506	S	321.1769	161.0921	304.1503	152.5788	303.1663	152.0868	3
11	1297.5365	649.2719	1280.5100	640.7586	1279.5259	640.2666	S	234.1448	117.5761	217.1183	109.0628	216.1343	108.5708	2
12							K	147.1128	74.0600	130.0863	65.5468			1

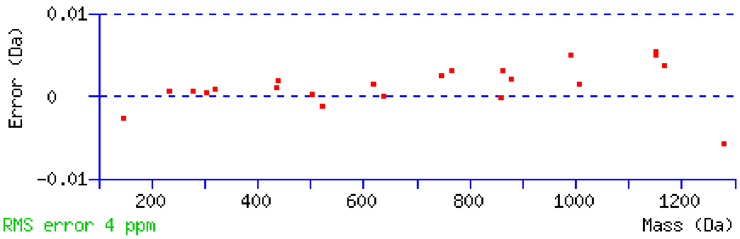

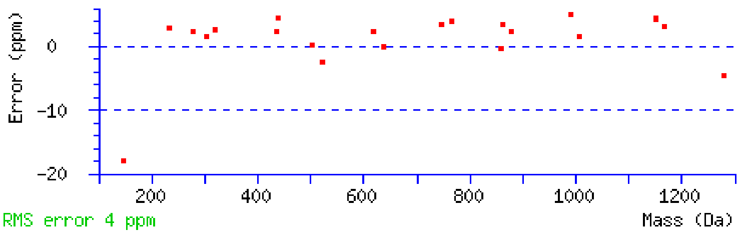

NCBI **BLAST** search of YICENQDSISSK
(Parameters: blastp, nr protein database, expect=20000, no filter, PAM30)
Other BLAST web gateways

All matches to this query

Score	Mr(calc):	Delta	Sequence
94.3	1442.6347	0.0000	YICENQDSISSK
3.1	1441.6217	1.0130	EKPYKCEECEK
1.8	1442.6282	0.0065	YLCMATNAAGTDR

Mascot: http://www.matrixscience.com/

Mascot Search Results

Peptide View

MS/MS Fragmentation of **YICENQDSISSK**
Found in **sp|P02768|ALBU_HUMAN**, Serum albumin OS=Homo sapiens GN=ALB PE=1 SV=2

Match to Query 5198: 1442.632548 from(722.323550,2+) intensity(294534784.0000) scans(2808) rtinseconds(530) index(2347)
Title: 150801_TTSH_Patient_Plasma_43_Spectrum019232_scans__2808_RTINSECONDS=530
Data file L:\\Ard_TTSH\\T1D\\T150801_TTSH_Patient_Plasma_43.mgf

Click mouse within plot area to zoom in by factor of two about that point
Or, Plot from 100 to 1300 Da Full range
Label all possible matches ○ Label matches used for scoring ⊙

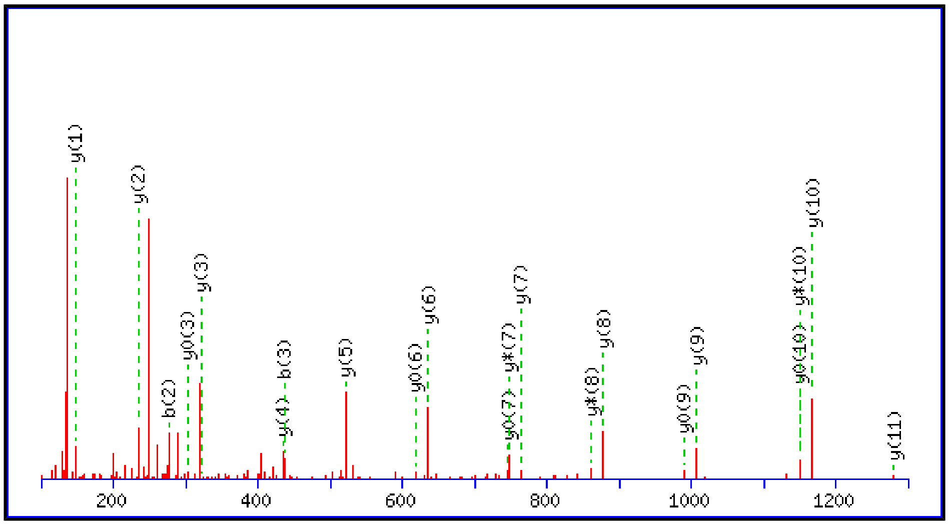

Monoisotopic mass of neutral peptide Mr(calc): 1442.6347
Variable modifications:
C3 : Carbamidomethyl (C)
Ions Score: 94 **Expect:** 8.4e-008
Matches : 21/116 fragment ions using 31 most intense peaks (help)

#	b	b^{++}	b*	b^{*++}	b^{0}	b^{0++}	Seq.	y	y^{++}	y*	y^{*++}	y^{0}	y^{0++}	#
1	164.0706	82.5389					Y							12
2	**277.1547**	139.0810					I	***1280.5787***	640.7930	1263.5522	632.2797	1262.5681	631.7877	11
3	**437.1853**	219.0963					C	***1167.4946***	584.2510	1150.4681	575.7377	1149.4841	575.2457	10
4	566.2279	283.6176			548.2173	274.6123	E	***1007.4640***	504.2356	990.4374	495.7224	989.4534	495.2304	9
5	680.2708	340.6391	663.2443	332.1258	662.2603	331.6338	N	***878.4214***	439.7143	861.3949	431.2011	860.4108	430.7091	8
6	808.3294	404.6683	791.3029	396.1551	790.3189	395.6631	Q	***764.3785***	382.6929	747.3519	374.1796	746.3679	373.6876	7
7	923.3564	462.1818	906.3298	453.6685	905.3458	453.1765	D	***636.3199***	318.6636	619.2933	310.1503	618.3093	309.6583	6
8	1010.3884	505.6978	993.3618	497.1846	992.3778	496.6925	S	***521.2930***	261.1501	504.2664	252.6368	503.2824	252.1448	5
9	1123.4725	562.2399	1106.4459	553.7266	1105.4619	553.2346	I	***434.2609***	217.6341	417.2344	209.1208	416.2504	208.6288	4
10	1210.5045	605.7559	1193.4779	597.2426	1192.4939	596.7506	S	***321.1769***	161.0921	304.1503	152.5788	303.1663	152.0868	3
11	1297.5365	649.2719	1280.5100	640.7586	1279.5259	640.2666	S	***234.1448***	117.5761	217.1183	109.0628	216.1343	108.5708	2
12							K	***147.1128***	74.0600	130.0863	65.5468			1

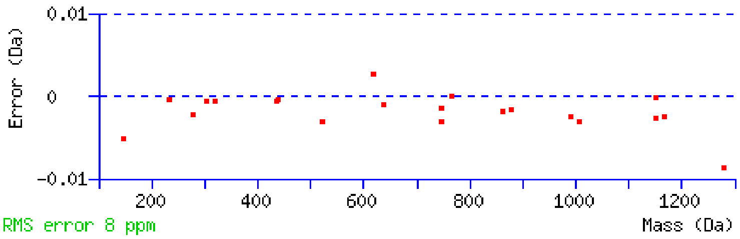

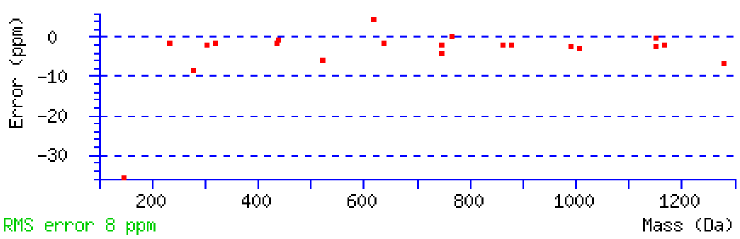

NCBI **BLAST** search of YICENQDSISSK
(Parameters: blastp, nr protein database, expect=20000, no filter, PAM30)
Other BLAST web gateways

All matches to this query

Score	Mr(calc):	Delta	Sequence
94.0	1442.6347	-0.0022	YICENQDSISSK
2.6	1441.6217	1.0108	EKPYKCEECEK
1.3	1442.6282	0.0043	YLCMATNAAGTDR

Mascot: http://www.matrixscience.com/

Mascot Search Results

Peptide View

MS/MS Fragmentation of **YICENQDSISSK**
Found in **sp|P02768|ALBU_HUMAN**, Serum albumin OS=Homo sapiens GN=ALB PE=1 SV=2

Match to Query 5057: 1442.634608 from(722.324580,2+) intensity(27988470.0000) scans(3952) rtinseconds(805) index(3107)
Title: 150808_TTSH_Patient_Plasma_72_Spectrum019361_scans__3952_RTINSECONDS=805
Data file L:\\Ard_TTSH\\T1D\\T150808_TTSH_Patient_Plasma_72.mgf

Click mouse within plot area to zoom in by factor of two about that point
Or, Plot from 100 to 1300 Da Full range
Label all possible matches ○ Label matches used for scoring ⊙

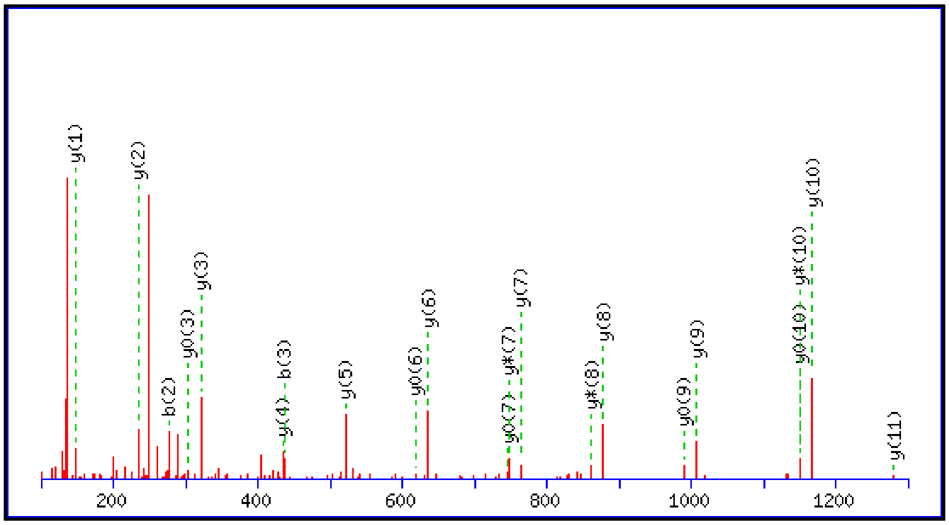

Monoisotopic mass of neutral peptide Mr(calc): 1442.6347
Variable modifications:
C3 : Carbamidomethyl (C)
Ions Score: 94 **Expect:** 8.8e-008
Matches : 21/116 fragment ions using 31 most intense peaks (help)

#	b	b^{++}	b*	b^{*++}	b^0	b^{0++}	Seq.	y	y^{++}	y*	y^{*++}	y^0	y^{0++}	#
1	164.0706	82.5389					Y							12
2	277.1547	139.0810					I	1280.5787	640.7930	1263.5522	632.2797	1262.5681	631.7877	11
3	437.1853	219.0963					C	1167.4946	584.2510	1150.4681	575.7377	1149.4841	575.2457	10
4	566.2279	283.6176			548.2173	274.6123	E	1007.4640	504.2356	990.4374	495.7224	989.4534	495.2304	9
5	680.2708	340.6391	663.2443	332.1258	662.2603	331.6338	N	878.4214	439.7143	861.3949	431.2011	860.4108	430.7091	8
6	808.3294	404.6683	791.3029	396.1551	790.3189	395.6631	Q	764.3785	382.6929	747.3519	374.1796	746.3679	373.6876	7
7	923.3564	462.1818	906.3298	453.6685	905.3458	453.1765	D	636.3199	318.6636	619.2933	310.1503	618.3093	309.6583	6
8	1010.3884	505.6978	993.3618	497.1846	992.3778	496.6925	S	521.2930	261.1501	504.2664	252.6368	503.2824	252.1448	5
9	1123.4725	562.2399	1106.4459	553.7266	1105.4619	553.2346	I	434.2609	217.6341	417.2344	209.1208	416.2504	208.6288	4
10	1210.5045	605.7559	1193.4779	597.2426	1192.4939	596.7506	S	321.1769	161.0921	304.1503	152.5788	303.1663	152.0868	3
11	1297.5365	649.2719	1280.5100	640.7586	1279.5259	640.2666	S	234.1448	117.5761	217.1183	109.0628	216.1343	108.5708	2
12							K	147.1128	74.0600	130.0863	65.5468			1

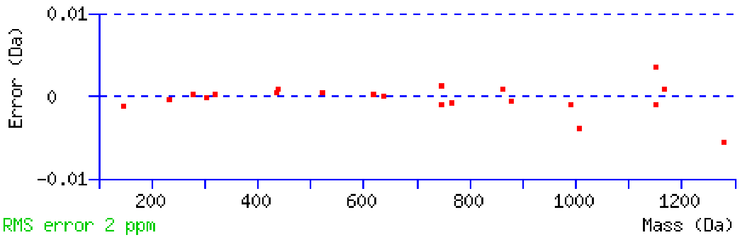

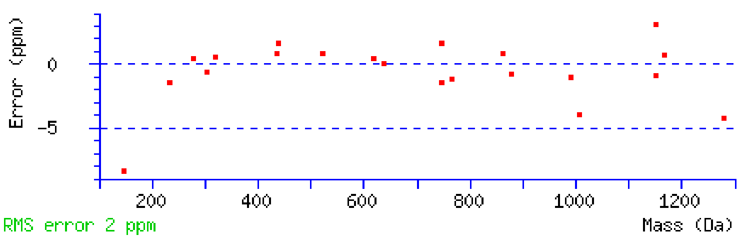

NCBI **BLAST** search of YICENQDSISSK
(Parameters: blastp, nr protein database, expect=20000, no filter, PAM30)
Other BLAST web gateways

All matches to this query

Score	Mr(calc):	Delta	Sequence
94.0	1442.6347	-0.0001	YICENQDSISSK
3.5	1441.6217	1.0129	EKPYKCEECEK
2.3	1442.6282	0.0064	YLCMATNAAGTDR

Mascot: http://www.matrixscience.com/

Mascot Search Results

Peptide View

MS/MS Fragmentation of **YICENQDSISSK**
Found in **sp|P02768|ALBU_HUMAN**, Serum albumin OS=Homo sapiens GN=ALB PE=1 SV=2

Match to Query 4516: 1442.633888 from(722.324220,2+) intensity(6423852.5000) scans(4271) rtinseconds(839) index(3454)
Title: 150808_TTSH_Patient_Plasma_73_Spectrum019919_scans__4271_RTINSECONDS=839
Data file L:\\Ard_TTSH\\T1D\\T150808_TTSH_Patient_Plasma_73.mgf

Click mouse within plot area to zoom in by factor of two about that point
Or, Plot from 100 to 1300 Da Full range
Label all possible matches ○ Label matches used for scoring ⦿

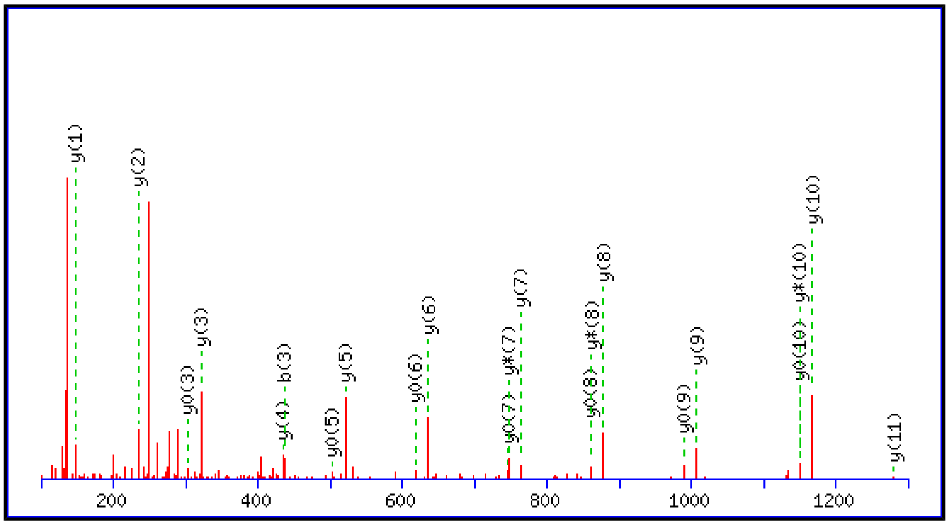

Monoisotopic mass of neutral peptide Mr(calc): 1442.6347
Variable modifications:
C3 : Carbamidomethyl (C)
Ions Score: 94 **Expect:** 8.9e-008
Matches : 22/116 fragment ions using 32 most intense peaks (help)

#	b	b^{++}	b*	b^{*++}	b^0	b^{0++}	Seq.	y	y^{++}	y*	y^{*++}	y^0	y^{0++}	#
1	164.0706	82.5389					Y							12
2	277.1547	139.0810					I	1280.5787	640.7930	1263.5522	632.2797	1262.5681	631.7877	11
3	437.1853	219.0963					C	1167.4946	584.2510	1150.4681	575.7377	1149.4841	575.2457	10
4	566.2279	283.6176			548.2173	274.6123	E	1007.4640	504.2356	990.4374	495.7224	989.4534	495.2304	9
5	680.2708	340.6391	663.2443	332.1258	662.2603	331.6338	N	878.4214	439.7143	861.3949	431.2011	860.4108	430.7091	8
6	808.3294	404.6683	791.3029	396.1551	790.3189	395.6631	Q	764.3785	382.6929	747.3519	374.1796	746.3679	373.6876	7
7	923.3564	462.1818	906.3298	453.6685	905.3458	453.1765	D	636.3199	318.6636	619.2933	310.1503	618.3093	309.6583	6
8	1010.3884	505.6978	993.3618	497.1846	992.3778	496.6925	S	521.2930	261.1501	504.2664	252.6368	503.2824	252.1448	5
9	1123.4725	562.2399	1106.4459	553.7266	1105.4619	553.2346	I	434.2609	217.6341	417.2344	209.1208	416.2504	208.6288	4
10	1210.5045	605.7559	1193.4779	597.2426	1192.4939	596.7506	S	321.1769	161.0921	304.1503	152.5788	303.1663	152.0868	3
11	1297.5365	649.2719	1280.5100	640.7586	1279.5259	640.2666	S	234.1448	117.5761	217.1183	109.0628	216.1343	108.5708	2
12							K	147.1128	74.0600	130.0863	65.5468			1

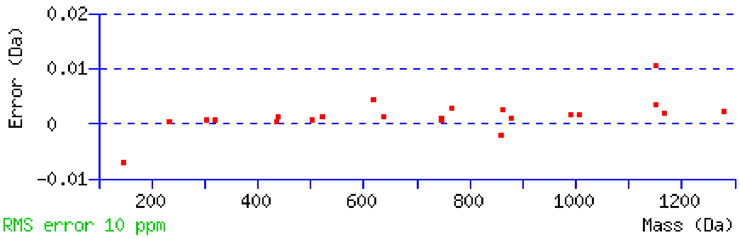

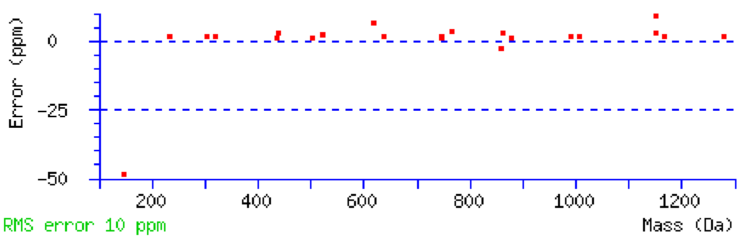

NCBI **BLAST** search of YICENQDSISSK
(Parameters: blastp, nr protein database, expect=20000, no filter, PAM30)
Other BLAST web gateways

All matches to this query

Score	Mr(calc):	Delta	Sequence
94.0	1442.6347	-0.0008	YICENQDSISSK
2.8	1441.6217	1.0122	EKPYKCEECEK
1.5	1442.6282	0.0057	YLCMATNAAGTDR

Mascot Search Results

Peptide View

MS/MS Fragmentation of **YICENQDSISSK**
Found in **sp|P02768|ALBU_HUMAN**, Serum albumin OS=Homo sapiens GN=ALB PE=1 SV=2

Match to Query 5140: 1442.631568 from(722.323060,2+) intensity(31132046.0000) scans(5062) rtinseconds(912) index(4412)
Title: 150818_TTSH_Patient_Plasma_05_Spectrum022669_scans__5062_RTINSECONDS=912
Data file L:\\Ard_TTSH\\T1D\\T150818_TTSH_Patient_Plasma_05.mgf

Click mouse within plot area to zoom in by factor of two about that point
Or, [Plot from] 100 to 1500 Da [Full range]
Label all possible matches ○ Label matches used for scoring ⊙

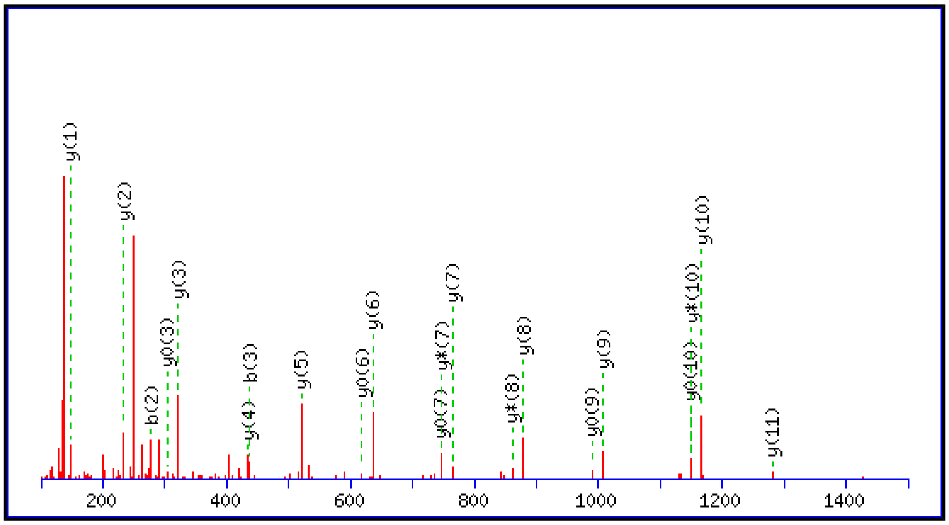


```
Monoisotopic mass of neutral peptide Mr(calc): 1442.6347
Variable modifications:
C3     : Carbamidomethyl (C)
Ions Score: 94  Expect: 8.5e-008
Matches : 21/116 fragment ions using 31 most intense peaks   (help)
```

#	b	b^{++}	b*	b^{*++}	b^{0}	b^{0++}	Seq.	y	y^{++}	y*	y^{*++}	y^{0}	y^{0++}	#
1	164.0706	82.5389					Y							12
2	**277.1547**	139.0810					I	***1280.5787***	640.7930	1263.5522	632.2797	1262.5681	631.7877	11
3	**437.1853**	219.0963					C	***1167.4946***	584.2510	1150.4681	575.7377	1149.4841	575.2457	10
4	566.2279	283.6176			548.2173	274.6123	E	***1007.4640***	504.2356	990.4374	495.7224	989.4534	495.2304	9
5	680.2708	340.6391	663.2443	332.1258	662.2603	331.6338	N	***878.4214***	439.7143	861.3949	431.2011	860.4108	430.7091	8
6	808.3294	404.6683	791.3029	396.1551	790.3189	395.6631	Q	***764.3785***	382.6929	747.3519	374.1796	746.3679	373.6876	7
7	923.3564	462.1818	906.3298	453.6685	905.3458	453.1765	D	***636.3199***	318.6636	619.2933	310.1503	618.3093	309.6583	6
8	1010.3884	505.6978	993.3618	497.1846	992.3778	496.6925	S	***521.2930***	261.1501	504.2664	252.6368	503.2824	252.1448	5
9	1123.4725	562.2399	1106.4459	553.7266	1105.4619	553.2346	I	***434.2609***	217.6341	417.2344	209.1208	416.2504	208.6288	4
10	1210.5045	605.7559	1193.4779	597.2426	1192.4939	596.7506	S	***321.1769***	161.0921	304.1503	152.5788	303.1663	152.0868	3
11	1297.5365	649.2719	1280.5100	640.7586	1279.5259	640.2666	S	***234.1448***	117.5761	217.1183	109.0628	216.1343	108.5708	2
12							K	***147.1128***	74.0600	130.0863	65.5468			1

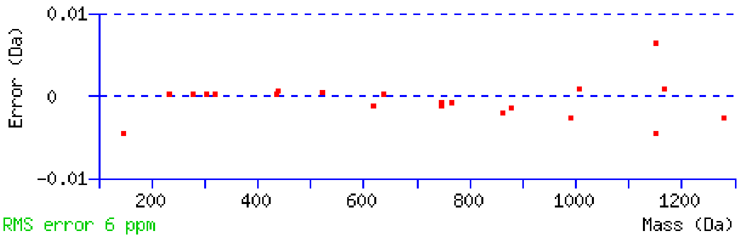

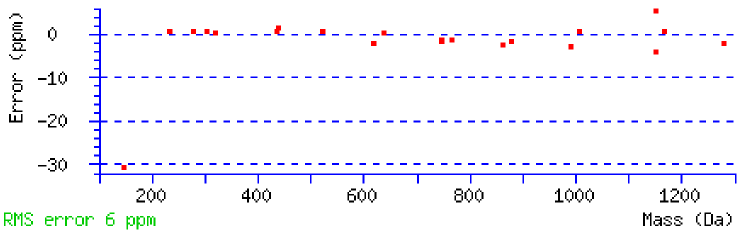

NCBI **BLAST** search of YICENQDSISSK
(Parameters: blastp, nr protein database, expect=20000, no filter, PAM30)
Other BLAST web gateways

All matches to this query

Score	Mr(calc):	Delta	Sequence
93.9	1442.6347	-0.0032	YICENQDSISSK

Mascot: http://www.matrixscience.com/

Mascot Search Results

Peptide View

MS/MS Fragmentation of **YICENQDSISSK**
Found in **sp|P02768|ALBU_HUMAN**, Serum albumin OS=Homo sapiens GN=ALB PE=1 SV=2

Match to Query 5073: 1442.632908 from(722.323730,2+) intensity(3074227.2500) scans(10611) rtinseconds(1912) index(9069)
Title: 150801_TTSH_Patient_Plasma_39_Spectrum026089_scans__10611_RTINSECONDS=1912
Data file L:\\Ard_TTSH\\T1D\\T150801_TTSH_Patient_Plasma_39.mgf

Click mouse within plot area to zoom in by factor of two about that point
Or, Plot from 100 to 1300 Da Full range
Label all possible matches ○ Label matches used for scoring ⦿

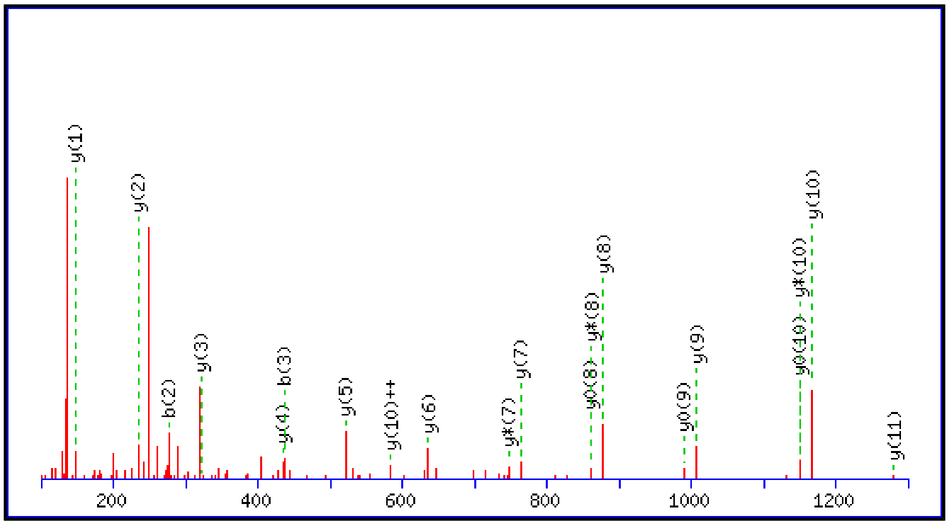

Monoisotopic mass of neutral peptide Mr(calc): 1442.6347
Variable modifications:
C3 : Carbamidomethyl (C)
Ions Score: 93 **Expect:** 9.8e-008
Matches : 20/116 fragment ions using 30 most intense peaks (help)

#	b	b^{++}	b*	b^{*++}	b^{0}	b^{0++}	Seq.	y	y^{++}	y*	y^{*++}	y^{0}	y^{0++}	#
1	164.0706	82.5389					Y							12
2	**277.1547**	139.0810					I	***1280.5787***	640.7930	1263.5522	632.2797	1262.5681	631.7877	11
3	**437.1853**	219.0963					C	***1167.4946***	584.2510	1150.4681	575.7377	1149.4841	575.2457	10
4	566.2279	283.6176			548.2173	274.6123	E	***1007.4640***	504.2356	990.4374	495.7224	989.4534	495.2304	9
5	680.2708	340.6391	663.2443	332.1258	662.2603	331.6338	N	***878.4214***	439.7143	861.3949	431.2011	860.4108	430.7091	8
6	808.3294	404.6683	791.3029	396.1551	790.3189	395.6631	Q	***764.3785***	382.6929	747.3519	374.1796	746.3679	373.6876	7
7	923.3564	462.1818	906.3298	453.6685	905.3458	453.1765	D	***636.3199***	318.6636	619.2933	310.1503	618.3093	309.6583	6
8	1010.3884	505.6978	993.3618	497.1846	992.3778	496.6925	S	***521.2930***	261.1501	504.2664	252.6368	503.2824	252.1448	5
9	1123.4725	562.2399	1106.4459	553.7266	1105.4619	553.2346	I	***434.2609***	217.6341	417.2344	209.1208	416.2504	208.6288	4
10	1210.5045	605.7559	1193.4779	597.2426	1192.4939	596.7506	S	***321.1769***	161.0921	304.1503	152.5788	303.1663	152.0868	3
11	1297.5365	649.2719	1280.5100	640.7586	1279.5259	640.2666	S	***234.1448***	117.5761	217.1183	109.0628	216.1343	108.5708	2
12							K	***147.1128***	74.0600	130.0863	65.5468			1

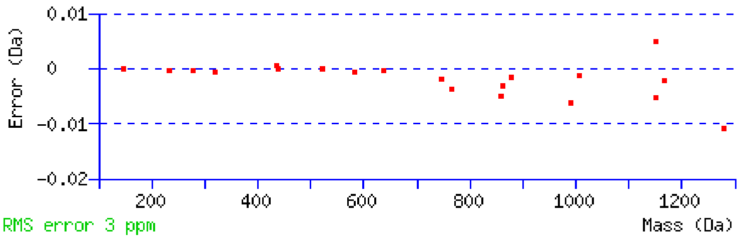

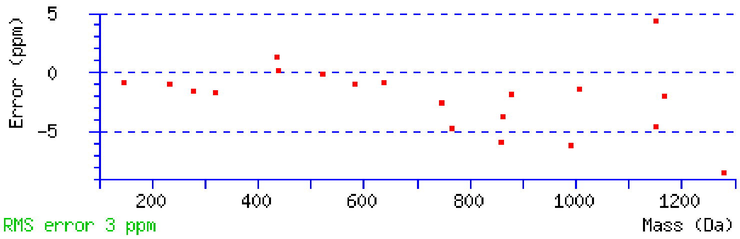

NCBI **BLAST** search of YICENQDSISSK
(Parameters: blastp, nr protein database, expect=20000, no filter, PAM30)
Other BLAST web gateways

All matches to this query

Score	Mr(calc):	Delta	Sequence
93.3	1442.6347	-0.0018	YICENQDSISSK
3.5	1441.6217	1.0112	EKPYKCEECEK
2.8	1442.6282	0.0047	YLCMATNAAGTDR

Mascot: http://www.matrixscience.com/

{MATRIX SCIENCE} Mascot Search Results

Peptide View

MS/MS Fragmentation of **YICENQDSISSK**
Found in **sp|P02768|ALBU_HUMAN**, Serum albumin OS=Homo sapiens GN=ALB PE=1 SV=2

Match to Query 5152: 1442.633648 from(722.324100,2+) intensity(1559449.0000) scans(16098) rtinseconds(2870) index(13986)
Title: 150801_TTSH_Patient_Plasma_40_Spectrum030921_scans__16098_RTINSECONDS=2870
Data file L:\\Ard_TTSH\\T1D\\T150801_TTSH_Patient_Plasma_40.mgf

Click mouse within plot area to zoom in by factor of two about that point
Or, [Plot from] [100] to [1300] Da [Full range]
Label all possible matches ○ Label matches used for scoring ⊙

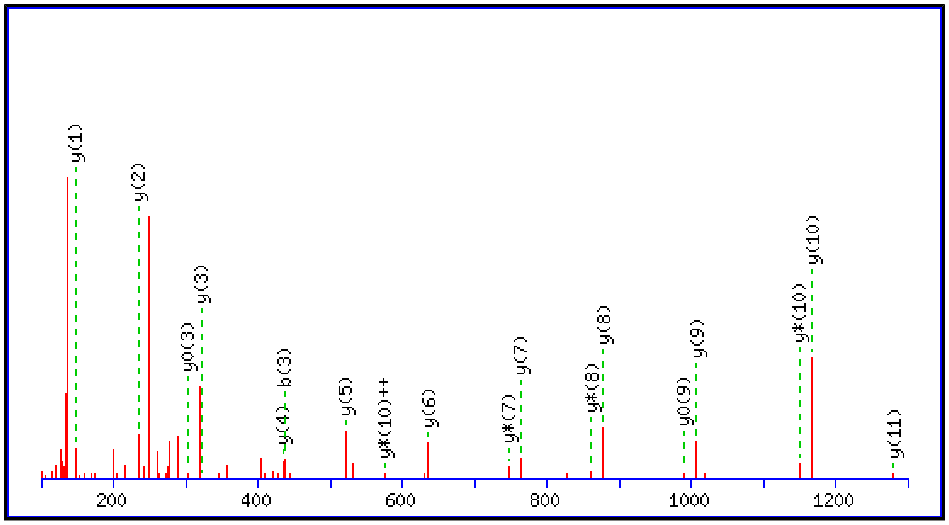

Monoisotopic mass of neutral peptide Mr(calc): 1442.6347
Variable modifications:
C3 : Carbamidomethyl (C)
Ions Score: 92 **Expect:** 1.2e-007
Matches : 18/116 fragment ions using 28 most intense peaks (help)

#	b	b^{++}	b*	b^{*++}	b^{0}	b^{0++}	Seq.	y	y^{++}	y*	y^{*++}	y^{0}	y^{0++}	#
1	164.0706	82.5389					Y							12
2	277.1547	139.0810					I	1280.5787	640.7930	1263.5522	632.2797	1262.5681	631.7877	11
3	437.1853	219.0963					C	1167.4946	584.2510	1150.4681	575.7377	1149.4841	575.2457	10
4	566.2279	283.6176			548.2173	274.6123	E	1007.4640	504.2356	990.4374	495.7224	989.4534	495.2304	9
5	680.2708	340.6391	663.2443	332.1258	662.2603	331.6338	N	878.4214	439.7143	861.3949	431.2011	860.4108	430.7091	8
6	808.3294	404.6683	791.3029	396.1551	790.3189	395.6631	Q	764.3785	382.6929	747.3519	374.1796	746.3679	373.6876	7
7	923.3564	462.1818	906.3298	453.6685	905.3458	453.1765	D	636.3199	318.6636	619.2933	310.1503	618.3093	309.6583	6
8	1010.3884	505.6978	993.3618	497.1846	992.3778	496.6925	S	521.2930	261.1501	504.2664	252.6368	503.2824	252.1448	5
9	1123.4725	562.2399	1106.4459	553.7266	1105.4619	553.2346	I	434.2609	217.6341	417.2344	209.1208	416.2504	208.6288	4
10	1210.5045	605.7559	1193.4779	597.2426	1192.4939	596.7506	S	321.1769	161.0921	304.1503	152.5788	303.1663	152.0868	3
11	1297.5365	649.2719	1280.5100	640.7586	1279.5259	640.2666	S	234.1448	117.5761	217.1183	109.0628	216.1343	108.5708	2
12							K	147.1128	74.0600	130.0863	65.5468			1

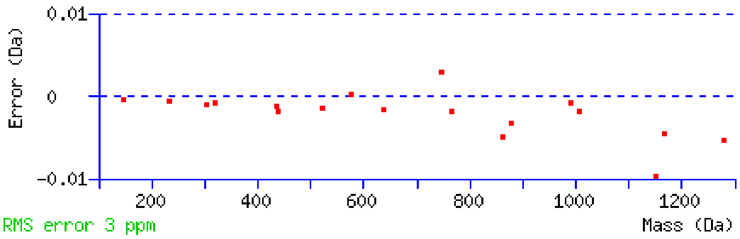

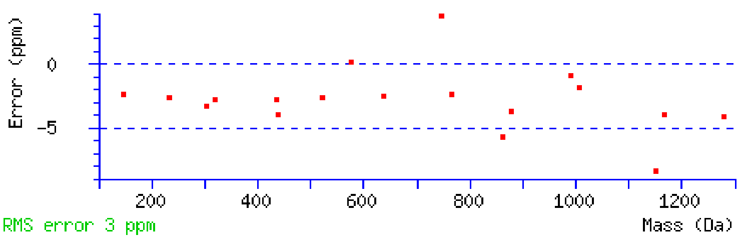

NCBI **BLAST** search of YICENQDSISSK
(Parameters: blastp, nr protein database, expect=20000, no filter, PAM30)
Other BLAST web gateways

All matches to this query

Score	Mr(calc):	Delta	Sequence
92.4	1442.6347	-0.0011	YICENQDSISSK
4.3	1441.6217	1.0119	EKPYKCEECEK
3.2	1442.6282	0.0054	YLCMATNAAGTDR

Mascot: http://www.matrixscience.com/

Mascot Search Results

Peptide View

MS/MS Fragmentation of **YICENQDSISSK**
Found in **sp|P02768|ALBU_HUMAN**, Serum albumin OS=Homo sapiens GN=ALB PE=1 SV=2

Match to Query 4912: 1442.633888 from(722.324220,2+) intensity(116247816.0000) scans(4996) rtinseconds(1020) index(3950)
Title: 150808_TTSH_Patient_Plasma_44_Spectrum020191_scans__4996_RTINSECONDS=1020
Data file L:\\Ard_TTSH\\T1D\\T150808_TTSH_Patient_Plasma_44.mgf

Click mouse within plot area to zoom in by factor of two about that point
Or, Plot from 100 to 1300 Da Full range
Label all possible matches ○ Label matches used for scoring ⦿

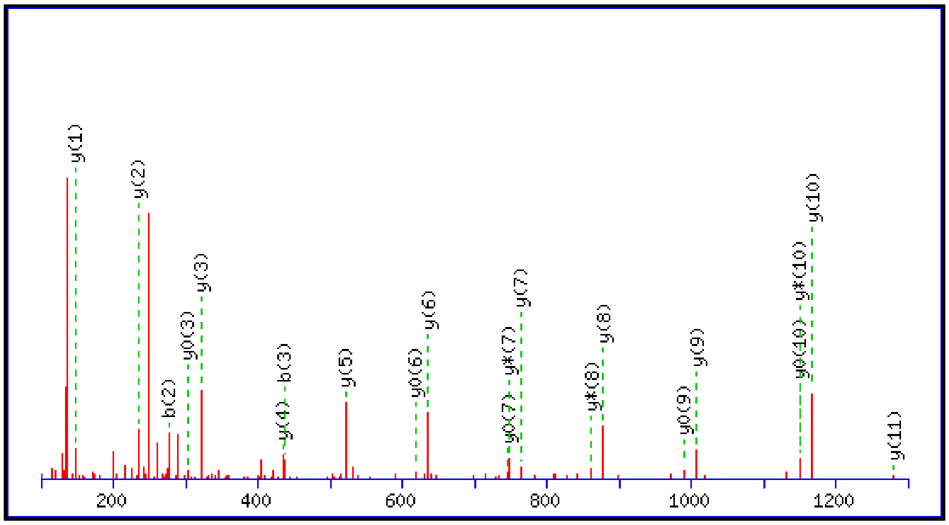

Monoisotopic mass of neutral peptide Mr(calc): 1442.6347
Variable modifications:
C3 : Carbamidomethyl (C)
Ions Score: 92 **Expect:** 1.4e-007
Matches : 21/116 fragment ions using 32 most intense peaks (help)

#	b	b^{++}	b*	b*$^{++}$	b^{0}	b^{0++}	Seq.	y	y^{++}	y*	y*$^{++}$	y^{0}	y^{0++}	#
1	164.0706	82.5389					Y							12
2	277.1547	139.0810					I	1280.5787	640.7930	1263.5522	632.2797	1262.5681	631.7877	11
3	437.1853	219.0963					C	1167.4946	584.2510	1150.4681	575.7377	1149.4841	575.2457	10
4	566.2279	283.6176			548.2173	274.6123	E	1007.4640	504.2356	990.4374	495.7224	989.4534	495.2304	9
5	680.2708	340.6391	663.2443	332.1258	662.2603	331.6338	N	878.4214	439.7143	861.3949	431.2011	860.4108	430.7091	8
6	808.3294	404.6683	791.3029	396.1551	790.3189	395.6631	Q	764.3785	382.6929	747.3519	374.1796	746.3679	373.6876	7
7	923.3564	462.1818	906.3298	453.6685	905.3458	453.1765	D	636.3199	318.6636	619.2933	310.1503	618.3093	309.6583	6
8	1010.3884	505.6978	993.3618	497.1846	992.3778	496.6925	S	521.2930	261.1501	504.2664	252.6368	503.2824	252.1448	5
9	1123.4725	562.2399	1106.4459	553.7266	1105.4619	553.2346	I	434.2609	217.6341	417.2344	209.1208	416.2504	208.6288	4
10	1210.5045	605.7559	1193.4779	597.2426	1192.4939	596.7506	S	321.1769	161.0921	304.1503	152.5788	303.1663	152.0868	3
11	1297.5365	649.2719	1280.5100	640.7586	1279.5259	640.2666	S	234.1448	117.5761	217.1183	109.0628	216.1343	108.5708	2
12							K	147.1128	74.0600	130.0863	65.5468			1

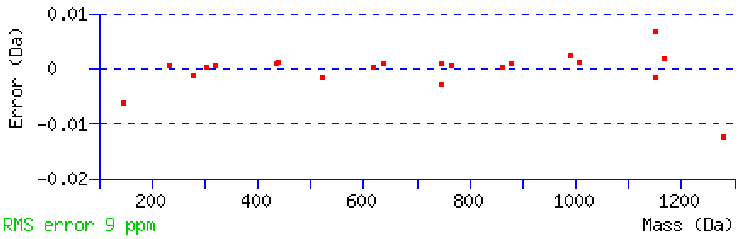

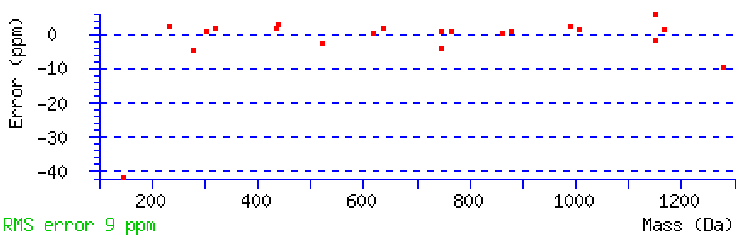

NCBI **BLAST** search of YICENQDSISSK
(Parameters: blastp, nr protein database, expect=20000, no filter, PAM30)
Other BLAST web gateways

All matches to this query

Score	Mr(calc):	Delta	Sequence
91.9	1442.6347	-0.0008	YICENQDSISSK
3.1	1441.6217	1.0122	EKPYKCEECEK
1.6	1442.6282	0.0057	YLCMATNAAGTDR

Mascot: http://www.matrixscience.com/

Mascot Search Results

Peptide View

MS/MS Fragmentation of **YICENQDSISSK**
Found in **sp|P02768|ALBU_HUMAN**, Serum albumin OS=Homo sapiens GN=ALB PE=1 SV=2

Match to Query 4028: 1442.634988 from(722.324770,2+) intensity(3553170.0000) scans(2493) rtinseconds(560) index(1774)
Title: 150825_TTSH_Patient_Plasma_67_Spectrum018981_scans__2493_RTINSECONDS=560
Data file L:\\Ard_TTSH\\T1D\\T150825_TTSH_Patient_Plasma_67.mgf

Click mouse within plot area to zoom in by factor of two about that point
Or, Plot from 100 to 1300 Da Full range
Label all possible matches ○ Label matches used for scoring ⊙

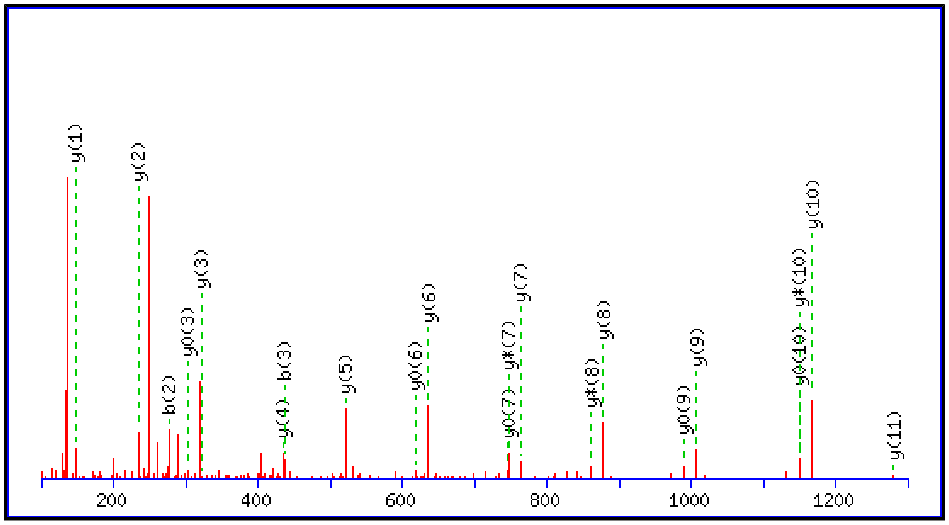

Monoisotopic mass of neutral peptide Mr(calc): 1442.6347
Variable modifications:
C3 : Carbamidomethyl (C)
Ions Score: 92 **Expect:** 1.5e-007
Matches : 21/116 fragment ions using 32 most intense peaks (help)

#	b	b^{++}	b*	b^{*++}	b^0	b^{0++}	Seq.	y	y^{++}	y*	y^{*++}	y^0	y^{0++}	#
1	164.0706	82.5389					Y							12
2	**277.1547**	139.0810					I	***1280.5787***	640.7930	1263.5522	632.2797	1262.5681	631.7877	11
3	**437.1853**	219.0963					C	***1167.4946***	584.2510	1150.4681	575.7377	1149.4841	575.2457	10
4	566.2279	283.6176			548.2173	274.6123	E	***1007.4640***	504.2356	990.4374	495.7224	989.4534	495.2304	9
5	680.2708	340.6391	663.2443	332.1258	662.2603	331.6338	N	***878.4214***	439.7143	861.3949	431.2011	860.4108	430.7091	8
6	808.3294	404.6683	791.3029	396.1551	790.3189	395.6631	Q	***764.3785***	382.6929	747.3519	374.1796	746.3679	373.6876	7
7	923.3564	462.1818	906.3298	453.6685	905.3458	453.1765	D	***636.3199***	318.6636	619.2933	310.1503	618.3093	309.6583	6
8	1010.3884	505.6978	993.3618	497.1846	992.3778	496.6925	S	***521.2930***	261.1501	504.2664	252.6368	503.2824	252.1448	5
9	1123.4725	562.2399	1106.4459	553.7266	1105.4619	553.2346	I	***434.2609***	217.6341	417.2344	209.1208	416.2504	208.6288	4
10	1210.5045	605.7559	1193.4779	597.2426	1192.4939	596.7506	S	***321.1769***	161.0921	304.1503	152.5788	303.1663	152.0868	3
11	1297.5365	649.2719	1280.5100	640.7586	1279.5259	640.2666	S	***234.1448***	117.5761	217.1183	109.0628	216.1343	108.5708	2
12							K	***147.1128***	74.0600	130.0863	65.5468			1

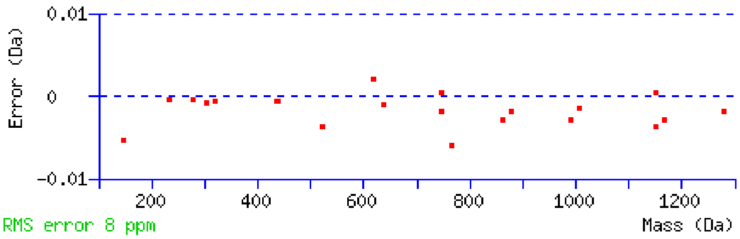

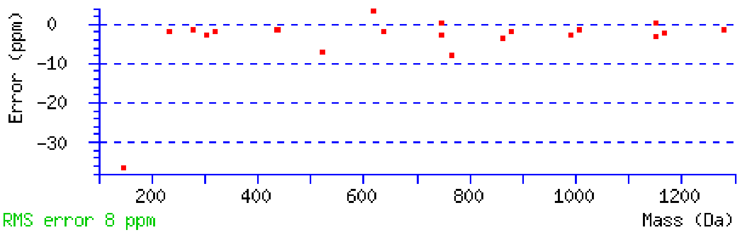

NCBI **BLAST** search of YICENQDSISSK
(Parameters: blastp, nr protein database, expect=20000, no filter, PAM30)
Other BLAST web gateways

All matches to this query

Score	Mr(calc):	Delta	Sequence
91.8	1442.6347	0.0003	YICENQDSISSK
2.8	1441.6217	1.0133	EKPYKCEECEK
1.3	1442.6282	0.0068	YLCMATNAAGTDR

Mascot: http://www.matrixscience.com/

Mascot Search Results

Peptide View

MS/MS Fragmentation of **YICENQDSISSK**
Found in **sp|P02768|ALBU_HUMAN**, Serum albumin OS=Homo sapiens GN=ALB PE=1 SV=2

Match to Query 6296: 1442.633528 from(722.324040,2+) intensity(71788048.0000) scans(5856) rtinseconds(1189) index(4642)
Title: 150801_TTSH_Patient_Plasma_83_Spectrum021140_scans__5856_RTINSECONDS=1189
Data file L:\\Ard_TTSH\\T1D\\T150801_TTSH_Patient_Plasma_83.mgf

Click mouse within plot area to zoom in by factor of two about that point
Or, [Plot from] 100 to 1300 Da [Full range]
Label all possible matches ○ Label matches used for scoring ⊙

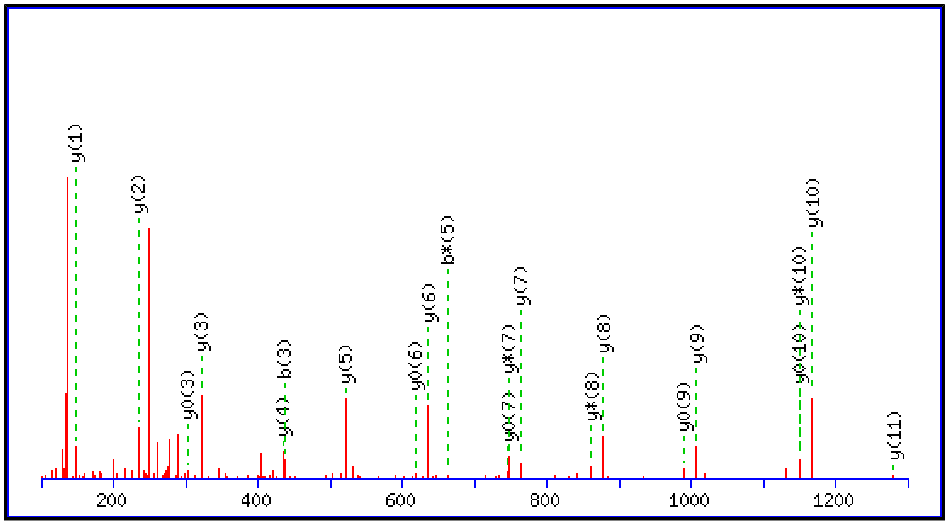

Monoisotopic mass of neutral peptide Mr(calc): 1442.6347
Variable modifications:
C3 : Carbamidomethyl (C)
Ions Score: 92 **Expect:** 1.5e-007
Matches : 21/116 fragment ions using 32 most intense peaks (help)

#	b	b^{++}	b*	b^{*++}	b^0	b^{0++}	Seq.	y	y^{++}	y*	y^{*++}	y^0	y^{0++}	#
1	164.0706	82.5389					Y							12
2	277.1547	139.0810					I	***1280.5787***	640.7930	1263.5522	632.2797	1262.5681	631.7877	11
3	**437.1853**	219.0963					C	***1167.4946***	584.2510	1150.4681	575.7377	1149.4841	575.2457	10
4	566.2279	283.6176			548.2173	274.6123	E	***1007.4640***	504.2356	990.4374	495.7224	989.4534	495.2304	9
5	680.2708	340.6391	663.2443	332.1258	662.2603	331.6338	N	***878.4214***	439.7143	861.3949	431.2011	860.4108	430.7091	8
6	808.3294	404.6683	791.3029	396.1551	790.3189	395.6631	Q	***764.3785***	382.6929	747.3519	374.1796	746.3679	373.6876	7
7	923.3564	462.1818	906.3298	453.6685	905.3458	453.1765	D	***636.3199***	318.6636	619.2933	310.1503	618.3093	309.6583	6
8	1010.3884	505.6978	993.3618	497.1846	992.3778	496.6925	S	***521.2930***	261.1501	504.2664	252.6368	503.2824	252.1448	5
9	1123.4725	562.2399	1106.4459	553.7266	1105.4619	553.2346	I	***434.2609***	217.6341	417.2344	209.1208	416.2504	208.6288	4
10	1210.5045	605.7559	1193.4779	597.2426	1192.4939	596.7506	S	***321.1769***	161.0921	304.1503	152.5788	303.1663	152.0868	3
11	1297.5365	649.2719	1280.5100	640.7586	1279.5259	640.2666	S	***234.1448***	117.5761	217.1183	109.0628	216.1343	108.5708	2
12							K	***147.1128***	74.0600	130.0863	65.5468			1

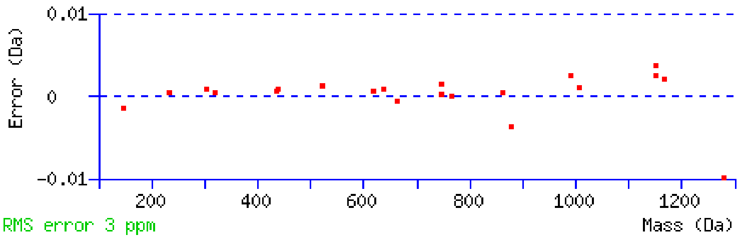

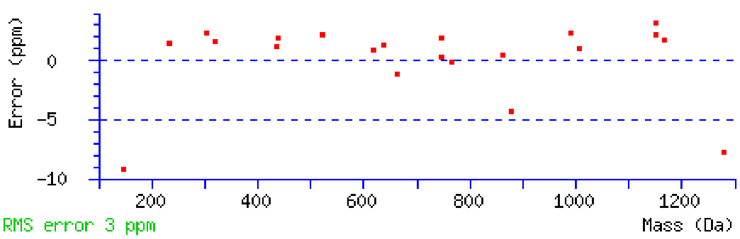

NCBI **BLAST** search of YICENQDSISSK
(Parameters: blastp, nr protein database, expect=20000, no filter, PAM30)
Other BLAST web gateways

All matches to this query

Score	Mr(calc):	Delta	Sequence
91.7	1442.6347	-0.0012	YICENQDSISSK
2.9	1441.6217	1.0118	EKPYKCEECEK
1.7	1442.6282	0.0053	YLCMATNAAGTDR

Mascot: http://www.matrixscience.com/

Mascot Search Results

Peptide View

MS/MS Fragmentation of **YICENQDSISSK**
Found in **sp|P02768|ALBU_HUMAN**, Serum albumin OS=Homo sapiens GN=ALB PE=1 SV=2

Match to Query 5380: 1442.637548 from(722.326050,2+) intensity(3423875.0000) scans(2924) rtinseconds(547) index(2463)
Title: 150801_TTSH_Patient_Plasma_44_Spectrum019340_scans__2924_RTINSECONDS=547
Data file L:\\Ard_TTSH\\T1D\\T150801_TTSH_Patient_Plasma_44.mgf

Click mouse within plot area to zoom in by factor of two about that point
Or, [Plot from] 100 to 1300 Da [Full range]
Label all possible matches ○ Label matches used for scoring ⊙

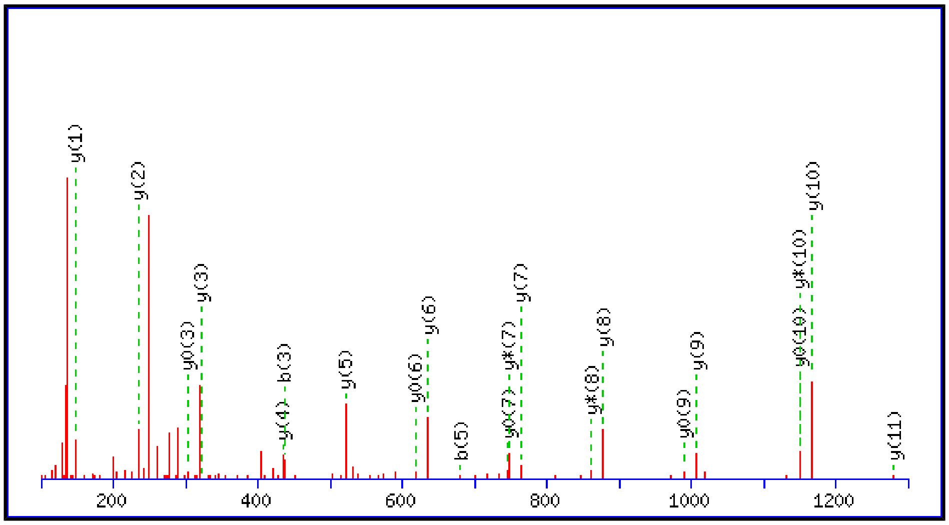

Monoisotopic mass of neutral peptide Mr(calc): 1442.6347
Variable modifications:
C3 : Carbamidomethyl (C)
Ions Score: 92 **Expect:** 1.7e-007
Matches : 21/116 fragment ions using 32 most intense peaks (help)

#	b	b^{++}	b*	b^{*++}	b^0	b^{0++}	Seq.	y	y^{++}	y*	y^{*++}	y^0	y^{0++}	#
1	164.0706	82.5389					Y							12
2	277.1547	139.0810					I	1280.5787	640.7930	1263.5522	632.2797	1262.5681	631.7877	11
3	437.1853	219.0963					C	1167.4946	584.2510	1150.4681	575.7377	1149.4841	575.2457	10
4	566.2279	283.6176			548.2173	274.6123	E	1007.4640	504.2356	990.4374	495.7224	989.4534	495.2304	9
5	680.2708	340.6391	663.2443	332.1258	662.2603	331.6338	N	878.4214	439.7143	861.3949	431.2011	860.4108	430.7091	8
6	808.3294	404.6683	791.3029	396.1551	790.3189	395.6631	Q	764.3785	382.6929	747.3519	374.1796	746.3679	373.6876	7
7	923.3564	462.1818	906.3298	453.6685	905.3458	453.1765	D	636.3199	318.6636	619.2933	310.1503	618.3093	309.6583	6
8	1010.3884	505.6978	993.3618	497.1846	992.3778	496.6925	S	521.2930	261.1501	504.2664	252.6368	503.2824	252.1448	5
9	1123.4725	562.2399	1106.4459	553.7266	1105.4619	553.2346	I	434.2609	217.6341	417.2344	209.1208	416.2504	208.6288	4
10	1210.5045	605.7559	1193.4779	597.2426	1192.4939	596.7506	S	321.1769	161.0921	304.1503	152.5788	303.1663	152.0868	3
11	1297.5365	649.2719	1280.5100	640.7586	1279.5259	640.2666	S	234.1448	117.5761	217.1183	109.0628	216.1343	108.5708	2
12							K	147.1128	74.0600	130.0863	65.5468			1

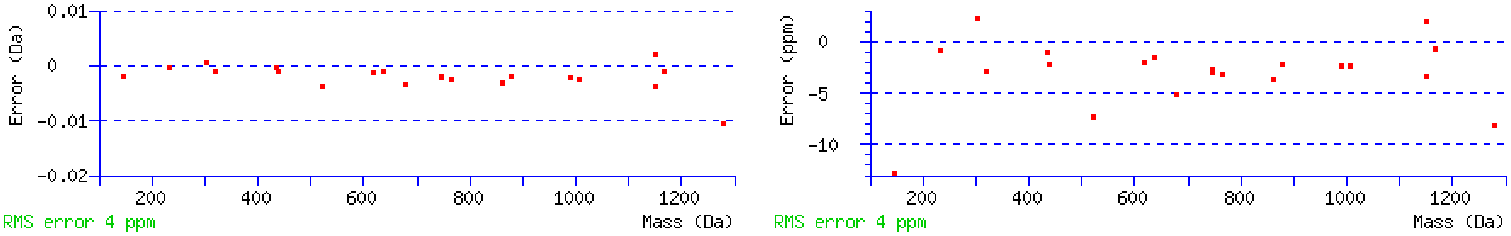

NCBI **BLAST** search of YICENQDSISSK
(Parameters: blastp, nr protein database, expect=20000, no filter, PAM30)
Other BLAST web gateways

All matches to this query

Score	Mr(calc):	Delta	Sequence
91.7	1442.6347	0.0028	YICENQDSISSK
3.1	1441.6217	1.0158	EKPYKCEECEK
1.8	1442.6282	0.0093	YLCMATNAAGTDR

Mascot: http://www.matrixscience.com/

Mascot Search Results

Peptide View

MS/MS Fragmentation of **YICENQDSISSK**
Found in **sp|P02768|ALBU_HUMAN**, Serum albumin OS=Homo sapiens GN=ALB PE=1 SV=2

Match to Query 4732: 1442.633648 from(722.324100,2+) intensity(7533499.0000) scans(4509) rtinseconds(813) index(3896)
Title: 150801_TTSH_Patient_Plasma_34_Spectrum021928_scans__4509_RTINSECONDS=813
Data file L:\\Ard_TTSH\\T1D\\T150801_TTSH_Patient_Plasma_34.mgf

Click mouse within plot area to zoom in by factor of two about that point
Or, Plot from 100 to 1300 Da Full range
Label all possible matches ○ Label matches used for scoring ⊙

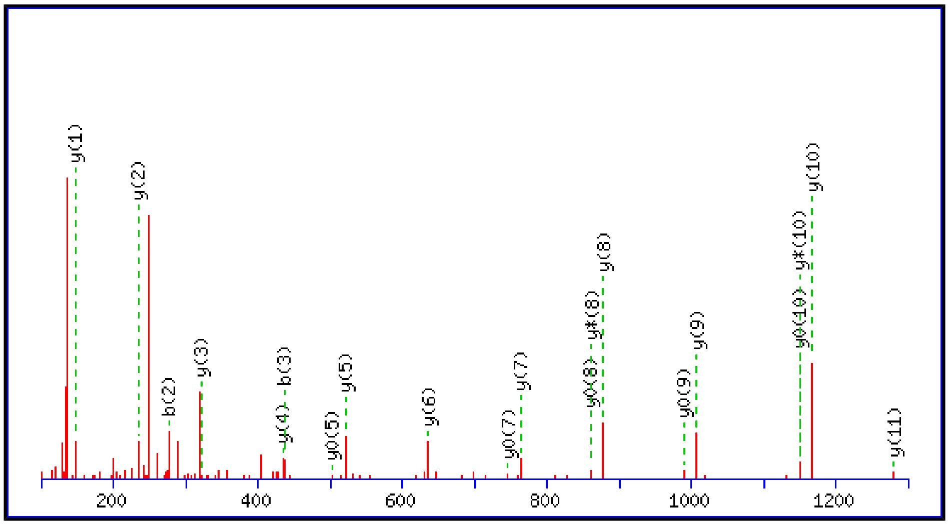

Monoisotopic mass of neutral peptide Mr(calc): 1442.6347
Variable modifications:
C3 : Carbamidomethyl (C)
Ions Score: 91 **Expect:** 1.6e-007
Matches : 20/116 fragment ions using 31 most intense peaks (help)

#	b	b++	b*	b*++	b0	b0++	Seq.	y	y++	y*	y*++	y0	y0++	#
1	164.0706	82.5389					Y							12
2	**277.1547**	139.0810					I	***1280.5787***	640.7930	1263.5522	632.2797	1262.5681	631.7877	11
3	**437.1853**	219.0963					C	***1167.4946***	584.2510	1150.4681	575.7377	1149.4841	575.2457	10
4	566.2279	283.6176			548.2173	274.6123	E	***1007.4640***	504.2356	990.4374	495.7224	989.4534	495.2304	9
5	680.2708	340.6391	663.2443	332.1258	662.2603	331.6338	N	***878.4214***	439.7143	861.3949	431.2011	860.4108	430.7091	8
6	808.3294	404.6683	791.3029	396.1551	790.3189	395.6631	Q	***764.3785***	382.6929	747.3519	374.1796	746.3679	373.6876	7
7	923.3564	462.1818	906.3298	453.6685	905.3458	453.1765	D	***636.3199***	318.6636	619.2933	310.1503	618.3093	309.6583	6
8	1010.3884	505.6978	993.3618	497.1846	992.3778	496.6925	S	***521.2930***	261.1501	504.2664	252.6368	503.2824	252.1448	5
9	1123.4725	562.2399	1106.4459	553.7266	1105.4619	553.2346	I	***434.2609***	217.6341	417.2344	209.1208	416.2504	208.6288	4
10	1210.5045	605.7559	1193.4779	597.2426	1192.4939	596.7506	S	***321.1769***	161.0921	304.1503	152.5788	303.1663	152.0868	3
11	1297.5365	649.2719	1280.5100	640.7586	1279.5259	640.2666	S	***234.1448***	117.5761	217.1183	109.0628	216.1343	108.5708	2
12							K	***147.1128***	74.0600	130.0863	65.5468			1

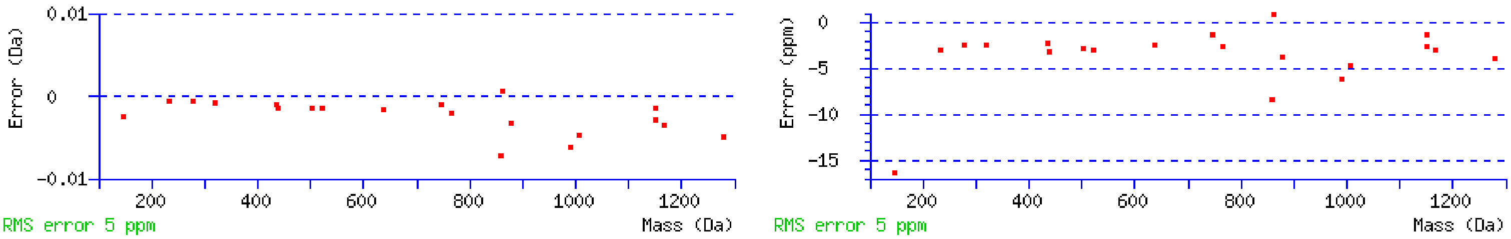

NCBI **BLAST** search of YICENQDSISSK
(Parameters: blastp, nr protein database, expect=20000, no filter, PAM30)
Other BLAST web gateways

All matches to this query

Score	Mr(calc):	Delta	Sequence
91.4	1442.6347	-0.0011	YICENQDSISSK
4.3	1441.6217	1.0119	EKPYKCEECEK
3.2	1442.6282	0.0054	YLCMATNAAGTDR

Mascot: http://www.matrixscience.com/

Mascot Search Results

Peptide View

MS/MS Fragmentation of **YICENQDSISSK**
Found in **sp|P02768|ALBU_HUMAN**, Serum albumin OS=Homo sapiens GN=ALB PE=1 SV=2

Match to Query 4170: 1442.634868 from(722.324710,2+) intensity(19054836.0000) scans(2640) rtinseconds(512) index(2138)
Title: 150801_TTSH_Patient_Plasma_47_Spectrum019484_scans__2640_RTINSECONDS=512
Data file L:\\Ard_TTSH\\T1D\\T150801_TTSH_Patient_Plasma_47.mgf

Click mouse within plot area to zoom in by factor of two about that point
Or, Plot from 100 to 1300 Da Full range
Label all possible matches ○ Label matches used for scoring ⦿

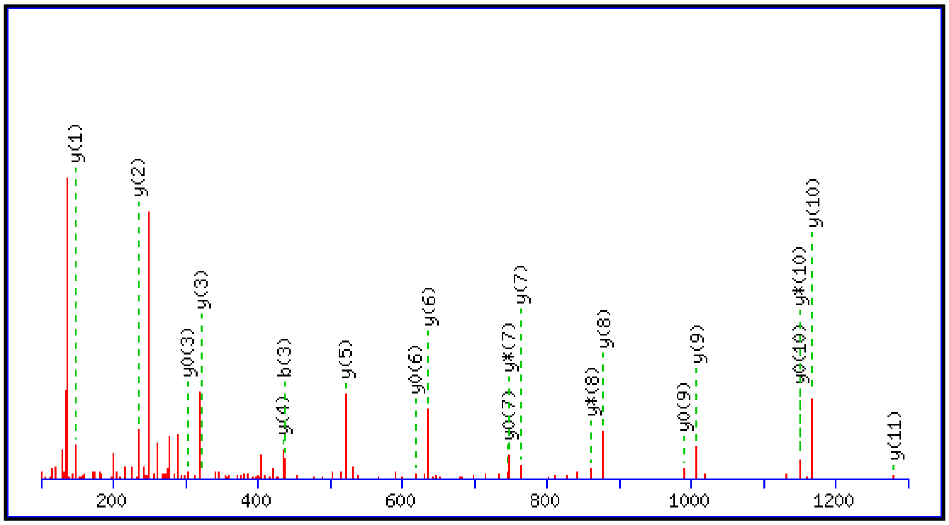

Monoisotopic mass of neutral peptide Mr(calc): 1442.6347
Variable modifications:
C3 : Carbamidomethyl (C)
Ions Score: 91 **Expect:** 1.7e-007
Matches : 20/116 fragment ions using 31 most intense peaks (help)

#	b	b^{++}	b*	b^{*++}	b^{0}	b^{0++}	Seq.	y	y^{++}	y*	y^{*++}	y^{0}	y^{0++}	#
1	164.0706	82.5389					Y							12
2	277.1547	139.0810					I	***1280.5787***	640.7930	1263.5522	632.2797	1262.5681	631.7877	11
3	**437.1853**	219.0963					C	***1167.4946***	584.2510	1150.4681	575.7377	1149.4841	575.2457	10
4	566.2279	283.6176			548.2173	274.6123	E	***1007.4640***	504.2356	990.4374	495.7224	989.4534	495.2304	9
5	680.2708	340.6391	663.2443	332.1258	662.2603	331.6338	N	***878.4214***	439.7143	861.3949	431.2011	860.4108	430.7091	8
6	808.3294	404.6683	791.3029	396.1551	790.3189	395.6631	Q	***764.3785***	382.6929	747.3519	374.1796	746.3679	373.6876	7
7	923.3564	462.1818	906.3298	453.6685	905.3458	453.1765	D	***636.3199***	318.6636	619.2933	310.1503	618.3093	309.6583	6
8	1010.3884	505.6978	993.3618	497.1846	992.3778	496.6925	S	***521.2930***	261.1501	504.2664	252.6368	503.2824	252.1448	5
9	1123.4725	562.2399	1106.4459	553.7266	1105.4619	553.2346	I	***434.2609***	217.6341	417.2344	209.1208	416.2504	208.6288	4
10	1210.5045	605.7559	1193.4779	597.2426	1192.4939	596.7506	S	***321.1769***	161.0921	304.1503	152.5788	303.1663	152.0868	3
11	1297.5365	649.2719	1280.5100	640.7586	1279.5259	640.2666	S	***234.1448***	117.5761	217.1183	109.0628	216.1343	108.5708	2
12							K	***147.1128***	74.0600	130.0863	65.5468			1

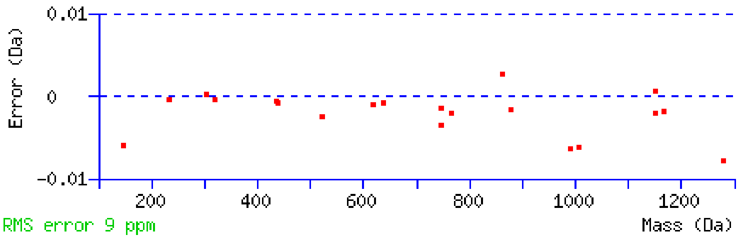

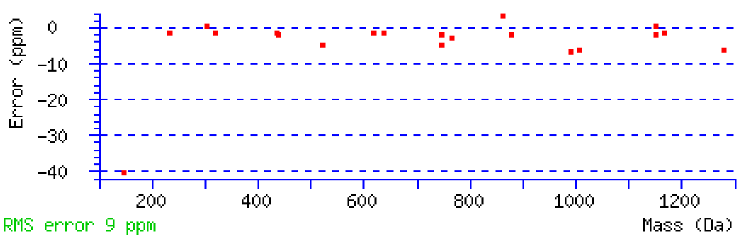

NCBI **BLAST** search of YICENQDSISSK
(Parameters: blastp, nr protein database, expect=20000, no filter, PAM30)
Other BLAST web gateways

All matches to this query

Score	Mr(calc):	Delta	Sequence
91.2	1442.6347	0.0001	YICENQDSISSK
2.8	1441.6217	1.0132	EKPYKCEECEK
1.5	1442.6282	0.0066	YLCMATNAAGTDR

Mascot: http://www.matrixscience.com/

Mascot Search Results

Peptide View

MS/MS Fragmentation of **YICENQDSISSK**
Found in **sp|P02768|ALBU_HUMAN**, Serum albumin OS=Homo sapiens GN=ALB PE=1 SV=2

Match to Query 4550: 1442.635848 from(722.325200,2+) intensity(3174353.5000) scans(4981) rtinseconds(962) index(4096)
Title: 150808_TTSH_Patient_Plasma_73_Spectrum020561_scans__4981_RTINSECONDS=962
Data file L:\\Ard_TTSH\\T1D\\T150808_TTSH_Patient_Plasma_73.mgf

Click mouse within plot area to zoom in by factor of two about that point
Or, Plot from 100 to 1300 Da Full range
Label all possible matches ○ Label matches used for scoring ⊙

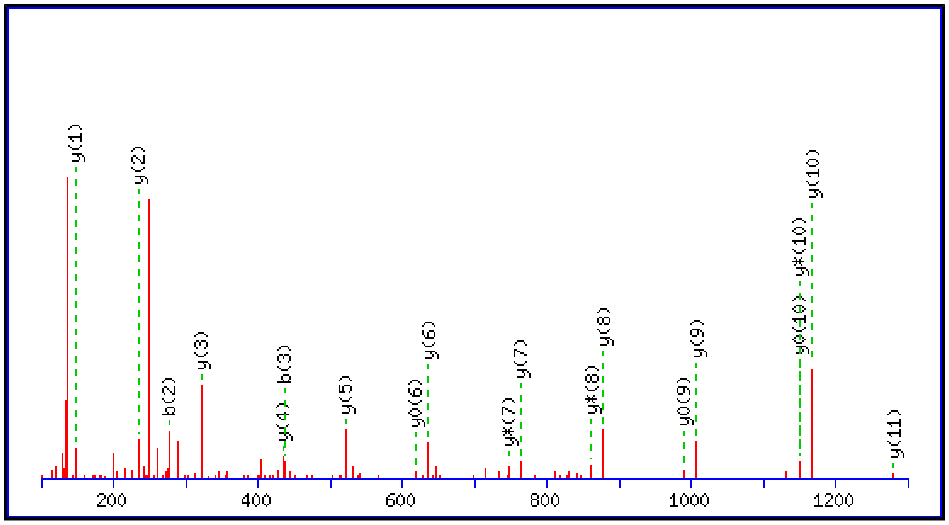

Monoisotopic mass of neutral peptide Mr(calc): 1442.6347
Variable modifications:
C3 : Carbamidomethyl (C)
Ions Score: 91 **Expect:** 1.9e-007
Matches : 19/116 fragment ions using 30 most intense peaks (help)

#	b	b^{++}	b*	b^{*++}	b^{0}	b^{0++}	Seq.	y	y^{++}	y*	y^{*++}	y^{0}	y^{0++}	#
1	164.0706	82.5389					Y							12
2	**277.1547**	139.0810					I	***1280.5787***	640.7930	1263.5522	632.2797	1262.5681	631.7877	11
3	**437.1853**	219.0963					C	***1167.4946***	584.2510	1150.4681	575.7377	1149.4841	575.2457	10
4	566.2279	283.6176			548.2173	274.6123	E	***1007.4640***	504.2356	990.4374	495.7224	989.4534	495.2304	9
5	680.2708	340.6391	663.2443	332.1258	662.2603	331.6338	N	***878.4214***	439.7143	861.3949	431.2011	860.4108	430.7091	8
6	808.3294	404.6683	791.3029	396.1551	790.3189	395.6631	Q	***764.3785***	382.6929	747.3519	374.1796	746.3679	373.6876	7
7	923.3564	462.1818	906.3298	453.6685	905.3458	453.1765	D	***636.3199***	318.6636	619.2933	310.1503	618.3093	309.6583	6
8	1010.3884	505.6978	993.3618	497.1846	992.3778	496.6925	S	***521.2930***	261.1501	504.2664	252.6368	503.2824	252.1448	5
9	1123.4725	562.2399	1106.4459	553.7266	1105.4619	553.2346	I	***434.2609***	217.6341	417.2344	209.1208	416.2504	208.6288	4
10	1210.5045	605.7559	1193.4779	597.2426	1192.4939	596.7506	S	***321.1769***	161.0921	304.1503	152.5788	303.1663	152.0868	3
11	1297.5365	649.2719	1280.5100	640.7586	1279.5259	640.2666	S	***234.1448***	117.5761	217.1183	109.0628	216.1343	108.5708	2
12							K	***147.1128***	74.0600	130.0863	65.5468			1

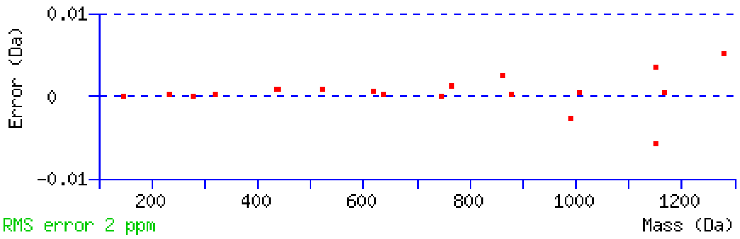

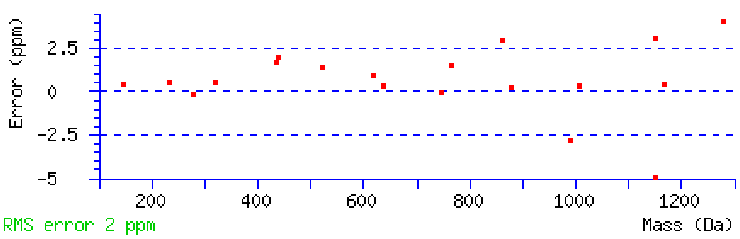

NCBI **BLAST** search of YICENQDSISSK
(Parameters: blastp, nr protein database, expect=20000, no filter, PAM30)
Other BLAST web gateways

All matches to this query

Score	Mr(calc):	Delta	Sequence
90.8	1442.6347	0.0011	YICENQDSISSK
3.8	1441.6217	1.0141	EKPYKCEECEK
2.7	1442.6282	0.0076	YLCMATNAAGTDR

Mascot: http://www.matrixscience.com/

{MATRIX SCIENCE} Mascot Search Results

Peptide View

MS/MS Fragmentation of **YICENQDSISSK**
Found in **sp|P02768|ALBU_HUMAN**, Serum albumin OS=Homo sapiens GN=ALB PE=1 SV=2

Match to Query 5158: 1442.633648 from(722.324100,2+) intensity(172401440.0000) scans(3436) rtinseconds(657) index(2765)
Title: 150801_TTSH_Patient_Plasma_40_Spectrum019700_scans__3436_RTINSECONDS=657
Data file L:\\Ard_TTSH\\T1D\\T150801_TTSH_Patient_Plasma_40.mgf

Click mouse within plot area to zoom in by factor of two about that point
Or, [Plot from] 100 to 1300 Da [Full range]
Label all possible matches ○ Label matches used for scoring ⊙

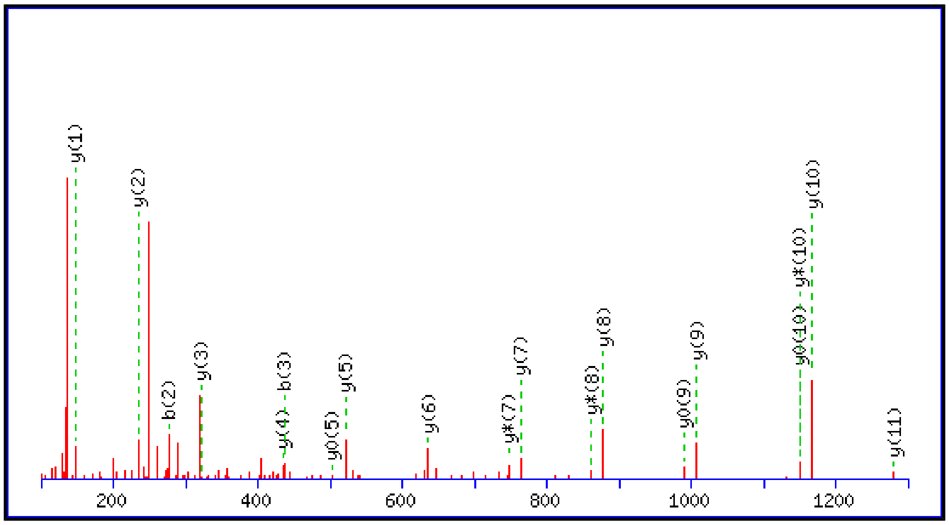

Monoisotopic mass of neutral peptide Mr(calc): 1442.6347
Variable modifications:
C3 : Carbamidomethyl (C)
Ions Score: 91 **Expect:** 1.8e-007
Matches : 19/116 fragment ions using 30 most intense peaks (help)

#	b	b^{++}	b^{*}	b^{*++}	b^{0}	b^{0++}	Seq.	y	y^{++}	y^{*}	y^{*++}	y^{0}	y^{0++}	#
1	164.0706	82.5389					Y							12
2	**277.1547**	139.0810					I	***1280.5787***	640.7930	1263.5522	632.2797	1262.5681	631.7877	11
3	**437.1853**	219.0963					C	***1167.4946***	584.2510	1150.4681	575.7377	1149.4841	575.2457	10
4	566.2279	283.6176			548.2173	274.6123	E	***1007.4640***	504.2356	990.4374	495.7224	989.4534	495.2304	9
5	680.2708	340.6391	663.2443	332.1258	662.2603	331.6338	N	***878.4214***	439.7143	861.3949	431.2011	860.4108	430.7091	8
6	808.3294	404.6683	791.3029	396.1551	790.3189	395.6631	Q	***764.3785***	382.6929	747.3519	374.1796	746.3679	373.6876	7
7	923.3564	462.1818	906.3298	453.6685	905.3458	453.1765	D	***636.3199***	318.6636	619.2933	310.1503	618.3093	309.6583	6
8	1010.3884	505.6978	993.3618	497.1846	992.3778	496.6925	S	***521.2930***	261.1501	504.2664	252.6368	503.2824	252.1448	5
9	1123.4725	562.2399	1106.4459	553.7266	1105.4619	553.2346	I	***434.2609***	217.6341	417.2344	209.1208	416.2504	208.6288	4
10	1210.5045	605.7559	1193.4779	597.2426	1192.4939	596.7506	S	***321.1769***	161.0921	304.1503	152.5788	303.1663	152.0868	3
11	1297.5365	649.2719	1280.5100	640.7586	1279.5259	640.2666	S	***234.1448***	117.5761	217.1183	109.0628	216.1343	108.5708	2
12							K	***147.1128***	74.0600	130.0863	65.5468			1

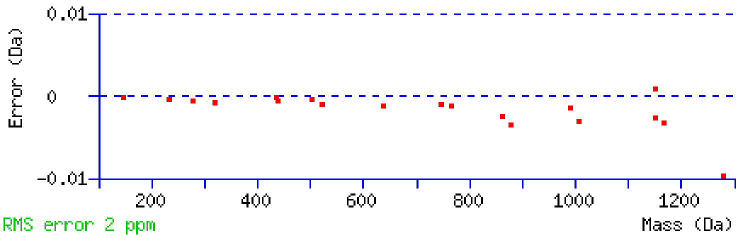

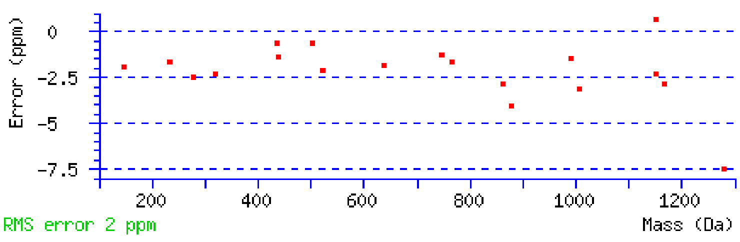

NCBI **BLAST** search of YICENQDSISSK
(Parameters: blastp, nr protein database, expect=20000, no filter, PAM30)
Other BLAST web gateways

All matches to this query

Score	Mr(calc):	Delta	Sequence
90.7	1442.6347	-0.0011	YICENQDSISSK
3.9	1441.6217	1.0119	EKPYKCEECEK
2.8	1442.6282	0.0054	YLCMATNAAGTDR

Mascot: http://www.matrixscience.com/

{MATRIX SCIENCE} Mascot Search Results

Peptide View

MS/MS Fragmentation of **YICENQDSISSK**
Found in **sp|P02768|ALBU_HUMAN**, Serum albumin OS=Homo sapiens GN=ALB PE=1 SV=2

Match to Query 5514: 1442.632308 from(722.323430,2+) intensity(581207.3750) scans(6215) rtinseconds(1347) index(4597)
Title: 150801_TTSH_Patient_Plasma_89_Spectrum020282_scans__6215_RTINSECONDS=1347
Data file L:\\Ard_TTSH\\T1D\\T150801_TTSH_Patient_Plasma_89.mgf

Click mouse within plot area to zoom in by factor of two about that point
Or, [Plot from] 100 to 1500 Da [Full range]
Label all possible matches ○ Label matches used for scoring ⊙

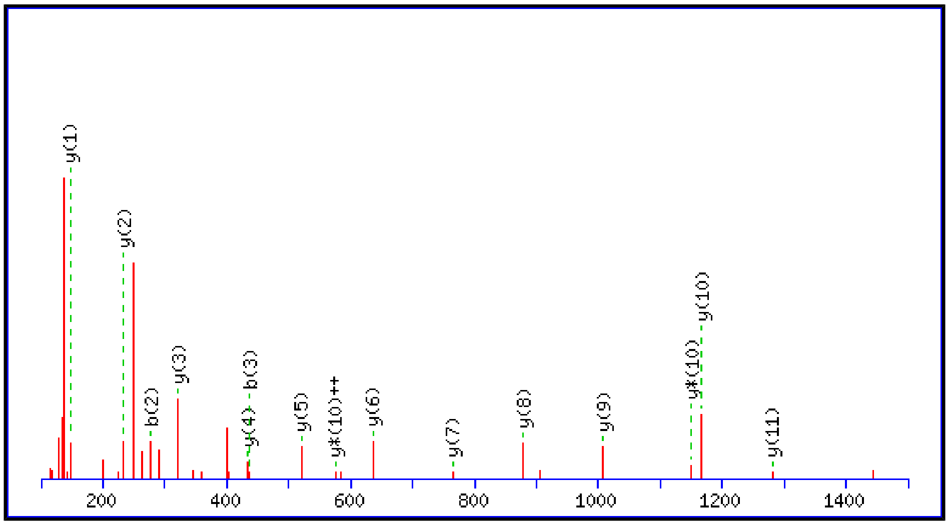

Monoisotopic mass of neutral peptide Mr(calc): 1442.6347
Variable modifications:
C3 : Carbamidomethyl (C)
Ions Score: 91 **Expect:** 1.8e-007
Matches : 15/116 fragment ions using 25 most intense peaks (help)

#	b	b^{++}	b*	b^{*++}	b^0	b^{0++}	Seq.	y	y^{++}	y*	y^{*++}	y^0	y^{0++}	#
1	164.0706	82.5389					Y							12
2	**277.1547**	139.0810					I	***1280.5787***	640.7930	1263.5522	632.2797	1262.5681	631.7877	11
3	**437.1853**	219.0963					C	***1167.4946***	584.2510	1150.4681	575.7377	1149.4841	575.2457	10
4	566.2279	283.6176			548.2173	274.6123	E	***1007.4640***	504.2356	990.4374	495.7224	989.4534	495.2304	9
5	680.2708	340.6391	663.2443	332.1258	662.2603	331.6338	N	***878.4214***	439.7143	861.3949	431.2011	860.4108	430.7091	8
6	808.3294	404.6683	791.3029	396.1551	790.3189	395.6631	Q	***764.3785***	382.6929	747.3519	374.1796	746.3679	373.6876	7
7	923.3564	462.1818	906.3298	453.6685	905.3458	453.1765	D	***636.3199***	318.6636	619.2933	310.1503	618.3093	309.6583	6
8	1010.3884	505.6978	993.3618	497.1846	992.3778	496.6925	S	***521.2930***	261.1501	504.2664	252.6368	503.2824	252.1448	5
9	1123.4725	562.2399	1106.4459	553.7266	1105.4619	553.2346	I	***434.2609***	217.6341	417.2344	209.1208	416.2504	208.6288	4
10	1210.5045	605.7559	1193.4779	597.2426	1192.4939	596.7506	S	***321.1769***	161.0921	304.1503	152.5788	303.1663	152.0868	3
11	1297.5365	649.2719	1280.5100	640.7586	1279.5259	640.2666	S	***234.1448***	117.5761	217.1183	109.0628	216.1343	108.5708	2
12							K	***147.1128***	74.0600	130.0863	65.5468			1

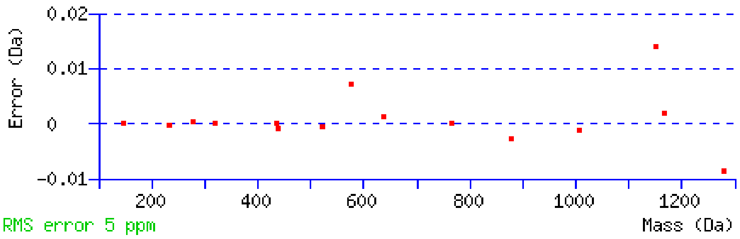

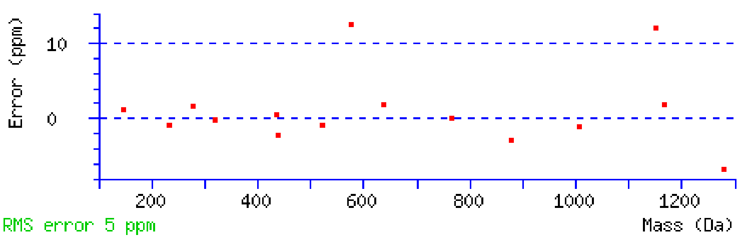

NCBI **BLAST** search of YICENQDSISSK
(Parameters: blastp, nr protein database, expect=20000, no filter, PAM30)
Other BLAST web gateways

All matches to this query

Score	Mr(calc):	Delta	Sequence
90.6	1442.6347	-0.0024	YICENQDSISSK

**Mascot:** http://www.matrixscience.com/

Mascot Search Results

Peptide View

MS/MS Fragmentation of **YICENQDSISSK**
Found in **sp|P02768|ALBU_HUMAN**, Serum albumin OS=Homo sapiens GN=ALB PE=1 SV=2

Match to Query 4921: 1442.634128 from(722.324340,2+) intensity(2154714.5000) scans(5731) rtinseconds(1147) index(4614)
Title: 150808_TTSH_Patient_Plasma_44_Spectrum020855_scans__5731_RTINSECONDS=1147
Data file L:\\Ard_TTSH\\T1D\\T150808_TTSH_Patient_Plasma_44.mgf

Click mouse within plot area to zoom in by factor of two about that point
Or, Plot from 100 to 1300 Da Full range
Label all possible matches ○ Label matches used for scoring ⊙

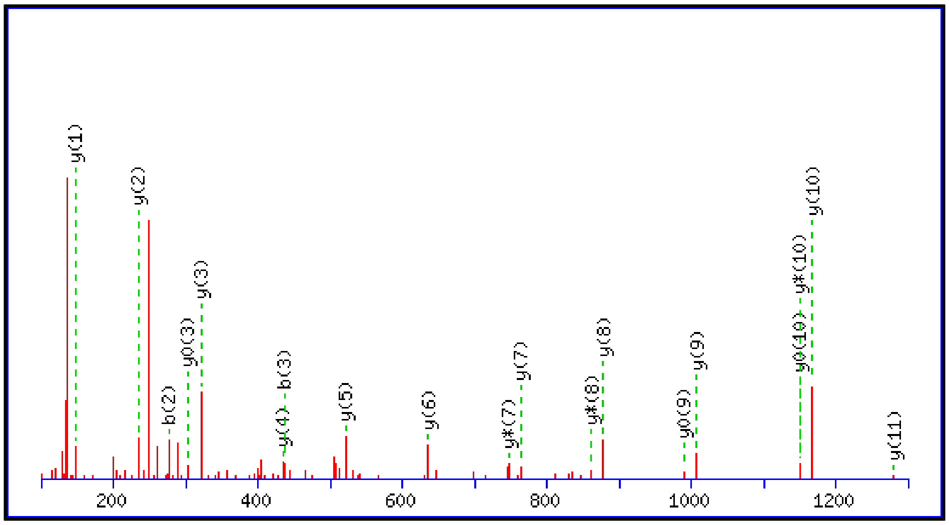

Monoisotopic mass of neutral peptide Mr(calc): 1442.6347
Variable modifications:
C3 : Carbamidomethyl (C)
Ions Score: 90 **Expect:** 2e-007
Matches : 19/116 fragment ions using 30 most intense peaks (help)

#	b	b^{++}	b*	b^{*++}	b^{0}	b^{0++}	Seq.	y	y^{++}	y*	y^{*++}	y^{0}	y^{0++}	#
1	164.0706	82.5389					Y							12
2	**277.1547**	139.0810					I	***1280.5787***	640.7930	1263.5522	632.2797	1262.5681	631.7877	11
3	**437.1853**	219.0963					C	***1167.4946***	584.2510	1150.4681	575.7377	1149.4841	575.2457	10
4	566.2279	283.6176			548.2173	274.6123	E	***1007.4640***	504.2356	990.4374	495.7224	989.4534	495.2304	9
5	680.2708	340.6391	663.2443	332.1258	662.2603	331.6338	N	***878.4214***	439.7143	861.3949	431.2011	860.4108	430.7091	8
6	808.3294	404.6683	791.3029	396.1551	790.3189	395.6631	Q	***764.3785***	382.6929	747.3519	374.1796	746.3679	373.6876	7
7	923.3564	462.1818	906.3298	453.6685	905.3458	453.1765	D	***636.3199***	318.6636	619.2933	310.1503	618.3093	309.6583	6
8	1010.3884	505.6978	993.3618	497.1846	992.3778	496.6925	S	***521.2930***	261.1501	504.2664	252.6368	503.2824	252.1448	5
9	1123.4725	562.2399	1106.4459	553.7266	1105.4619	553.2346	I	***434.2609***	217.6341	417.2344	209.1208	416.2504	208.6288	4
10	1210.5045	605.7559	1193.4779	597.2426	1192.4939	596.7506	S	***321.1769***	161.0921	304.1503	152.5788	303.1663	152.0868	3
11	1297.5365	649.2719	1280.5100	640.7586	1279.5259	640.2666	S	***234.1448***	117.5761	217.1183	109.0628	216.1343	108.5708	2
12							K	***147.1128***	74.0600	130.0863	65.5468			1

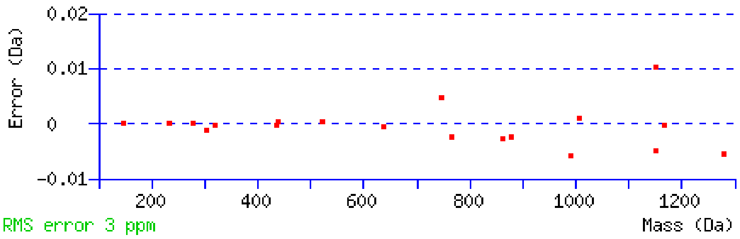

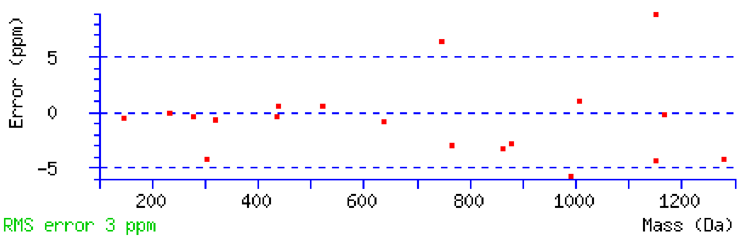

NCBI **BLAST** search of YICENQDSISSK
(Parameters: blastp, nr protein database, expect=20000, no filter, PAM30)
Other BLAST web gateways

All matches to this query

Score	Mr(calc):	Delta	Sequence
90.4	1442.6347	-0.0006	YICENQDSISSK
3.0	1441.6217	1.0124	EKPYKCEECEK
1.9	1442.6282	0.0059	YLCMATNAAGTDR

Mascot Search Results

Peptide View

MS/MS Fragmentation of **YICENQDSISSK**
Found in **sp|P02768|ALBU_HUMAN**, Serum albumin OS=Homo sapiens GN=ALB PE=1 SV=2

Match to Query 5103: 1442.633888 from(722.324220,2+) intensity(2842633.5000) scans(12547) rtinseconds(2261) index(10773)
Title: 150801_TTSH_Patient_Plasma_41_Spectrum027496_scans__12547_RTINSECONDS=2261
Data file L:\\Ard_TTSH\\T1D\\T150801_TTSH_Patient_Plasma_41.mgf

Click mouse within plot area to zoom in by factor of two about that point
Or, [Plot from] 100 to 1300 Da [Full range]
Label all possible matches ○ Label matches used for scoring ⊙

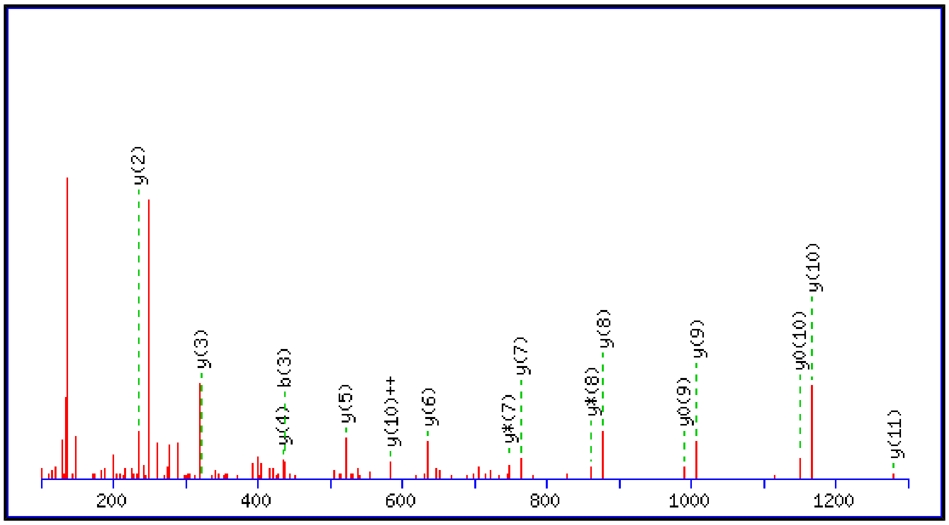

Monoisotopic mass of neutral peptide Mr(calc): 1442.6347
Variable modifications:
C3 : Carbamidomethyl (C)
Ions Score: 90 **Expect:** 2.1e-007
Matches : 16/116 fragment ions using 21 most intense peaks (help)

#	b	b^{++}	b*	b^{*++}	b^0	b^{0++}	Seq.	y	y^{++}	y*	y^{*++}	y^0	y^{0++}	#
1	164.0706	82.5389					Y							12
2	277.1547	139.0810					I	***1280.5787***	640.7930	1263.5522	632.2797	1262.5681	631.7877	11
3	**437.1853**	219.0963					C	***1167.4946***	584.2510	1150.4681	575.7377	1149.4841	575.2457	10
4	566.2279	283.6176			548.2173	274.6123	E	***1007.4640***	504.2356	990.4374	495.7224	989.4534	495.2304	9
5	680.2708	340.6391	663.2443	332.1258	662.2603	331.6338	N	***878.4214***	439.7143	861.3949	431.2011	860.4108	430.7091	8
6	808.3294	404.6683	791.3029	396.1551	790.3189	395.6631	Q	***764.3785***	382.6929	747.3519	374.1796	746.3679	373.6876	7
7	923.3564	462.1818	906.3298	453.6685	905.3458	453.1765	D	***636.3199***	318.6636	619.2933	310.1503	618.3093	309.6583	6
8	1010.3884	505.6978	993.3618	497.1846	992.3778	496.6925	S	***521.2930***	261.1501	504.2664	252.6368	503.2824	252.1448	5
9	1123.4725	562.2399	1106.4459	553.7266	1105.4619	553.2346	I	***434.2609***	217.6341	417.2344	209.1208	416.2504	208.6288	4
10	1210.5045	605.7559	1193.4779	597.2426	1192.4939	596.7506	S	***321.1769***	161.0921	304.1503	152.5788	303.1663	152.0868	3
11	1297.5365	649.2719	1280.5100	640.7586	1279.5259	640.2666	S	***234.1448***	117.5761	217.1183	109.0628	216.1343	108.5708	2
12							K	147.1128	74.0600	130.0863	65.5468			1

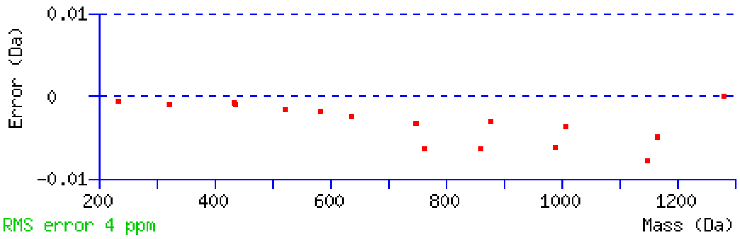

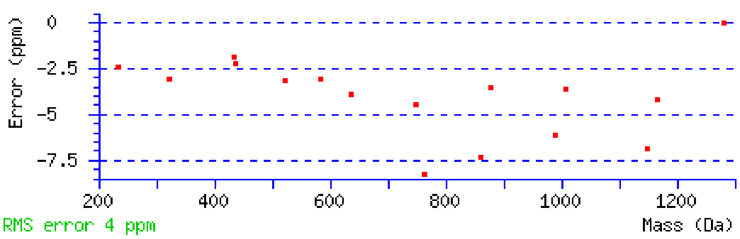

NCBI **BLAST** search of YICENQDSISSK
(Parameters: blastp, nr protein database, expect=20000, no filter, PAM30)
Other BLAST web gateways

All matches to this query

Score	Mr(calc):	Delta	Sequence
90.3	1442.6347	-0.0008	YICENQDSISSK
3.2	1441.6217	1.0122	EKPYKCEECEK
2.1	1442.6282	0.0057	YLCMATNAAGTDR

Mascot: http://www.matrixscience.com/
