# Supplementary material for: Serum albumin cysteine trioxidation is a potential oxidative stress biomarker of type 2 diabetes mellitus
Source: Sci Rep. 2020 Apr 15;10:6475. doi: 10.1038/s41598-020-62341-z (PMC7160123; doi:10.1038/s41598-020-62341-z)
Supplement: Supplementary file 5 — Supplementary Information 5. [file 41598_2020_62341_MOESM5_ESM.docx]

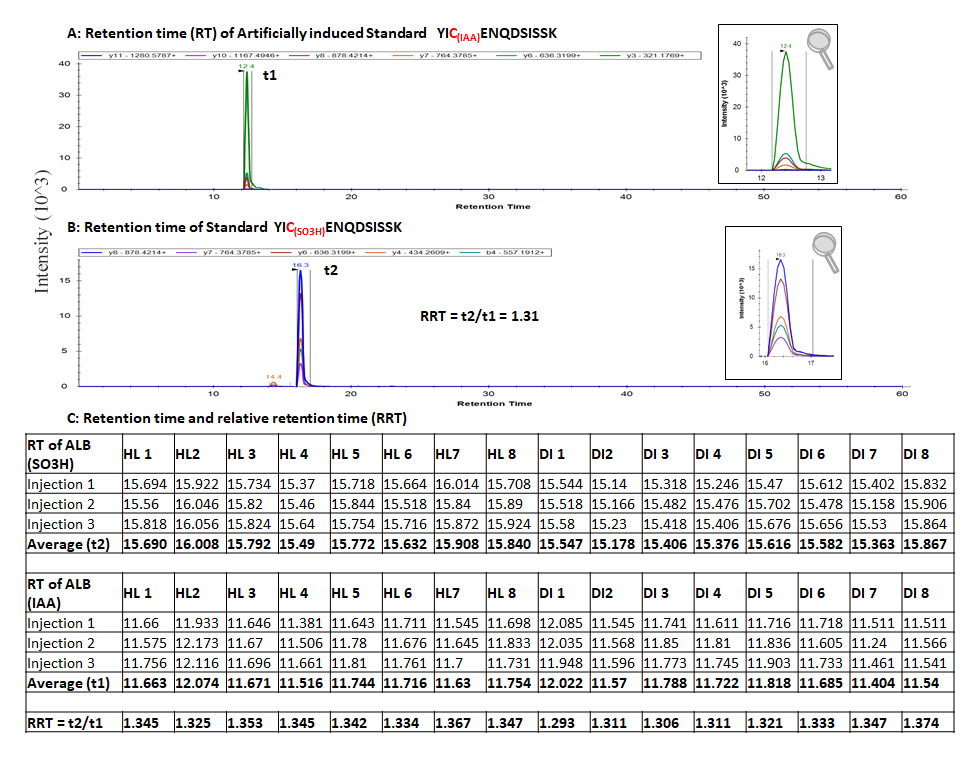


Figure S1. Spectrum, retention and relative retention of cysteine trioxidation and carbamidomethylation of the YICENQDSISSK peptides. Individual plasma samples of healthy subjects (n=8) and patients with diabetes (n=8) were analyzed in triplicate (HL=healthy, DI=diabetes).


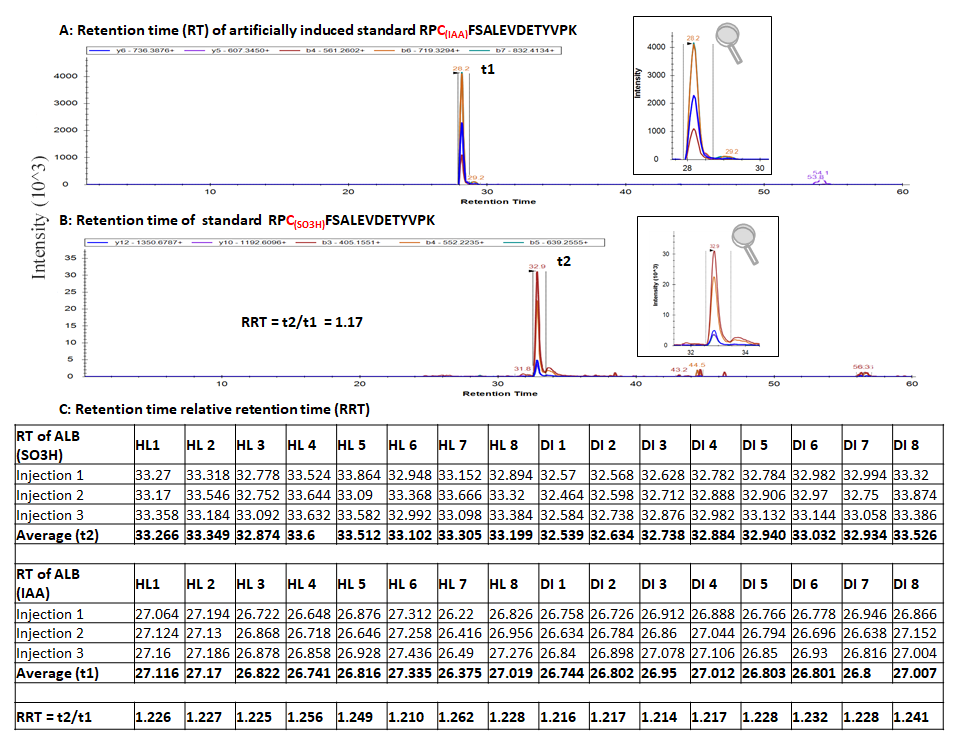


Figure S2. Spectrum, retention and relative retention of cysteine trioxidation and carbamidomethylation of the RPCFSALEVDETYVPK peptides. Individual plasma samples of healthy subjects (n=8) and patients with diabetes (n=8) were analyzed in triplicate (HL=healthy, DI=diabetes).

**
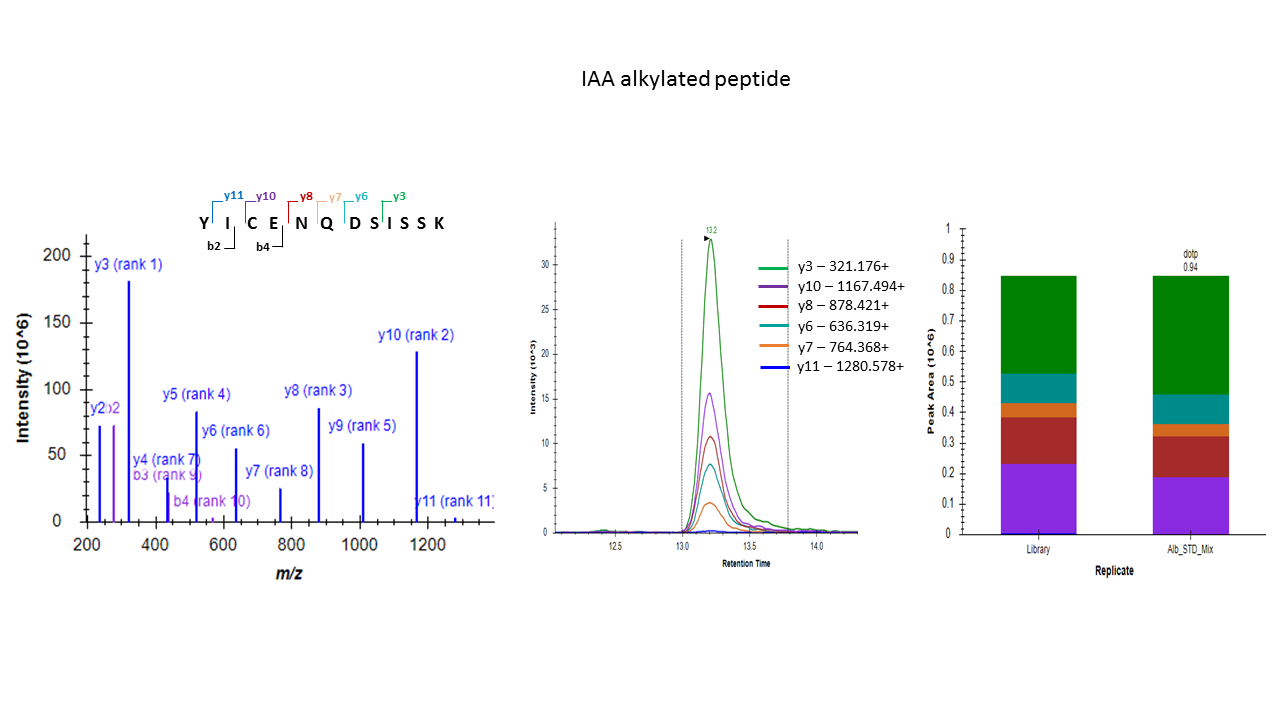
**

Figure S3. MS/MS spectrum, fragmentation pattern, and MRM dotp value (=0.94) of the YIC_(IAA)_ENQDSISSK peptide.


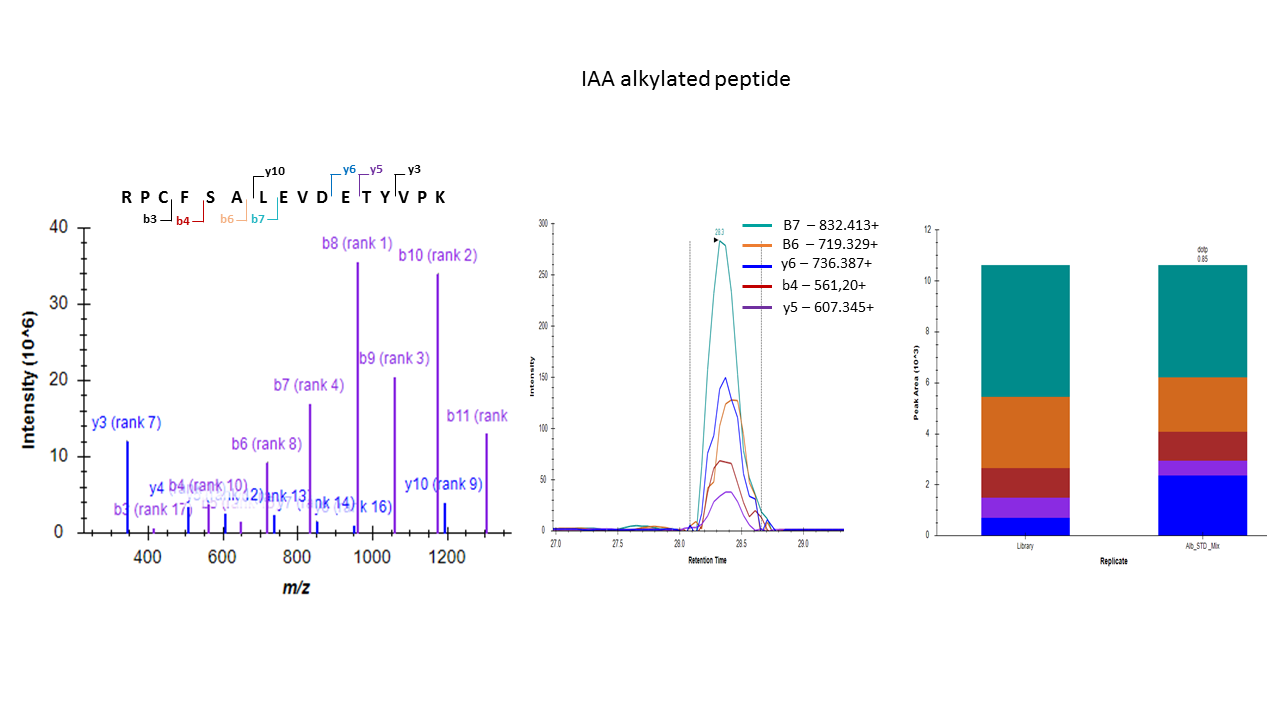


Figure S4. MS/MS spectrum, fragmentation pattern, and MRM dotp value (=0.85) of the RPC_(IAA)_FSALEVDETYVPK peptide.


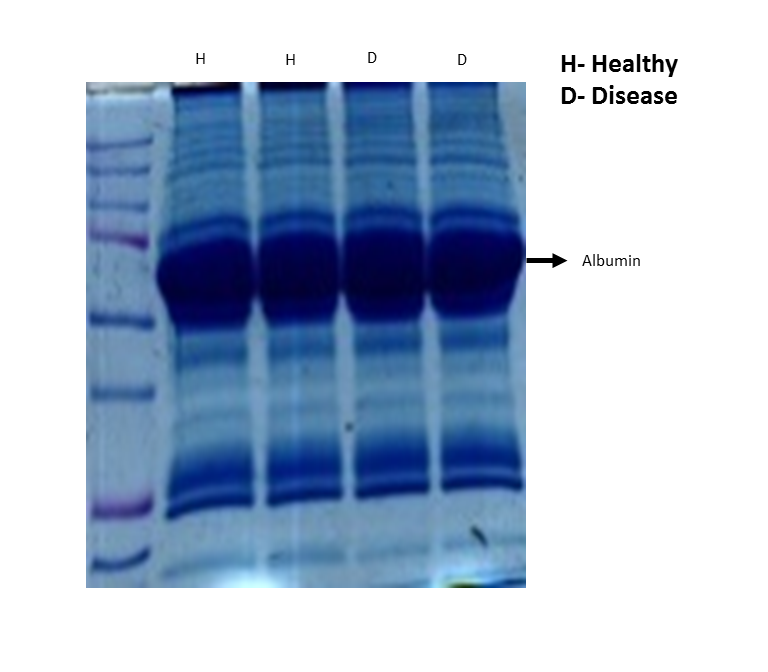


Figure S5 Plasma samples (100 µg/each) were separated on a 12% SDS-PAGE gel, and protein bands were visualized by staining with Coomassie blue. The albumin bands were excised for in-gel digestion and quantitative proteomics analysis.

Table S1. Details of the sequences, site of modification and other characteristics that were chosen for MRM analysis. The albumin trioxidation site is marked with *, while the corresponding carbamidomethyl site is marked with #.

| **Albumin peptide sequence** | **Precursor m/z** | **Precursor charge** | **Product ion** | **Fragment ion** | **Product charge** | **Collision energy** | **Measurement time [min]** |
| --- | --- | --- | --- | --- | --- | --- | --- |
| YIC*ENQDSISSK | 717.807 | 2+ | 434.262 | y4 | 1+ | 28 | 10 to 20 |
| YIC*ENQDSISSK | 717.807 | 2+ | 557.192 | b4 | 1+ | 28 | 10 to 20 |
| YIC*ENQDSISSK | 717.807 | 2+ | 636.32 | y6 | 1+ | 28 | 10 to 20 |
| YIC*ENQDSISSK | 717.807 | 2+ | 764.379 | y7 | 1+ | 28 | 10 to 20 |
| YIC*ENQDSISSK | 717.807 | 2+ | 878.422 | y8 | 1+ | 28 | 10 to 20 |
| YIC#ENQDSISSK | 722.325 | 2+ | 321.17 | y3 | 1+ | 28 | 6 to 16 |
| YIC#ENQDSISSK | 722.325 | 2+ | 636.32 | y6 | 1+ | 28 | 6 to 16 |
| YIC#ENQDSISSK | 722.325 | 2+ | 764.379 | y7 | 1+ | 28 | 6 to 16 |
| YIC#ENQDSISSK | 722.325 | 2+ | 878.422 | y8 | 1+ | 28 | 6 to 16 |
| YIC#ENQDSISSK | 722.325 | 2+ | 1167.494 | y10 | 1+ | 28 | 6 to 16 |
| YIC#ENQDSISSK | 722.325 | 2+ | 1280.578 | y11 | 1+ | 28 | 6 to 16 |
| RPC*FSALEVDETYVPK | 951.452 | 2+ | 405.156 | b3 | 1+ | 36 | 26 to 38 |
| RPC*FSALEVDETYVPK | 951.452 | 2+ | 552.224 | b4 | 1+ | 36 | 26 to 38 |
| RPC*FSALEVDETYVPK | 951.452 | 2+ | 639.256 | b5 | 1+ | 36 | 26 to 38 |
| RPC*FSALEVDETYVPK | 951.452 | 2+ | 1192.609 | y10 | 1+ | 36 | 26 to 38 |
| RPC*FSALEVDETYVPK | 951.452 | 2+ | 1350.687 | y12 | 1+ | 36 | 26 to 38 |
| RPC#FSALEVDETYVPK | 955.97 | 2+ | 561.261 | b4 | 1+ | 36 | 21 to 31 |
| RPC#FSALEVDETYVPK | 955.97 | 2+ | 719.33 | b6 | 1+ | 36 | 21 to 31 |
| RPC#FSALEVDETYVPK | 955.97 | 2+ | 832.41 | b7 | 1+ | 36 | 21 to 31 |
| RPC#FSALEVDETYVPK | 955.97 | 2+ | 607.34 | y5 | 1+ | 36 | 21 to 31 |
| RPC#FSALEVDETYVPK | 955.97 | 2+ | 736.38 | y6 | 1+ | 36 | 21 to 31 |
| ALVLIAFAQYLQQC*PFEDHVK | 1241.136 | 2+ | 498.267 | y4 | 1+ | 41 | 21 to 35 |
| ALVLIAFAQYLQQC*PFEDHVK | 1241.136 | 2+ | 871.430 | y7 | 1+ | 41 | 21 to 35 |
| ALVLIAFAQYLQQC*PFEDHVK | 1241.136 | 2+ | 1022.424 | y8 | 1+ | 41 | 21 to 35 |
| ALVLIAFAQYLQQC*PFEDHVK | 1241.136 | 2+ | 1278.541 | y10 | 1+ | 41 | 21 to 35 |
| ALVLIAFAQYLQQC*PFEDHVK | 1241.136 | 2+ | 1391.626 | y11 | 1+ | 41 | 21 to 35 |
| ALVLIAFAQYLQQC#PFEDHVK | 1245.647 | 2+ | 498.267 | y4 | 1+ | 41 | 19 to 35 |
| ALVLIAFAQYLQQC#PFEDHVK | 1245.647 | 2+ | 871.430 | y7 | 1+ | 41 | 19 to 35 |
| ALVLIAFAQYLQQC#PFEDHVK | 1245.647 | 2+ | 1031.461 | y8 | 1+ | 41 | 19 to 35 |
| ALVLIAFAQYLQQC#PFEDHVK | 1245.647 | 2+ | 1159.520 | y9 | 1+ | 41 | 19 to 35 |
| ALVLIAFAQYLQQC#PFEDHVK | 1245.647 | 2+ | 1278.587 | y10 | 1+ | 41 | 19 to 35 |
| ALVLIAFAQYLQQC#PFEDHVK | 1245.647 | 2+ | 1400.662 | y11 | 1+ | 41 | 19 to 35 |
